# Distribution and diversity of ROS-generating enzymes across the animal kingdom, with a focus on sponges (Porifera)

**DOI:** 10.1186/s12915-022-01414-z

**Published:** 2022-09-30

**Authors:** Olivia H. Hewitt, Sandie M. Degnan

**Affiliations:** School of Biological Sciences and Centre for Marine Science, University of Queensland, St Lucia, QLD 4072 Australia

**Keywords:** Antioxidants, Sponges, Evolution, Reactive oxygen species (ROS), Metazoans, Redox, Genomics, Signalling

## Abstract

**Background:**

Reactive derivatives of oxygen (reactive oxygen species; ROS) are essential in signalling networks of all aerobic life. Redox signalling, based on cascades of oxidation–reduction reactions, is an evolutionarily ancient mechanism that uses ROS to regulate an array of vital cellular processes. Hydrogen peroxide (H_2_O_2_) and superoxide anion (O_2_^•−^) are employed as signalling molecules that alter the oxidation state of atoms, inhibiting or activating gene activity. Here, we conduct metazoan-wide comparative genomic assessments of the two enzyme families, superoxide dismutase (SOD) and NADPH oxidases (NOX), that generate H_2_O_2_ and/or O_2_^•−^ in animals.

**Results:**

Using the genomes of 19 metazoan species representing 10 phyla, we expand significantly on previous surveys of these two ancient enzyme families. We find that the diversity and distribution of both the SOD and NOX enzyme families comprise some conserved members but also vary considerably across phyletic animal lineages. For example, there is substantial NOX gene loss in the ctenophore *Mnemiopsis leidyi* and divergent SOD isoforms in the bilaterians *D. melanogaster* and *C. elegans.* We focus particularly on the sponges (phylum Porifera), a sister group to all other metazoans, from which these enzymes have not previously been described. Within Porifera, we find a unique calcium-regulated NOX, the widespread radiation of an atypical member of CuZnSOD named Rsod, and a novel endoplasmic reticulum MnSOD that is prevalent across aquatic metazoans.

**Conclusions:**

Considering the precise, spatiotemporal specificity of redox signalling, our findings highlight the value of expanding redox research across a greater diversity of organisms to better understand the functional roles of these ancient enzymes within a universally important signalling mechanism.

**Supplementary Information:**

The online version contains supplementary material available at 10.1186/s12915-022-01414-z.

## Background

Reactive oxygen species (ROS) are widely known as toxic derivatives of oxygen that induce oxidative damage. Yet, aerobic organisms across the tree of life depend on ROS as signalling molecules for a vast array of life-sustaining cellular functions [1, 2]. Indeed, it is the readiness with which ROS react with neighbouring molecules that makes them both potentially toxic and particularly well-suited to form an integral part of the redox signalling network.

Redox is the loss and gain of electrons during oxidation–reduction reactions, which can both generate and consume ROS [1]. Thus, redox signalling may be generalised as a chain of redox relays that transfer electrons from one chemical species to the next, resulting in a cascade that carries a signal from receipt to response [3]. In turn, cellular ROS is tightly regulated by antioxidants that mediate localised redox states by scavenging ROS in reduction reactions and thus also function to prevent oxidative damage [4, 5]. The interaction of target proteins with ROS changes the oxidation state of those proteins, causing a conformational change (primarily thiol-based modifications; [6]) that can either inhibit or activate gene activity. For instance, redox signalling can mediate both the activation and inhibition of specific transcription factors, such as nuclear factor kb (NF-kb) that regulates the inflammatory response of an organism [7, 8].

**Fig. 1:**
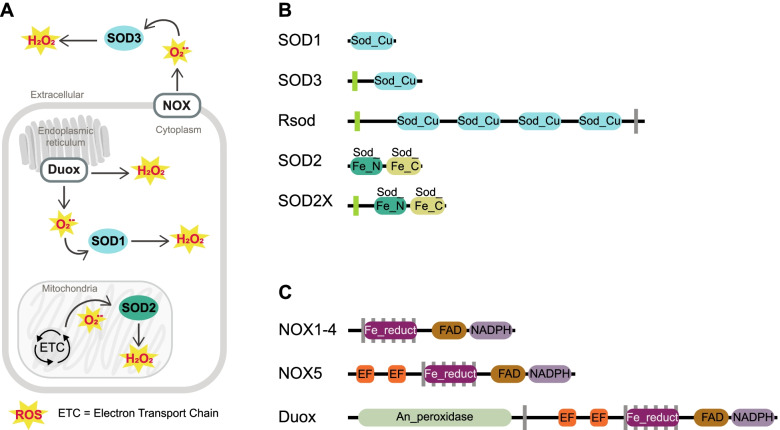
**A** Schematic representing enzymatic processes of H_2_O_2_ generation in animals. Incomplete reductive processes within the mitochondrial electron transport chain (ETC) generate a basal level of ROS, in accordance with the rate of metabolic activity which mitochondrial SOD2 converts into H_2_O_2_. Specialised transmembrane NOX enzymes produce relatively small and precise spatiotemporal fluctuations of ROS across membranes, either intra- or extracellularly. NOXs may either generate H_2_O_2_ directly or O_2_^•−^ that is subsequently converted into H_2_O_2_ either spontaneously or via activity of SODs localised within proximity, e.g. extracellular SOD3 or cytoplasmic SOD1. **B** Generalised domain structure for SOD enzyme families used in the identification of protein sequences. CuZnSOD enzymes comprise a copper/zinc binding domain (Sod_Cu; F00080; IPR001424), whilst MnSOD comprise a C-terminal Mn/Fe SOD domain (Sod_Fe_C; PF02777; IPR019832) and an N-terminal Mn/Fe SOD domain (Sod_Fe_N; PF00081; IPR019831). Green rectangles indicate signal peptides and grey rectangles indicate transmembrane regions. Domain structure for Rsod and SOD2X is variable from that depicted; the observed number of Cu_SOD domains within Rsod varied from 2 to 6, but in total, we found 43 sequences comprise at least three domains. Additionally, signal peptides observed on 35 Rsod sequences and transmembrane regions are found only in membrane-bound Rsods. Signal peptides are not present on all SOD2X sequences. **C** Generalised domain structure for NOX enzyme families used in the identification of protein sequences. All NOX enzymes comprise a Ferric reductase NAD binding_6 domain (purple; PF08030; IPR013121), FAD-binding_8 domain (brown; PF08022; IPR013112) and heme-containing ferric reductase transmembrane domain composed of 6 α-helices (Ferric_reduct; PF01794; IPR013121). The ferric reductase transmembrane domain, responsible for the electron transfer that generates O_2_^•−^, is distributed in a superfamily spanning both prokaryotic and eukaryotic lineages [17]. NOX5 and Duox additionally comprise calcium-sensitive EF-hand binding domains (orange), and Duox comprises animal haem peroxidase (green; PF03098; IPR019791)

One of the evolutionary oldest components of the redox signalling network is the antioxidant family known as superoxide dismutase (SOD) that targets a specific type of ROS known as superoxide anion radical (O_2_^•−^) [9]. Superoxide is the most readily formed ROS, since it requires only a one-electron reduction from ground state molecular oxygen (O_2_) [10, 11]. SOD arose prior even to the differentiation of eubacteria from archaea; its first function was to protect anaerobic organisms from ROS toxicity by scavenging O_2_^•−^ [12, 13]. Interestingly, as a by-product of the reaction to remove O_2_^•−^, SOD generates another, less reactive ROS that is today the predominant form of ROS used in redox signalling — this is hydrogen peroxide (H_2_O_2_) (Fig. 1A) [5, 10, 11]. Thus, SOD represents one of the most ancient mechanisms of enzymatic ROS generation that is still used today across all domains of life [14, 15]. Three types of SOD have been described in animals, namely cytoplasm SOD1 and extracellular SOD3 that use copper zinc (CuZn) as the electron acceptor and mitochondrial SOD2 that uses manganese (Mn) (Fig. 1B) [9, 12, 15, 16]. SOD is spatially localised within these compartments or extracellularly to limit O_2_^•−^ diffusion distances by converting it to H_2_O_2_, which in turn facilitates the tightly regulated and spatially oriented ROS generation that is required for redox signalling (Fig. 1A; reviewed by 15).

Two processes generate the majority of ROS in animals (reviewed in [5, 18]). First, incomplete reductive processes within the mitochondrial electron transport chain generate a basal level of ROS, in accordance with the rate of metabolic activity (Fig. 1A; reviewed in [19–21]). For a long time, this mitochondrial ROS was considered only as a toxic by-product of metabolism, but it is now understood that mitochondrial SOD facilitates tight regulation and spatial specificity of mitochondrial ROS required for redox signalling [1], reviewed by [15, 22]. Second, specialised transmembrane enzymes, NADPH oxidases (NOX), are known as “professional” ROS generators that produce relatively small and precise spatiotemporal fluctuations in either O_2_^•−^ or H_2_O_2_ when activated by specific signals (Fig. 1A) [23].

These NOX enzymes perform an essential role in the redox signalling network of animals. In mammals, there are currently seven described NOX subfamilies: NOX1-5 that generate O_2_^•−^, and two dual oxidases, Duox1 and Duox2, that can generate both O_2_^•−^ and H_2_O_2_ (Fig. 1C) [24]. All NOX family proteins share a structurally conserved region comprising six ferric reductase transmembrane domains, a single FAD-binding domain and a single ferric reductase NAD-binding domain (Fig. 1C) [25, 26]. NOX1-4 have only these three domain types and rely on additional subunits for their activation, except for NOX4 that is constitutively active. In contrast, NOX5 and Duox are Ca^2+^-activated via their calcium/calmodulin-sensitive EF-hand domains [27, 28] and Duox also has a single animal haem peroxidase domain (Fig. 1C) [29, 30]. Calcium-activated NOXs are considered the most evolutionarily ancient of the NOX proteins, with ancient subfamilies NOXC/D present in amoeba and algae, and the respiratory burst oxidase homolog (RBOH) present in plants [31, 32]. The activity of NOX allows for specialised generation of O_2_^•−^ that is tightly controlled and converted into H_2_O_2_ by the activity of SODs localised in close proximity (Fig. 1A). For instance, SOD-NOX generation of H_2_O_2_ has been specifically implicated in the activation of redox-sensitive transcription factors such as NF-Kb [33]. Despite the fundamental roles of SOD and NOX enzymes, they are generally poorly characterised outside of vertebrate, arthropod and nematode model species. To address this, here, we conduct a comparative genomic assessment of these two major redox signalling enzymes — SOD and NOX — across 19 metazoan species, encompassing 10 different phyla. Through multiple sequence alignment, domain architecture comparisons and phylogenetic analyses, we explore the distribution, diversity and conservation of these enzymes across the metazoan tree. We pay particular attention to sponges (phylum Porifera), by including genomes of five marine and one freshwater species belonging to 4 classes. Given that sponges originated at least 700 million years ago [34] and are considered the oldest of the extant animal phyletic lineages [35, 36], they are particularly valuable when viewed in a comparative framework. Their unique phylogenetic position as sister to all other animal phyla means that traits shared between sponges and the rest of the animal kingdom may logically be traced back to the last common animal ancestor [37]. By expanding awareness of redox signalling components across the metazoan tree, we provide the foundation for a broader understanding of the functional roles of these ancient enzymes within a universally important signalling mechanism.

## Results

### Superoxide dismutase (SOD)

**Fig. 2:**
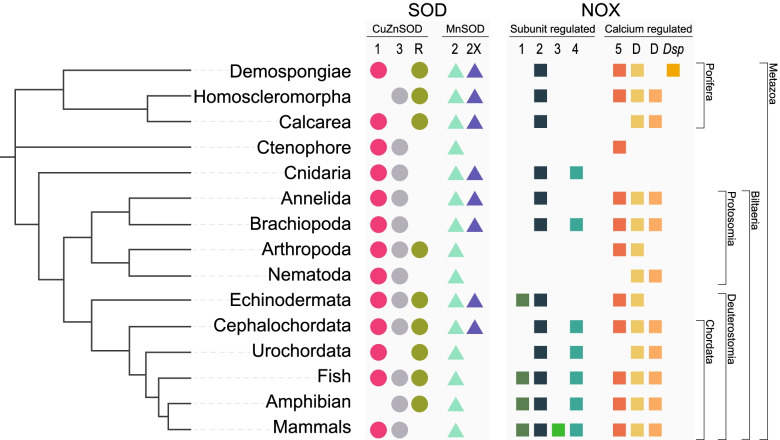
Cladogram summarising the distribution of SOD and NOX gene subfamilies across the major phyletic lineages assessed in this study. Numbers or letters at the top denote individual SOD or NOX subfamily members correlating with those displayed in SOD and NOX evolutionary trees

Across all 19 metazoan species, we identified a total of 149 unique protein sequences encoding at least one CuZnSOD domain and 50 containing both N and C terminal MnSOD domains. Filtering by characteristic domain structures (Fig. 1B) reduced this number to 113 CuZnSOD, whilst for MnSOD, we retained all 50 sequences (Table 1; Fig. 2; Additional file 2).

**Table 1:** Total counts of superoxide dismutase (SOD) enzymes identified from genome sequences of 19 metazoan species. Main numbers indicate the total number of unique sequences identified, including isoforms, splice variants and fragmented gene sequences. Superscript numbers indicate the number of additional identical protein sequences (exact sequence variants)

**Species**	**Phylum**	**CuZnSOD**	**MnSOD**	**Total**
*Amphimedon queenslandica*	Porifera	4	3	7
*Xestospongia bergquistia*	Porifera	5	3	8
*Tethya wilhelma*	Porifera	21	2	23
*Ephydatia muelleri*	Porifera	6	1	7
*Oscarella carmela*	Porifera	2	3	5
*Sycon ciliatum*	Porifera	13	2	15
*Mnemiopsis leidyi*	Ctenophora	8	1	9
*Nematostella vectensis*	Cnidaria	3	3	6
*Capitella teleta*	Annelida	5	3	8
*Lingula anatina*	Brachiopoda	4	6	10
*Drosophila melanogaster*	Arthropoda	4	1	5
*Caenorhabditis elegans*	Nematoda	7^2^	2^2^	13
*Strongylocentrotus purpuratus*	Echinodermata	4	2	6
*Acanthaster planci*	Echinodermata	6	1	7
*Branchiostoma floridae*	Chordata	6	1	7
*Ciona intestinalis*	Chordata	3	2	5
*Danio rerio*	Chordata	4	1	5
*Xenopus tropicalis*	Chordata	2^1^	1	4
*Homo sapiens*	Chordata	3	8^2^	13

**Fig. 3:**
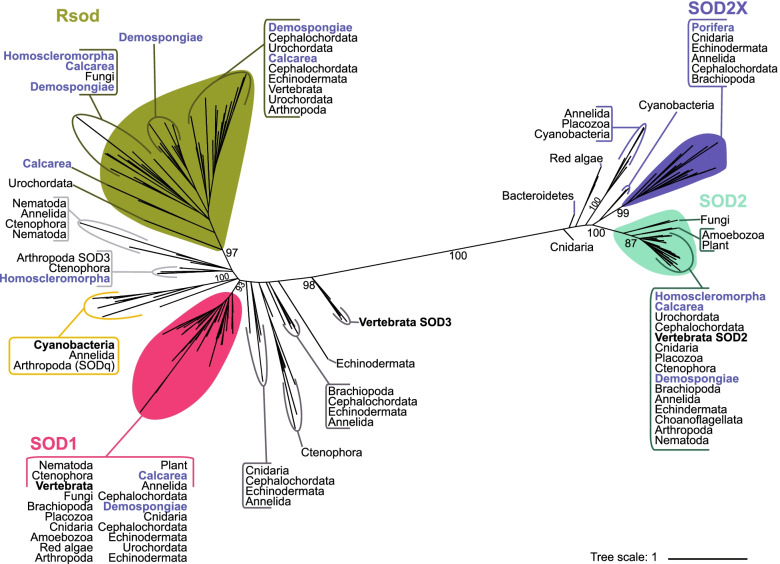
Maximum likelihood phylogenetic tree of SOD enzyme family. Unrooted tree displaying CuZn and Mn SOD sequences that are convergently arisen evolutionary groups. Filled in coloured shapes indicate monophyletic group; SOD1 (pink), SOD2 (turquoise), Rsod (green) and SOD2X (purple). The yellow box indicates the monophyletic group comprising CuZnSOD sequences from ancestral cyanobacteria, *Pseudanabaena *sp. and *Gloeobacter violaceus* [38]. SOD3 is paraphyletic and thus CuZnSOD sequences that lay outside one of these identified monophyletic groups may not easily be classified and are annotated here in two shades of grey, corresponding with Table 1. Paraphyletic cytoplasmic localised MnSOD sequences (pale yellow shape, grey dashed line). Labels in blue denote sequences encoded by phylum Porifera, named by class. Vertebrate SOD sequences are denoted in bold font. Black numbers on branches indicate bootstrap support. Constructed based on edited alignment, 1000 bb and the WAG + R6 evolutionary model

**Fig. 4:**
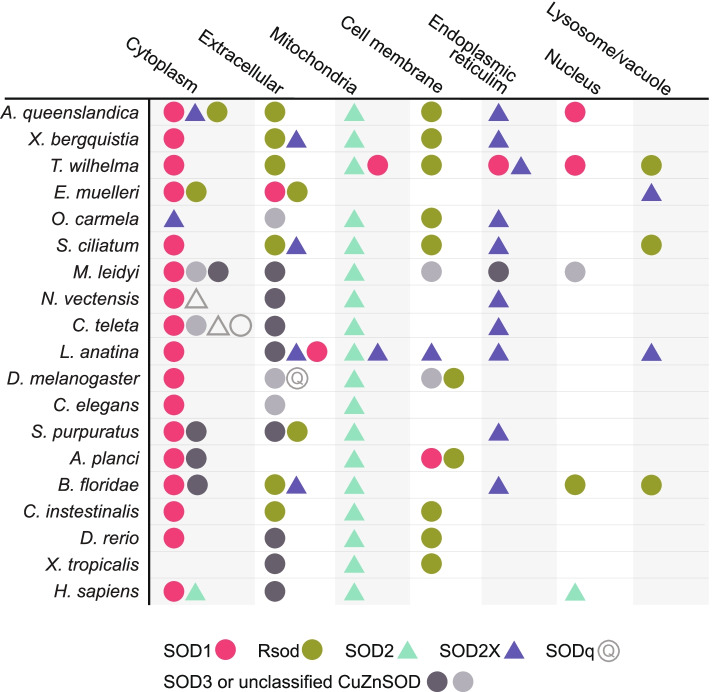
Presence of SOD antioxidant enzymes within 9 different subcellular compartments of 19 metazoan species. Shapes denote either CuZnSOD (circle) or MnSOD (triangle). Subcellular compartments indicated as column headings are predictions based on amino acid sequence analysis by DeepLoc-2.0, https://services.healthtech.dtu.dk/service.php?DeepLoc-2.0 [39]. Colours denote monophyletic clades SOD1, SOD2, Rsod and SOD2X, based on Fig. 2. SOD3 is denoted by two shades of grey that corresponds with the two phylogenetically distant paraphyletic groups in Fig. 2. Sequences that do not align with any particular group are not coloured. Total number of SOD genes encoded by each species in Table 1

Phylogenetic assessment of relationships amongst all 163 putative CuZnSOD and MnSOD genes revealed high support (> 97%) for four main monophyletic clades. These are cytoplasmic CuZnSOD (SOD1), an atypical CuZnSOD called Rsod, mitochondrial MnSOD (SOD2), and reported here for the first time, a predominantly endoplasmic reticulum (ER) localised MnSOD that we refer to as SOD2X (Figs. 1B and 3; Additional file 1 Fig. S1 & S2). In addition, 13 metazoans encode at least one additional CuZnSOD gene that appear to have arisen through multiple independent evolutionary events. For 11 species, these additional CuZnSOD sequences are predicted to localise extracellularly (Fig. 4). Although some of these sequences have been annotated previously as the extracellularly localised subfamily SOD3, they form independent branches that are evolutionary distant from each other rather than forming a monophyletic SOD3 clade (Fig. 3). Additionally, 11 of these sequences do not have a signal peptide, and 10 sequences (from seven species) encode for additional domains that are not typical of the SOD family. Thus, we do not classify these paraphyletic CuZnSOD sequences as SOD3. Notably, the ctenophore *Mnemiopsis leidyi* encodes eight CuZnSOD genes outside of the Rsod and SOD1 clades, of which six appear to represent a lineage-specific diversification (Fig. 3). Within phylum Porifera (sponges), only *Oscarella carmela* (class Homoscleromorpha) encodes one further CuZnSOD gene in addition to the subfamilies Rsod and SOD1.

Of the 50 putative MnSOD genes that we identified, 31 fall within a strongly supported (100%) monophyletic clade that includes previously described SOD2 sequences from the Vertebrata, Nematoda and Arthropoda [9, 40]; the majority of these were predicted with DeepLoc-2.0 to be localised to the mitochondria (Fig. 4; Additional file 3). The second strongly supported (99%) MnSOD monophyletic clade comprises 18 metazoan SOD2X sequences; DeepLoc-2.0 predicted 10 of these to be localised to the endoplasmic reticulum, five extracellularly, three to the lysosome/vacuole, two to the cytoplasm and one each to the cell membrane and mitochondria, where four sequences were predicted to localise within multiple subcellular compartments (Fig. 4; Additional file 3). Notably, the freshwater sponge, *Ephydatia muelleri*, is the only species that lacks a mitochondrial-localised SOD, but it does have a single MnSOD (SOD2X) that is predicted to localise within the lysosome/vacuole (Fig. 4).

We identified 51 putative CuZnSOD sequences with a domain architecture distinct from other described CuZnSODs (Fig. 1B). However, these sequences do share structural similarity with Rsod (Related to SOD), previously identified in *Drosophila melanogaster* (FBgn0051028; Dmel\CG31028) [40]. Together, these unusual CuZnSOD sequences form a strongly supported (97%) monophyletic clade (Fig. 3). Sequences falling within this monophyletic clade Rsod are distinct from SOD1 and SOD3 in comprising multiple Cu_SOD domains. Using that criterion, we find that 43 of the unusual CuZnSOD are putative Rsod sequences because they encode at least 3 Cu_SOD domains, and 26 comprise at least four. Also unusual, TargetP-2.0 predicted that 35 of these Rsod sequences contain a signal peptide region (Additional file 4). DeepLoc-2.0 predicted that 23 of the putative Rsod sequences are localised to the cell membrane, 21 extracellularly, 5 in the cytoplasm, 4 within the lysosome/vacuole and one within the nucleus; two sequences are localised to multiple compartments (Fig. 4; Additional file 3).

### NADPH oxidases (NOX)

Across all 19 metazoan species, we identified a total of 420 unique protein sequences that encode at least one NOX-associated domain, namely NAD-binding_6, FAD-binding_8, or ferric_reduct. Filtering by domain structure (Fig. 1C) characteristic of a NOX reduced this number to 143; of these, a further 25 sequences were removed because they lacked the full “HHHH” motif encoding O_2_^•−^ production (Table 2; Fig. 2; Additional file 2).

**Table 2:** Total counts of NADPH oxidise (NOX) enzymes identified from genome sequences of 19 metazoan species. Main numbers indicate the total number of unique sequences identified, including isoforms, splice variants and fragmented gene sequences. Superscript numbers indicate the number of additional identical protein sequences (exact sequence variants)

**Species**	**Phylum**	**Total**^a^	**NOX1**	**NOX2**	**NOX3**	**NOX4**	**NOX5**^**#**^	**Duox**
*Amphimedon queenslandica*	Porifera	**6**		1			3 (1)	1
*Xestospongia bergquistia*	Porifera	**3**		1			(1)	1
*Tethya wilhelma*	Porifera	**4**					2 (1)	1
*Ephydatia muelleri*	Porifera	**5**		1			(1)	3
*Oscarella carmela*	Porifera	**4**		1			1	2
*Sycon ciliatum*	Porifera	**6**		2				4
*Mnemiopsis leidyi*	Ctenophora	**1**					1	
*Nematostella vectensis*	Cnidaria	**3**		2		1		
*Capitella teleta*	Annelida	**5**		1			2	2
*Lingula anatina*	Brachipoda	**12**		1		1	6	4
*Drosophila melanogaster*	Arthropoda	**2**					1	1
*Caenorhabditis elegans*	Nematoda	**2**						2
*Strongylocentrotus purpuratus*	Echinodermata	**6**	1	1			3	1
*Acanthaster planci*	Echinodermata	**8**		1			6	1
*Branchiostoma floridae*	Chordata	**6**		1		1	1	3
*Ciona intestinalis*	Chordata	**8**		2		1		5
*Danio rerio*	Chordata	**7**	1	1		1	1	3
*Xenopus tropicalis*	Chordata	**9**	1	1^1^		3	1	2
*Homo sapiens*	Chordata	**25**	3	1	1	7^2^	6	4^1^

^a^Total numbers include gene duplications and fragmented genes

^#^Presence of *Dsp*NOX is included in count for column NOX5 within parenthesis

**Fig. 5:**
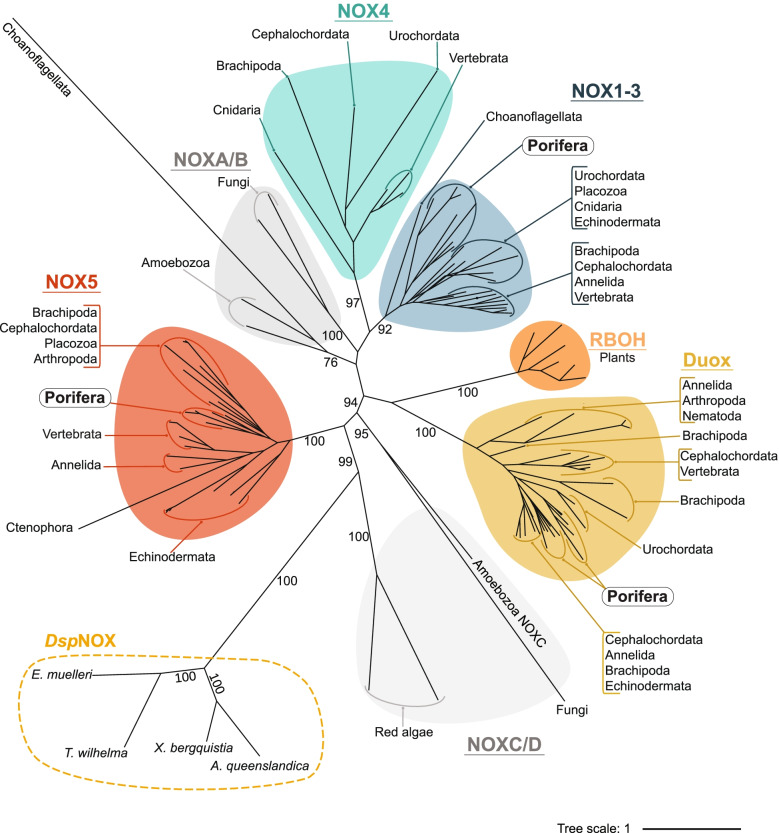
Maximum likelihood phylogenetic tree of NOX enzyme family. Unrooted tree displaying NOX subfamilies; NOX1-3 (blue circle), NOX4 (red circle), NOX5 (yellow circle), Duox (purple) and RBOH (green circle). Grey circles indicate subfamilies absent within metazoans. Including subunit-regulated NOXA/B and calcium-regulated NOXC/D. Dashed line surrounds sequences of novel calcium-regulated NOX encoded by species of class Demospongiae, named *Dsp*NOX. Black numbers on branches indicate bootstrap support. Branch lengths represent evolutionary distances, indicated by the tree scale. Tree displayed using 5 equal-daylight algorithm iterations to improve branch visibility. Constructed based on edited alignment, 1000 bb and the Dayhoff + F + R6 evolutionary model

Phylogenetic assessment of relationships amongst the 118 putative NOX genes revealed high support (92–100%) for four monophyletic clades, namely NOX1-3, NOX4, NOX5 and Duox (Fig. 5). Non-metazoan sequences fall into three previously identified groups, namely calcium-regulated NOXC/D, RBOH and subunit-regulated NOXA/B [31]*.* No single NOX subfamily is ubiquitously represented in all species assessed. In the NOX1-3 clade, seven genes encoding NOX2 sequences in sponges (phylum Porifera) together form a monophyletic clade, indicating diversification of these genes after the sponges diverged from the metazoan stem. However, within this larger group, NOX1 and NOX3 genes were identified only in vertebrates and echinoderms (NOX1), and mammals (NOX3) only (Table 2; Fig. 2). NOX4 was found in 7 of the 19 species, including the non-bilaterian *Nematostella vectensis* (phylum Cnidaria), but not in poriferans.

NOX2, NOX5 and Duox were the most common, found in 13 of 19 animal species. Indeed, phylum Porifera encode only these common NOX2, NOX5 and Duox subfamilies, although *Tethya wilhelma* (class Demospongiae) and *Sycon ciliatum* (class Calcarea) appear to lack NOX2 and NOX5, respectively. Although most metazoans encode only a single Duox, the sponges *O. carmella* (class Homoscleromorpha), *S. ciliatum* (class Calcarea) and *E. muelleri* (class Demospongiae) have multiple that appear to have derived via multiple independent/lineage-specific gene duplication events (Fig. 5). Indeed, species-specific duplication of NOX genes was commonly observed.

**Fig. 6:**
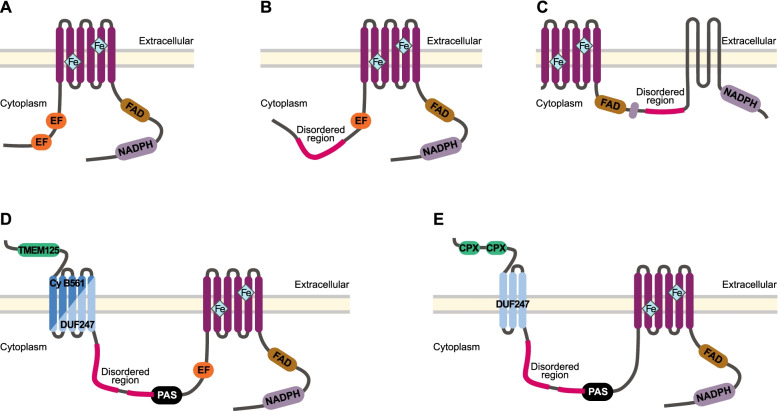
Structural representation of five subfamilies of calcium-regulated transmembrane NOX proteins. Displaying **A** NOX5 in animals, **B** NOXC in amoeba and **C** red algae, **D** *Dsp*NOX in marine sponges *A. queenslandica*, *X. bergquistia* and *T. wilhelma* and **E** *Dsp*NOX in freshwater sponge E*. muelleri*. Located at the c-terminal are the cytoplasmic NADPH-(purple, PF08030) and FAD (brown; PF08022) binding domains, leading to the heme-containing ferric reductase transmembrane domain composed of 6 α-helices (dark pink; PF01794), on which four conserved histidine residues bind two heme molecules. Together, these three domains form the canonical structure shared by all NOX enzymes. The presence of one or more **EF**-hand binding domains (orange) makes these subfamilies sensitive to activation via calcium/calmodulin binding, except for **C** red algae NOXC and in **E** *E. muelleri Dsp*NOX. Red algae NOXC is hypothesised to be calcium activated via alternative mechanisms [31], whilst *E. muelleri Dsp*NOX encodes two cupredoxin-blue copper binding domains (green). Long intrinsically disordered protein (IDP) regions are present in NOXC **B**, **C** and *Dsp*NOX **D** and **E**. Additionally, **D** and **E** *Dsp*NOX sequences comprise a cytoplasmic PAS domain (black; PF00989) and five (three in *E. muelleri*) additional transmembrane components. Transmembrane components in **D** sponges *A. queenslandica* and *X. bergquistia* are composed of eukaryotic cytochrome B561 (dark blue; PF03188) domains, whilst in sponges *T. wilhelma* and *E. muelleri* a domain of unknown function (light blue; DUF247; PF10348)

We identified four sequences in phylum Porifera, class Demospongiae, that did not group within any of the four main metazoan clades, although they cluster together with strong support (100%) (Fig. 5). Separated by large evolutionary distances, these sequences share some similarity to NOX5, possessing the three core NOX domains and EF-hand regions, but otherwise comprise a novel domain architecture (Fig. 6). We herein refer to these Demospongiae NOXs, described here for the first time, as *Dsp*NOX. It is the C-terminal region of *Dsp*NOX that has sequence and structural similarity to animal NOX5 (Fig. 6A), including a single calcium-binding EF-hand region. The exception is the freshwater species, *E. muelleri* that uniquely encodes two Cupredoxin domains at the C-terminal (predicted by Gene3D: CATH Superfamily 2.60.40.420; Fig. 6E). The N-terminal region of *Dsp*NOX comprises three additional structural features, conserved in all four demosponge species. These are a PAS or PAS_9 domain (PF00989; PF13426), a long intrinsically disordered protein (IDP), and in addition to the typical six alpha helixes, an additional 5 (except 3 in *E. muelleri*) transmembrane domain regions (Fig. 6D, E).

IDP regions are similarly observed in other unusual calcium-regulated NOXs of Amoebozoa and red algae, and the red algae NOXC/D clade that is a sister clade to *Dsp*NOX encodes four additional transmembrane regions (Fig. 6B, C) [31]. In the sponge species, *T. wilhelma* and *E. muelleri*, all five or three additional transmembrane regions are predicted to comprise a domain of an unknown function (DUF2427; PF10348). In contrast, for *Amphimedon queenslandica* and *Xestospongia bergquistia* (class Demospongiae), the lowest *e*-value score predicted that all five transmembrane domains comprise the eukaryotic cytochrome b561 (Cyb561; PF03188). However, significant matches for DUF2427 localised across the same five transmembrane regions were also observed in both these sponges. Notably, *E. muelleri* additionally encoded two other *Dsp*NOX-like sequences, but these lacked the HHHH motif for O_2_^•−^ production.

## Discussion

The ROS generators, SOD and NOX (including Duox), both represent ancient and large enzyme families that are widespread across the animal kingdom. Genes encoding both are present in all of the 19 metazoan species that we assessed (Tables 1 and 2; Fig. 2), but it is notable that the gene numbers vary considerably between species (from 1 to 25; Tables 1 and 2). Below we discuss the dynamic content of these gene families across the animal kingdom. For each of the two enzyme families, we draw upon our new findings from the phylum Porifera — considered sister to all other animal phyletic lineages — to provide new insights into the evolution of these gene families since the dawn of the Metazoa more than 700 million years ago.

### Superoxide dismutase (SOD)

The SOD family has been evolving for some ~ 2.5 (Ga) billion years of the Earth’s history, under vastly divergent selective pressures and with prolific cross-domain horizontal gene transfer events [15, 38, 41, 42]. In the animal kingdom alone, we observe various metalloforms and their subfamilies are not distributed equally across the metazoan tree, and neither are their localisations to various subcellular compartments. Consistent with previous analyses based on smaller numbers of animal taxa, we find that CuZn SOD1 and Mn SOD2 are the most conserved widespread SOD subfamily members [40, 43, 44]. However, the broader taxonomy that we present here extends the known SOD diversity by revealing the widespread metazoan prevalence of an atypical CuZnSOD, named Rsod, and an additional MnSOD subfamily member, SOD2X.

### Evolution of the SOD family

Amongst the CuZnSODs, it is widely accepted that cytoplasmic SOD1 arose first and later gave rise to the extracellular SOD3 with the addition of signal peptides [12, 40, 44, 45].Our analysis reveals that SOD1 forms a strongly supported monophyletic group comprising metazoan, fungi, plant, algae and amoebozoan species (93%), whilst SOD3 is paraphyletic. We found metazoan species commonly have at least one additional CuZnSOD outside of SOD1 or Rsod, but these sequences are diverse and do not necessarily classify as SOD3; not all sequences encode a signal peptide, and many possess additional domains not typically associated to CuZnSOD. Indeed, together, we found these unclassified CuZnSOD sequences with those previously annotated as SOD3 formed six independent monophyletic groups, and three further independent branches (Fig. 3).

Thus, our results strongly support the hypothesis that SOD3 has arisen via multiple, independent evolutionary events in different metazoan lineages [40, 44, 45]. Moreover, some of these “SOD3” branches are separated by considerable distances, and many are not localised extracellularly as is typically described for SOD3 in vertebrates, nematodes and arthropods [40, 44] (Fig. 4). Considering that localisation of SOD dictates its signalling pathway involvement, we suggest that these unclassified CuZnSOD sequences likely exhibit diverse functionality across the metazoan phyla. Given this, we further suggest that the term SOD3 is not sufficient to capture the complete diversity of paraphyletic CuZnSODs that have independently arisen, on multiple occasions across the metazoans.

Interestingly, most of the sponges we survey lack any additional CuZnSOD SOD3-like gene, which we suggest may be functionally substituted by the extracellular Rsod that is encoded by all sponge species except for *O. carmela* that does encode an extracellular SOD3. As with SOD1, the Rsod monophyletic clade comprises representatives from multiple kingdoms and thus supports previous suggestions that Rsod belongs to an ancient group of proteins [44]. Based on these observations, we propose that Rsod predates SOD3 and that this explains the predominant absence of SOD3 in phylum Porifera where Rsod is common.

Of the various SOD metalloforms, it has most commonly been hypothesised that MnSOD (homologous to non-animal FeSOD) is ancestral, with CuZnSOD arising later, after the great oxidation event (reviewed by [12, 15, 42]). Alternatively, [38] recently proposed instead that CuZnSOD first arose in the Archean, predating Fe/MnSOD that subsequently appeared much later in the mid-Proterozoic. In support of this, here, we find SOD1 is the closest monophyletic clade to CuZnSODs from Archean cyanobacterial lineages *Pseudanabaena *sp. and *Gloeobacter violaceus* [38] (Fig. 3). Moreover, unlike MnSOD2 where the metazoans form an independent monophyletic group (87%), the SOD1 clade comprises genes from multiple kingdoms, reflecting an ancient evolutionary origin that predates separation of the eukaryotic kingdoms (Fig. 3). SOD1 sequences are also more variably localised; they are found within the nucleus, ER, mitochondria, cell membrane and extracellularly across five metazoan species (Fig. 4; Additional file 3). Conversely, Mn SOD2 are consistently localised within the mitochondria, indicating a comparatively greater degree of conservation (Fig. 3).

We must also consider the prevalence of cross-domain horizontal gene transfer. Evolutionary analyses of bacterial SOD indicate CuZnSOD was lost as cyanobacteria diversified within new ecological niches [46] and then later spread via horizontal transmission between non-cyanobacterial phyla likely multiple times, resulting in the distribution found today [38, 42]. Thus, it seems possible the distribution of metazoan SODs may similarly have arisen via horizontal acquisition across the domains.

### An atypical CuZnSOD — Rsod — is widespread in the animal kingdom

We reveal a widespread metazoan presence of an atypical CuZnSOD, phylogenetically distant from both SOD1 and SOD3, that is largely localised either extracellularly or membrane bound (green shape, Fig. 3). These atypical CuZnSOD sequences share sequence and structural similarity to a *D. melanogaster* sequence named Rsod, “Related to SOD” (Dmel\CG31028, FBgn0051028), clustering together within a single, well-supported monophyletic clade [40] (Fig. 3). The structure of Rsod is unique from other known CuZnSODs, comprising multiple CuZnSOD domains (between 2 and 6) often with signal peptide and/or transmembrane regions (Fig. 1B). Rsod is considered to belong to an ancient group of proteins containing CuZnSOD homology domains [40], but has been little explored since its initial description, and its function is not yet known.

To date, Rsod sequences have been identified across the animal kingdom, including in insects, fish and the urochordate *Ciona intestinalis* and also in fungi [40, 45], BmSOD6, Accession Nos. LC229593 from 45]. Here, we additionally identify Rsod homologues within six sponges, echinoderms, cephalochordate (*Branchiostoma floridae*) and two vertebrates, indicating the widespread prevalence of this atypical CuZnSOD (Fig. 3). Notably, we show here for the first time that Rsod is particularly abundant and diversified within phylum Porifera, and especially within *T. wilhelma* (class Demospongiae) and *S. ciliatum* (class Calcarea)*.* Subcellular localisation predictions indicate that most Rsod sequences we identified are either extracellular (21 sequences) or cell membrane bound (23 sequences), except five sequences localised within the cytoplasm in sponges (1 in *A. queenslandica*, *X. bergquistia*, *E. muelleri* and 2 in *T. wilhelma*), four in the vacuole/lysome (1 in *B. floridae*, and *T. wilhelma* and 2 in *S. ciliatum*) and one within the nucleus (Fig. 4; Additional file 3). Intriguingly, five species of Porifera, the tunicate *C. intestinalis* and Ascomycota fungi *Phaeosphaeria nodorum*, that do not encode any other extracellular CuZnSOD (e.g. SOD3), all encode both extracellular and membrane forms of Rsod; the exception is *E. muelleri* that encodes a cytoplasmic Rsod instead of membrane bound. In contrast, *O. carmela*, *Acanthaster planci*, *Danio rerio*, *Xenopus tropicalis*, *D. melanogaster* and *Bombyx mori* (BmSOD6; Kobayashi et al. 2019), all of which do encode extracellular SOD3, have only the cell membrane localised form (Fig. 4; Additional file 3). Thus, we suggest the extracellular Rsod in these species may functionally replace an extracellular SOD3. That said, *B. floridae* and *Strongylocentrotus purpuratus* comprise only extracellular localised Rsod, despite also encoding extracellular SOD3 (Fig. 4).

To date, the possible function of Rsod remains unknown. Kobayashi et al. assessed the responsiveness of different SOD genes within silk moth (*B. mori*) under various oxidative stressors and found Rsod gene (BmSOD6) is mostly expressed within the testes on day 3 of fifth instar larvae, indicating a role in removing ROS generated during spermatogenesis [47]. Additionally, honeybees (*Apis mellifera*) exposed to caging stress for up to 4 weeks showed a constant upregulation of only two genes, namely Rsod and another antioxidant thioredoxin-1 (Trx-1) [48]. In crown of thorns starfish, *A. planci*, maintained in captivity, one Rsod homologue (gbr.190.13.t1) is upregulated in three tissues (skin, tube feet and spines), and a second homologue (gbr.190.14.t1) in skin only, compared to wild populations [49].

### The NADPH oxidase family

Much of the current understanding of NOX function is based on mammals, but variation in NOX subfamily membership across the metazoan and beyond indicates NOX functions are also likely to be variable. In accordance with 30 and 31, we find the calcium-regulated subfamilies have the earliest origin and are the widest distributed, followed by subunit-regulated NOXs that first appeared in fungi and amoeba, and for which we find the sponges (Porifera) have the earliest metazoan NOX2 co-ortholog (Fig. 5). However, neither the calcium- nor subunit-regulated NOXs are universally found in all metazoans, nor is any single NOX subfamily member. This is consistent with previous analyses based on smaller numbers of taxa [31, 32] showing large variability in gene number across the metazoan tree, indicative of lineage-specific gene duplication and apparent gene loss involving all NOX gene family members.

### There are no core NOX subfamilies present across the Metazoa

NOX gene duplication and losses have been commonly documented but are not yet well understood [32]. Whilst some species encode multiple (up to 7) sequences for a single NOX, others lack the subfamily entirely (Table 2). Different NOXs are documented to function with precise, spatiotemporal specificity (Nathan and Cunningham-Bussel 2013; Sies and Jones 2020). Thus, it is somewhat surprising to find extensive gene losses in *M. leidy* that comprises just a single NOX5, and *Caenorhabditis elegans *and* D. melanogaster* that each comprise only two calcium-regulated NOXs (Table 2; Fig. 2). Whilst plants similarly encode a single subfamily, RBOHs that are NOX5-like homologues, species possess up to 10 different members of these in the subfamily [31, 50]. This raises the question of how species may compensate the absence of key subfamily members, NOX2, NOX5 and Duox, that each have disparate described signalling roles [27, 29, 51].

One possibility is that where a single NOX subfamily is represented by multiple genes, each gene exhibits distinct spatio-temporal specificity. For instance, *S. ciliatum* and *C. intestinalis* encode multiple Duox enzymes but lack NOX5. Considering that both Duox and NOX5 may be activated by calcium-sensitive EF-hand domains, the multiple Duox genes may compensate for the missing NOX5 function, as has been predicted in rodents [52]. That said, *N. vectensis* (Cnidaria) lacks both these calcium-regulated NOXs but does encode a subunit-regulated NOX4 that is absent in other basal metazoans. An alternative possibility is that missing subfamily members may be compensated for by the existing NOX genes having a broader range of functions that depend on their spatiotemporal localisation. As an example, NOX2 was documented first in phagocytic cells of sea urchin and mouse oocytes, where it was determined to function in the “oxidative burst” during phagocytosis [53, 54] but since has been identified in diverse cell types, and with diverse functions [reviewed in 23].

The ecological niche of an organism also will impact its redox states [2, 55] and thus likely the redox machinery it requires. The ctenophore, *M. leidyi* that encodes a single NOX5, exhibits daily vertical migrations [56, 57], perhaps in response to high irradiance levels, that are known to influence localised redox states [58]. We propose that vertical migratory species such as *M. leidyi* may also be able to mediate O_2_^•−^ production via timing their daily migrations. Indeed, [59] show that antioxidant protection in cetaceans differs between shallow-diving and deep-diving habitats, as reflected by their O_2_^•−^ production and antioxidant levels [60]. The metabolic activity of vertical migrations may sufficiently influence ROS generation in the mitochondrial ETC, endoplasmic reticulum or peroxisome to compensate fewer NOX enzymes. For example, *M. leidyi* encodes a comparatively wide range of CuZnSODs that are localised to those ROS-generating subcellular compartments, which may reflect greater ROS generation during cellular and metabolic processes (Table 1; Figs. 2 and 3).

### Class Demospongiae encode a novel, calcium-regulated NOX

Within class Demospongiae (phylum Porifera), we identify here for the first time a structurally unique and phylogenetically distant NOX, which we call *Dsp*NOX (Fig. 6). The C-terminal region of *Dsp*NOX shares sequence and structural similarity with animal NOX5, comprising the core NOX domain features, and except for in the freshwater demosponge *E. muelleri*, is EF-hand calcium sensitive. *Dsp*NOX is ~ 700 residues longer than NOX5 and red algae NOXC/D and has a unique domain structure towards the N-terminal region that likely confers additional functionality and modes of activation.

Specifically, the N-terminal region of *Dsp*NOX comprises three novel elements (Fig. 6D, E). First, there is a PAS domain (PF00989) that functions as a sensory unit for diverse signals, including chemoreception, redox, photons and voltage, and thus acts as a highly versatile signal transducer [61]. It is well known as a light sensor, helping entrain the canonical circadian clock, but is also important in abiotic stress responses and innate immunity [62, 63]. The presence of both EF-hand and PAS suggests that, in addition to calcium, *Dsp*NOX could be regulated directly by abiotic factors. Second, *Dsp*NOX contains a long intrinsically disordered protein (IDP) region, defined as 30 or more consecutive disordered residues [64, 65]. The nature of IDP interactions (high specificity, but low affinity) increases a protein’s possible interactions and functional plasticity, making them well suited for signalling and regulatory functions [66]. Third, *Dsp*NOX contains five (or three within *E. muelleri*) additional transmembrane regions encoded within either a domain of unknown function DUF2427 (PF10348) or in the eukaryotic cytochrome b561 (Cyt-b561; PF03188).

Cyt-b561-containing proteins are an enzyme family of transmembrane, ascorbate-dependant oxidoreductases, most well known for their role in recycling ascorbate (i.e. vitamin C) via electron transfer from two heme b groups across the membrane [67]. Cyt-b561 enzymes may also be involved with iron metabolism, first described in the mammalian duodenal Cyt b561 (Dcytb), essential in the uptake of dietary nonheme iron (Fe^+3^) [68]. Dcytb uses ascorbate in the cytoplasm as an electron donor to reduce either Fe^+3^ into soluble, ferrous iron (Fe^+2^) or monodehydroascorbate (MDHA), depending on substrate availability [69]. Considering that the fenton reaction of Fe^+2^ with O_2_^•−^ generates the highly reactive hydroxyl radical (•OH), it is particularly interesting that *Dsp*NOX has the potential to produce both these molecules. Indeed, because of the significant damage that •OH causes if not contained, organisms have evolved under strong selection to keep ferrous iron and ROS apart [1]. Thus, we suggest that *Dsp*NOX may provide an adaptive mechanism to keep Fe^+2^ away from O_2_^•−^ if the ASC binding sites are on the same side as O_2_^•−^ generation.

NOX or pre-NOX genes are distributed right across all the eukaryotic supergroups, except for Rhizaria [17, 25]. It is hypothesised that an enzyme similar to the red algal NOXD gave rise to animal NOX5, via acquisition of calcium-binding motifs [31, 70, 71]. Interestingly, similar to *Dsp*NOX, red algal NOXD and amoebozoan NOXC also comprise long IDP regions, which thus may indicate ancestral calcium-regulated NOXs (Fig. 6B, C). Red algae similarly encode four additional transmembrane domains, although these are located between two NADPH-binding site sub-regions and thus predicted to function only as an anchor to the membrane [70]. Results from our phylogenetic analysis reveal that red algae NOXD is sister to *Dsp*NOX (Fig. 5). Although the possibility of long-branch attraction cannot be discounted, the described structural similarities of these two proteins and the consistent placement of NOXC/D with previous assessments [31] together suggest this relationship is unlikely to be an artefact. Thus, we propose that *Dsp*NOX also represents an ancient NOX lineage that is likely a lineage-specific innovation within the class Demospongiae (phylum Porifera).

## Conclusions

Across 10 metazoan phyla, we observe a very high level of conservation within certain SOD and NOX subfamilies. In particular, we find that SOD1 and SOD2, as well as NOX2, NOX5 and Duox, are widely distributed across the metazoans and form strongly supported monophyletic clades (Figs. 3 and 5). However, our broader coverage of metazoan phyla significantly expands the known diversity and distribution of these enzyme families, revealing several cases of gene loss, gene duplication and lineage-specific expansions. Importantly, our inclusion of sponges (phylum Porifera), considered sister to all other animal phyletic lineages, allows us to logically trace the distribution and characteristics of SOD and NOX families to the last common animal ancestor.

In sponges, we find all three of the major animal NOX subfamilies — NOX2, NOX5 and Duox — indicating the likely presence of these in the last common animal ancestor. That said, the ctenophore *M. leidyi* encodes only NOX5; under the alternative hypothesis that phylum Ctenophora is sister to all other animal phyletic lineages, then NOX5 but not Duox or NOX2 were likely present within the last common animal ancestor. NOX4 is absent from sponges and appears to have arisen later, after sponges diverged from the animal stem, but before the divergence of the cnidarian phyletic lineage. We also reveal the presence of a novel, calcium-regulated NOX, namely *Dsp*NOX, in class Demospongiae only. *Dsp*NOX, together with previously identified, unusual red algae and amoebozoan NOXC/D, reflects the broader structural and likely functional diversity of calcium-regulated NOXs amongst diverse species.

MnSODs comprise two strongly supported metazoan subfamilies, namely mitochondrial SOD2 and another predominantly extracellular clade, SOD2X, that we identified within all sponges. The CuZnSODs also comprise two strongly supported metazoan subfamilies, namely Rsod and SOD1. In contrast, SOD3 is paraphyletic and its absence in sponges supports the hypothesis that SOD3 arose sometime later after SOD1. The atypical CuZnSOD, called Rsod, is found within all six sponges and likely represents an ancient protein group that also predates SOD3. The SOD1 clade includes genes from all six sponges, and also genes from amoebozoans, plants and red algae, indicating a much deeper origin, for SOD1, earlier in eukaryotic evolution. Evidently, the evolution of the SOD family is complex with much remaining unclear. However, the diversity and lineage-specific divergences we observe across the animal kingdom illustrate the presence of highly specialised SODs in redox signalling networks of diverse animals with diverse ecologies.

The comparatively greater metazoan diversification and subfamily distribution of SOD than NOX is indicative of the deeper evolutionary origin of the former. However, both these enzyme families are integral to ancient systems of redox signalling and oxidative defence. Thus, the observed evolutionary relationships also reflect the considerably variable, species-specific life-history trade-offs between redox signalling and ROS toxicity.

## Methods

### Enzyme identification and subfamily classification

To search for gene sequences encoding candidate members of the SOD and NOX enzyme families, we assessed protein-coding sequences for 19 metazoan species of 10 phyla, with representation from each of the major clades, namely non-bilaterian, protostome bilaterians and deuterostome bilaterians (Additional file 1: Table S1). Coding sequences were scanned against the Pfam database using hmmscan in HMMR v3.1b2 (hmmer.org) for sequences encoding domains specific to each enzyme family and their respective subfamilies (Fig. 1B, C) [72], and the number of and position of all identified domains was determined. For all identified candidate gene sequences, protein subcellular localisation was predicted using DeepLoc-2.0, https://services.healthtech.dtu.dk/service.php?DeepLoc-2.0 [39, 73], that uses protein sequences as input for the Neural Networks algorithm trained on Uniprot proteins with experimental evidence. The algorithm incorporates the importance (“attention”) of particular amino acids and those neighbouring within the region. Positions in the sequence with high “attention” give more weight to the final prediction of the model. DeepLoc-2.0 is able to predict proteins that are located in more than one compartment. The criterion for deciding subcellular localisation is based on probability scores surpassing thresholds (set by 72) or if no score crosses the threshold, the label closest to the threshold is chosen.

The methodology for enzyme identification and subcellular localisation was cross-validated by comparing the number and type of SOD and NOX genes identified through our analysis with those that have previously been described. Candidate SOD enzyme sequences were assigned to either CuZnSOD or MnSOD based on the presence of domains for either copper/zinc binding (PF00080) or manganese/iron SOD C- and N-terminals (PF02777 and PF00081), respectively (Fig. 1B). For 50 CuZnSOD encoding sequences that clustered separately from the previously described SOD1 or SOD3 lineages, we predicted the presence of signal peptides using TargetP-2.0, https://services.healthtech.dtu.dk/service.php?TargetP-2.0 [74].

Candidate NOX enzyme sequences encoding the domains ferric reductase (PF01794) and at least one of either the FAD- (PF08022) or NAD-binding (PF08030) domains (Fig. 1C) were retained and scanned for the presence of the conserved NOX motif, His^101^, His^115^, His^209^, His^222^ (based on *Homo sapiens* NOX2 numbering), required for O_2_^•−^ generation [75]. NOX5 and Duox encoding sequences were initially classified based on the presence of EF-hand regions (both NOX5 and Duox) and of the domain for animal haem peroxidase (PF03098; Duox only; Fig. 1B). For four sequences identified in phylum Porifera, class Demospongiae, EF-hand regions were not initially identified in the hmmscan used as described above, but subsequently were predicted based on Gene3D ontology (http://www.cathdb.info/), and later manually assessed following MAFFT version 7.455 alignment of EF-hand domain regions from Pfam seed sequences. Additionally, for these same four sequences, intrinsically disordered protein (IDP) regions were predicted using IUPred3 (https://iupred.elte.hu/).

Multiple sequence and phylogenetic assessment.

Identified candidate sequences within each enzyme family were aligned using MAFFT version 7.455 ([76, 77], https://mafft.cbrc.jp/alignment/software/) with default parameters and visualised in the multiple sequence alignment editor, AliView [78].

To assess phylogenetic relationships, alignments were manually edited in AliView v1.27 ([78]; https://ormbunkar.se/aliview), removing regions containing more than 50% gaps, then imported to IQ-TREE [79] to construct maximum likelihood trees using ultrafast bootstrap [80], based on 1000 bb, and the most appropriate evolutionary model as identified by ModelFinder [81]. Models identified and used to construct each gene family tree were as follows: SOD, WAG + R6, NOX, Dayhoff + F + R6, MnSOD only, WAG + I + G4, and CuZnSOD only, WAG + R5. Phylogenetic trees were first visualised in iTOL v.6.2.1 [82] before importing and annotating within Adobe Illustrator. Classification of SOD and NOX gene subfamilies was inferred from the relative placing of putative sequences within known subfamily clades of phylogenetic trees. To provide evolutionary context to the metazoan phylogenetic relationships, we also included sequences obtained from organisms outside the metazoans that have previously been described (Additional file 1: Table S2).

## Supplementary Information

**Additional file 1:** **Table S1.** Details and availability of the 19 metazoan genomes used in this study. **Table S2.** Details and availability of non-metazoan genomes used in this study. **Fig S1.** Maximum likelihood phylogenetic tree of CuZnSOD enzyme family. **Fig S2.** Maximum likelihood phylogenetic tree of MnSOD enzyme family.

**Additional file 2.** Table displaying total number of sequences identified within the genomes of each metazoan species based on the presence of characteristic domains for NOX, CuZnSOD, and MnSOD proteins.

**Additional file 3.** Output from DeepLoc-2.0 analysis predicting subcellular localisation for identified CuZnSOD and MnSOD proteins.

**Additional file 4.** Output from TargetP-2.0 analysis predicting presence of signal peptide target region.

**Additional file 5.** All alignments and original protein sequences used to generate phylogenetic trees.

## Data Availability

All data generated or analysed during this study are included in this published article, its supplementary information files and publicly available repositories.

## Abbreviations

CuZnSOD: Copper zinc superoxide dismutase
Duox: Dual oxidase
ER: Endoplasmic reticulum
FeSOD: Iron superoxide dismutase
IDP: Intrinsically disordered protein
MnSOD: Manganese superoxide dismutase
NF-kb: Nuclear factor kb
NOX: NADPH oxidase
RBOH: Respiratory burst oxidase homolog
ROS: Reactive oxygen species
Rsod: Related to superoxide dismutase
SOD: Superoxide dismutase
