# Supplementary material for: Distribution and diversity of ROS-generating enzymes across the animal kingdom, with a focus on sponges (Porifera)
Source: BMC Biol. 2022 Sep 30;20:212. doi: 10.1186/s12915-022-01414-z (PMC9524095; doi:10.1186/s12915-022-01414-z)
Supplement: Supplementary file 1 — Additional file 1: Table S1. Details and availability of the 19 metazoan genomes used in this study. Table S2. Details and availability of non-metazoan genomes used in this study. Fig S1. Maximum likelihood phylogenetic tree of CuZnSOD enzyme family. Fig S2. Maximum likelihood phylogenetic tree of MnSOD enzyme family. [file 12915_2022_1414_MOESM1_ESM.pdf]

Table S1. Details of the genomes used in this study.

Species	Phyla	Website/source
Amphimedon queenslandica	Porifera	http://metazoa.ensembl.org/Amphimedon_queenslandica/Info/Index
Xestospongia bergquistia	Porifera	Data available from the authors on request
Tethya wilhelma	Porifera	http://tethya.spongebase.net/
Ephydatia muelleri	Porifera	https://spaces.facsci.ualberta.ca/ephybase/
Oscarella carmela	Porifera	http://compagen.unit.oist.jp/index.html
Sycon ciliatum	Porifera	http://compagen.unit.oist.jp/index.html
Mnemiopsis leidyi	Ctenophora	https://research.nhgri.nih.gov/mnemiopsis/
Nematostella vectensis	Cnidaria	https://www.ebi.ac.uk/ena/browser/view/GCA_000209225.1?show=blobtoolkit
Acanthaster planci	Echinoderm	https://marinegenomics.oist.jp/cots/viewer/info?project_id=46
Strongylocentrotus purpuratus	Echinoderm	https://www.ebi.ac.uk/ena/browser/view/GCA_000002235.4?show=blobtoolkit
Branchiostoma floridae	Chordata	https://mycocosm.jgi.doe.gov/Brafl1/Brafl1.home.html
Ciona intestinalis	Chordata	https://www.ebi.ac.uk/ena/browser/view/GCA_000224145.1
Capitella teleta	Annelida	https://www.ebi.ac.uk/ena/browser/view/GCA_000328365.1?show=blobtoolkit
Lingula anatina	Brachipoda	https://www.ebi.ac.uk/ena/browser/view/GCA_001039355.1
Caenorhabditis elegans	Nematoda	https://downloads.wormbase.org/releases/WS279/species/c_elegans/PRJNA13758/
Drosophila melanogaster	Arthropoda	https://flybase.org/ (release dmel_r6.32 (FB2020_01))
Danio rerio	Chordata	https://www.ncbi.nlm.nih.gov/grc/zebrafish
Xenopus tropicalis	Chordata	http://www.xenbase.org/
Homo sapiens	Chordata	https://www.ncbi.nlm.nih.gov/grc/human

Table S2. Details of sequences used from species for evolutionary comparison. Sequences were obtained from either the UniProt data base https://www.uniprot.org/ or NCBI https://www.ncbi.nlm.nih.gov/

**Species**	**Phylum**	**Gene ID**	**Uniprot accession**
Pseudanabaena.ABRG5-3	Cyanobacteria	CuZnSOD	A0A2Z5WSM0
Pseudanabaena.PCC7367	Cyanobacteria	CuZnSOD	K9SG01
Pseudanabaena.PCC7429	Cyanobacteria	CuZnSOD	L8N810
Pseudanabaena.SR411	Cyanobacteria	CuZnSOD	A0A256BEG9
Pseudanabaena.ABRG5-3.sodB	Cyanobacteria	MnSOD	A0A2Z5WSL4
Pseudanabaena.PCC7367	Cyanobacteria	MnSOD	K9SFI2
Pseudanabaena.PCC7429	Cyanobacteria	MnSOD	L8MYC0
Pseudanabaena.SR411	Cyanobacteria	MnSOD	A0A256BCV0
Gloeobacter.kilaueensis.JS1.SODC	Cyanobacteria	CuZnSOD	U5QI46
Gloeobacter.kilaueensis.JS1.GKIL_3379	Cyanobacteria	CuZnSOD	U5QPK5
Gloeobacter.violaceus.PCC7421.glr2170	Cyanobacteria	CuZnSOD	Q7NIL3
Gloeobacter.violaceus.PCC7421.glr1981	Cyanobacteria	CuZnSOD	Q7NJ51
Gloeobacter.violaceus.PCC7421.sodB	Cyanobacteria	MnSOD	Q7NDA9
Gloeobacter.kilaueensis.JS1.sodA	Cyanobacteria	MnSOD	U5QI99
Gloeobacter.violaceus.PCC7421.sodB	Cyanobacteria	MnSOD	Q7NMT3
Algoriphagus machipongonensis	Bacteroidetes	MnSOD	A3HUU8
Arabidopsis thaliana	Spermatophyta	CuZnSOD_1	P24704
Arabidopsis thaliana	Spermatophyta	CuZnSOD_2	O78310
Arabidopsis thaliana	Spermatophyta	RBOHD	Q9FIJ0
Arabidopsis thaliana	Spermatophyta	RBOHF	O48538
Arabidopsis thaliana	Spermatophyta	RBOHC	O81210
Arabidopsis thaliana	Spermatophyta	RBOHB	Q9SBI0
Arabidopsis thaliana	Spermatophyta	RBOHA	O81209
Arabidopsis thaliana	Spermatophyta	RBOHE	O81211
Podospora anserina	Ascomycota (filamentous fungus)	CuZnSOD_1	Q711T9
Podospora anserina	Ascomycota (filamentous fungus)	CuZnSOD_2	B2ATY8
Podospora anserina	Ascomycota (filamentous fungus)	MnSOD_1	Q711T6
Podospora anserina	Ascomycota (filamentous fungus)	MnSOD_2	Q9C208
Podospora anserina	Ascomycota (filamentous fungus)	NOX3(C/D)	B2AAS7
Podospora anserina	Ascomycota (filamentous fungus)	NOX2 (A/B)	B2AL10
Podospora anserina	Ascomycota (filamentous fungus)	NOX1 (A/B)	B2AA06
Phaeosphaeria nodorum	Ascomycota (Sac fungi)	CuZnSOD_1	Q0UEY3
Phaeosphaeria nodorum	Ascomycota (Sac fungi)	CuZnSOD_2	Q0UPE0
Phaeosphaeria nodorum	Ascomycota (Sac fungi)	CuZnSOD_3	Q0UD72
Phaeosphaeria nodorum	Ascomycota (Sac fungi)	CuZnSOD_4	Q0UMZ2
Chondrus crispus	Red algae (Irish moss)	CuZnSOD_1	R7QQ00

Chondrus crispus	Red algae (Irish moss)	CuZnSOD_2	R7QRY5
Chondrus crispus	Red algae (Irish moss)	MnSOD_1	R7QCE3
Chondrus crispus	Red algae (Irish moss)	MnSOD_2	R7QI02
Chondrus crispus	Red algae (Irish moss)	NOX_rboh (C/D)	Q2FA46
Pyropia yezoensis	Red algae (glume blotch)	MnSOD	Q3YB09
Pyropia yezoensis	Red algae (glume blotch)	NOX_rboh (C/D)	Q2F9N3
Dictyostelium discoideum	Amoebozoa	CuZnSOD_1	Q55GQ5
Dictyostelium discoideum	Amoebozoa	CuZnSOD_2	Q54TU5
Dictyostelium discoideum	Amoebozoa	CuZnSOD_3	Q54G70
Dictyostelium discoideum	Amoebozoa	CuZnSOD_4	Q54TW8
Dictyostelium discoideum	Amoebozoa	MnSOD	Q55BJ9
Dictyostelium discoideum	Amoebozoa	NOXA	Q9XYS3
Dictyostelium discoideum	Amoebozoa	NOXB	Q86GL4
Dictyostelium discoideum	Amoebozoa	NOXC	Q54F44
Fonticula alba	Amoebozoa	CuZnSOD_1	A0A058ZE05
Monosiga brevicollis	Choanoflagellata	MnSOD	A9VBJ0
Trichoplax adhaerens	Placozoa	CuZnSOD	B3S9R7
Trichoplax adhaerens	Placozoa	MnSOD_1	Q55BJ9
Trichoplax adhaerens	Placozoa	MnSOD_2	B3SFQ5
Trichoplax adhaerens	Placozoa	MnSOD_3	B3SFI3
Bombyx mori	Arthropoda	BmSOD6 (Rsod)	A0A455R4Z2
			**NCBI accession**
Monosiga brevicollis	Choanoflagellata	NOX1(A/B)	XP_001746171.1
Monosiga brevicollis	Choanoflagellata	NOX2	XP_001748123.1
Trichoplax adhaerens	Placozoa	NOX_1(NOX5)	XP_002115436.1
Trichoplax adhaerens	Placozoa	NOX_2	XP_002112911.1

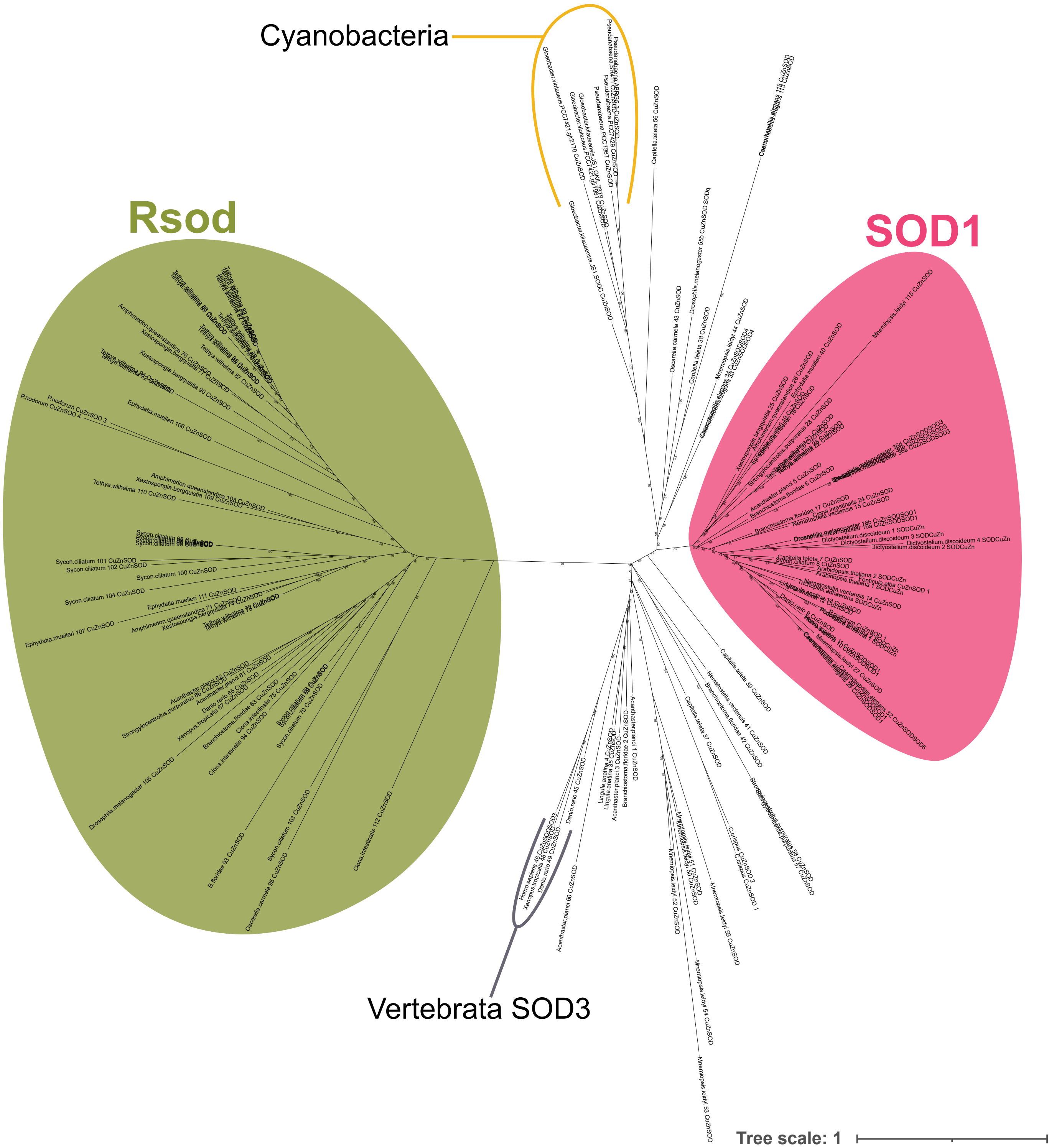

Figure S1. Maximum likelihood phylogenetic tree of CuZnSOD enzyme family. Black numbers on branches indicate bootstrap support. Filled in coloured shapes indicate monophyletic group; SOD1 (pink), and Rsod (green). SOD3 sequences from Vertebrata and CuZnSOD sequences from ancestral cyanobacteria, Pseudanabaena sp. and Gloeobacter violaceus [40] are indicated. Constructed based on edited alignment (Additional file 5), 1000 bb and the WAG+R5 evolutionary model.

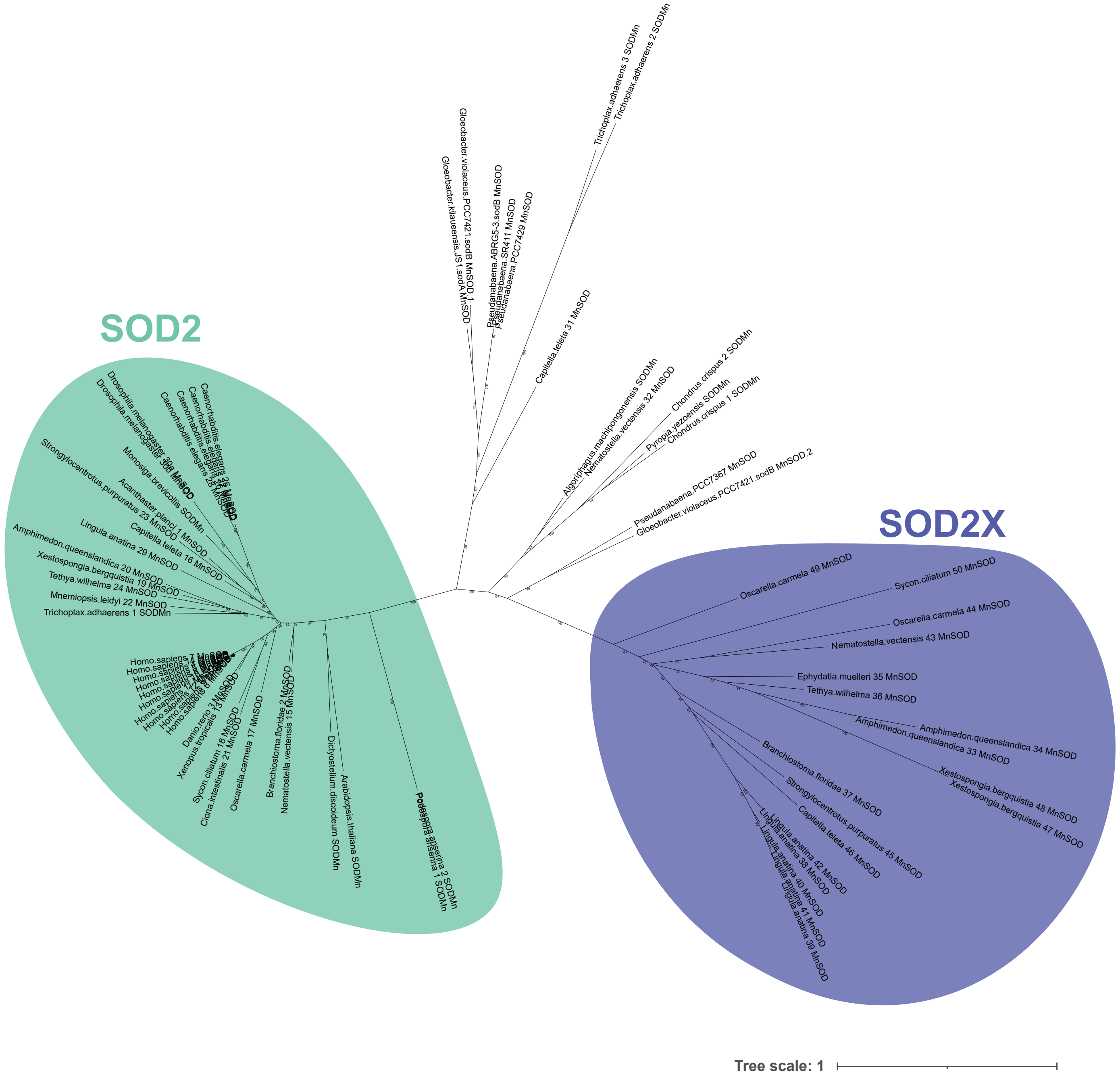

Figure S2. Maximum likelihood phylogenetic tree of MnSOD enzyme family. Black numbers on branches indicate bootstrap support. Filled in coloured shapes indicate monophyletic group; SOD2 (turquoise), and SOD2X (purple). Constructed based on edited alignment (Additional file 5), 1000 bb and the WAG+I+G4 evolutionary model.
