# Supplementary material for: Distribution and diversity of ROS-generating enzymes across the animal kingdom, with a focus on sponges (Porifera)
Source: BMC Biol. 2022 Sep 30;20:212. doi: 10.1186/s12915-022-01414-z (PMC9524095; doi:10.1186/s12915-022-01414-z)
Supplement: Supplementary file 5 — Additional file 5. All alignments and original protein sequences used to generate phylogenetic trees. [file 12915_2022_1414_MOESM5_ESM.docx]

**Protein sequences used to generate alignments and phylogenetic trees**

**CuZnSOD family**

>Acanthaster.planci_61_CuZn gbr.190.14.t1

MLAEVVAQDRTYIATFSMAGASGNVTFAQGPGANTTIYLAMSLPAGTYNWTVHERPNLYDQRPPCNEIFTGGVLASGGDLTAAHGQLTGGTNVMAEFGNDQLRLMGTNSLSGRSLVLEDTSSGAVICANILDFDTDVVTAVAKFMSPFAGTVTFRQRQDDPSALASILVDLYDVLGPLEAVSYRWGIHSVEEYNKNMDLEQRCSNTMPAIFNPTGADASSCQSDEHSTCPIGGLSGKFGDVTVNPVSSSRTRYYIDTNLSLGQDGNSVLGKNLVFADSSGTFIACSPIQVLPSKTVSAEISVSGVNGSLEFRQRSPYDATHISVNLANLQGLAGGYHVHEQPVKGRINKDDLPASNDNVGGHYNPFGVDAGGSPSPGTGTHDQYEVGDISGKFGRLTSMDAFSDQFVDWNMQLFGKYSVIGRSIVIHKEEANARWVYGNIGYPTEVTTVKGDFSSVVAGEIVFRQDAADPFSDTTIFVEVTHADGSDTTSNHNFHVHDKPIENDYVSATDRCASAAAHFNPFVDVDNGYDQCNFTNPYRCEVGDLSQKLATIDIPSVSSSSNKYFFTDPLLPLTGYYSISGKSVVIHTSERGAPRYACANLYCLPTVSVQTREWVGGPVISGSVALSQASRFDQAKISIELSGLDQRAGGWHIHVLPIDPNNPDGPCSAASVQAHFNPFGVVGSPATGTHDMYEVGDFSGKFNSLAGVGSISDVYMDTNLDLTGPRSAADRSIVIHRDDSEGSRMVCSNVYHSLTNEDFHLVAHATFQGGVVTLKQIQFACGYQGDTSLEVDLTAVEENVEHRLEIPQRICADDGIYNPYEISVAVDQCSISRMRLCAVGDLASKHGTVNGTQRTLFTDINLPLFGLHSVIGRLLKITAAGGDGFCSMITPDVSSGKKVTHVFPSTTTFNAYDFRVTVALAIAEGRQWQVVTSEEPVQSTEFPSCTSLSFWVIGPNADDLVTAFSKAVKEGSLGSYNPTDLCSAGSGGSGHAAPSGLMVLLSTILGILVTALPQREV

>Acanthaster.planci_62_CuZn gbr.190.13.t1

MNQTNLHVASFSMRGVSGKVTFFEAEAASGQAMVMINVALIGLELGNYTWAIHEKPMVYGQEPACSEELVGPVYTHAGDLTGALNATTTVTESGSWNASYSSALVSLTGPQSITGRTLVLQEASSQAAYCAQIANARALVTAVARFDAPFAGTVTLRQESGNPEADTTILVDLYDVTDPIEQMTYAWRVSSNSGYNQDVDLETRCKETSSNTYNPTGVDVITCRPEDPLNCPIGYLFGKFNNVTVTPVPSAVTHYFIDTNLPLSGENSVISKSLVFSDASGDPVACSPILEMKPKSSEAMISVNNVSGMVRFRQSSPYDVTEIKVHLENLRGLAGGFHVHVLPPVPRTYASDQPAGPQNVAGHFNPYGIGAAPPPGTGTNDQYEVGDLSGKFGLLTGYDNFNATFLDWNLPLFGRHSIIGRSLVIHKKEGDRWVYAAIPYPTKKKTVKAVFKAPSVGKIVMSQDINDPYADTSIYVEMAYADGTDTTTGHNWHIHTKAVANDYISKDRRCSSCAGHFNPFDADLGPSYGQCSPAFPQRCELGDLSKKHGQLTITNDMNLGMGKFFFTETSLPLSGVNSVSGRSIVIHASDGGSPRISCADLLEVPPTVTTADLWSDGPVSGHITFQQESIFDPVQIHVDVKGLSNVAGGWHVHVLPIDKDMDSAPCSPVSVLGHFNPFGVVGSPPYGTQDMYEMGDLSGKFGSFYGYVDSADHVYHDTNLALEGPRSIIGRSLVVHKSADGSRWVCATLHRKTHPDDFIVTAKAVVTATDYTGYAILSQTRYHTGALGDTTVEFGVLSNVAEMDTFYWNFRPVEPEDDACTGSSSIFNPHKVSVNRDYGSQCDAKTQLRCAVGDMTGKHGNVSSIVKRTVYTDVNLPLSGQDSVLGLSLHLISTNTGQFACGTLEPDASTGRVEYLSYPRSTSFDPYKFRQALHDYMTGAEMWQIVTTQEPEVNQVTGCATLKFYFIGAGLDDMDREFKQKVQADGLGEYSPTSQCKDISGGGIQTMPAALPMFFVLLANCFILFLLQ

>Acanthaster.planci_1_CuZn gbr.2.213.t1

MNVSVQLTGLDTSSATLHGFHVHQVGDLSNGCASTGGHFNPFDEQHGGPSDTQRHVGDFGNVERDSVGSVNEEFSDEVASLVGADTIIGRAIVLHKDTDDLGRGDYDDSLTTGHAGARLACCIIEQV*

>Acanthaster.planci_5_CuZn gbr.677.1.t1

MWRISSWFILAALLVIGGPTLSRGLPEAPRYSLNLDLAEEERWNAILRHWDVGDYQRKFKEIMQGCTSIVAEDTKGNIWHARNMDYDFTATLRNWTVVVDFQSKGKALFRNDTMTYDSALKHLAYHPETAPNYFIIGGNKATEGAVITRDRLTAADVWRLDLSKGRWFVLETNYDHWEPPPKSDDRRDPGNKAMNAIGQDAIDLDGMLKVLSVRLVCNSGTVYTTLMSAAHPSSYVTFIRHFGTVADNTSPQDWSVSIELSNTEPTEYERTVLQCNAAVQPGQVMAIMKDNQPISWNGDLLPNPDPLGPGSSRFVLPNIPQPTPGRVISLLTIDPVCRQDRGNYSCTVFAPRASSGYDVVGSASKEISSVVDFDSQGMCNPVGPISVLIGDEVTLICTARRDGGPTWTLDSGTIVDSPVMHDTMRDIYSRKLSLTITEDHHGAIYHCKANLNGCSTDSTDARTCTVGPITVLSEHQVVVSYAPNPVAVGEMAVFLCTVMPNATDLTVRVSWHTTPSLESNRAYFSYSGPSRMRLETVREEDQGMTVYCTVHLKGGQQIRGQIQLVISPDVPGQNTVVYPTAETTQEAGTGDTPVTSSRLPPDDPFLLFGSYPATVAIISGGSILVALLLALIVFCCCRKCKRGGAGKPPAASKQPLRISPSTFGGPTPGHQVPGSGTDGMYMYATIFDPDEKWGATPGSTPMKESVDARGYQQPMSGASTPPVYSHVGSPNFVMETNIDTPSSRKNVEISVPDGMVGNALYQSSGEYVNPETVNQQQINREMVTIRAEKNLPVVSPVRSVPPGSSTGTGGYLAIGEGTSSRSAPSGVNLLLKEVIYLLQFHHVRALFGGPVKVTGDVTGLAPGKHGFHVHEFGDYTGGCTSTGGHFNPFKKNHGAPTDEERHIGDLGNVEAGSDGTVSVNITDKMISLIGPNSIIGRAVVVHADVDDLGKGGHELSLTTGNAGGRLSCGVIGISK

>Acanthaster.planci_60_CuZn gbr.75.132.t1

MEPKDLENKSKGPASTFNVSKSVTVHVISTETGKALNKSPASDVVSEEHPKKDCSSFSDFIESKLAKSGDPKNQATEDGKDITSTGGEVTAEKKLVSQALVQKIDREESRDVEHPTNGLEELSNSKMRNTHGIKLQFEGKNEVALTAKEINYDSLVTHSNDTNDDIEEEQDLDKVVTQERVMLSKQLQGLARSTNDFENHEQDALKALHRDGLKDFDIEHNDFETVHTLTRLTPISKLAETKQMLTKNEALEKAGKERNTMEDVGAAKTECVEQGMGAEKPSQSTEQTILQDDSIENRDRELGTTDKLPQDIRDDETGVKSVVGKRYVKQVNCVEKTSKSPEEVNSEANVPTDGQDRKVETIVREMAKDMTNTETNDNIASATEVGRQGSKPNVKQVDKGEEEKDVPVSSSTDPGHRSADSSLKVGGHTEKITNEGNESSNKIRVQAHSQARADISLEEANIDADKGVNDHQIVFQLMQFLKQMNKELKLGAGSEGVSSKARYDEGPEQMHPNTMGGHPHIKTQGRTDQEANDDDSDDKAYQGKIKDGQEEKEQEIKGAEEIQSNMVHQKADEIDKAPNISNYVKVNEATDKKVGTKTTGMEEGLKYDIVEEASENIQDTNGNAPNAIKQAVPACITPGGSEHPQDVCNKSSGDTNTTEANTIASDTAANKQDGTDSLGMSQDGAQEKSKLKKVNHVKVPVTKAKLQIGLDEKEKTKMTNSEHKFGGVNKDSPDSATTNVDSTLNEVGHQDEVKSIPGDNTGAAETDAGKVGAKPDAGWKIISSDDNGMSNDRSRHEMSGWKLDDTEGGTKDSVTEAVLQNGDMLSKENQEHNTAITAAKPDGVGAAEGKAGDIKCRDGPLEAAGRASGDGTSLKKEVMNEKEGAVTSHQGLAYNNKDSAEQIQGISQVKEKEDGALNKEGAVTDDNHASAAYEFARCDLQANFPYSDSAVKNHVVGLLVFKQKKSGGPMEIKASLYGLDRDGLVDHTVDVHEFGDLSNGCKSTGTRYSGATYSLSGVMGSALRDETSEVRAAWSTTSEGLVGEHSILGRAVVVHAVPHDSGEREESPKLACCNVARSSEVTPWI

>Acanthaster.planci_3_CuZn gbr.9.16.t1

MQSQIYILRRDVDQLLAELHRTRDPEPVNPEGDGNVYAHCRMVPNGIYDPDTYEPIVGDINMRQRAVGGQMDVKVFLYGFMTPKSVHGIHVHTYGDLGNGCGSAGGHYNPGGNNHSAPYDKDRHMGDWGNIQVDDFGMVEHTFSDKVATLVGPNSILGRTIVIHIGEDDLGKGGTADSKTTGNAGGRLGCCVIGHSDGSAWANF*

>Amphimedon.queenslandica_108_CuZn Aqu2.1.40651_001 Aqu3.1.40651_001

MKAALLLLVSFAALLHLVAGWGSQRSKMVYYVKPGEETRFWRAPGQHVPPQTGRAILVAAYFDMNGIKGSVAFYQEKEHEPVTITLSLSGLSQFGGNERWGWHVHEYPINWAHLERNPCNTEHIGGHYDPENRASDPNYAQLCMNNASLCEVGDLGGRHGHLRGNQSVYTFTDNTLNLYGPYSIVGRSLIIHRTNGFRYACTNIEYNGISTIHTYRAAFPCNVKEQSPVFQGEVIMRRSALRHGVTLEANLYRVDGGSINTTHNWYLKSDGNFNVDCSVCKNDNADLFVFGNRTHGSSTNPFAGCGAATRAQHLKCPYGDLTRKCGPLSYTNGDQIQAFCTDTQLGIYPYESVNVNTILVIEDEDGNNLACAKFEPVVPVAANIDIRNRNVYVAGRLFQYDPHDPTYVQFYNTRLGTASHFQVRAHPVPPDGSCTGLGPVYEPRFRFTNLPSSVLINQTGDRDFLGELQNKIIVPNQSIRNSYYQRTSYLPLFGDYSVIGRSFVVLDNNEFVIGCGNITDADPNRDENLRRSLEFYHKPRPWYE*

>Amphimedon.queenslandica_71_CuZn Aqu2.1.41326_001 Aqu3.1.41326_001

MQVHAPITLTVLIWLLLIECCSANLTAIATVSQRGVTGTIIFTQNTPTSETNIRLTLTGLPPDELYQWHVHQYPFASALPSPCSPPNTGGHFDPLGAFNQANYSTNCNKNNPAACEIGDLSGKFGLLNVSDLPFSEGDDTISLYGRHSIIGRSIVFHFSNGTRFVCANILINSSQDVISRAYSPFRRSSIVGNIYFTQYSFNLTTVFVDLLSPLATQDHNWHVHEDPVTGGDNACSSTAGHYNPRNVNVTNNDYLNNCNESNPFECEVGDLSNKGGTISFSSTSNQGRLFYSDIDLPLLIDSEGHYIENRSIVIHQENRGAPRVACGNLTALKPREAVSVFNGEEGVYGSIKFKQDSPFDPTTVLVNITGLNEMAGGYHVHETPVGEGVTDRSRCGPSYTGGHWNPRNAPYPLPPGLTSDQYEVGDLSGKFELLDGLNESAAEYSDPNIPLFGVDSIIGRSVVIHLAEPGAPRWVCANINYARPTVQVSAAFSFSNNQSFEVVFVQPDDDPYSDTTIFIRNNSFIEPPSPSPSPSPSVTSPTSLSSLPQTSLISSLEFAMSSSLSPSPSPSLLLGSGDGDMMLRKKRFSDSDIMNELLEADDVYMYEENDEMNDQFVNEKEAEHPIMVAKRQIMDLSPISWSVSNLPAQQGGGVACDLFMPILSNSNDTLCMNGVQLGCSGGDLTAKHGPLTVNSNGMIRTVYTDPYLPLSGDNSVLGKGLLVQSDGEGQLAAIQPSDMPALTNATSCIVSTSSTSSISSTTSTAAITLTNTISTSQVTTTSTLSTTSTSAGPSISVVTETPSGVLPFSNTILIIICAGGGGVLLLLLILFLALCIICCKCRRSNRYDKYMKDHIRSAGNIELGKYKRTNPISGVSCYRVLYDYDPQQDGDLELKEGEMVTLLESPLDGDWWCGKLENEKQGWFPKSYVEYVDIVGENKKRQEDSFAVAAATIRVASMAFTSPNTPSTNSKLHKDLKEEESTFIIPNLHTGTPQASPQIARAHTPIKRAPSEATPSPPPSYESPIPPEDIYIAKFDYSPQSDSELKMAQGDRVIIIERADGGWWHGVIGEEHGWFPETFVEPEESSPPMYDEDKKEETKKEEEEEPFRPRGMTEFHKETSDEVEASEYKAVYTYTSDTDGDLKFQEGDTILVYWANPNGWWFGSVDGRQGYFPGSYVEAVMEHTASPNLSGEGLGVIKEESPPSEAGGTSFSDEISNAFKKRKEAAETGGERAAISPVPSSSGSLSDEIANAFKRREERKPGATDSQKSSPALARGGTSGSLEDQLAAAIKKRSTSPPQEPPVVSSGGATGGGGSLEDQLAAALKKRSSSPPAHAIKEKEQVDEKEKEEEKREASGSLGNIQTEIMSMLTSFGGSSESSTVGTDKEKLIKDEEPQATDKKPVKRRQAPPPPKSKSVTSKSPTPASPEPPLQTTPTPTPSNETPKSPDDTQATAIPPVVKATDQTTPTGRRKPPVVPLVVDKPVEATPTNISKTPPIEQKVKTEEPKKQSRTLPPKVVKQLTASPGTKARRAPPPPPSSLATHTKTASVSSDDDKSGQPQNKNKPIALEAAKPTSTSSDSASEKILSSETQEPKAIVQKGSGWKPQTRKGKKQAPRRNPPLPPPTATPTTVAASTQSKNKPKVQIVRRPTARNIPPPPNIPLPAPPKKSPSNIPPPPSTSLPPPPSSPLPPPPTSSLSPPPTSFLSPPPSSPLPPPPSSPLPPPPKQKSSDSDNSERKKSVTRPSRPPLAKQKSTDNSGDPPSVPSHDSDYEKPISTPSKKASADYEEPLPKPLHGYEKIDPSKLVERGSYDKPVPPKETKPAGNEYEEPLPKPRVRTSATKPPQIANQYEEVKSPTKDDDGNNDYEPVRPPTVAELAVTTNPAPPVASRDSNLKPSEPSNPLTGPSPKPKVKPKPAKLLKPKPPVAKKPASLSPSVNKKEENESAEGTAPKPKPRPPKPSPPPRVSSLPSTPQGSPSPTAKRKHSPVPSPLATRSFPVPPKSPSSPLPPTQPSTTPSSAPPTKAERKSSLPPPRPKPPPIRKLSAASAEQTDAGNNKVTSPLSNGPIISNGVKPDKLDQPVPKARRTSGEGSTKASIPARPPPPRSIKKEKKDDDDGGQAQKYYKALKSYTSGGGNGDHLSFSKGDILVVLDDGGAGKEGWVHGMLDDGTTGLFPLSHVTDFQPNQ

>Amphimedon.queenslandica_26_CuZn Aqu2.1.23371_001 Aqu3.1.23371_001

MAVDKAKFETSPPVARAVCILASSDDVKGTIEFIQNEQGITKVTGKVTSLAPGDHGFHIHQFGDYTSGCVSAGSHFNPAGKNHGGPKDGERHAGDLGNITSTGGDTEIELYDDQIPLTGPNSIIGRSVVVHADPDDLGKDGHPDSLTTGHAGARLACGVIGSTKLQ

>Amphimedon.queenslandica_76_CuZn Aqu2.1.41997_001 Aqu3.1.41997_001

MKTKVMLAFSLLLLLVAKGTEGQTYGRATFNIRGITGYITFMPQPNNRMMIMSNISGTNANHHWHVHLLPVDGSLPPAMRCSNDLIGGHFDPLGAVVSLGSNYSMYCNPNNQSACEVGDLSGKVGPLTSGVRNYNETTGELVLFGVNGIIGRSIKIHGTEDVCATIVSSTELNGSPTITMLKAAFIYPVGGVVYMRQVMGESAVIFGKLYWVNNSSSTMNHPWSIRMNKPSQYYREGNCNNLGSVVSDLGSRHRTISISNAPQPYYAWSFFFQDPTLNLTDPANGGSLVINFNSSNNVLACAPLVQVETLSVAAINRFTASQSSRFASTLVTTGYSPSGLRVFSSAIAPNSLCKNEFSAGRPRIYNPHSAPPLDGSEGTPDRYPVGNFSIKYRRFSPSGSRVTELPIHGHETISGHSLGSMRMGSSGMTHSCSSLWPQYPSGAEVRMAKATFNSTVTGAIYFIQRRFGDGVLGMTHIMVDIWYTNPSTAQTSGHKWHIHERPPQEGVRCVDAIGGHFNPFNVDLNNNYSTECTPFAPLRCESGDLSGKHPKLSISAASPTRPTYTYVDNNLHLWGPANYSIIGRSIGLHSTAGPLFDCGPITEIVFSSRNRTIMFSYDANFDRTTFATQLTRNARTASPSDVLILDQEMTTDSSGNTGCVSATVRTVGGSTEDADDMLNSLEIPDSAGCTSSPPSSSALMLPSLFLILFSVLLVIAGAI*

>Branchiostoma.floridae_42_CuZn XP_002605534.1

HGFHIHADGDLSMGCDTAGPIYDPFNKSHGGLNTTERKVGDLGNLDCDELGRVMMLLEVEDASLLGPYSILGRSMVIHANEDDFGHGGHEMSPVDGNSGVRLACCVIGRTSDAELRWAAVTER

>B.floridae_93_CuZn XP_002603657.1

MLSPTTRKRKLLDSLGSVTADPGLSHGEKVRRLADKLAGASGTDLYGPDKEVEDLLKQAPPRVMVKRGDSRIHESNSCLSNRTNYPELTRQIEQDVQISRIMNRLDIVDGTSTHDVHHGVPKGFNQDQRKEQGNYATRCKPGNPLRCELGDLSGKQGKYAVGGGQRVYTDVDIPLYGELTVVGRSIVIHEANGGGGRLGCGTIRPMTQTATIAFPPIDNFDSTDFRTTVANLLEIAVWKVAIFYVPKTNTIRECQEVTFYVLGNIERSKVEALVSGSQASELGKYAPGDSCKSYAFLKLHCLDCMEYLQNGVTTGCGEC

>Branchiostoma.floridae_63_CuZn XP_002603653.1

CAEIYHLEAVSVSARFDMSGVKGTMVFSQNSPYEYTTVTININNIGANAINYHVHEYPIPAKAADESMCSGTYTAGHLNPFGKVPSSDSYPDPAAGTGDQYELGDLSGKFGNFNGMDTVSTVNTDWYLPLYGRYSIVGRSVIIHDTTGSRWVCANIGYPGEVITAMAVFKTPIAGHMVLRQVKDKPYLDTSVFLTVSYIDGTAGTSDHSWHVHEDSLGLDYAAVENRCSSTAGHFNPYAVDLSGNYASQCNGNNPLRCELGDLSGKHGKVDVGSEDSISRNFYTDVELPLSGPEAVIGRSMVIHQANSGGGRLACANIFQLPAVEVSTQTWHAQGGVSISGTFSVTQASPFDSTTTVIDVTGLSNNADTYHVHKLPVPFGEDNPCSAASVAGHYNPFNVVIANSPASGSGTSDQYEIGDLSGKYGSMQGQTGFSETNEDGNLPLFGTYSSIGRSIVIHDNSGGRWHCANLTRVLPVDASTVSAMAEFEA

>Branchiostoma.floridae_2_CuZn XP_002599809.1

YGVTVHELGDQSKGCTSTRDHFNPFNLGHGGPKDEDRHLGDWGNVEADSTGEVNAIIKDEVASLVGPYSIIGRTIVIHANEDDLGRGETAESKKTGNVGKRLACCVLG

>Branchiostoma.floridae_6_CuZn XP_002594377.1

QSPGGPVRVTGEVQGLTEGPHGFHVHEFGDYTNGSCTSMGAHYNPIGTNHGGPNDAVRHVGDLGNIVANVAGVAQVDITDNQLSLYGADSIIGRGVVVHADEDDLGKGGHELSDTTGNSGGRLACGIIGITK

>Branchiostoma.floridae_17_CuZn XP_002590336.1

MSLKAVCVLVGETVKGTVTFTQASSDSPVEVTGTISNLTPPGKHGFHIHEFGDTTNGCTSAGSHFNPAKKNHGGPQDAERHVGDLGNVEVGDDGVATINITDSQLQLTGPNSIVGRAVVVHAGEDDLGKGGFEDSLTTGHAGGRLACGVIGITKQA

>Caenorhabditis.elegans_29_CuZnSOD C15F1.7a

MFMNLLTQVSNAIFPQVEAAQKMSNRAVAVLRGETVTGTIWITQKSENDQAVIEGEIKGLTPGLHGFHVHQYGDSTNGCISAGPHFNPFGKTHGGPKSEIRHVGDLGNVEAGADGVAKIKLTDTLVTLYGPNTVVGRSMVVHAGQDDLGEGVGDKAEESKKTGNAGARAACGVIALAAPQ

>Caenorhabditis.elegans_30_CuZnSOD C15F1.7b.1

MSNRAVAVLRGETVTGTIWITQKSENDQAVIEGEIKGLTPGLHGFHVHQYGDSTNGCISAGPHFNPFGKTHGGPKSEIRHVGDLGNVEAGADGVAKIKLTDTLVTLYGPNTVVGRSMVVHAGQDDLGEGVGDKAEESKKTGNAGARAACGVIALAAPQ

>Caenorhabditis.elegans_31_CuZnSOD C15F1.7b.2

MSNRAVAVLRGETVTGTIWITQKSENDQAVIEGEIKGLTPGLHGFHVHQYGDSTNGCISAGPHFNPFGKTHGGPKSEIRHVGDLGNVEAGADGVAKIKLTDTLVTLYGPNTVVGRSMVVHAGQDDLGEGVGDKAEESKKTGNAGARAACGVIALAAPQ

>Caenorhabditis.elegans_33_CuZnSODSOD4_F55H2.1a

MKTRVVLILALSVCIEAASEVIRARAYIFKAEAGKIPTELIGTIDFDQSGSFLKLNGSVSGLAAGKHGFHIHEKGDTGNGCLSAGGHYNPHKLSHGAPDDSNRHIGDLGNIESPASGDTLISVSDSLASLSGQYSIIGRSVVIHEKTDDLGRGTSDQSKTTGNAGSRLACGTIGTV

>Caenorhabditis.elegans_34_CuZnSODSOD4_F55H2.1b

MKTRVVLILALSVCIEAASEVIRARAYIFKAEAGKIPTELIGTIDFDQSGSFLKLNGSVSGLAAGKHGFHIHEKGDTGNGCLSAGGHYNPHKLSHGAPDDSNRHIGDLGNIESPASGDTLISVSDSLASLSGQYSIIGRSVVIHEKTDDLGRGTSDQSKTTGNAGSRLACGTIGIVEERILETTTASLPPVTQSQPIGSSSYYYSTFYLPIILYFLLSRIL

>Caenorhabditis.elegans_32_CuZnSODSOD5_ZK430.3

MDILSDIANAVLPQDVVSKVESKRAVAVLRGTAVFGTVWLTQKAEGEETEFEGEIKGLSPGLHGFHIHQYGDSTDGCTSAGPHFNPCKMNHGGRDSVVRHVGDLGNVEAGADGVAKIKFSDKVVSLFGANTVIGRSMVVHVDRDDLGQGIDDKAEESLKTGNAGARAACGVIALAAPA

>Capitella.teleta_7_CuZn CapteP181944

MVLKAICILKAYGPQEPGSTPVEGTINFTQEGDGPVTLEGQIAGLAPGKHGFHVHEFGDNTNGCVSAGSHFNPFGKTHGGPDSEVRHVGDLGNAVAGDDGIAKINITDDQVTLTGPHSVIGRTMVVHADPDDLGLGGHELSPTTGNAGGRLACGVIGITK

>Capitella.teleta_39_CuZn CapteP100118

MTYVQCVVTVNAPLTFLIPEAAGEKITAECVIEANSGTGPIQGTIRFEQDITGGATSITGSVSGFTTGDGSKHGFHVHAVGNLGNRCSDATGHYNPFDKNHGAPDASERHVGDLGNIVENANGVADISMDDSLVSLVGEYTVIGRSIVVHAGEDDLGLGGDSGSLTTGNAGARLGCCIIQEVGGQILHQGNRK

>Capitella.teleta_37_CuZn CapteP148692

MFYRCIILALVLQNSLCRSSVDRVDTFEDEIRSARVAVQDKESYIYAQCQVAVNSAIDTHPNHVIGTIDLRQTVGRAVSEIRLNLTGFAADDGEMYHGFHVHELGDLSNGCDSTGSHYNPLDVDHGAPHDSAFNRHIGDLGNIEEDLGGNCIRTITDTLVTLQGRFSVIGRALVIHETYDDLGRSGVDDSLTTGNAGARLSCCVIGLTDDQHWD

>Capitella.teleta_38_CuZn CapteP72786

HGIAIHQYGDLSEGCTNLGDHFNPLNVNHGGRLQSPFQRHVGDLGNIHVNLDGSASSELKDYLIQLQGRYTVVGRSVAIHELTDDLGKGGDSDSSTNGHSGSAIAC

>Capitella.teleta_56_CuZn CapteP51623

EVVMFQVSPAGNGPTVGKVVIHYADDDDVAQGVRFQPALHDLPPGAHGFHVHENPSCEPGEKDGQKVAALAAGSHYDPEKTGHHEGPKGHGHLGDLPALTVDDYGNAVTPVEAPRLSLPELKGHSLVIHAGSDNYSDHPPLGGGGARIACGIIQ

>Ciona.intestinalis_75_CuZn ENSCINT00000029133

MLRLCVIIAAVVHLGHSLSYRATINMAGIKGYVDFTPDTSDMNVTVKLTGTGVSNYTTFMIKSIPTMFGQVKSTQACDNLGNTITVLTGATNDATNTTVGLSEEAIFHRSVLITGNSNDPKLCAVILPINENLVTYRAVFMGPITGEVWVRKRQSLNGIFVYSNLAYATNPTNPTATTNLKMVSYASGSIGQTFSTRCNTLASPTDIMVAIPVGNSAAAAGRMSATFVQSISDVKTFWGLTNSSSPTIIACTHINLQNPRSAVTRFAWSNLRGTVTFTQDSPFDVTMTSVDLLGVQFLNATYHIHKFPVPQRLGASENKCSFNNVVGHYKPFGASSVGPANAAGTHDQYEVGDLSGKYGKLTSATFAMNYTDWNLPLFGVHSILGRSVVVHKSSSERYACANIVYNGAVVTAVATFKYPAYGYMYFQQAANDVNAPTVIFGALANTDHGVSVWHNFHVHEF

>Ciona.intestinalis_94_CuZn ENSCINT00000004617

MIQPNEFSATSVTANLAGLANTAGGYHIHILPLEAGSSGVGACSTSVISGHYSPFDLPAIIPNATVGTTDQYEVGDLSNKYGLLTSQASLSLPVTLDDNLPLSTDVSVAGRSVLIHKASGTPWKCNSLLEQPYDNTGHVIVAEVTFADAFTGYIRLRQVIYDDGTSTPTTIEVSVANNNGSLTNNHNWHVHIDPITTETSCKIAKGHYNPHGAPVSSANYAGAATCNRMQPLACELGDLSNKHAKYNIGGGRKLFVDVDLPLNTGNSVLGRSIVFHAAEAGSARIGCSNIQPINNGSNSMTIAFPSSVTYDYYRFTKAIADVFAISRHRVVVVRNTAVPVSGVGCSKVMVYIAGTLPSSPTLADVVAGLNVNLAPYAPTVACKSEIGLQVVGLSDLSGASINAVQLSFVLFVALTSCFMQFALF

>Ciona.intestinalis_24_CuZn ENSCINT00000035102

MVLEAVCVMKGSESVSGTIKFSQVGDGEPCKISGSLTGLAAGKHGFHIHEFGDHTNGCTSTGGHFNPQKCDHGAPEAEVRHFGDLGNVTADSSGVAEVNISDKYVTLTGINSVIGRAVVVHADVDDLGLTSHPQSKTTGNAGGRLACGVIGITNHR

>Ciona.intestinalis_112_CuZn ENSCINT00000010939

MKLFFLFTLFVASVAGSKYAHCMFKQQEPVSGHICFTEKHGGQVEAHVQVSGLDRCENYRVHVHEHAPASDGSCTTVGGHYNWADPQNIDEGDMLTLKSNTESVLHKHIHPLNSALSLNGERSIIGRSVAIHVGGHKVCCPIVACSKRTYRHMTRNLESYVHPPVCPMSPIRHSKRIPLVNLDVIGIRPEPVVPDGVWIEIQPCAAGNEAEHNAYFEEERINATNLALAERLIKFQRITQKRVVRAANLQRRNIDQAQSEKMEEEREVLHRSASAAERSTPKKNRCSYNVNPITIDGSGKITSSAQLSASNTKVGAELDLKSDETSNQRRIARQKLAEKLIVPNTRGSKELQDRETPLPGGVWGVGASRDKSSTRIHDSHVSISEQNTDIPNGNSALNETVQTIPAVLAPHDVALASLQESITDPTRKPVRTDLLQTLLSTNCTIDETNQPLDREQEQIENLPGHASSHTPHTVHFKLNKEQWKSENITPVEEVTTGVVVTRATRSTGDFPHPTEYPVPRRELASIENKGVVEINAKKASERARWMSRRVFADAERERAREMLRMKEHKQRVNILKSEKERQRKELEEKSRHQVEAVAVVTEQLKRDEIKKESKMRKRKEKEEKKQQKQQRFSAAIRNQLKERVEQNKIELPPLCMCGLTFWDTNPNTCANNCMFYKNDGAYAKALQAVLEAVERNIGPFHKDALQRCAVSLPLVTDDFEDAPPLESARTTVSIRSQLSNAFTFEKLMKLFTICLVLLLVGKLCEGKDDVHRYQVTLDKAACPDGDCYKMVQEKVKELEIENDVKIDVQEYEKFQHQDL

>Danio.rerio_45_CuZn XP_001332758.1

MIRFNILPLLAFLLSCHVLFCGSGSGLAYASNSDSLQAVCRMQPNTRLEPGMPRVYGHILFRQSGPKEKLSVTFRLYGLPADSQQPRAMHIHEYGDLSRGCDSTGGHYNPLNVNHPQHPGDFGNFVPVNKKIRQSLESPATLFGKLSIVGRSVVIHEGKDDLGRGGNVGSLLNGNAGGRLACCVIGLRNPQN

>Danio.rerio_49_CuZn NP_001092706.1

MKKLISVILLALHIQRGDFSDNSDFHLIEAPKLEVFEFNNTIYATCEVSPIPNLPAGQPKIFGQVLFRQVFPNGTAEVKINLRGFPETDNQVRAIHIHQYGDLSQGCVTAGPHYNPQDVPHPNHPGDMGNFMPKQGLIRRFLKLPEVKLFGGQSVLGRAVVVHEKEDDLGMGADEESKRSGNAGRRIAGCVIGITKPHLWQKTEHVEEEESKR

>Danio.rerio_9_CuZn NP_571369.1

MVNKAVCVLKGTGEVTGTVYFNQEGEKKPVKVTGEITGLTPGKHGFHVHAFGDNTNGCISAGPHFNPHDKTHGGPTDSVRHVGDLGNVTADASGVAKIEIEDAMLTLSGQHSIIGRTMVIHEKEDDLGKGGNEESLKTGNAGGRLACGVIGITQ

>Danio.rerio_65_CuZn XP_001343650.5

MHTQTTTANMLLQGALTLFILWGSASCVRYQADFNMMGITGWIRFDSADQRSTVNLTGTSRCQSFNISLTTFPVMYGHFASPCQKSHIGDSVFSFFVEQPQATVNVSTLFEQHLSLDALSVLVDTCNGTRVCAALTPDSQVRTLQARFFSPVSGNVYIRQLTGEAGARVLSNLRNVDQTSTLTNVTIFVSQSVMSCTTLISSLDPKSLTKLGVLNLGSSLEAVKSRLEISTFNPNRFAVLSLTSGYICAEIRSISEKTVNAVLNMQGIKGYFSFQQKSPFDLTTITVNLTNLNRRVGPYHVHQFPLPQMRSPSDSSCSNNNAGGHWNPFNVNVQAPAYPPPKGSTHDRFEVGDLSSRHGSLENTSNFQATLIDWNLPLFGWNSIVGRSVVIHMPNGTRFACSSIDYPGEVTVAKAVFRGPVVGTVLLSQLTGDPYSDVAIFADLSYGQLSAQSTVNHSWHIHNYPISTETDSDVGCCLSTGGHWNPYNINTTGSAYTVNCSPGNPFACEVGDISGKHKTLDLQSDMGSVATKNFFTDTTSWVLGMIGRSLVIHGSDRTATRIACANLTLYRFPSALSKSWFGLESSGGQIRFSQVSPQGPTILDISFTGLNSKAGGYHIHMLPIKSTQDPCSDSNIMGHFNPFSVNATLSPAPGNGTVDQYEIGDISGKFGDLTGQNNFQNQYRDGNMPLSGPNSIIGRSLVIHYSNTSRMRCADISAEASKDGNWVTAEATFSNAVTGTVMMSQQTFPDGSYGDVQIEVDLQSSNFSWASWYIADKPMYPDGSCPEDDEIFNPFNMTNMNNCSQTRALACMVGDLTGRYGPIRLSKRQLFTDSLVQLTGDFTVIQRALVLRVNGTTAVCADIYPESPSALQIFPKIDSFNRYDFRKKVAEVLNIPISRISILPGSPSSLSNGTCQQVNFVVSGEVSPEKLNSVKNSDKMGSFRETKQCSRTGNAGLILVPCWMSVFTLTAAVCLLRL

>Drosophila.melanogaster_36a_CuZnSOD FBgn0033631

MMQYLVVSLALCATICSAAQTRNMPIQAIAYLIGPVQSDNTQVKGNVTFTQNDCGQNVHVRVQLEGLKEGKHGFHIHEKGDLTNGCISMGAHYNPDKVDHGGPDHEVRHVGDLGNLEANSTGIIDVTYTDQVITLTGKLGIIGRGVVVHELEDDLGLGNHTDSKKTGNAGGRIACGVIGINGPSVPAPAPPTPRHQPLYYVPHGEPQDHVVYPLQHYPLPYPHPYPYPHPYPYPYPLSHQLYL

>Drosophila.melanogaster_36b_CuZnSOD FBgn0033631

MMQYLVVSLALCATICSAAQTRNMPIQAIAYLIGPVQSDNTQVKGNVTFTQNDCGQNVHVRVQLEGLKEGKHGFHIHEKGDLTNGCISMGAHYNPDKVDHGGPDHEVRHVGDLGNLEANSTGIIDVTYTDQVITLTGKLGIIGRGVVVHELEDDLGLGNHTDSKKTGNAGGRIACGVIGIK

>Drosophila.melanogaster_36c_CuZnSOD FBgn0033631

MMQYLVVSLALCATICSAAQTRNMPIQAIAYLIGPVQSDNTQVKGNVTFTQNDCGQNVHVRVQLEGLKEGKHGFHIHEKGDLTNGCISMGAHYNPDKVDHGGPDHEVRHVGDLGNLEANSTGIIDVTYTDQVITLTGKLGIIGRGVVVHELEDDLGLGNHTDSKKTGNAGGRIACGVIGIK

>Drosophila.melanogaster_36d_CuZnSOD FBgn0033631

MMQYLVVSLALCATICSAAQTRNMPIQAIAYLIGPVQSDNTQVKGNVTFTQNDCGQNVHVRVQLEGLKEGKHGFHIHEKGDLTNGCISMGAHYNPDKVDHGGPDHEVRHVGDLGNLEANSTGIIDVTYTDQVITLTGKLGIIGRGVVVHELEDDLGLGNHTDSKKTGNAGGRIACGVIGINSDVDEWPCRDGGAGALRYSFSILTVIVALIMARSLD

>Drosophila.melanogaster_36e_CuZnSOD FBgn0033631

MMQYLVVSLALCATICSAAQTRNMPIQAIAYLIGPVQSDNTQVKGNVTFTQNDCGQNVHVRVQLEGLKEGKHGFHIHEKGDLTNGCISMGAHYNPDKVDHGGPDHEVRHVGDLGNLEANSTGIIDVTYTDQVITLTGKLGIIGRGVVVHELEDDLGLGNHTDSKKTGNAGGRIACGVIGIK

>Drosophila.melanogaster_16a_CuZnSOD FBgn0003462

MVVKAVCVINGDAKGTVFFEQEVRIQNHLNFSARQNSSGTPVKVSGEVCGLAKGLHGFHVHEFGDNTNGCMSSGPHFNPYGKEHGAPVDENRHLGDLGNIEATGDCPTKVNITDSKITLFGADSIIGRTVVVHADADDLGQGGHELSKSTGNAGARIGCGVIGIAKV

>Drosophila.melanogaster_16b_CuZnSOD FBgn0003462

MVVKAVCVINGDAKGTVFFEQESSGTPVKVSGEVCGLAKGLHGFHVHEFGDNTNGCMSSGPHFNPYGKEHGAPVDENRHLGDLGNIEATGDCPTKVNITDSKITLFGADSIIGRTVVVHADADDLGQGGHELSKSTGNAGARIGCGVIGIAKV

>Drosophila.melanogaster_105_CuZn FBgn0051028

MAPGGRQLLWMASICWIASLMSGADAQHLIAYISQRGLHGEITFRQMNATTVEIKANLEATLQYPDQVWSWGVRRFPVDYSNIDPDERCELSRLGSQVLSFDEDLEYLVLPGNETSSWERNMQLIGDRGIWGKSLVLTEVNANARICATITTIQSSVEHMAEARFNTPVAGSVHFRWLAPAEGAVGDTLIYSDLYHIRAQPAALEEDRSQAGPFTQHHWKIYVTDIFKHDHHRTEDNCNFLQQVFDPQGSGAGKGIGDLDARLGRIGVAKNALRSPQRSVFRDAQLALLPSDLTIPHRTLYLVLFDNQHPDSYLACTKIRHVQPLTHKTFINSGGVKGEVTFMQRSKFDPTFLNFTLGAPLSQHVSRKFAEDVAAFRIHSLPPVPSRMGHEDYCLTTGDMHNPREINENIPPPGYGTQEQYPVGDLSGKLQGRNKGYWHQYVLPGTSSELNGLYWDVFLPLQGRYSIAQRSLVIYTFNRSNVTNITKMIWGCSSLSQYQRNGIYQQNMVTAQVLFRYPVVGRVIFRQTAEQPWQDTTVLFEYLIQADGSTQNTTHDHRWAIHSNAPGKDFYDWQQRCISAGPVYNPFRVDWGNRSVDDFCRPSLASMCRVGAMDARSGTLTIAGGRRVAQKISRKMFVDGNLPLSGRHSILGKSLVIYEDSGPKARGERLACSAVIGHFRRKVVAKEWYANGDSLTVSGRVEITQQSEYDVSNVEVQLKGLQDNTGYYIHRTPVEANLAFPCEASTLYGHWNPFDVSPKSSPPPKRGTTDQYEMGDLSGKFGGLEGVTQFEDAYNDTNLPLFGYNSIIGRSVVIQKKQKNARWACSTLERGYSPSEARELRAIASFHHPTGYAYGYIKMTQLIHNDGSQSETVIEVKLRHPGKNDRNSTRNHNWQIFVNPVGVDAAVKPTITRCVAGGYVWNPYYTQLADPLNLDLYEQECSPDNPLRCYVGDVGARLGTIDLGGERVVLTDSNFPLEAPVGAIGRSIVIFGPDHSHERFACANIEPDHNVIKYINLQKPPRFVVAQFLDELRSVMGIPEWMLDVDARKTKELHGGACIQMIIHFKGPLAHRLELDMSRLIAAGRLDAPSLFIPGYVNQKRKATISYRTCGVRDTNEKRTKNFKGGFYSSSSAPNEPKPLVLAFVVIGLAFRFL

>Drosophila.melanogaster_55a_CuZn FBgn0039386

MLIKLILALVIGYGGIFAPGSAQNLLDRLRPVNMTRFDDGIKVVSGTKVKRYERLTVPLGGPVGIPGAAGLLGPLLPPQPSYLGYTYLVPQWQAGAKLMGDGEGAGVAGMISFVQLPYNSDIRVTINVTGLPPGKHALHIHTFGDLSDGCKSTGGQFPNNFLGNVDTKDDGSISAVFQSIYLQLFGINGIVGRSIVIHSKAIDLNTALNAEVFSSSLQAMPNPLAYQNEENSLGPAIACGVISIMSTAASSSGMATAAPAPPAMENPEI

>Drosophila.melanogaster_55b_CuZn FBgn0039386

MLIKLILALVIGYGGIFAPGSAQNLLDRLRPVNMTRFDDGIKVVSGTKVKRYERLTVPLGGPVGIPGAAGLLGPLLPPQPSYLGYTYQLVPQWQAGAKLMGDGEGAGVAGMISFVQLPYNSDIRVTINVTGLPPGKHALHIHTFGDLSDGCKSTGGQFPNNFLGNVDTKDDGSISAVFQSIYLQLFGINGIVGRSIVIHSKAIDLNTALNAEVFSSSLQAMPNPLAYQNEENSLGPAIACGVISIMSTAASSSGMATAAPAPPAMENPEI

>Ephydatia.muelleri_107_CuZn Em0003g1549a

MGLELDTSYTWRVHQYPSSGQHDPCSYSAAGGCYDPQYVGQQPNYTALCAANQSLCEIGDLTGRHGPLTVTRAVSVIEDSCLNLYGVSNIVGRSLVLNWNETQLACTNIGYPTSTPAQTILYASFRVGFVGAVYFRPFTDEAMTVFADLYIANDLMESEEHSWHTYELATNSSDCTQTTEFHTGSEMIVTCDPNMQEICESGDLVGKGSQLEIYNGTLKTFYMDTTSLSLPFLDIYSINRSIVVVGTNETTNVIACASMRVLSQLHAVAQFDTGGMVGTIQFKQASPFESTILTVHLARPNTIPSGFQIHKLNIADGDCNSAGEPWTPVGMTSNGGKHLNGGYNYNFGNVYGPTSIYQTYSDSDISLYGTDSVIGHSILVYFANSSLMACANIQHMTTTWVSSVHFNTSEIEGSITFVQPTSDLYAATTIIVDINSYPGITPLGWPSSSIVPLPSVLPTLHHDPLTSSISEWTTQLYDFPSQTSMPPPPLVPSTTGMMKKRDAPKQLPKRQTTQTTAKYSYGWRINGISNGSNPGIFSPYGPSRLACSPSNQFMCAVGDLKGKHGILTSNRAVLTDTNLPLAGLHSIIGSLLCIQIESSGPTVCVAISPVSIVAVPNDIIITIPSNSKSTSPMPSSVMITTPLSSSAMITSSKPSNVVPTSSMASEIDYHLELPTTSVAVQLVTATEIPCYRALHNYKPINEDDLQLRQGDIVTLVQATSSGEWWKGTVEDQSGWFPKICVQYFDTKAEERQRQTVGIITPKSEWKSKINHNFRKACDIKVTTTFGQAGPALSSSSCTKLVTTGPDCRPGSPESMSEVIVNMAALGLPHQEVEALQHTAADHEVMSPGVSSDGLLDGVLHGGHYFETNSTQLSLGKHGMLTISSDTEVAWNGAEETRDGTNSQLYGRNRLFDH

>Ephydatia.muelleri_106_CuZn Em0013g891a

MLAIVVGVLFCNVVVAYSQSAAVATFDNANGVVKGTVRFTALADGLQVQVSLSGLSGNLTHPWHVHVNQLNSTYGYPLVCDYVDAHFDPFNANITSTNYTQRCSANQSLCEAGDLAGKFGSIPVNGTYGNGTYSDTFLDTTGTLLVSDCVGRSVVVHNIFTGSPMQHRECANVYAVSPTPTTAASPTPTTAGAHSVVDQIKLVALLVLLVLFIA

>Ephydatia.muelleri_111_CuZn Em0022g124a

MCALGDLSGRYGYLNSSSSTFSASDADLSLYGAYTIFGRSVVLNWNSTLFACANIGYPAGIVLYAPFRAGFAGNVYFLSNYGNSTAVFADLNAVNGLVNSSGHNWHVHIQPALSNNCAAAGDHYNPLGVQTGGNYSLSCGPKAPELCEVGDLSGKGGVLDVTNGMARLLYVDLLLPLLPQDGNPLYIVGRSVVVHGPNGSLARLACANISLLFPLDALAQFNTSGVVGIVRFRQATPFDVTVVTVSLTGLGGVASGYHVNALQVQGGDCSTAGNVWDPTGVGGASTPLHTSDQFKVGDLSGKFGNLAGLAAVNYTFSDSNLPLFGQESIVGRSLVLSFSNDSAWLCSNVQHSSAVLQSTVNLNTNGFNGSVIFSQPTNDPFAATTIVVEFAVYPDLALPSVVSPSIVLPPTVASPSIVLPPTVASPSIVLSPTVVSSPPVTSVPSLVSAASEVIPVPSGVTSGITSAAGMTSGVTFAAGVTSGVTSAAGVTSEVTSAAGVTPTVSADTSKLLLSGQETSYLLTTYYGQLTLLDSLVPSPTPTGIAVGKREVPIARESFLRAKRQQPNSFLWSLQSGGCGSASFNPFNSSRLAGCSSTNQLLCSAGDLTGKHGPIITNRAVFTDLNLALVGPYSAIGSALLIQLGVNGPSVCVTLPPASLVIATPTSTSATPTSTSVTNATPTSTSVTSATPTSTSVTPISTGVTGVTPTSTSVTTTSTSVTNTVPLIPIIAGSAGGGAAILAILLIVTVICCCKCCRTSKYTLNHTLSKQTQAGHEELAVFKRKMHEPVIGVSLKSAAAVITLASMSFSSPHTLSQSMRRSHGVTRGQVDSTPRLVNKLDIGASVIVNGCVDSTPSTVPGSPKSVSEVIMNTGAIDSLVPGDAGHAHQDHTPKLTADQSGAEQLTGGDDKSAGLFRAIFDFEASSDQELTLRSGDLVTVLDKSDSGWWKGECEGGSGWFPETYVQPVASSTEEAVPGGVVSSPSMWEQEVAEYKALYDHQSSVSGDLSFKEGDTILVYWANSNGWWYGSYSNMEGWFPGSYVEPLLEPSVEATSKPSVEATNKPSVEAMSKPSVEATRKPSVEATRKPSVGRKDLSEYVSLEEPMATSEGPASGGLMVTSEGPASGGLMDEMLAALQSKRRVTELSDKGVATETQKSHDPVNASDDHAVLSHDTSLISRDQSHVLQKPTIAPKPVKAVVSTSVGITPQSPAVQDTSSEATPTSDGVVELNAKESSNKGTPLEVALSTSSEAGKMIHKAGSKQGTPTDVTTPIKEDKFTSTTTLSAPKTRRAPPPPPPSATKATADISTLPKTTPPELSQKTTPTSISKGTPPMPRTDKKQLADVSASKTTPPSKGPKIKPRPNISSPIAVNVPAQVPRLDISAPIPTDAQPAENATPISTASYYATPTAAVVAIQPRSLPSAATPTSSAAMPTSSAVTPTSSAAPPTSATMPLSLASPAAMTTIVVHSNTAYGALKPQATPPVVSERKPLASLRDNKTKPRRPPPSTPAPFASSRPHPFASKHDDTDGVMVAVPNQRVKKIAGFPSKRSIPPPPSIPLPPPPKRSLPPPPKSALPPPPSATLPSSTSIDINPPINATLIPAPKTPAPPPPLTFVFPSKAEDIPDPEAGYAPLTTSVPPAEKPLSSPVQLPPSLQSAKSITPPKPAVLPKPAVLPKPAVLPKPAVLPKPAVLPKPATQSKLHSGSLRRAITESELSSVVSLPSGEGSAKQTSQCFSNRTTGFKRFSHPKVSKQGIVPPQPPKRAISQKADTAAVAPDDVTTGTDMMQSSASIPIPKTRTIKKGSEPPSAVVPPPSSSSSSSADSEVKSAAKPERPPPPKRSAIPPKVLSPPNNASSNKSTSSFYRATQDYQAQREGEINFSKGDIMVLIDGSNPDALYGMLDNGDTGLFPATCVEPFSPQ

>Ephydatia.muelleri_18_CuZn Em0023g415a

MSQAVCVLEGQTVGGTIFFTENSDGSTHVTGMVTGLTPGDHGFHVHEYGDYSDGCVSAGEHFNPYKKQHGGPNDKERHAGDLGNITANESGEALVDIVDKQIPLTGPNSIVGRSIVVHADKDDYGRGGFDDSKTTGHSGARLACGVIGIAANQVSVVVKK

>Ephydatia.muelleri_19_CuZn Em0023g418a

MAQAVCVLEGQTVRGTISFTESGDGATHVTGTVTGLTPGDHGFHVHEYGDYSGGCVSAGPHFNPYKKQHGGPNDEERHAGDLGNITANESGEALVDIVDKQIPLTGPNSIVGRSIVVHADKDDYGRGGFDDSKTTGHAGARLACGVIGIAKPVVAKQ

>Ephydatia.muelleri_40_CuZn Em0023g422a

MISNGIKVILVLSILLPAVVPSQPPVSVPETTRAVSVMSGPTDVKGVIVFESLGHDWIRVTGSITGLQPGPHGFHVHKLGDLTNGCVSAGEHFNPFRQTHGGLKSKVRHAGDLGNIVANAEGVAMVDLVARQMALSGPLSIIGRSIVIHADKDDLGLGHEPDSPTTGHSGTRVGCGVIGRS

>Homo.sapiens_10_CuZnSOD SOD1

MATKAESNGPVKVWGSIKGLTEGLHGFHVHEFGDNTAGCTSAGPHFNPLSRKHGGPKDEERHVGDLGNVTADKDGVADVSIEDSVISLSGDHCIIGRTLVVHEKADDLGKGGNEESTKTGNAGSRLACGVIGIAQ*

>Homo.sapiens_11_CuZnSOD SOD1.2

MATKAVCVLKGDGPVQGIINFEQKESNGPVKVWGSIKGLTEGLHGFHVHEFGDNTAGCTSAGPHFNPLSRKHGGPKDEERHVGDLGNVTADKDGVADVSIEDSVISLSGDHCIIGRTLVVHEKADDLGKGGNEESTKTGNAGSRLACGVIGIAQ*

>Homo.sapiens_46_CuZnSOD SOD3

MLALLCSCLLLAAGASDAWTGEDSAEPNSDSAEWIRDMYAKVTEIWQEVMQRRDDDGALHAACQVQPSATLDAAQPRVTGVVLFRQLAPRAKLDAFFALEGFPTEPNSSSRAIHVHQFGDLSQGCESTGPHYNPLAVPHPQHPGDFGNFAVRDGSLWRYRAGLAASLAGPHSIVGRAVVVHAGEDDLGRGGNQASVENGNAGRRLACCVVGVCGPGLWERQAREHSERKKRRRESECKAA*

>Lingula.anatina_4_CuZn g5290.t1

MFFGILRCTFLILLIWTSCQATPATETPPETTPVYLNNVKTARLNMRMPTLDINAFYHGYAGFQSKDCDSAYEFKCKNRRCIPATWRCDRENDCGDRSDEADCVHFTRKSAPKPSVKSLTFPHGLPIGYPAKTGTCSTFPVYGQKYLWQCTNDHDCILDMKCCNDGKGKVCKPPVLESLRPYLSPQQLARMYTVGHCDMRPNTGVKHGISGDIYLVQRGNLLEVRVNISGLPTEGNSLKHGLHVHTYGDLSDGCASTGGHFNPANVRHGSPTDLPNKRHVGDWGNVERDSDGNVVTAFLDSVASLWGPNTIIGRAIVIHASEDDLGRGGDKGSSLSGNAGPRLACCVIGISNGNNLLQQG

>Lingula.anatina_35_CuZn g3127.t1

MASSGIVPLLLVLVFGCGWSVHGTYFPLFSSASRYFRFFPLADKPGECSTAPFYRVRSRLCNQDHLCPGDWKCCPHAYHGYICRPPKFESVPKTESRIESLAMQGVFAHCALKPNMGTPEPDRVNVHGNIDMIQRQGVLEVRVNISGLPLDGSQEHGLHVHAFGDLSGGCGSTKGHYNPTGVTHGAPTDVVRHIGDWGNVPQDKNGQIVTSFFDGIASLVGKNNIVGRAIVLHTGKDDLGRGGNAASLANGNAGPRLGCCVIGITNGQRVAVPAA

>Lingula.anatina_12_CuZn g17842.t1

MALKAVCVLKGASDVIGTVYFEQTSADGPCKVTGEVTGLKEGLHGFHVHQFGDNTNGCTSAGPHFNPSGKTHGGPDDENRHFGDLGNIKAGSDGKATVAITDKLVTLTGPNSVIGRTIVVHEGQDDLGKGGNEESLKTGNAGGRLACGVIGITH

>Lingula.anatina_13_CuZn g17843.t1

MRSNWIAQWVGVCVHFGDLGNIKAGSDGKATVAITDKLVTLTGPNSVIGRTIVVHEGQDDLGKGGNEESLKTGNAGGRLACGVIGITH

>Mnemiopsis.leidyi_50_CuZn ML01109a

MLFYVLLLTLIQLSVSTKKASCIMKLNGENTEVGKVTFSVHDNTQTVEAHLHNYAAIAPGLHGFHVHTLGVDGYDCGSTGGHFNPYKKYHGVLGLDKDSRHLGDFGNVLAGDRGEINFKAHMTVDPEGKVGYTMVPHTSTCKRKYYCSKKQELLGKCSRKNRCYQAAVENYHYTVTSPQFELEGENSIVGRAVVLHAGQDDLGQGGDDGSLATGNAGSRVACCTLHLEYAHGFGPYHGR

>Mnemiopsis.leidyi_51_CuZn ML011011a

MIFFILFLTSLQLSAANHHIRRASCSMKLNGKDKVIGKVTFSEHNNIQTIHAHLHERDEIVPGLHGLHIHTDGVTDNDCASSGGHFNPDGNKHGELGLDADLRHVGDFGNVEAGERGEINFKAVITVDPEGNAASEKMPKEDIHCRRKYYCTRKKENMGMCSRKPHCYQAMTETYHYTIHSPRFNLKGKNSVVGRAVVLHAGEDDLGLGGDEGSRKTGNAGERVACCTLELD

>Mnemiopsis.leidyi_59_CuZn ML01179a

MSLVLILLTVLVAETSATRASCYLRKHNDGRIIGTAKFYSITGRYQNVSVDLYDSGLTDGLHGFHIHEQGVTGCDCLTTGGHYNPDGVNHGGAGGAASSRHVGDFGNVESRQVLYSKIQVMSKASEVNILEISPKDRLFFTGPFTEISKTHITLKNATFKSIIFKIKTTEPKKYCVRPNQGIIHPQDSAIATVILQPIEEEVLTNCQGQHKFQILYTIRDNDQPSKSVEELWLVANPALVFHHKLRCFFESPNEAEQRKKLEELKTDVINHKDALKLISELRSKIDELKKENSKLNISVSETQKRLNLALKPSQFPDLLTYIVIALLGWLFAKFVI

>Mnemiopsis.leidyi_52_CuZn ML018118a

MKLHNNGTDIGEVSFSSGSGNTQEVSVFLQTDPSKIPQGYYGLHVHTFPVIEFDCSSSSTGDHFNPKGNRHGSPLSSANDRHVGDFGNIRAGERGEINYRGTYMVNPGKKITASALLEPVVYCEPYKVVKTVIREKRSNKKEKRGKKRRRKNKKVKLEYIKQDCYQPKWLQVEASIKSPLFRLEGEDSIVGRAAVIHAHEDDQGKSGTAKSTKTGNAGKPIACCTLQLV

>Mnemiopsis.leidyi_44_CuZn ML021130a

MAVLYKNSEQKGQTTSLDLVGIVQFTQAGPAAKLMVNINVFTDAEYFADGLHGFHIHQMGNVYGGCTQTLGHYNPLDVLHGGPDSAVRHTGDFGNIEIKNHAFTGEHTDSQASLYGQYSIIGRGVVLHETQDDLGKGGDLGSQKTGNAGSRLACGVIGISSEGYDNASSSVVSSLGLLLTTTLLRFLFQMMWALQETTKIPTMSLQNSICSEPAKPGCWTFLSHGTDEQERFSSPNHGSKIRKGTKMLFSELPVVQAIRYRSSIGNQKFKDLQENVDIKMKTRPQFNFAQHHFSALSTYGTSTTSYFLEKGFKKSETPYFDRWFKNDEEDEYKVTDGTTITPSLPPPKSTYTRLQAEHELITASTALNAFQGFEWTNTRPNENSTNMTAQSSTPTKEPKKTTPPPRPEEMLWFCYTSGYHPHPSKAAENFISRTFYRCWMYCKYYKNCVLFTFTDVTRGRCLLFTKRYEDMVPGDVGTITASRECMDGKRCGGQKNTIDHISKLSYKNGGYIIKNRKTKKCLAVKVAEKLVEVGSRLHNLNTRKKIAKKLNLKWRGCYHRQVQKWFVILKDPIINLPLAGKDPSHPVVSSKVVISPADPNNKYLSGETLCLTWQRDGLKIQKCEKTLARQALQLNYRRLPYQECRISLSNFNLPNNPETYIPIDFFGEVPSKEVKYEELLYKKPCRIENSSINHGYIIDQEKRVPFQLPGDNITIKCWDGFRVNLDKVKYEETYSVTCSKDMSLLKCVKKPPPTEPPTQPIHVTSVYLIT

>Mnemiopsis.leidyi_27_CuZn ML073256a

MNAICVLRNDKESFGCIKFSQASEGAPTTLKVEINNSSLTEGEHGFHMHQFGDNTNGCISAGPHFNPFGKTHGGPTDEVRHVGDLGNVVVDGSGVCSTEITDPQVSLFGANSIIGRTMVLHAGVDDLGKGGNEESLKTGNAGGRVACGIVGLCS

>Mnemiopsis.leidyi_53_CuZn ML08913a

MPQLSIELEENCMLLRFFSEPNLEYNANLLLKDQAQWIDDVPKDICHSDPSSEDSQNPLRVKRRNALPECRAVRGNPGNCDRTGAIFKKRGRVLGDLGNVRAGERGEVNHYHVIKVDPAAREERVGLVEPVRHCPPVPYKVWRDGSEEKIENMEQAEMELQKLRMKGKDLFLIVYVEPDCYQPVYNRDTHTLNKPLFHLQGKFSIVGRAVLLQVGSLTSVDGSPGDAIACCTLQLEQ

>Mnemiopsis.leidyi_54_CuZn ML26794a

MAIIRHNYFLVSAIVVIGTIVVIGQFNRSVQNQGFSMIKLITMVLIGAAAAATQGSWMPVKPDIDLETTPLEIKTDSAVGSGDKLYVYFYTSQGDYAGRVYLRFTSTLQYYIEWCSTSWTNFPTNPPADVNKVWRITKTRTSGIRLQIQCNDVEVLNILMSDTTCSDSRWSTFWNRDIEKIYFGNSDTASDYYKLSHQGNWTPVKPDIDMEATPLEIKTDSAVGSGDLVYVHFYTSQGDEAGRVNLHFTSTLQYKINSCITTWTNFPTNPPADVNKAALQLSWAKYEAECTMKLHNTEIEIGKVVFKSEEGFQSIQANLEGDPEVIKEGKYGLKVYENSVLVNDCSAASTGAEEIEVGTVQAGDRGEINLKMSITIDPLGSPHSFLLSEPAISCAPIKVKIYADGREEPYVARKRRESQRKKEKLKEKKGKKEKKRKRKERKERKDSTAKKSTAKKCTKKRELKGKCTNAPTLEALKDIFYVIEKKPDCYSPKSDTYKITMNSPKFQLDGEDSIVGRAVAALQVSWAEYEAECTMKLHNTEIEIGKVVFKSGEGFQSIQANLEGDAEVITEGLHGFHVHEKPVMGNDCGAASTGGHYNPDGSNHSNLMSPSGERHAGDFGNVMAGQRGEINLDMKITVDPAGLPSGTMLGEPSMRCAPIKVKVYPDGREEPLDGPHEVRMKRSGKRKSEIRKTKRKAERKLRVLRNARRKGNKRANAQMRRHLSLWKKCPTVTPRNALPTDGPCQPLSLNFKEVNPLWDERWFYMLDGTTWDEEGTRGV

>Nematostella.vectensis_41_CuZn EDO43919

INVRLRGVPPLTVHGFHIHKSGDIITKGCQSAKGHFNPYGKTHAGPRKRDRHVGDLGNVWSDYHGNVRTSFFDHMVSLYGPDSVIGRSIVLHAERDDLGRGIGEYRTGSLATGNAGARLACCVIVH

>Nematostella.vectensis_14_CuZn EDO42041

MVIRGVCCLVGDNEVKGVIHFTQQAPDGPCTLRGRITGLTEGKHGFHIHEFGDNTNGCTSAGAHYNPHGKMHGAPEDKDRHLGDLGNIEADANGIADVSITDCLVSLTGQCSIIGRSLVVHEGMDDLGAGGHELSLTTGNAGGRVACGVIGIAL

>Nematostella.vectensis_15_CuZn EDO42040

MPIQAVCCMSGTEGVKGTIKFVQEAEGKPCKITGTIEGLKAGNHGFHIHVYGDNTNGCVSAGPHFNPFKKEHGGPSDENRHVGDLGNVVAGDDGKACIDMTDALVTLVGEHSVVGRSVVVHADEDDLGRGGHEDSKTTGHAGGRLACGVIGITQAS

>Oscarella.carmela_95_CuZn m.11490

MSFLSAFICLLVVTASVVKGAHLPALVNIDYNGIKGVIRINATEGSGTVAFDFHDLHTYNLANFVNMTAAIHQLPAPYRSKCQPTATDVGLIYDPTDAMSGSNYTKACQSDATKCAVGDLGLRLGKINHESNGDVLKTVFPGNNWYLSGSDGIVGRTLVLYNYTGYAFACGIIERHVIGSKKEVIRKSLLIAPVGGEVTFRQVIDTKTGQRFDAQIIVDLFYVDGSNTSSGHGWYLDDGQISSDANCAALNSAPATNAGCSRTNQAACSQNDLTGKHGTLSIHSGKGLHYFFIDTNLEVDTFVQKAVAVTDVKKSTSLSCGNVYNEDLQLGARFDGAVKGTVMFKPAIGGSIVSVNLTNMNSTAGTYHIHVYPVQNGDCMTTGGHMNPFGIKGTPQNGSADMYEVGDLSGIFGKFSGKNHVVFQKFSSNLKLTGRFGVLGRSIVIHSDKDGSRWQCANIVPMAPGTKKLTSLAKFVGKFEGMVKMIQWQLGDGRLTDTMVDIDIKSVGESETKNHNWHVHNRPAASFPGFGSKNCASAFTSGHYNPYDVDLGDNYKSECSVSNPLRCELGDLSGKHEKYNIFPKTAKAGRGYSSDPNLPLMGPLSVIGHSIVIHAENAGGPRISCASLYPENHKFFSVLFKKPSTINEKDIQSSIAQGLKVSADEVKFLSVNTDEESNCTSATFVVADGANVPDGNFSSIVSDGNFGNYKVDDDCLSEMPTNTAACTAFSIFTLLVILLVKNLL*

>Oscarella.carmela_43_CuZn m.19442

MCFTNTAQIGAILLVFLFIGTSGQCNVGWKSFQGNCYKFGANSKQTLVEEPAVATWYDARLVCLNLGADLVSIHSTAENEFVRKEMKGDSWLGYFEQKSSGPEIVDYWWSDGSSTHYDYENWAYNRPHYGSGTESGGQTSTTCAFMYSGLPHRGRRWRRHSIPSATIPGRWFGSVQCATAKKYYVCKKSACCQDLVAVANLVSNSDRTVTGTVLFHQLAEGGETLVRGVVKGLQAGSRHGFHIHQQSDNTLGCSSYGGHYNPFHAHHSGRFDSERHVGDLGNIEFEETGVASFTFRDSLAQLLGPQSVLNRGVVIHARGDDLGKGGDEGSRKTGNAGHRVACGPILCRSCMDNLAAEAHLHSDDGNLSGVLALDQVIYWGVAGVVSGNESGLPEPAEYTHSRVTFLSYDDYSKADKKCDRLSTPPEQAPSPRDRTSGIIDLIGQVAVYHLSQVDSGNNVTKTATLCGPVWPVAHVTCACADLKNNENKLYGHICFSQQSCGGPVKIVGKLSGEDLPSSIGFSKYPYGLSTDVSCPAEDYDTWHELSSHLENGSLVICDESSLLSLTGFGSDIGRSLIVSSGDEDSRKTCGTISYRPVCPSSSCN*

>Strongylocentrotus.purpuratus_57_CuZn SPU_005072

MTISRDELSQLFDGFLDKLEQWGDDPAKVDEITHQVEPHIVNEAEENPAKVEEIARQVEPHIDNEAEENPAKVEDIARQVEPHIVNEAEESPAELSQKLISILRELHEVESDMDTRRTPPPPSLEQDVKSADAVLIELLNESRRMTESVNDSDDDDHNEYEGEDQSTRSPASVITMSNPPAAEDLPNDNENDTHLYDFAACELRLNERLEPLTLAKYHRFRGTVNLRQNRSNGSSDVTIRLFGLSPSNSNSQGHGVYIREFGDLGDGCQRLGPIFATNRNPDQQVGSTGLLAVVSPDESGFVQYRTTEVGHFDLAGRNSIFGRSIVIEQNQDTYNAPLGCCVIGVTRDAEEWM

>Strongylocentrotus.purpuratus_28_CuZn SPU_006214

MSVKAVCMLVGEAVKGRIEFEQGEGSNSVSVKGCVSAGGHFNPFGKEHGAPEDEMRHVGDLGNIIADASGKVDVNLSDKLLSLSGPQSIIGRAVVVHADVDDLGKGGHATSKTTGNAGGRLACGVIGIQA

>Strongylocentrotus.purpuratus_58_CuZn SPU_014006

MMNVFNVCKRATWLSLLSMVFLSVIIESSQAMTISRDELSQLFDGFLDKLEQWGDDPAKVEEITHQVEPHIVNETEENPAKVEEITRQAEPHIVNEAEETPAELSQKLLSILRELHEVESDMDTRRTPPPPALEQAKSADAVLIELLNESRRMTESLNDSDDDDHNEYEGEDQSTKSPASVITMSNPPAAEDLPNDNENETHLYDFAACELRLNERLEPLTLAKYHRFKGTVNLRQNRSSGSSDVTIRLFGLSPSNSNSQGHGVYIREFGDLGDGCQRLGPIFDTNSNPDQQIGSTGLLAVVSPDESGFVQSRTTEVGHFDLVGRNSIYGRSIVIEQNQDTYNAPLGCCVIGVTRDAEEWM

>Strongylocentrotus.purpuratus_66_CuZn SPU_022576

MLVRWHLTTLYNIRLEGINIGLMLASLVSASEHIAKFSMKGMSGRVIFTEDPSTMGVHIQVDPLIGLEDGQDYEWSIRTLPMLYDREDKCAEEYIGPVYRNLTAAIGRLTNVTTGSMYVDPMPMLDGNDNISGRTLAIEDANSVVYCASIIPSDEVITAVAQFGTPLAGTVTFRQRAVAGAVTTIFVDVYDVTEPIIAQDYDWHISDNTGITKDMTVEEWCTATTAEAYADSGDLTPKFGSVNVKTVLLRQRYFFIDTDITLNGAESILNKSLVFSLPGDAGTRQACSPIRLVKSKTVRAEFSQNNVTGIVTFTQASLWESTNITVDLENLRQMAGGYHVHKFPVPMRYLTSDFPGSADNVAGHFNPYNIDTTADPDYPNGTNDQYELGDISGKFGSLAGQDDFSGTFEDWNMPLFGANSIVGRSVIIHLASSERWAYGLIEYPAPVRTVTSVFTYPLVGQITFRQDAEDPYSDTSVFVDLAHSDGEDTTVEHDWHIHVAKLGDDHLDQTGRCSSTLGHFNPFAVKLTEEYATECGIDVTRRCEIGDFSKKYGRITVPGSVRTQVGKYFFTDVQLPLSGVYTVADRAVVVHVKDGAPERLGCGDTLDVFQATATAEMWIGVSDSGTGTVTTPVTGSVTLTQDSEFDDVTITADLDGLNGVVAPWHIHVLPVPYSDASPCSPTSTQGHYNPFGIIGSPPAGTFDYYENGDLSGKFGYWNDLQTISTTYSDSSLSLFGSRSVSGRSVIVHRSDDGSRHRCSSLMPVLGDGDFRIEAGVDISNSDFTGYLLLGQTHFSNGRLGDTTILANFSPVDVLISDVYDWTIRTDTISSSSCPSSNSVFNPNTVATDGDDYPGTTNPQTTTLYDNHPLEHLPPDNYPRGVSVQTQTTDTVRLFTNLFANYSKDIRPVERASQSINVLYGMAIRQIIDMVVPFDKLMSGARICQPFELDFTNQPDIVAVSLRIGQNERLEDELLFPLAFSTPHRRKRTSPQDPGGQKDFDEKLTTVARKVAKREEIDNLGKSLGFGPEDIQRYFDTNMKNSEVSYMGTLSMLRKWRKKQTEAKECEALNRVLRKAGQIRLIDEL

>Sycon.ciliatum_96_CuZn scpid21456

MERYLRMQLLLLLLAVIGELALVAQSRRAVAEFSLNGVRGSLVFEDSSSENGTSGSSGNWVQIDSRRLHGIDHLGPLTWHVHLYPFKYTISPMHRCTSTGGHYDPHGKATAVRYAQLCKPSSGNPFRDCEVGDLSGKFGPLRRTLYRDRTPNMSVAELIGRSVVLHVFSGERFACANIVEVNRFVVAPYGCQRRHVDRMRTLEATFSTPVAGSVFIRQLNYKSNRGIMSPGQPNMAELPVTVIFSDLRTVSGLNNPMYSTVGLNWTVHIRQPSMAPSAGGAVQRSCGTGPVYSPMTMGRLRVQTHANTDSRTLLYTVDRLLSPLDRLRQRDLVLSDAVTSQRVACAWIEEVQPFSATAEFHRGIGQLRMVQSSVFDSTQVLVNLSKLANTAADYTIRETPLSRFTPARMEPDCTPARSSYIFAPFPLPTLSRHRDGGTPDMFTTGDLSGKYGSLRGLFAFHESFCDGNLPLFGPHSVIGRTVLVTQRGAARVSYTCARVTQQLTPGSRTLLAAASIQGSFDGRIVLQQVKYPDGSLGDTLFTLTTAIRAGMSREAGNFMFPGMRHGGIGGMAMRPGGIDGTAMMPGGNGGTAMMPGGHSMMQGQVPLYKFHVHRNRIGVPSSLRSVCDSSYTGPRYNPHNVTVDQSYSSRCSATQPHLCELGDTSGKVGFLRATSETKLRVDPELPLIGQNSVVGRSFVIYIAFPESGPIACGNILPKDAVDTQVTFLMEDATGVSLTKFKYRVALRLHISVNEVLFPEAYRFGRCTAVSFALHGINAPGLVELVRRTDLGEFTHNQKCEDSMMRNSDVLYTNPKVVEWLPSWLPSPPSSGARVRQDPGTPYAVSLLAVMFLRVLSC

>Sycon.ciliatum_70_CuZn scpid34776

MCTVPPSSSWCRFATSTSFLIGLVLIGISTLVQGQDVQLAAMFNSGGVSGQIVFQQASPVSSVIITVSLTGLSSTGTQSLPWHVHQFPIDVDLPPSQRCSAASVGGHFDPDGRAGTNYTQRCTSDPALCEVGDLSGRHGGLSNAVFNDSTLALSRSARSIAGRSVVIHHANGSRWVCASIGYSAARVTTAIARLQGTLRGTVVFRQAAGSNDTTVLMRLYQSQLLGALSYHVHAKPVRSNTDQTVVRCNATGGLFDPFGMASAANYSALCAAQRNACQVGDLSRKHGTIPASQNFTTLFFTDTYLPLSGVNTIVRRSIVFHNSSSDRVVCSNIQELRSRRATAVFDYGGVRGRVELNQQSPWDNTIVSVQLNGLMMMAGGYHIHEWPVPWLDLLSTLPPLSTRFCGSQAVGGHYNPFSASTPLPPGLTQDFYEVGDLSAKFGGLAGLNSLNATYSDTYITLFGPYSVVGRSIVLHNDIASSPRWQCATILLESTTLSVARAYFTGSVVGSIWFIQDESSKPSDTTVFSDLRLISGNSTATSHNWHVHSSPLGADGLATTLRCASAGGHFNPHNVDLSPALYQCSLSNQLLCEVGDLVGKSSQLMLSTGASAKHFYTDVDLPVAANGILGKSVVIHGTLYGGRLACADISRFAVQRGVADFGTKGRIDFSQIGPFSPTQLAVNVANLAGMAGGYHIHQLPVFSHCASTGGHFNPLQTIYSGGVVPVGGTLDAYEAGDLSGKFGSLSGLASLDRVYSDLRFVPLLGKLSSIGRSVVVHFPNGSRWFCANIGAPSSANTTTLTATISSSFVTGTVTLSQIPSNPALTFVTLDLQLVAGTGGSTRRKRQTTSSAPYTWLLTSSPPSASCNGIAIDPSGAAQASDYAQRCSQSQPLLCSTGDLSGKLGNIMFSGSSSVLRQFSITENLPLSGSNSVASTTVVLADTNGNRACAQLQTPSTAPTTSATTSPTTAAPTTGNPSTAPPVTTPPTMQSSQGTTPGPSTQPTTVPNPSTQPTTTPSPSTQPVTTSGPSTQPVTTPSPST

>Sycon.ciliatum_100_CuZn scpid33674

MVNVQIRETQNRCMERRSSLALFGASLWHQHQQQEQRSSSGVKMPSRRCLYRRAWNSPVALHRRMVAVLFLLFIVVMSLSKTSCRAQLNARADFHMNGVGGNILFSWNPQSHQMTINTMGLKGLSPQLLTSGLTWHVHEFPFKLNSPDPCSASSTGGHFDPLGRASAPNYAQICQSQMSMRFRDCELGDLSGKFGTLQLREYVELTPNLSLSGNFGFVGRSVVIHRNGARWVCANIEPVLQHTATCAQAEGSNKRRLLVAKFKWPIAGEVFFRQVDTRDPAVRPTPPIPAGQPVHTSVVNISTVVHSHLYWLDQTAAMSPAQSATSFLPPSQMAGNSFLPAPNQPAAALPMGIRTFQAGPGASPGYMWQVEAHPVLEKAQFSPNPQSRCSGTGATLVHLTSMLGPVSVGIDPNSASTSKSLYTTNNSMFYPIDNFIRRSLVLLDPTRRRIACATIHEVQPATALATLPHDMGTVELSQSSEFDNTEVKANFNKLFNVASDYGIRTLSMKQSNGRENACNERQTGPMFNPLRTQSPPTKRGSPNLYQIGDLSGKFGELRNRFSYRRRFCDPNLPMFGTNSVVGRSLVIDHQTTGNAPIACARLDRTPQHNSRLLKAKATFTSPVRGTILLEQEQFADGQLGNTHITSYGLRNFNSWQTDGHMYHIHINRIGILSTQSEMCSNAHAGPHYNPFNASVDATYPKQCSPQNPYRCELGDTSKKTGKIAVRNVPQYHIDPDLPLTGPHSALGRSMVIHNPNGAAARLACGNVIPEDSHDVVMTVLVSPLQFKAQHFKQRMAGAMRISAQDVLYLKSSPLGPCTAVSFALRHPQAEQLASTILLTDLGPYTHDVRCADPSQLVAATPRNGQRDRNDDDDDD

>Sycon.ciliatum_101_CuZn scpid49237

MFSPATSSGSLATTFLVLLLPGLSVAQSSFTLLSAYFSNGGVAGHVDISGSIAIHTRASTPEPITITLNSLTGFPSGGSGALRWGVNERAFRPGVQSNACGTESVGGLWDPEMKATAQDYATNCLPTASSRFTDCAAGDLSGKFGPPSSSGTFADSSALRLIDFLGRSLVLRYSNGTALACANIVYVYPDGNAAGTSRPSPTTSCQNMPAPSAFQAQFVWPVAGDMLFRYYSRASSVIGVNIAAQLYDVTGSGNLTGLSWTAHPAEISHAEMTRSKGARCTGAGMPLVNYTAAYQAEVSVGATPERGHQILSFSGNGSHAPVNGSSIKSIILWQGSEALACANVYSSKLMSVRASFPVTGNTVTYTQQSPLDTVLRQVSTATTLGSTGGTLRIRNSPVNPLARSTQHGSDNGMCSSTGLLFSLTARTAPVSVTDGTATSSVGCVESHTLFGYSSVVHRSATIESMSGSSPIQQCATILPLNLPNATRTLQARLTFPSDSEVQGSIYFTQYAYDDGSYSDTYALFRLGNSSATTSGHNFHVHVERVLPYNNSLCGVTGGHYNPLNVNLSSATGYSTACGPRSQLRCEVGDLSGKIGQLTIDGTARMYPIAQLPLEGPNSVFGRSIVIHAANGGAPRIACANIIGVAAADAYIAFRVPAGGFMAGAFSTAVQTWLSTSSAVEQINITHVTSTVDPGTNCVFVNFAIESSSETVSQRLARDVLTAPLGIYSHRGSCLDPSLVSSAASPSILAGSSPWLLIFSLCTAIRMVFLLLR

>Sycon.ciliatum_68_CuZn scpid23623

MCKPVCTRPIPVFLVLSTLAALGHCRLPSLEMPGFVAVNPDRPVVSHPDQRVSWSTWSPWSSCSASCGGGLQKRIRFCPRQEQHEHGCYGAGVQVRRCSLVPCFGRGLLDSWSAWTQWSSCCGQREQLRTRACRVRNMDTQKGRCHGSSLEVRACDSSTCNEMHAVASINMNGVVGNISFRQSSLSSAVSIKIDLHGAGLDARGITWHVHEYPLDYSTLPAQQCMAESVGGHYDPYRKAGLSQYAMRCARNAADCETGDLSGRHGELTLGAAYEDRFLQLRGKESIVGRSLVLHRPDGSRWVCATILPASPAGGNNMLTAVARFTGPVIGGTITIRQPAGDPKADSTVLVDLYRTDGAVVDPLPYVWGLHTRPVIVANDTLHTDVSIRCQSAGSRYDPLDARQNVNYVRDCNNRQPENCAFGDLSSKHGRLSVPSSGHLRAFYTDSFLPLSGPYHVVGRPLLISDGVGDSQRIACSDLLTLESREMSATFDMQGVKGVMVFSQQSPWDPTWVTLDLSGLAGQAAGYHVHEFPRSVLGTVQYGSACSAQEVGGHWNPFRAPFPVTVPPGFASTPDLYEVGDLSGKHGSLANLQSVQQEMYDGQLPLFGPHSIAGRSIVIHRNAPNAPRWVCANIEMPQTAKRIAVAHFYAPLAGTIYFRQIDAFETSVFVDLARTDGGPSSAGHHWHIHANPIGNDRFPSVAQRCQSASGHYNPFNVSVAQLTYNCNAHDQMRCELGDASGKSSPLDFMATGSNRFVYTDVFLPLQGRFSVLGRSVVVHTANAGAPRLACATIEPYADRNAQASFGRAGVSGFVSFRQGSPFSSTVVTVQLSGLRGRASHYALHYLPSAEQASSAVAMASPPVTTPQESCSLTTIGQVYNPMGARFDSTVAANTASSASNDNSKTFSADTVAIGQLSWKFGSLERLTGVVSSFADTHYLPMSGPLNFVGRSVSLHDRQADTPWVCSNVNYLGGRLVTAVAKFTGHLQGEVRFEQVDNLPTQITIRLQHALQAIATASHQWTINVMPVGTDAMAVGNQRCMSAGPVHNPANVSVTDRLYLRRCSSLSATCQQGDLWYKHGMLRLAGSQQQSSLRVLLTEEFAPLSGPFSVIGRSVVIMSQEMGAERIACATIQQQ

>Sycon.ciliatum_97_CuZn scpid20938

MERYLRMQLLLLLLAVIGELALVAQSRRAVAEFSLNGVRGSLVFEDSSSENGTSGSSGNWVQIDSRRLHGIDHLGPLTWHVHLYPFKYTISPMHRCTSTGGHYDPHGKATAVRYAQLCKPSSGNPFRDCEVGDLSGKFGPLRRTLYRDRTPNMSVAELIGRSVVLHVFSGERFACANIVEVNRFVVAPYGCQRRHVDRMRTLEATFSTPVAGSVFIRQLNYKSNRGIMSPGQPNMAELPVTVIFSDLRTVSGLNNPMYSTVGLNWTVHIRQPSMAPSAGGAVQRSCGTGPVYSPMTMGRLRVQTHANTDSRTLLYTVDRLLSPLDRLRQRDLVLSDAVTSQRVACAWIEEVQPFSATAEFHRGIGQLRMVQSSVFDSTQVLVNLSKLANTAADYTIRETPLSRFTPARMEPDCTPARSSYIFAPFPLPTLSRHRDGGTPDMFTTGDLSGKYGSLRGLFAFHESFCDGNLPLFGPHSVIGRTVLVTQRGAARVSYTCARVTQQLTPGSRTLLAAASIQGSFDGRIVLQQVKYPDGSLGDTLFTLTTAIRAGMSREAGNFMFPGMRHGGIGGMAMRPGGIDGTAMMPGGNGGTAMMPGGNGGTAMMPGGNGGTAMMPGGHSMMQGQVPLYKFHVHRNRIGVPSSLRSVCDSSYTGPRYNPHNVTVDQSYSSRCSATQPHLCELGDTSGKVGFLRATSETKLRVDPELPLIGQNSVVGRSFVIYIAFPESGPIACGNILPKDAVDTQVTFLMEDATGVSLTKFKYRVALRLHISVNEVLFPEAYRFGRCTAVSFALHGINAPGLVELVRRTDLGEFTHNQKCEDSMMRNSDVLYTNPKVVEWLPSWLPSPPSSGARVRQDPGTPYAVSLLAVMFLRVLSC

>Sycon.ciliatum_98_CuZn scpid21234

MERYLRMQLLLLLLAVIGELALVAQSRRAVAEFSLNGVRGSLVFEDSSSENGTSGSSGNWVQIDSRRLHGIDHLGPLTWHVHLYPFKYTISPMHRCTSTGGHYDPHGKATAVRYAQLCKPSSGNPFRDCEVGDLSGKFGPLRRTLYRDRTPNMSVAELIGRSVVLHVFSGERFACANIVEVNRFVVAPYGCQRRHVDRMRTLEATFSTPVAGSVFIRQLNYKSNRGIMSPGQPNMAELPVTVIFSDLRTVSGLNNPMYSTVGLNWTVHIRQPSMAPSAGGAVQRSCGTGPVYSPMTMGRLRVQTHANTDSRTLLYTVDRLLSPLDRLRQRDLVLSDAVTSQRVACAWIEEVQPFSATAEFHRGIGQLRMVQSSVFDSTQVLVNLSKLANTAADYTIRETPLSRFTPARMEPDCTPARSSYIFAPFPLPTLSRHRDGGTPDMFTTGDLSGKYGSLRGLFAFHESFCDGNLPLFGPHSVIGRTVLVTQRGAARVSYTCARVTQQLTPGSRTLLAAASIQGSFDGRIVLQQVKYPDGSLGDTLFTLTTAIRAGMSREAGNFMFPGMRHGGIGGMAMRPGGIDGTAMMPGGNGGTAMMPGGNGGTAMMPGGHSMMQGQVPLYKFHVHRNRIGVPSSLRSVCDSSYTGPRYNPHNVTVDQSYSSRCSATQPHLCELGDTSGKVGFLRATSETKLRVDPELPLIGQNSVVGRSFVIYIAFPESGPIACGNILPKDAVDTQVTFLMEDATGVSLTKFKYRVALRLHISVNEVLFPEAYRFGRCTAVSFALHGINAPGLVELVRRTDLGEFTHNQKCEDSMMRNSDVLYTNPKVVEWLPSWLPSPPSSGARVRQDPGTPYAVSLLAVMFLRVLSC

>Sycon.ciliatum_103_CuZn scpid10258

MRMVGLCVLAVTLLLTYCSLPSTLAQTCQDERAVAYFNDNGFRGRVIISKDAGELSFNASGLQLATGQFSLAIHELSAFVGTSESSCQGIGAVYNPTSAQAGMELGGLSTLPALANQVLSVTTGLNMINPYGVIGRTLVIQATSDGTATSCATIQHVYATSGSVCPEPDQHNYEVTHVAAFNGDISGRVFLRQASRATTQGTFLTTVYSHLYSTQGRAASTGHQWAIRSGLCTSLGSFVANLSVTADGQVPISAGPSGYAGRHVTSLCLDSQLPTGTTERSLAVYNADSSLLACARVHRVPHRSIRATFTGDTSGYVAISQGSPFTASSLRINLMSNTVSSVGSTINTLPIQSGQGSSAGQVFNPFMSQTNVVGRIGDKLGNATQAQVCDRYSTLRGLNSLAGRSATITYNSQQSSMNLEEQLAGADRVLRARADFSGAVTGTIYLAQVAREDGSRGETLITSSGLTNAAQSMQHNMHVHVNRIGWQNDASACGGQFTAGHYDPHGVARNSTLYSAECGQMYPHRCEVGDTSRKTGQFNLDGSGFVRHDVNLPLDGNYRVIGRSFVIHAPNGGGPRVACANIVPMTARTFQLRFNVSALNGGNIDLADIRNRLQQILNDNGPIQASIQQNYVDAYNTTELGYIRGVVDGAQANRAIDLLAQCQMLGPYTRPDCPSSTSTATLPTAAASLVALLIATTTLLGLIF

>Sycon.ciliatum_8_CuZn scpid78906

MSVNAACVLAGDSAVKGVITFTQAEGATTVTGEVTGLAPGNHGFHVHVYGDNTNGCTSAGPHFNPSNKTHGAPCDENRHAGDLGNVVAGDDGVAKVNITDSQIPLSGPNSIIGRTVVVHADPDDLGKGGHELSLSTGNAGARVACGVIGIAK

>Sycon.ciliatum_99_CuZn scpid21742

MERYLRMQLLLLLLAVIGELALVAQSRRAVAEFSLNGVRGSLVFEDSSSENGTSGSSGNWVQIDSRRLHGIDHLGPLTWHVHLYPFKYTISPMHRCTSTGGHYDPHGKATAVRYAQLCKPSSGNPFRDCEVGDLSGKFGPLRRTLYRDRTPNMSVAELIGRSVVLHVFSGERFACANIVEVNRFVVAPYGCQRRHVDRMRTLEATFSTPVAGSVFIRQLNYKSNRGIMSPGQPNMAELPVTVIFSDLRTVSGLNNPMYSTVGLNWTVHIRQPSMAPSAGGAVQRSCGTGPVYSPMTMGRLRVQTHANTDSRTLLYTVDRLLSPLDRLRQRDLVLSDAVTSQRVACAWIEEVQPFSATAEFHRGIGQLRMVQSSVFDSTQVLVNLSKLANTAADYTIRETPLSRFTPARMEPDCTPARSSYIFAPFPLPTLSRHRDGGTPDMFTTGDLSGKYGSLRGLFAFHESFCDGNLPLFGPHSVIGRTVLVTQRGAARVSYTCARVTQQLTPGSRTLLAAASIQGSFDGRIVLQQVKYPDGSLGDTLFTLTTAIRAGMSREAGNFMFPGMRHGGIGGMAMRPGGIDGTAMMPGGHSMMQGQVPLYKFHVHRNRIGVPSSLRSVCDSSYTGPRYNPHNVTVDQSYSSRCSATQPHLCELGDTSGKVGFLRATSETKLRVDPELPLIGQNSVVGRSFVIYIAFPESGPIACGNILPKDAVDTQVTFLMEDATGVSLTKFKYRVALRLHISVNEVLFPEAYRFGRCTAVSFALHGINAPGLVELVRRTDLGEFTHNQKCEDSMMRNSDVLYTNPKVVEWLPSWLPSPPSSGARVRQDPGTPYAVSLLAVMFLRVLSC

>Sycon.ciliatum_69_CuZn scpid32648

MLCYTTCAEMHAVASINMNGVVGNISFRQSSLSSAVSIKIDLHGAGLDARGITWHVHEYPLDYSTLPAQQCMAESVGGHYDPYRKAGLSQYAMRCARNAADCETGDLSGRHGELTLGAAYEDRFLQLRGKESIVGRSLVLHRPDGSRWVCATILPASPAGGNNMLTAVARFTGPVIGGTITIRQPAGDPKADSTVLVDLYRTDGAVVDPLPYVWGLHTRPVIVANDTLHTDVSIRCQSAGSRYDPLDARQNVNYVRDCNNRQPENCAFGDLSSKHGRLSVPSSGHLRAFYTDSFLPLSGPYHVVGRPLLISDGVGDSQRIACSDLLTLESREMSATFDMQGVKGVMVFSQQSPWDPTWVTLDLSGLAGQAAGYHVHEFPRSVLGTVQYGSACSAQEVGGHWNPFRAPFPVTVPPGFASTPDLYEVGDLSGKHGSLANLQSVQQEMYDGQLPLFGPHSIAGRSIVIHRNAPNAPRWVCANIEMPQTAKRIAVAHFYAPLAGTIYFRQIDAFETSVFVDLARTDGGPSSAGHHWHIHANPIGNDRFPSVAQRCQSASGHYNPFNVSVAQLTYNCNAHDQMRCELGDASGKSSPLDFMATGSNRFVYTDVFLPLQGRFSVLGRSVVVHTANAGAPRLACATIEPYADRNAQASFGRAGVSGFVSFRQGSPFSSTVVTVQLSGLRGRASHYALHYLPSAEQASSAVAMASPPVTTPQESCSLTTIGQVYNPMGARFDSTVAANTASSASNDNSKTFSADTVAIGQLSWKFGSLERLTGVVSSFADTHYLPMSGPLNFVGRSVSLHDRQADTPWVCSNVNYLGGRLVTAVAKFTGHLQGEVRFEQVDNLPTQITIRLQHALQAIATASHQWTINVMPVGTDAMAVGNQRCMSAGPVHNPANVSVTDRLYLRRCSSLSATCQQGDLWYKHGMLRLAGSQQQSSLRVLLTEEFAPLSGPFSVIGRSVVIMSQEMGAERIACATIQQQ

>Sycon.ciliatum_104_CuZn scpid17636

MLTRLAVLCIVVAVGVIGDHHLMMDEAKGLDKAIAFFAMNGFHGPVSLYLNRTTLHVDMRKLNYSYPKPLAIDVHRLSFTVGMSGSPCESIGDHYNPTNNPMLALGDLSGSPGGRLLNATTRRFAIETNLTFTGHHSIIGRSVAFHEEGNETAVACANIVHANMLTDKPSCGSMSTDNDQDTLEAVFNGMIAGRITFRSLKSFGMGVSNSTTILSRLFYTNGNNASRNHLWHAHNNSVGDNGDCINLGAISQDFTSGSGGHAPHLLTIGDGDGDEDSREVHNLLQAAPFGNLIGKSLAIHRPSDLKIIACANIAKVHPRSFVAKFSDNSEISISQKDRYSSSEVNTNLKSRASKVNINKFKAGQMSGGCGDAKVGGVFNPFNKMGSDMMKPERSPTGDIGGKFGRMMQGQDCDNSLSLFGSNSVGRRSVSITVGGQVWCANLTEKLATGEKMMRAKAMFNMSGSVRGMMSIEQVVRSDGTMGITTISSDGLMNMDGASMKHNYHVHIGRKGYQNTSAACGSAYAGGHYNPFNVSVTSDSKYSTDCTSDNPLRCEVGDTSKKTGTIDLDTKPLIGQDSFMPLMGGQSVMGRSFVIHSTNQGAPRIACANIIPMSAYDVQIGVKTSRLSGGFSAATFNQRVKDSSSMAKDVDVLYTTMNTDAATGCTYINAAIDGPNAQTVSEQLSRADLGTEYSQMASECTQGVSFASQLSSASTVSHVASLILLACAAVLLL

>Sycon.ciliatum_102_CuZn scpid35317

MDASVSALLISVLLTITTMPCSAADVQAYFTNGGVSGRIIFSGSFSSSGGVTINTTQLNGYQQSGGSGILWHVHQFAFPTGAPDLCGAAATGGPWDPGSRMANPNYASQCVKSAWSVCSLGDLSGKIGPLSSGQFADGSTLGLLDLLGRAVVLHRSDGTRFACANIQYTESRQAPVTCSSASEPQVYWAQFVWPIAGTMLVRSYSTQAGGGGAQTPVATTVDAQLYDVTGMPSQFQGNLSWTGHEGEVGVTDTTNEAVARCFRAGNMLAGGNFSGRFRLPVTVGATPQQSRQVLSYSDATAVQFSSVRSIVLWVGPLPVACAAVKPVSRLSAIAALPMGNSVVYAQSSPLDQPARALQVAGTSTPGYVAVRNAPVNPYTSTTQSASNSACSSTGNLYNPSGATAPQNTAVGNGLVSTVSCDSSRTLFGNNSILGRSVTIETNASPSSAQQCGTLFLASQPSGARMLQARVSFPSYAPVTGDIYLTQWAYSGQTNLTATDYRLNGYSDTQLLINVKRASSTSNHNFHVHVNRIPPYPYNKSPCQELYAGGHYNPLEVNISTVSGYLSACSPGAELRCEVGDLSGKTGQLNVDGSPRFSTVAFLPLEGPYSIMGRSIVIHDPNSGAGRLACGNIVDARSTDAYVTFVTPVDPIDHPRFLQLAQAALNVNQSVLQYVHSYSDTATQCTLLSVNVLANDAAASKSLASRLRQVDLGAYTHQPKCDAFSDDASGLVHSLAIVLLCLCATLLL

>Tethya.wilhelma_78_CuZn Twilhelma_g2770.t1

MFKRAVLIISAVVYTAVIASAQVANSHVAVVRFNSRGIQGNITFTELGNGNVRIVANLQGLRGFHNWHIHHYPVDQSLDPAIQCRGVQIGGHYDPFGRSTDPNYDAQCAANQTLCEVGDLEGKFGPLPASGQVDVVDTTGQLALSGRYSVVGRPINIHDSVDLSVHLECGTIRLLEESEGAEVTLLQATFISPVAGTIYMRQVERQNVEIFGKLFWVNDSESTLQHNWHIHERQVST

>Tethya.wilhelma_79_CuZn Twilhelma_g4069.t1

MFKRAVLIISAVVYTAVIASAQVANSHIAVVRFNSRGIQGNITFTELGNGNVRIVANLQGLRGFHNWHIHHYPVDQSLDPAIQCRGVQIGGHYDPFGRSSDPNYDAQCAANQTLCEVGDLEGKFGPLPASGQVDVVDTTGQLALSGRYSIVGRPINIHDSVDSSVHLECGTIRLLEESEGAEVTLLQATFISPVAGTIYMRQVERQNVEIFGKLFWVNDSESTLQHNWHIHERQPGEEYRIGNCDITVGGNVGPHYDPTGNGPTSRAGYSTDCTPDTPQGCEVGDLTGKHSRIDVPARPDPYRNAAFFFTDSFLNLTDVTGRSLVVHVPGGGGPALACAPLVVSEEFTLTAAGNRVVQASQSTPFEDTSIISFGEVGSITTPLSTNDLAILSPGLNPVSTVCARENVYSPYDLYAGEGEDTPDRYDLGDWRSRYSGDFMAFSTFTVFDMPVYGSNSIAGRTMRAVVGSNPICSSWIPVLTRPNVIRARAAFTGSDFGPLEGAIYFFQHDLGNGVRGNTLIMVDVYYEGTSIDTSGHNWHVHENPPVAGQMCEGTIGEHYNPYPVSTGTLNAEEEEERSMFSDYDEDCSPTYPLRCESGDLAGKHERIRITSDGPSRPTYAYFDPDVQLAGDFSIIGRAIGIHVHNATGLLFDCGQIVPLQFSSNLLIIRINSGSMLRSEFARELTEGTSVDPTDVFIVTQTVTTLQPESGVQCTSFIMRINGETSEQANSNYDEVVNSGNLQKFGCIDPSSSGSVTFTDVPPRQLPFNRPYILRVVARMSDGSQAVIRRPVRVEQEGFCGVHFVNNGITVAGNIAIADFVSVGTVSSMLCSMDRGPLLPXAQFDVDYLERISSEGDSQASALARESLYVKFDPLVSKASPRKGGKGKSPRTRREEIASSDLMAMNTPPSKIESRLPTAVNVDTDTSNTPLSTGHEWDTTQEDDSLQYSSDDILMAPDEETLSLPESVDGGSMPEQQEELKYTEDEAVYMIKAAELEGQKSIREEVRTYNL

>Tethya.wilhelma_80_CuZn Twilhelma_g4069.t2

MFKRAVLIISAVVYTAVIASAQVANSHIAVVRFNSRGIQGNITFTELGNGNVRIVANLQGLRGFHNWHIHHYPVDQSLDPAIQCRGVQIGGHYDPFGRSSDPNYDAQCAANQTLCEVGDLEGKFGPLPASGQVDVVDTTGQLALSGRYSIVGRPINIHDSVDSSVHLECGTIRLLEESEGAEVTLLQATFISPVAGTIYMRQVERQNVEIFGKLFWVNDSESTLQHNWHIHERQPGEEYRIGNCDITVGGNVGPHYDPTGNGPTSRAGYSTDCTPDTPQGCEVGDLTGKHSRIDVPARPDPYRNAAFFFTDSFLNLTDVTGRSLVVHVPGGGGPALACAPLVVSEEFTLTAAGNRVVQASQSTPFEDTSIISFGEVGSITTPLSTNDLAILSPGLNPVSTVCARENVYSPYDLYAGEGEDTPDRYDLGDWRSRYSGDFMAFSTFTVFDMPVYGSNSIAGRTMRAVVGSNPICSSWIPVLTRPNVIRARAAFTGSDFGPLEGAIYFFQHDLGNGVRGNTLIMVDVYYEGTSIDTSGHNWHVHENPPVAGQMCEGTIGEHYNPYPVSTGTLNAEEEEERSMFSDYDEDCSPTYPLRCESGDLAGKHERIRITSDGPSRPTYAYFDPDVQLAGDFSIIGRAIGIHVHNATGLLFDCGQIVPLQFSSNLLIIRINSGSMLRSEFARELTEGTSVDPTDVFIVTQTVTTLQPESGVQCTSFIMRINGETSEQANSNYDEVVNSGNLQKFGCIDPSSSGSVTFTDVPPRQLPFNRPYILRVVARMSDGSQAVIRRPVRVEQEGFCGVHFVNNGITVAGNIAIADFVSVGTVSSMLCSMDRGPLLPCSSPQIFPGLSAGEHRLKVVPSQADCGRNRYRIHKKFTVEM

>Tethya.wilhelma_85_CuZn Twilhelma_g9299.t1

MSGLLCLYEDIYRLVWLAGVTVASVERSRVVGVASRTSMTAPWSIAQRSATARFNSRGIRGYITFTEEGPENVRIVANLDGLRGLHDWHIHHYPVDQSLEPTVQCSGRQVGGRYNPFNVNRSDPNYITNCQNDQSLCEVGDLENKFGPLPQSGQFNGVDTSTQLDLTGRYSIVGRPVVIHQSDTDPAHLECATIRFDDEMQGAEVTLLQATFISPVAGTIYMRQVERENVEIFGKLFWVNDSMSTTEHNWHIHERQPGEEYRMADCDITAGGNVGPHYDPTGNGPTSRANYTMECTPDTPQGCEVGDLTGKHARIDVQARPDPYRDAAFFFTDSFLNLADVTGRSIVVHVPGGGGPALACAPLVRSENITDVATVTAFIRASQSSPFEDTIIHTFTQLDGERRTLPSNNVTILTSTLGTQTLGCSGDVYTPYQPIREGGERTADSYPLGDWTNRYGDSLVPSTSSSFQVFDVPVYGPNTISGRTVRIDANSRRYCSFLSSKFSPDGDNIIRARAVFSGSEFGPIEGAIYFSQRDYGNGELGNTAVLVDIYYAASGLNTSGHSWHVHQTPPVMGQACVDAIGGHYNPFGVSLGLLDPAEGDPSINSDYSVDCSPQYPLRCESGDLSGKHGQLMISSEGETRQTFSYIDPNIQLTGSTSIINRSIGLHDTPGPLFDCGAVTPIYAAEELLNVFLTANDMLRTEFASKLTQGTGVDPVNVVVISEVVTPLQFRVECVNVTVAILGTTRQPELTLTAASPVNGRCSPSKSVGSSPTSARRQVHQQKERVDHHYLSVGHPVVKSGSLPDFARIENDLVPDDRYSCGVSMMMSPANKEGGTLLKRSKSLQRLVDQFFRHGTSSGSSNSQYRLHNATGARELLNELQKGADANAVNDQQDSPLHSIVRRQFSNKEDKQKLLLALLMHSEVNINQKGAHGVTALHLAVEGYDLATAETLIAFGANVNSKDDYNCTPLDLVDGVTSSSTIAVHKTTTNQGETQLGFGFESEHRNLVTLLQSVGALRGKYAHDVNPVLPFCDELGREEKPDEECDSAVFVPEQTETTKEAVAVHRGKPLPSSYREVEKYIEQRLLNVSLSLTEFDGEALSLALQMRELKMYKQAYGSRILFLDGGGVRGLVQIEILSQLEENTGRKITELFDWIVGTSTGGVIALAMVYGKKSLNEIRRLYLKMKDEVFGAKGVGFSYDTDALEKILKDELGADICMNSVTHPRVLIPAVRKEFINLKLHFFNNCFNNEYSTQPVWFVARCTSAAPVYFNEQSNYVDGGILANNPSASGLTEIQSYYRSRGEKLPISLVVSIGSGKNPQKKLGATDGFLGKKNVVKLLATALGESETIANNCECRCHEEGILYFRLNPRLEHIISAGETDNEKLIDMILQTRKELCLRQEFSNLVVSLHKLSQASRTIRSS

>Tethya.wilhelma_86_CuZn Twilhelma_g9299.t2

MSGLLCLYEDIYRLVWLAGVTVASVERSRVVGVASRTSMTAPWSIAQRSATARFNSRGIRGYITFTEEGPENVRIVANLDGLRGLHDWHIHHYPVDQSLEPTVQCSGRQVGGRYNPFNVNRSLHDWHIHHYPVDQSLEPTVQCSGRQVGGRYNPFNVNRSDPNYITNCQNDQSLCEVGDLENKFGPLPQSGQFNGVDTSTQLDLTGRYSIVGRPVVIHQSDTDPAHLECATIRFDDEMQGAEVTLLQATFISPVAGTIYMRQVERENVEIFGKLFWVNDSMSTTEHNWHIHERQPGEEYRMADCDITAGGNVGPHYDPTGNGPTSRANYTMECTPDTPQGCEVGDLTGKHARIDVQARPDPYRDAAFFFTDSFLNLADVTGRSIVVHVPGGGGPALACAPLVRSENITDVATVTAFIRASQSSPFEDTIIHTFTQLDGERRTLPSNNVTILTSTLGTQTLGCSGDVYTPYQPIREGGERTADSYPLGDWTNRYGDSLVPSTSSSFQVFDVPVYGPNTISGRTVRIDANSRRYCSFLSSKFSPDGDNIIRARAVFSGSEFGPIEGAIYFSQRDYGNGELGNTAVLVDIYYAASGLNTSGHSWHVHQTPPVMGQACVDAIGGHYNPFGVSLGLLDPAEGDPSINSDYSVDCSPQYPLRCESGDLSGKHGQLMISSEGETRQTFSYIDPNIQLTGSTSIINRSIGLHDTPGPLFDCGAVTPIYAAEELLNVFLTANDMLRTEFASKLTQGTGVDPVNVVVISEVVTPLQFRVECVNVTVAILGTTRQPELTLTAASPVNGRCSPSKSVGSSPTSARRQVHQQKERVDHHYLSVGHPVVKSGSLPDFARIENDLVPDDRYSCGVSMMMSPANKEGGTLLKRSKSLQRLVDQFFRHGTSSGSSNSQYRLHNATGARELLNELQKGADANAVNDQQDSPLHSIVRRQFSNKEDKQKLLLALLMHSEVNINQKGAHGVTALHLAVEGYDLATAETLIAFGANVNSKDDYNCTPLDLVDGVTSSSTIAVHKTTTNQGETQLGFGFESEHRNLVTLLQSVGALRGKYAHDVNPVLPFCDELGREEKPDEECDSAVFVPEQTETTKEAVAVHRGKPLPSSYREVEKYIEQRLLNVSLSLTEFDGEALSLALQMRELKMYKQAYGSRILFLDGGGVRGLVQIEILSQLEENTGRKITELFDWIVGTSTGGVIALAMVYGKKSLNEIRRLYLKMKDEVFGAKGVGFSYDTDALEKILKDELGADICMNSVTHPRVLIPAVRKEFINLKLHFFNNCFNNEYSTQPVWFVARCTSAAPVYFNEQSNYVDGGILANNPSASGLTEIQSYYRSRGEKLPISLVVSIGSGKNPQKKLGATDGFLGKKNVVKLLATALGESETIANNCECRCHEEGILYFRLNPRLEHIISAGETDNEKLIDMILQTRKELCLRQEFSNLVVSLHKLSQASRTIRSS

>Tethya.wilhelma_88_CuZn Twilhelma_g9300.t1

MEPYKKTLFEWPFLYPYQNSAFFFTDSYLNLTDVVNRTVVVHLPDGGGPGIACAPLVMSENFTLNAAGAEARFITASQTGPFEDTLINAYGIFGDDVRSLPTSDLTILGSAFGPTSTDCPREGIYSPYDNYTGEGLNNTPDRYDLGDWKSRYGNYLTPFNSFNVFDMPIYGANSMAGRTMRSVLNSTSRVCSSWTPVLAPQLGVLSARAVFSGTNFGLIEGVMYFFQTDYGNFNYGQTLVVLDIYYTGPGLNTSGHNWHVHQTPPVDGMSCVDSIGGHYNPFSVSLGTLTNQNSNYSDYASDCSPTYPLRCESGDLSGKHGKLTITSDGVNSYPTYAYLDPNLQLTGLYSIVGRSIGIHNFTGPLFDCAPVSRFVLSTNTLAVNVTAGQYSRNEFNRLITDGTDVSPTDVFIAGGQFVYPLEEEPEVECILFRLRILGDTSTEADANYDLVSTNVENMGIRCQATADAKALAVSFMLLGLSLLLALFSN

>Tethya.wilhelma_89_CuZn Twilhelma_g9300.t2

MEPYKKTLFEWPFLYPYQNSAFFFTDSYLNLTDVVNRTVVVHLPDGGGPGIACAPLVMSENFTLNAAGAEARFITASQTGPFEDTLINAYGIFGDDVRSLPTSDLTILGSAFGPTSTDCPREGIYSPYDNYTGEGLNNTPDRYDLGDWKSSVETARDXYGNYLTPFNSFNVFDMPIYGANSMAGRTMRSVLNSTSRVCSSWTPVLAPQLGVLSARAVFSGTNFGLIEGVMYFFQTDYGNFNYGQTLVVLDIYYTGPGLNTSGHNWHVHQTPPVDGMSCVDSIGGHYNPFSVSLGTLTNQNSNYSDYASDCSPTYPLRCESGDLSGKHGKLTITSDGVNSYPTYAYLDPNLQLTGLYSIVGRSIGIHNFTGPLFDCAPVSRFVLSTNTLAVNVTAGQYSRNEFNRLITDGTDVSPTDVFIAGGQFVYPLEEEPEVECILFRLRILGDTSTEADANYDLVSTNVENMGIRCQATADAKALAVSFMLLGLSLLLALFSN

>Tethya.wilhelma_87_CuZn Twilhelma_g9301.t1

MKLHTVALLLSFCCAALASHSARVVFNSRGIRGYITFTEQEDGNIRIVANLTGLRGGAALPELHLWHIHNFPVDQSLDPADQCAINQLGGFYDPFGALANQNYSADCFQNQNQSLCAAGDLASKFMPLPMDGQLDIVDTTGQLSLSGRYSIVGRSLVIHVPDATFAPLECGTIRLVEEVEGAQVRLMQATFISPIAGTIYMRQVEGQDVEIFGKLYWVDDTTSTSGHNWHIHTGQPEERYLEGYQAGCANVGSPYDPTQIGPGFVQNYAALCTPATPGRCRIGDMVGKHGSLSVTG

>Tethya.wilhelma_72_CuZn Twilhelma_g9578.t1

MRGCTCHNWKQPGLIFLSAVSLIILHLPASAHSQLTVSADFNQRGVKGSITFSQNDPQSSTTISVNLTGLDAVSGGPFQWHVHEYPYPNNGFTSGDICSAAVVGGHYDPLGANNVVNYSTVCNPSNRQACEVGDLSGKFGPLPVDMPTVTLSLDDQFLSLYGVYSIIGRSIVIHLNDNSRFICANIGYPDQDQIPDILLVPFRGDFTGSIYFRQHSTDYTASVYTDLFRVMNEENSIGHNWHVHQSPINSAGDDCNIAGPHYNPRGVNVSSESGYNEMCGPNNMTSQRNCEIGDLSNKGAPFDVESRVTKQYYTDTDLPLVGGDISIADRSVVIHEENRGGPRIACANITLYRPLEAIVTFDEDGISGSIQFFQLSPFDPTRVSVNLDGLDGMTDGYHVHVTPVGPVSAGSPARCLGEYTGGHWNPTNADYNAVPPITSDQYEVGDLSGKFGYLSGLNEISQVYFDQNVPLFGPYNIIGRSIVIHRPGGNRLICSNIEHVRRVVQVETIINTTVFSGRIRFIQPADDPNAETTIIVEFEGLVPAAVTPSVTIATSSMVTPSPSSLVMAPMTTSLAAASSSSVTLVQVQPTSSIAVGQASSSASGGLSPTPSPTPTDAGSGFVPTTTTIDNGPLMPFSTLPEMPTQTPMGNMKRRRRRSSTSGRSKRSMRARRQTTSGPILFDWTVRVSSVANPTDCSSLPLVNEVANILTQDLEPSSPILISLSLDLMLPSLTLRHRLRYCFFNLLQEVWVLLSANRLL

>Tethya.wilhelma_73_CuZn Twilhelma_g9578.t2

MRGCTCHNWKQPGLIFLSAVSLIILHLPASAHSQLTVSADFNQRGVKGSITFSQNDPQSSTTISVNLTGLDAVSGGPFQWHVHEYPYPNNGFTSGDICSAAVVGGHYDPLGANNVVNYSTVCNPSNRQACEVGDLSGKFGPLPVDMPTVTLSLDDQFLSLYGVYSIIGRSIVIHLNDNSRFICANIGYPDQDQIPDILLVPFRGDFTGSIYFRQHSTDYTASVYTDLFRVMNEENSIGHNWHVHQSPINSAGDDCNIAGPHYNPRGVNVSSESGYNEMCGPNNMTSQRNCEIGDLSNKGAPFDVESRVTKQYYTDTDLPLVGGDISIADRSVVIHEENRGGPRIACANITLYRPLEAIVTFDEDGISGSIQFFQLSPFDPTRVSVNLDGLDGMTDGYHVHVTPVGPVSAGSPARCLGEYTGGHWNPTNADYNAVPPITSDQYEVGDLSGKFGYLSGLNEISQVYFDQNVPLFGPYNIIGRSIVIHRPGGNRLICSNIEHVRRVVQVETIINTTVFSGRIRFIQPADDPNAETTIIVEFEGLVPAAVTPSVTIATSSMVTPSPSSLVMAPMTTSLAAASSSSVTLVQVQPTSSIAVGQASSSASGGLSPTPSPTPTDAGSGFVPTTTTIDNGPLMPFSTLPEMPTQTPMGNMKRRRRRSSTSGRSKRSMRARRQTTSGPILFDWTVRVSSVANPTDCSSLPLVNEVANM

>Tethya.wilhelma_20_CuZn Twilhelma_g10069.t1

MSAAAAVLIAVCVLVLVEGAMTNTAAVCVLEGAEVKGTISFTPVNREGSSAVRVTGQVTGLSPGNHGFHIHQFGDYSAGCVSAGPHFNPAGKEHGGPTDEERHVGDLGNIVADASGTATVAITDSQLTLSGPNSIIGRSVVVSDSTTEYTRLQFT

>Tethya.wilhelma_21_CuZn Twilhelma_g10069.t2

MLSFLSSTSTQGSSAVRVTGQVTGLSPGNHGFHIHQFGDYSAGCVSAGPHFNPAGKEHGGPTDEERHVGDLGNIVADASGTATVAITDSQLTLSGPNSIIGRSVVVSDSTTEYTRLQFT

>Tethya.wilhelma_22_CuZn Twilhelma_g10070.t1

MSTVGVCVLEGEVKGTIRFQPEGSAVKVTGEVTGLKPGKHGFHIHQFGDYSAGCVSAGSHFNPAGKEHGGPTDEERHVGDLGNIVADDSGKAVVAITDSQLTLSGPNTIIGRSVVSLDDSLMDSMYFGDEDPQLLTEQQQTAKENKVYTEQDEVLLFDVEANDFSFSDGSGGHESVKFGDGECEFIDPLTSPYDIPNGCQLEKRKRKRGVKDKLLSNRLIIDQEIGLNTHDMKLELNNIQCSTRELNFATLKLPKRVKAQELLFRPFAEKNEVPGVIYNTLNLKLCRQLIAEIRKSNHVSTSVDIQNTSDSPIIGRNWFGADRTHRDSELEYTGSNLEVELMRNGLPDEEMSYEDPLRNASAFSDHGRDPPPPTMGGLSPILDVEEEMMDLSGNFGLPELDDTYGSIPYDQSFSAVQTSPFIESEPHSYRRHVITVMGAREQLSFSELLPTNSNRKTASKAFSHILGDNVNRTARGQAGTRQRVRYTQDGCGLVEKATWSTLAVTHAVMDKYMYTALCALLWVQRVYLRSVQQSIAVTLTGKEGFTPLHVSLINNVHSNIEDIELCGSISIGTPPEKFTMAFLTDIPYILVDSLNCDCKENSHTRYNHMKSSSYEPNGKQFSITNNTHLTATGFLSNDKIIIADANITATFGEISHEKEITFTISGCDGVLGLGFTNSGHLPTVLAELVNDNIIPQPMFGIYFNHDSKSENGGELVLGGTNTHHYNPKDLLYANLTSGNSWEVRAHAVKVSDKSSLLCDGGCNVTLNPMDPFIVGNFENILALNKQLGAKSPAFGIFVFNCSTLQSLPVVTIALDGFELNLSGEDYVYKTVDPAACFSRFQGILNSSKKKLALGNSILRRYYAVFDAKEKRLGFARQQYI

>Tethya.wilhelma_23_CuZn Twilhelma_g10070.t2

MSTVGVCVLEGEVKGTIRFQPEGSAVKVTGEVTGLKPGKHGFHIHQFGDYSAGCVSAGSHFNPAGKEHGGPTDEERHVGDLGNIVADDSGKAVVAITDSQLTLSGPNTIIGRSVVSLDDSLMDSMYFGDEDPQLLTEQQQTAKENKVYTEQDEVLLFDVEANDFSFSDGSGGHESVKFGDGECEFIDPLTSPYDIPNGCQLEKRKRKRGVKDKLLSNRLIIDQEIGLNTHDMKLELNNIQCSTRELNFATLKLPKRVKAQELLFRPFAEKNEVPGVIYNTLNLKLCRQLIAEIRKSNHVSTSVDIQNTSDSPIIGRNWFGADRTHRDSELEYTGSNLEVELMRNGLPDEEMSYEDPLRNASAFSDHGRDPPPPTMGGLSPILDVEEEMMDLSGNFGLPELDDTYGSIPYDQSFSAVQTSPFIESEPHSYRRHVITVMGAREQLSFSELLPTNSNRKTASKAFSHILVLLSTSKVLEVEQIVSYGPIILKRGTDF

>Tethya.wilhelma_81_CuZn Twilhelma_g12188.t1

MKNLMTALQTLSLLSLLCTAHAGYKIATATFNAQGIEGYITFTDLGNGSIRINTNLDGLRGMHNWHLHHFPVDQSLSPDVQCGGQLIGGHYDPFGRNNDSNYDARCAADINMCEVGDLEGKFGPLPPDGQVDVIDTTGELTLRGRYSIVGRPINIHDHIDSSVHLVCSTVRLYNGSDATVTLLEATFLSPVAGTVYMRQVGSNNVEIFGKLFWVNDSESTQDHNWHIHQRQPGEEYRLGECGITEEGNVGPHYDPTGQGPTSRSGYSTDCTPDTPSGCEVGDLTGKHARINIPARPDPYRNGAFFFTDSALDLNEVVGRSLVIHIPNGSGGGPALACAPLMESHNFTLFSAGTGYLKASQASPYEDTLINGFSNMLGVPRGLPTDDLSVLTSSFGSQLTSCLKEDVYSPYDLYEGEGGMTPDRFQVGDWMNRYSDQLGPFASFNVFNMPIYGPNTISARTMRSVVGSSRLCSLWIPSLNPVNVIEAKASFSSSESGTINGAVYFIQDNINDGVEASRGHTIIIVDIYYPDMDMDTSDHDWFVYVNPSDDSSTCEDSIGDRYNPYPVSIGTLDASEDGDRSMFSDYSEDCSPTNPLSGNLVSVSEEALQATRATLDSGVPLGSGFPGLQTASGIPVNVSKDALEAAKAKLDDIAPEPGSSGFPGLLTASGRKVNISKESLEAARSLLGENTARDPQNEFQNLQTTSETEMDVCMEPFEAPQVTSNIPQGSTFPGLQTASGNKVVISKESLEAAKAVLNENTSTSANFPGLQTASGHKVHVSKESLDAARSVLDSDTSTLPSSTGRFPGLQSAGGNKVDISKKSLDAARRVLDTDTKITASGGFPGLQTAGGQNVHISKESLDAARRVLDSDAKTLSTASGGFPGLQTAGGTEVSISSESLEAARATLNRDTSSSFPGLQTAGGTKVHISRESLEAAKNVLGSIETTNKDWDCTKPVSHPPQPPHCTSQQTARNSDSTKRLDDGKYRPIFKPSPVSRGGFHVPTVTSREAATKELSRGESSVTPNDSSWLQPVRTTTRGVISTPEGEVFVFLVHTGTVCEGQLKTVIILWSVVGSSRLCSLWIPSLNPVNVIEAKASFSSSESGAINGAVYFIQDNINDGVEASRGHTTIIVDIYYPDMDMDTSDHDWFVYVNPSDDSSTCEDSIGDRYNPYPVSIGTLDASEDGDRSMFSDYSEDCSPTNPLRCASGDLSNKHGRLRITSNAPDRPTYSFLDPDLQLTGPFSIIGRSLGVHNSSGSLFDCATITPFHVSPFPIFVRFTTGEFLRTEFALELTEGTGVDPTDVLIVLQVVAPLKPNSDRECVNMRVRILGETDEFQVRFINKSPRVEGNDIIAEIETNRPVRSMHCALTGQPQQDXFALELTEGTGVDPTDVLIVLQVVAPLKPNSDRECVNVRVRILEETDKFQVRFINKSPRVEGNDIIAEIETNRPVRSMHCALTGQPQQDCSSGSVIFRDVSLCGRFNRPYILRVVAFSFEGVRTVIRRPIRIEQDEFCGVHFINDGVTVAGTTAFAEFASVGEMGSFMCSLDKQELQSCRSPQIFVNLTTGEHTLKIVPTIDDCGRDNRHRSIQTFTML

>Tethya.wilhelma_82_CuZn Twilhelma_g12188.t2

MTALQTLSLLSLLCTAHAGYKIATATFNAQGIEGYITFTDLGNGSIRINTNLDGLRGMHNWHLHHFPVDQSLSPDVQCGGQLIGGHYDPFGRNNDSNYDARCAADINMCEVGDLEGKFGPLPPDGQVDVIDTTGELTLRGRYSIVGRPINIHDHIDSSVHLVCSTVRLYNGSDATVTLLEATFLSPVAGTVYMRQVGSNNVEIFGKLFWVNDSESTQDHNWHIHQRQPGEEYRLGECGITEEGNVGPHYDPTGQGPTSRSGYSTDCTPDTPSGCEVGDLTGKHARINIPARPDPYRNGAFFFTDSALDLNEVVGRSLVIHIPNGSGGGPALACAPLMESHNFTLFSAGTGYLKASQASPYEDTLINGFSNMLGVPRGLPTDDLSVLTSSFGSQLTSCLKEDVYSPYDLYEGEGGMTPDRFQVGDWMNRYSDQLGPFASFNVFNMPIYGPNTISARTMRSVVGSSRLCSLWIPSLNPVNVIEAKASFSSSESGTINGAVYFIQDNINDGVEASRGHTIIIVDIYYPDMDMDTSDHDWFVYVNPSDDSSTCEDSIGDRYNPYPVSIGTLDASEDGDRSMFSDYSEDCSPTNPLSGNLVSVSEEALQATRATLDSGVPLGSGFPGLQTASGIPVNVSKDALEAAKAKLDDIAPEPGSSGFPGLLTASGRKVNISKESLEAARSLLGENTARDPQNEFQNLQTTSETEMDVCMEPFEAPQVTSNIPQGSTFPGLQTASGNKVVISKESLEAAKAVLNENTSTSANFPGLQTASGHKVHVSKESLDAARSVLDSDTSTLPSSTGRFPGLQSAGGNKVDISKKSLDAARRVLDTDTKITASGGFPGLQTAGGQNVHISKESLDAARRVLDSDAKTLSTASGGFPGLQTAGGTEVSISSESLEAARATLNRDTSSSFPGLQTAGGTKVHISRESLEAAKNVLGSIETTNKDWDCTKPVSHPPQPPHCTSQQTARNSDSTKRLDDGKYRPIFKPSPVSRGGFHVPTVTSREAATKELSRGESSVTPNDSSWLQPVRTTTRGVISTPEGEVFVFLVHTGTVCEGQLKTVIILWSVVGSSRLCSLWIPSLNPVNVIEAKASFSSSESGAINGAVYFIQDNINDGVEASRGHTTIIVDIYYPDMDMDTSDHDWFVYVNPSDDSSTCEDSIGDRYNPYPVSIGTLDASEDGDRSMFSDYSEDCSPTNPLRCASGDLSNKHGRLRITSNAPDRPTYSFLDPDLQLTGPFSIIGRSLGVHNSSGSLFDCATITPFHVSPFPIFVRFTTGEFLRTEFALELTEGTGVDPTDVLIVLQVVAPLKPNSDRECVNMRVRILGETDEFQVRFINKSPRVEGNDIIAEIETNRPVRSMHCALTGQPQQDXFALELTEGTGVDPTDVLIVLQVVAPLKPNSDRECVNVRVRILEETDKFQVRFINKSPRVEGNDIIAEIETNRPVRSMHCALTGQPQQDCSSGSVIFRDVSLCGRFNRPYILRVVAFSFEGVRTVIRRPIRIEQDEFCGVHFINDGVTVAGTTAFAEFASVGEMGSFMCSLDKQELQSCRSPQIFVNLTTGEHTLKIVPTIDDCGRDNRHRSIQTFTML

>Tethya.wilhelma_110_CuZn Twilhelma_g21126.t1

MKQVLLLVVALLATTLVHAFRTDTGPKAVDGIYNGRFGVEKNVQAEFDMGGVRGYVQFFQERPGDPVEINVNLQGLDQYSDPYPWRIHTYPSRFALLADYPCGEAEVGPVLDNHPGVEGGVGDLFAQHGGLRPDMPIQTFVNPHITLCGPESIVGRSLRIDRGDNEWICANIQYHGARVERLRASYEFSNTSSIQGDVIITKVIGRDDATIEVDIVRNQGVDMPLETSGHEWGIYRGPCSDLQGIYGAGDPMVNHHVIRDPYADCSPYNQRHCFIGDFTSKCGNLSFSNMGRSRAFCVDNQLGTVPCNYFYSEDDPEPVTSLVIRDVDANMTILGCSELGPPPPPQVAQCNSHFDRFTTAAQFYQASPNDKTYVKATVTGINGANVGLVILQGPTPADYDCSELGPILAKNSGVSFFPGPVEGGIKTSDRGVLGSFAGILDIPPGAKTFSASGDSDYLPLFGQCSIIGNSLVLVSDTGMRLGCCTIERTTSYSDREIASLLGYQNEENPPVGK

>Tethya.wilhelma_91_CuZn Twilhelma_g23165.t1

MANAKFIMLHLCAAVLILSTSLASQVASQEPENVATAVFGANGITGWIRFFEYEDDRVVIIVNMVGFASNASWSMHDLPVDLTLSPSEKCSEDYLGPVYDPMGMYNATLCEEDLIYCAGDLGASYGLIDDTPQLFNDSMTFITLSGPTSIVGRSLVVKNSEGVLCATIRSNSEKELQDDDIVTLKATFFSPLAGSIYFRQARDQPVTIFGKLYWVNDNEISFDHNWGIFGDRVAEDYREGNCSTVMTPYDPLSAGPASGEGYSNNCTSDFTDGCEIGDLSGKHSPIAIHSRPAPYSELAFFFTDSSLTLTNIEDLTLGIYSAEFEVIETVQYPDIYFTARQSSPYENTTVTFGSNLPMGGVHSILEPALMTATPCSADEMPYNPFDVPTNANFSTYKRTPDQYPLGDLSGKYDFLVSPSSILSVPELPLYGRNTIQGRTLRTVVKSQNDSEPTKRICSALVPVEEGVKYAKVSFSQNVFGSIYFTQNQNLAFSEQPGLASIVTNIYNMDVQQDFIWTMFDKSPDDDVNCSAATASPEITYNPFNATFDLEECSVQFPLRCPYGDFVAKHGKISVNTTAVYQDPHVQLDGRYTVIGKYMRLISFDPAPLVLECAKIEQVEFNTFDKDIVVGFPENEQSNDQQEFARYFDALVFHEEVGTYKTTDGTPCVVQHLKVTASTVKEANRFAVTVQNMSCSMYGDNACMQQLTVCDDEDGSTGTPMTPEGPAMTTEIVPDMTDRVPTDDIVSTTGTTATEKVTTTESPSTTAEETPMTTIKVSSTTEETPVTTESTSTITEETPMTTIKVSSATEETPVTTESTSTTTEETPMTTIKVSSATEETPVTTESTSTTTEETPMTTITVSSATEETVMTTKSTPSSEIKETSTESDSATTKMNGSKTIAPLSFTVSILLTGIIAGITM

>Tethya.wilhelma_92_CuZn Twilhelma_g23165.t2

MANAKFIMLHLCAAVLILSTSLASQVASQEPENVATAVFGANGITGWIRFFEYEDDRVVIIVNMVGFASNASWSMHDLPVDLTLSPSEKCSEDYLGPVYDPMGMYNATLCEEDLIYCAGDLGASYGLIDDTPQLFNDSMTFITLSGPTSIVGRSLVVKNSEGVLCATIRSNSEKELQDDDIVTLKATFFSPLAGSIYFRQARDQPVTIFGKLYWVNDNEISFDHNWGIFGDRVAEDYREGNCSTVMTPYDPLSAGPASGEGYSNNCTSDFTDGCEIGDLSGKHSPIAIHSRPAPYSELAFFFTDSSLTLTNIEDLTLGIYSAEFGSDIVACAPIVLSEVIETVQYPDIYFTARQSSPYENTTVTFGSNLPMGGVHSILEPALMTATPCSADEMPYNPFDVPTNANFSTYKRTPDQYPLGDLSGKYDFLVSPSSILSVPELPLYGRNTIQGRTLRTVVKSQNDSEPTKRICSALVPVEEGVKYAKVSFSQNVFGSIYFTQNQNLAFSEQPGLASIVTNIYNMDVQQDFIWTMFDKSPDDDVNCSAATASPEITYNPFNATFDLEECSVQFPLRCPYGDFVAKHGKISVNTTAVYQDPHVQLDGRYTVIGKYMRLISFDPAPLVLECAKIEQVEFNTFDKDIVVGFPENEQSNDQQEFARYFDALVFHEEVGTYKTTDGTPCVVQHLKVTASTVKEANRFAVTVQNMSCSMYGDNACMQQLTVCDDEDGSTGTPMTPEGPAMTTEIVPDMTDRVPTDDIVSTTGTTATEKVTTTESPSTTAEETPMTTIKVSSTTEETPVTTESTSTITEETPMTTIKVSSATEETPVTTESTSTTTEETPMTTIKVSSATEETPVTTESTSTTTEETPMTTITVSSATEETVMTTKSTPSSEIKETSTESDSATTKMNGSKTIAPLSFTVSILLTGIIAGITM

>Tethya.wilhelma_83_CuZn Twilhelma_g23977.t1

MHNWHIHHFPVDQSLSPDVQCGGQLIGGHYDPFGRNNDSNYDARCAADTNMCEVGDLEGKFGPLPHDRPVQFVDTTGELTLNGRYSIVGRPINIHDHIDSSVHLVCSTVRLYNRSDVTLLEATFVSPVAGTVYMRQVGSDDVEIFGKLFWVNDSESTQDHNWHIHEQQPGEEYRLGECGITEDGNVGPHYDPTGQGPTSRPGYSTDCTPDTPSGCEVGDLTGKHARINIPARPDPYRNGAFFFTDSALDLNEVVGRSLVIHVPNGGGPALACAPLMESHNFTLFAAGTGYLKASQASPYEDTLINGFSNMLGVPRGLPTDDLSVLASSFGSQLMSCLKEDVYTPYDLYEGEGETTPDRFQLGDWMNRYSDQLGPFASFNVFSMPIYGPNTISGRTMRSVVDSSRLCSLWIPSLNPVNVIEAKASFSSSESAAINGAVYFIQDNINDGVEASRGHAIIIVDIYYPDMDMDTSDYDWFVYVNPSDDSSTCEESIGGRYNPYPVSIGTLDASEDGDRSMHSDYSEDCSPTNPLRCASGDLSNKHGQLRITSNAPDRPTYSFLDPDLQLTGPFSIIGRSLGVHNSSGSLFDCATITPFHVSPFPIFVRFTTGDFLRTEFALELTEGTNVDPTEEKTTAGNRQTRNPNRVSYQLVLSLQSYNS

>Tethya.wilhelma_84_CuZn Twilhelma_g23977.t2

MHNWHIHHFPVDQSLSPDVQCGGQLIGGHYDPFGRNNDSNYDARCAADTNMCEVGDLEGKFGPLPHDRPVQFVDTTGELTLNGRYSIVGRPINIHDHIDSSVHLVCSTVRLYNRSDVTLLEATFVSPVAGTVYMRQVGSDDVEIFGKLFWVNDSESTQDHNWHIHEQQPGEEYRLGECGITEDGNVGPHYDPTGQGPTSRPGYSTDCTPDTPSGCEVGDLTGKHARINIPARPDPYRNGAFFFTDSALDLNEVVGRSLVIHVPNGGGPALACAPLMESHNFTLFAAGTGYLKASQASPYEDTLINGFSNMLGVPRGLPTDDLSVLASSFGSQLMSCLKEDVYTPYDLYEGEGETTPDRFQLGDWMNRSVVDSSRLCSLWIPSLNPVNVIEAKASFSSSESAAINGAVYFIQDNINDGVEASRGHAIIIVDIYYPDMDMDTSDYDWFVYVNPSDDSSTCEESIGGRYNPYPVSIGTLDASEDGDRSMHSDYSEDCSPTNPLRCASGDLSNKHGQLRITSNAPDRPTYSFLDPDLQLTGPFSIIGRSLGVHNSSGSLFDCATITPFHVSPFPIFVRFTTGDFLRTEFALELTEGTNVDPTEEKTTAGNRQTRNPNRVSYQLVLSLQSYNS

>Xenopus.tropicalis_47_CuZn NP_001106630.1

MNNLLYLAVALTVCELLSAGAEVVKPVEEELLTDTNKKVNELWINLLNMKPTDNDGIAYATCSLSPSSKLEPSEVKVTGLVLFKQVFPSGTLEAIFDLEGFPTDANQSARAIHIHTYGDLTNGCDSAGGHYNPMSVDHPQHPGDFGNFRVRDGKIQKFFANLDATLFGPFSVIGRSVVVHKQADDLGKGNNQASLENGNAGKRLACCIIGSSSKNNWEKYAQDSAAPRNLRFSRRVKNG

>Xenopus.tropicalis_48_CuZn XP_012827249.1

MNNLLYLAVALTVCELLSAGAEVVKPVEEELLTDTNKKVNELWINLLNMKPTDNDGIAYATCSLSPSSKLEPSEVKVTGLVLFKQVFPSGTLEAIFDLEGFPTDANQSARAIHIHTYGDLTNGCDSAGGHYNPMSVDHPQHPGDFGNFRVRDGKIQKFFANLDATLFGPFSVIGRSVVVHKQADDLGKGNNQASLENGNAGKRLACCIIGSSSKNNWEKYAQDSAAPRNLRFSRRVKNG

>Xenopus.tropicalis_67_CuZn XP_017953150.1

MLLLAGLGLLVLGSLCQGEFSAQIDMEGVRGYISFNISSQTITANLSGTCKNINISVHEFPVFYGASQDPCHTRNVGPSHYQLGLSNSSQESLKWPLGQSVVVEACGHRSCRNLHDTRGPLQIWHATFHSLVVGHIYFLKVMDEDPIVVLTELALPQGIPESPASLFFANSCEAQVNQSLGNFTVGNRTEALKSRKELPPTTIMPFASLEFQGKIICAKLKALRSKTASAHVSMNGVSGHFNFRQNSPLHPTEIDLNLRNLKGQFGEYSIHSLPVLARRDPGENLCNKNSTGDIWNPLGVNRSASSYPTQPGSAHHLWEMGDLSGCHGSLQGYEEMRTKLIDWNLPLYGNNSVVGRSVVLSKANGTEWVCSTIRQEGDMVVATASFHKGVVGRVMFRQSLEDVDNDLSILVELSSLSGQTSNGHNWHVHEFPLQMESDSCASAGGHFNPYNVSTGGNYKYECKYLNPLLCELGDYAGRHTPITLAGPHPTRYLFTDSYSSLTGPNSILGKALVIHGPDGAAPRLACANIIPQHPVEGKTGPWFGSGDAQGMLTASQISDLDPTAINISFYELRNRSGGFHIHVLPVISGSVAPCSDSLIKGHFNPFGVNVSTSPPAGNGTDDEYEVGDISGRRGLLVGKDSLVKQFIDMNFPLSGTHSILGRSLVIHYTNGSRMQCANVLPELKFGGDYIRAQAKFNGSISGSISMTQVIYPDGGFSDTIILVDFLSTPGNDNKSTNLQWFIPNEPNGTEFYNPYEIPAEGENMCRSQSPLHCKVGDLTSKHGVVSAQKRNLLTDINLPLASEFTVIGRFLMVKKDTLEPISAQILPDVPVFTVKLPAVTPFNRTAFRKAVSVALKVPSWKVILISGGHGRNETCYPVTFFIIGFNDTAALASLETPGSLGPFSTSSSCKSAVSQTSGAYRTSGFPGIGCFIVLFLLKILNC

>Xestospongia.bergquistia_25_CuZn Xb.00564.mrna

MMKAKGGGTIDRPRGIKFENMGLVNSKHHTLQAPHVTDYQPLSKVKLSGLCYITGEVTGLTPGKHGFHIHEFGDYSAGCVSAGGHFNPHRKNHGGPDDTDRHAGDLGNIVADDSGKATINITDKQIPLSGENNIIGRSVVVHADPDDLGKGGFPDSLTTGHAGGRLSCGVIGYAKS*

>Xestospongia.bergquistia_77_CuZn Xb.02636.mrna

MYRGLLLLSCALLVMISTSQGQTYGIANFNMRGIQGTIKFMPNDNDHVNITVDIQGTTMDHVWHVHNFPVDTSLPPQTRCHNDNIGGHFDPFGANAALGSLYSTYCGPNNQTVCEVGDLSGKVGNLTSGTFNYIDKSGDLTLFGRYGIIGRSIKIHGNDDVCATIYSSTEVMNRNIKVTTLQASFTYPVGGTIYMRQVAGEESVIFGKLFWVTNSQTTMNHKWHVHLNQPSMRYREGNCSDVGGHFNPYSVNISAPSYVYCGPTNLTECEVGDLSGKHGKITISDNPSPYNQYGFLYHDSFLNLTGMNTVMGRSIALHSNVGATPVMACAPLIEVETLTVTTLSEMFSASQSSRYSPTSVNTAYNAEDLTIFDSAIAPNQFCQATFSAGQARIYNPHSAPSLDGSDNTPDRYPVGQISTKYNFSSETQVSELPIHGIETIAGHSLGQRIQSSPSRTQFLCATLWPSYPGLENSNIKMAKATFNNTVDGAIYFIQRSFGRGVFGVTYILVDIWYTDNNRAISKGHKWHIHVNPPVTGQTCTNAIGGHFNPFNVNVSSSENYTTECSPNTPLRCESGDLSGKHPRLQISPGIANRTSYTYVDSNLHLWGTNDYSIIGRSVGLHLPASEGGALFDCGEIQEVYYASNSLNLTLSYDVNFDRTNFATRITEGVTDASPSDLLIVQQTMTVDSSSGTGNVNMTVRVLGNSTVNANTKAAAVQARFNELYSSAALILPSFFSITCFIVLAIFIGQS*

>Xestospongia.bergquistia_90_CuZn Xb.02637.mrna

MEKILQTIGLLLVLLTYVSAIDFSRTPLEATARINLKGVVGFVKFKTLNESAIQITTNFSGLPINGGSIRGLNWHVHRFPVDLTINPDHRCLNIYVGGHYDPLMARANENYTMNCSPGNPFACEIGDLTGKFGQLQNGYFVHVDNTGLLELGGLRGIVGRSIVVHASNGINFVCGTIRSNIEEQQNVEVITLSATFLSPLAGTIYLRQAADEHVVIFGKLFWVNGSPTTMRHNWHIHESAPSVNYRTGICDVGPHFDPFLRPSPNDSNYFCSPNRSNITNCEVGDLTGKHDTVNIAAAPLPFQEFAFFYTDIFLNLTGVRAVNERSIAIHASNRTAPIIACAPLVRTEERRVIQFPDGYFQASQSSPYEETKIRTSNSPSLNISVLSEVFNTYNLCPVNRPLYNPFPSNNVYPIATPDTQAVGSIFQKYPLELRGNLQPCVTELPVHGVNTITSRTIQIVTENRRICSAIPAYYNVNTTIVAVASFNSTIQGNIIFIQEKLSNISLGETNIIVDLHYSDSSHPSTSAHNWHIHQLPPSLDQPCEGTIGPHFNPFNVSLNSNNSISNYSNACSPSSPLRCESGDMSGKHGQLRIDPPGPNMITYSYLDPSLYLYGPDNQTIIGRSVGIHEAIPGGPVQFCAKVQRAYFTSVDITITEPTNYNRVDFVQTLVNMAQISAQSIVLQHVDVRTSNERSCTQMTLRTLGETETQSMEMASSVQSTLCSQYREDPCLTSRMDFDTLCSTNSGSTVRSLSISLALLILVAVSAWNFGMGHVLFELFKCFHRNISQLGNIQNFKTGGSLARTSNETSVILVSERLSFTIDIGDSDVHKILIASSLIL*

>Xestospongia.bergquistia_109_CuZn Xb.02836.mrna

METRVVVVVAALLLLAFWHVEATGKRRRYKNTYYKLPKGKTYHWKPADPWWVPDNRIPINVGAYFDMNHITGTIQFYQEREGKPVVIHIVLNGLDQFSEREMWGWHIHEYPINWALLESVSCGTPSVGGHYDPEKVGLASDYDQRCASDPKNCEVGDLSGRHGTLKPNQTTYTFTDSTLNLYGSYSLVGRSIVIHRTHGYRWACANIEYDGHNSLHTFRAQFPHCPKEQNPAFQGEIIMRWSGHRAGVTLESNLYRVDNECNPPDNETEYSWGLWGGKIGECGDCSGLTQMFGSVTYPPTNFPNCGQRSDDNTNHQRECAKGDFTGKCGPLVSDENCRIQGFCTDTQLGLYPYRYYKDLVVGIQDGDGNIVACTKFETVTPRAGIAFFYNYTRDHWIYSHLLFYQYDPHDPTYIRSYVVGLNAKAAYFRIRTHQASDRRDKTKCIGTGAVFEPRFGFQPLPIIRGLTQTGDRVYVGDLRYKLKDLRGEYVYRLQERNSYIPLFGDFNVIGRSLVLHDINGHVLGCANIEPYHPAFFTPDRSLEGYHKK*

>Xestospongia.bergquistia_74_CuZn Xb.15410.mrna

MQMPVVVIATVLLLIVQFSPTLGSLTAIATFSQNGISGTITFAQNSSSSPTSIHVNLTGLSTEELYQWHVHQYPFTAALPSPCSPANVGGHYDPLGASTNNNNYKADCAMNKTICEIGDLSGKFGLLNTSLLPLNINDSSLSLYGYQSIIGRSVVLHLQNGTRYVCANIDVSQPSNITEIEYVPFRALSIFGNIYFTEYNFGTTSVFVDLFSANPTVRHDWHVHNYSVQHGDTNGCSSTGGHYNPRNVDVKNYSLCNGANPLACEVGDLSGKGGQISFSGTQGRLLYTDTDLPITAAPNRISIIDRSVVIHQANGGAPRIACGNLTRLQSKMAAAIFNGEEGVSGTITFRQNSPYEPTTVIVNINGLQGMAGGYHVHETPVGEGNLSGREQCAPIYTGGHWNPRGVSYLNGIPPDNGTNDQYEVGDLSSKYGSLDGLPNINEEYSDSDLPLFGKDSIIGRSVVIHFAKNNSRWVCADINHSPPIVQTSSLFSIYGHTIQVLLVQPADDPFAETTIIIKNITSFVTPSVTSSSSSTLASSTSMLVSLSTSELLSPSPSASSILSSSFIQSPSDILSPSLSLSSMLSSSLVQSSSPSDTLSPSPSFSSMLSSSFVQVSLTSYTLSPSSSTYFTPLPFTTTLSPFSSLSLSPSSLMDDGSGDMKRRRRDTDEESIEDYLIPEVDYNYGWENDAPVIVERQITTETIKLSIRDVPQQPITYCELLSISLAAPSENYDLCQAGNQLACSGGDLLSKHGALSISATTGIRKVFTDSYLPLSGPDSVVNKVLLLETQDGSQTNQFAFIQANVPPVLDVSTCSISPTTSSIPTMSSSVTAPPISDTNLPLPLPYFIAVVAGGGVLLMLLILFLIICIACCVWKNRKSKSYKKYLMKDNLKDTHRNVELGLYKRSNPIFGVTTYKALFNYEPQQPDDLKLQEGDMVMLIEAPPGGEWWRGKVDNREGWFPKSYVNYIDIIAEEKKRKDESFAAAAATIRVASIAFQYDTAASTPTVSKKIDSTPLLVTKFDDPNRSEIETAFIVANVNTQTPDASPNVKRSTTPLLDDRALSPIDNNGSRTPTPPLHSPLPSDDIYIAQYNYYPQADSELQLQKGNEVIVIEKTDTGWWHGVVGEDHGWFPETFVKQTDKKAEEKMKDEENIELKKDEFRPRGMTEFHKGTSEEVEAMEYKAIFSYISEAEGDLQFYEGDSILVYWANPSGWWYGAAGGNQGYFPGSYVEPIMEVSEASSSVASPIPPDEDQSPLEEQAISLTNVPAIEGTPVHLGSSSLNDEIAAALKKRQNKESVKIEQEIRVEVPANEGGSISLNDEIAAALKKKQDKESIKTDSEIKTEVPYISSSPIAGDLQSDIMSVLNSFGSSKINDPSLLSDEHDDEIITATADQLSEENSIEKESKPVEVAKNSSSIKPRRHAPPPPSGRSSGRSSTSSRSPLPKSPSPLLSSMSPKPPEGLTAADIVVPNQLDDMTPRESPQSLESPKETPVENEVSKTETYEEATITSEQQPIPSTAEVAETKLTKESPEFPKKSQKENQASETGLTNQPNIKREEIRSGAPKVVKTLKTSPSTRPRRVPPPPPPSSLSTHTKTAITEDNNTPLKAPQTLTMPTSLTQKPNTSTKNNQTSLNHPPLTVTMPTNTSIKNNHTSTLSQGSESKSVKRPESRVAYGDTGSGGGWKPQVKKSRRPTAPRRLPPNPPPGSPQVIQKSQPKPKMTKIKRPSHRNIPPPPNIPLPPPPNIPLPPPPVKTKDIPPEPTFPPPSLNAIPAAAQQPSSVEPESEIPLPPDSEPGTITPPINSSTNISAKKTDKKDLKSQNDDKISALEHHNYGDDNKDTVITNSLMTFDTTEPKGTQPHPPKIEKQTQPLSLNPLDTMEPSNTQKVVETNKSEPQPPKMIATEEPITKEVQSHSPSTTNTPTTPNVQMEEIKPVDKPVRPVSQKSSIPNKPPPHVLAGANTKKKVPSTEKPLLKPKPKIKLNKIKQINPEKPEVGGVTVSELNPPLQNNVLQEETSQVKEPVPNQQSTQPDETSQVKEPVPKPRSAQPDETSQVKEPVPKPRSVQPDETSQVKEPVPKPRSAQPDETSQPVPNHGQLNQRDISSERTCSQTTVSSIR*

>Caenorhabditis.elegans_113_CuZnSOD

MIPADVPAYHIIDSFNCLFFLGLCEAEGSCRHCADSRYSYYRCRMQDDCFIGEICDNGFCCPNVLPTFSLQRELNPTIAMVCGANEQYSACFSSCQPSCQDPSTPACPAPGCQPGCICLPGYIRRDSSPRSACVPRGLCQAYDLTIRCADEKRQYQTCGSACPISCATRNQPRCNERCVTGCFCKIPFILENADDPLHSRCILPSSCPEIPTPAPEIVEQFVQRNPGMIGSFGNTNIQYPTSPRATPSPVFTTTTRGLTTSQQKLPTLIPEQHCEHPLKNYQSCGSKCPASCDRPLSQAASIDCALSCEPGCFCRLPYVLADSKDPNSTCILPQLCSRKSIPPSTASVPASQSCPDPRKEWSQCGALHCSRSCANPLGRCGSGQCFSGCVCRQPYVLLHPNDPTSRCVLPAECDRGCEDPTKEFMTCGSSCPMGCDNRHPKNCAPCQTGCFCKNGLVFENSATWHTSKCIKIEECPPEEETTTEESTTTTTTEASVPPATTVIAEVHETRGRPFSSDSDLLALKPTVSQSECPATTFDVGGRGCNTDMDCPMEQRCCRPMIVSLGVNPQRCVCPDKHAVWSSCGTLCPEYCGQPSVPVCSGTCSAGCHCAPGFVRARNDVTAPCVPRESCSSSLGSSTTSRMRISPVAASMDEPMRFATIREITPHDAFVDDTIAVAILITREGAHIGRFTFSQLTSTALRIHGEVYTLPVGRHAVVLHQFGDSSEGCSRVGAPFSKSLSPSLGDITETGKFDRIVEWPVIDVVGRAVVIYSFSTAEWSLRAHFGEKPLACGTIGIAKVRAR

>Caenorhabditis.elegans_114_CuZnSOD

MLRILLLIVIFSSSWKEVHGQLRLCEAEGSCRHCADSRYSYYRCRMQDDCFIGEICDNGFCCPNVLPTFSLQREPTSPKPKRPLNITSDPPADEPCPDESRWSKRCSRDEDCTHQEELCIEGKCCKTCAMRRRELLDELRSLEIVGQAIPLCEESGLAWRRRQCLAGTEVCWCVTRYGRRLSGKESKDEKGCETVRRKQENLALKAAALLREQKEREETCKSTSPGECPLPLNTSITTLTRCFCDSECERGSKCCPGSITNSNYHACLPALTKIMNEPISNEINPTIAMVCGANEQYSACFSSCQPSCQDPSTPACPAPGCQPGCICLPGYIRRDSSPRSACVPRGLCQAYDLTIRCADEKRQYQTCGSACPISCATRNQPRCNERCVTGCFCKIPFILENADDPLHSRCILPSSCPEIPTPAPEIVEQFVQRNPGMIGSFGNTNIQYPTSPRATPSPVFTTTTRGLTTSQQKLPTLIPEQHCEHPLKNYQSCGSKCPASCDRPLSQAASIDCALSCEPGCFCRLPYVLADSKDPNSTCILPQLCSRKSIPPSTASVPASQSCPDPRKEWSQCGALHCSRSCANPLGRCGSGQCFSGCVCRQPYVLLHPNDPTSRCVLPAECDRGCEDPTKEFMTCGSSCPMGCDNRHPKNCAPCQTGCFCKNGLVFENSATWHTSKCIKIEECPPEEETTTEESTTTTTTEASVPPATTVIAEVHETRGRPFSSDSDLLALKPTVSQSECPATTFDVGGRGCNTDMDCPMEQRCCRPMIVSLGVNPQRCVCPDKHAVWSSCGTLCPEYCGQPSVPVCSGTCSAGCHCAPGFVRARNDVTAPCVPRESCSSSLGSSTTSRMRISPVAASMDEPMRFATIREITPHDAFVDDTIAVAILITREGAHIGRFTFSQLTSTALRIHGEVYTLPVGRHAVVLHQFGDSSEGCSRVGAPFSKSLSPSLGDITETGKFDRIVEWPVIDVVGRAVVIYSFSTAEWSLRAHFGEKPLACGTIGIAKVRAR

>Caenorhabditis.elegans_115_CuZnSOD

MLRILLLIVIFSSSWKEVHGQLRLCEAEGSCRHCADSRYSYYRCRMQDDCFIGEICDNGFCCPNVLPTFSLQREPTSPKPKRPLNITSDPPADEPCPDESRWSKRCSRDEDCTHQEELCIEGKCCKTCAMRRRELLDELRSLEIVGQAIPLCEESGLAWRRRQCLAGTEVCWCVTRYGRRLSGKESKDEKGCETVRRKQENLALKAAALLREQKEREETCKSTSPGECPLPLNTSITTLTRCFCDSECERGSKCCPGSITNSNYHACLPALTKIMNEPISNEINPTIAMVCGANEQYSACFSSCQPSCQDPSTPACPAPGCQPGCICLPGYIRRDSSPRSACVPRGLCQAYDLTIRCADEKRQYQTCGSACPISCATRNQPRCNERCVTGCFCKIPFILENADDPLHSRCILPSSCPEIPTPAPEIVEQFVQRNPGMIGSFGNTNIQYPTSPRATPSPVFTTTTRGLTTSQQKLPTLIPEQHCEHPLKNYQSCGSKCPASCDRPLSQAASIDCALSCEPGCFCRLPYVLADSKDPNSTCILPQLCSRKSIPPSTASVPASQSCPDPRKEWSQCGALHCSRSCANPLGRCGSGQCFSGCVCRQPYVLLHPNDPTSRCVLPAECDRGCEDPTKEFMTCGSSCPMGCDNRHPKNCAPCQTGCFCKNGLVFENSATWHTSKCIKIEECPPEEETTTEESTTTTTTEASVPPATTVIAEVHETRGRPFSSDSDLLALKPTVSQSECPATTFDVGGRGCNTDMDCPMEQRCCRPMIVSLGVNPQRCVCPDKHAVWSSCGTLCPEYCGQPSVPVCSGTCSAGCHCAPGFVRARNDVTAPCVPRESCSSSLGSSTTSRMRISPVAASMDEPMRFATIREITPHDAFVDDTIAVAILITREGAHIGRFTFSQLTSTALRIHGEVYTLPVGRHAVVLHQFGDSSEGCSRVGAPFSKSLSPSLGDITETGKFDRIVEWPVIDVVGRAVVIYSFSTAEWSLRAHFGEKPLACGTIGIAKVRAR

>Mnemiopsis.leidyi_115_CuZnSOD

MRRFEFLIATSCEAPSIESLNELLLGKGNIKSYDHLNGLVIFETLQTASTVESILKKRFSKVKLVGQSSESLIQAAGVAIIGGNKAKGIVRLLEDENGLNIEGTVSGLEKGSYSVSINEYGDISNACLSTGNPVSKASEETPCGILGQVESDGSIDTKFSLNSGRLSIMECIGRSLVLQNETKKIACGIVGRSPGVSENNKKICACDGTVIWEEQAFGPYAVEPPSET

>Dictyostelium.discoideum_1_SODCuZn sp|Q55GQ5|SODC1_DICDI Superoxide dismutase [Cu-Zn] 1 OS=Dictyostelium discoideum OX=44689 GN=sodA PE=2 SV=1

MSKTAVCVIKGEKVNGVVKFTQENKDSPVTVNYDITGLEKGEHGFHVHAFGDTTNGCVSAGPHFNPFGKNHGAPSDEDRHVGDLGNIVADGESNTKGTISDKIISLFGEHTIVGRTMVVHADQDDLGKGGKPDSLTTGAAGARLGCGVIGVSQ

>Dictyostelium.discoideum_2_SODCuZn sp|Q54TU5|SODC4_DICDI Superoxide dismutase [Cu-Zn] 4 OS=Dictyostelium discoideum OX=44689 GN=sodD PE=2 SV=1

MVKAICVVKGAVVNGTIIFSQENEGSPVYVNGTISGLSGGLHGFHIHEFGDTSNGCLSAGAHFNPFHVEHGGPNSAIRHVGDLGNITSCPSSKVANVLIQDNVISLFGDLSIIGRTLVVHENQDDLGLGGNLSKTTGNAGARVACGILAKI

>Dictyostelium.discoideum_3_SODCuZn sp|Q54G70|SODC5_DICDI Superoxide dismutase [Cu-Zn] 5 OS=Dictyostelium discoideum OX=44689 GN=sodE PE=3 SV=1

MSAICVIKGDGVDGIINFKQNDNKSPVIISGVISGLKEGKHGFHVHEFGDTTNGCLSAGAHFNPFKKEHGSPNDENRHVGDLGNIESNKDKKSIINITDNIITLFGQNSIIGRSIVVHDKEDDLGRGNSQDSKITGNAGSRLGCGIIALSKI

>Dictyostelium.discoideum_4_SODCuZn sp|Q54TW8|SODC6_DICDI Probable superoxide dismutase [Cu-Zn] 6 OS=Dictyostelium discoideum OX=44689 GN=sodF PE=3 SV=1

MVNAIVIIKGLGVEGKVTLSQECEGSPIYINGTVSGLTPGQHGMHVHEFGDTSNGCISAGDHYNPLHREHGSPLDVERHIGDLGNIKALSNGVATISIRDTIMSLFGDISVMGRTMVIHSDRDDYGRGNFPDSKTAGHSGKRVGCGIIAKI

>Trichoplax.adhaerens_SODCuZn SOD1 tr|B3S9R7|B3S9R7_TRIAD Superoxide dismutase [Cu-Zn] OS=Trichoplax adhaerens OX=10228 GN=TRIADDRAFT_38351 PE=3 SV=1

MALKAVCCLQGPVVSGTIFFQQESGTGPIRISGEVKGLAPGKHGFHVHEFGDNTQGCTSAGGHYNPHKKVHGAPGDEIRHVGDLGNIEANEQGVASINMTDRMVTLTGPYSCIGRTIVVHEGVDDLGKGGHELSLTTGNAGARVACGVIGITKC

>Arabidopsis.thaliana_1_SODCuZn SOD1 sp|P24704|SODC1_ARATH Superoxide dismutase [Cu-Zn] 1 OS=Arabidopsis thaliana OX=3702 GN=CSD1 PE=1 SV=2

MAKGVAVLNSSEGVTGTIFFTQEGDGVTTVSGTVSGLKPGLHGFHVHALGDTTNGCMSTGPHFNPDGKTHGAPEDANRHAGDLGNITVGDDGTATFTITDCQIPLTGPNSIVGRAVVVHADPDDLGKGGHELSLATGNAGGRVACGIIGLQG

>Arabidopsis.thaliana_2_SODCuZn SOD1 sp|O78310|SODC2_ARATH Superoxide dismutase [Cu-Zn] 2, chloroplastic OS=Arabidopsis thaliana OX=3702 GN=CSD2 PE=1 SV=2

MAATNTILAFSSPSRLLIPPSSNPSTLRSSFRGVSLNNNNLHRLQSVSFAVKAPSKALTVVSAAKKAVAVLKGTSDVEGVVTLTQDDSGPTTVNVRITGLTPGPHGFHLHEFGDTTNGCISTGPHFNPNNMTHGAPEDECRHAGDLGNINANADGVAETTIVDNQIPLTGPNSVVGRAFVVHELKDDLGKGGHELSLTTGNAGGRLACGVIGLTPL

>Podospora.anserina_1_SODCuZn SOD1 sp|Q711T9|SODC_PODAS Superoxide dismutase [Cu-Zn] OS=Podospora anserina OX=2587412 GN=SOD1 PE=3 SV=3

MVKAVAVVRGDSKVSGSVVFEQETENGPTTITWDITGHDANAKRGMHIHTFGDNTNGCTSAGPHFNPHGKTHGNRTDENRHVGDLGNIETDAQGNSKGTVTDNLIKLIGPESVIGRTVVVHAGTDDLGKGDTEESLKTGNAGARPACGVIGISA

>Podospora.anserina_2_SODCuZn SOD1 tr|B2ATY8|B2ATY8_PODAN Superoxide dismutase [Cu-Zn] OS=Podospora anserina (strain S / ATCC MYA-4624 / DSM 980 / FGSC 10383) OX=515849 GN=PODANS_1_17400 PE=3 SV=1

MVKAVAVVRGDSKVSGSVVFEQETENGPTTITWDITGHDANAKRGMHIHTFGDNTNGCTSAGPHFNPHGKTHGNRTDENRHVGDLGNIETDAQGNSKGTVTDNLIKLIGPESVIGRTVVVHAGTDDLGKGDTEESLKTGNAGARPACGVIGISA

>C.crispus_CuZnSOD_1 tr|R7QQ00|R7QQ00_CHOCR Potential copper-zinc superoxide dismutase, CuZn SOD1 OS=Chondrus crispus OX=2769 GN=CHC_T00008473001 PE=4 SV=1

MTPLLVAALLAAVSLAAAAEVPLFTQEGCSNLPTLVCTVQPTDGYSVEGVVYFTPAWRRRGVGEQPDLFTCYVRIMAAVAGLTNPQHGFHVHTYGDVSVSDGSSTGGHFTNVAGDEIEHGLPDDEIRHWGDLGNLINDGKGNAEYDRVDKVVRLGALVGRGITIHEDQDAGSSEQPTGASGTRIGFCVIGYANPEVIASASR

>C.crispus_CuZnSOD_2 tr|R7QRY5|R7QRY5_CHOCR Potential copper-zinc superoxide dismutase, CuZn SOD2 OS=Chondrus crispus OX=2769 GN=CHC_T00010182001 PE=4 SV=1

MASFLTPLALLTLVLAVSCMPAPTSVPTAGNSTAAMAEKRKRCLNSLPLVCNVVATKGYNCTGYVKFDPVFIMNRRGMPSCHARVRANLKGLSPGRHGFHIHTYGDIRGLDGSSTGGHFTNVKGDDLPHGYASSPARHMGDLNNVMARANGEAKYSREDNVIRLGAIRGRGITIHQDRDMGPGSQPSGAAGDRVGTCVIGVVDAKIEVFGEEGDVTVVNFLVNEKPATPTSSLQKFLTHLLPLSVHMFLCSRYPWCSQGSAVNAAHSADLSRREALEDSPVSFRIQKSRREIVTK

>P.nodorum_CuZnSOD_1 tr|Q0UEY3|Q0UEY3_PHANO Superoxide dismutase [Cu-Zn] OS=Phaeosphaeria nodorum (strain SN15 / ATCC MYA-4574 / FGSC 10173) OX=321614 GN=JI435_096810 PE=3 SV=1

MVKAVAVLRGDSNVKGTVTFEQENESSPTKISWDITGNDANAERGMHVHAFGDNTNGCTSAGPHFNPHNKTHGAPEDEERHVGDLGNFKTDGQGNAQGSVSDKLIKLIGSESVIGRTIVVHGGTDDLGRGGHEESKKTGNAGPRPACGVIGISN

>P.nodorum_CuZnSOD_2 tr|Q0UPE0|Q0UPE0_PHANO Superoxide dismutase OS=Phaeosphaeria nodorum (strain SN15 / ATCC MYA-4574 / FGSC 10173) OX=321614 GN=JI435_063740 PE=3 SV=1

MNTTLLRTSALRSAARATAPAVSRAGLAGTTFVRGKATLPDLSYDYGALEPAISGKIMELHHKNHHNTYVTSFNNFSEQIAEAKQKQDIAAQIALQPLINFHGGGHLNHTLFWENLAPTSQGGGEPPTGALSKAINDSYGSLDAFKEKFNTALAGIQGSGWAWLVQDTQTGSVQIRTYANQDPVVGQFRPILGVDAWEHAYYLQYQNRKAEYFKAIWDVINWKAAEKRFK

>P.nodorum_CuZnSOD_3 tr|Q0UD72|Q0UD72_PHANO Sod_Cu domain-containing protein OS=Phaeosphaeria nodorum (strain SN15 / ATCC MYA-4574 / FGSC 10173) OX=321614 GN=JI435_102920 PE=4 SV=1

MHSSMLLGLLPLAAAQVAQVHEAPPPPPVLLNATKSGVAPTLPTPFNGTQTLQGAIIMPLPPQPGYIGLNGTATAQTSMPASTYRATMPPSMFNDLVGTTVSGYIEGVGSPSGVRFTVKLQNLPEQAKYGPFNWHIHALPVPADGNCTATLGHLDPTNRGELIMCDAAAPETCQAGDLAGKHGGKIMAAGSFETAFVEKYLTTETGSPFSFAGLGFVLHSMNTTRLTCANFVQVNGTVGGNGTMPSATGPPAGEFTGGAGKVGAALGALGVAVVAALL

>P.nodorum_CuZnSOD_4 tr|Q0UMZ2|Q0UMZ2_PHANO Sod_Cu domain-containing protein OS=Phaeosphaeria nodorum (strain SN15 / ATCC MYA-4574 / FGSC 10173) OX=321614 GN=SNOG_06872 PE=4 SV=1

MRTSVFTLLAAASAVSAQTGPAPQVAGNPAGASYVATLPVKEGSPLRGAITAVSAPDGVGVLFSVSFSGLPATGGPFMYHLHEKPVPENGNCTATGAHLDPYKRGEVPICDASKPETCQTGDLSGKHGNFTSGEFSAEYVDPYSALIPGNNAYFGNLSFVLHLANKTRIGCANFVSQGAKPSSGASLPAPSATGSYNTTVMPTGGSPVTPTSAPPSEFTGAAVKAASAAGAVLVAAAAFVL

>Fonticula.alba_CuZnSOD_1 tr|A0A058ZE05|A0A058ZE05_FONAL Superoxide dismutase [Cu-Zn] OS=Fonticula alba OX=691883 GN=H696_01116 PE=3 SV=1

MTKAVAVLNGLNGLKLVATFSQQSPDSPTVIDIEGSGIKPGLHGFHVHEFGDTTNGCVSAGPHFNPHGHTHGAPSAAIRHVGDLGNLSVDETGSVKVTITDHLVSLSGVNNVVGRALVLHADVDDLGLGGHELSATTGNAGDRIACGVIGITK

>Pseudanabaena.ABRG5-3_CuZnSOD tr|A0A2Z5WSM0|A0A2Z5WSM0_9CYAN Superoxide_dismutase_copper/ zinc_binding_protein OS=Pseudanabaena sp. ABRG5-3 OX=685565 GN=sodC PE=3 SV=1

MNQNNFQSRFSSKFVVLLSCATVAAIATSTTAIAQTTQSRASSRIFNVKGDLVGTATFIQTPLGVKVDLKVQNLAQGEHMVHLHEQGKCDAPDFKTSGNHFDPEEKDDDDHMQMHHKHEHGKHKPAGDLPNIIVKQDGNGNLTALLPKLTLGSGKNSLLKQGGTSILIHAGANGKSTIPNVDFKTRIACGVIKSE

>Pseudanabaena.PCC7367_CuZnSOD tr|K9SG01|K9SG01_9CYAN Superoxide dismutase copper/zinc binding protein OS=Pseudanabaena sp. PCC 7367 OX=82654 GN=Pse7367_0398 PE=3 SV=1

MQIFNSIKTKHFTIAATSLLIAIGSSTIAPAHADQHSGHKHDQNQTEHSHSNHSAHSDKGAMAMIKDIEGQLVGTASFRDTEAGLEVIVEAQNLAPGEHAIHIHEVGKCEAPDFKSAGGHFNPSNPSHSSSHANHASDKHGAMHHNSHSSNALDAHDHGAAGDLPNLNISRDGTGVLTAVLPDLALGDSSNSILHPAGTAVLIHAGAAGVTTIPGVDADTRVACGVVSAAE

>Pseudanabaena.PCC7429_CuZnSOD tr|L8N810|L8N810_9CYAN Superoxide dismutase copper/zinc binding OS=Pseudanabaena biceps PCC 7429 OX=927668 GN=Pse7429DRAFT_0087 PE=3 SV=1

MTNKTNKMIDKNYLLALFRHKFRHKFRHKFNLVNFGLACMTITAATAVTSATVIAQNATSVIIRNPQSAIASIFNIKGEQVGTATFTQTPLGLQVIVNVENLTQGEHMIHIHENGKCDAPDFKTSGNHFNPKNPKDDDDDNEINPMHGNHGAHENHEKHEGEKHNPAGDLPNIVVKKDGKGKLNALLPRLSLDNKPNSLLKQGGTSILIHAGANGNSTIPNVDYKTRIACGVIKKF

>Pseudanabaena.SR411_CuZnSODtr|A0A256BEG9|A0A256BEG9_9CYAN Sod_Cu domain-containing protein OS=Pseudanabaena sp. SR411 OX=1980935 GN=B9G53_12540 PE=3 SV=1

MRGLTWRESQFSDVGAKEHMINSNKYQFQAKFASRLAVLLPLITMATLTDINAAISKTLKSSASSQIFNAKGELVGTATFTQSTSGVKVTLQVQKLAQGEHMVHLHENGKCEAPDFKSAGNHFDPKRSPMDATHDHMHHKHEEMKHGVDQHQPAGDLPNIMVQQDGTGTLTATLSELTLGTGHNSLLKKGGTAIVIHAGANGKSTIPNVDYKTRIACGVVKSQ

>Gloeobacter.kilaueensis.JS1.SODC_CuZnSOD tr|U5QI46|U5QI46_9CYAN Superoxide dismutase OS=Gloeobacter kilaueensis JS1 OX=1183438 GN=sodC PE=3 SV=1

MKKSILSGCLLLLIPVFAASAQTGTRQATAALVDSNGQTVGTAALSQSSAGVLSVKLDVKGLPPGEHGIHFHAVGLCEGPGFTSAGGHFNPATRQHGLENPKGAHAGDLPNLSVAADGTASYSATDVRASLEPNAFNNLLDSDGTALIIHARPDDLKSDPVGNSGARIACGVLKPAK

>Gloeobacter.kilaueensis.JS1.GKIL_3379_CuZnSOD tr|U5QPK5|U5QPK5_9CYAN Superoxide dismutase OS=Gloeobacter kilaueensis JS1 OX=1183438 GN=GKIL_3379 PE=3 SV=1

MKKIFLSPALIALLLAAPVLAGNPPQATAQIKDAKGEPVGTASFVQQPKGVLVTVEVKGLEPGKHPMHIHAVGKCDAPDFRSAGPHLGDHKMAGMKGSMAMETMAGDLPELLVGADGTGKAEVLNPEISLGSKSLLQKQGTALLIHDATKPSKRIACGVISTNPNP

>Gloeobacter.violaceus.PCC7421.glr2170_CuZnSOD tr|Q7NIL3|Q7NIL3_GLOVI Glr2170 protein OS=Gloeobacter violaceus (strain ATCC 29082 / PCC 7421) OX=251221 GN=glr2170 PE=4 SV=1

MQKMVHNALVDRPGTRWQGFAVFAGIFGLLCLLLMAVPAIDADRWLQAKIELKNADGLPVGNASLTQLSDGVRVSVQVQGLLPGKYPIHFHSKGKCVAPDFRSSRGVFDTHSLGHKTRPDGQPVPPAGLLPALIVGAAGTGELNALNTDVTLRAHKLHSLLRPGGSALVIHAAHSRQIIACGAVTRTPVSD

>Gloeobacter.violaceus.PCC7421.glr1981_CuZnSOD tr|Q7NJ51|Q7NJ51_GLOVI Glr1981 protein OS=Gloeobacter violaceus (strain ATCC 29082 / PCC 7421) OX=251221 GN=glr1981 PE=3 SV=1

MVVCRSKVSGFSLESSGCNGSEEASMSKHLFALGAVGWLLAAPALAGEQPAAVSAIKDLNGQVVGTATFRQQPEGVLVNIQVQGLQPGKHAMHIHAVGKCEPPAFKSAGPHLGDAKMAPTSGTPHSHGAKGATALSMNHAHSAGSMASSIPHNHGAMHAAMAGDLPDLVVGSDGSGKAEILNAEVRLTPGRNFLLQSEGTALLIHDVADPNQRIACGVISKAPNSSPEAKVNP

**MnSOD family**

>Acanthaster.planci_1_Mn gbr.180.4.t1

MLSRAVVLRRLLASPGTLSTVNSRLEHTLPPLPYDYGALSPVINADIMELHHKKHHATYVNNLNLAEKQLQEAQEAGDIGKMIALQPAVKFNGGGHINHSIFWTVMSPNGGGEPSGELLSAINRDFGSFDNMKQKLSAASIGVQGSGWGWLGYNKTTRTLAIATCANQDPLEPTTGLVPLFGIDVWEHAYYLQYKNVRPDYVKAIFNVANWANIEERFQKAMADS*

>Amphimedon.queenslandica_20_Mn Aqu2.1.22475_001 Aqu3.1.22475_001

MLSATRSFLLFGGKNSLSSLSSISRRHKHVLPELPYGYKALEPVISGDIMELHHTKHHATYVNNLNATEGKMKECLEAGDVSGAVALEGAYRFNGGGHINHSIFWNNLSPNGGGTPQGKLMEAIERDFGSFDEFKSQLTARTVAIQGSGWGWLGFNQVTGRLQIATCPNQDPLQATTGLVPLLGIDVWEHAYYLQYRNVRPDYVKAIWDVINWDDVSKRLP*

>Amphimedon.queenslandica_33_Mn Aqu2.1.21481_001 Aqu3.1.21481_001

MEFIRIFSLFSFFTLTLSQSSIYDEMFYPVDEYSFPQLPGYDYHELEPYIDQRTLTVHHKKHHQGYTVKMNQALKDWRKQEPQSDLAKSSIIDILQNLEMVPDKWRTTLQNNAGGYVNHIYYWVTMCPKPGEISKALLKKIKASFPAGMSEFKESFTTASLSLFGSGYVWLVTDDEGSISIISTKNQDCPISSNLYPLLVLDVWEHSYYLKHQNLRADYISDWWNVVCWQNVETLRQFWINRQIKDEL*

>Amphimedon.queenslandica_34_Mn Aqu2.1.06765_001 Aqu3.1.06765_001

MFHPADEYSFPQLPGYDYHELGPYIDQRTLTVHHKKHHQGYTVKMNQALKDWRKQEPQNDLARSSIINILQNLERVPDKWRTTLQNNAGGYVNHIYYWVTMCPKPGDIPKALLKKIEASFPAGMSEFKNSFTTASLSLFGSGYVWLVTDDEGSISIISTKNQVLYCHAFILIMLYMYHYKRFLRELYFTRCTKFLIWRF*

>Branchiostoma.floridae_2_Mn XP_002604137.1

MLACVKLTQRVASHPGLLAAAGTRLKHTLPDLAYDYGALEPTISAEIMQLHHSKHHATYVNNLNVAEEKLAEAQAKGDVTTEIALGPALKFNGGGHLNHSIFWTNLSPNGGGEPQGEVLEAINRDFGSFENLKKKMSAASVAVQGSGWGWLGYDKENNRLSIAACANQDPLQATTGLIPLLGIDVWEHAYYLQYKNVRPDYVNAIWNVVSWENINERFLAAKK

>Branchiostoma.floridae_37_Mn XP_002596245.1

MAVQQVFCTIIFVVTILSNASAQDSMPSPRRQPKWCPVEVQEVKPYRVRSGSRTECRRACTWNRFCCEFNIIPVYTTAYRQVSRQEWKCCDDTVPCPDKTVSVDTPYKGIQLFADEYPVPPLPYEYDALEPYIDERTARVHHLGHHAGYTKKMNAALKAWRDEVGPGVGLQSNSILHILENLNQVPDKHRTAIRNNGGGFVNHAIYWATMSPRREGEPPRLPEATLATEIIANFQNFSNFQEQFTNTAKTLFGSGYVWLSRNPREQTLIIHTTANQDSPISDKLRPILVIDIWEHAYYLKHQNKRPDHIQDWWALVDWGQVKALSDWWAAYDAGHDEL

>Caenorhabditis.elegans_25_Mn F10D11.1.1

MLQNTVRCVSKLVQPITGVAAVRSKHSLPDLPYDYADLEPVISHEIMQLHHQKHHATYVNNLNQIEEKLHEAVSKGNVKEAIALQPALKFNGGGHINHSIFWTNLAKDGGEPSAELLTAIKSDFGSLDNLQKQLSASTVAVQGSGWGWLGYCPKGKILKVATCANQDPLEATTGLVPLFGIDVWEHAYYLQYKNVRPDYVNAIWKIANWKNVSERFAKAQQ

>Caenorhabditis.elegans_26_Mn F10D11.1.2

MLQNTVRCVSKLVQPITGVAAVRSKHSLPDLPYDYADLEPVISHEIMQLHHQKHHATYVNNLNQIEEKLHEAVSKGNVKEAIALQPALKFNGGGHINHSIFWTNLAKDGGEPSAELLTAIKSDFGSLDNLQKQLSASTVAVQGSGWGWLGYCPKGKILKVATCANQDPLEATTGLVPLFGIDVWEHAYYLQYKNVRPDYVNAIWKIANWKNVSERFAKAQQ

>Caenorhabditis.elegans_27_Mn F10D11.1.3

MLQNTVRCVSKLVQPITGVAAVRSKHSLPDLPYDYADLEPVISHEIMQLHHQKHHATYVNNLNQIEEKLHEAVSKGNVKEAIALQPALKFNGGGHINHSIFWTNLAKDGGEPSAELLTAIKSDFGSLDNLQKQLSASTVAVQGSGWGWLGYCPKGKILKVATCANQDPLEATTGLVPLFGIDVWEHAYYLQYKNVRPDYVNAIWKIANWKNVSERFAKAQQ

>Caenorhabditis.elegans_28_Mn C08A9.1

MLQSTARTASKLVQPVAGVLAVRSKHTLPDLPFDYADLEPVISHEIMQLHHQKHHATYVNNLNQIEEKLHEAVSKGNLKEAIALQPALKFNGGGHINHSIFWTNLAKDGGEPSKELMDTIKRDFGSLDNLQKRLSDITIAVQGSGWGWLGYCKKDKILKIATCANQDPLEGMVPLFGIDVWEHAYYLQYKNVRPDYVHAIWKIANWKNISERFANARQ

>Capitella.teleta_16_Mn CapteP152709

MLSAAVNSLKHAVPRCAVSSRLKHTLPDLPYDYNALEPTISADIMQLHHSKHHATYVNNLNVAEEKLAEARATGDVSTEIALGSALIFNGGGHINHSIFWQNLSPNGGGEPTGELMSAIQRDFGSFENMKKTLSASTVAVQGSGWGWLGYNKAAGKLQIATCANQDPLEATKGLVPMFGIDVWEHAYYLQYKNVRPDYVNAIWNVANWADITERFNKARQ

>Capitella.teleta_46_Mn CapteP229194

MSLSSSNFPTICIILVGCHLSGLNGESPFTTIASKAELYSLPALTFGYADLEPFFDEATLHAHYDGHHETYRKKMNSALSEWRESDPLDSFSTEPILEILKNLNKVPENFRNAIKNSGGGFVNHALYWACMSPNPTREIRQPTGALLQDIENEFGSFVKFSLKFTDRAVSLFGSGYVWLSRNPSSGALIITTTVNQDSPISDGLHPILVIDVWEHSYYLKHQFRRHVYVADWWKVVDWENVEELDKWWREVGSYARDEL

>Capitella.teleta_31_Mn CapteP95976

HISKETLDYHYGKHHNTYVVKLNGLVEGTELANKSLEEIIKTSEGGIFNNAAQVWNHTFYWNCLSPNGGGKPSGELAAAIDKAFGSFDEFVAKFSDMAVNNFGSSWTWLVKNTDGSLEIVNTSNAATPLTGDQKPLLTCDLWEHAYYIDYRNVRPDYLKGFWALANWEFVADNFAG

>Ciona.intestinalis_21_Mn ENSCINT00000008803

MLRLLSSRCTSKVVPVWASRGKHTLPDLPYDYSALEPHISAEIMETHYAKHHATYVNNLNIAEEKLHEAEAKNDISSIISLGPALKFNGGGHINHSIFWETLSPNGGSEPCGELKTAIDRDFGSFENLKAKLTAASVGVQGSGWSWLGLDKEKGQLQVVACPNQDPLHATTGLVPLFGIDVWEHAYYLQYKNVRPDYIKAIFNVVNWENVGKRFTDA

>Danio.rerio_3_Mn NP_956270.1

MLCRVGYVRRCAATFNPLLGAVTSRQKHALPDLTYDYGALEPHICAEIMQLHHSKHHATYVNNLNVTEEKYQEALAKGDVTTQVSLQPALKFNGGGHINHTIFWTNLSPNGGGEPQGELLEAIKRDFGSFQKMKEKISAATVAVQGSGWGWLGFEKESGRLRIAACANQDPLQGTTGLIPLLGIDVWEHAYYLQYKNVRPDYVKAIWNVVNWENVSERFQAAKK

>Drosophila.melanogaster_30_Mn FBgn0010213

MFVARKISQTASLAVRGKHTLPKLPYDYAALEPIICREIMELHHQKHHQTYVNNLNAAEEQLEEAKSKSDTTKLIQLAPALRFNGGGHINHTIFWQNLSPNKTQPSDDLKKAIESQWKSLEEFKKELTTLTVAVQGSGWGWLGFNKKSGKLQLAALPNQDPLEASTGLIPLFGIDVWEHAYYLQYKNVRPSYVEAIWDIANWDDISCRFQEAKKLGC

>Ephydatia.muelleri_35_Mn Em0018g430a

MLWICLLCFVFACNSEQIYEDLVVYHDELKLPDLPYDYNALEPYIDEATMKVHHLGHHKTYGDNTNKALKEWRKGDPTSPLARSSIVQILQKITEVPETWQVAIRNNGGGFVNHVIYWATMCPGGGGSPSGELLMQISRSFGSYVAFQEHFTSAASKLFGSGYVWLCENAKGDLVITSSKNQDSPLSDGLYPLLVMDIWEHAYYLKHQNKRAGYIQSWWNIVCWDEVPKLQHFWHQNRN

>Homo.sapiens_14_Mn SOD2

MLSRAVCGTSRQLAPVLGYLGSRQKHSLPDLPYDYGALEPHINAQIMQLHHSKHHAAYVNNLNVTEEKYQEALAKGDVTAQIALQPALKFNGGGHINHSIFWTNLSPNGGGEPKGLIPLLGIDVWEHAYYLQYKNVRPDYLKAIWNVINWENVTERYMACKK*

>Homo.sapiens_4_Mn SOD2.2

MLSRAVCGTSRQLAPVLGYLGSRQKHSLPDLPYDYGALEPHINAQIMQLHHSKHHAAYVNNLNVTEEKYQEALAKGELLEAIKRDFGSFDKFKEKLTAASVGVQGSGWGWLGFNKERGHLQIAACPNQDPLQGTTGLIPLLGIDVWEHAYYLQYKNVRPDYLKAIWNVINWENVTERYMACKK*

>Homo.sapiens_7_Mn SOD2.3

MLSRAVCGTSRQLAPVLGYLGSRQKHSLPDLPYDYGALEPHINAQIMQLHHSKHHAAYVNNLNVTEEKYQEALAKGDVTAQIALQPALKFNGGGHINHSIFWTNLSPNGGGEPKGELLEAIKRDFGSFDKFKEKLTAASVGVQGSGWGWLGFNKERGHLQIAACPNQDPLQGTTGLIPLLGIDVWEHAYYLQYKNVRPDYLKAIWNVINWENVTERYMACKK*

>Homo.sapiens_8_Mn SOD2.4

MLSRAVCGTSRQLAPVLGYLGSRQKHSLPDLPYDYGALEPHINAQIMQLHHSKHHAAYVNNLNVTEEKYQEALAKGDVTAQIALQPALKFNGGGHINHSIFWTNLSPNGGGEPKGELLEAIKRDFGSFDKFKEKLTAASVGVQGSGWGWLGFNKERGHLQIAACPNQDPLQGTTGLIPLLGIDVWEHAYYLQYKNVRPDYLKAIWNVINWENVTERYMACKK*

>Homo.sapiens_5_Mn SOD2.6

MLSRAVCGTSRQLAPVLGYLGSRQKHSLPDLPYDYGALEPHINAQIMQLHHSKHHAAYVNNLNVTEEKYQEALAKGELLEAIKRDFGSFDKFKEKLTAASVGVQGSGWGWLGFNKERGHLQIAACPNQDPLQGTTGLIPLLGIDVWEHAYYLQYKNVRPDYLKAIWNVINWENVTERYMACKK*

>Homo.sapiens_9_Mn SOD2.7

MQLHHSKHHAAYVNNLNVTEEKYQEALAKGDVTAQIALQPALKFNGGGHINHSIFWTNLSPNGGGEPKGELLEAIKRDFGSFDKFKEKLTAASVGVQGSGWGWLGFNKERG

>Homo.sapiens_11_Mn SOD2.8

MFAFWPTHRRVAFQAWPQSLARRRTPPGEESTSRQLAPVLGYLGSRQKHSLPDLPYDYGALEPHINAQIMQLHHSKHHAAYVNNLNVTEEKYQEALAKGDVTAQIALQPALKFNGGGHINHSIFWTNLSPNGGGEPKGELLEAIKRDFGSFDKFKEKLTAASVGVQGSGWGWLGFNKERGHLQIAACPNQ

>Homo.sapiens_12_Mn SOD2.9

MLPRVGGPGATSRERPWAASRGRLLLAEAHLTSRQLAPVLGYLGSRQKHSLPDLPYDYGALEPHINAQIMQLHHSKHHAAYVNNLNVTEEKYQEALAKGDVTAQIALQPALKFNGGGHINHSIFWTNLSPNGGGEPKGELLEAIKRDFGSFDKFKEKLTAASVGVQGSGWGWLGFNKERGHLQ

>Homo.sapiens_6_Mn SOD2.11

MQLHHSKHHAAYVNNLNVTEEKYQEALAKGELLEAIKRDFGSFDKFKEKLTAASVGVQGSG

>Homo.sapiens_10_Mn SOD2.12

MQLHHSKHHAAYVNNLNVTEEKYQEALAKGDVTAQIALQPALKFNGGGHINHSIFWTNLSPNGGGEPKGELLEAIKRDFGSFDKFKEKLTAASVGVQGSGWGWLGFNKERGHLQIAACPNQDPLQGTTGLIPLLGIDVWEHAYYLQYKNVRPDYLKAIWNVINWENVTERYMACKK*

>Lingula.anatina_38_Mn g32380.t1

MVVSSHRFVASWPYDDSALSKETYLLPALPYGYGALEPHMDEATVHHQGHHAAYTAKMNAALKQWRRYVWLSRDQDGKLIISTTANQDSPVTERLHPILVIDIWEHAYYLKHQNKRANFVEAWWNLVDWRAVETLDTWWKGRQTRDEL

>Lingula.anatina_29_Mn g595.t1

MLCARIGMLKRFVPQQKAALGFTASRLAHSLPDLPYDFNALEPVIGAEIMEIHYKKHHATYVNNLNIAEEKMEKALAEGDTNAAISLLPALNFNGGGHINHSIFWTNLSPNGGGEPTGDLKESINRDFGGFESMKKELISKTVAIQGSGWGWLGYNPHTQRLRLAVRPNQDSLFPTLGLIPLLGIDVWEHAYYLQYKNDRGAYVNAIWNIINWDNVADRYANARMQN

>Lingula.anatina_39_Mn g20669.t1

MAIVTVLFCVITLIVTPHQTADARMYEDCVQQREDYPLPPLPYSYNSLEPHMDATTVQIHHLKHHAAYTKKMNAALKQWRDEDPENTLATGSIVEILQHLYNVPEKYRTVLRNNGGGYVNHAIYFACMSSRGAAISQDLENDIIKDFGSYNNFTKQFTTKAMGLFGSGYVWLSRGGNDKLVISTTANQDSPVTESFRPILVIDIWEHAYYLKYQYRRADFVAAWWKLVDWKAVVDLDKWWRVNYRYG

>Lingula.anatina_40_Mn g20670.t1

MAIVTVLSCLITLVVTPHQTAAIGMYEESAVPKEAYPLPPLPYPYGGLEPHMDERTVQIHHQGHHAAYTTKMNAALKQWREEDPGNTFAKSSILNILQNVSSVPEKYRTALRNNGGGYFNHAIYWACMSPGGAAITPDLKSDIIQNFGTYDNFKDQFTTKAMALFGSGYVWLSRGENEQLVISTTANQDSPVTEDLHPILVIDIWEHAYYLKHQNKRADFVADWWKLVNWNAVRDLDQWWKRI

>Lingula.anatina_41_Mn g32378.t1

MAIVTVLFCVITLIVTPHQTAAAAMYEDSVVLREDYLLPPLPYAYNSLEPHMDEKTVEASNMTYDSPHPSFQFPYVIHVASIHHLEHHAAFTTKMNAVLKQWRDEDPENTFAKSSILNILENLYDVPEKYRTALRNNGGGYFNHAIYWACMSPRGAVTCINPDLENDIKKDFGSYNNFTNQFTTKAMALFGSGYVWLSRGKNEQLVISTTANEDSPVTEDLRPILVIDIWEHAYYLKHQNKRADFVEAWWNLVNWDAVWDLDQWWKEKHMSMDSGSKNMKDEPCSEMGRSDSLVDSVEEKKRRTWGGQLEFIISCAAVGLGAVWRFPYLCYRNGGGAFLIPYCVSWALIILPLYFMELCFGQFASLGPIAVWKINPLFKGIGYGMVVTCWFLTLYYSVLIGYCIHYFFASMTAILPWSTCGNAWNTEDCLVDKNSKYITVDGWNITVNPMGYTLFNICSRKVLEMTDGIENSGGLKWDLVLCHLLAWVIVSLVLIKGVKSMGKAIYFTAIFPHIIMAVLLVRGALLEGSAEGIKYYLTPDFKRLLHSEVWGDAVTHNFYSLSISLGGLVMMSSHNKFHHNCFRYTGLLTETVANAVSEFTAFS

>Lingula.anatina_42_Mn g20666.t1

MAVSSLRFVASWPYDDSGFSKDTYLLPALPYGYGALEPHMDEATVKVHHQGHHAAYTAKMNAALKQWRSEAAGDKFANSSILNILQKLSHVPEKYRMTLRNNGGGYFNHAIYWACMSPKGARISPGLEKDIKDNFGTYENFKEQFTAKSLSLFGSGYVWLSRDQDGKLIISTTANQDSPVTERLHPILVIDIW

>Mnemiopsis.leidyi_22_Mn ML012511a

MLQSRLLKLSRLAQARCKYTLPELPYNYEALEPVINREIMEIHHSKHHATYVNNLNATAEKVSAAVAEGNAGAVIGLHGALKFNGGGHINHSIFWNNLSPNGGGVPSGELLSAIEAEFGSFDQFKAKMSAATIAVQGSGWGWLGLNPDSKRLQIATCANQDPLQATTGLAPLLGIDVWEHAYYLQYKNVRPEYVNSIWDIVNWEDVAARYAAASS

>Nematostella.vectensis_43_Mn EDO44816

MEKYTLPELPYDYNELEPHIDEATLRVHHLGHHAAYTKKLNAALKDIVEILRNNEQIPDKWRTDVINNGGGFVNHALYWATMSPNPKSEPRTPTGKIGDLIDKSHGNFSMFKQWFDEQVNSMFGSGYTWLCQDVTSGFLTILNMGNQESPVAYRLNPVLVIDLWEHAFYLKHQNKRPGYVHSWWHLVDWERVNELLEWWQNQNIHDEL

>Nematostella.vectensis_32_Mn EDO27221

MAFELPKLPYAYDALEPHIDARTMEIHHSKHHNGYTTNLNNAISGTDLEGKSIENILINLDLNNGAVRNNGGGYYNHNLFWTVMSPNGGGNPTGELAAAIDAAFGSFEAFKTEFSKAAATRFGSGWAWLCVHKGGKLEVCSSANQDNPLMPGIGCGGTPILALDVWEHAYYLNYQNRRPDYVEAFFNVIDWTEVTRRFATEK

>Nematostella.vectensis_15_Mn EDO49016

MLLARVARLSSRKAVLPVALVRAKHTLPDLPYDYDALEPTINTEIMRLHHSKHHATYVNNLNIAEEKCLEAQAKGDVATAIALQPAVKFNGGGHLNHSIFWTNLSPNGGGEPTGELMEAIKRDFGSFENFKERFNAATIAVQGSGWGWLGYDKVNKRLAIATCFNQDPLQPTTGLVPLLGIDVWEHAYYLQYKNVRPDYVKAIYDVINWTNVAERLQAASS

>Oscarella.carmela_49_Mn m.306373

LCGRGAPYCDPITFSAMAGSGVDFPIVPPSLMSYLKIKDSAEGYLLPEVGYDYADLEPHIDEATMKLHHSRHHAGYTRKMNAALDAWRKSGEVPELAAKPIENIIQHVDDVPKKWQRAIRNNGGGFINHAFYWQNMSPPNDPNAQREPSGPLLEKIKSNFGSFEALKQKITETAAGLFGSGWVWLVEVPGSGELQLLSSENQDNPMSQGLKPILALDVWEHAYYLKHKNDRPSYIQSWWNIADWRKVGDILAAWNSQQ*

>Oscarella.carmela_17_Mn m.307964

RSVPYNAMSQALLSRLGVRSLGGFLCCRSKHTLPDLPYDYGALEPAISAEIMQLHHSKHHATYVNNLNGAEEKLAEALATGDTASQIALQPALKFNGGGHINHSIFWTNMCPGGSGEPQGDLMNAIKRDFGSSEKLKTSLSAASVAVQGSGWGWLGYNKIRKRLEVATCANQDPLEATTGLVPLLGIDVWEHAYYLQYKNVRPDYVKAIWEVVNWPNVAERFAAAQGMPKR*

>Oscarella.carmela_44_Mn m.309974

MMKPVAFPSLFLLCLAWSYALDIPDGLQKLLKIEKPEDGADYKLPTLDFDYNALEPYIDEKTVRVHHLGHHAGYTHKMNAILRKWAEKKEQQDLLSLPIVNILEQVNDIPLDIREDLQNHGGGFINHAWYWATIAPVKAESRESEPVGSVAGAIEKRYGDFKKFQQEFNHTAFTFFGSGYVWLCEVPPTGILQITTFPNQMSPLGFGLKPILVLDVWEHAYYLKHQNKRAGYVHDWWEIIDWNKVELISQAWKNEQAKDEL*

>Strongylocentrotus.purpuratus_45_Mn SPU_021817

MASACYVDSKMFAFFIILWMCTTSYCVSYPFECIAEIRSEYILPDLPFGYDDLEPYIDSATLNVHHRGHHAGYTRKLNAALHQWGSEEPDSYAATSILDLLQHLDDIPPKYKQSIRNNGGGFVNHNIYWSTMASNKENVTTMPSGDLLGEINESFGSFEEFKNQFTGRALTLFGSGYVWLSRRKPSSPFEEVEEEPASLIISTTANQDTPISNGLQPILVLDVWEHAYYLKHQNKRANHIEDWWRVVDWSAVANLRDWWSGDYEVHDEL

>Strongylocentrotus.purpuratus_23_Mn SPU_024657

MQIFRLPPPLQNVTFPHGVTAPGRALLRSQARVVSGVSSLHTLPELPYDYNALSPVISTEIMELHHKKHHNTYVTNLNVAEEALEHAVEAGDISTIISLQAALRFNGGGHINHTIFWQNLSPDGGGEPTGELLNVIKKDFGSYDDMRSKLSAATVAIQGSGWGWLGYNKGTRSLQIATCPNQDPLKATTGLIPLFGIDVWEHAYYLQYKNVRPDYVKAIFNIANWSDVEKRLTDAMTT

>Sycon.ciliatum_50_Mn scpid75220

MNHICSVAACLQLLVCVFALSPVRLFVLAAEDIDDHADASSGEIEAASDPPAWLPAVSVRLLELLDIRLENGSYELPRLMYSYEALEPYMSERTVRVHHTGHHAAYTKKMNSILSRMPSADAVVASNPLQQLMNLDALPEDVRDAFRNQAGGFVNHAFTFFVMGPLDQSVEVADQGPSGKILELITESFGSTDAMKEQFSGAAAKLFGSGYVWLCLENVAGVRRLTITENANQDSPLSGGFRPVLGIDLWEHAYYLQYQNKRVDYIATWWQLVSWERVGLLLDAWIETEPADSTASSVEEQVGTTPPDDTPETRDEL

>Sycon.ciliatum_18_Mn scpid63635

MALALCSAARQCAARSLASACAAQKHTLPDLPYDYGALAPHISPEIMQLHHSKHHQTYVNNLNVAEEKLQECAAKGDASGVIALGGALKFNGGGHINHSIFWQNLSPNGGGEPTGELNNAIVTQFGSFDAFKQKMTTAAVGVQGSGWAWLGLNKETMQLSVSACPNQDPLQSTTGLVPLLGIDVWEHAYYLQYKNVRPDYLKAIWNVINWEDVASRYTGAQ

>Tethya.wilhelma_36_Mn Twilhelma_g11565.t1

MKIVFAALLLSLLHCNSGQRDAYEQLTHPATDYPLPDLPYSYDELEPLIDTHTLKVHHLGHHKAYTDKMNAALNQWKHEEPTSKLAQSSIITILQSILSVPEKWQTAIRNNGGGYVNHIFYWVTMCPHGHDQDVPGGKLLEAIINTFGGFEEFKSEFSQTAAKLFGSGYVWLCESQAGDLSIVTTYNQDSPLSDGLYPLLVLDVWEHAYYLKHQNKRTEYIRDWWSVVCWDHVADLEQFWMTGANRNKHTEL

>Tethya.wilhelma_24_Mn Twilhelma_g23748.t1

MLSAASRQLLRHSRQATCVAATQTRNKFTLPELPYKYNALEPVISGEIMELHHSKHHATYVNNLNAAEEKLKQAIEDGNIKGILDTQGAIKFNGGGHLNHSIFWTHLSPNGGGKPTGDLAAAIDRDFGSFDNLMDQMTTKTVAIQGSGWGWLGYSKSNGRLVISTTANQDPLQATTGLIPLLGIDVWEHAYYLQYKNVRPEYVKAIWKVMDWKDIESKFSAAKSS

>Xenopus.tropicalis_13_Mn NP_001005694.1

MLCRLSVCGRGRLRCAPALTYFTSREKHTLPDLPYDYGALQPHISAEIMQLHHSKHHATYVNNLNITEEKYAEALAKGDVTTQVSLQAALKFNGGGHINHTIFWTNLSPNGGGEPQGELLDAIKRDFGSFEKFKEKLSTVSVGVQGSGWGWLGYNKESNRLQLAACANQDPLQGTTGLIPLLGIDVWEHAYYLQYKNVRPDYMKAIWNVINWENVAERYRASKK

>Xestospongia.bergquistia_19_Mn Xb.06078.mrna

MLSILRASHPFKGTGVTALTSLSRQNHSLPELPYRYNALEPVISGEIMELHHKKHHATYVNNLNTAEEQLKQCVDEGNVGGVIALQPAIKFNGGGHINHSIFWNNLSPNGGGEPQGDFRAAIERDFSSFDNFKSQLSAKTIAIQGSGWGWLGYNKESGRLQIATCANQDPLQGTTGLIPLLGIDVWEHAYYLQYKNVRPDYVKAIWDVINWQDVTERYSNAMT*

>Xestospongia.bergquistia_47_Mn Xb.08027.mrna

MRSGWKFVQCCQEKQNLFGITGTAGTVLPPVVVDINKDGKMDLVISQFNDHTILYDGGSGSIAWDHYNHNTQTYSVPAPIHFNNDDTLDILVRFNKGHWMTYDYSYMGILDGNNGELLWSLNCSQGVMSSPVTLQHKNRGQDGMLFIAIGCGRMPQKETIEENESGRRRKRSDICPRTFFENYGQVYERKEYERKEAVGQEGFFPDSQTEGSGNNMHSPVNPAGTDIDFSQFLPDDLWEIRDNSDSFPDPWKKTEEFIEDYCEYEPDLLEASVYFLTPELTSTSKINPLFVFQPYVYKPSSISTSSTQNGHKKRHGDSDPEDSGREHSIMELMKNRKALRCAHILAPVQSASTPAITDVNGDGKLDAVVSIAYSEASGKYGMPELSFLHPPKVVVQTFTLEDMLKAIYKDSAANVDFSSYYSMDKQPWTEYMGRNGDGIFVDEATIYDRIFQKADKYELPNLPYQYNDLEPYLDERTLIVHHQGHHKAYTDKMNAALQEWREKYHWSHDLELDQIPEKWQTALKNNGGGYVNHIFYWATMCPIPKAPPPTLTAAIEENFPGGISYFFDNFTSSAKSLFGSGYVWLVQDKEDNLYIIQTKDQCYFLRVVINLDFLRSKQTGHVLADKKQSLERERRGLVHILQEYLSSSLCHYVNPVSKGMNKSQITAIELKSSGDEHQNYHDVSNSSIVTRDFQFLQELVPFQNDSLQVSKQWLFENANHFSLDLDLNQIDLDLNQIDFDLNQIDLDLNHEGDSPVANLNISVNEYSSFSISRFTGDDPNELVCNDSRLSVGPWSSNGGSEDERG*

>Xestospongia.bergquistia_48_Mn Xb.28131.mrna

MVYMCFIFLLLSRVQVDEATIYDRIFQKADKYELPNLPYQYNDLEPYLDERTLIVHHQGHHKAYTDKMNAALQEWREKMPHDTLATLSIFDILKRLDQIPEKWQTALKNNGGGYVNHIFYWATMCPIPKAPPPTLTAAIEENFPGGISYFFDNFTSSAKSLFGSGYVWLVQDKEDNLYIIQTKDQ

>Arabidopsis.thaliana_SODMn sp|O81235|SODM1_ARATH Superoxide dismutase [Mn] 1, mitochondrial OS=Arabidopsis thaliana

MAIRCVASRKTLAGLKETSSRLLRIRGIQTFTLPDLPYDYGALEPAISGEIMQIHHQKHHQAYVTNYNNALEQLDQAVNKGDASTVVKLQSAIKFNGGGHVNHSIFWKNLAPSSEGGGEPPKGSLGSAIDAHFGSLEGLVKKMSAEGAAVQGSGWVWLGLDKELKKLVVDTTANQDPLVTKGGSLVPLVGIDVWEHAYYLQYKNVRPEYLKNVWKVINWKYASEVYEKENN

>Dictyostelium.discoideum_SODMn sp|Q55BJ9|SODM_DICDI Superoxide dismutase [Mn], mitochondrial OS=Dictyostelium discoideum OX=44689 GN=sod2 PE=3 SV=1

MLPRSLKLIKKVGESNGLRNFGSQSNSYTLPDLPYDYGALSPVISPEIMTLHHKKHHQTYVNNLNIALDKLSSASSAKDVAQMIALQSAIKFNGGGHVNHSIFWTNLAPKNQDGGVAPSGPLADAINKQYGSIEKLIEKMSAETTAIQGSGWGWLGYDKANDRLVIQTQQNQDPLSVSGYVPLLGIDVWEHAYYLDYKNVRADYVKNIWQIVNWKNVAERYNTAKK

>Trichoplax.adhaerens_1_SODMn tr|B3RPN1|B3RPN1_TRIAD Superoxide dismutase OS=Trichoplax adhaerens OX=10228 GN=TRIADDRAFT_21084 PE=3 SV=1

MLANVLRQSLGRNSCHSALLITQARRKHDLPPLPYAYNALEPTISAEIMELHHSKHHQTYVTNLNAAEEKLAEATSKNDISGVITLQGALRFNGGGHINHSIFWKNLSNDGGGLPTGELGDAINACFGSFDNFKSKLSAATIAIQGSGWGWLGYCKESNSLKIATCANQDPLQATTGYVPLLGIDVWEHAYYLQYKNVRPNYVNAIFDVINWNDVANNFRNAKA

>Trichoplax.adhaerens_2_SODMn tr|B3SFQ5|B3SFQ5_TRIAD Superoxide dismutase OS=Trichoplax adhaerens OX=10228 GN=TRIADDRAFT_35123 PE=3 SV=1

MIKALPLPYDKNELEPTLSKNTFDYHYDKHYLGYANKLSALVDGTKYYNMLLADIIIESYKNNDTPIYNNAAQVWNHIFYWKSIGKTQKCPTKLIELLNRDFGSYDTFIEKFIQAGVTLFGSGWIWLVQDKESKKLSILQTKDADNPLILDKTPILTIDVWEHAYYIDYKNDRLKYLDQIIKNNIKWLFAVSNLN

>Trichoplax.adhaerens_3_SODMn tr|B3SFI3|B3SFI3_TRIAD Superoxide dismutase (Fragment) OS=Trichoplax adhaerens OX=10228 GN=TRIADDRAFT_35056 PE=3 SV=1

EDIVKKSYISKDTKIFNNAAQVWNHEFYWDSIRKKTIIDDLNQNESLEAKPTLYDLFNKINECFGSFEEFCKKFVEAGVALFGSGWIWLVQDPDTKKLEIMQTYNADSPMLHNKIAILTIDVWEHAYYIDYKNDRLSYLENVIKFHLNWDFAKNNMKL

>Podospora anserina_1_SODMn tr|Q711T6|Q711T6_PODAS Superoxide dismutase OS=Podospora anserina OX=2587412 GN=sod2 PE=3 SV=1

MAVQEYTLPALPYAYNALEPHISAQIMELHHSKHHQAYVTNLNNALRLHVAAVGAGDIASQIEMQQVIKFNGGGHINHSLFWKNLAPAESEETKPEAAKELVAAVEKTWGSLDDFKNAFSSTLLGIQGSGWGWLVKDSANGLRIVTTKDQDPVVGRDVPVFGVDMWEHAYYLQYLNGKAAYVENIWNVINWKTAEERFLGTSEATKL

>Podospora.anserina_2_SODMn tr|Q9C208|Q9C208_PODAS Superoxide dismutase (Fragment) OS=Podospora anserina OX=2587412 GN=sod2 PE=2 SV=1

NLNNALRLHVAAVGAGDIASQIEMQQVIKFNGGGHINHSLFWKNLAPAESEETKPEAAKELVAAVEKTWGSLDDFKNAFSSTLLGIQGSGWGWLVKDSANGLRIVTTKDQDPVVGRDVPVFGVD

>Monosiga.brevicollis_SODMn tr|A9VBJ0|A9VBJ0_MONBE Superoxide dismutase OS=Monosiga brevicollis OX=81824 GN=34408 PE=3 SV=1

MAMLARAMRSATTGARRMKHTLPDLAYDYAALEPVISAKIMELHHSKHHNTYVNNLNIAEEQYAEAVHTGDLTKAIGLQSAIKFNGGGHINHSIFWTNLAPKEQGGGELQDGELKTAIEEAFGSVETMQQKLNAMTAAVQGSGWGWLGYNKASKQLQLATCANQDPLETTHGLVPLFGIDVWEHAYYLDYKNVRPDYLKAVWEIANWQNVEERYQAAKL

>Algoriphagus.machipongonensis_SODMn tr|A3HUU8|A3HUU8_9BACT Superoxide dismutase OS=Algoriphagus machipongonensis OX=388413 GN=ALPR1_01725 PE=3 SV=1

MAFELPSLPYAYDALEPNIDAKTMEIHHSKHHNGYVTNLNKAVEGTDLEGKSLEELLKIAGSNTAVRNNGGGHFNHSLFWSILSPDGGGEPTGELADSISAKFGSFAAFKETFNKAAATRFGSGWAWLCIDTKKELCVCSSPNQDNPLMDVAECPGTPILGLDVWEHAYYLNYQNRRPDYINAFWNVIDWDAVSKRYAAAK

>Chondrus.crispus_1_SODMn tr|R7QCE3|R7QCE3_CHOCR Superoxide dismutase OS=Chondrus crispus OX=2769 GN=CHC_T00003067001 PE=3 SV=1

MSFALPNLPYQYDALEPYVDSTTMNIHHTKHHQTYVNNINKVIDGPSGSALKGLSLPAIQANITSLPAEIQTPVINSGGGHFNHAMFWTLMGRPGSCNTAPVGSIKDKINADFGSFDEMKAKFNSAAAARFGSGWAWLSVGADGKLFISSTKNQENPLMAGVVDQPGSPVLGLDVWEHAYYLKYQNRRPEYISAFWNVVNWDQVTKNYDSVCSGNTAVFDVPMA

>Chondrus.crispus_2_SODMn tr|R7QI02|R7QI02_CHOCR Superoxide dismutase OS=Chondrus crispus OX=2769 GN=CHC_T00005214001 PE=3 SV=1

MVWLGNALFISPNPSTRRFATLLDCIASISCLPPAHAAPQVTIDIALANYQHSMALRAGASRLLSRAAPLARSLSQDVASPAARSVSTFSLPDLKYGYASLEPHFDEKTMTVHHTRHHQTYVSNLNGVLTGEHGSEIQDLSLSQIQRKVSSLPEAIRTTVMNHGGGHYNHTLFFSILAPETETTSAPVDELKTMVETDFGSFDAMRKEFNAAAMKVFGSGWAWLGVGNDGKLCISNTRNQENPLMEGIVDAPSTPIMGLDIWEHAMYLKYLNRRPEYIDAFWHIIDWEQVAANYSAARDGQTAIFDVPMEE

>Pyropia.yezoensis_SODMn tr|Q3YB09|Q3YB09_PYRYE Superoxide dismutase OS=Pyropia yezoensis OX=2788 PE=2 SV=1

MAFALPPLPYAYDALEPYIDSTTMNIHHTKHHNTYVTNVNNVLAGENGGALKGLSLSAIQKEVVTLPDSIKTAVRNSGGGHWNHSFFWSVMGKTGSIAEAPTGDLKSSIESTFGSLDEMQKKFNTAAASRFGSGWAWLSVNADGQLFISSTPNQDNPLMEGIVDQPGTPILGLDVWEHAYYLKYQNRRPEYIASWWKTVDFDVIAKNYSAAKSGGLPAFDTPLV

>Gloeobacter.violaceus.PCC7421.sodB_MnSOD.1 tr|Q7NDA9|Q7NDA9_GLOVI Superoxide dismutase OS=Gloeobacter violaceus (strain ATCC 29082 / PCC 7421) OX=251221 GN=sodB PE=3 SV=1

MAHTLPPLPYDENALAPYVSAQTLSFHYGKHHTGYLNNMNKAIAGTELESLSLVDLIRTAAKNAEQKTLFNNAAQVWNHTFYWNSMRPGGGGEPGGTLGEMIKDAFGSYDEFKKQFVTAGTTQFGSGYAWLVKDGEKLVVTKTPNAETPITDESKLPLLNMDVWEHAYYLDYQNLRPDYENAFADNLINWEFAEKNLERVFAG

>Gloeobacter.kilaueensis.JS1.sodA_MnSOD tr|U5QI99|U5QI99_9CYAN Superoxide dismutase OS=Gloeobacter kilaueensis JS1 OX=1183438 GN=sodA PE=3 SV=1

MAFTLPPLPYDESALAPYISAQTLSFHYGKHHKGYVDTLNKLVAGSEAENTPLEELIKSVHGQPDKAAIFNNAAQIWNHTFYWNSLKPGGGGEPTGTIAELIKDAFGSYDEFKKQFITAGTTQFGSGYAWLVKDKNSGKLSVIKTPNAETPLTDPTKVPVLTFDVWEHAYYLDYQNLRPKYEEAVVDHLLNWEFAEKNLASA

>Gloeobacter.violaceus.PCC7421.sodB_MnSOD.2 tr|Q7NMT3|Q7NMT3_GLOVI Superoxide dismutase OS=Gloeobacter violaceus (strain ATCC 29082 / PCC 7421) OX=251221 GN=sodB PE=3 SV=1

MVLGVLLTGKARYSPLLKAACNFPAGRAPCGSPEAKTGSLCQEFVSMKCSVSTLVGTLCLTAAALLSTAAEAQINIPTPTITPPTVTVPSVQPPSVTMPSATQASTTASGYPYKLAPLPYDYSALEPYIDAETMKLHHDKHHQAYVDNLNKALEKYPDLQKKSPEQLLRDLKQVPEDTRAAVRGNGGGHVNHTMFWEIMKPKGGGEPAGPIAAAIRTNFGSFDAFKTQFNEAGTKRFGSGWVWLVSNPGGKLEIVSTANQDSPIADGKYPIMGNDVWEHAYYLKYQNRRADYLSAWWNTLNWDEINRRFQKASTGL

>Pseudanabaena.ABRG5-3.sodB_MnSOD tr|A0A2Z5WSL4|A0A2Z5WSL4_9CYAN Superoxide dismutase OS=Pseudanabaena sp. ABRG5-3 OX=685565 GN=sodB PE=3 SV=1

MAFELPPLPYAQDALAASGMSAETLSFHYGKHHKAYVDNLNNLIKDTDLADKSLEEIIKISYKEGKAGIFNNAAQVWNHTFYWNGIKPAGGGAPTGALLDAINASFGSLDNFKTEFKNAGATQFGSGWAWLVAEGGTLKITKTPNAENPLIHEGQVPLLTMDVWEHAYYLDFQNSRPNFMANFVEKLINWDFVAANFAAV

>Pseudanabaena.PCC7367_MnSOD tr|K9SFI2|K9SFI2_9CYAN Superoxide dismutase OS=Pseudanabaena sp. PCC 7367 OX=82654 GN=Pse7367_0596 PE=3 SV=1

MKRRDFLTWLGVGVGTLAIANDFLLSPQAAKAMGLTGSLDHLLAQADTPDDPFTLPPLPYDYNALEPHIDVRTMQIHHDRHHAGYVRNLNKAIATYPDLAGMSAEDMLRDLTQVPEPIRTTVRNNAGGHVNHSMFWEIMSPNGGGTPTGAIAAMIAATFGNFENLQTAFNQAGASRFGSGWAWLVLDKQGNLKVTSTANQDSPLLEGLFPIMGNDVWEHAYYLNYQNRRGDYLKAWWNLVNWEEVNNRFLNAMV

>Pseudanabaena.PCC7429_MnSOD tr|L8MYC0|L8MYC0_9CYAN Superoxide dismutase OS=Pseudanabaena biceps PCC 7429 OX=927668 GN=Pse7429DRAFT_1829 PE=3 SV=1

MAFELPSLPYAQDALAASGMSAETLSFHYGKHHKAYVDNLNNLIKDTDLADKPLEEIIKISYKEGKAGIFNNAAQVWNHTFYWNGIKPAGGGAPTGALLDAINASFGSLDNFKTEFKNAGATQFGSGWAWLVAEGGTLKITKTPNAENPLIHEGQVPLLTMDVWEHAYYLDFQNSRPNFMANFVEKLINWDFVAANFAAA

>Pseudanabaena.SR411_MnSOD tr|A0A256BCV0|A0A256BCV0_9CYAN Superoxide dismutase OS=Pseudanabaena sp. SR411 OX=1980935 GN=B9G53_17315 PE=3 SV=1

MAFELPSLPYAQDALAASGMSAETLSFHYGKHHKAYVDNLNNLIKDTDLADKSLEEIIKISYKEGKAGIFNNAAQVWNHTFYWNGIKPAGGGAPTGALLDAINASFGSLDNFKTEFKNAGATQFGSGWAWLVAEGDKLKITKTPNAENPLVHEGQVPLLTMDVWEHAYYLDFQNSRPNFMANFVEKLINWDFVAANFSAV

**NOX family**

>A.queenslandica_6_NOX2 Aqu2.1.33096_001 Aqu3.1.33096_001

MSLLPSGWIINEFPKWLVFGTWFLLNIVLFIYQFNLFQTSNTQFYMRFYLKEALAFARGPALPINFNVILILLPVCRNIISVIRGCTRVVPRSIRRVLDKNLTFHKAIAWMLVVSSTMHVVAHYYNYERLARYSPPNSLPPGDQATSIPTQALPFNGSVGQLNPIHACFITYAGVTGHIITLVLFLMITSSVTYIRRSFFEVFWYTHQLFIVFLVGLGIHQYGRLLPVFRSASQLQDVGLGVCAYAPTSSENYTRFCTNLNFAPAGPTSWMFLLCGLLIYGLERILRVVRGFYSVVIIKVVQHPSNTIEIQMRRKGFHAEAGQYVFVNCPSVAWFEWHPFTLTSAPEEDYFSVHIRILGDWTRGIADKLGMNRNDFQQSWELPSIYIDGPFGTASEDAFEHPVALLVGAGIGVTPFASVLKSLYYRITEEGSGLVLKKLYFVWICPETYAFEWFANLFQELEQQLIDKGIDDFLTSWIYLTRGWKDNQAFYLMLNEGRVTAEHATDDMVDAITGLRAETTFGRPEWEKIFEKVALAHPNTDVGVFFCGPSVLSHNLHKQCNAFTKKGGSAGARFYYNKENF*

>A.queenslandica_4_NOX5c DspNOX Aqu2.1.35277_001 Aqu3.1.35277_001

MERIAALLAAAFFLCCYRVCGNLQFESCSSQGSYVLIGDKVQCSGSGSCSGSNGTANGTYFSLPGVFPYPSCSNSSDDDDGLTVLSSQYRVGLCSSCGIQDLLVKRGQMIHWSATQDLNCSRIRLVMEADGCIGEPVSTFFMDSGSTHHVHTFNQTGVYQLYCNQDCSRLLATITVLSNGTETVEEVNHKEIIYAHAVVMVLTFAAILPLGALLANLKKPIIHMIVQPLGLILAIIGLLLAVVYKELNKSTHFDRLHTIFGLILMVIAVLALPMLKFSVMSPVREKWKKLVTLWHKRLGLVAVFSGIFNIFLGLSILPDVPNYVTILYGVWTFVLMVIFVIAKRGGSSMREDDEESALNTCCHYRKDEVTLTRRDSLPPSKLTRDPFEKEKFVMVRKKSVDMLELKLKPNAGGGGGGGGNMPQRKISNVHFGPVAIAAQLTPPVPYTPQDNTTQFGFNNQLANHYNEAKDDLSSGSSENEESSDDSCSVDGESPEVDIRGRKEGKCDSQFSLNSIASSGGSLRITRHSERAHKRVNEIFPLEQQQSGPPVIVTPSPLLQSSSSTTKNSTGFNEGSPNKEAPRKDSTSDEEEKQRKEVSPDNFPRRCPMSRSGFSDSSLPAAGQRRTRKNAYTAAPTDIEDRLKLPDEAPPQQQEPRRPGLQRGLSRNASFRRKQAMVDNTLRSVMEMSDNAIICANAAGEIVFWSAGAMKMFGYTPGEAIGSSLQIIMPHRFREKHQNGVNGRTDRATEYIRKHHCAPEEQKHARLYSVSGVSKTGKEFEVEVVVTSFVIEEDIFYTGIITEVKGLTRHLSVSSISSDVEIREKQQKAMAIGAQMKLAHKLKSADHINRNELIKILKTSLQGTQFEVPPEDITVMVDGIMKAADADETGIIKFVDLIELLVKHRLYISDSGFIAKYGKTKSSERKNIKATKRCSGQAANFLSSHVTSIGWGMTYLFVNVLLMLIGVLSTSNSGWGQWAYGTGPALSFNLVLVLVPMMKSLIQNMRGSPWLNKVLPLKDSLFFHLFIIFVIFGLTLIHTVTHLCSFAFDGVEENSTLADNLTDNVQTHLMPLITGGILILILLVMSVSSFKCLSKLCLFIGFNLIHWIGFVALYMILLIHGIDYYNPSFWKWLLPVILVYGLERAYFRWVVPRYTVQILKSSPYDEQSRTTKLEIQKPTHYKFIEGQHLLINMPQIGYFKWQPCYVSNTNKEKTLTLYFPHTGSGWLERVYQTYKENPIKPQDGEPSSQSVTITTPFGPGVDHVFDYKAIVLLCGAGGAGLPTCRSIIQKFLQYRRKGASCPCGLHPKLTKMYFVWVTPSGNDNEWFTDFLSELIEDAHLQKYLDIRIFLTNIDSPPDELASALLHLALKTGGPALSVRRPSQPQLSKLYANTSYGVPDFESLLDFVSMQQGTSDEKVAECGVFTAGSFPHTVIKDLVHYCDVCSKKKGPSLNFTNSPYRKLSASSPPNSIASPTRTDGGSPAETPQVPPEWHITFKHQPLIL*

>A.queenslandica_2_NOX5a Aqu2.1.36084_001 Aqu3.1.36084_001

MATMSSEVFISENEADLPEDQQVKGNEKSAILDRLGEVFSSVTVDGYLTVKDLSQAMTSHKDFSCMLFELIDVCSIGSVSPGEVIKAVTKLQKGNKKYLKRNDMKWFENKIWHVARNNNNSLKKDQFQELFEVDGFVVKFSELLLGGTDRINVSDMLQSFQLVLISDTEDNSLLWYERKFSAVMDGNKQVKFEEFKKALNITKSTFFAERFFQIFDTDHSGSVSVKELIDGLTIVIHGSQVDKLKFLFMVYDVDGNGYIDFEELKTVLRSCMDESALNLSDENLNELTNTLFEAADADRSGEISFDELKQELERHPEIVNNLTVSAASWLQPNSQKRSCFEHVPHWLKWKYIRNNIPLIFCIALWISVNVILFVEAAMRHKDKGTFVLIARGCGQCLNFNPVIVLSLMMRKCMTWLRSSRVSGLLPLDQYIELHKLTGYAILFYSTLHFLAHLANFSSREIQGIETNFTMWMYLFGVGTDLGWVGGMAGLTGFLLLIIIAIMFICALPFIRRSGHFQVFYWTHNLYIAWYIILILHGPNFWKWFAVPAIIYIGEFVLRLKIIKLVRYGKTYIEEGILLPSKVVHLVITRPGNFNFYPGDYVFIQIPKVAKYEWHPFTISSAPEKQGVFWLHIRAVGTWTERLYKLFEAKNKRKSKGSVPLQLLTQPKIHHPVSNKVAPIAITATEDEEHPMAATDRHNGQNFGHMQYDPVVIQVQLDNENEEAIEVFFDGPYGSPSVHIFQAEHAVLIGAGIGVTPFASILQSIMIRYQNGRQVCPRCNHHWTGDIPPTIMTLKKVDFFWINRHQKYFEWFVSLLSQLEIEQMESGFNRFLDMHMYMTSALDKTDMKAIGLHMALDLLHKKEKRDLITGLKTRTQAGRPNWEEVFATINCEKKGKVTVFYCGLPSLGKILQKYCVKYGFGFRKENF*

>A.queenslandica_5_Duox Aqu2.1.39906_001 Aqu3.1.39906_001

MKLLLLLGTLVVLSAASNPNDPAVQMKLRDLGLNNSGFEPDGHYEYQSYDGWYNNAAHPEWGGADMPMERRTPIAYPDGVYEFAGRDRPNVLIIANLTQNGLTGHLSTTRTGFFIYFGQQVVEEVLDAQRQGCIPEYEFIEVPKCHALYDRDCRGDRVIPFLRNRYDFQTGYNPNNPRMQLNEITPWFDGGLMYGPFKAWTDAIRSFQGGELAANDNQANIADQFPQSNDELGLPYANPPPPANFSLFPVNRFWTIGNPRGNENPFLLTMGVLWFRVHNWWARRLRVFYIQNRMRYNLSHEEWENDEWLFNRARQFTIATHQKVVYKEWMPLFLPSKYNGNPNAFPYNNTPGYTLYPGVSGYNPGINPQVAHIFQSAAMRFGHTMVTPGIWRRLGGGNQTCEFGPTLSPDFARFSNAYLRYVTNNENVQFDTMTSLITRLCNSNNPYNNTDLNDTLCNTINPGINRDDPSAGSYRVGNLHESYFAPRTCNQFWNPTVTIMQTNVDSFYLGMASQHAEREDFIITPDLRGFVFGALDHTRRDLMAQNLQRGRDHGLSDYNSARIAYGLQPLTSFEDLNSEYGNNPDITDNIERLRDVYNNDISKCDIWACGLAETTEDNGPGTLFTEVLFDQFMRIRHADRFWYENYKNNHLFTEEEINMIESVDMKSILIAITNSINADNIQDNPFQVTTNNQYCPQPFQLSELFMDDCRPLENFDYYDSSVWQVPFIWALVFIYIFIIIGLMLLIAELNKRRRAKILSSGRKSRTKKIEGTDLTDNDGEVIVAYEEKAGLQGVTRYVSFKLGPEKQLKLFYGEELLRTVDLRNQTSINILLPVDSELYFAVKIKNEYDILIRCKTVHDRNAIINKLKEFLEGLQIEVEEERGMKRVMLRNIFTKKQRQQLLENFFKSVFAEVGSKQNVETRTDILECELTKDEFADAMSTSKDSLFVEQMFALCDADQNGFISFREFADMTVIFSKGSPDEKLELMFRMYDLSGEGQLDRDEFKKMLKSMMELVNASVTGDQMNSLVDSMFTAAGFENKQHLTVDDFKVLMRDHKEELSNAKISVQGIEAPEIEAPASKEEEESGAVPSRYKARENATARARRTIIKAYGRTTKQDPRAQQQLEESQVTLQIKKKSKFTSTPVGRRFAAFLRWVENYKLHIFYLSMFFLITAAIFVERAYYYSVEREFGGLRRIAGYGVTVTRGAASCMMWTYSVLLITMARNLFTYLRETIFNYYIPFDSHISFHKVVAMTALFTTVMHCIGHGINFYHIATQTPSDLTCLFREVYFRSHFLPKFSYWLFLTMTGFSAFVLTLITVIIFVFAVQYARRYAFQSFWLTHHLYIVFYILMFLHGSGRLVQDPLFGNFFLGPGIVYVIDRLVSLGRSKAEVSIVRADILPSQVIGIYFKRPPSFDYVAGQWVRIASLAQNPGEYHPFTLSSAPNEENLSLHIRAVGPWTHNFREICSQKKSAGDPLPKLFVDGPFGEGHQDWYRFEAAVLVGGGIGVTPFASILKELVHRFNIGARIQCKKVYFIWVTRTQHQFEWMADIIKEVEEADTKRLVEVHVFVTQFFDKFDLRTSMLYVAERHFQRLSGRSLFTGMRAITHFGRPDFGTFFDSLAEEHNLLPKIGVFSCGPPGMTNGVEEACAATNRYEGPAFIHHFENF*

>A.queenslandica_3_NOX5b Aqu2.1.17524_001 Aqu3.1.17524_001

MAVYVNHTALNISDSSLSSSAEPIVVSTSKFRFTGSEVIKESDTVITVDENEELSPGTSVKTTGEILEWIRRRLEGHLVVTGRGYVTLGDLKSAVDTDLPGFAYHLFQLIDVHQSGSISQQELIGGLSRLTVKETKHKILSWFETLFIETAGEDQLLQPDEFKKALETDGFLDKLFLLMNVSGSGKMTVPQMLDSLSKLSCVDKDANWLLWFETQFISIAGDDRQIDFEGFRKALHLSQSSFFASRFFSIFDKDGNGSISLNEMINGVTLLMSGTQLDKLKFLFQVYDVDGDGSIDYDELKIILRSCTSESSLRINEENLDTLTNALFDFADADKNGSISFEELKNVFDKYPDIIDNLTISASNWLKPQKFSQNKKLSERLWPSWLSWNRIRNDLSYFIFLSVFFMINIVLFVEAAIRYRKSMFLISIARGAGACLNFTPVVVLILMYRHIATLIRSTRLSFLFPLDKFIKLHKVVGYTIIALSLVHFVAHLANFSYIEIDDSIYSNYSMVHYLFGVGTIGWVYGTAGLTGFLLLIILIIMCVCSLPVVRRKGKFEIFYWSHCLYIMWYIVLILHGPHFWKWFIGPAIVFIIEKIFRSKLFQIIHYGRTYIEEVNLLPSKVTHLSITRPPNFNYQPGDYVFIQIPSITRYEWHPFTISSAPEMSDVFWLHVRGVGSWTNGLYEHYNNTYNDESTTGTAPSSPTATGNILEEGHEDILLLYCVHYNVQCFKGVRDILEFSCPPH*

>A.queenslandica_1NOX5b2 Aqu2.1.21928_001 Aqu3.1.21928_001

MAVYVNHTALSVSDSSLSSTSEPTVVSTSKFRFTGSEVIKESDTVITVDENEDLSPGTSVKTTGEILEWIRRRLEGQLVATGREYVTLGDLKSAVHTDLPGFAYHLFQLIDVHQSGSISQQELIGGLSRLTVKDKKNKILSWFETLFIETAGEDQLLQPDDFKKALETNGFLDKLFLLMNISGSRKMKIPQIIDALSKLSCVDKDANWLLWFETQFLSIAGDDRQIDFEGFRKALHLSQSSFFASRFFSIFDKDGNGFISLNEMINGVTLLMNGTQLDKLKFLFQVYDVDGDGSIDYDELRIMLKSCVSESSLSIDEENLDTLTNTFFDFADADNNGSISFEELKNVFDKYPDIIDNLTISSANWLKPQKLRQDKKPFEKFWPFWLSWKHIKNYWSYFIFLSVFFMINTVLFVEAAIRYRESMFLVSIARGAGACLNFSPVVVLILMYRHIATLIRSTRLSFLFPLDKFIKLHKIVGYTIIVLSLVHFLAHLANFSFIEINDSINSNSSMVQYLFGVGTIGWVYGSAGLTGLLLLIILIIMFICSLPVVRRKGKFEVFYWSHCLYIIWYIVLILHGPHFWKWFVGPAIVFIIEKIFRSKLFHIIHYGRTFIEEVNLLPSKVTHLSITRPPNFNYQPGDYVFIQIPSITRYEWHPFTISSAPEMSDVFWLHIRGVGSWTNELYEHFNNAYNDKYTTGTPTFTGNTVDSLPRRLGTLVHRRFSFKGREKIQSQDERQPSTHTIRKYGGKYTCKSFFCDSRFEVHIDGPYGTPSSAIFQSEHAVLISSGIGVTPFASILQSIMNRFKLSKQKCPHCSHSWTGEIPSTILNLKKVDFIWINRDQRHFEWFMELLNELEMEQNEYGTLMDRFFDMHMYITSALQRTDVKALGLQMALDLIHKEKNIDLITGLKTRTQTGRPNWDKIFKELKENGYGPVTVFYCGSPVLARMIDVSQWRASIGLWNYCQAASSRPANGHHSHSFEAAVDSKSGSTTSGEKTSKLPAALSLIVSLLLSLLRYVKLNFIPPTATIGSSSDLIYYLVLLLLLLLSGDVELNPGPMIDDQPDIFLLLQWLEPLVDWQSFGLLLPGITQDEITIIEQVDTKYQKLALFTKWLNTDPTATWRDVLNALTKREEIILLQTITDQLQVHQCTGGDTDAPTSTVPAVSTVLSSVSVINKPVTSTTSGNTPSAILRMNYAILVDAVTNNLYRVTNGLYAKGLIPMETINNIQTAASSDIVKSGQLMSVIQQQFESSLNPEQYLIDICHVLINQQHRILTDIATSVLHQLGQSIPDNVSSHTVLPSPVDDISNTPVTNIRGQSDVAKPISSIPDDVQGYADNMRQHYKHQPIVATDWPPRIGKDFFGRLALVEKQDSSTQAKSAWHMLRGQIDKTVKLTENKEISVEDVLQSTNSSLSLRVVIDGPPGIGKTTLCRKLLNIWSNGTLLHQQYDLVLYCPLRNSKIATATTLADLFVRQRYEVPMVAEWFEKRNGEGLLIIFDGWDELSEQLRQSSLAASIICKEKLDQCSVIVTSRSYASSSLLKMDTLSRHVQVIGFFEKEISTVIIQTLQKDTKLAQELIHEKEQHDNFIFTKSHFTTTQSSKDSQLAVKLINDLKVRNDVQSLCYVPLVCSMVILVYCKEGGHLPTTLTQLYENFILQTIRRHVKRHDINPHTLGSLSSLPSQLAKPLQEMCQLAYTNLANTRMTFSSHQLQSLSEAVKEDYLGLMTRFTEYDEEKYQFLHLSIQEFLTAWWIAKHEKKTEEVFKDHFDDDHFQMCLRFVAGLTHLEHESYQQYFNKQQLDLQCKRKPLFGFETCYTFYQNPEIRTMHDVKSDHIRSDDFDSVPILLLQLLYESQNTTLCQVLAQSINNHSLCLYRDRVSLSLFDWLCLSYCINNSNTTWNHLHLGTLSNQSLSVFTAGLTNNSLQTQCKRLEILHLMMYRLTIPTVDNTYYTDKCTELEKCIEINSTLQEMKIEYRGKNEITSTIISVIRGVTRNKTITSLAMHVLARPPPQPDGVIEQLLKHNNTLQALSLAIHDKLLPSSLNIVEVNTPLTALEIGKWSSELMTSSLLPHIKGLHCLILHDDPYPPHLLFLSHPSLHTLTLPLDSAESAIKLFTILQTNTTLKALSVEIKEERVYTSSMGTSLQDMLTQNQTLKYLEIHNSYKDTIPSSFLSFLTTGLRHNTNLQQLSISIPLNEEIRTFINVISQKNNFTELKVNFELDQSYSNCSKEEKEQIMTPLFYEQVLPAVTNMLQSHTTIRLLRIECRGINEESSQPNWIEL

>X.bergquistia_3_Duox Xb.00539.mrna

MQSLVAVVLACLLGLSVAAVLNDPAVIQLNDMFETEGHYEWESYDGWYNNPAHPEWGGADMPMERKTPIAYPDGVYEFAGRDRPNVLIIANLSQNGLTGQGSDLRTGFFIYFGQQVVEEVLDAQRAGCTPEYEFIKVPKCHPLYDPDCRSERFIPFLRNRYDFNTGYAPNNPRMQLNEITPWFDGGLMYGPFKAWTDAIRAFVRGELAANNNNLDIADQFPQSNDQLGLPFANPPPPFNNTLYPVNRFWTIGNPRGNENPFLLTMGILWFRVHNWWARRLYRFYYNNRNNASFHFDEEWGMDEWLFNRARQFTIATHQKVVYKEWLPLFLSSKFNGNASAFPYKETRGYSLYFGRSGYNPAINPQIAHIFQSAAMRFGHTMVTPGIWRRLGGGDPDGASCQFGSTLSPDAAQYTNRYLRSLTNDSARFPTFNSIYDEFCQQKYIGTEIYQLICGNSTYTNIGRNGYQPGDLGSQYFAPRTCNQFWNPQVTISETNVDSFYLGMASQRAEREDFIITPDLRGFVFGALDFTRRDLMAQNLQRARDHGLPDFNSARIAYGLPPVYSFEDLNENYGVDVAITENIERLRDVYNNNITQCDIWACGLAETTTEGGPGTLFTEVLFDQFMRIRHGDRFWYENYENNGLFTEEEFNMIESIDIREILIAITTSDVNDTNLQPNPFMVSNESMFCPQPFQLTELVMDDCTLVKNFDYYDSSIWQVPLIWALVFIYVFLVIFIMLLIAAVNKRQRAKILGAGRQTRVKKIDGDDLTDNDDQVLIAWEEKAGLNETTRYVSFKLGPGKQIKLFYVEELLRTIDLRNHTSVTIQVAVDSEQYFSLKILNEYDVLIRAKTIHDREVFITKITEHLQSVEINVVNEQLRKKDIIRNTFTKKARQVLLENFFKSVFAEVGGNQRYDTRQNVLDCELTQDEFAEALSMKKESLFVEQMFQLCDSDQNGFISFREFADMTVIFSKGSPDEKLDLMFRMYDVSGTGQLERESFKRMLKSMMEMVNASVTGEQMDQLVSSMFTAAGFENKQFLTVEDFRVLMRDHKEELSNAKLNVQGVEGPSLEAAPVSKEEESGAVPSRYRSRETATARARRTVVRAYGRTTKQDPRADRGFEDSQVTVQTTRRKAFYSTPIGRKIAVFLRWIENYKLHIFYLTMFFLIVFCIFIERAYYYSIEREQAGLRRIAGYGVTVTRGAASSMMFTYSVLLITMARNFFTYLRETIFNYYIPFDSHISFHKIVALTALAFTVMHGIGHGINFYHIATQTSNDLTCIFREVYFRSHFLPKFSYWLFLTMTGFSAFILSLVTVVIFVFAVQYARRYAFQAFWFTHHLYIVFYILMFLHGSGRLVQDPLFGNFFLGPGIVFVLDRLISLGRSKAEVSVVRADILPSQVIGIYFKRPPSFDYVAGQWVRIASLGQNPGEYHPFTLSSAPNEENLSLHIRAVGPWTHNFREIFSQKKSAGMPYPKLYVDGPFGEGHQDWYRFEAAVLVGGGIGVTPFASILKELVHRFNIGARVQCKKVYFIWVTRTQSQFEWMADIIKEVEEADTKQLVEVHIFVTQFYDKFDLRTAMLYIAERHFQRLSGRSLFTGMRAVTHFGRPDFNTFFDSLAVEHSLLPKIGVFSCGPPGMTNGVEEACAASNRYEGPAFIHHFENF*

>X.bergquistia_2_NOX5c DspNOX Xb.13305.mrna

MSLKTALLLTSVAVLICSILASPLEFCTNSEYILVGEHLNWSINVTELMCTIDGRQLNDGNVLFSTPGSYQYNCHNISGNLSVVAIQHKLAFCSNCTPNYLVVFTGQAVSWTGPDWECSLQLVNNSCHGNVLRDFSSHTLGLQQVHHFQEPGTYQFLCHDNCEPLATVTVVNVTDTHLVEFCSQPQPSIVDFKYKELIYSHGAIMAVTFGILFPLGAFLANIKKPIAHMILQPLGIVLAVIGFVLATVYKGLNSSSHFNQLHSIVGLLLLAVVIIIIPGLKLSLLTPIKEKWQKLVIIWHKRLGISAVFFGFSNIFLGLLVLSDTPVYIKIIYGVWLLICIVVFVISGSVAKQDVYDDDEAVSVMVTCCRYRADKVTLNRQDPIRLSKLSKDVLSEGKYIQARKKSMDMLEMAVEPVKTVPQRKISNVHFGPIDIAANLTPDIVTPQSSNQFGFTNRLSSEELSDNSESEEEQSTDEVDADINDKKNVRDSQFSINSIPSDGGSLRITRHSDRAHTKVTDVFARSNSTSQHPSPLMTSQHVKVDDNNDSDNEKHKKEGSSSGEDITKCPFHRSAKSDSDLPLQQRRMRKNALTVPENQSPMTLPGQQKIQRQISRNSSFRRQQHKKLIDNTLQSVMEISDDAIICANAQGEIVFWSAGAMKMFGYTPGEAVGSSLQMIMPERYKEKHQNGVNARTERALDYIRKHHTAPEEQKHARLYAVHGVSKTGKEFGIEAVVTSFVIEEDIFYTGIISEVKPLTRQQSSCSLSSETEIREKHQKALAIGAQMKMVHKLRNADYMTKRELTRILENSLQAAEFEVPPEDITVMVDTISKAADKDETGVIKFVDLIDLLAKHQLYISDSGLIAKYGKAKASEGNLKPRQRCCNKVFTFLFTHIITLSWILAYISLNIIMLIIGVLTEKRKGWSKFSYGTGPVLSMNCVLILLPMMYSVIHIFRGSSFLTKILPLKQSFLFHIIIAMGIIFWSAIHVTTHLCSFSFDDVNENGTYHDNFTANIKTHLMPLITGICAIFIIIILVVSSIKPLRSLCQFIGFYSLHWIGITLFYILLIFHGTNYYNPSFWKWLLPVLFVYLLERLYHCCVATKYSVVLQKAAPYDEMSRTTKIEVQKPKHYKFILGQYLLINIPQIGYFNWQPCKITSTNKEKMLTLYIPHTGNVWIEEVFKTFKSNPIKPTTDDQIIPALTVNITKPYGVGADVVFDYKVAVLVCGGDGPLTCRSIIQTFLEYRKGSSCHCGCHPKLTKLYFVWVTPSGNDYEWFTDYLSELTEDENLKKYLDIRIFLTNINSPPDELASALLHLALKTGGPALSVRRPSQPQLSKLYAYTSYGIPDFENLLDFVSMQQGTASDQGMECGVFTVGDLPRTVKKDLLHYCTICSNKKGLPVTNTPVDSPYKQPVDSSELEQVPREWHITFTQQTLIL*

>X.bergquistia_1_NOX2 Xb.23945.mrna

MNEASELIYAEKGTEKLERTARIAMLQFITHAQYYLNDCIGHGTWFLLNIALFIFQFHQFQTIPGHYYIRFYIREALAFARGPALPINFNVILILLPVCRNIISVIRGCTRVVPRSIRRLLDKNLTFHKAIAWMLVVSSAMHIMAHYYNYERLVRYSPPNTLPPGEQANPPFDALPRRGDVGKLNPIQACFVTYAGVTGHIITLALFLMVTSSLEFIRRSYFEVFWYTHHLFLIFLIGLGIHQYGRLLPVFRSVNLLEPKGVGGRPGLGHCAYSKDSEYTFVNASGLNQTVNCNTNLSFIPAGPTSWMYLLCGLLIYGLERILRVVRSFYSVVIVKVVQHPSNTIEIQMRRKGFHAEAGQYVFLNCPSVAWFEWHPFTLTSAPEEDYFSVHIRIVGDWTRGLAEILGVDRDDFQQSWELPSMYVDGPFGTASEDVFDHSVGLLVGAGIGVTPFASVLKSLYYRLSDDSAGLVLKKLYFVWICPETHSFEWFANLFQELEQQLHERGIDDFLTSWIYLTRGWRDNQAFYLMLNEGRVTSEDASEDMVDAITGLRANTTFGRPEWDKIFEKLSNDHPNTDVGVFFCGPSVLSHNLHGYCNTFTKKGGSTGARFYYNKENF*

>T.wilhelma_1_NOX5c DspNOX Twilhelma_g1524.t2

MLTPSTPHTPVDAGGDNLALDTPGIYHYACGNSTEPSSVYIVPAIYSISLCRECTNRDVQYKINTGEAIKWSLVGEPAENCTVLIKMDKNQEANYSEEFLLDFRGETVLRKPEGHTAFYFPVAGLYELFCKEEDNSSNILATILVGNVSQSQLNETTKESDQKYIELLYTHVAIMGVVFGVLFPIGAFLAYHQITLVHKFTQPIGLALALIGFVMVIVYVQLSHGNHFRYLIHGVIGLALLIMVLLVMPLLLISERSRKYHHKFGHIIAFFGMGNILLGLAILPASRAVALTLCILYGLWLVVSLSFYVFFPGNKKKEECKESNKKDHISSRNGFYKVNSQNSFVSNIQRGTSNGSLSSGRGPQPLRKNIISEDKMIELRKKSLDFLEASRHRRQLDRELGLTSSRPLIENQTSMTDVDLEEEEEDINVQFKVGGIRRDSKPDQDTPPETDKEPQQLSSPEPDSPFTPPPMRSQMSATPDSDDEDVEDDDALESPNYNKFKLKSPPMSSSLSRAHTFNYGDTAVGAHMIHDSDDNSSEKGGSLRITRHSKRARGRVDNLFSVPPTSLTISATAQGTSSYSVTEETPEPDNEGPLSESYPPPPSRPQHMMVTVSPPDPTSNGDVQQTVPTKAAMPAHGGRRRKAALVPPLITTTPVVDDDTPPPSGCPFHSHSSPNFGTLSTSDNKRNTSLSPRPIRRGVAQSPADDDTDGSSPRPRRKKVSVAQIEATLFPPTPVNLHPPQHLGLSRSSSFNTMRTHHDVFADNAFRSVMQISQDAIVCANSIGDIVYWSKGACKMFGYTPGEAIGSSLEMVIPQSYKERHRVGITQRTTDTLKYVRKHSYTPEEQKHARLYAVHGMTKSGQEIPVEVNVTSFVIDGEIFYTGVISELARHSGVNRSRANSVSSGSDVSETSTKQRALAVGAQMKIAHQLESAEQISRKELERVLTGSLQASSFEVDAHQMEQMMAGIMKAGDPDGDGAISFVNLIQLLSQHQLYISESGLIARYGKTKGALGSNLTRMQKQAKRTKQFFTNNVASLSWIGFYVLLNVFLAVLGVLSTSQDGWKRFAYGTGPVLSMNCILILVPSLTSFIHAMRSSYWMNKVFPLTKTVTLHVIIVLTIFLWTGVHILTHFVSFALDDKNTSMTNSTSEHFKMNIVTHLCPVITGSVILGIFAIMGLTSIKAISKLGRFVPFHLIHWIGTVLFYSLLVIHGIRYVNPSFWKWLIPAAIILALERIYWFVFAGKQKVAIKSAGRYDDQSRTGIIELEKPKGFKFEPGQFVLLNLPWIGYFDWQPCHISSGPKDNKLTVYVPHSNDAWFSQAFKKLKENPYKMAKQVNPNAKESKRGEQLPTTEENHPIVQFASHIKGPYGPRDNSVMLFDYTISALVCGGGDGPHAAKAVVKRLLEYRKVAACHCGCQPVLTKLYLVWVIPSGNDFEWFTDYLSDLCEDPHLHRYMDIRVFLTNIDSPPDELASALLQLGLKTAGDSCKVRRPSLPQVNNLYQKSSYGAPDFPALLDYISSQHSSKWNKGDGEVEFGVFTVGDVSQSARTELTRACKTSSKPGKLSYKPCHIIT

>T.wilhelma_3_NOX5a Twilhelma_g10073.t1

MEIIPHAKLIKVRGSLDEDQDEQRAEPESNNARRAITLGMLANTTRKARNLLERQRQAHRGTVTSPRSPPAVQPQAVRRTPQTSHSANHGCCGTPFHPDHSEDVDHLNVFQNESCSLELEEDDEVELQLRLGNDRTPPEPEDNLLEQSTRRKRNGLVHQAHHQEIECISAADEDRDGAERDEDHLHSSTTVAEEDEEEGGGERSSGSPHQHHGAADGVDISAEEEVIVSDEEEIALILEWFRVTFTKIGKHGRVTLRDLKYAVKENEEFAEQLFKLFDTDHSNSVSLQELVGGMGRLTTHNRKVKILKWFEQVFDRVSGDDQLLQLDEFKRALQISGFMERFYQLVDTNGKLTLPELLDALGKIACMDRDAKWLAWFEQQFSIVAGNNRQIDLEEFKKALHVKESFFAERFFQLFDRDNSGYISLRELMDGLTLLTNGSEVDKLRFLFQVYDVDGNGHIDYNELRTVLKSCVGESTLQFSEDKLDELTHALFEDADVDNSGTISFEELKAELDKHPGVIENLTISAANWLKPPQSQRRQTLHNLLPHWATWKYVRNNLTWVIWLILYIGVNTILFVEAAVRHRSKGPAVAIARGCGQCLNFNPTFVIIVMMRRGLTWLRSTRVAFLFPLDQHIELHKMCGWVIFIFAIIHTMAHIVNFTLLSLTEEGLPLWKYFFWVKTGFGWVGGTAGITGDILILIITIMVLCSLPCVRRKGYFEVFYWTHFLFIVFYILLILHATHFWKWFLVPGIIYILERILRSKWVKLARFGRTYIQEGILLPSKVTHLVMTRPTNFSYQPGDYVFLQVPSIAKYEWHPFTISSAPEQQGFIWLHIRSVGTWTNKLYEFFDKRNKSRKRETIQMQLPRDQQNHNMEQLEQVVRTWGDSQSCDFDEPLNDVHPLAVTGGFGDPARLDGGSNVTHGQDFSHLNAPPTIINVQVDNEEDRGIEVYIDGPYGTPSAHIFQAEHAVLIGAGIGVTPFASILQSIMMRYRNARQVCPRCNHGWTGDIPLSVMRLKKVDFYWINRDQRSFEWFVSLLSQLEIEQSEQCGFERFLDMHMYMTSALRKTDVKAIGLQMALDLIHKKEKRDLITGLRTRTQAGRPDWNKVFRQLDNDRKGKVTIFFCGPPAISNILKTKCSQYGFEFHKENF

>T.wilhelma_2_NOX5a Twilhelma_g10073.t2

MEIIPHAKLIKVRGSLDEDQDEQRAEPESNNARRAITLGMLANTTRKARNLLERQRQAHRGTVTSPRSPPAVQPQAVRRTPQTSHSANHGCCGTPFHPDHSEDVDHLNVFQNESCSLELEEDDEVELQLRLGNDRTPPEPEDNLLEQSTRRKRNGLVHQAHHQEIECISAADEDRDGAERDEDHLHSSTTVAEEDEEEGGGERSSGSPHQHHGAADGVDISAEEEVIVSDEEEIALILEWFRVTFTKIGKHGRVTLRDLKYAVKENEEFAEQLFKLFDTDHSNSVSLQELVGGMGRLTTHNRKVKILKWFEQVFDRVSGDDQLLQLDEFKRALQISGFMERFYQLVDTNGKLTLPELLDALGKIACMDRDAKWLAWFEQQFSIVAGNNRQIDLEEFKKALHVKESFFAERFFQLFDRDNSGYISLRELMDGLTLLTNGSEVDKLRFLFQVYDVDGNGHIDYNELRTVLKSCVGESTLQFSEDKLDELTHALFEDADVDNSGTISFEELKAELDKHPGVIENLTISAANWLKPPQSQRRQTLHNLLPHWATWKYVRNNLTWVIWLILYIGVNTILFVEAAVRHRSKGPAVAIARGCGQCLNFNPTFVIIVMMRRGLTWLRSTRVAFLFPLDQHIELHKMCGWVIFIFAIIHTMAHIVNFKGLPLWKYFFWVKTGFGWVGGTAGITGDILILIITIMVLCSLPCVRRKGYFEVFYWTHFLFIVFYILLILHATHFWKWFLVPGIIYILERILRSKWVKLARFGRTYIQEGILLPSKVTHLVMTRPTNFSYQPGDYVFLQVPSIAKYEWHPFTISSAPEQQGFIWLHIRSVGTWTNKLYEFFDKRNKSRKRETIQMQLPRDQQNHNMEQLEQVVRTWGDSQSCDFDEPLNDVHPLAVTGGFGDPARLDGGSNVTHGQDFSHLNAPPTIINVQVDNEEDRGIEVYIDGPYGTPSAHIFQAEHAVLIGAGIGVTPFASILQSIMMRYRNARQVCPRCNHGWTGDIPLSVMRLKKVDFYWINRDQRSFEWFVSLLSQLEIEQSEQCGFERFLDMHMYMTSALRKTDVKAIGLQMALDLIHKKEKRDLITGLRTRTQAGRPDWNKVFRQLDNDRKGKVTIFFCGPPAISNILKTKCSQYGFEFHKENF

>T.wilhelma_4_Duox Twilhelma_g23520.t2

MTVKACILLLACAALAAAQSSGRNLGPTYVLETFIQVNNSQFRDPLRFSGFSNRTHFEWENYDGWFNNPAHVEWGGADLPMERKTPIAYPDGTYEIVSEASRGNVLVIANLTQHGLSGLGSSRRTAFFTFFGFAAFMVTLVTVVIFVFATQYARRYTYRAFWVTHHWYVIFYILSFLHGSGRLVQDPLFGNFFLGPGIVYALDLIISVGRRTQELAVVRADILPSNVIGIYFKRPVTFDYKAGQWVKIASAAQNPGEFHSFTISSAPHEDLLSLHIRAVGPWTYNFRENYNPANLRGQQYPKVFIDGPFGEGHQDWFRYEVAVLVGGGIGVTPFASILKELVHRFSIGARIECKKSLDLILFPSKQVFFIWVARSQKQYEWFSDIIRDVEDADKKNNVEIHIFVTQFFDKFDLRTTMLYVCERHFQKLSGKSLFTGLRAVTHFGRPDFNQFFDSLQEEYILLPKIGVFSCGPPGMTNGVEDACAATNRFEGPAFIHHYENF

>E.muelleri_4_NOX5c DspNOX Em0019g755a

MAGGKCPAVVTVIALLISLQNGAADDEPFGFTCPVGSQVILGQHLVSTAMQNCTFSRTDGANGQGYQANYTELLDVDSVEYALNTTGRYSYTCGNHSGNISVVLSQYHIALCQGCERHVHTIVNGTAVAWTVANEPCGMVTITSEKGVVNVSDASYTFDSNGDFLLQCSKIPATRAAIRVVSSCSSSSSSSNSNSNSSQAQSTASYLPLLYAHAAAMILTFGVLLPVGAFMAHNRKHLVHKLMQPATTVLGLAGLALVVAYVQMTSRAHFRQTVHAVVGVVVLRLDVGHSPAARARAMEAMASGGLAIWPAPKVAAITVGVLYGAWLLAIAAIYVLVVVGGVCKERSEHSFKTNGTDMEAIAPAPSRPRTNSILKPLSKGVISEAKMVQLRKKSIDYLEAVHQCNKLNKELGLTTPHPVAMDADQSTLTAETDLSRTEEEPKRENSNGSLTPPPQHTLPPSHIQQQPFLSPEPQTPPPTQEDKSLNMIISTSDEEGEGLSEKEEVPDLELDRFNRTSSPRPVKKAHSFNVPPRHLDLDLESHGDSLRVTRHSVKPKTPPKPPMDELSVEIPTGKPGNLNARRRRPALVPPIITTTPAEESDCVFTKQVSLDSDVGRDLSPCPSTSSLQNESSKPRKKKVSVAPLEAIPIPPTPVALRPPSMSRSASFAQTNVFSDNALRSVMQMSHDAIVCANAAGDIVFWSAGAVKMFGYTPGEAIGSSLEVHGLSKNGHEFPVEVNVTSFVIDGELFYTGVISELVPKNAQGRSRGTSVSTMGSENHECTMKQKALAVGAQMKMAHKLKSAEHITHKELENILATSLAASKIEVQLLCLIQPRQLEEMITGIMKAGDPGNTGVVTFMNLLPLLYQHRLYISESGLIVRYGKTKSNRGGTTRHQRTLSQVKNFFHLNASFLLWFGVYLAANAALVMGGMLSNGRSGWERWAYGTGPVLSMSCVLVLLPTMSSLIQAMSSAQWMAKLLPLHRSVHFHLAMAVGVAFWSAVHVTLHLCSFALDTHNTTTGAGGKEEESRSDYFMSSLVTHLCPAVTGLMILVALGLMCVSALPVIRKRMRFIAFYMIHWMTTILVYVLILIHGDWYWNPSFWKWLLPLVFAMGFELVHRHWITPKYTVAVKTAGPYDDISRVTVIETEKPKQFDFIPGQFVMLNFQKIDYFGWHRCYISSGPKEPKLTIYVPKEGNVWFTEMFLTMKAHPFNRVISQSNSTSEEKKASPPTECLSEQLTLQFSGPYGPGSNSVFDYKISALVCAGDGPHASKSLLSKLLQYRKHNSCLCGCHPILTKLYFVWVIPSGNDFEWFTDYLSDLCLDEHLQRYLDIRVFLTNIDSPPDELASALLQLGLKTAGESCIGRRPSQPQLNNLYSMTSYGIPDFDGLLEYIYTQHSNLWSEGSDQMEIGVFTLGDIHKTMLDDLNKACRTATRPGQVLFKQSRIAS

>E.muelleri_7_Duox Em0023g489a

MDSRTVLLLLGTAALVYAKSFECGSSSVCDGVVSTVYEKFKSTVVNNTFKVYNSTHYEWLSYDGWYHNIGHPDWGGADMPMERKEPAAYGDGVYEIAGKDRPVTVLISNMTQFGFTGNGSRTRTGFFIFFGQQVVEEMLDVQLPGCIPEYENMPVPKCHPLFDPNCQSERFIPFLRSRFDFRTGYSPSNPRQQLNEITPWFDGTLVYGPFKGWADALRAYTRGELAANIQDAPLYLQVPAVNTIGLPYANAPPPANHTLLSVNRFWTIGNPRGNENPFLQSMQILWFRVHNMWARYLVKYAKNNNLGEEWLDDEFIFNRARHFTIATYQHIVYDEWMPVFLRSKLLRLNRTTIPEYESNPGYSLYYGRTGYNPSINPQIAHAFQTSAMRFGHTMVTPGIWRRVLRTSPTDSCLFGSTATKPNSYLNMVGLKPQGSRTTVPGDTGDLNQYPMTQIASYLRANSLNQNSGAHQDFLSFLYNDSHNINPVPPDPAVHLYNMEVNPNQPNFTQKYTYGDAPAQYFSVRTCNSYFNPQGPIIATNVDPLFLGMASQRCEREDFIITPDLRGNVFGPLDFSRRDLMAINMQRARDHGLPDYNTIRAAFGLPRLSGFEQLNPYYGVRPDVTENIERLREVYNNDVNKCDMWTCSILETTDNGPGTLFSEVLFDQFMRIRHGDRFWYENWKENKQFSEDEFYAIKNMTLKKILLLITNISDFDISDTPLQFNENTVCPQPFQLTEFFMDDCSALQSRDYYADSGSDWMVPFIWGLVFIYIAIVILVMFLMASYNKHSRIALQAALQPKYLMKIDGEDIMTGVGQVIIATEEKADLKSTNRPISFKVGPGKRIVLHAGGVILRTIDLRNQTNIVIQRSNDNPLYLVIKIANEYDIIIRCQIPLDRTALQTKLTNYCSLVGIGVTIEEHPKSVMLRTAFTKKDRQYLLETFFRAVFQQGGQEGNASIQPQILECELTRSEFADAMGLKEDSLFVEQMFQLIDKDGNGFISFREFLDMSVIFSKGSPDDKLKLMFDMYDANKSGFLHRDQFKKMLKSMMELVNATVSAEQMDSLIDSMFVAAGFQSKQELTLGDFNLMLRDHKEELSGAHLNITGFDVEIPEVEGAKKDLSGASSGTLPSRLRQKETAPARARRTIIRAYSKKRSHKQPQGELPEGNDVNENITSIPTEHHTVTNSRIVQQFYVLKRYIENNRLNIFYLSLFFLISAGIFVERAYYYSTETEHGGLRRIAGYGVTITRGAASGMMWTYSILLITMSRNFITYLRETPFNYYVPFDSYITFHKIVAFTGLLFTITHCIGHGLNFYHISTETASDLSCIFREVYFRTHYLPSFLYWIFLTMTGFSAILLTLITIIIFVFATQYARRYAFQAFWFTHHWYVLFYVFMFLHGSGRLVQDPLFGNFFLGPVCVYTLDKIIGVSRKKASVSVTRAEMLPSGVVALHMKRPTGFEFKAGQWVRVACSKLNPEEYHPFTISSAPHEEEMTMHVRAIGPWTHNIREVYDPVNLQGAPYPKLSIDGPFGEGHQDWYQLMLPSWLELGLASLHFHPSSRTLFTNTTLGHEFNARRCTSCGSPVPRSSMSGSPM

>E.muelleri_2_Duox Em0023g490a

MDWKVPALILLCALSCNARVYDCQTASACQTLALDVFQRFQQSGVTLQPEANTQFEWEGYDGWFNNFANPEWGGEDMALLRQEPAAYSDGVYAIAGNDRPDPILISNMTQSGFSGNGSRTRTALFIYFGQQIMEEMLDVQIPGCIPEYENMLIPKCHPLYDPDCRAQRTMPFQRSRYDTRTGSSPNTPREQLNVNTPWFDGALIYGPGKAWTNIIRAFTRGELAADQQDLQLANMFPVLNSKVLLPLSNMPPPANHSLFSSGRFWALGNIRGNENPFLLSLGLLWFRVHNMWARYLVSFANIRKGNWLDDEFIFNRARQFTIATYQHIVYDEWLPLYVQSKLLRLGRTTIFPYKETPHYSASYGRSGYNPSINPQVSNIFQSAAMRFGHTMVTPGVWRRTNRANPADKCLFGSTATARNAYFKQIALEPQGAPGGADPQQYPMSQVAGYLRDNALGLTSPHQAFLSFLFNDSRNINGSNPNLPNFTTNYTYGDAPAQFFSVRTCNSYWNPDVTIRASNLDPFFLGMSSQRTEREDFVVTQDLQGSVYGPLEFSRRDLMAINMQRAQILYDQFMRIRHGDRFWYENWEQNKLFSEKEFLAIKSLTMKKLISLVTNVQEFDISNAPFEFTKDTVCPQPFQLSEFLMDDCKPLKTRDYYVESGSNWMVPFIWGLVLVYIAIVILVMFIIAAYTKHRRAQIISAGRAKNPKAPKKLQGADIFTADGNLLVFWEEMSGLKSVPRLASLKLGPGKRIVLFTGETVLRTIDIRSHTCITIQEPVDNTVYLVIKIPNEYDIIVKTNTPIDRIAIVDRLKAFLSECGINLSIEEPRKNDMMANVFTMKERQRLVENFFKSVFSQAGTDGFSRTAGDIRPEVLECELTRKEFADAMSLKEDSLFVEQMFQLIDKDGNGFISFREFLDMSVIFSKGSPEDKLKLMFDMYDANKSGFLHRDQFKKMLKSMMELVNATVSSEQMDSLIDSMFVAAGFQSKQELTLGDFNVLLRDHKDELSGAYLGLAGVDVPDVETKKEVPTENGGRYRQRENAPSRARRTIIRAYNRTVRHPHHTDTHGLDNDTVHHIPTDSQSTRSKSGLARQFHVLKRFVENHRLHIFYLSIYAFITMGIFVERAYHYSIETEHGGLRRIAGFGVTITRGAASGMMWTYSVLLVTMSRNFITYLRETPLNNYVPFDSYVSFHKIVALTGLIFTLTHIVGHGINFYHISTQTANDLSCIFREVYFPGNYLPSFLYWLFLTTTGFSAFVLTLITIAIFVFATQYARRHTFQAFWLTHHWYILFYMFMLLHGTGRLVQDPIFGNFLLGPAILYTIDKIISVSRNKTEVGVVRAELLPSGVTGVFMKRPASFEYKAGQWMRISSAQLNPDEYHPFTISSAPHEEHLSVHVRAVGPWTHNLRELYSHSISHSLPLPKLRVDGPFGEGHQDWYQFDVSILVGGGIGITPFASILKDIVHRSSLGARFQCKKLYFIWVTRTQNQYEWFTDLIREVEEADSKGLVEVHIFITEFFSKFDLRTTMLYVCERHFQKLSGRSLFTGLRSVTHFGRPDFKAFFDNLQEEHIVLPKIGVFSCGPPGMTTTVNEACSATNRFEGPAFIHHFENF

>E.muelleri_6_Duox Em0023g493a

MGLKLMLLFLGLLALASAKSYTCDSQICENIVSLVYDTFRNISGVNITFNVSKETNYEWESYDGWYHNLAHPDWGGADMPLERKEPAAYGDGVYEIAGKDRPVTVLISNMTQFGFTGNGSMTRTGFFIFFGQQVVEDMLDVQLPGCIPEYENMPVPKCHPLFDPNCQSERYIPFLRSRYDFRTGYSPGNPRQQLNEITPWFDGTLIYGPAKGWADALRAYTRGELAANNQNYSLADQVPAMNTIGLPYANAPPPANHTLLSVNRFWTIGNPRGNENPFLQSMQILWFRVHNMWARYLVKYAKENNLGGQWLDDEFIFNRARQFTIATHQHIVYDEWMPIFLRSKLLRLNRATIPEYESNPGYSLYYGRTGYNPSINPQVAHAFQTAAMRFGHTMVTPGIWRRPLRNSSNDTCLFGSTATKQNSYLKLINETAKGAKTNSTDEAGDPWQYPMTQIAKYLTNNFNNASGTGQHQDFLSWLFNDSANININSEVAPGEGVYNMYKNPNQPSFNKNYTYGDAPAQYFSVRTCNSYFNPQAPIIATNVDPLFLGMASQRCEHEDFVITPDLRGNVFGPLDFSRRDLMAINMQRARDHGLPDYNTIRAAFGLPRLSGFEQLNPYYGVRADVTENIERLREVYNNDVNKCDMWTCGILETTDNGPGTLFSEVLFDQFMRIRHGDRFWYENWRENLMFTESEFYAIKNLTMKKILLLITNIKDFDISDHPFEFTNDTVCPQPFQLTEFFMDDCTALQGRDYYADIGSDWMVPFIWGLVFIYIAIVVLVMFVIASYNKHTRAKLFAHNDRKKGRNIAGDDVQNSRGEILLATEEKSGLKGTSRHVNIKLGPGKRIVLHLGGVVQRTVDLEKQKRMMIQTAVDDPLYCLLRLSHECDIILRCQEFADCPHLIEQLRAYCQRVGIEVNTESFEKAKYLLRGAFTKKHRQEQLEEFFKSIFQEQALASGAVDLEYELTRQEFAEAMSLKADSLFVEQMFQLIDADGNGFISFQEFLNMSVIFSKGSPEDKLKLMFDMYDANKSGFLHRDQFKKMLKSMMELVNATVSAEQMDNLVETMFVAAGFQNKQELTLGDFNLLLRDHKEELSNARMQLTTIDVPDIEVKKELPGAEATPTRYQKRETATARASRTVVRAYARTQAPRAKKSVTDELDSNDNVADIPTVKDKPTFKFGAISRRWNVFTRYVENMRLQIFYLSLFFLITAGIFVERGYYYSTETEQGGLRRIAGYGVTITRGAASGMMWTYSVLLITMSRNFITYLRETPFNYYVPFDSYIAFHKIVAFTALLFTITHCIGHGLNFYHISTETASDLSCIFREVYYRTHYLPSFLYWLFLTMTGFSAFLLTLIVIVIFVFATQYARRYAFQAFWFTHHWYVLFYIFMFLHGSGRLVQDPLFGNFFLGPVCVYTLDKIISVSRKKVSVSVTNADMLPSGVVALHMKRPTGFEFKAGQWVRVACSKLNPEEYHPFTISSAPHEEEMTMHIRAIGPWTHNIREVYDPANLQGAPYPKLSIDGPFGEGHQDWYQFDVAILVGAGIGVTPFSSILKDIVHKYNIGARIQCKKVYFLWVTRTQKQYEWFTDVIREVEEADTKGLVEMHIFITQLLNKFDLRTTMLYACERHFQKLSGRSLFTGLRSVTHFGRPDFKVFFDSLQEDHFTLPKVGVFSCGPPSLTNAVEDACSATNRFEGPAFVHHFENF

>E.muelleri_1_NOX2 Em0023g779a

MAALADTLSFTKSTACGPSGLRVQHLLDATEVPMQSSICSSLRSIVNLLATGSVSNVISKFLAGGNLTALTKDKPGSPPDIRPIAVGETRRCLVGKCLCQITKGKASDYFSPHQFGVACPSGAEKIVHGLRSCIEEHQNEQDFVVMKIDLRNAFNLVSRQALLDECSAHFPELLQWAAWCYGLFWSPMGTIMSESGVQQGDPLGPLIAPQHLVCRVVQSLHTNHMQMSIIEVFICRRLHMNTSMILICIWLCLLFCLVLQKVLSAIASDPNCFDLLFHAWYIDDGVIAGSKQAVVQALSIIQDLGPPLGLMINSSKCELYGDCDFQPFPSEMKKCNAFNFEILGAPIGDTIFCAKFIAEKRAGASKFLALLKGVGSLDSQVALVLLRQCGGSCRFVHIARCTPPSLASEGLHFFYIDVRQCFSDCLSIDLPNTAWQQAQLCLSRGGLGLRSLSQHSAAAYVASSAVSGSATNTSHHLLQSIDCFNSLVSPADVTSTDELLTSPKNQKELSSRIEHSQFQALFESSSLPNRARLLSVASTHAASWLSVVPSPGLNLHLESAEFQTAIKWWLEIDLFSGEKCPCCLTLSLDPLGHHALTCRHNGDVVSRHNRVRDVFFESCRQAGIGGQMEVGSGLGHDARRTRPADVLVPNWVLGKPAAFDITVTSPLTPITLHEASVTSGSTAQVAENRKHASNDAKCSELGWVCVPLAVEAYGCWGPEAQTNLSRLAARLAIRSNCCKSQATLALYGRLNLVLVRANARALLSRSMATGCLNYLTQQENRIPRYNAFVHKMVSGLVINELPKWIVFGSWICMNIALFIYQFYNFTVLPRYFYSRVVLREALSFARGPAIIINFNIILILLPVCRNLISLLRGTGRCVPRTVKRVLDKNLTFHKAVAWMIIAASAMHIMAHYYNYERIAAIVRVGPLVPTEGPRPPLQALPVASVPPGTPLDPIAVCLVTVAGLTGHVITVALFLMVTSSLEFMRRSYFEIFWYTHHLFVIFLLGLAFHQFQQLLPVQSNGEDAFTVGPTGNVTGTHPPSYCSLVSPIQCAAAKFKGAGPLSWKFMLGGLTIYIIERVIRFIRSLQRVVLIKVVQHPSKTIEFQMRKKGFHAEAGQYVFINVPSVALFEWHPFTLTSAPEEDYFSAHIRVVGDWTEEVARQFGSGGDGFKQSFELPIVAIDGPFGTASEDVFDHEVGMLVGAGIGVTPFASILKSVFYKLTTEKATALKKVYFYWICPEPQSFEWFASMLASVEEQLSQRGMSDFLDIHIYLSRGWKDKDAFAVYLREGENKDAVTGLQARTHYGRPDWSNIFDEISLSHPKTNIGVFFCGPPVLSHSLHEKCNEFTGRSDGNRARFFYNKENF

>O.carmela_3_NOX5a m.91408

SEFKKALHVKESFFAERFFHLFDKDGNGSISLQELMAGLGVLTSGSDTDKLHFLFQVYDVDGNGCIEPHELRVVLKSCMDESRLKFSDDKLDELTRALFEDADADGSGAISFEELKAELDKHPGVLENLTISAANWLRPPPPAPPKSFSSFLPHYFTWKYLRNNLRVVVAAIIFIIVNAGLFACNAYLYRKSGAPISIARGCGMCIDFNSVLMIVLMLRKFLTWLRGTRLAPFLPLDQHIEFHKIVGMLLAVYSAIHSAAHFVNLGIVVGRPNATHAYWEYLFTTKTSFGWVAGTSGISGLVLCVILAVMIICSQPCIRRKGYFEVFYWSHMLFVLWYLLLILHAQNFWKWFVVPGLLYSFERIMRSKWVKLVQYGRTYIKEGILLPSRVSHLVISRPPGFHFQPGDYIFIQIPEIAKYEWHPFTISSCPEQSDVIWLHIRSVGTWTNRLYHFFEKHNERHKKVSIRLNLPKEPLPQHRFSATVINAPWPPPGSTSPSLLSADLDDEPHPLSPQSTLSPPQILLSPPPLHTPYIERCSSPSPSAVSRGQDFSHMFSPPTIINVDVDPDDDETAKRVEIYMDGPYGTPSGHIFQAEHAVLIGAGIGVTPFASILQSIIMRYRQAKQRCPKCNHAFSGEIPSSVMRLKKVDFYWINRDQHSFEWFVSLLSQLEIEQMEQDGTFDRFLEMHMHMTSALKKTDMKAIGLQMALDLIHKKEKRDLITGLKTRTEAGRPDWQKVFEKINRDKRGRLTVFFCGSPALSSTLKSKCDQFGFEFRKENF*

>O.carmela_2_NOX2 m.124702

MPSWLFNEGPKWLFMFLWLGANIGLYFGFHYKFELGIRYYYTRKILGSALAVARGSAACLNFNCMLILIPVCRNLISMMRGSCRCTPRSIRRILDKNLTFHKLVAWAIVFFTALHTGSHMFNYLFYLSASTKEVAPGILLMSTVPDRLNFLSDTDTDGIKVMFTTVAGITGHVIIIVLFLMVTSSIEVMRRSYFEVFWYTHHLFVIFFGCLIAHGVQGLVRGQTNPATYNPLSFDNSTQDPKVCYAFHARDYRDLSDYNVSLLQTKEQQDLLGVYCADRNATVAAGGPQTWKWVVGPLFLYFLERIVRFYRSCQQVVVTKVVEHPSKVLEIQLKKRGFHAEAGQYVFVNAPEVRFFEWHPFTLTSSPEEDYFSVHIRLMGDWTEKMAKRCGMGVKGTEFKQTYEMPKILIDGPFGTPSEDIFKHEVGIGIGAGIGVTPFASILKSLWYQSLDPHTTMKLKKVYFFWICPDTGAFEWFQSLLQHLEEQMLERGTTDFLEYHIYLTRGWDPKMAREIYMKEDEEGDIITGLQQKTNYGRPHWDNIFAELGRRHPGVDMGSCPAYCTRNAVSDNCIQMLLALMELASSTIKKTFKNVLCGGVKCEVLSVLQSELLR*

>O.carmela_1_Duox m.1711

MRFVVVGFCRSSFLFSLLVFVVDGQSDPCSACKKPGGTRTLIENLGDESMWKTGRFFCPGAAITFLCSHNRVLQGPAALLRTQRGRCTLSGWVSLDGQRIDMTTVKCQETVNPDGGDSEAVVATVFTVRNDSVWPDNQTSPPTELARSTEGVKFNANGTETSVSTQETAVPSGTSDQGGVTAPLEDEEHEWDVEGYDGWYNNIAHPDWGAAEMPLTRKSPPAYSDGVYQLSGADRPNPLAVSERVFKGSTGERSYRNRTVLLTFFGQQVVEEVLDAQRPGCPPEYENIAIPSDHPYASDGFKNMPFLRSRYDMKTGYSPNVPREQLNEITPWIDGNLFYGTTRAWADALRSFSNGSLACEDTECLFPRENGLGLPMANPPPPRDHALKSAKRFNMLGNPRGNENPFVLTFGILWFREHNRHARRLTQMNPSWSDEKIFNRARQWVIAEHQKIVVYDWLPEFLGRDETESYRHYDSSVHPGIAHEFQSAAMRFGHTLVPPGVVLRHPDCRPMTPPGNDTVGVRTCNSYWIPQETLTAVGIEPVLLGMATQVTEREDNVVTEDLRGKVFGPLEFSRRDLMALNIQRGRDHGLPDYNTVRLTYNLPQIDTWTNINKESLDYMQEAIAAAKEVYDGDINKLDLWTGGLLETTTQGPGELFQAIISDQFLRIRNGDWYWFENERNKLFDAEEIEIIRNTTLKDIITGNTNIQPSDIQHNVFVNRFDDPSSPCHYDFQLNQQMMAPCTNLETFDYFDGSAGPYIGVLTSIGLFVVGLFCLLYLLSYVRRSARPQSTKPRRVPSETMSSSTDDIVHGVATVRVIELKSECGDRPVLIFIRTQPQKEIVLTNDNSKKLRSISLSVKEVELQQASNDVGLLLLRVPGTYDVLVRFSNRETRDFFTRHLNRQLQQDGVAVKTTEIKRQELLAKAVTKRRRQKIVAKFLKEVFKEASQVGGNGNDHGEDGEPEIRPEEGDTRRVLDCSLTREEFAESMSSNPESLFVDQMFRLADKDGDGAVTFREFLNLIVMFSSGGVERKLRLMFDLYDTRKENRLHREPFKKMFVAMMEMVNASVNDGDLNRLCDAMFQSSGLQEKNYLTFDDFKELMGGHKAALADLSLPGSASPHMKRKQRTSGGASLGSRLRTKLTDFARSPRRWNSSTEEAPKEKEDSTSPPEAGERTSAKSSLETLMKATLSLDLTEENKKDDERAKKEGASVKKGVYFEETPIYRNLQELAQAYSPLKEQPPLVAKPPPSMSILLSPPVRKPNSKLKTLDHRQSSISLQKPQSWLQRKARKLRTLIANYKRQIFLTIIYQLVTVCIFVERAYNYSFEREHSGLRRIAGYGVTVTRGAASAMMFTYSTLLLTMCRNAITHLRSTQFNEYIPFDSFHLFHRQFAMTALFYTAIHIVGHAINFYHISTQPADDLTCLFREVFHTSDQLPKFQYWLFGTLTGVTAVLLTAVTAVIFVFATPYARRHVFNAFWITHRLYIVFYILMILHGAGRLVQAPIFYLYFVGPVCAFIIDRLISSSRDHVRLAVTNAELLPSGVTCLKIKKPRNFEYKSGQWVRIACLAQGQREFHPFTLTSAPHEESLSLHIRAVGPWTSNLRNTYDRSTLKDRPFPKLYLDGPFGEGHQDWYKFEVAVMVGGGIGVTPFASILKDIVRKKEKGGALNCKKCYFVWVTRTQRQFEWMTDIIREVEEQDDKRDGKSSLIDVHIFITQFYNKFDLRTTMLYVCERHFQRFYNRSLFTGLRAITHFGRPEFDQFLNEIKVANPLTKKIGVFSCGPLPMTGAVKSACSKLNEVDGPEFVHHYENF*

>S.ciliatum_1_NOX2 scpid51544|

MGDFLVNEAPRYLVLILWIGMQAYFFVYFCWVFDSSRDFFYLKLVIGKGLCIARGSAANLNFNSMLILLPVCRNLISFLRGSNQGVRRVVRRLLDKNITFHKCIAWSVVLWTAIHTGSHFWNYERITVVHSEVPGMSCIPLNAQPVQLPGVSPIKVMWTTYAGISGHLIVVALFLMVTSSMEIIRRSYFEVFWYTHHLFIIFYVGAVVHGLQGLIQGQVNTGIHDPDRCYQTYMNGSGICKIDHATDPDCDLATARFKSAGAQTWRWVVGPLVLYLVERIIRFIRAQQKVVITKVVQHPSKTIEIQMQMKGFHAEAGQYIFMQVPEISWFEWHPFTLTSSPEENYFSLHIRIAGDWTDALAKRLGVGSGEFQPAQNMPTIRIDGPFGTPSEDIFRFRTGVCIGAGIGVTPFASILKSIWYQSLNPDKQLKLKKVYLFWICPDTNSFEWFADLLKSLEDQMIEHGRADFLDYNIYLTRGWGTDEAKNIMLHEDDEQDPITGLQQKTHYGRPQWAQIFSSMAAAHKGEDLGVFFCGPKILSTKLHMMCNQHTDQAAETRFFYHKENF

>S.ciliatum_2_Duox scpid13674|

MALADYAINRRDDRRLLVLSAVILVLVANPLNHHASHAQTVVNISGRTQERHWEYEPYDGWFNNLGNPEWGAVDTPLQRRLPPAYSDGVYQLAGQKRPNPLSLSAGIMKGMTGRRSYQSKTAMLVFFGQQVVEEILDAQRAGCPPEYANIKIPDDHEYSEETSVGDMPFLRTRYDMRTGQSPNNPRQQLNEITPYIDGGLIYGTSKAWADVLRSFKDGMLRCWDSNCLYPERNTIGLPMANPPPPFDHKLKSAKRFFMLGNPRGNENPFLLTMGVLWFREHNYWAKELKKSYAGWNDEQLYQRARQFTIAVYQNIVLYEWLPAFLGIGGQNVSEPGCPCSDDPGVISPYSGYKPHVHPGIVHAFQSAAMRFGHTMVPPGVWKRNSTCYARPLRGVRTCNSYWNPQDAILDEGGIDDVLMGMASQIAEREDNIITEDLRGKVFGPLEFIRRDLMALNIQRGRDHGLPDYNTARQHYGLRRLKFDDINPCSCGNSTIRQAVNASRELYKGDDRDIDIWVGGLLETTPRGPGPLFREIIKDQFERIRDGDRFWFENIENELFNASEIGAIRARRLSSVIVSNTEIGVGDIQADVFHLRPGDPCASHSFDAGNQIDETLLDACSPWATFDYFEGSAGPYKATFVAVGLYVLLLLTVVFVGIRCRRGGLTRRMTRSKSSGAVGDSTYGLAGSGVVRIIAEELKVEGEHTRHVGILVNESERRLTVVEPDSMTALRRIDLALTRSVEFHMCFDDDTLLLLRFPHFYDLVLQFRHASDRNFFVQDLKTTLSKLGIGEADLFPTRRRLLAYAFTKEDRQQLVEDFFRRVFKDAFGTSTKPENPRLQQVRGMISPIVSQVGSLFGRGKEIVDARDIMQCELTKSELASSLGMKPDSVFVEQIFKVVDETGSGTVAFQEMLTFLVKFAKGDEENKLRLIFRIYNTDGSGLLKRAEFNDMLKHLLEGAGTTVEDAKVDTTWRSMFSDAGLDEQNELTFEEFHRLFSPHMAQIMPNVHLEFAGMSEATSRSPPHTVGVRSPAGRRGTGHDGVSPSPRRKGSRQDNEDIYQKRTRSFVSAYQSHDEQARQQAAGPSIQISPPTVDEELSEKDSLPEAYEAWLSVRRFISNYQAHVFWLCLYHLVVFCIFAERAYYYSVEREHRGLRRIAGYGITVTRGAASAMMFTYSTLLVTMCRNFITRLRETPVNRFIPFDAAHTFHKVVAMTALLFTCIHIIGHSMNFYHISTQTADDLSCLFRNFYHYSDELPKFQYWMFGTLTGMTGFLLTLVVATMYIFALPVVRQYLYFVFWKIHQLYVVLYILLILHGTGKLVQLPIFQWYFLAPVVCYTLDRLISLSRKKVEITVLKAELLPSDVTHLQFRKPDNFQHKSGQWVRIACLALSGNEYHPFTLTSAPHEDTLAVHVRAVGPWTRNLRRVFDPESARVHRNSAGQVLLPKLHLDGPFGEGHQDWYKYSVSVLVGGGIGVTPFAAILKDIVHRMATNVKLPCKKVYFLWVTRTQRQFEWFTDIIRLVEEQDKKGFIDIHIFITQFYQKFDL

>S.ciliatum_3_NOX2 scpid51330|

MGDFLVNDFPRYFVLFIWIAVNCFMFGYFCWIFESEPKWFYLRLVLKRGLCLARGSAACLNLNCMLILIPVCRNLISLVRGSGRSVRRTVRRLLDKNITFHKIVAWSIVFFSAIHTGSHFWNYERICYIWDELPGMRCIPTDAQPINSPGPVPINVMWTTWAGFTGHIILVALFLMVTSSIEIIRRSYFEVFWYTHHLFVVFYACLIIHGFQGLLKGQTNTDYHDPNFCYNKYLLRNGSSCILTTEEKSCRLEEAQFAAGGAQTWKWVVGPLALYLLERLIRFYRAQQRVVITKVVQHPSKTIELQMQKKGFFAEAGQYIFMQVPEVSALEWHPFTLTSSPEERYFSVHIRVVGDWTEALAKRLGVGNEEFQQSYDMPTVNIDGPFGTASEDIFRFRVGVCIGAGIGVTPFASILKSIWYQMFGANRSDNDGSLKLQKVYVYWICPDTQAFEWFADLLKTLEDQLIENGMADFLDYNIYLTRGWDQNMAKNIMLHEDESRDVITGLQQKTHFGRPQWRNIFENMAAAHRGEDIGVFFCGPKILSSALHVECNRCTDLQNGTRFYYNKENF

>S.ciliatum_4_Duox1 scpid10741|

MQPTSIILLGLCAVGAQAFLASNITEDVEINGTFHLPEVEGYDGWYNNLAHPEWGGADMPLSRRLTPVFADGTYQPSGAGRPNPLDIAEATMRGAVGQKSYRNRTALLTFFGQQVVEEILDAQRPGCPPEYFNIAIPKGHRLYDSDGDGGKYIPFLRSRYDKNTGQSPNNVRQQLNEITPWIDGGLTYGITKAWADALRSFHRGQLKCLGNQTWDCTLPDRNDIGLPIVNPPPPADHELKSARRFFRIGNPRGNENPMLLSMQVVWFRNHNYITNRLYNVFKGLGVEVNDEILYNRARQWNIAEYQHIVLDEWLPAFLGQNIPSYPGYSSTTFPGITHIFQSAAMRFGHTIVPPIAYRAMVREEQGVPTCRFRSLGGEESVNPATTVTSVRTCQSYFNSPDIINNEEGVDELLFGLSRQVTEREDNIITEDLQGFVFGGLDFSRRDLMALNIQRARDHGLPDYNTARMSFGLPRVNSYEDINPTVFDDSENDNNTYSTTVEAIRNLEILHNNFTNQPSNVSIDTVDIWPGGLLETVDGPGPLFTAVILDQFLRIREGDRFWFENEQNGLFNQEQIATIRAVSFKDVLVRIMRASWFNSSMLTRNPFRYDGQEDEDFPLFRTQGCRPHIEYNVPNLAQYMEPCSPKKTFDYFQGSEGPFIGSFVSLGLYLLLNVVILFVAVRVVKQRAIKARASNASDGGADVWLKAGVISTSGGVPMVADELKVNPARKVVVAPSVSKKQLKVYDAVSGAVVRSIDLAHIKHCDMLLTPQNKVLTDGVILLRFANLYDLVLRFAGSPEPFSNAMNDFLNHIGVTTSRRYLAENAILADVFTKEDRQKLVEEFFRILFRESLAEEGQPREKTNFIGIRRRTTKRRKQILSLELTKAEFAEAMSMKPDTLFVEQVFKLVDDDHSGTVSFQEFLKFLILFTKGTADNKLELMFNIYDIDGSGTLEREEFTEMLGHLIEQANEQVDSGNLKTVVDSMMASAGLADQRTIDIHQFKRLFGEYRSELDDAAVQVRGKTARKSNAQALSVGQLQALDSIKGGNGASSEPSQLEEEAPAPQAGRYCSVRRRKIDVATAAYENEQDNSASSDLHIEPLPVEAEIGDNAQLSTQADNILRIQRFWENYRLHIFWFTLYQLVVGAIFVERAYYYSVEREHVGLRRIAGYGVTVTRGAASAMMFTYSSLLVTMSRNIITRLRETFLNKFIPFDFAHTMHKLIAYTALIYTIVHCIGHAINFYHISTQTANDLTCLFREYYHLSDELPKFQFWLFNTLTGVTGYLLVLVCVTLYIFAMPYARRYLFRTFWITHHLYIVMYILLILHGSGRLVQPPLFQWFFIVPASVFAIDKLISYSRKKVEIVVYKAEKLPSGVTHLQFKKPSNFDYLSGQWVRIACLSLNESEYHPFTLTSAPGEPTLDLHIRAVGPWTQNLRTVYDPENPKNRDAHGALSFPRLYLDGPFGEGHQDWFKYEIAVLVGGGIGVTPFASILKDVVHKQANNTRFAAKKVYFLWVTRTQKQFEWMTDIIRQVEEQDKKQLTDIHIFITQFYQKFDLRTIMLYICERHFQKVSNRSLFSGLRSVTHFGRPQFNSFFEGLQDLHNSVGQIGVFSCGPPPMTRSVEEGCQHCNQIEGPIFIHHSENF

>S.ciliatum_5_Duox1 scpid15779|

MAASFLLLAVAFFLSQAAGQDNDSITEPIHGSAFHLPEVVGYDGWYNNPSHPEWGGADMQLSRRVPPRYSDSTYEPWTAGRPNPHEVATATMLGYKGLKSYQNRTALLTFFGQQVVEEILDAQRPGCPPEYFNIPIPTGHTLYDPDSEGGKELPFLRSRYDKSTGQSPNNPRQQLNEITPYIDGGLFYGVTKAWADALRSYTNGTMACADPPECRFPAKNTIGLPMANPPPPADHVLKTASRFYKLGNPRGNENPMLLAFGVIWFRNHNYHAQRLAKEWAAIGRPLNDELLFNRARQWVIAEHQNIVLNIWLPSFLGVEINPYPGYSTSTFPGITHAFQTSAMRFGHTLVPPIAYRAYAKDSSSGAVPTNNSDWKCEFRPLKPSSTSSSSAIRTCQSYWNSPDIITEGSVNVVDEILFGLSKQITEREDNIVTEDLRGFVFGSLDFSRRDLMAINIQRGRDHGVPDYNTVRQHYNLSRVTRYRDINPTMYGKEGNITMAIDNLMKTYTNSSEADANSTQYGMDNIDLWPGGLLETTETGPGELFRAIIQDQYERIRNSDRFWFENYRMNGLFTEEEVQTIRATTFRDIMIRNFKADWFQPYMLQQDVFRWNSVVDDPNGRCYTPFESYSDEQLTDSIEACSSMKTFDYFEGSAGPYIGTIISLGLYLLLNIALLVLAVKYQEYKNLKARSSGQQKYDKGRDVWANVGVFSLQGNPLIAEAIEEKTRHPVVVAPLPDTKQLRVFDAISGSVDRTIDLVGVNQVVIRTCPAGDSPGLVCVRFPNRYDLVLEFSTPVDDFLTPLGQYLGSIGVSINQDVMGSKQLLADIITESHRKELVEAFFRELFQDALTQDGGKKKTRKIFQRRKKERKEVLGTELTKHEFADAMGMKESSLFVEQVFHLVDKDKSGSISFKEYLQFVALFTNGSFDSKMDLMFSIYDLNGDGELSKEEFIKMLMLMAEEAGENIGQESIERSIASMIADAGLAHKTSLTVEDFKEIFGKYGNELDSTAMQIHGKTARKSVVKATTTVTSATSQKKLAGVSSAGGGQDETDAAQFRQRLATGLRADKSRSAAAKEARKAYASHEDEDFTSSIHVEPLPVDEELGNSEDTQKDSRSLLYMQRVWENYRLHIFWFALYQLVTLAIFGERAYYYSVEREHAGLRRIAGYGVTVTRGAASAMMFTYSSLLVTMSRNVITHLRETFLNRVIPFDAAHGAHKAFAMTALFYTVIHILGHCINFYHISTQTANDLTCLFREYYHLSDELPKFQFWMFSTLTGVTGYVLTLVIITMYVFALPTARRYLFNTFWFTHHLYFVLYALLILHGSGRLVQPPLFQWYFIGPALLFAADKLISISRKKTKIIVYKAEKLPSGVTHLQFKKPTNFNYLSGQWCRIACLALNSEEYHPFTLTSAPHEVTLDLYIRAVGPWTHNVRQVYDPDNAKNKDLITGAVTFPKLYLDGPFGEGHQDWYKFQVAVLVGGGIGVTPFASILKDLVFKRQANSHIACKKVYFLWVTRTQKQFEWLTDIIRQVEQDDKDHLTDIHIFITQFYQKFDL

>S.ciliatum_6_Duox2 scpid25805|

MLCYRPLLVALASLALFHVSVCIASNTSRTPRGQYYHLPEVEGYDGWYNNPSHPEWGSADSPLSRRIWPAYSDGTYQPNGQGRPNPHDVATACLQGPKGLKSYRNRTALLTFYGQQIVEEILDAQRAGCPPEYHNIPIPKCHALFDPECKGNKEVPFLRSRYDKSTGQSPNNPRKQLNEATPWIDGGLFYGPNKAWADALRSFTNGTLACSDSTCRFPAKNKVGLPFANPPPPADHVLKSSSRFFKIGNPRGNENPMLLSLGITWFRNHNWHANRLRKEFDAQGIKYKDETLFNRARQWNIADQQKISLNDWLPAFLGRNITPYTGYKSTRFVNIGHMFQTSAMRFGHTLVPPVVYRASVRNSKSRDWQCVFRQLSRKKDPTKSSAFRTCQSYWNSPDHLSEDNGDAVDELLLGMSKQVCEREDNIVTEDLRGFVFGSLDFSRRDLMALNIQRARDHGIPDYNTVRASFGLPRIRSFAEINNSTDKFIRAAIRNLSRLYNNDVNNIDLWPAGLLETTPQGPGPTFAAIISDQFEGIRDADRFWFENKDNGLFTDEEIEVIRKTTFRTILSRNIKASWFREDLLQKDVFHFNADVDDSERRCRTEFEQYNDATLTAEIDACSPMKTFDYFSGSGGPYITTIIGIAVYFILNVAVLIVSVKYVSKSRSSGKPKRKQSGPASDFWSNAGVFTAQNGEPLTGELVKSDTVMSVSLGPIIASRQLKVYNTTLGGVERTIDLTGVSTVNIEECHDVTGNNTSGLLCMRMAHRYDLAMRVDTHLQHMISNIAVFCESLNIQVSRKVVFSSKQLLWSVVTKKDRHNVVQSFFRALFKQTSGADASKGATQFIKKQRAKKREALSTELTRPEFAEAMGMKPTSIFVEQVFRFVDTDKSYTVSFQEFFQFLAVFTQGSSDTKMELMFAIYDIDGDGVLSHDDFSTMLGHLAEQAGETIGKEKLDSILSNMLKTSKSTDSMGMTLEEFKTIFGEYKEVMGDTTVQLKHAKSAVKNLRNQGVNNSKSVQISKNSAQFGLGGSHQSTIRGKSSTTSFRNHYTPAEKSDGLQVKETSMPGEQLDNLSPFQHQVLYVQRLWENYRFHIFWLTLYLLVVLCIFAERAYYYSVEREHVGLRRIAGYGVTVTRGAASAMMFTYSSILVTMCRNLLTKLRETPLNRYIPFDAAHSAHKVIACVALFFTVMHILGHCIKFYHISTQTANDLTCLFREYYHFSHELPKFQFWMFSTLTGITGLLLTFVVVTIYIFALPQARRYLFNTFWIVHRLYIVLFILLILHGSGRLVQPPLFQWFFIVPAALFVVDKLISLKHKKHDIPVFKAEKLPSGVTHLQFKKPKNFNYLSGQWCRISSRALNSGEYHPFTLTSAPHEETLDLYIRAVGPWTENIRQIYDPENPKNQNTENKGLTFPRIYLDGPFGEGHQDWYKYDIAVLVGGGIGVTPFASILKDLVCKKQSKVQIPCKKVYFFWVTKTQKQFEWLTEIIKQVEQQDDQKFTGIHIFITQFYQKFDIRTIMLYICERHFQKISKQSLFTGLKSTVHFGRPQFDKMFDSLQDIHDDVAQIGTFSCGPPPMTRSVEAACVAANQNEGPVFFHHYENF

>S.ciliatum_7_NOX2 scpid47436|

MVSKWLVNEGPRWAFTAIWIGLNIFLFAWFCHFFETADDFWYLRRYIGVGLCLARGAAVPINLNCTLILLPVCRNLISLVRGSGIGVWRWMRRLLDKNLTFHKTLAWAILFWSAIHTGAHFWNYHRVVTVWEENPGMTCIPPMSNPVRGPGNDPIIIMWATVAGFTGFIILAVFFLMITSSIEVIRRSYFEVFWYTHHLFLIFFAAFLAHGFGELIVGQNNTDEHNPEFCYSRNRRFANGTCETVIIDGSGDRNQCDLNTATFSHAGAQSWKWVVGPLALYFLERVIRIWRSWHPATICRVVQHPSKVLEIQMQKEGFYADVGQYVFIQAPEIAPLEWHPFTLSSSPEEDCFSVHIRIVGDWTENLARRCGMNVSGRAEDEPPPISAPPKLLIDGPFGTASEDVFKHRVAVCIGAGIGVTPFASVLKSIWYQSLEAGSLISVQKVYFFWLCSDLSAFEWFADLMRSLEDQMNAQQRGDFVQFRIYLTRPGNTAEQIRALVASTMAEDGSPIVQMQDANVLQFARPNWVQIFDSIGSNHLGEDIGVFFCGPKALSSRLHTICNQFTNTATGTRFFYNKENF

>M.leidyi_4_NOX5d ML18201a

MVNKKEYKVLVSEEESGGEEEDPRTQPVPCRSPPDSDSDLENSGEDHRLLPSLSLQNSPDSDRRSQSESDRRSRSDSDKRSRSDTDKRSRSSKSPNKGRLSRMEADTILGGASFTANEPDFEHNTFLGKLRELAKQSEIERQRVLEDKKRETKAWFETYFRELLKTPAGNEGDLEITKKDFLRLLNNKDEFAEHCFRLFDSDDSGDVSIQEIQAGLNKLSETGEANKKTIKWFVSRLEKFTNGDVTKEIFKKAINQTFLSKLFELMDFDSGGTVTLEEFKRSVDRLLMMGSDEKILAYLENMFHEHAKDPENITYDEFKKVIYNKQEYFTKKMFSICDKDKSNSISLQEFCESLGKLMKQSDNEDEMAKINKDKLHFLFRIYDDDDSGEIDRDEMRECIEICTKESNLDLKDVEALADTLFDKADADKSGSISFDEFYNILKPFTNNLEINPKGWVLPEKPKKYSDKLKEITGKYFSKNYLKNNKVLISNIIVLVLLYVLVFTIGIVYYKETYPALMIAKGAGKCLNLTSTLLFVSMCRWVLTSIRNTPAGKFFALDVIVDIHKVLGVVYVVFALIHTAAHVFTFLENSKINTVVQEDNSTKVDYNFLLTTGGYFGGSYISGILLDIVLCVMIPFAQSCVRRSGKFTQFYWVHFFCYWLLAIGTILHCQNFWKWIFIPLFIYSMEKITNINFIKRTIYGKFRIINYKVLPSNVVELHISRPTTFNFHSGSYAFLTVPLITGYEKHPFTISSSSEDKEKITFHIKMMRGWTKQLYEVLASDQEFKKRREMKELEDAKADGNGEVSLKIEQSANQRYLNRNSEKYAYLRNFGRLQKRDLECYIDGPYGTPATEIFESEHAVLVAAGIGVTPFASILQTIVDKYNSSGCFCSECDHWNDHGTFNIKIKKLDFVWITRDQKSLQWFLELLKRLEEVMDRFSENNPGHTKQPTIYDRKKSRTPAVRIIKDSNKKNGIELQELDVSVTENGQNGSENGEIPEKKKEEKERFLDIHIYLTSALSKKDFRAVGLQYALQLMREGNPEGQDHITGLKAQTNTGRPDWDELFDKIDKPEKHRKVTVFFCGSPVIEEVLKEQCIKRRYKFKAEKF

>N.vectensis_2_NOX2 EDO38325

MNFYFSQIIWAVINIILFATTYHNYGVTKKYTYLREVIKNGLPIARGAAMVLNFNCMLILLSMCRNLNSLIRRHCKSTCMAPVIRVLDKSITFHKYIAYTICFFTIVHVGAHCYNFENLIDSWSKENEIDAKLSQLDGADNWVNPIRSEMSVGKRGSKFAGVTGAVITLCLVVMVSSSTELIRRSYFEVFWYSHHLFIIFFAGLVAHGCGEILRYQTNMDKHDPDVCRKQENLATWGVKDPCRVLPEFEAAGAMTWKIVLLPMVIYFLERCLRFWRSMQQVKVVKVVVKHPSRVVEIQMKKPGFVCEAGQYVFLQVPKISQLEWHPFTLTSAPEEDYFSLHIRVVGNWTTDLANQLGAGNQQIAIDQMPRIAVDGPYGTASTDVFRYEVVMCIGAGIGVTPFASILKSIWYRHNQDLENLRVKKVYFYWICRDTFAFEWFSDLLKHIEIQMDEMNMPGFIQINIFLTGWDKKLANQVVMERSADRDPITGLFARTKYGRPEWAKIFNEVAEAHNQTSVGVFFCGPSGLSHELHKMCTQSCDETKGVRFFYNKENF

>N.vectensis_3NOX2 EDO38588

MANLGNDVARYIVLIIWMGINIYLFIDVYYKLATTKENMYLNKLVKFGLALARAPAMVLNFNCMLILLTMCRNLLSFIRGSCKCCSFMLRLLDKHHVTFHKYIAYMICLQTAIHCIAHIFNVEFLILAWQKGGIYTKLCQLEDVGNETYINPIRDPNADPITVLWTIVSGVTGAVITLALILMLSSSTELIRRSYFEVFWFNHHCFVIFYIGLVLHGVQGIIRYQSNVDKHDPEICMNHTLWYNHPKCMEPPKFVPFGMNTWRWVVGPMFLYCIERLIRFVRSHQAIQIIKVVKHPSNVIEIKMKKAGFRPEVGQYIFLQCPKISKLEWHPFTLTSAPEEDTFSVHIRIVGDWTGDLAKLCGYKGQKLQSVNEMPRLALDGPFGTASIDVFKYGVGMYIGAGIGVTPFASVLKSIWYRYNENPSDLNIKKVYFFWICNDTNAFEWFTDLLKILEEKMIDSGNAGFLEYNIYLTRGWGANMARDIYLREDEVEDPITRLRQKTRFGRPEWNKIFNDIGNKHPKTDIGVFFCGPKALSHTLHKMSNAHSRDGDGAKFYYNKENF

>N.vectensis_1_NOX4 EDO46686

YFFFQVLWTGPFIVIFWQTYNKYSNCPEYFYLHNMLGNSLSWSRGAAAVLWFSCSLMLLPMCRNLLAFVRNTLCKSSRRLRRLLDKHIWFHKACAITTIIAAVVHTVAHLINGKRFSENYSTDHPPLNFAKNRDQDPLEFVMLSVAGFTGMGMMLVLLIMIAASTPIVRNRSYEVFWFTHHAFIAFYLMLAVHGLGGVIKHQTNLAAHTPGCKVPLNDSMDTPFNMTSLPLCREAEFAPDQPQVYHKSNTQVYQKPYHTVYCKCYHTGIPQSWKLLILPLSIYLIDRLIRVVRGYQEVTVIKVVNHPCGVIELHMKKSGFYAEPGQFVYVRCHSVARFEWHPFTLTKCPSSKDDSFSIHIKRTGDWTKSLSDQKPLLAALDDSGSLVAAPSVRSAVLSVDGPYGSPCMDVEEYRVSMCIATGIGVTPFAALITRIRSQIIHAQRPPRPHRLYFVWICREVGALQWFADLIHETSRQLWELNRPDFLTCLFYITSKGQKEKICINHTQSSWFDARLTHGRPDWFQIFRRVSQENPKTCVGVFYCGARGPSSMLRRCCQRMYKNGASFVFNKEVSA

>A.planci_6_Duox gbr.169.31.t1

MPVTMPTTESNAATTGVPPASTTGAAPMATTGAPTIANPGTMAPPTTSGGVQPTSASDDGMLSPEERRQYEERIGVFDYYHREYEGYDGWYNNMAHPDWGGSELPLTRRLPVAYADGVYMMAGKDRPNPIDVSEATMRGDTGLPSFLNRTAFMTFFGQQVVEEILDAQGAGCPPEYENIEIPEGHEYKDMTKNNPHGSVDFIPFLRTRYSFNTGYSPNVPREQLNEITPWIDGGLVYGTTKAWADALRSFEGGRLADNSKNNPNEKGFPEENTIGLPMANVPPPAEAEDHVLRNVRRFFKLGNPRGNENPFLLTFGILWFRWHNYWADKIYNSTMDHRYEWNDERIFNEARKWVIATYQKIVLYDWLPAFLNMDAQEMETNRYTGYKSYVHPGITHEFQSAAMRFGHTLVPPGVYRRDAQCNFLNTSLESSNIPESQFRSVHGVRTCNSFWNPQLPIREYDIENFLMGMASQITEREDHIITIDLQRRVFGPLDFSRRDLMAQNIQRGRDHGLPDYNTAREALGLTPVKSFREINNATIFENDQTGIDPSILDNLKEVCNGDLSKIDIWPGGLLETTANGPGPLFRKIILDQFLRTRDGDRFWFENEQNGLFTKEQIEYIRNNISVYDVLILATNIKPGEVQQDVFHYMAGDPCWDMHPNENRSINEKDMEECTMLYTFDYFTGSEISYAVSYLALGLFCVGVVLTLFICARRRTKATEEVRKATRTKTRKGKGPESLQKFPALEWCGSKSGTRPIQVALGPGKAIKVMNERGNKLYRTIDLAHHQKITLCVSPDGNRRYVLLHMEREYDLVLKFDDIESRTEYISELQSFLGSGEVGLGQERQETREKELLRMAITKEHRQKTLERFFRVAFAQAFNESVDSTDLSELERTDAKDILNNELTKTEFAEMLSMPPDSIFVEQMFDLVDKDQSGALSFREFLDVIVIFAKGKPEEKLKLMFNMYDVDRSGHLSRDEFMTMLKSMMETVSASLEQKQLEDVISSMFKAAGFENKEELSLEDFITLMGDHKEELSQAALQLQGADVPEAAQPTPDRGATVIRRENAPSRARKTIVRAYAEKDSDRPRTAKNRYQSRVIRVETKKATYTENRTQQKLNRFLRYIENNRLQLFYLVLYNLVLAGIFIERAYYYSVEREHGGLRRIAGYGVTVTRGAASAMMFTYSTLLATMCRNTITFLRGTFLHRYVPFDSALFFHKFVAMLALFWSVVHTIGHSLNFYHIATQTASDLSCLFRDFFNGSHELPKFHFWAYRTITGFTGILLVMVCAIMYTFAFSYARRKVFNLFWLTHNLYAFYFFLMVMHGSGNLVQPPFFYYFFLPPVIVFTLDKLVSVSRKKAEITVVKAELLPSNVTMLEFKRPTTFEYKSGQWVRIACKTLNSSEYHPFTLSSAPHEENLSLHIRAVGPWTINLRHTFDPNVVREQPYPKLFLDGPYGEGHQDWYQFEVSVLVGGGIGVTPFASILKDLVYRSSQGQKFTCKKVYFIWVTRTQKQFEWLTDIIREVEDKDVNDLVSVHIFITQFFQKFDLRTTMLYICERHFQKISDRSLFTGLRSITHFGRPQFEPFLQSLQEEHPSVGKIGVFSCGPPGMTNNVEKACSELNKYDGAAFIHHFENF*

>A.planci_7_NOX2 gbr.25.198.t1

MGDRLINEGLKTIFLLLWLGANVGYWIQVFWTYEYGPQYFYIRRIIGPGVSVAKACGACLNLNSMLILLPVCRNLISFLRGSCETNKLCRRSVRRQLDKNLTFHKTLAYMITLLNIVHCIAHFFNFRNLYDHFRDCENYFPADSPYKGVSCKLRDLEAMGGTWINPVRFENKALPFGFPLIEQGVIQIAGWSGVVLTLVFIIMFSSATEFIRRSYFETFWVTHHLFVIYFAMLLVHGVGGVIRSQNNLAEHNITYCSQQPELWTPSSQPCPYPNFVMGSAASWKWCVGPIAIYILERFVRFIRSCQKVQVIKVVKHPSKVIELQMKKSGFKMLSGQYVFLKCPALSHIQWHPFTLTSAPEDDFFSIHIRTVGDWTTGLAKVMGADNDTPIESNQLARVAVDGPFGTASLDVFKYQAAVCVGAGIGVTPFASILRSIWHQSMDPNLDLKLKKVYFFWICPDTNAFEWFSEMLDSLENHMIEQGKADFLKYNIYLTRGWNTKQAKNIYLQEEEEVDAITGLKQKTNYGRPKWDDIFKMIAEENPGTSVGVFFCGPKVLSEVLHKCSNKHSNIKGGARFFYNKENF*

>A.planci_1_NOX5b gbr.37.16.t1

MEKGEEFRLTVEDDAKWLEWAQRQFAAIAGNDRLIDWQEFKSALKVKKEFFAKRFFALFDADNSGTISLDELMDGLRMLTHGEPAEKLRFLFNVYDVDGSGQIDHEELRTVLESCMEESALQLSSSVLDELTDALFEAADEDGSGAISFDELRRELEKYPDVMENMTIGAAGWLKPPEDQPKTRMRMPRYLTLRYINNNLRKVIFVIAYCLLNAALFTEAAHRFASNGANWCIVIARGCGQCLNLNGSLILVLMLRQTLTYVRSTRLARFLPIDQHIIFHKTVGLIIAALSLVHSLAHVGNAVIITSPSGPLPNNTAWEVLFTQPHLTPLGLVKHSAFLTGWLLDIILVVMVICSLPFVRRSGNFQVFYWTHLMYIFFWVLLLMHGPRFWYWFIIPGIIFILERISQLRLVRQAQFGKMYVEKAELMPSGVTHLMITRPPHFDYSPGDYLFLNIPEIAKHEWHPFTISSAPEQPDFTDTYSLTKRLPHNFSSRVDTLSLHIRSAGNWTNKLYSFFEERHKRNRQQSIRNRSRRANGSTSTEKAGEDDSVCLDVPPSPPSDSYRYRPGGEKVMSRQPVTKSMSWSDKNGKTATYVEVSTGKKHSNGKHRKLKRHPAEERIEVFINGPYGTATRAIFRAEHAVLIGAGIGVTPFASILQSIMLRCRTNQQTCPNCHHEWIGSVPEHVMRIKKVDFIWINRNQKAFEWFVRLLTQLELQQAGMSERFLDMHMFMTAALGKTDMKGVGLQMALDIMHSKGNKDMITGLKTRTQPGRPDWDKLFADIAGQRKGKVQVFFCGSPILGKTIKAMCEKFKFHFHKENF*

>A.planci_4_NOX5a gbr.452.9.t1

MDPYTSQDHFRKISILSGRPPYWRRNVLDADTKWLVWAESRFASIAGKDTDIDRESFKNALEVRNVFFADRFFDMFDYDHSGTVSQQEMMDGLFLLTKGTKEEKLRFFFDVYDLDGYDTVKKILTKHFNLPDAETEAAHRTGRAPKATTDKPRHIITRFLYRTKRHSVLMGAKEALTNTGMFILPDLPAADVAKKTSLRDVMTLVTTRLPYLLTDIQKNGTIEKDELRALLKTGVEESSLQLSDRKVDHLTETLFNTADQNSDGSISFDEFMTALKRYPDLLENFSIGAAAWLKDTRPKRRGCRGIPWLRVNYLRNNVQRIVLLLTIILVTGVVFAEAAYRHSFNPDANWCFLIARGCGQALNFNCAIIVLMMLRKTLSFLRSSTLLMQILPFDDNIMFHKFIGYLAAILALAHTLGHVGNALILETYKNFTAVELLFTDPTLLPVGLRVGKLVGSAFITGWILDIVFLVLILGSLSYVRRSGHFEIFYWTHKICYFIFWFLLILHGPVFWAWFVCPLILFLLEKFSNLQIIRRLRHGKTYIKEANLLPSGVTHLVMTKPRNFKYKAGDYVFVKIPEIARNEWHPFTISSAPEQADILSLHIRSVGNWTKRLYRFFDDMQSITIKKSQRVNSSLRLPPMAGKVPSDAGQHINPGFEMSPKDDCKTSNGTPRSRDANKPYASEVSDTVVRIATDYSPTSTDEVEPPPVMKIRRVSFNAEVDSQTVVQWKKTSLPRRIPEVDEELEELCKIKEDRRHENQSAHVGKVAAETTNESEGSSSICEASTSSVQTSKKRKQAVCKPNALVKETSRNMMTSITQQYGRKHSKAPPRPQILFDMNTNNKIQVHIQGPYGTPSTSVFESEHAVLIGAGIGVTPFASIMQSIMCRHRSIVTTCPNCQHTWVDEEREKQALRVKKVDFIWLNRNHTAFEWFVDMLLNLEQGQSQYSLDRFLDIHLFMTGIQRFDMANFGLQMALDLVHERENRDLLTGLRTKLQAGRPDWNKLFTQIQEEDKGRVSVFFCGSPALGIVINKHCQKFGFIFHKENF*

>A.planci_2_NOX5c gbr.460.7.t1

MADRSGKCKEQSIAMETLESESGMDKDASWLAHFEEQFSQVAGDDRNIYLEEFTKALNVKKSFFAERFFHLIDTDCSGSISLKELLVALRLLVHGTATEKLRFLFRVYDVDGNGSIDFSELRTVLKSCTAESALQLSEETLDELTEVLFEEADTDSSGAISFEELQAQLEKYPDIAENLTISAADWLRPPSASQKKKKQSASHDCCRNRCPRLRLKSVRNNQSIIIFIAVYVALHIGLTVWGVVGSWRAFPDKPWLMVARGAGRCLSFDCVFVLCLMLRKCLTVIRSTNVAAALVLIDQHVDLHKLVGYAIVGLSLVHINGHVFNFLDMSLGATNYTLVEYLFTVTPGIGWVAGTASITGHALTVILLIIFVFSFSFIRRRGYFQVFHMTHQFSILFWILLILHATTFWMWFILPGTLYAIERFLRLKLVRRARDGRTFIKKAVVLPANVTLLIIPRPPKLRFHPGEYVHINIPQIAKNEWHPFTISSAPEQKAFITLHIRAIGNWTKRLRAVCRERQQKELTCFENPASEIREGKTLEVLAEEGSLRSPIASPKRSPGQSAEGHELQFALPGTLGSEDERPSRTTDYRSKVDRCNNVREQGVGGDDKNRLTDLSDTESGIETVVPVESTKVKPLSGPLILPPIQKMSPLISECYAEDRPLSSKATPSKPTSTPTSTAGPSRKDSGVSIEQRVLQSVRASSGHSETDLRGKSTGSQPRTFLSWTPQSPPVSRRRSSSFNRKLTQHSLDVVESLVEDPDEHTGIEVYIDGPYGAPSQHIFETDHAVLIGAGIGITPFASILQSIHERHKMATKRCKKCNYAWLEDTPSLLRLKKVDFFWITRQQRSFEWFLSLLNHIELEQEEMMSSDRFININLYMTAALGKHDMKAIGLHMALDLIHKKKRRDMITGLMTRTQAGRPDWDRVFDDIDQRKRGKVTVFFCGSPSLGEVLKRKSQDHGFGFRKENF*

>A.planci_9_NOX5a gbr.534.2.t1

MGTRVVLSMFFTPFYYGVYYHESVGVKGNVLDADAKWLVWAESRFASIAGKDTDIDRESFKNALDLRNEFFADRFFDMFDYDHSGTISQQEMMHGLFLLNKGTREEKLRFFFDVFDLDENGTIEKDELRALLKTGVEDSSLQLSDRKVDHLTETLFNTADQNRDGSISFDEFMTALKRYPDLLENFSIGAAAWLKDTRTKRRGCRGIPWLRVNYLRNNIQRIALLLTIILVTGVVFAEAAYRHSFNPDANWCFLIARGCGQALNFNCAIIVLMMLRKTLSFLRSSTLLMQILPFDDNIMFHKFIGYLAAILALAHTLGHVGNALILQTYKSFTAVELLFTDPTLLPVGLRVGKLVGSAFITGWILDIVLLVLILGSLSYVRRSGHFEIFYWTHKICYFLFWFLLILHGPVFWAWFVGPLILFLLEKFSNLQIIRRLRHGKTYIKEVDLLPSGVTHLVMTKPRNFKYKAGDYVFIKIPEIARNEWHPFTISSAPEQAGILSLHIRSVGNWTMRLYRFFDGMQSITIKKSLGVKCPKSPSDADQRVNAGFEISQDDCNNFNGTPQSDDPYRPTVDDTAVRIATDDSPTSLDEVESPPVMKVCRVSFNAEVDPQTVVQWKKTSLPSRIPEVDEELVELSEIKVDQGHENQSADVGKVAEEIGEEVHIQGPYGTPSTSVFESEHAVLIGAGIGVTPFASIMQSIMCRHRSIVTTCPNCQHTWVEEEREKRALRVKKVDFIWLNRNHTAFEWFVDMLLNLEQGQSQYSLDRFLDIHLFMTGIKRFDMKNVGLQMALDLVHKQENRDMLTGLRTKLQAGRPDWNKLFKEIQEEDKGKVSVFFCGSPALGTVISKHCQKFGFMFHKENF*

>A.planci_3_NOX5a gbr.534.4.t1

MECGNVLDADAKWLVWAESRFASIAGKDTDMDRESFKNALDVRNVFFADRFFDLLDNDHSGTVSQREMMDGLFLLTKGTKEEKLRFFFDVYDLDENGTIEKNELRALLKTGVEESSLQLSDGKVDHLTETLFNTADQNSDGSISFDEFMTALKRYPDLLENFSIGAAAWLKDSRPKRRGCRGIPWLRVNYLQNNIQRIALLLIIIVVTGVLFAEAAYRHSFNPDSNWCFLIARGCGQALNFNCAIIVLMMLRKTLSFLRSSTLLMQILPFDDNIMFHKFIGYLAAILALTHTLGHVGNALILETYKNFTAVELLFTNPTLLPVGLRVGKLVGSAFITGWILDIVFLILILGSLSYVRRSGHFEIFYWTHKICYFIFWFLLILHGPVFWAWFVGPLILFLLEKFSNLQIIRRLRHGKTYIKEANLLPSGVTHLVMTKPRHFKYKAGDYVFIKIPEIAMNEWHPFTISSAPEQAGILSLHIRSAGNWTKRLYRFFDDMQSNTKKKSKXI*

>A.planci_5_NOX5a gbr.534.3.t1

MDIQACQKAVIGQIAQAVKTPSVSFLHDQSQYSRDQVNSDLNQIGPNTNSDPRPSRTHNQLGPTPIRIHANLDPPRVNSDPLSHLVALHHKRVRRPKECRLLNLAKRNVLDADAKWLVWAESRFASIAGKDTDIDRESFKNALGIRNVFFADRFFDMLDNDQSGTISQQEMMDGLFLLTKGTKEEKLRFFFDVYDLDESGNIEKDELRALLKTGVEESSLDLSDQKVDHLTETLFNTADQNSDGSISFDEFMTALKRYPDLLENFSIGAAAWLKDTRPKRRGCRGIPWLRVNYLRNNIQRIALLLIIILVTGVVFAEAAYRHSFNPDANWSFLIARGCGQALNFNCAIIVLMMLRKTLSFLRSSTLLMQILPFDDNIMFHKFIGYLAAILALAHTLGHVGNALILETYKNFTAVELLFTDPTLLPVGLRVGKLVGSAFITGWLLDLVFLVLILGSLSCVRRSGHFEIFNWTHKICYFLFWFLLILHGPVFWAWFVGPLILFLLEKFSNLQIIRRIRHGKTYVKEANLLPSGVTHLVMTKPRHFKYKAGDYVFIKIPEIAMNEWHPFTISSAPEQAGILSLHIRSAGNWTKRLYRFFDDMQSTTKKKSQRVKSFHHEKSLSDAGQHVNAGFEMTPKEDRKSSNGTPLSDDPKGPTVDRVDDTAVRIATNDSPTSLDEVEPPSVMKVSRVSFNSEVDPQTFVQSKKTSLPSKIPEVDEELVELSVIKVDRGHENQSADVEKVDEETGKECEDTTTIPEVDAAASKNRRHGVCKGNNLVEQTSESMMTPNGPTAYFSGQVSKDNLRPQVLFDMNINNKLQNDENSTSCEATTSSEEASNKREQAVCKPNALVKQTSRNMMTSNTEYYSRKHSKAPPRPQILFDMNTNNKIQVHIQGPYGTPSTSVFESEHAVLIGAGIGVTPFASIMQSIMCRHRSIVTTCPNCQHTWVDEEREKQALRIKKVDFIWLNRNHTAFEWFVDMLLNLEQGQSQYSLDRFLDIHLFMTGIQRFDMKNVGLQMALDLVHKQENRDMLTGLRTQLQAGRPDWNKLFKEIQEEGKGRVSVFFCGSPALGRIINKHCQKFGFIFHKENF*

>L.anatina_9_NOX5b g14052.t1

MASARSKGRIDTGHLPAGTYHPGLEQRNRNNGMRVVLATPEPRRRFDIQTHLPSVFSVCVYVLLVASLLTEKWFAFAADHVDEEFTGWEVLARMSARLINFHALLVLLMMLRVTMTLISSRIRIPFWIPLDKHIKYHKVAGMVLLVCSLLHTVGHIGNAAWAVTEHNHTLVEYLFTVHPHHHQASWMGGTAYISGWALLAILLALSLSGMPWVRAKNFQAFYFTHLLYLPFMIVMFIHAPSCWKWIIVPSSLLVIEKVYRTKAVKRLEYGKIFIQEGVLLPSKVTHLVIKRPDNFNFTAGDYIFLNIPKIATFEWHPFTISSAPEDPDHLWLHIRAVGRWTNKLYEYFEEKREIAKRMNLPHIRNIYGDVDGIEEDASSLSSLESSSDGHGIDNVAFEAIDESRETKNNAKNEEKSRRSITHGVIPIIKFDLPDKGNGSGSDNISESESETENDIGFEDDTNIEVVTLKSDTGMGNGSYTSSNYSLQTYSDEDDESFNIKQSRRLTSFRNFSDRLGIPENFRQNLSGGVQKKVQRSKSIDNAHIMKTELDPFVQIPPGFDRNRRKSACALKLPETEVVKHLQRRLSTWQGMSHGQNFGHLSQMPFIYKMNADEHKPSIDEALEVYIDGAYGAPASNFFQAEHAVMIALGIGVTPFASILQSIMRRYNTAKHECPKCTHSWIDEDLSDTLGSLKKVS

>L.anatina_1_NOX5a g7124.t1

MSQSNTPGSRPNSMSASSSRWIINVQKQFDLVADKDGEVTLEGFKKALALKEDFFAARLFHILDKDKTGGLNVHEWVVGLSLFHHGTYEEKLKLLFQAYDVQDLGYLDFSAVRNVIKSCVKESRLTISEETIDDLVVSIIDEADKDLDGRINFEELSNVLKSYPGVIENLTVSSVSWLQSDSSDFVPDVQPPCPCCPQFDREYAQNRIPDIIFVTLYFIINIVILTHRSLEYYFFDVNQGPVTGFDIVARACGQALNFNCSLVVALMLRVTLTLLRATWIGNWLPIDKHIKYHKYVAVVIVILSTIHTFAHIGNFAMISPSLNITLAEALFTTAAGIGWVGQSASITGWLLVIILIVLVIGALPCVRRSGHFEVFFFTHFLYAPWLLLVFLHGSRFWMWFIAPCTIFICEKIYSSRWLKRLYQGQTHVKEGVLLASRVTHLVINRPSNFDYGPGDYLFINIPKIAKFEWHPFTISSAPEQSEYIWLHIRGVGQWTNKLYDFFDEKRKRWSRALSSKGKSMKGRAGRRLSIITPSLLHQAEDNSGRSPNGKANGQKLLNRFSDILEADEEEKRNSLQVPTEGEDVCQSDEDVYEECDDTKHDHPYENTSFQADESEVKIEIPNVDESKKDPSLKPTPAKRNKLQVPGEDGGLGAEENVIKRRKTVLQRRLTKWGRESHGQNFGHLTNAPFVYKVEPDNEEAPQEESLEVYLDGPYGSPSAHIFSAEHAVLIASGIGVTPFASILQSIMLRYKIARHVCPRCNHGWTDKRPITLKNLKKVDFFWISRDQKAFEWFVSLMSQLEIEQQEEGSGLEHFLDMNVYLTAAVKKNDLKGVLLQMALDLVHSKEARDKITGLRTRTQAGRPDWDQVFSEIKRTKHGKVTVFFCGNPALAGTLRAKCRQFGFNFRKENF

>L.anatina_5_NOX4 g12656.t1

MNMWRWLRGFLRNCGLRYSVVVLWIVLNAVVFTSTYRHWKYAPKYYYVHQILGPTLCISRGTAAVLNLNCGLVLLPMCRVLVSLMRFSRISRRSFRMLLDHCKGFHMLCGLTIVLASVIHFIGHLFNAANFSIHFNPYYVDDPLYIVAGSVPGITGLVLMLVLCTMATTSLYVVRTNNYDLFWYTHRFYLVFYFFLMVHAVRGVLKEQINLSRHSPGCDKENLNLSIPWPEPEHWDTQETVCEEPPVFGTVGCSSWMWVGLPLLVYLVDLTYRRCKQGHCTRILNVTEHPGDVLEIRVWKKGFKAKPGQFILVQCPAISSFEWHPFTVSMCPTSQCESFTIHIHAVGDWSGRLQQYLTGSEGWKYVLPPLASSKEDLDKRLVIEGPYGSPSGDILKYRVNVLIAGGIGVTPFTAFLHHLYSNEKLHLQNIERIYFIWMCKDLRSFKWFTQLLCRLYQKLWSLNRPDLLCIRLHVTAPVPGSQLKMFVKQYPLLEKRLYIGRRPDWPLTLMEICSSHKGTDIGVFCCGPKGLATNVEEECSRIWHKNANLFCHHESF

>L.anatina_4_NOX5b g5876.t1

MASARSKGRIDTGHLPAGTYHPGLEQRNRNNGMRVVLATPEPRRRFDIQTHLPSVFSVCVYVLLVASLLTEKWFAFAADHVDEEFTGWEVLARMSARLINFHALLVLLMMLRVTMTLISSRIRIPFWIPLDKHIKYHKVAGMVLLVCSLLHTVGHIGNAAWAVTEHNHTLVEYLFTVHPHHHQASWMGGTAYISGWALLAILLALSLSGMPWVRAKNFQAFYFTHLLYLPFMIVMFIHAPSCWKWIIVPSSLLVIEKVYRTKAVKRLEYGKIFIQEGVLLPSKVTHLVIKRPENFNFTAGDYIFLNIPKIATFEWHPFTISSSPEDPDHLWLHIRAVGRWTNKLYEYFEEKREIAKRMNLPHIRNIYGDVDGIEEDASSLSSLESSSDGHGIDNVAFEAIDESRETKNNAKNEEKSRRSITHGVIPIIKFDLPDKGNGSGSDNISESESETENDIGFEDDTNIEVVTLKSDTGMGNGSYTSSNYSLQTYSDEDDESFNIKQSRRLTSFRNFSDRLGIPENFRQNLSGGVQKKVQRSKSIDNAHIMKTELDPFVQIPPGFDRNRRKSACALKLPETEVVKHLQRRLSTWQGMSHGQNFGHLSQMPFIYKMNADEHKPSIDEALEVYIDGAYGAPASNFFQAEHAVMIALGIGVTPFASILQSIMRRYNTAKHECPKCTHSWIDEDLSDTLGSLKKVS

>L.anatina_8_NOX5d g14051.t1

MEDILAEARKQHASHAGEDGEVDLETFRTCVAFCEPMYADRLFKILDKDGSAGLSLQEWEEGVRRVYSEKEIERLQILFDVFVTEGEGAIDTSAVKAILIEALGGEDHTFSEEYVDSLVQLLIEEAAKDLEDEEDCRLDFEEFEELMNKFPALSTHLVKSTTGWFRNIIKAGSKTTLPRPKESSRTRSCCPNCQTTELLNRSPSGVAIFLYFSVSAGLIAERVLRFLANNSEITGFDILARVCGQLLNLNCMLVVVLMLRVTLTLIRSSKVAFWLPIDQHIDFHKAVGVVILILSILHTGGHIGNFIMVAPSMNITVAEALFTTKSQVGWVAGTACPTGWALLVLIIIMFIGAQSFVRRRGHFELFYFSHILYAPTLILLVIHGPSFWKWFVAPGAIFVVEKLYRFKWIRRLAYGRTFVQEGILLPSKVTHLVLDRPESFSFNPGDYVFINIPKIAKFEWHPFTISSAPEYRGHIWLHIRAVGTWTTKVNEYFDEKRKRWEATMPTLKSRRLAVSNFQFSPAELMIQERRMSHQIDAFRSSIRSKVSASGSSLRSMRSRANTGGSSMRSISRRLHGHDVGAEENALSNHLLERERRMNHFNRVSNGGLNSTSGHAHEGHINIAFEIENDGAGNEKESVPMAEQNLQQGSNLLSVSRIDDILEEDEDSKSLVMGQSDSQDISTVEPYLNSKERKPSDNLNILNTQKKQHTADLDLCNVSLEDKLDIENNKIGDNTGETTNVTAANNGGEDSECKNSENSHMDHGNEDVATSDESYKRENSDSGNVSDDKESRTSMEKYKPSDGSTKEDKSEKSENSDKSEDEEDDILPPDFKPPPLRRLGTSNSMTGHNRNRRMTLCQRRLSTWEAVSSGQNFAMTKQKPFIYNIDPAADTTLNELLEAEHAVLVAGGIGVTPFASILQSILLRYQREKNVCPKCNHAWLDELPCTLYNLKKVDFFWLCRDQSSFEWFVSLLAQLEMEQEDLDNLDDSIIDFHIYMTAAKTDMRSVLQQITFDLNHQVEARDAITGLRTKTQPGRPNWDEEFQKLKNLKKGRVTIFYCGPPGLAVVLGKKAAEYGFGFRKESF

>L.anatina_11_NOX5d g5875.t1

LIEALGGEDHTFSEEYVDSLVQLLIEEAAKDLEDEEDCRLDFEEFEELMNKFPALSTHLVKSTTGWFRNIIKAGSKTTLPRPKKSSRSRSCCPNCQTTELLNRSPSGVAIFLYFSVSAGLIAERVLRFLANNSEITGFDILARVCGQLLNFNCMLVVVLMLRVTLTLLRSSKVAFWLPIDQHIDFHKAVGVVILILSVLHTGGHIGNFIIVAPSMNITVAEALFTTKSQVGWVAGTACPTGWALLVLIIIMFIGAQSFVRRRGHFEVVRPRLSGFDSMCDVVKSGVCRIRELFYFSHILYAPTLILLVIHGPSFWKWFVAPGAIFVVEKLYRFKWIRRLAYGRTFVQEGILLPSKVTHLVLDRPESFSFNPGDYVFINIPKIAKFEWHPFTISSAPEYRGHIWLHIRAVGTWTTKVNEYFDEKRKRWEATMPTLKSRRLAVSNFQFSPAELMIQERRMSQQVDAFRSSVRSRASASGSSLRSMRSRANTGGSSMRSISRRLQGQDAGAEENALSNHLLERERRMNHFNHVSNGGPNSTSGHAHEGHINIAFQIENDAAENEKESLPMAERKLQQGCNLLDVSRIDDILEEDEDSKSLVMGQSDSQDISTVEPNLNSEEPELSDNLLKLNTEKKQHIADSDHRDVPPEHKFDIENNKNGDNTEETAKVTTANSGKKDSECKNSENSHMDHGNEDVATSDESYERENSDSGNVSDDKESRTSMDKYKPSDGSTKEDRSEKSEKSDKSEDEEDDILPPDFKPPPLRRLGTSNSMTGHNRNRRMTLCQRRLSTWEAVSSGQNFAMTKQKPFIYNIDPAADTTLNELLEAEHAVLVAGGIGVTPFASILQSILLRYQREKNVCPKCNHAWLDELPCTLYNLKKVDFFWLCRDQSSFEWFVSLLAQLEMEQEDLDNLDDSIIDFHIYMTAAKTDMRSVLQQITFDLNHQVEARDAITGLRTKTQPGRPNWDEEFQKLKNLKKGRVTIFYCGPPGLAVVLGKKAAEYGFGFRKESF

>L.anatina_10_Duoxc g22171.t1 1

MTYRWQKVRLFNKHSIMKNKMPVAMEMQITFICLLVGTLNIVTATHAAFGTERQRYDGWFNNLANQRWGTRGCHLLNTVPQSYDDYTYQPSGQGRPAARDISNAVFNGSSGLPSYNNLTALMAFFGQLVTYEITYSSMPTCPVELVKVPIPKCDQDFDPKCSGREMMPFYRAAYDKRTGRSPNNPRKQINEVTSWIDGSFLYGTQEVWANCLRAMHDGRLKSVNGSREEEFPAMNHIRLPLDNFPSPHKHKLSDPETKWMFGHPRTHVHPGLTALGIVWFRWHNYMADRVTSMMNTTWADEVMFHKTRRWVIATMQNIIMYEWLPTLLNEKIPEYTGYKNYIQPGVTAAFHAAASRYLITLIPPGLYRRNDQCEFNKGGNFTKPWRLCNSYFESQDLLESYKGALDELLMGMASQIAEREDTVVVDDVRDKSPGPLHFSRHDVVVQTIMRGRDFGLGNYNSARRAFGLEEVDTWDALESLVNTTTGEEEKTVEQPGVTAAFHAAASRYLITLIPPGLYRRNDQCEFNKGGNFTKPWRLCNSYFESQDLLESYHGALDELLMGMASQIAEREDTVVVDDVRDKSPGPLHFSRHDLVVQTIMRGRDFGLGNYNSARRAFGLEEVDTWDALERLVNTTTNEENKTVQETVEKLRNVYNDSMEHLDVFAGGMAETGRNGPGPLFREILLDQFLRIRDADRYWFENKQNGLFTDEEIAQIKSIKFADIITNVTNIQPDWLQRDVFLRQQGDPCPQEKQLVIEDMEPCTDLQGWDYFAGSELWFIGSLVVLGLVPFFCILVAYLIYRVKKFRTKRVKRTLHMDKLRSMVRANAGNIAATEWTDPKDLPRSIELHFDDMTCGLLVLTASGTKIRHLDFSMSEEVNVWLSMNRGKETMLIQIPNEYDLVVMFDDEEDRMEFLRKFKSYLNDFNKNLQFFHDKQQNILENAVTKRKRQQLLERFFKTVFAEALTLDHQPRMETEAIEQGTAKEILNLELSKAEFAEALALKPSSLFVEQFFSMLDKDQNGYISFREFLYAVVLFSKGSCEDKLHMLFRMYDLNHSGSLNKEEIAALLRSLLEMANTDLTADEVDKLTETMFVNSGLEGKGSLRFEEFQQLLADDMTKLWDVCIDWKGTKVCFPKKRSNVDKEKVKFGNTGQDAFGATREKYHPIGAKMKNVLHFIENNRQHIFYLIIFYGICIGLFSERFYTYSVEKEHFGLRRIASYGVPVSRGSAAALTFCYSLILLTVCRNLITRLRETFLNLYIPFDSNVAFHKVIAWTALFFTCKYALTLDHQPRMETEAIEQGTAKEILNLELSKAEFAEALALKPSSLFVEQFFSMLDKDQNGYISFREFLYAVVLFSKGSCEDKLHMLFRMYDLNHSGSLNKEEIAALLRSLLEMANTDLTADEVDKLTETMFVNSGLEGKGSLRFEEFQQLLADDMTKLWDVCIDWKGTKVCFPKKRSNVDKEKVKFGNTGQDAFGATREKYHPIGAKMKNVLHFIENNRQHIFYLIIFYGICIGLFSERFYTYSVEKEHFGLRRIASYGVPVSRGSAAALTFCYSLILLTVCRNLITRLRETFLNLYIPFDSNVAFHKVIAWTALFFTLAHVVGHALNFYHISTQPVQHLCIFDEIFFKSEYLPTFAWWLLGTLTGMTGVLVTIVIFVIYIFATQCARQFIFNAFWATHKLIIPMYILTILHGSGRLLQLPYFWAYFVGPAILFTIDKLVSISRRKMEISVINAELLPSDVTFLEFKRPPNFEYKSGQWVRIACLSQGRHEYHPFTLTSAPQEDTLSLHIRAIGPWTWNLRNLYDPDMLRDGPYPKLFLDGPYGSGHQDWYQYDVSILVGGGIGVTPFASILKDFVHMSTIKNTFKIKCQKLYFIWVTASQRHYEWLIDILRQVEEVDKNGLVNIHIFITQFFQKFDLRTAMLYICEEHFTKVSGKSLFTGLRAKTHFGRPQFEKIFQSVQETSKVTKVGVFSCGPPGLTKGVEKACIESSKHSKALFEHHYENF

>L.anatina_13_Duoxa g3440.t1

MNGASAFIDGSVIYSNSKVWNNAMRTFVNGTMKSRDGDGLWPVLNTPSVPLANPPPAREHVLMAPERQWLLGDPRLFQHPAMLAFGVLFFRYHNKLAQELAAQNPAWDDETIFQEARKWVIATQQNIYAYEFFPAFTNTDLPAYSGYKEHVYPAVSHIFQSAAFRFGHSVVPPGFYRRDAQCNFFNTTTNTTNPGYPAVRMCNIWWHGDEPFEFGLEPVLLGMASQITEREDHILVDDVRGLVFGPIQHSRRDLGTLNIMRGRDNGLPDYNTARKAFGLAAVTNYSQINPWLYQQDPQALENLKTAYNDNIDKMDIFVAGLYESQPDRPLGELFLAVVTDHFTRIRDGDRFWFENTQNGLFNSSEIEQIKNTTLYDVILKVTNIQPGEIQRNVFFWNEGDPCPQPKQLNISDMAQCNDNLTHDFFTGSEVVYILVIILIGLLPFCFAGLVIVLHACKKIQNRRNQTSLKNNKVAVQLDDSIEEGENIIATQWRGSNSPMTVAIELHKGLVMVKDPVTGKTYRTVDVVKNGAVISKISNENGGRVICLHFPKEYDVVLQFSDEQKRERLSAMMTTFLKECNVKEELAFLPLQQLYAEAETKERRKIKVEKFLHHVFAQVYSKDDNKQKQTNNADLKNIFDLEMSREEFAEAMVLKPDSMFVDQMFNMVDKDGNGYISFREFLDVLVLLSKGSEDDKMRLMFDMYDLDGGGTMDRGEFTALLRSMIEMTDSSLSEEEMNVLLEGMFAAGGFENKEELTFEDFKTMLTQEKDVVNLSGGQLKSEIAKKEAASRPASAVSRPRSAEKLKVETDTAKAPTGVKKHFITFQKFVENNFLHIFYLALFFLITIAIFVERSYYYAVLNEAKGLRRMAGYGISVTRGAASSMSFTFSVVLLTMCKNTMNLLRTTWVNNYVPFDAAFGFHKVSAYTSTFIALGHTIGHCINFYHLSTQPPDFVGCVFSNTIWFRSDYLPTFGFYLFQTVTGLTGVVLVVLLTLMIVFATPWARKKVYTAFWTVHNLYVLLYALIILHGSAVLVQDPWFYYFYLGPMLLFALDKLVSISNKSVPVTVLKAEHLPSNVTHITLRKPRTFKNKSGQWARIAIVGHGDNEYHPFTITAAEHEDVIAFHIRAVGPWTTQLRQLMDPERLREGPYPKMYIDGPFGGVSEDWSKYEVAILVGGGIGVTPFAAILKHVSYLASIKAKINCKKIYFFWVTQNQRQFEWMIDIIREVEESDFDDLVTVNVFVTQFFGKFDLRTVMMFLCEKHFVSISERSLFTGLKAPTNFGRPDFQKVIRDISLQWPKVHKFGVFACGPPALTNSVSAACSHVNKTSSASFSFYEENF

>L.anatina_6_Duoxb g19435.t1

MGNATGASDTLRVAVLVACLLWGTSLGKESGFSPPSSRLDDMVEFQRYDGWYNNRDNPKWGAPGSTLVRQLPTAYADSSYDYSGPDRPHPRDISLAMKGRSGLASYMNRTVMLPIFGEVIVMETMKSVGAHCPIELDPIRLPKCDHEYDPNCTGKKNALTSYIDGSFIYSKKKAWADSLRSFRGGKLKSRDQGKFPPYNTVNLPFLNPPPPREHKLLPRKRMWLLGEVRSFQHPGILTFGVLFFRWHNYLADMFHKRHPEWDDEQLYRAARRRLIATLQKIAMYDFFPAFTNETLPKYTGYNDMINPGISHVFQSAAYRFGHSISPAGFYRRNRNCEFYNTTTVTKFTGYPGLRLCNSYFVSDESMEFGLEPMLLGMSSQITEREDHILVDDLRGKLFGPLHNSRRDLAMINVLRGRDNGLPDYNTARRAYGLPVIEKIKSVYGGNLDNLDLWVGGLYESRPSGPGPLFMEAIRNQFIRIRDGDRFWFENRLNGLFTDEDIDEIMNTTLYTIMLKVTDIQPGEVQENVFFWHNGDPCPQPKMLEASDMEECGPNQVYDYFQGSEVPFILIVCSLIALPIVCAVLIKILLICRRKDHRRKQAIYYINDKPALILTPRPLARAAKKKSIERLQNAELALEYIDPKSRRWVILDVQESTEGPFVQLFDTKGTQLRQIEIQKFAQLKQTISSDHGKKALLLHVPHEYDLVLYFEKPEDRNFVTQLLHDVAERHDKRCIEDYKPFNKMLSCAETKAKRNVKIEKFLRDVFEKLFRTDHGHSHGGNKVKGRRIERKEMELLAMEITKEELGEALSLQMNASFLVDVFRIADPQNHGYITLQEFVDVLIILSKGSDDEKLTMMFQMYDHTGKGFVKKEDLFNVVKSMADISDSPIQAYEIQELLDSMFETAGLRGKTFLDINEFRQLFAKVKGKNVRGRLLSGFDPIAKGVSPNISNITRQSDKKSETSLKVDVLDVSTSHNAADENPIYNALLELQTHMENNYLNIIYLSFFYLIAIALFIERSMADISDSPIQAYEIQELLDSMFETAGLRGKTFLDINEFRQLFAKVKGKNVRGRLLSGFDPIAKGVSPNISNITRQSDKKSETSLKVDVLNVSTSHNAADENPIYNALLELQTHMENNYLNIIYLSFFYLIAIALFIERSYRYAIEFEHAGLRRMVGHGITLTRGAASAMCWTYSVQLLTMCRNTINALRGTVVAAYIPSDVHVFTFHMIVGWTSFGISVFHFVGHMFNFYHLTIQPPFFVKCVFSNTIYYSSTFLHTFQFYLLGTVTGMTGVLLTGVMGIMAVFALPWARRKLYNIFWRVHKLYIILYALIILHGSAVLVQEPWFYFFFLGPALLFVLDKTVSLSRFSHPVDVIRSDILPSDVTFLEIHKPTGFSFKAGEWIRLAIKGYNEFEYHPITIGSAPHEETLKVYIDGPFGSASQDWSNYDVTVMVGGGIGVTPFASILKHIIFLAQTDGRISCTKIYFVWVSTDQHQFEWMIDIIREVEEHDLQALVHISLFITRFYKKYDLRTLLMYLCERHFIRVSGRSLLMGLQSPLYFGHPNFDLLLKSVGKNHSSVKRIGVFSYGPPQMVKSVAKACFERNKTSKMIYEHHEEVY

>L.anatina_2_Duoxd g5129.t1

MSVENDGSRSKRMSSTPGFKVETTKQKAPKTKTQKRYTAVLRYFENYRFHIFWSVLYSLVCIGIFVERAYYYSIEREHAGLRRIAGFGVTVTRGAASGMMFTFACLLVTMCRNLITFLRETVAHRFIPFDSAITFHKFIAAWALFFTAMHIIGHGINFYHISTQLPGDVTCYFRDFFRATHQLPTFTYWCWNTITGFTGVLLTLLVILMYVFATQYSRRHCFQAFWITHNMFYLLYILMVMHGAGRLVQPGFTHYFILGPLILFALDKLVSVSRNKVEIAVLKAEALPSDITFLEFKKPTSFEYKSGQWVRIACITMGENEYHPFTLTSAPHEDTLSLHIRAVGPWTMNLRHTYDPSVLKEHAFPKLYLDGPFGEGHQDWYKFDVSVLVGGGIGVTPFASILKDLVHRSAIGAKFTCKKIYFLWVTRTQKHFEWLTDIIREVEEKDRNGLVSVHIFITQFFHKFDLRTTMLYICERHFQKISERSLFTGLRSITHFGRPQFENFLDSLQEEHPDVGKIGVFSCGPPAMTRNVETACTNLNKYDGAAFIHHYENF

>L.anatina_3_NOX2 g5253.t1

MASRLMNEAPKWVVLIIWLLTNVGLFIGYFFYYYNGEKFFYIRKIVGLGLPFARGAAACLNFNCMLILLPVCRNLISFLRKSCTCCKRNMRRQLDKNITFHKYIAYMICLHTAIHAGAHCFNVEFFVDAQSPTINKDEAALLTALSSIAGGNGTAAINPVRLPNASPVLEALKLIAGVTGIVITLALIVMVSSSTDFIRLHYFEAFWFTHHLFIVFYIALVVHGIQGVVTFQENVSEHNPEKCSQEPLDWGKEGKCPVPRFQNGTAGTWKWVLAPMIFYFIERCIRFFRSQQKVTITKVIKHPSRVIELQMQKAGFQAEAGQYVFLHCPSISKLEWHPFTLTSAPEDDYFSLHIRLVGDWTNALAGACHADSRGDQKGSSKLPSIAVDGPFGTATEDVFRYQVDVFVGTGIGVTPFASVLKHIWHKCNNSNFETKLTKVYFYWICPDTNAFEWFTDLLSSLEVQMAEQGKADFLDYNIYLTRGWDSDQAKNIMLQDEEATDAVTGLQKKTNYGRPNWDHIFQDIAEQHVDTRIGVFFCGPKALSATLHKMSNRYSSPNPNGTHFVYNKENF

>C.teleta_1_NOX5 CapteP147607

DEETAWLKHMEKQFKEIAGEDGSIDLDEFKRALKVKDSFFADRFFALFDEDGSGSLELEEVLNGLRQITKGSRRDKLKFLFDIHDVDSNHVRCGCVTGSGCIDHSEMKAVLESCVTESALKISDQDIEDLTTILFEAADADDSGSITFEEMITEFEKHPEALDSLTISAANWLKPPVQATKHNKEETWRRFFTKRFVVNNFRLISFLSVFALVNAVLFAFNAWRYWGANGYVIIARGCGMCLNFDCAFVVVLMLRYTLTWLRMTALRFILPFDESVMLHKVVGYVICILTVLHTVAHLINIDTGVPSNSTAAPLPEYWEYLFTFKSKIGFIGPGFGYLSGVILDVILLIMFLCSLSFVRRTGHFQIFYWSHVLYLAFWALLIMHGPMFWYFFILPGILFVIEKIYSLKIVKIARHGHMYVTEVNLLPSKVTHLVISRPAKFVYEPGDYIFINIPDIAANEWHPFTISSAPEHRGEFWLHIRGAGYWTNRLHNFFSNYDEGIDDMDGLQSPPKHTSLKHIRVAREGCPSECYKNQRRRKFVKVKCFVDGPYGSPTREIMNTEHAVLVASGIGVTPYASILQSIMLRRKGRRVTCPCCDKSFYPDVPTELMGIKKVDFIWINRDQKCFEWFVSLLTQLEAQECEDGTVDRFLEMQMYMTAAASKNDMKGLGLQMALELLHAKRGTDLLTGLKTQTKPGRPDWREIFTKISAEKKGSVKVFFCGSAALAKQLNLQCDLHGFEFKKESF

>C.teleta_4_NOX5 CapteP147619

MTLVEQDEDTRWLAWIKKQFEGIAGDDQQIHLAEFKGAIKAKEPFFVERIFALLDADGSGTIDLDELMEGMTKLTKGDQMDKLRFLFDIFDVDGVGTIDPEELKTVLRSCMNESSMKLSEESLHALTMALFESADDDDSGEITFEELVGELSKHPGVMDNLTISTSHWLRPPEQEKVKKKPGCSRYFTKRYLQNNTRKVIFLLVYFIINIGLFILSAYRYRKFNWAVIIARGMGMDLNFNCSFIVVLMLRRFLSWLRTTTLGNYLPMDQSILIHKLVGFMIAFQVTVHTVAHLINIRECSAVANPSKLWLFTLQSRIGYIGPGFGYLSGVILDVILFIMVICSLGFIRRGGHFKVFYWTHQLCLLFWIFLIMHGPIFWCFFLVPGLFYLLERVLGSRLMKLYRWGRIYATRVHLLPSRVTQLVITKPPNFRYRPGDYIFLNIPDIAANEWHPFTISSCPEMEDEIWVHIRSAGHWTGKVYDLFESLEEVSDFDDIEKGRDFINKGKRFTRDFYEPALHTKSTNSLVFVDGPYGTSSREIFDSDHAVLIASGIGVTPYASILQSIMHRYTMSKLTCPNCHKDWYCDRMDRKMKLKQVDFIWINRSQNSFEWFVRLLAQLEMQQLDAGTEDFLQMQMYMTSAMAKNDVKGLGLQMAMEILHKKDNKDVMTGLKTRTQAGRPDWPQVFSKIADANKGRVKVFFCGAPSLAKSIKGHCEKVGFTFSKENF

>C.teleta_3_Duoxa CapteP159209

MIRLFFLLITGSLVLAEVPHVEHPPFDGWYNNWAHPDWGAAETTLYRRLAPAYSDGVYEPAGAHRPNPFEISDAVFSGKTGHASVKNRSALLVFFGQQVVEEVLDAQRPGCPPEYFNIKIPKNHAQYDKDGRGGREMPFLRSRYDQNTGYSPHVPREQLNEISAFLDGGLVYGPNKAWADALRSYKGGRLAAYNDNDASKPNFPAENDIRLPMANPAPPFDHKLKPIKRFFKLGNPRGNENPFLLTFGVLLFRWHNHQAAQLQANHPDWSDERLFLEARKLVIAHHQKIVMYDWIPAWLGTEVSEYKGYNPSVHPGIAHVFQSAAMRFGHTLVPPAVYRRNRNCVFRNTSDISGFSGHSALRTCNTFWNSPVSLHQTDIDELLMGMASQITEREDNIVTPDLRGDVFGPLEFPRRDLMAVNIQRGRDHGLPDYNTARKLYGLKPITDWTDINPDFFTNSPDVCGPGVLEKIRNLYNNSLDDVDIWPAGLLETTANGPGELFRTIIKDQFERIRDGDRFWFENRDNGLYTDEEIDAINKTTLRDIIIAVTDIGENDLQENIFFVGDDVPCKQPDQLGDLMTTGSIGECKRPRDAPEVQDGVVFDYFWGSEVSYALTFIAYHSLLAVTVLVLILLARRRDKEVQKKMAMTREKTRKRTKVPSKQISVITASEWIGKKQGHRNVIIKLESSKKHILVTEASSRKSKVLRTIDLNLLLKNRQTGVELILSADGKMTMMALKVPKEYDLVLVFDDQRDRDQSQVELERFMFKAGLGRTLSTVPEKDLLRDAHSVQDRKSLLTQFFQTVLANAFHEEESENREDLDFVKAKEILETELTRYEFAEALSLKPDSLFVNSMFKLVDKNENGYISFREFLDFFVIFSKGNADDKVKLMFDMYDLDNTGTLTKEEFRTMLTSMVDLANEKVEEKDIETLIGSLFSSAGLEGKPAITFTDFKKILKNYESELNYTSLNLAGISPSEVYLYNNNDCAGTGVPMNAQPRRENASSRARRTVILSYDNLDLSSASTGRTANKENRRLSTIQEVKTIKKTAPKGKFALFCNDILRYFVNYKLHIFWCTLYTLITLGIFIERAYYYSIEREHAGLRRIAGYGVTVTRGAASGMMFTYASLLVTMCRNTVTFLRETFLHKYIPFDGAISFHKYIAYLALFFTLLHIIGHCINFYHISTQTPGDLICLFRNFFHATDDLPKFHRWCWNTLTGVTGVLLTINMFVIYVFAVQYARRHTFKAFWFTHNTYPIFFILMILHGAGRLVQPPFTHLFILGPVVLFTLDRLVSINRKKVEIAVITAELLPSDVTYLQFKRPLNFEYKSGQWCRIACLVQGSNEYHPFTLTSAPHEENLSLHIRAVGPWTMNLRRTYDPDHVGEHSWPKVFLDGPYGEGHQEWYRYEVAVLVGGGIGVTPFASILKDIKYKSRTNAQFVCKKVYFLWVTRTQRQFEWLTDIIREVEDSDLVSVHVFITQFKEKYDIRTTMLYICERYFQKISERSLFTGLRSITHFGRPEFKQFFQMLQVEHPNNSEFGVFSCGPPPMTSSVEKACAHVNKTSTNVLFQHHFENF

>C.teleta_2_NOX2 CapteP223007

MADICELMNYMLITNQLFISLQTLFLIINIVLFVVYFMKFYNGKQYYYLNQMLGLSLPFARASAGALNFNCMLILLPVCRNLITLMRGSCQRCCYRNTLRQLDKHIMLHKLCAYTICFWTAVHYFAHCFNFERFILAWSSPNNSTKELLATLSAIKQDGDQNWVNPIRSPLTNPMLELFKNIPGYTGVLITIPLIFIVTSSTELIRRSYFEVFWFTHHLFVVFFIALVTHGLGGVVRMQTNLADHDPEYCSLGDRYKYWGVLPECPLPNFQGGGAQTWKWVIGPMVLYVIERIVRFVRSEQKVTVVKVIEHPSRVVELQMQKSGFTTEPGQYVFLKCPSISHLEWHPFTLTSSPQEDFFSVHVRIAGDWTAKLAELCCHDEYQVHDSSTLPKISVDGPFGTCSEDVFRYQVDVLVAAGIGVTPFASVLKDIWFKKCDADHQMKLRKVYFYWICPDTNAFEWFVDLLKCVEDQMIEMNQADFLEINIFWTRGWSARQANIIGKTVTEETDVITGLMHKTKFGRPNWDLEFERIAQSNPDTRVGVFFCGPKVLSSTLHKMSNKYSQSRASGTNFVYNKENF

>C.elegans_2_Duox WBGene00000253

MRSKHVLYIAILFSSIFGGKGIQQNEEFQRYDGWYNNLANSEWGSAGSRLHRDARSYYSDGVYSVNNSLPSARELSDILFKGESGIPNTRGCTTLLAFFSQVVAYEIMQSNGVSCPLETLKIQVPLCDNVFDKECEGKTEIPFTRAKYDKATGNGLNSPREQINERTSWIDGSFIYGTTQPWVSSLRSFKQGRLAEGVPGYPPLNNPHIPLNNPAPPQVHRLMSPDRLFMLGDSRVNENPGLLSFGLILFRWHNYNANQIHREHPDWTDEQIFQAARRLVIASMQKIIAYDFVPGLLGEDVRLSNYTKYMPHVPPGISHAFGAAAFRFPHSIVPPAMLLRKRGNKCEFRTEVGGYPALRLCQNWWNAQDIVKEYSVDEIILGMASQIAERDDNIVVEDLRDYIFGPMHFSRLDVVASSIMRGRDNGVPPYNELRRTFGLAPKTWETMNEDFYKKHTAKVEKLKELYGGNILYLDAYVGGMLEGGENGPGELFKEIIKDQFTRIRDGDRFWFENKLNGLFTDEEVQMIHSITLRDIIKATTDIDETMLQKDVFFFKEGDPCPQPFQVNTTGLEPCVPFMQSTYWTDNDTTYVFTLIGLACVPLICYGIGRYLVNRRIAIGHNSACDSLTTDFANDDCGAKGDIYGVNALEWLQEEYIRQVRIEIENTTLAVKKPRGGILRKIRFETGQKIELFHSMPNPSAMHGPFVLLSQKNNHHLVIRLSSDRDLSKFLDQIRQAASGINAEVIIKDEENSILLSQAITKERRQDRLDLFFREAYAKAFNDSELQDSETSFDSSNDDILNETISREELASAMGMKANNEFVKRMFAMTAKHNEDSLSFNEFLTVLREFVNAPQKQKLQTLFKMCDLEGKNKVLRKDLAELVKSLNQTAGVHITESVQLRLFNEVLHYAGVSNDAKYLTYDDFNALFSDIPDKQPVGLPFNRKNYQPSIGETSSLNSFAVVDRSINSSAPLTLIHKVSAFLETYRQHVFIVFCFVAINLVLFFERFWHYRYMAENRDLRRVMGAGIAITRGAAGALSFCMALILLTVCRNIITLLRETVIAQYIPFDSAIAFHKIVALFAAFWATLHTVGHCVNFYHVGTQSQEGLACLFQEAFFGSNFLPSISYWFFSTITGLTGIALVAVMCIIYVFALPCFIKRAYHAFRLTHLLNIAFYALTLLHGLPKLLDSPKFGYYVVGPIVLFVIDRIIGLMQYYKKLEIVNAEILPSDIIYIEYRRPREFKYKSGQWVTVSSPSISCTFNESHAFSIASSPQDENMKLYIKAVGPWTWKLRSELIRSLNTGSPFPLIHMKGPYGDGNQEWMDYEVAIMVGAGIGVTPYASTLVDLVQRTSSDSFHRVRCRKVYFLWVCSTHKNYEWFVDVLKNVEDQARSGILETHIFVTQTFHKFDLRTTMLYICEKHFRATNSGISMFTGLHAKNHFGRPNFKAFFQFIQSEHKEQSKIGVFSCGPVNLNESIAEGCADANRQRDAPSFAHRFETF

>C.elegans_1_Duox WBGene00018771

MAAENFYNVNNFQSLPLEIKVQFSKETLFSALQQEAETQRYDGWYNNLANSEWGSAGSRLHRDARSYYSDGVYSVNNSLPSARELSDILFKGESGIPNTRGCTTLLAFFSQVVAYEIMQSNGVSCPLETLKIQVPLCDNVFDNECEGKTTIPFYRAKYDKATGNGLNSPREQINERTSWIDGSFIYGTTQPWVSALRSFKQGRLAEGVPGYPPLNNPHIPLNNPAPPQVHRLMSPDRLFMLGDSRVNENPGLLSFGLILFRWHNYNANQIYREHPDWTDEQIFQAARRLVIASMQKIIAYDFVPGLLGEDVRLSNYTKYMPHVPPGISHAFGAAAFRFPHSIVPPAMLLRKRGNKCEFRTEVGGYPALRLCQNWWNAQDIVKEYSVDEIILGMASQIAERDDNIVVEDLRDYIFGPMHFSRLDVVASSIMRGRDNGVPPYNELRRTFGLAPKTWETMNEDFYKKHTAKVEKLKELYGGNILYLDAYVGGMLEGGENGPGEMFKEIIKDQFTRIRDGDRFWFENKLNRLFTDEEVQMIHSITLRDIIKATTDIDETMLQKDVFFFKEGDPCPQPFQVNTIGLEPCAPLIQSTYWDDNDTTYIYTLIGLACIPLICYSIGHYMVERRIRIGHNSACDSLTTDFSTESPKVNVYKVNALEWLQEEYIRQVRIEIENTTLTVKKPRGGILRKIRFETGQKIEVFHSIPNPSAMHGPFVLLSQKNNHHLVIRLSSDRDLSKFLDQIRQAASGINAEVIIKDEENSILLSQAITKERRQDRLDLFFREAYAKAFNDSELQDSETSFDSSNDDILNETISREELASAMGMKANNEFVKRMFAMTAKHNEDSLSFNEFLTVLREFVNAPQKQKLQTLFKMCDLEGKNKVLRKDLAELVKSLNQTAGVHITESVQLRLFNDVLHKSGVSDDAEYLTCNNFDALFSEISDVQPIGLPFNRKNYNSHIKEPSCHTSFPIVDHSTPAPLSLIQRICAFLETYRQHVFIIFCFVAINIVLFFELFWHSRYLNEDRDLRRVMGAGIAITLSSAGALSFCMALILLTVCRNIITLLRETVIAQYIPFDSAIAFHKIVALFTLFWSTLHTIGHCVNFYHVGTQSDRGLACLFQETFFGSDVVPTLSYWFYGTITGLTGIGLVIVMSIIYVFALPKFTRRAYHAFRLTHLLNIGFYALTILHGLPSLFGSPKFGYYVVGPIVLFVIDRIIGLMQYYKSLDIAHAEILPSDIIYIEYRRPREFEYKSGQWITVSSPSISCTFNESHAFSIASSPQDENMKLYIKAVGPWTWKLRSELIRSLNTGSPFPLIHMKGPYGDGNQEWMNYEVAIMVGAGIGVTPYASTLVDLVQKTSSDSFHRVRCRKVYFLWVCSSHKNFEWFVDMLKNVENQAKPGILETHIFVTQMFHKFDLRTTMLYICEKHFRATNSGISMFTGLHAKNHFGRPNFKAFFQFIQSEHKEQSEIGVFSCGPVNLNESIAEGCADANRQRDAPSFAHRFETF

>C.intestinalis_2_NOX4 ENSCINT00000026975

MQLKNYLVNDGLRLFIWVAWISINASLFYFTFMYYYNGIQFYYLHQMLGYGLCISRASAACINLNSSFILFPMCRGLVTFMRGLPRGVGRQVRRLLDRGRSFHILCGYILCLLAGVHCAAHAYNAVYFSKYYNSRYKDLNVAKYSNQNPLLMLVTSLSGITGILLVISLVVISAFASRPIRRNNHNKFWKTHHIFIVFYALIFIHAMDGVIKYQTNVDQHKPGCFIIIDQTNNTSNISMVQPEPEPFPNHPSMVKPKMVAVPEPPHGEPFPNMKLASKMPSKTDMKFMNNTAPQPHHVIMPEPEPMPHKGAHGAPNTTRIFVHGSWVEVMECVQPPPKFSSCGQEAWLWLCAPLIIYVIERIGRHFRSSHDVTTIVKFIEHPCDVIELRLYRNGFSAKPGQCIWVRCPQLSKVESHPFSLTSVPSKDDPTFGIHVKLRGDWTEELRDLMVRELNPVVEISKDKIIAGGNFLEKDDELSNRGETEYKRNPNNSQYLATMASPNQSINKPCLVHNFRTQETDFQMHCTLSNSTISLYDNHEMPTHQENIDDQTCKKTSSTCLSQYSLESESQRNLPKSTTLHSKDQQNLQNETTLYSEDQRNVPKSTALVSEACSTKNDIHLPCNQTVSKQLPILCVEGPTGGAMEDIFKYKISMCVAGGIGVTPYASVLNALLKDEDLFSRMKLKRLYLIWSCKDPRSFSWFASLIRDVQIVLWKRNCPDLLSVRLHITGSNTLQSEESGDQLLSDIQGCHVAYGRPDVTQVFEEIRTAAQYQRSTVGVFCCGHRLLVSSVKHHCLKTKSSKVKFLFNKEAF

>C.intestinalis_7_Duoxb ENSCINT00000008209

MLRIIISFVIVINWGLCTQGQQEIGKEFPPFNGWYNNRGNPSLGIPDSSLTRSLPPHYKDGVYEPSGWERPNVRSISNLIFSGPQGLPSTNNKTALFLFFGEHVMQDILDTSRPGCPPEYFNIMLNASDSHFSTTEMPYERSSYQASNTGYSPNHPREQINAVTSYLDGSQIYGHTKAWSNNLRLLRSTATDCRGELASLDDQGEFPALNNVGLPLDNSWTTKDHVIQSAKRFYRVGSRRGNENPFVLTIGITWFRHHNWLARNIRDSNPNWSDDDVFNEARIQNIAMYQKVLMYEWLPGLLGTCSSLNQTYSSGTSASVSDIFEGAARHFLSTITPPGVFVRSKFNESSGSCEFRTTSATSPALSLCNSYWEPAETFKVASIDELTMGMASQIAELEDNVITPSLRHNYYGSRRFSRRDLMATILQKGRDHGLPDYNVAREEFGMQTKATMMDINQNLFQSNLQLLDDLTSLHDGKLSKLDIFTGGLLETTGGSPGELFRYVIADQFLRLRNGDRFWFENVKTSLLSSTKLNEILNTTFRDVILRTNPTINGSFDIQDNPFVWATGDSKLFCPQPYQLTENVLEECTPLQRYDYFTSSEVSFPLSFAFFGVFVLITLLVMYLVGRQRMKSALKTKKDITTKKRATIETSIASGNVTICTEILGENVRQIQIILRPENLILIKAVIGNQNLRKIELIQQQDIKIYCSDGTDRRTLIVRLQEDTYDLVLSFNDSYELDEFFNELHNFVKPLGLKCITFAIPEKHIMEEARTKEMRDKQLQNFFRTALSQVLTQAMNIESEDLDRPKRKTLKDLMKVNITKSEFAEYLKLKEDSLFVEQMFLVADSDEDGTISFREFLDIIVLFTKGTPKEKAQLMFNMYDLDKSGGLSKEEFTTMLKSMMEMVNSSADVDNIDSVVDDMMRANGFSSKDSLDLNDFLMLLGQYSDISNQQSTKSSLYIISYSDSIIGNGHKTYQAKRQSKYHRYSKRFKEGYVRLHCIKSMFTKLYITHRPQTPQRRLTRKVKTVREDYKTKSYEKFFVALVKLTEHYANHIFCLSLYSLITAGVFLNAFFYSKNATGLYGIGGPLMALARASAAALMFNFSTLLLTMCRNIITFLRETFLHRFIPFDSAVTMHRIVAWMALAFTALHILAHGINFYSIVTQSPDDMACLFRDMWYPSDYIPTFVFWLFQTITGITGVILTLALIVMYVFASNYARRMIFNWFRWTHKLGYLSLYFFSFVHGSGMLISSPQFYYYFLVPGILFTLDKVYTYSRKKAYISVVRAELFPSDVTHLEFKRPKNFDYKAGQWVRIACLAQSSSEYHPFTLSSAPHEDTLKLHIRAVGPWTRNLRNIYDPNVLRDSPYPKLFLDGPFGEGHQDWYKYEVSVLVGGGIGVTPFASILKDLVNRSQSGVAITCKAVYFIWVTRDQNQYEWLTDIIQEVEGKDKKQILNTHIFITQFPQKFDLRTKMLYICEENFQKIAGKSLFTGLRAITHFGRPDFPDFFVTLGEEHSSVETFGVFSCGPPPMTEGVEKACAKLNKYEGPTFSHHFENF

>C.intestinalis_6_Duoxb ENSCINT00000008222

QQEIGKEFPPFNGWYNNRGNPSLGIPDSSLTRSLPPHYKDGVYEPSGWERPNVRSISNLIFSGPQGLPSTNNKTALFLFFGEHVMQDILDTSRPGCPPEYFNIMLNASDSHFSTTEMPYERSSYQASNTGYSPNHPREQINAVTSYLDGSQIYGHTKAWSNNLRLLRSTCNELASLDDQGEFPALNNVGLPLDNSWTTKDHVIQSAKRFYRVGSRRGNENPFVLTIGITWFRHHNWLARNIRDSNPNWSDDDVFNEARIQNIAMYQKVLMYEWLPGLLGTCSSLNQTYSSGTSASVSDIFEGAARHFLSTITPPGVFVRSKFNESSGSCEFRTTSATSPALSLCNSYWEPAETFKVASIDELTMGMASQIAELEDNVITPSLRHNYYGSRRFSRRDLMATILQKGRDHGLPDYNVAREEFGMQTKATMMDINQNLFQSNLQLLDDLTSLHDGKLSKLDIFTGGLLETTGGSPGELFRYVIADQFLRLRNGDRFWFENVKTSLLSSTKLNEILNTTFRDVILRTNPTINGSFDIQDNPFVWATGDSKLFCPQPYQLTENVLEECTPLQRYDYFTSSEVSFPLSFAFFGVFVLITLLVMYLVGRQRMKSALKTKKDITTKKRATIETSIASGNVTICTEILAVIGNQNLRKIELIQQQDIKIYCSDGTDRRTLIVRLQEDTYDLVLSFNDSYELDEFFNELHNFVKPLGLKCITFAIPEKHIMEEARTKEMRDKQLQNFFRTALSQAMNIESEDLDRPKRKTLKDLMKVNITKSEFAEYLKLKEDSLFVEQMFLVADSDEDGTISFREFLDIIVLFTKGTPKEKAQLMFNMYDLDKSGGLSKEEFTTMLKSMMEMVNSSADVDNIDSVVDDMMRANGFSSKDSLDLNDFLMLLGQYSDISNQQSTSNKNIPLTQCPVYLQSSLYIISYSDSIIGNGHKTYQAKRQSKYHRYSKRFKKFFVALVKLTEHYANHIFCLSLYSLITAGVFLNAFFVVYSKNATGLYGIGGPLMALARASAAALMFNFSTLLLTMCRNIITFLRETFLHRFIPFDSAVTMHRIVAWMALAFTALHILAHGINFYSIVTQSPDDMACLFRDMWYPSDYIPTFVFWLFQTITGITGVILTLALIVMYVFASNYARRMIFNWFRWTHKLGYLSLYFFSFVHGSGMLISSPQFYYYFLVPGILFTLDKVYTYSRKKAYISVVRAELFPSDVTHLEFKRPKNFDYKAGQWVRIACLAQSSSEYHPFTLSSAPHEDTLKLHIRAVGPWTRNLRNIYDPNVLRDSPYPKLFLDGPFGEGHQDWYKYEVSVLVGGGIGVTPFASILKDLVNRSQSGVAITCKAVYFIWVTRDQNQYEWLTDIIQEVEGKDKKQILNTHIFITQFPQKFDLRTKMLYICEENFQKIAGKSLFTGLRAITHFGRPDFPDFFVTLGEEHSSVETFGVFSCGPPPMTEGVEKACAKLNKYEGPTFSHHFENF

>C.intestinalis_3_Duoxb ENSCINT00000008215

FNGWYNNRGNPSLGIPDSSLTRSLPPHYKDGVYEPSGWERPNVRSISNLIFSGPQGLPSTNNKTALFLFFGEHVMQDILDTSRPGCPPEYFNIMLNASDSHFSTTEMPYERSSYQASNTGYSPNHPREQINAVTSYLDGSQIYGHTKAWSNNLRLLRSTGELASLDDQGEFPALNNVGLPLDNSWTTKDHVIQSAKRFYRVGSRRGNENPFVLTIGITWFRHHNWLARNIRDSNPNWSDDDVFNEARIQNIAMYQKVLMYEWLPGLLGTCSSLNQSQCLNVTPYTAYSSGTSASVSDIFEGAARHFLSTITPPGVFVRSKFNESSGSCEFRTTSATSPALSLCNSYWEPAETFKVASIDELTMGMASQIAELEDNVITPSLRHNYYGSRRFSRRDLMATILQKGRDHGLPDYNVAREEFGMQTKATMMDINQNLFQSNLQSNGTVIAFSRSFRLLDDLTSLHDGKLSKLDIFTGGLLETTGGSPGELFRYVIADQFLRLRNGDRFWFENVKTSLLSSTKLNEILNTTFRDVILRTNPTINGSFDIQDNPFVWATGDSKLFCPQPYQLTENVLEECTPLQRYDYFTSSEVSFPLSFAFFGVFVLITLLVMYLVGRQRMKSALKTKKDITTKKRATIETSIASGNVTICTEILGENVRQIQIILRPENLILIKAVIGNQNLRKIELIQQQDIKIYCSDGTDRRTLIVRLQEDTYDLVLSFNDSYELDEFFNELHNFVKPLGLKCITFAIPEKHIMEEARTKEMRDKQLQNFFRTALSQVLTQAMNIESEDLDRPKRKTLKDLMKVNITKSEFAEYLKLKEDSLFVEQMFLVADSDEDGTISFREFLDIIVLFTKGTPKEKAQLMFNMYDLDKSGGLSKEEFTTMLKSMMEMVNSSADVDNIDSVVDDMMRANGFSSKDSLDLNDFLMLLGQYSDISNQQSTILAGNISVLNKYALGNGHKTYQAKRQSKYHRYSKRFKEGYVRLHCIKSMFTKLYITHRPQTPQRRLTRKVKTVREDYKTKSYEKFFVALVKLTEHYANHIFCLSLYSLITAGVFLNAFFYSKNATGLYGIGGPLMALARASAAALMFNFSTLLLTMCRNIITFLRETFLHRFIPFDSAVTMHRIVAWMALAFTALHILAHGINFYSIVTQSPDDMACLFRDMWYPSDYIPTFVFWLFQTITGITGVILTLALIVMYVFASNYARRMIFNWFRWTHKLGYLSLYFFSFVHGSGMLISSPQFYYYFLVPGILFTLDKVYTYSRKKAYISVVRAELFPSDVTHLEFKRPKNFDYKAGQWVRIACLAQSSSEYHPFTLSSAPHEDTLKLHIRAVGPWTRNLRNIYDPNVLRDSPYPKLFLDGPFGEGHQDWYKYEVSVLVGGGIGVTPFASILKDLVNRSQSGVAITCKAVYFIWVTRDQNQYEWLTDIIQEVEGKDKKQILNTHIFITQFPQKFDLRTKMLYICEENFQKIAGKSLFTGLRAITHFGRPDFPDFFVTLGEEHSSVETFGVFSCGPPPMTEGVEKACAKLNKYEGPTFSHHFENFMGGTQSQYMCDEHVAAHIKSRPFLEQELNSCVQGKAKLASFDVVDGNGVISYTLENGATVVECKSKLDEFFKKFMTFSESYDPQKIPFHLSSHMLHAGVDCTVKWDNPNHSVQITGNKNDVKEVIKKLREYQNPTLPPAVSTAPPPPTVTVNNNLPPKSFGFTYPLAQFQTTSVQPNTTTSSVTTANAPSNAPINPLPTPSPPSGPSTSSSAHQPPETFTYDIEIGFVSYFLV

>C.intestinalis_4_Duoxa ENSCINT00000001891

EYPSFNGWYNNLANPSSGATETALTRRLKSHYEDGVYAPSGSGRPNPRTLSVEIASGPSGLHSYDNKTTLFVYFGQHIIKEIVENSRQGCPPEYFWIPVPEGDEIFDPRGDGNIRLPYHRSPYIASNTGYTPNHPRDQLNEVTSLLDGDSIYGSTKAWSGELRLRKQKNPTDCRGELRSADDDGKLPMRNTIGLPLQNPVVPVYHEMRNAKRLFATGSRTGNENPFLLTIGVTWFRHHNWLARQLRDNNPDWSDEHVFMESRTRNIATYQKVFFYEWLPILLGSCPSCEEIPRYTGYHASVPVGVSNVFQSAAMNYLDTMVPPGGFIPKTFDSSKHGCNSSNSDEMDKWRTCNTYWNSDELFENATPFNIVYGMMGQMAESEDHIISEDLRNSFHGSMEFSRTDKVATIIQRGRDHGLPDYNTAREDLGLKKKETIRDINPDLFKKDEKLLERLHSLHGGSPSNMDIYVGGMLESKDGRPGELFRSIILEQMVRLRDGDRFWFENLNNGLFTVDQVNGIFNVTFLDVLLQKTKNMLKSNLNTIVLFTESNKHICRQPYQMTGDNMTSCTTMQRQDDFNHNQISFLAIFCGVACVIFMIYVGSWKEDKRNKEKTKTTSTYSEQDAFTAKEILGRKRHERKDVQETNTSFDLQKNTENNTTHITHITESEEVVIMYTDSGKRKKMLLRCAKRTYDLILAFTGSSERDIFLTKLRLFFKQNDILWKEANTKDERDTELSNFYQAVIAQNRNYNSMRMEFGIQISSFPENLVDIKLSQEEFASLLQMKSSSLFVQHIFTTADEDQDGFISFHDFRKIIVLFVKGSPNEKLRLLFDMFDLNQNGSLTKQQFKEMCVCTADTYQSTVDQNVLENVLSKVVPLKNDEEIQYLKFDDFKKLMDLPEMDEAMLNLTAPGRNERPNRGNFIQSPNVSTVENVMPDVYFQNSSKFRLAYRAIRRLFECYALHIFWTSLYIWITIGVFLWAFNSVRSNKAAGLGVIAGNALPLARASAAALMFNISTLLLTMCKNILTFLRETQLHLYIPFDAAVAFHKLVAWMALFFTALHIIAHGINFYSIQTQTPSDLLCLFRDLWFPSDYQPTFVFWCLQTLTGNTGVLLTIIFIVMYVFSLDYPRQVMFNWFQWIHFFGYISVYFFTVLHGSGMLIQIPSFYYYFLVPAILYTFDKLYSVYRKKLQLPVIKAEILPSDVVYLEFVRPSDFYYKAGQWVRIACVGLSKWEYHPFTLSSSPDEETLQLHIRAVGPWTRNIRNIYKEGEPYPKLYVDGPFGEGHQDWYKYEVAVLVGGGIGVTPFASILKDLVNKSTVGVGIPCKSVYFLWVARDQRQFEWLLDIIEETEKNDALGILSTHIFITEIPNKFDLRTTMLYVCEQHFKKVSEKSMFTGLNAVTHFGRPNFPDFLKTLSWKHSEVKKIGVFSCGPPSMTESVESACLKANKHKGPMYAHHFENF

>C.intestinalis_5_Duoxa ENSCINT00000001894

FNGWYNNLANPSSGATETALTRRLKSHYEDGVYAPSGSGRPNPRTLSVEIASGPSGLHSYDNKTTLFVYFGQHIIKEIVENSRQGCPPEYFWIPVPEGDEIFDPRGDGNIRLPYHRSPYIASNTGYTPNHPRDQLNEVTSLLDGDSIYGSTKAWSGELRLRKGELRSADDDGKLPMRNTIGLPLQNPVVPVYHEMRNAKRLFATGSRTGNENPFLLTIGVTWFRHHNWLARQLRDNNPDWSDEHVFMESRTRNIATYQKVFFYEWLPILLGSCPSFPIIPFFKWTGYHASVPVGVSNVFQSAAMNYLDTMVPPGGFIPKTFDSSKHGCNSSNSDEMDKWRTCNTYWNSDELFENATPFNIVYGMMGQMAESEDHIISEDLRNSFHGSMEFSRTDKVATIIQRGRDHGLPDYNTAREDLGLKKKETIRDINPDLFKKDEKLLERLHSLHGGSPSNMDIYVGGMLESKDGRPGELFRSIILEQMVRLRDGDRFWFENLNNGLFTVDQVNGIFNVTFLDVLLRTYPELNLAGNTAFNPFIWSEESNKHICRQPYQMTGDNMTSCTTMQRQDDFNHNQISFLAIFCGVVFFAIDFLLLACVIFMIYVGSWKEDKRNKEKTKTTSTYSEQDAFTAKEILGRKRHERKDVQVVLGPERDIQVITQENNTTHITHITESEEVVIMYTDSGKRKKMLLRCAKRTYDLILAFTGSSERDIFLTKLRLFFKQNDILWKERAIHEKHFFEEANTKDERDTELSNFYQAVIAQSMRMEFGIQISSFPENLVDIKLSQEEFASLLQMKSSSLFVQHIFTTADEDQDGFISFHDFRKIIVLFVKGSPNEKLRLLFDMFDLNQNGSLTKQQFKEMCVCTADTYQSTVDQNVLENVLSKVVPLKNDEEIQYLKFDDFKKLMDLPEMDEAMLNLTAPGRVRITGSQMFIFIGKNKKSLAKSSKNERPNRGNFIQTAEGQGIHIHSSRSPNVSTVENVMPDVYFQNSSKFRLAYRAIRRLFECYALHIFWTSLYIWITIGVFLWAFNSVRSNKAAGLGVIAGNALPLARASAAALMFNISTLLLTMCKNILTFLRETQLHLYIPFDAAVAFHKLVAWMALFFTALHIIAHGINFYSIQTQTPSDLLCLFRDLWFPSDYQPTFVFWCLQTLTGNTGVLLTIIFIVMYVFSLDYPRQVMFNWFQWIHFFGYISVYFFTVLHGSGMLIQIPSFYYYFLVPAILYTFDKLYSVYRKKLQLPVIKAEILPSDVVYLEFVRPSDFYYKAGQWVRIACVGLSKWEYHPFTLSSSPDEETLQLHIRAVGPWTRNIRNIYKEGEPYPKLYVDGPFGEGHQDWYKYEVAVLVGGGIGVTPFASILKDLVNKSTVGVGIPCKSVYFLWVARDQRQFEWLLDIIEETEKNDALGILSTHIFITEIPNKFDLRTTMLYVCEQHFKKVSEKSMFTGLNAVTHFGRPNFPDFLKTLSWKHSEVKKIGVFSCGPPSMTESVESACLKANKHKGPMYAHHFENF

>C.intestinalis_1_NOX2 ENSCINT00000008272

MNARLNGFLVNEFPKYIVFLLWLGLNGFLFGYYYNFYNTKKTFFYTRVLLGPALALARPAACLNLNCLLVLLPVCRNLLSLFRKACMCCPRRIRRVLDKNIKFHRMCAYMIVLMTLIHYFAHCFNVDFFTSAYQSKILATDTPAIIQQKKLIAKLIQIGNNGNETYLNPIRKSVFSVGAVFLLTGGWTGVIITLSLFFMVTSSLEFIRRSYFEVFWFTHHLFIVFYGFLVVHGISMQVRGQTPQSLTVHDPIRCSTIDPATWSQNNCPTPVFAGSPPMTWKWVIAPMVLYVIERIIRLVRFNQQVEVLKVIKHPSRVLEIQMRKNGFFAEVGQYVFIMCPQLSQLEWHPFTLTSAPEEDYFSIHVRIVGDWTTGLSKVLGADEAGNEVQPSWKMPRLAIDGPFGTASEDVFNYPVAICVGSGIGVTPFASLLKSVWYKNLNPEHEMVLKKVYFFWICPETHAFEWFGDLLKYLERQLTEIGRQDLIEYHIYLTRGWDHKQAKAIYAHEEDTHDVITGLEQKTNYGRPNWDEIFSKTARDYPNTHIGVFFCGVAALSAKLHKMSNKHSGGGVYFHYNKENF

>D.melanogaster_1_NOX5 FBgn0085428

MNADQESNNHRGSVSSSRSLEIPATPSRSPKKVSFSDELPQSQTPAQQPINQQSAPTVVSHVLQQAAQYLERLHGARDSAEEKTEEVQPERADIRDSSDTSEAAVLDLDALDASADEPAANAGPIVASSPSILIGSVNRQEAIGSANGNGNANPNQRQSNLYMERYNLNLDKNCSSMELEARREKQRWLLISECSALFDEGEGKHTREAFRKLFLDEEFQQKLFQLFDLERNGYLLQDRWIEHLKGRLTDDRQMDFAEQIESVAYVICGENKRVSFKNFRDIWHTRGILDKLYRLIELDGSNLVSTNQVMEFISHLTNSRPRTGFDKSSLARLEQLFRTTVGNEQEIRREEFQKIVTSKNPFFTERVFQIFDKDNSGSISLQEFIDAIHQFSGQSADDKIRFLFKVYDIDGDGLIQHKELHDVIRHCIKENGMEFSEDQIEDLTSAMFEDADPHNSGEITYEALKNQLHKHGGLLENLSITIDRWLVPIAEDRQAGGAAKSGFWNSLPHQFSLAYMKNNQVFVTYLFFYITVNLCLFISRAIQYRASNGFVIIARACGQCLNFNCAWVLVLMLRHSLTYLRGRGLSSYLPLDHHVYLHKLTGITISVLSLIHTIMHLFNFSIIVINDPNINAGHYTIGEWLLTDRPGLFGLIPGCANPTGVALLAILVVMFVCSQPFVRRKGSFEVFYWTHLLYVPFWILCLFHGPNFWKWFLLPGLVYIVERALRFIWMRGEHGKTYISSGLLLPSKVVHLVIKRPHHFNFRPGDYVFVNIPAIANYEWHPFTISSAPEQEDYMWLHIRTVGEWTNRLYRYFEREQQKLQQSGSSQEIPQHMHAIPTPSFMLLNEARNPAIAGERSATPQTDFLAKNLGVQAVPPVRPPRQNRKPAPGAPIDPPATGVNRIRSIKKTLQRTFSRKEAVDPKKGIPNGAFIADGEREDSNLKQRPLEKSISLPDISVKSKKRSRLKALRALGRSESESAFDEKRVRRARNNSVGLAYLSPQNKSLAQSFRYMRTKPTIIAFKTPSMEEREHQVAAGEANGASPASRAEQGQLSSRMDSADKLQLARLSLSAEGASKPLEDQTQTGSPSRKSILRRPTFLRSLSASINNRTGGGGGGSTGSSTTNSGGKVTLDAGVMEIFIDGPYGAPSSHIFGAQHAVLIGTGIGVTPFASILQSIMHRYWKARHSCPRCQFEWASEIPKSVMNLRKVDFFWINRDQRSFEWFVNLLSQLEIEQAELGGAMERFLDMHMYITSALQRTDMKAVGLQLALDLLHEKGKRDLITGLKTRTNAGRPNWDKVFKQLQAQQKGKVTVFYCGPPQLAKTLRYKCDQYGFAFRKECF

>D.melanogaster_2_Duox FBgn0283531

MSVPSAPHQRAESKNRVPRPGQKNRKLPKLRLHWPGATYGGALLLLLISYGLELGSVHCYEKMYSQTEKQRYDGWYNNLAHPDWGSVDSHLVRKAPPSYSDGVYAMAGANRPSTRRLSRLFMRGKDGLGSKFNRTALLAFFGQLVANEIVMASESGCPIEMHRIEIEKCDEMYDRECRGDKYIPFHRAAYDRDTGQSPNAPREQINQMTAWIDGSFIYSTSEAWLNAMRSFHNGTLLTEKDGKLPVRNTMRVPLFNNPVPSVMKMLSPERLFLLGDPRTNQNPAILSFAILFLRWHNTLAQRIKRVHPDWSDEDIYQRARHTVIASLQNVIVYEYLPAFLGTSLPPYEGYKQDIHPGIGHIFQAAAFRFGHTMIPPGIYRRDGQCNFKETPMGYPAVRLCSTWWDSSGFFADTSVEEVLMGLASQISEREDPVLCSDVRDKLFGPMEFTRRDLGALNIMRGRDNGLPDYNTARESYGLKRHKTWTDINPPLFETQPELLDMLKEAYDNKLDDVDVYVGGMLESYGQPGEFFTAVIKEQFQRLRDADRFWFENERNGIFTPEEIAELRKITLWDIIVNSTDVKEEEIQKDVFMWRTGDPCPQPMQLNATELEPCTYLEGYDYFSGSELMFIYVCVFLGFVPILCAGAGYCVVKLQNSKRRRLKIRQEALRAPQHKGSVDKMLAREWLHANHKRLVTVKFGPEAAIYTVDRKGEKLRTFSLKHIDVVSVEESATNHIKKKPYILLRVPSDHDLVLELESYGARRKFVKKLEDFLLLHKKEMTLMEVNRDIMLARAETRERRQKRLEYFFREAYALTFGLRPGERRRRSDASSDGEVMTVMRTSLSKAEFAAALGMKPNDMFVRKMFNIVDKDQDGRISFQEFLETVVLFSRGKTDDKLRIIFDMCDNDRNGVIDKGELSEMMRSLVEIARTTSLGDDQVTELIDGMFQDVGLEHKNHLTYQDFKLMMKEYKGDFVAIGLDCKGAKQNFLDTSTNVARMTSFNIEPMQDKPRHWLLAKWDAYITFLEENRQNIFYLFLFYVVTIVLFVERFIHYSFMAEHTDLRHIMGVGIAITRGSAASLSFCYSLLLLTMSRNLITKLKEFPIQQYIPLDSHIQFHKIAACTALFFSVLHTVGHIVNFYHVSTQSHENLRCLTREVHFASDYKPDITFWLFQTVTGTTGVMLFIIMCIIFVFAHPTIRKKAYNFFWNMHTLYIGLYLLSLIHGLARLTGPPRFWMFFLGPGIVYTLDKIVSLRTKYMALDVIDTDLLPSDVIKIKFYRPPNLKYLSGQWVRLSCTAFRPHEMHSFTLTSAPHENFLSCHIKAQGPWTWKLRNYFDPCNYNPEDQPKIRIEGPFGGGNQDWYKFEVAVMVGGGIGVTPYASILNDLVFGTSTNRYSGVACKKVYFLWICPSHKHFEWFIDVLRDVEKKDVTNVLEIHIFITQFFHKFDLRTTMLYICENHFQRLSKTSIFTGLKAVNHFGRPDMSSFLKFVQKKHSYVSKIGVFSCGPRPLTKSVMSACDEVNKTRKLPYFIHHFENFG

>D.rerio_1_NOX4 ref|XP_005173476.4|

MSVALKSWSANEGLKHFMMMVWVAVNVFLFWRTFMLYYSGPQYYYLHKMLGLGLCISRASASVLNLNCSLVLLPMCRSLLTFLRGSQRVTGRSVRRLLDKSKTFHVACGVTICIFSVVHVSAHLVNLVNFSAQFSEDFPALNVAQYRGQDPRILIVSTVPGVTGVLLVLVLFLMCTASSYCIRLSNYQIFWYTHNLFILFYIILMVHVAGGALKYQSNVETHPPGCIQNNLSSDSWTNHRAAVEERAELSDRSQTVCQEEACFHPHFPQTWLWVSAPLCLYCAERLYRFIRSSVSPVAIVSVISHQCDVIELRMLKSGFKPRPGQYILLNCPTVSSFENHPFTLTTCPTGKNETFGIHLRVLGDWTSRFSQLLLQPSNSSEILPMMHQRRYPTVHVDGPFGSPSEEVFNYEVSLCVAGGIGVTPFACVLQALYDDWCHYKLKRLYFVWICRDIQCFYWFADLLCGLYERLWRDNRPDYLNVQLYLSSSQGLQSIGEERYRFLSSRLRIGRPNWKLLFQEIGRANQLKRVGVFCCGPKGISKALHTLCNSNPHSHTAFEYNKESFS

>D.rerio_2_Duox1 ref|XP_021326396.1|

MGSFELLLRTFLAMFILCQAVGAYSAITWEVQRYDGWYNNLADHDRGAADASLVRLYPAQYMDGVYLARQEPHLPNPRRISTTAMSGQSGLLSHKNRSVLSVAFGYHVWSEISESRRAGCPPEFMHIKVQKDDPVFVSNSSQPVLLQFQRADWDTSTGKSPNNPRTQVNHVTAWIDGSSIYGSSSSWSDALREFSGGRLSSSSSRDMPRRSSNGYLMWSSPDPSSGPDSGSQELYEFGNAWANENIFSVTEGIIWFRYHNYLASKLHKEHPSWSDEELFQHARKRVIATFQNIAFYEWLPAFLGTQVTSYPGYQKYVDPGISVEFEAAAVRFGLTLAPPGVYKRNRTCHYRSVVNDDASKSPGLRLCNTFWNRNNPHLQSSLDVDELIMGMASQIAEREDNIIVEDLRDYMYGPLRFSRSDAVALTIQRGRDFGLPSYNQIREALSMAPVNSFEDINPKLKDTKLLKELADLYENDISRLELFVGGLLETQEGPGPVFSTIILDQFERIRNADRFWFENKQNGLFTEEEIKAIRNTTFHDVLLHVTSAEKGDIQRSVFFWKNGDPCPQPQPIRASDLQPCTKAFSMSYFDDSSKVGFGVTVVVLFLFPVVSYIVASAVAQVRTARFKRFQQKRSGSTKHKEPAHGTTASEWLGHNTAPRQVTLAFSEKKVFQTFDENGSPLSSHSLGNQDHLNVIVSNDHQHRALLLKIPKEYDLVLFFEDGVQRSEFLSLLRSELEGRIQSLTVMEKSEKEMLCDAVTREQRGKIVETFFKHAFSKVLDIDKSDAGDLSSRTREALQCELTRAEFASVLGLKSDSLFVESMFTLADKDGNGYLSFQEFLDVIVIFMTGTSEEKSKLLFSTHDIKGDGFLSKEEFTSLLRSFIDISGALSKSQADDGIAAMLQTAGLYNKDRFSWEDFHFLLRDHSAQLNIKGMEVLGKKKLGRQHKVSFIRKNSSSSSVEELTHTPEEEHGQELRQRQTKKAGQSQAKLYVRPQRERFNRNPVQQCVQQFKRFIENYRRHIICTVVIYAISAGLALERCIYYGLQAHSSGIPETSMVGVLVSRGSAAAISFLFPYMLLTVCRNLITMCRETFLNRYIPFDAAIDLHRQMAATALILSVVHSLGHLVNVYIFCISDLSILACLFPKVFSNNGSELPMKWTFWFFKTVPGITGVILLLIFAFMYVFASHYFRRISFRGFWITHHLYVLIYVLTVVHGSYGLLQQPRFHIYLIPPGLLFLLDKLISLSRKKVEIPVLKAELLPSDVTMLEFKRPQGFVYRSGQWVRIACLTLGTDEYHPFTLTSAPHEETLSLHIRAAGPWTSKLREAYSPEKHQELGGLPKLYLDGPFGEGHQEWTDFEVSVLVGAGIGVTPFASILKDLVFKSSVKFKFHCKKVYFLWVTRTQRQFEWLSDIIREVEDMDMQDLVSVHIYITQLPEKFDLRTTMLYVCERHFQKVWNRSLFTGLRSVTHFGRPPFLAFLSSLQEVHPEVEKVGVFSCGPPGLTKNVEKACQQMNKRDQTHFVHHYENF

>D.rerio_3_Duox1 ref|XP_017209762.1|

MGSFELLLRTFLAMFILCQAVGAYSAITWEVQRYDGWYNNLADHDRGAADASLVRLYPAQYMDGVYLARQEPHLPNPRRISTTAMSGQSGLLSHKNRSVLSVAFGYHVWSEISESRRAGCPPEFMHIKVQKDDPVFVSNSSQPVLLQFQRADWDTSTGKSPNNPRTQVNHVTAWIDGSSIYGSSSSWSDALREFSGGRLSSSSSRDMPRRSSNGYLMWSSPDPSSGPDSGSQELYEFGNAWANENIFSVTEGIIWFRYHNYLASKLHKEHPSWSDEELFQHARKRVIATFQNIAFYEWLPAFLGTHVTSYPGYQKYVDPGISVEFEAAAVRFGLTLAPPGVYKRNRTCHYRSVVNDDASKSPGLRLCNTFWNRNNPHLQSSLDVDELIMGMASQIAEREDNIIVEDLRDYMYGPLRFSRSDAVALTIQRGRDFGLPSYNQIREALSMAPVNSFEDINPKLKDTKLLKELADLYENDISRLELFVGGLLETQEGPGPVFSTIILDQFERIRNADRFWFENKQNGLFTEEEIKAIRNTTFHDVLLHVTSAEKGDIQRSVFFWKNGDPCPQPQPIRASDLQPCTKAFSMSYFDDSSKVGFGVTVVVLFLFPVVSYIVASAVAQVRTARFKRFQQKRSGSTKHKEPAHGTTASEWLGHNTAPRQVTLAFSEKKVFQTFDENGSPLSSHSLGNQDHLNVIVSNDHQHRALLLKIPKEYDLVLFFEDGVQRSEFLSLLRSELEGRIQSLTVMEKSEKEMLCDAVTREQRGKIVETFFKHAFSKVLDIDKSDAGDLSSRTREALQCELTRAEFASVLGLKSDSLFVESMFTLADKDGNGYLSFQEFLDVIVIFMTGTSEEKSKLLFSTHDIKGDGFLSKEEFTSLLRSFIDISGALSKSQADDGIAAMLQTAGLYNKDRFSWEDFHFLLRDHSAQLNIKGMEVLGKKKLGRQHKVSFIRKNSSSSSVEELTHTPEEEHGQELRQRQTKKAGQSQAKLYVRPQRERFNRNPVQQCVQQFKRFIENYRRHIICTVVIYAISAGLALERCIYYGLQAHSSGIPETSMVGVLVSRGSAAAISFLFPYMLLTVCRNLITMCRETFLNRYIPFDAAIDLHRQMAATALILSVVHSLGHLVNVYIFCISDLSILACLFPKVFSNNGSELPMKWTFWFFKTVPGITGVILLLIFAFMYVFASHYFRRISFRGFWITHHLYVLIYVLTVVHGSYGLLQQPRFHIYLIPPGLLFLLDKLISLSRKKVEIPVLKAELLPSDVTMLEFKRPQGFVYRSGQWVRIACLTLGTDEYHPFTLTSAPHEETLSLHIRAAGPWTSKLREAYSPEKHQELGGLPKLYLDGPFGEGHQEWTDFEVSVLVGAGIGVTPFASILKDLVFKSSVKFKFHCKKVYFLWVTRTQRQFEWLSDIIREVEDMDMQDLVSVHIYITQLPEKFDLRTTMLYVCERHFQKVWNRSLFTGLRSVTHFGRPPFLAFLSSLQEVHPEVEKVGVFSCGPPGLTKNVEKACQQMNKRDQTHFVHHYENF

>D.rerio_4_NOX2 ref|NP_956708.1|

MGNFAANEGLSVFVILVWLGINVFLFVYFYLAFLIDKYYYTRVILGHALSWARAPAACLNFNCMLILLPVCRNLLSFLRGSIQCCSRTAARQLDRNITFHKLVAYMIAFHTAVHIIAHLFNFERFMDSQLMINSSHLPYVLSQIGNNDNRSYLNPIRSNDTNPTIVMFTTVAGLTGVVITLALILIITSSMEVIRRSYFEVFWFTHHLFIVFFIGLVLHGIGRIVRGQTDADLQVHDPTICHSKFEKWGQNVTDCPVPIFAGNPPKTWKWVVGPMFLYVCERLVRFYRSQQKVVITKVVTHPSKTLELQMKKKGFKMEVGQYIFMMCPSISQLEWHPFTLTSAPEEDHFSVHIRIVGDWTQALYSACGGDKTAVLDAWTLPKMAVDGPFGTASEDVFRYEAVMLVGAGIGVTPFASVLKSVWYKHVQENQNVFTKKIYFYWLCPETQAFEWFADLLQSLEKQMSDKNMSDFLSYNIYLTRWKDAEAAHLRVQYEAEDDPITGLKQKTRYGKPNWDNEFSLIASQHPGTKVGVFLCGPTALGKALSKQCLSHTEGGTEFIFNKENF

>D.rerio_5_NOX1 ref|NP_001095857.1|

MGNWIINHGLSAFIVVVWMAINIALFVHFYLFYDQGERFEYTRELLGSALAWARAPAAVLNFNCMLILLPVCRNLLSLLRGSFVCCGRTVRKQLDKNLTFHKLVAYMIALMTAVHTIAHLFNAERYSNSLEGEDGDLAFELSLLQDSSELNTTYLNPFPSNSTTPMIFVFTSIAGLTGVVITLALILMITSSMEVIRRSYFEVFWYTHHLFIVFFAGLVFHGAGRVVRGQVTTDPPHNNSFCEDQPDNWGKIPECPIPQFAGGSPQTWMYVIGPMIIYICERLLRFIRYMQPVTYRKIVIRPSKVLELQLVKPGFSMDVGQYVFLNCPAISQLEWHPFTLTSAPEEDFFSVHIRSVGDWTEKLLKMVENLPEGGQGPKYVLLWIFTITSRMGVDGPFGTASEDVFHYEVSMLVGAGIGVTPFASILKSIWYKFKDSDPKLRTKRIYFYWLCRETHAFEWFADLLQVLEREMEERGMRDFLTYKLYLTGWDQSHADHAMVHFDKDTDIITGLKQKTHYGRPNWDKEFEQVRQENPSSVVGTFLCGPQALAKDLEKKCVKYSDVDPRRTKFYFNKENF

>D.rerio_6_Duox1 ref|XP_021326397.1|

MGSFELLLRTFLAMFILCQAVGAYSAITWEVQRYDGWYNNLADHDRGAADASLVRLYPAQYMDGVYLARQEPHLPNPRRISTTAMSGQSGLLSHKNRSVLSVAFGYHVWSEISESRRAGCPPEFMHIKVQKDDPVFVSNSSQPVLLQFQRADWDTSTGKSPNNPRTQVNHVTAWIDGSSIYGSSSSWSDALREFSGGRLSSSSSRDMPRRSSNGYLMWSSPDPSSGPDSGSQELYEFGNAWANENIFSVTEGIIWFRYHNYLASKLHKEHPSWSDEELFQHARKRVIATFQNIAFYEWLPAFLGTHVTSYPGYQKYVDPGISVEFEAAAVRFGLTLAPPGVYKRNRTCHYRSVVNDDASKSPGLRLCNTFWNRNNPHLQSSLDVDELIMGMASQIAEREDNIIVEDLRDYMYGPLRFSRSDAVALTIQRGRDFGLPSYNQIREALSMAPVNSFEDINPKLKDTKLLKELADLYENDISRLELFVGGLLETQEGPGPVFSTIILDQFERIRNADRFWFENKQNGLFTEEEIKAIRNTTFHDVLLHVTSAEKGDIQRSVFFWKNGDPCPQPQPIRASDLQPCTKAFSMSYFDDSSKVGFGVTVVVLFLFPVVSYIVASAVAQVRTARFKRFQQKRSGSTKHKEPAHGTTASEWLGHNTAPRQVTLAFSEKKVFQTFDENGSPLSSHSLGNQDHLNVIVSNDHQHRALLLKIPKEYDLVLFFEDGVQRSEFLSLLRSELEGRIQSLTVMEKSEKEMLCDAVTREQRGKIVETFFKHAFSKVLDIDKSDAGDLSSRTREALQCELTRAEFASVLGLKSDSLFVESMFTLADKDGNGYLSFQEFLDVIVIFMTGTSEEKSKLLFSTHDIKGDGFLSKEEFTSLLRSFIDISGALSKSQADDGIAAMLQTAGLYNKDRFSWEDFHFLLRDHSAQLNIKGMEVLGKKKLGRQHKVSFIRKNSSSSVEELTHTPEEEHGQELRQRQTKKAGQSQAKLYVRPQRERFNRNPVQQCVQQFKRFIENYRRHIICTVVIYAISAGLALERCIYYGLQAHSSGIPETSMVGVLVSRGSAAAISFLFPYMLLTVCRNLITMCRETFLNRYIPFDAAIDLHRQMAATALILSVVHSLGHLVNVYIFCISDLSILACLFPKVFSNNGSELPMKWTFWFFKTVPGITGVILLLIFAFMYVFASHYFRRISFRGFWITHHLYVLIYVLTVVHGSYGLLQQPRFHIYLIPPGLLFLLDKLISLSRKKVEIPVLKAELLPSDVTMLEFKRPQGFVYRSGQWVRIACLTLGTDEYHPFTLTSAPHEETLSLHIRAAGPWTSKLREAYSPEKHQELGGLPKLYLDGPFGEGHQEWTDFEVSVLVGAGIGVTPFASILKDLVFKSSVKFKFHCKKVYFLWVTRTQRQFEWLSDIIREVEDMDMQDLVSVHIYITQLPEKFDLRTTMLYVCERHFQKVWNRSLFTGLRSVTHFGRPPFLAFLSSLQEVHPEVEKVGVFSCGPPGLTKNVEKACQQMNKRDQTHFVHHYENF

>D.rerio_7_NOX5 ref|XP_017209809.1|

MSLDDDTRWLEWVTKQFESIAGEDKEIDLDEFKTALKVKESFFAERFFALFDSDGSSSISLDELLKALDLLIHGTETDKLRFLFQVYDVDGSGSIDPDELRTVLKSCLRESAISLPEEKLDDLTLALFESADKDNSGSITFEELKAELETFPEVMENLTISAANWLKPPDLEQNKRKTPRYLTRAYWHNNSRKLFFLCLYGLLNTFLFIMAMLKHADGGLWIMLARGCGQCLNLNCTFVMVLMLRRCLTWLRATWVVRVLPLDQNILLHQIVGYAILIFSVGHTGAHIMNFARLSQNDGAYQLWEYLFTIRPGIGWVNGTASITGVVIQILIGLMVVCSSTFVRRSGHFEVFYWSHLSYIWVSALLVVHCANFWKWFVVPGVAFLIEKLVGIAVSRMGGLYIVEVNLLPSKVTHLVIKRPPFFQFKPGDYVYINIPTIAKYEWHPFTISSAPEQQETLWLHIRSMGQWTNRLYEYFRQPDTQTNKRLTASLRSRRHQSRAQEEMFRSASCRGSVASNKDDAVELTMYRTNGTRRNSQNPVCDPAAQAELGDASLATRELTAKLSENHRYCNIKCYVDGPFGTPTRQIFASEHAILIGAGIGITPFASILQSIMCRYRMRKQNCPNCSYSWCETIKDNEMKLRKVDFIWINRDQKSFEWFVSLLTKLEMDQADEEPEGRFLEMHMYMTSALSKNDMKAIGLQMALDLLAKKEKRDSITGLRTRTQPGRPDWAKVFQKVSEEKKGKVHVFYCGSPALAKVIKAQCERFGFHFYKENF

>S.purpuratus_9_Duoxb SPU_000513

MTPRDLRGPICVGLLLCMIALCSGLTTTAAPATNATTPATNATTPATNATTPATNATTVNATETTTATQPDNTTTGQTTENATTVMMMTEVTNGTTAAMMTEVTTVPTQTAQGPSTAQTTSSTDPPGTTTAPPVTTPADEDEPIVNDNELLRSIGAFKDDSHSEPEGYDGWYNNLAHPDWGGAELPLTRRLPVSYADGVYAMAGKDRPNPMSISKATMQGETGQRSHLRRTALMTFFGQQVVEEILDAQRGGCPREYENIKIPDGHEYLKEKEGISIMPFVRSRYSFNTGYSPNVPREQLNEITPWFDGGLVYGTTKAWADALRSFKDGRLADNGEIGGEPQFPEQNTLGLPMANPPNPIAEGAARLQRSERFFKLGNPRGNENPFLLTFGVLWFRWHNYWADKFKAETDWEDERIFNEARKWVIATYQSVVFYEWLPGYLNLNENETAEVEYSGYKGYIHPGITHEFQSAAMRFGHTLVPPGVMRRNEQCVFRTSTMKSSGFTEDQDEFSESQGNHGVRTCNSFWNPQHSVREHDIEEFLLGMASQVTEREDNIITEDLQRRVFGPLEFSRRDLMALNIQRGRDHGLPDYNTARVSLGMDRRETFESINNASAHADGVETFIDSEVLDNLARVYENDIDKVDIWAGGLLETTSNGPGELFRFIILDQFVRSRDADRFWFENNVSGQFTNDQIDFIKKVKLWDIIVACTNINGSSLQKDPFHYTPDDPCYDQHPFEGENRTISENDMENCTKLQTFDYFEGSEVSYALSFLALGIWVVGVVAVLLILAHIRQHSIQEARKLQHRKTRSRDPSSLKTYAAAEWCGKKEGSRQVQVKLGPGKKIRITNERGNKILRTVDLTHYTKVGLLVSPDGKRRHLVVKFDREYDIVLKFSDPDSRLEFISDFESFLGSEEVGVGRERQEIGEKELLKVAVTKEHRTKLLEKFFLMAFSQAFKLDFDPENLESLNNKETKDILECELTKTEFADVLTMKPDSLFVEQMFELVDQDNSGSISFREFLDVIVVFAKGQPEDKLKLMFNMYDIDRSGHLSREEFRQMLKSMMEMVSASVEETDLDKLIHDMFQNAGLGDKEALSLDDFIAVMAEHKDELNNAKLDIAGNIPQIAGQKDAGPPGRGAATVIRRGNLNSRARQTIIRAYKDRDQASTKANGAAKGAGGSGGGVRKRAQSKSVRVETVQQEEAKTQSSKAYNTVVRFFENNRLQIFYVVLYLLVLAGVFIERAYYYSVEREFAGLRRIAGFGVSVTRGAASAMMFTYSSLLVTMCRNTITKLRETFLHRYVPFDSALNMHKLIAMLALFFSIMHTIGHSINFYHISTQTADDLTCYFRDFFHRSHELPKFHYWAWGTITGFTGILLVMVCTVIYTFAFQYARRRVFNLFWFTHNMWIIYFILMFLHGSGRLVQPPFTHYFALGPIVLFTLDKLVSISRKKAEIAVTRAELLPSDVTMLEFKRPQGFEYKSGQWVRIACKTLSSSEYHPFTLTSAPHEENLSLHIRAIGPWTMNLRATYDPNVVREHPLPKLFLDGPYGEGHQDWYQYEVAVLVGGGIGVTPFASILKDIVNKSTIGARVTCKKVYFIWVTRTQKHYEWLTDIIRDVEDNDTNDLVSVHIFVTQFFQKFDLRTTMLYICERHFQKISNRSLFTGLKSITHFGRPQFTSFLQSLEDEHPGVGKIGVFSCGPPGMTGGVEQACVDLNKFDGAAFIHHYENF

>S.purpuratus_2_NOX5b SPU_011499

MEYRKLLIVLISLLNTAISQSTRDFYVSHCKDTGQDVDGCSCSINGSHITSLECSASGFPHPPVITWEEGTASYQSSSINLTRFSENMTLVCKARDENLRDVSANRSVCVYYSAKGTQLCVQDRRSRRTSTPHSVLDDLRFDRIQSRMKSEDETASVGSPLEDYNKDIIFEDAPEVSLPLVGQVLLDDLRSLSEDFKKSLKAVIIRLLNKRVKPYKELPMDASANLNASLLHSSMKNSMTRSASTDRDFAFVELVIQKLDQILDETDRDECEVLITKPDFQEVTDESQIPDYKGNRFINRSMLQSTLLQTSMRSSMNSSRITCSVESSVIREELKAKTPSERTAARNAISQIIVDALGKRRRANQHDLNRSVDMLQSRLADPTGEPVNESLQDPVAETEKGFMKEPIDEPPEETTREVFEEHMKEKPQEPMDSMKGQRETCNEGKPIGVRFETSNLVTTRILNRIISANAIWQECRQEKDEKWLVKLEKHFQEVAGDDNLIDLDEFINALNVKKSFFAERFFELIDTDQSGSISLKELIGALRLLVNGTEQEKLHFLFQVYDVDGSGFIDFDELKTVLRSCTAESAMTLCDETLTELTEILFDDADVDGDGEVSFEELSEQLQRYPGITSNLTISYNTLRNHFSVIIFWIVFVMINAGLAAWGAYEGYQSVSDERHPAAISIARSAGRCLSFECCFVLVLMLRKLLTILRNTFLMSVLPLDQHVVIHKIVAVFIIILSVIHTAGHIANIGLIYQVENVNGTTAAWILDVLVRPFPGLGLVEGSCIITGILIIIVLIIMTICSLPFIRRNGYFKVFYWTHQLCIVFWCLIIIHSKYFWIWFIAPGIIYLAERLVRLQFFRRARFGKVYIQKGYVLPANVVQLVIQRPAKFKFHAGEYIHVNIPSIASHEWHPFTISSAPEQQEYLTLHIRCVGHWTKRLYDVVRERELTLLEHENAGFGEIDKDHDEPLEVIVDVSSTKSTTAVEPNGQQSIISDTHSNRNGRRSSQTYSKRRRTASGAINSGFEPELPNGDKDGTKTVTEGKPYNTGQNDNESVTQKVNAAQLSLPRKADHPSASQGDCQPMSYKELGEVSMTLEVNGDSFKMKSLNSRQSIDVQEAPRGSTTKRQSLRVRQSLVGREKRSVCGKPNGDVNRKMSLVTLRRNGARHSLDLSKDLGGRPHTGLEVILDGPYGAPAQHIMEAEHAVLIGAGIGITPFASILQSINERYKAARKHCPNCNHTWVTDSSSILKTKKVDFVWINRDQHSFEWFISLISAIELEQAEIPAADRFLDIHLYMTSALSPSDMKAIGLHVALDLIHKKKKRDTITGLKTRTQAGRPDWDEVFQNLKQQHKGKITVFFCGSPALGKVLSTKCLQYQMEFRKENF

>S.purpuratus_8_NOX2 SPU_018924

MGDKFLNEGLKYFFLLLWLAANVAYWVVTFLVYEQGPQYFYIRRITGVGLSIAKASGATLNLNSMIILLPICRNLISFFRGSCATNTLCRRSVRRQLDKNLTFHKTVAYMIVIWTIVHVVAHAFNFRNLYNHYLCVTTNNDDLCDGISAIGRKFTVKPEDNWLNPIQGAKNLPAGLGLIEQALIPIAGWSGAVLTLVLILMFSSATEFIRRSYFETFWITHHLFIVYFAMLLAHGVGGIIRSQTNLDRHDVVFCSENLDLWGPTSEECEDPVFKDGSAASYKWVSGPLFIYLLERMIRFWRSCQTVTLTKAPEDDHFSVHIRVVGDWTRDLFKAMGADKPEQQSQEELARVAVDGPFGTASIDIFKYEVAICVGAGIGVTPFASILKSIWLKSVNNSASLKLKKVYFFWICPDTNAFEWFSTLLDSIDTHFTEQGKPDFLKYYIYLSRGWNNTQAKNIYLQEEQEIDAITGLRQKTHYGRPKWDSNFKMIAEENPRTSIGVFFCGPKALSSVLHENANKFTSLTPDGAKFFYNKENF

>S.purpuratus_5_NOX1 SPU_021226

MRNFGGRLVNDFVRWAVLAAWAIVNLIIWLVTFFKYMDNANYIYTKYLMKNGLPVARASAACLNFNSMLILFPVCRNMISYLRGSCESTKFSRRNLRRQLDKNITFHKLIAYAIGFFVILHVGAHCFNLQNLYNGRKATSEDDWLANRLSQPSFDLNPFKTIRSSDVSGLGVIGPGLSLLAGWTGAVLALTYILMFTSATEFIRYYFETFWLTHHLFVIYYAMLMTHGMGGVVKYQTNVDEHDPVECMVDEETFDQCVIDNPPLFAGTPGASWKWCVTPLCVYFLERILRMIRTWPDVTIVQVVQHQSKVIELRMKKQGFKMLPGQYIFLKCSPISKVQWHPFTLTSAPEEDYFSLHIRRVGDWTDELAVKMGADQAEPLSITQLPRVQVDGPFGTSCTDIFDYDVVMCVSAGIGVTPYASTLKSICHRLQAGDETLHLKRMYFYWICRDTHAFEWFVELLSSLELILRQIDKEHLLSYSIYLTRGWDYTQAKNIFMQEDREIDAVTGLRQKTHYGRPKWDSNFSYIAEKNQGATIGVFFCGPKSLSTILHQSCNKHTSDESDGTRFVYYKENF

>S.purpuratus_3_NOX5b SPU_026673

MSKSQHSSISTRQTRQSHSAETPQVNVSFKDIIQHEDFLDAVKKAVKEAVGDELKDIKKELEKVSHQLDLYESRIVTLEIEKDLNSSKLVTMEKKLSEQSEEIRNLQNKAQEAEQYSRRNSIRIFGCPEAEGESTDSIILGIAKDKLGVDISSNDLDRKTYWGSVCNEPLGDDTYSESHSISRYAMTGRQGKDGKWLVRLEKHLHEVAGDDNLIDLDEFINALNVKESFFAERFFELNDKDQSGSISLEELIVALRLLVNGTEQQKLHFLFQVYDVDGSGFIDVDELRTVLRSCTDECALTLPEETLTELTEILFADADVDGDGEVSFEELSEQLQRYPGITSNLSFCAAEWLNPKKKKPKSQSRCSYHALRNNFSVIIFWIVFVIINVGLAAWGAYEGYRSVNDERHPAAIIIARSAGRCLSFECCFVLVLMLRKLLTILRNTFLMSVLPLDQHVIIHKIVAIFIIILSVIHTAGHIANIGLIYQVENVNGTTAAWIRNVLVRPFPGLGLVEGSCIITGILIIIVLIIMTICSLPFIRRNGYFKVFYWTHQLCIVFWCLIIIHSKYFWIWFIAPGIIYLAERLLRFGFFRRARFGKVYIQKGYVLPANVVHLVIQRPANFRFHAGEYIHVNIPSISFHEWHPFTISSAPEQQDFLTLHIRCVGHWTKRLYNVVRERQLALLEDEPGFGEIDKEHDSPLEVIVEVSSTKSTSAVESNSQQSTTSDIPIDNNGRRSSKTYNKRRRTARGATNLGFVPELPNGDKDGTKTVTEGKPYNTGQNDDESLAQKVNPTQLSLPRKADHPSASQGDCQPMPHKELGEISMTLAVNGNSFKMKSLNSRQSIDVQEAPRGNTTKRQSLRVRLSLAGRQRRSVCGKPNGDVNRKMSLVTLRRNGARHSLDLSKDLGGTPHTGLEVILDGPYGAPAQHIMEAEHAVLIGAGIGITPFASILQSIHERYKAAKKQCPNCNHTWVTDTSSILKTKKVDFFWINRDQHSFEWFISLISAIELEQAEIPAADRFLDIHLYMTSALLPIDMKAIGLHVALDLIHKKKNRDTITGLKTRTQAGRPDWDEVFQNLKQQHKGKITVFFCGSPALGKILSTKCLQYHMEFRKENF

>S.purpuratus_1_NOX5a SPU_028175

MATARRKSSLAFSSVCGNAQPADEDSQWLSWAEKQFCQIAGEDRQIDEDEFKMALNIKKSFFAERFFHLFDQDGSGYISLDELMEGLYLLTKGDPVDKLRFLFSVYDVDGNGAIDHEELKVVLRACLCESSMTISEATIDALTSALFEAADTDGSGAISFEELKEELEKNPDVMENLTIRLVTHFNSNGAIDHEELKVVLRACLCESSMTISEATIDALTSALFEAADTDGSGAISFEELKEELEKNPDVMENLTISAASWLKPPSLKPSRRVLPRYLTWRYVHNNYRKILFLVVFILINVALFTEAAYRYAKKKSNWCLITARGCGQCLNFNSAFVLVLMLRKTITTLRTTKAAEILPTDQNIVFHKLVGIFIALLSGIHTLGHIGNAWFVEKTTDGNVTMSALLFTNPHLTSLGLAPVSGSAFLTGWVLDIILAIMVICSMPFVRRSGHFQVFYFTHMLYVVFWGLLLIHGPRFWYWFVVPGIIFIVEKLSQTKCVKQARYGKTYVQEVNLLPSGVTHLALTRPNRFHYKAGDYIFINIPQIAQYEWHPFTISSAPEQQGTISMHIRSAGNWTNRLYAFFEDRQKRNRDETELLLGSASDIRVAMETEEVEETNHAGEFIRLREMECDAAEADVVKPTLNHRGANGNLPHGLPRGYSVEREESEDNQHQHRTIACQTTFEMKGAKSWRSSLREEKIQVFIDGPYGTATRGIFQAEHAILVGAGIGVTPFASILQSIMHRYRVGRQTCPICQHTWLGNIPTDMMRLKKVDFIWINRNQNAFEWFVSLLTQLEMEQAQEPFDRFLELHMYMTSAMAKNDMKGIGLQMALDIMHKKGHRDLITGLKTRTQPGRPDWNKIFTQIAREKKGKVQVFFCGSPTLAKIIKKSCEKFNFSFHKENF

>X.tropicalis_1_NOX2 gi|71895755|ref|NP_001025689.1|

MGNWIVNEGLSIAVIIVWLGLNGYLFWNFYLVYDEGEKYFYSRKLYGSALAWARAPAACLNFNCLLILLPVCRNLLSFLRGSSACCGRSLRRQLDRNLTFHKMVAWMIALHTAIHTGAHLFNVERLVDARVEANGTIQAALTDLGDREGESYLNFVRSRVPNPIGGINVAFTFLAGLTGVVITLALILIITSSTKTIRRSYFEVFWYTHHLFVIFFIGLVIHGAGKIVRGQTDKSLEKHNSTECEDKFTEWGNITSCPIPQFAGNEPMTWKWVVAPMVLYVFERLVRFWRSQQKVVITKVVTHPFKTIELQMKMKGFKMEVGQYIFVQCPAVSKLEWHPFTLTSAPEEDFFSIHIRIVGDWTEGLFKACGCDKTEFQDAWKMPKIAVDGPFGTASEDVFSYEVAMLVGAGIGVTPFASVLKSVWYRYVNDASTLRLKKIYFYWLCRDTQAFEWFADLLQSLETQMQERDNANFLVYNIYLTGWDESQATAFSLHHDQEKDVITGLKQKTLYGRPNWENEFKTIANAHTSSRVGVFLCGPESLAETLNKQSIANSTVDPRGVHFIFNKENF

>X.tropicalis_2_Duox2 gi|1062863110|ref|XP_017947900.1|

MCGSEGFPASEWCGYKEPSQDVIIQLHPNQILKVLDSSKKKPRVVNLHKHPNVKVVLSNSDGSRTVLIKIPKEYDLVLQFNNQRDRDVFIEQLKESLAGSTISPTFSHLKETVLLKESFTKKQRQQMLETFIRHSLSHVIDINKEHAGTTQGQNFRDVLQCELSREEFADSLGLNPNAQFVESMFSMADKDHNGYLSFEEFCFILCSLIKGSAEDKLKFIFSMHDVNGNGILPKEEFSRMLRSFRNVSSFLSNEKTENVIESMFNEAGISNKKELAWEDFYGLFKDHKNILNQTNLYFDGVTKDPVKNTVSINRNTPQNNHSNNIFTTDSEGLTMRKRTGRTNTSQIQLPNIYTVARREKYETSTFRQKIQQFKRLVENYRRHIVCLIIFYGISAGLFAERAYYYGFASPSSGIADATFIGLIISRGSAASISFMFSYMLLTMCRNLITLLRETFLNQYIPFDSAVDFHRLIAVTALVLSILHSLGHLVNVYIFTIIPLSVLSCLFPTVFVDDGSDHPNKYYWWFFETVPGMTGVLLLAVMALMYVFSCYHFRRVSFRCFWLTHHLYVVFYILTIIHGSFALIQQPRFHIFFIVPALIYSADKLISLSRKKIQINVLDIQRLPSDVIHLEFQRPNDFDYKSGQWVRIACLDLGTDEYHPFTLTSAPHEDTLSLHIRAVGPWTTKLRELYSSKKVENIPYPKLYLDGPFGEGHQEWNKFEVSVLVGGGIGVTPFASILKDLVFKSSVNSRIHCKKVYFIWVTRTQHQFEWLTDIIREVEKNDKQELLSVHIYITQLAEKFDFRTTMLYICEQHFQKVKNQSLMTGLRSVTHFGRPPFAGFFSSLQDVHPKVKKIGVFSCGPPGMTKNVENACRKLNKRDESYFVHHYENF

>X.tropicalis_3_NOX4 gi|847106037|ref|XP_012813485.1|

MCRTVIGLLRGPKMVLSRKTRRMLDKHKTFHAACGLAICLFSAVHVGAHVLNAVNFSVNYNHEFPSINVARYKNEDPRKIIFTSVPGVTGVLMVLILFLMCTASTSSIRTANYGIFLHTHNLFFIFYLLLLLHACAGVLKYQSNLEEHPPGCLYLNRSAQGEVPGAAADGGEFPGRAARALMGSFFSHEDMSVHNNSEKICTKGPTFRPHFPETWLWISGPLCLYCAERLYRYIRSSKPVTIVAVITHPCDVVEIRMVKEKFSARPGQYITLLCPSVSALETHPFTLTMCPTESKATFAIHIKVVGDWTERFYELLESHLTAGTEILPKCQQRKNPKIYVDGPFGSPSEEVFNYQISLCIAGGIGVTPFASVLNRLLDSWDGYKLQRLYFVWVCRDIHSFLWFADLLCLLHRKLWQENRPDYLNIQLYLSQTNGIQNIIGEKYQALNSRLSIGRPQWKLLFEEVAKSSRGKTVGVFCCGPKGISKELHKLCNSANQYGTTFEYNKESFT

>X.tropicalis_4_NOX5 gi|1062865058|ref|XP_017947981.1|

MAKRTMSTEDDSKWLEWVTKQFENIAGDDKEIDLEEFKTALKVKESFFAERFFALFDSDGSGSISLDELLKALNLLIHGNETDKLRFLFQVYDVDGSGSIDPSELRTVLKSCLRESAISLPEEKLDDLTLVLFESADKDHSGSITFQELKEELERFPEVMENLTISAANWLKPPAVQNKSHTPRYLTRTYWHNNRSKLLFMCCYWCLNVLLFGLAAVNHASLGGWIMVAKGCGQCLNFNCTFIVVMMLRRCLTWLRTTCVVRFLPLDQNVVLHELIGYVIFVLTVIHTAAHVTNFINLTEKTGAYTFWEYLLTIRPGIGWISGTASITGILLQLLICLMLLFSNTFVRKGGYFEVFYWTHLSYIWIWILLFLHTPKFWKWFLVPGLLFLLEKLFGAAVSRTGDVYITEVNLLASKVTHLVIKRPPSFQFKPGDYIYLNIPVIAKYEWHPFTISSAPEQADTIWLHIRSLGQWTNSLYEYFHYPQTVNRHETKRQILTQKNRQHHSQEHLFSSGHICEAIASNDDDQIELTSYRPRRNPLGEEVRVSLDGTGSAQGQEICCMANDNQSLCNIKCYIDGPYGTPTRRIFTSDHAVLIGAGIGITPFASILQSIMYRYRMRKQNCPSCQYSWCETLKENEMDLRKVDFIWINRDQKFFEWFVSLLTKLELDQADEEPDGRFLEMHMYMTSALSKNDMKAIGLQMALDLLAQKEKKDSITGLRTRTQPGRPDWNKVFQKIEQENKGKVQVFFCGSPALAKIIKAHCEKFNFKFFKEN

>X.tropicalis_6 gi|512858697|ref|XP_002939249.2| PREDICTED: NADPH oxidase 1

MGNWIANNWFSVVVLATWLGLNIFIFINFFMIFEKGDSYSYTRELLGSALAWARGSAACLNFNCLLILLPVCRNLLSFLRGTCTCVQRSMRKQLDNNLAFHKLVGYTIALMTAIHTIAHLFNVERYCDAAQKKTDTLPGELSSIGEDGTWLNPVRSPTVTPPYFAFTTIAGLTGVVITLALILMITSSTEFIRRCYFEVFWYTHHLFVIFFIGLVFHGAGRIVRGQTSDSMETNNYEKCHNSFTQWQNSKSSNRDDDDHHNDKCTVPAFQGNEPGTWKWVLAPMILYIFERTLRFYRSRQTVVITKAVSHPSKVLEIQMQKRGFKMEVGQYIFINCPSVSALEWHPFTLTSAPEEDCFSVHIRSAGDWTDNLIKVFQEQAENPPRLEVDGPFGTASEDVFQYEVSMLVGAGIGVTPFASILKSIWYKFQRDDQRLKTKKIYFYWICRETGSFAWFADLLRSLEQEMICSGKDGFLNYRLFLTSWDSKIAGHVVIDFDHATDTVTGLRQKTSYGRPIWENEFSKVAEWHPKSTVGVFLCGPQALGKTLKQCCHQYSSLDPRKVQFYFNKENF

>X.tropicalis_9 gi|847100393|ref|XP_012812554.1| PREDICTED: cytochrome b-245, beta polypeptide isoform X1

MGNWIVNEGLSIAVIIVWLGLNGYLFWNFYLVYDEGEKYFYSRKLYGSALAWARAPAACLNFNCLLILLPVCRNLLSFLRGSSACCGRSLRRQLDRNLTFHKMVAWMIALHTAIHTGAHLFNVERLVDARVEANGTIQAALTDLGDREGESYLNFVRSRVPNPIGGINVAFTFLAGLTGVVITLALILIITSSTKTIRRSYFEVFWYTHHLFVIFFIGLVIHGAGKIVRGQTDKSLEKHNSTECEDKFTEWGNITSCPIPQFAGNEPMTWKWVVAPMVLYVFERLVRFWRSQQKVVITKVVTHPFKTIELQMKMKGFKMEVGQYIFVQCPAVSKLEWHPFTLTSAPEEDFFSIHIRIVGDWTEGLFKACGCDKTEFQDAWKMPKIAVDGPFGTASEDVFSYEVAMLVGAGIGVTPFASVLKSVWYRYVNDASTLRLKKIYFYWLCRDTQAFEWFADLLQSLETQMQERDNANFLVYNIYLTGWDESQATAFSLHHDQEKDVITGLKQKTLYGRPNWENEFKTIANAHTSSRVGVFLCGPESLAETLNKQSIANSTVDPRGVHFIFNKENF

>H.sapiens_NOX4.2

MLSAVTDLGSIESTVSKNLPVVSIHQLGLCLSRASASVLNLNCSLILLPMCRTLLAYLRGSQKVPSRRTRRLLDKSRTFHITCGVTICIFSGVHVAAHLVNALNFSVNYSEDFVELNAARYRDEDPRKLLFTTVPGLTGVCMVVVLFLMITASTYAIRVSNYDIFWYTHNLFFVFYMLLTLHVSGGLLKYQTNLDTHPPGCISLNRTSSQNISLPEYFSEHFHEPFPEGFSKPAEFTQHKFVKICMEEPRFQANFPQTWLWISGPLCLYCAERLYRYIRSNKPVTIISVMSHPSDVMEIRMVKENFKARPGQYITLHCPSVSALENHPFTLTMCPTETKATFGVHLKIVGDWTERFRDLLLPPSSQDSEILPFIQSRNYPKLYIDGPFGSPFEESLNYEVSLCVAGGIGVTPFASILNTLLDDWKPYKLRRLYFIWVCRDIQSFRWFADLLCMLHNKFWQENRPDYVNIQLYLSQTDGIQKIIGEKYHALNSRLFIGRPRWKLLFDEIAKYNRGKTVGVFCCGPNSLSKTLHKLSNQNNSYGTRFEYNKESFS*

>H.sapiens_NOX4.4

MNVLLFWKTFLLYNQGPEYHYLHQMLGLGLCLSRASASVLNLNCSLILLPMCRTLLAYLRGSQKVPSRRTRRLLDKSRTFHITCGVTICIFSGVHVAAHLVNALNFSVNYSEDFVELNAARYRDEDPRKLLFTTVPGLTGVCMVVVLFLMITASTYAIRVSNYDIFWYTHNLFFVFYMLLTLHVSGGLLKYQTNLDTHPPGCISLNRTSSQNISLPEYFSEHFHEPFPEGFSKPAEFTQHKFVKICMEEPRFQANFPQTWLWISGPLCLYCAERLYRYIRSNKPVTIISVMSHPSDVMEIRMVKENFKARPGQYITLHCPSVSALENHPFTLTMCPTETKATFGVHLKIVGDWTERFRDLLLPPSSQDSEILPFIQSRNYPKLYIDGPFGSPFEESLNYEVSLCVAGGIGVTPFASILNTLLDDWKPYKLRRLYFIWVCRDIQSFRWFADLLCMLHNKFWQENRPDYVNIQLYLSQTDGIQKIIGEKYHALNSRLFIGRPRWKLLFDEIAKYNRGKTVGVFCCGPNSLSKTLHKLSNQNNSYGTRFEYNKESFS*

>H.sapiens_NOX4.5

MAVSWRSWLANEGVKHLCLFIWLSMNVLLFWKTFLLYNQGPEYHYLHQMLGLGLCLSRASASVLNLNCSLILLPMCRTLLAYLRGSQKVPSRRTRRLLDKSRTFHITCGVTICIFSGVHVAAHLVNALNFSVNYSEDFVELNAARYRDEDPRKLLFTTVPGLTGVCMVVVLFLMITASTYAIRVSNYDIFWYTHNLFFVFYMLLTLHVSGDDWKPYKLRRLYFIWVCRDIQSFRWFADLLCMLHNKFWQENRPDYVNIQLYLSQTDGIQKIIGEKYHALNSRLFIGRPRWKLLFDEIAKYNRGKTVGVFCCGPNSLSKTLHKLSNQNNSYGTRFEYNKESFS*

>H.sapiens_NOX4.7

MAVSWRSWLANEGVKHLCLFIWLSMNVLLFWKTFLLYNQGPEYHYLHQMLGLGLCLSRASASVLNLNCSLILLPMCRTLLAYLRGSQKVPSRRTRRLLDKSRTFHITCGVTICIFSGVHVAAHLVNALNFSVNYSEDFVELNAARYRDEDPRKLLFTTVPGLTGVCMVVVLFLMITASTYAIRVSNYDIFWYTHNLFFVFYMLLTLHVSGGLLKYQTNLDTHPPGCISLNRTSSQNISLPEYFSEHFHEPFPEGFSKPAEFTQHKFVKICMEEPRFQANFPQTWLWISGPLCLYCAERLYRYIRSNKPVTIISVMSHPSDVMEIRMVKENFKARPGQYITLHCPSVSALENHPFTLTMCPTETKATFGVHLKIVGDWTERFRDLLLPPSSQDSEILPFIQSRNYPKDDWKPYKLRRLYFIWVCRDIQSFRWFADLLCMLHNKFWQENRPDYVNIQLYLSQTDGIQKIIGEKYHALNSRLFIGRPRWKLLFDEIAKYNRGKTVGVFCCGPNSLSKTLHKLSNQNNSYGTRFEYNKESFS*

>H.sapiens_NOX4.8

MAVSWRSWLANEGVKHLCLFIWLSMNVLLFWKTFLLYNQGPEYHYLHQMLGLGLCLSRASASVLNLNCSLILLPMCRTLLAYLRGSQKVPSRRTRRLLDKSRTFHITCGVTICIFSGVHVAAHLVNALNFSVNYSEDFVELNAARYRDEDPRKLLFTTVPGLTGVCMVVVLFLMITASTYAIRVSNYDIFWYTHNLFFVFYMLLTLHVSGVQLKPKQHLGFILK*

>H.sapiens_NOX4.9

MNVLLFWKTFLLYNQGPEYHYLHQMLGLGLCLSRASASVLNLNCSLILLPMCRTLLAYLRGSQKVPSRRTRRLLDKSRTFHITCGVTICIFSGVHVAAHLVNALNFSVNYSEDFVELNAARYRDEDPRKLLFTTVPGLTGVCMVVVLFLMITASTYAIRVSNYDIFWYTHNLFFVFYMLLTLHVSGGLLKYQTNLDTHPPGCISLNRTSSQNISLPEYFSEHFHEPFPEGFSKPAEFTQHKFVKICMEEPRFQANFPQTWLWISGPLCLYCAERLYRYIRSNKPVTIISVMSHPSDVMEIRMVKENFKARPGQYITLHCPSVSALENHPFTLTMCPTETKATFGVHLKIVGDWTERFRDLLLPPSSQDSEILPFIQSRNYPKLYIDGPFGSPFEESLNYEVSLCVAGGIGVTPFASILNTLLDDWKPYKLRRLYFIWVCRDIQSFRWFADLLCMLHNKFWQENRPDYVNIQLYLSQTDGIQKIIGEKYHALNSRLFIGRPRWKLLFDEIAKYNRGKTVGVFCCGPNSLSKTLHKLSNQNNSYGTRFEYNKESFS*

>H.sapiens_NOX4.10

MNVLLFWKTFLLYNQGPEYHYLHQMLGLGLCLSRASASVLNLNCSLILLPMCRTLLAYLRGSQKVPSRRTRRLLDKSRTFHITCGVTICIFSGVHVAAHLVNALNFSVNYSEDFVELNAARYRDEDPRKLLFTTVPGLTGVCMVVVLFLMITASTYAIRVSNYDIFWYTHNLFFVFYMLLTLHVSGGLLKYQTNLDTHPPGCISLNRTSSQNISLPEYFSEHFHEPFPEGFSKPAEFTQHKFVKICMEEPRFQANFPQTWLWISGPLCLYCAERLYRYIRSNKPVTIISVMSHPSDVMEIRMVKENFKARPGQYITLHCPSVSALENHPFTLTMCPTETKATFGVHLKIVGDWTERFRDLLLPPSSQDSEILPFIQSRNYPKLYIDGPFGSPFEESLNYEVSLCVAGGIGVTPFASILNTLLDDWKPYKLRRLYFIWVCRDIQSFRWFADLLCMLHNKFWQENRPDYVNIQLYLSQTDGIQKIIGEKYHALNSRLFIGRPRWKLLFDEIAKYNRGKTVGVFCCGPNSLSKTLHKLSNQNNSYGTRFEYNKESFS*

>H.sapiens_NOX4.11

MNVLLFWKTFLLYNQGPEYHYLHQMLGLGLCLSRASASVLNLNCSLILLPMCRTLLAYLRGSQKVPSRRTRRLLDKSRTFHITCGVTICIFSGVHVAAHLVNALNFSVNYSEDFVELNAARYRDEDPRKLLFTTVPGLTGVCMVVVLFLMITASTYAIRVSNYDIFWYTHNLFFVFYMLLTLHVSGGLLKYQTNLDTHPPGCISLNRTSSQNISLPEYFSEHFHEPFPEGFSKPAEFTQHKFVKICMEEPRFQANFPQTWLWISGPLCLYCAERLYRYIRSNKPVTIISVMSHPSDVMEIRMVKENFKARPGQYITLHCPSVSALENHPFTLTMCPTETKATFGVHLKIVGDWTERFRDLLLPPSSQDSEILPFIQSRNYPKDDWKPYKLRRLYFIWVCRDIQSFRWFADLLCMLHNKFWQENRPDYVNIQLYLSQTDGIQKIIGEKYHALNSRLFIGRPRWKLLFDEIAKYNRGKTVGVFCCGPNSLSKTLHKLSNQNNSYGTRFEYNKESFS*

>H.sapiens_NOX4.12

MAVSWRSWLANEGVKHLCLFIWLSMNVLLFWKTFLLYNQGPEYHYLHQMLGLGLCLSRASASVLNLNCSLILLPMCRTLLAYLRGSQKVPSRRTRRLLDKSRTFHITCGVTICIFSGVHVAAHLVNALNFSVNYSEDFVELNAARYRDEDPRKLLFTTVPGLTGVCMVVVLFLMITASTYAIRVSNYDIFWYTHNLFFVFYMLLTLHVSGGLLKYQTNLDTHPPGCISLNRTSSQNISLPEYFSEHFHEPFPEGFSKPAEFTQHKFVKICMEEPRFQANFPQTWLWISGPLCLYCAERLYRYIRSNKPVTIISVMSHPSDVMEIRMVKENFKARPGQYITLHCPSVSALENHPFTLTMCPTETKATFGVHLKIVGDWTERFRDLLLPPSSQDSEILPFIQSRNYPKLYIDGPFGSPFEESLNYEVSLCVAGGIGVTPFASILNTLLDDWKPYKLRRLYFIWVCRDIQSFRWFADLLCMLHNKFWQENRPDYVNIQLYLSQTDGIQKIIGEKYHALNSRLFIGRPRWKLLFDEIAKYNRGKTVGVFCCGPNSLSKTLHKLSNQNNSYGTRFEYNKESFS*

>H.sapiens_DUOX2

MLRARPEALMLLGALLTGSLGPSGSQDALSLPWEVQRYDGWFNNLRHHERGAVGCRLQRRVPANYADGVYQALEEPQLPNPRRLSNAATRGIAGLPSLHNRTVLGVFFGYHVLSDVVSVETPGCPAEFLNIRIPPGDPVFDPDQRGDVVLPFQRSRWDPETGRSPSNPRDLANQVTGWLDGSAIYGSSHSWSDALRSFSGGQLASGPDPAFPRDSQNPLLMWAAPDPATGQNGPRGLYAFGAERGNREPFLQALGLLWFRYHNLWAQRLARQHPDWEDEELFQHARKRVIATYQNIAVYEWLPSFLQKTLPEYTGYRPFLDPSISPEFVVASEQFFSTMVPPGVYMRNASCHFRKVLNKGFQSSQALRVCNNYWIRENPNLNSTQEVNELLLGMASQISELEDNIVVEDLRDYWPGPGKFSRTDYVASSIQRGRDMGLPSYSQALLAFGLDIPRNWSDLNPNVDPQVLEATAALYNQDLSQLELLLGGLLESHGDPGPLFSAIVLDQFVRLRDGDRYWFENTRNGLFSKKEIEDIRNTTLRDVLVAVINIDPSALQPNVFVWHKGAPCPQPKQLTTDGLPQCAPLTVLDFFEGSSPGFAITIIALCCLPLVSLLLSGVVAYFRGREHKKLQKKLKESVKKEAAKDGVPAMEWPGPKERSSPIIIQLLSDRCLQVLNRHLTVLRVVQLQPLQQVNLILSNNRGCRTLLLKIPKEYDLVLLFSSEEERGAFVQQLWDFCVRWALGLHVAEMSEKELFRKAVTKQQRERILEIFFRHLFAQVLDINQADAGTLPLDSSQKVREALTCELSRAEFAESLGLKPQDMFVESMFSLADKDGNGYLSFREFLDILVVFMKGSPEDKSRLMFTMYDLDENGFLSKDEFFTMMRSFIEISNNCLSKAQLAEVVESMFRESGFQDKEELTWEDFHFMLRDHDSELRFTQLCVKGGGGGGNGIRDIFKQNISCRVSFITRTPGERSHPQGLGPPAPEAPELGGPGLKKRFGKKAAVPTPRLYTEALQEKMQRGFLAQKLQQYKRFVENYRRHIVCVAIFSAICVGVFADRAYYYGFASPPSDIAQTTLVGIILSRGTAASVSFMFSYILLTMCRNLITFLRETFLNRYVPFDAAVDFHRWIAMAAVVLAILHSAGHAVNVYIFSVSPLSLLACIFPNVFVNDGSKLPQKFYWWFFQTVPGMTGVLLLLVLAIMYVFASHHFRRRSFRGFWLTHHLYILLYALLIIHGSYALIQLPTFHIYFLVPAIIYGGDKLVSLSRKKVEISVVKAELLPSGVTYLQFQRPQGFEYKSGQWVRIACLALGTTEYHPFTLTSAPHEDTLSLHIRAVGPWTTRLREIYSSPKGNGCAGYPKLYLDGPFGEGHQEWHKFEVSVLVGGGIGVTPFASILKDLVFKSSLGSQMLCKKIYFIWVTRTQRQFEWLADIIQEVEENDHQDLVSVHIYVTQLAEKFDLRTTMLYICERHFQKVLNRSLFTGLRSITHFGRPPFEPFFNSLQEVHPQVRKIGVFSCGPPGMTKNVEKACQLVNRQDRAHFMHHYENF*

>H.sapiens_DUOX2.2

MLRARPEALMLLGALLTGSLGPSGNQDALSLPWEVQRYDGWFNNLRHHERGAVGCRLQRRVPANYADGVYQALEEPQLPNPRRLSNAATRGIAGLPSLHNRTVLGVFFGYHVLSDVVSVETPGCPAEFLNIRIPPGDPVFDPDQRGDVVLPFQRSRWDPETGRSPSNPRDLANQVTGWLDGSAIYGSSHSWSDALRSFSGGQLASGPDPAFPRDSQNPLLMWAAPDPATGQNGPRGLYAFGAERGNREPFLQALGLLWFRYHNLWAQRLARQHPDWEDEELFQHARKRVIATYQNIAVYEWLPSFLQKTLPEYTGYRPFLDPSISPEFVVASEQFFSTMVPPGVYMRNASCHFRKVLNKGFQSSQALRVCNNYWIRENPNLNSTQEVNELLLGMASQISELEDNIVVEDLRDYWPGPGKFSRTDYVASSIQRGRDMGLPSYSQALLAFGLDIPRNWSDLNPNVDPQVLEATAALYNQDLSQLELLLGGLLESHGDPGPLFSAIVLDQFVRLRDGDRYWFENTRNGLFSKKEIEDIRNTTLRDVLVAVINIDPSALQPNVFVWHKGAPCPQPKQLTTDGLPQCAPLTVLDFFEGSSPGFAITIIALCCLPLVSLLLSGVVAYFRGREHKKLQKKLKESVKKEAAKDGVPAMEWPGPKERSSPIIIQLLSDRCLQVLNRHLTVLRVVQLQPLQQVNLILSNNRGCRTLLLKIPKEYDLVLLFSSEEERGAFVQQLWDFCVRWALGLHVAEMSEKELFRKAVTKQQRERILEIFFRHLFAQVLDINQADAGTLPLDSSQKVREALTCELSRAEFAESLGLKPQDMFVESMFSLADKDGNGYLSFREFLDILVVFMKGSPEDKSRLMFTMYDLDENGFLSKDEFFTMMRSFIEISNNCLSKAQLAEVVESMFRESGFQDKEELTWEDFHFMLRDHDSELRFTQLCVKGGGGGGNGIRDIFKQNISCRVSFITRTPGERSHPQGLGPPAPEAPELGGPGLKKRFGKKAAVPTPRLYTEALQEKMQRGFLAQKLQQYKRFVENYRRHIVCVAIFSAICVGVFADRAYYYGFASPPSDIAQTTLVGIILSRGTAASVSFMFSYILLTMCRNLITFLRETFLNRYVPFDAAVDFHRWIAMAAVVLAILHSAGHAVNVYIFSVSPLSLLACIFPNVFVNDGSKLPQKFYWWFFQTVPGMTGVLLLLVLAIMYVFASHHFRRRSFRGFWLTHHLYILLYALLIIHGSYALIQLPTFHIYFLVPAIIYGGDKLVSLSRKKVEISVVKAELLPSGVTYLQFQRPQGFEYKSGQWVRIACLALGTTEYHPFTLTSAPHEDTLSLHIRAVGPWTTRLREIYSSPKGNGCAGYPKLYLDGPFGEGHQEWHKFEVSVLVGGGIGVTPFASILKDLVFKSSLGSQMLCKKIYFIWVTRTQRQFEWLADIIQEVEENDHQDLVSVHIYVTQLAEKFDLRTTMLYICERHFQKVLNRSLFTGLRSITHFGRPPFEPFFNSLQEVHPQVRKIGVFSCGPPGMTKNVEKACQLVNRQDRAHFMHHYENF*

>H.sapiens_NOX5

MNTSGDPAQTGPEGCRGTMSAEEDARWLRWVTQQFKTIAGEDGEISLQEFKAALHVKESFFAERFFALFDSDRSGTITLQELQEALTLLIHGSPMDKLKFLFQVYDIDGSGSIDPDELRTVLQSCLRESAISLPDEKLDQLTLALFESADADGNGAITFEELRDELQRFPGVMENLTISAAHWLTAPAPRPRPRRPRQLTRAYWHNHRSQLFCLATYAGLHVLLFGLAASAHRDLGASVMVAKGCGQCLNFDCSFIAVLMLRRCLTWLRATWLAQVLPLDQNIQFHQLMGYVVVGLSLVHTVAHTVNFVLQAQAEASPFQFWELLLTTRPGIGWVHGSASPTGVALLLLLLLMFICSSSCIRRSGHFEVFYWTHLSYLLVWLLLIFHGPNFWKWLLVPGILFFLEKAIGLAVSRMAAVCIMEVNLLPSKVTHLLIKRPPFFHYRPGDYLYLNIPTIARYEWHPFTISSAPEQKDTIWLHIRSQGQWTNRLYESFKASDPLGRGSKRLSRSVTMRKSQRSSKGSEILLEKHKFCNIKCYIDGPYGTPTRRIFASEHAVLIGAGIGITPFASILQSIMYRHQKRKHTCPSCQHSWIEGVQDNMKLHKVDFIWINRDQRSFEWFVSLLTKLEMDQAEEAQYGRFLELHMYMTSALGKNDMKAIGLQMALDLLANKEKKDSITGLQTRTQPGRPDWSKVFQKVAAEKKGKVQVFFCGSPALAKVLKGHCEKFGFRFFQENF*

>H.sapiens_NOX5.2

MSAEEDARWLRWVTQQFKTIAGEDGEISLQEFKAALHVKESFFAERFFALFDSDRSGTITLQELQEALTLLIHGSPMDKLKFLFQVYDIDGSGSIDPDELRTVLQSCLRESAISLPDEKLDQLTLALFESADADGNGAITFEELRDELQRFPGVMENLTISAAHWLTAPAPRPRPRRPRQLTRAYWHNHRSQLFCLATYAGLHVLLFGLAASAHRDLGASVMVAKGCGQCLNFDCSFIAVLMLRRCLTWLRATWLAQVLPLDQNIQFHQLMGYVVVGLSLVHTVAHTVNFVLQAQAEASPFQFWELLLTTRPGIGWVHGSASPTGVALLLLLLLMFICSSSCIRRSGHFEVFYWTHLSYLLVWLLLIFHGPNFWKWLLVPGILFFLEKAIGLAVSRMAAVCIMEVNLLPSKVTHLLIKRPPFFHYRPGDYLYLNIPTIARYEWHPFTISSAPEQKDTIWLHIRSQGQWTNRLYESFKASDPLGRGSKRLSRSVTMRKSQRSSKGSEILLEKHKFCNIKCYIDGPYGTPTRRIFASEHAVLIGAGIGITPFASILQSIMYRHQKRKHTCPSCQHSWIEGVQDNMKLHKVDFIWINRDQRSFEWFVSLLTKLEMDQAEEAQYGRFLELHMYMTSALGKNDMKAIGLQMALDLLANKEKKDSITGLQTRTQPGRPDWSKVFQKVAAEKKGKVQVFFCGSPALAKVLKGHCEKFGFRFFQENF*

>H.sapiens_NOX5.3

MENLTISAAHWLTAPAPRPRPRRPRQLTRAYWHNHRSQLFCLATYAGLHVLLFGLAASAHRDLGASVMVAKGCGQCLNFDCSFIAVLMLRRCLTWLRATWLAQVLPLDQNIQFHQLMGYVVVGLSLVHTVAHTVNFVLQAQAEASPFQFWELLLTTRPGIGWVHGSASPTGVALLLLLLLMFICSSSCIRRSGHFEVFYWTHLSYLLVWLLLIFHGPNFWKWLLVPGILFFLEKAIGLAVSRMAAVCIMEVNLLPSKTLSGCTFGPKASGQTGCMSPSRHQTHWAVVLRGCRGV*

>H.sapiens_NOX5.4

MSAEEDARWLRWVTQQFKTIAGEDGEISLQEFKAALHVKESFFAERFFALFDSDRSGTITLQELQEALTLLIHGSPMDKLKFLFQVYDIDVCARQGASAGTEWGAGAGPHWASSPLGTGSGSIDPDELRTVLQSCLRESAISLPDEKLDQLTLALFESADADGNGAITFEELRDELQRFPGVMENLTISAAHWLTAPAPRPRPRRPRQLTRAYWHNHRSQLFCLATYAGLHVLLFGLAASAHRDLGASVMVAKGCGQCLNFDCSFIAVLMLRRCLTWLRATWLAQVLPLDQNIQFHQLMGYVVVGLSLVHTVAHTVNFVLQAQAEASPFQFWELLLTTRPGIGWVHGSASPTGVALLLLLLLMFICSSSCIRRSGHFEVFYWTHLSYLLVWLLLIFHGPNFWKWLLVPGILFFLEKAIGLAVSRMAAVCIMEVNLLPSKVTHLLIKRPPFFHYRPGDYLYLNIPTIARYEWHPFTISSAPEQKDTIWLHIRSQGQWTNRLYESFKASDPLGRGSKRLSRSVTMRKSQRSSKGSEILLEKHKFCNIKCYIDGPYGTPTRRIFASEHAVLIGAGIGITPFASILQSIMYRHQKRKHTCPSCQHSWIEGVQDNMKLHKVDFIWINRDQRSFEWFVSLLTKLEMDQAEEAQYGRFLELHMYMTSALGKNDMKAIGLQMALDLLANKEKKDSITGLQTRTQPGRPDWSKVFQKVAAEKKGKVQVFFCGSPALAKVLKGHCEKFGFRFFQENF*

>H.sapiens_NOX5.5

MAFVCAGLSDTMSAEEDARWLRWVTQQFKTIAGEDGEISLQEFKAALHVKESFFAERFFALFDSDRSGTITLQELQEALTLLIHGSPMDKLKFLFQVYDIDGSGSIDPDELRTVLQSCLRESAISLPDEKLDQLTLALFESADADGNGAITFEELRDELQRFPGVMENLTISAAHWLTAPAPRPRPRRPRQLTRAYWHNHRSQLFCLATYAGLHVLLFGLAASAHRDLGASVMVAKGCGQCLNFDCSFIAVLMLRRCLTWLRATWLAQVLPLDQNIQFHQLMGYVVVGLSLVHTVAHTVNFVLQAQAEASPFQFWELLLTTRPGIGWVHGSASPTGVALLLLLLLMFICSSSCIRRSGHFEVFYWTHLSYLLVWLLLIFHGPNFWKWLLVPGILFFLEKAIGLAVSRMAAVCIMEVNLLPSKVTHLLIKRPPFFHYRPGDYLYLNIPTIARYEWHPFTISSAPEQKDTIWLHIRSQGQWTNRLYESFKASDPLGRGSKRLSRSVTMRKSQRSSKGSEILLEKHKFCNIKCYIDGPYGTPTRRIFASEHAVLIGAGIGITPFASILQSIMYRHQKRKHTCPSCQHSWIEGVQDNMKLHKVDFIWINRDQRSFEWFVSLLTKLEMDQAEEAQYGRFLELHMYMTSALGKNDMKAIGLQMALDLLANKEKKDSITGLQTRTQPGRPDWSKVFQKVAAEKKGKVQVFFCGSPALAKVLKGHCEKFGFRFFQENF*

>H.sapiens_NOX5.6

MNTSGDPAQTGPEGCRGTMSAEEDARWLRWVTQQFKTIAGEDGEISLQEFKAALHVKESFFAERFFALFDSDRSGTITLQELQEALTLLIHGSPMDKLKFLFQVYDIDVCARQGASAGTEWGAGAGPHWASSPLGTGSGSIDPDELRTVLQSCLRESAISLPDEKLDQLTLALFESADADGNGAITFEELRDELQRFPGVMENLTISAAHWLTAPAPRPRPRRPRQLTRAYWHNHRSQLFCLATYAGLHVLLFGLAASAHRDLGASVMVAKGCGQCLNFDCSFIAVLMLRRCLTWLRATWLAQVLPLDQNIQFHQLMGYVVVGLSLVHTVAHTVNFVLQAQAEASPFQFWELLLTTRPGIGWVHGSASPTGVALLLLLLLMFICSSSCIRRSGHFEVFYWTHLSYLLVWLLLIFHGPNFWKWLLVPGILFFLEKAIGLAVSRMAAVCIMEVNLLPSKVTHLLIKRPPFFHYRPGDYLYLNIPTIARYEWHPFTISSAPEQKDTIWLHIRSQGQWTNRLYESFKASDPLGRGSKRLSRSVTMRKSQRSSKGSEILLEKHKFCNIKCYIDGPYGTPTRRIFASEHAVLIGAGIGITPFASILQSIMYRHQKRKHTCPSCQHSWIEGVQDNMKLHKVDFIWINRDQRSFEWFVSLLTKLEMDQAEEAQYGRFLELHMYMTSALGKNDMKAIGLQMALDLLANKEKKDSITGLQTRTQPGRPDWSKVFQKVAAEKKGKVQVFFCGSPALAKVLKGHCEKFGFRFFQENF*

>H.sapiens_DUOX1

MGFCLALAWTLLVGAWTPLGAQNPISWEVQRFDGWYNNLMEHRWGSKGSRLQRLVPASYADGVYQPLGEPHLPNPRDLSNTISRGPAGLASLRNRTVLGVFFGYHVLSDLVSVETPGCPAEFLNIRIPPGDPMFDPDQRGDVVLPFQRSRWDPETGRSPSNPRDPANQVTGWLDGSAIYGSSHSWSDALRSFSRGQLASGPDPAFPRDSQNPLLMWAAPDPATGQNGPRGLYAFGAERGNREPFLQALGLLWFRYHNLWAQRLARQHPDWEDEELFQHARKRVIATYQNIAVYEWLPSFLQKTLPEYTGYRPFLDPSISSEFVAASEQFLSTMVPPGVYMRNASCHFQGVINRNSSVSRALRVCNSYWSREHPSLQSAEDVDALLLGMASQIAEREDHVLVEDVRDFWPGPLKFSRTDHLASCLQRGRDLGLPSYTKARAALGLSPITRWQDINPALSRSNDTVLEATAALYNQDLSWLELLPGGLLESHRDPGPLFSTIVLEQFVRLRDGDRYWFENTRNGLFSKKEIEEIRNTTLQDVLVAVINIDPSALQPNVFVWHKGDPCPQPRQLSTEGLPACAPSVVRDYFEGSGFGFGVTIGTLCCFPLVSLLSAWIVARLRMRNFKRLQGQDRQSIVSEKLVGGMEALEWQGHKEPCRPVLVYLQPGQIRVVDGRLTVLRTIQLQPPQKVNFVLSSNRGRRTLLLKIPKEYDLVLLFNLEEERQALVENLRGALKESGLSIQEWELREQELMRAAVTREQRRHLLETFFRHLFSQVLDINQADAGTLPLDSSQKVREALTCELSRAEFAESLGLKPQDMFVESMFSLADKDGNGYLSFREFLDILVVFMKGSPEEKSRLMFRMYDFDGNGLISKDEFIRMLRSFIEISNNCLSKAQLAEVVESMFRESGFQDKEELTWEDFHFMLRDHNSELRFTQLCVKGVEVPEVIKDLCRRASYISQDMICPSPRVSARCSRSDIETELTPQRLQCPMDTDPPQEIRRRFGKKVTSFQPLLFTEAHREKFQRSCLHQTVQQFKRFIENYRRHIGCVAVFYAIAGGLFLERAYYYAFAAHHTGITDTTRVGIILSRGTAASISFMFSYILLTMCRNLITFLRETFLNRYVPFDAAVDFHRLIASTAIVLTVLHSVGHVVNVYLFSISPLSVLSCLFPGLFHDDGSELPQKYYWWFFQTVPGLTGVVLLLILAIMYVFASHHFRRRSFRGFWLTHHLYILLYVLLIIHGSFALIQLPRFHIFFLVPAIIYGGDKLVSLSRKKVEISVVKAELLPSGVTHLRFQRPQGFEYKSGQWVRIACLALGTTEYHPFTLTSAPHEDTLSLHIRAAGPWTTRLREIYSAPTGDRCARYPKLYLDGPFGEGHQEWHKFEVSVLVGGGIGVTPFASILKDLVFKSSVSCQVFCKKIYFIWVTRTQRQFEWLADIIREVEENDHQDLVSVHIYITQLAEKFDLRTTMLYICERHFQKVLNRSLFTGLRSITHFGRPPFEPFFNSLQEVHPQVRKIGVFSCGPPGMTKNVEKACQLINRQDRTHFSHHYENF*

>DUOX1.2

MGFCLALAWTLLVGAWTPLGAQNPISWEVQRFDGWYNNLMEHRWGSKGSRLQRLVPASYADGVYQPLGEPHLPNPRDLSNTISRGPAGLASLRNRTVLGVFFGYHVLSDLVSVETPGCPAEFLNIRIPPGDPMFDPDQRGDVVLPFQRSRWDPETGRSPSNPRDPANQVTGWLDGSAIYGSSHSWSDALRSFSRGQLASGPDPAFPRDSQNPLLMWAAPDPATGQNGPRGLYAFGAERGNREPFLQALGLLWFRYHNLWAQRLARQHPDWEDEELFQHARKRVIATYQNIAVYEWLPSFLQKTLPEYTGYRPFLDPSISSEFVAASEQFLSTMVPPGVYMRNASCHFQGVINRNSSVSRALRVCNSYWSREHPSLQSAEDVDALLLGMASQIAEREDHVLVEDVRDFWPGPLKFSRTDHLASCLQRGRDLGLPSYTKARAALGLSPITRWQDINPALSRSNDTVLEATAALYNQDLSWLELLPGGLLESHRDPGPLFSTIVLEQFVRLRDGDRYWFENTRNGLFSKKEIEEIRNTTLQDVLVAVINIDPSALQPNVFVWHKGDPCPQPRQLSTEGLPACAPSVVRDYFEGSGFGFGVTIGTLCCFPLVSLLSAWIVARLRMRNFKRLQGQDRQSIVSEKLVGGMEALEWQGHKEPCRPVLVYLQPGQIRVVDGRLTVLRTIQLQPPQKVNFVLSSNRGRRTLLLKIPKEYDLVLLFNLEEERQALVENLRGALKESGLSIQEWELREQELMRAAVTREQRRHLLETFFRHLFSQVLDINQADAGTLPLDSSQKVREALTCELSRAEFAESLGLKPQDMFVESMFSLADKDGNGYLSFREFLDILVVFMKGSPEEKSRLMFRMYDFDGNGLISKDEFIRMLRSFIEISNNCLSKAQLAEVVESMFRESGFQDKEELTWEDFHFMLRDHNSELRFTQLCVKGVEVPEVIKDLCRRASYISQDMICPSPRVSARCSRSDIETELTPQRLQCPMDTDPPQEIRRRFGKKVTSFQPLLFTEAHREKFQRSCLHQTVQQFKRFIENYRRHIGCVAVFYAIAGGLFLERAYYYAFAAHHTGITDTTRVGIILSRGTAASISFMFSYILLTMCRNLITFLRETFLNRYVPFDAAVDFHRLIASTAIVLTVLHSVGHVVNVYLFSISPLSVLSCLFPGLFHDDGSELPQKYYWWFFQTVPGLTGVVLLLILAIMYVFASHHFRRRSFRGFWLTHHLYILLYVLLIIHGSFALIQLPRFHIFFLVPAIIYGGDKLVSLSRKKVEISVVKAELLPSGVTHLRFQRPQGFEYKSGQWVRIACLALGTTEYHPFTLTSAPHEDTLSLHIRAAGPWTTRLREIYSAPTGDRCARYPKLYLDGPFGEGHQEWHKFEVSVLVGGGIGVTPFASILKDLVFKSSVSCQVFCKKIYFIWVTRTQRQFEWLADIIREVEENDHQDLVSVHIYITQLAEKFDLRTTMLYICERHFQKVLNRSLFTGLRSITHFGRPPFEPFFNSLQEVHPQVRKIGVFSCGPPGMTKNVEKACQLINRQDRTHFSHHYENF*

>H.sapiens_DUOX1.3

MWMHCCWAWPPRSQSERTMCWLKMCGVSLRLSLQVVNSWPLGRGSAGLPEPDFWPGPLKFSRTDHLASCLQRGRDLGLPSYTKARAALGLSPITRWQDINPALSRSNDTVLEATAALYNQDLSWLELLPGGLLESHRDPGPLFSTIVLEQFVRLRDGDRYWFENTRNGLFSKKEIEEIRNTTLQDVLVAVINIDPSALQPNVFVWHKGDPCPQPRQLSTEGLPACAPSVVRDYFEGSGFGFGVTIGTLCCFPLVSLLSAWIVARLRMRNFKRLQGQDRQSIVSEKLVGGMEALEWQGHKEPCRPVLVYLQPGQIRVVDGRLTVLRTIQLQPPQKVNFVLSSNRGRRTLLLKIPKEYDLVLLFNLEEERQALVENLRGALKESGLSIQEWELREQELMRAAVTREQRRHLLETFFRHLFSQVLDINQADAGTLPLDSSQKVREALTCELSRAEFAESLGLKPQDMFVESMFSLADKDGNGYLSFREFLDILVVFMKGSPEEKSRLMFRMYDFDGNGLISKDEFIRMLRSFIEISNNCLSKAQLAEVVESMFRESGFQDKEELTWEDFHFMLRDHNSELRFTQLCVKGVEVPEVIKDLCRRASYISQDMICPSPRVSARCSRSDIETELTPQRLQCPMDTDPPQEIRRRFGKKVTSFQPLLFTEAHREKFQRSCLHQTVQQFKRFIENYRRHIGCVAVFYAIAGGLFLERAYYYAFAAHHTGITDTTRVGIILSRGTAASISFMFSYILLTMCRNLITFLRETFLNRYVPFDAAVDFHRLIASTAIVLTVLHSVGHVVNVYLFSISPLSVLSCLFPGLFHDDGSELPQKYYWWFFQTVPGLTGVVLLLILAIMYVFASHHFRRRSFRGFWLTHHLYILLYVLLIIHGSFALIQLPRFHIFFLVPAIIYGGDKLVSLSRKKVEISVVKAELLPSGVTHLRFQRPQGFEYKSGQWVRIACLALGTTEYHPFTLTSAPHEDTLSLHIRAAGPWTTRLREIYSAPTGDRCARYPKLYLDGPFGEGHQEWHKFEVSVLVGGGIGVTPFASILKDLVFKSSVSCQVFCKKIYFIWVTRTQRQFEWLADIIREVEENDHQDLVSVHIYITQLAEKFDLRTTMLYICERHFQKVLNRSLFTGLRSITHFGRPPFEPFFNSLQEVHPQVRKIGVFSCGPPGMTKNVEKACQLINRQDRTHFSHHYENF*

>H.sapiens_NOX3

MMGCWILNEGLSTILVLSWLGINFYLFIDTFYWYEEEESFHYTRVILGSTLAWARASALCLNFNCMLILIPVSRNLISFIRGTSICCRGPWRRQLDKNLRFHKLVAYGIAVNATIHIVAHFFNLERYHWSQSEEAQGLLAALSKLGNTPNESYLNPVRTFPTNTTTELLRTIAGVTGLVISLALVLIMTSSTEFIRQASYELFWYTHHVFIVFFLSLAIHGTGRIVRGQTQDSLSLHNITFCRDRYAEWQTVAQCPVPQFSGKEPSAWKWILGPVVLYACERIIRFWRFQQEVVITKVVSHPSGVLELHMKKRGFKMAPGQYILVQCPAISSLEWHPFTLTSAPQEDFFSVHIRAAGDWTAALLEAFGAEGQALQEPWSLPRLAVDGPFGTALTDVFHYPVCVCVAAGIGVTPFAALLKSIWYKCSEAQTPLKLSKVYFYWICRDARAFEWFADLLLSLETRMSEQGKTHFLSYHIFLTGWDENQALHIALHWDENTDVITGLKQKTFYGRPNWNNEFKQIAYNHPSSSIGVFFCGPKALSRTLQKMCHLYSSADPRGVHFYYNKESF*

>H.sapiens_NOX1

MGNWVVNHWFSVLFLVVWLGLNVFLFVDAFLKYEKADKYYYTRKILGFCSRTLRKQLDHNLTFHKLVAYMICLHTAIHIIAHLFNFDCYSRSRQATDGSLASILSSLSHDEKKGGSWLNPIQSRNTTVEYVTFTSIAGLTGVIMTIALILMVTSATEFIRRSYFEVFWYTHHLFIFYILGLGIHGIGGIVRGQTEESMNESHPRKCAESFEMWDDRDSHCRRPKFEGHPPESWKWILAPVILYICERILRFYRSQQKVVITKVVMHPSKVLELQMNKRGFSMEVGQYIFVNCPSISLLEWHPFTLTSAPEEDFFSIHIRAAGDWTENLIRAFEQQYSPIPRIEVDGPFGTASEDVFQYEVAVLVGAGIGVTPFASILKSIWYKFQCADHNLKTKKIYFYWICRETGAFSWFNNLLTSLEQEMEELGKVGFLNYRLFLTGWDSNIVGHAALNFDKATDIVTGLKQKTSFGRPMWDNEFSTIATSHPKSVVGVFLCGPRTLAKSLRKCCHRYSSLDPRKVQFYFNKENF*

>H.sapiens_NOX1.2

MGNWVVNHWFSVLFLVVWLGLNVFLFVDAFLKYEKADKYYYTRKILGSTLACARASALCLNFNSTLILLPVCRNLLSFLRGTCSFCSRTLRKQLDHNLTFHKLVAYMICLHTAIHIIAHLFNFDCYSRSRQATDGSLASILSSLSHDEKKGGSWLNPIQSRNTTVEYVTFTSIAGLTGVIMTIALILMVTSATEFIRRSYFEVFWYTHHLFIFYILGLGIHGIGGIVRGQTEESMNESHPRKCAESFEMWDDRDSHCRRPKFEGHPPESWKWILAPVILYICERILRFYRSQQKVVITKVVMHPSKVLELQMNKRGFSMEVGQYIFVNCPSISLLEWHPFTLTSAPEEDFFSIHIRAAGDWTENLIRAFEQQYSPIPRIEVDGPFGTASEDVFQYEVAVLVGAGIGVTPFASILKSIWYKFQCADHNLKTKKIYFYWICRETGAFSWFNNLLTSLEQEMEELGKVGFLNYRLFLTGWDSNIVGHAALNFDKATDIVTGLKQKTSFGRPMWDNEFSTIATSHPKSVVGVFLCGPRTLAKSLRKCCHRYSSLDPRKVQFYFNKENF*

>H.sapiens_NOX1.4

MGNWVVNHWFSVLFLVVWLGLNVFLFVDAFLKYEKADKYYYTRKILGSTLACARASALCLNFNSTLILLPVCRNLLSFLRGTCSFCSRTLRKQLDHNLTFHKLVAYMICLHTAIHIIAHLFNFDCYSRSRQATDGSLASILSSLSHDEKKGGSWLNPIQSRNTTVEYVTFTSIAGLTGVIMTIALILMVTSATEFIRRSYFEVFWYTHHLFIFYILGLGIHGIGGIVRGQTEESMNESHPRKCAESFEMWDDRDSHCRRPKFEGHPPESWKWILAPVILYICERILRFYRSQQKVVITKVVMHPSKVLELQMNKRGFSMEVGQYIFVNCPSISLLEWHPFTLTSAPEEDFFSIHIRAAGDWTENLIRAFEQQYSPIPRIEVDGPFGTASEDVFQYEVAVLVGAGIGVTPFASILKSIWYKFQCADHNLKTKKVGHAALNFDKATDIVTGLKQKTSFGRPMWDNEFSTIATSHPKSVVGVFLCGPRTLAKSLRKCCHRYSSLDPRKVQFYFNKENF*

>H.sapiens_CYBB

MGNWAVNEGLSIFVILVWLGLNVFLFVWYYRVYDIPPKFFYTRKLLGSALALARAPAACLNFNCMLILLPVCRNLLSFLRGSSACCSTRVRRQLDRNLTFHKMVAWMIALHSAIHTIAHLFNVEWCVNARVNNSDPYSVALSELGDRQNESYLNFARKRIKNPEGGLYLAVTLLAGITGVVITLCLILIITSSTKTIRRSYFEVFWYTHHLFVIFFIGLAIHGAERIVRGQTAESLAVHNITVCEQKISEWGKIKECPIPQFAGNPPMTWKWIVGPMFLYLCERLVRFWRSQQKVVITKVVTHPFKTIELQMKKKGFKMEVGQYIFVKCPKVSKLEWHPFTLTSAPEEDFFSIHIRIVGDWTEGLFNACGCDKQEFQDAWKLPKIAVDGPFGTASEDVFSYEVVMLVGAGIGVTPFASILKSVWYKYCNNATNLKLKKIYFYWLCRDTHAFEWFADLLQLLESQMQERNNAGFLSYNIYLTGWDESQANHFAVHHDEEKDVITGLKQKTLYGRPNWDNEFKTIASQHPNTRIGVFLCGPEALAETLSKQSISNSESGPRGVHFIFNKENF*

>B.floridae_2

MATTSQSTVGISTAGTSPPPSNTGGTGGVKIDFETDYEVEYAGYDGWYNNRAHPEWGIADSPLTRRLPSHYQDGTYEPSGWDRPNPRTLSELTMKGVTGMGSFRNRSALLTFFGQQVVEEVLDAQRPGCPREYFNIQIPKGDPEYDPDSRGGREIPLLRSRYDMRITGFSPNFPRQQLNEITAFLDGGLMYGVTKAWADALRLLSSDRRGRLASCTDQGVPGCVNDMYPAYNTIGLPMANPPPPREHYLKSSRRFFKLGNPRGNENAFLLTFGVFWFRWHNYLADQIAAKNLDWSDERVFNEARKWVIATHQKIVLYDWLPDFLGENRRPADYKGYSSAVHPGVTHVFQSAAMRWGHTIVPPGVYRRSRVKPDGTCDWRNTTYYTSPHTSGQYFHGVRTCNSYWNSVEPHEEGFKAEDVAPFDGMAEFLLGMSSQVTEREDNIITEDLRGRVFGPLEFSRRDLMAINIQRGRDHGLPDYNTARVEYGLPPRKTFYDVNPWLFNGTYVEHGPELLANITAFATANNWTTDKCDIWVCGLLETTEAGPGELFGAVIKDQFERIRDGDRFWYENDKNGLFTKGEIEQINNITLFDIIKSISNITDADMQSNPFIATDSPCFQPKQLSQFDMEPCTPDTVDKTVDFKRETFDYFTGSEASYILTFMALAVFIIATEYVGKKEGERSVQVKLGTGKEIKVLNDRGKLLRTIDLRNHNQLVLYVEADKHRTLMAIHVPKEYDLILNFDYTEDRDNALMQVEDFLGAVEVGRRRQEMKSSDLMKAIVTKADRQKQLEKFFRIIFAQAGASGFKEQNGDDVHSGYFNKAFKEEKAREDISTYNSKDAKDVLTCELTRAEFAESLSMKPDSLFVEQMFNLVDKDGSGYINFREFLDVIVIFAKGNPDEKAKLMFDMYDVDGDGKLSREEFKTMLKSMMDMVNTEVEQSQLDMLVDSMMEQGGVRDREQMTQQDFINILGEHKQALSNAALNLGANVLTPGEATRHQTVLNRARKTIIRAYQPPEERNGAASNRPSQRVGQGLKVDTLKDKKYTTNPVMKNFNALKRYIQNYRLHIFWLTLYTLVTIGIFAERAYYYSVEREHAGLRRIAGYGVTVTRGAASGMMWAFSIILVTMCRNTITHMRETFLHLYIPFDSAITLHKIIAMTGLFFTVMHCIGHAINFYHISTQTADDLTCLFRDYFHYSNELPKFHYWCFATTTGMTGVLLVLVIAIMYVFATQYARRYVFKAFWLTHNLYPILYILTIVHGSGNLVQEPFFYYFFLGPVILFTLDKLVSISRKKVEIPVVKAEHLPSNVTMLKFKRPTNFDYKSGQWVRIASAALGDNEYHPFTLTSAPHEDTLSLHIRSVGPWTNNLRKVYDPAKIQPDFGYPKVFVDGPYGEGHQDWYKFPVAILIGGGIGVTPFAAILKDIVQKSAQGAKFNCKKVYFLWVTRTQKQFEWLTDIIREVEEKDKNDLVSVHIFITQFYQKFDLRTTMLYICERHFQRISGRSLFTGLNSITHFGRPNFESFFDTLQDEHPEVSTFGVFSCGPPPMTYTADKACGAMNRVDGASFIHHFENF

>B.floridae_4

MGGTAPTVLAVLAIVVSSVSVGLGQSAAKGFQFQFSHYEREVEYEGYDGWYNNRAHPEWGIADSPLTRRLPSHYHDGVYRPSGSDRPNPRSLSELTMKGITGNGSYRNRSALLTFFGQQVVEEILDAQRPGCPIEYFNIPIPRGDPDYDPDSRGGRELPFLRSRYDMGSTGYSPNNPRQQLNEITPYLDGGLMYGVTKAWADALRLFSEDRPGRLISCGDQGHAECGDLYPAYNTLGLPFANPPPPRDHILKPSYRFFKIGNPRGNENPFLLTFGILWFRWHNYLADRIHAQFPDWHDERVFNEARKWVIATHQLLANITAFAAENDWTTDKCDIWVCGLLETTERGPGPLFREIIKDQFERIRDGDRFWFENYRQNGLFTEDEVTQIWNLTLYEVISSITNITDMDMQENVFLATDSPCFQPKQLSQFDMEPCTPDRLGDPADFKRQTFDYFSGSEWSYALSFGAMGLFVVGCVLVLVCLAKRRKLEEAKERIEHRRKETMSATRRENGQSTILASEYMGGKDGEHSVQIMLGPGKEVKVLDQCLNTVRTIDLKNYARLDIYLAVERNGNMLAMKIPKEYDLILHFDTMGEHNFCLRELEKFLGQEGLSCNKHEMKGKELMKKVKTKADRQKQLERFFRINFAQAFNEDNPIDDQMSFREAQEVLSCELTKAEFAQCLSMKSDSIFVEQMFNLVDKDSSGYINFREFLDVIVIFAKGTGDDKAKLMFNMYDVDGDGKLTRDEFKNMLRSMMDMVNTEVEQDQLNELVDAMMKAGGVSDQEILTLQDFLGILGDQKHALNKAGLHMDLPGNNIKIPDKVVGPRPGLRRESTIIRVGQVHQAYNKEAGDSELRRRHTLAAPPSVQRLSFGTKSKVYTANKLTQRLNSFTSKVQNNKLQIFWMTLYLLVTAGIFIERAYFYSVEREHAGLRRITGFGVSVTRGAASGQMWTYSVILLTMCRNTITHMRETFLHRYIPFDSAIAMHKIVAMSALFFTIMHCFGHGINFFHISTQPADDLTCLFRDFYHRSFDLPKFHYWLYETTTGMTGVLLVLLLAVMYVFSTQYARRYVFKAFWFTHNLYPILYILTIVHGSGHLVQEPYFYYFLLGPLVLFTLDKLVSISRKKVEIPVLNAELLPSAVTMLELKRPANFDYKSGQWVRIASAALGNNEYHPFTLTSAPHEDTLSLHIRSVGPWTNNLRKVYDPAKIEGHELPMVFVDGPYGEGHQDWYKFPVAVLIGGGIGVTPFAAILKDIVHKSEMGAKFVCKKIYFLWVTRTQKQFEWLTDIIREVEEKDKNDLVSVHIFITQFYQKFDLRTTMLFYQKFDLRTTMLYICERHFQRISGRSLFTGLRSITHFGRPDFQYFLTSLQEEHPELLKFRAITPVIEMARLVWLAFLSATLTLSHGNDRPNFVFFVADDLGIGDVGCFGNDTIRVEEMDWSVGVVMATLERLGMTDDTFVYFTSDNGGHIESGDKGGSNGIYKGGKGQAASEGGIRMPTIVQWPRQIKPGTIVSEATSQMDIFPTVADIIGAPLPQDRVIDGRNILPLLGGAAVTSPHDFLFHYCGEYVHAVRYRPREGNVTWKAHFVSYNWDPGTTGCYTTYVCHCAGPGVTYNDPPLLYDVTNDPAEDRPINVASDPRYAAVIDVIKKAVEEQQKSLTKVEKQFGLSNVIPRPWLQECCNFPFCDCQENVDLSSLQIGG

>B.floridae_5

MCRTVLAVIRGSTQCVGRKARRVLDGSRKFHMLCGAAICVAAATHCLGHFYNALNFSEHYNVKYPEVNLVCNTKQTMSFLMPTTVPGLTGVAMVLLLMLIVSTSSMAIRTVNYELFWYTHHLFLILYCLMLLHPMRGILKEQLNLDEHPPGCHMLMDTGGSNDTLNVTSEPEPEPEPYHGLMSYPEPEAEPLPEGSTDEFGRIVTCKGPPVFDTHAVEVWVWLVGPFCLYCAERVYRLVRSNSPAAISGIIEHPDEVVEVQLTKEGFVAKPGQYVVINCPDVSTLEYHPFTLTMPSSFRLMSIRLYTRIYVHPFSRLHTHTHTQTNTHTHTHTHTCIRIAFVISDENIKCVAPLLLYEKVLLSNLCVQMVHTHRSLYVDGPFGSPSENVFHYDVSLCIAGGIGITPFASVLNHLLNEGTKSSRLRRLYLVWVCKNITSFLWIAQVLCSLQRQAWNENRPDFLNIHLHLTRSRDVQILEREYPFLNSRVHVGRPQWRHIFDEVARYNPRTSIGVFLCGPKGMSKGVKKICLKKNKFHTKFEFHKENF

>B.floridae_6

ISIDNTAKYQAWLEYRFGLIVGPGTTISMERFKAELKSQQHSFLAEQLFHVLNKDGSSELKLSDLMAGLSCLLTGSEEEKARLLFQVYDVNGNGSIDRGELKLVLSSCMEEGAFSDDQISDLTEALFEDADSDCSGAITYIEFLEEMSRQLELLDCLALRSPVLEVKAKSSPLLGGMLSVQAVRNHLRTVVAVALYVVLNVALLCYAAIQYQPNVAMVVAKGCGQCLNLNSMFALLLMLRKMLSYMRSTPVGRYLPLDQHIAFHKVIGVMILVFSLLHTIAHVYNFVKLTETTPHTLMEYMLGTNLGIGWVSGSASMTGWVLLWLLLVITLSSTSLVRNSGHFQWFYWIHQLTIPWFVLLVIHAESFWLWLLLPGLFYVIERVLRTKMARVTRYGHMSIQQGIVLPSKVRQSSSSHFTSTSDPSGDSEYKNVFTLAPTKPRANKVSRDSGVYLCETTNVNIADGTRKVSDTASVLVAEGMPPMERNDSRMSCSSLGQDFSHMFRPPTVYMDGPYGAPSTRIFEAEHAVLVGAGIGVTPFASILQSIVHRYRRARQTCPRCRHAWTAETHRNFLTLKKVDFFWINRDHKSFEWFISLLSQLEIEQSEMGNFERNFLEMHMYMTSMYGRTDSKAIGLRMAMYLTHKKEKRDLITGLKTRTQAGRPDWDKVFRRLDAEKCGKVTVFFCGSAQLGKVLQKKCMEHGFEFCQENFS

>B.floridae_7

MGDWLVNEGPKWLILVLWFGINIGLFVGYFLFYHQGTQFYYLRLLLGHGLAWARAPAACLNFNCMLILLPVCRNLISYLRGSCGCCRRSIRRQLDKNITFHRAVAYMICLHTAIHFGAHCYNLELLVAAQTDPYNLPFFFYGNDTEGLISKLTSLQDGVNSTYINPIRLSNSDAIIEGLKTVAGVTGVVITLALIVMVSSSTEVIRRSYFEVFWYTHHLFIVFFIGLVLHGIGGIVRGQTNIDVHNPEECFRRYLEWSPDDATCKIPQFQGGGAQTWKWVLFPMILYIAERCIRFYRSQQKVVITKVVKHPSKVLELQMKKRGFQMMAGQYIFIHCPSISRLEWHPFTLTSAPQEDHFSVHIRIVGDWTGALSKACGAEEPEFQDAWKMPQIAVDGPFGTASEDIFRYQVGVLVGAGIGVTPFASVLKYIWYQYCNPDITLKLKKVYFYWICPDTLSFEWFADLLQSLENQMLERGMGDFLNYNIYLTRGWDANQAKNIILHEEENSDVITGLQQKTHYGRPHWDQIFSNIAQSHTGVSVGVFFCGPKGLSSTLHHMCNQHSSSDPRGVHFHYNKENF

>B.floridae_8

MLPKGPWRLRRASAPVVSSGDVVSATRRKTSPKLEPIPSVPCAANTTDSDNVFTSDAVTAPNGKRRSTGRSSSDIKVQLLRETYPASAYQRKMQAFKRWVENKRLQLLYLIFYFAITFALFVERAYHYAVEAEDFGIRQLTQYGIIASRGAAASMSFSFSLLLLTMCRNSLTKLRETFLNRYIPFDYAVDFHKIVAITALVFTIIHTLGHLVNVYHFAVHPLNHLVCLFPSLRTLDNGSDQPPKWYWWAFQTMAGVTGVLLLLVVSIIYVFATGYARRRAFSAFWATHQLYIVMFILTVLHGSGRLIQNPSFHLYLIGPAIIFTLDKLVSISHRRQLVDIVKTELLPSDVTNIQFKRPDDFEYKSGQWVRIACPEQGPNEYHPMTLTSAPHENTLSVHVRAVGPWTAQLRRTYDPEDFSNGTQKLFLDGPFGEGHQDWYKYKIAVLVGGGIGITPFASILKDVAYLSSTGANMNCKKVYFLWVTRTQRHFEWFIDIIREVEEQDRRGLVAVHIFITQFFEKFDVRTTMLYIYERHFQRLSGRSLFTGLRATTHFGRPEFVSLLDSIQHQNPEVKKIGVFSCGPPGMTRNVEDACAQLNKKDLAHYVSYYENF

>D.discoideum_NOXA sp|Q9XYS3|NOXA_DICDI Superoxide-generating NADPH oxidase heavy chain subunit A OS=Dictyostelium discoideum OX=44689 GN=noxA PE=2 SV=1

MRLPTKEEIQRYWVNEGNKLILVILYTLGNIAAFVYTFVHYYNSPAFEVVGYGVCFARGCAQLLKLNCALILVPVLRNLLSFLRGTFLNNYVPFDKNIVFHKLIAWVICFATFGHVMAHFNNFRLYQDITPQEYKRILGIDYPNLTPIKYAFATLAGWTGHVVCIVMVLMYTSAVESIRRPMFEGFWYTHHLFVVFFGLLVVHGLHSILEPTSFWKWVIGPCALYIVERLIRLLRSKKTTMLIQARIHPSRVIEVRMKTERFKYKPGQYLFLNCPTIAQNEWHPFTITSAPEEDFVSCHINVVGNWTGKLSTLLNPDKKMGIVQENVLKSPDGKPILRIDGPFGAASEEVFKYKQVILVGAGIGVTPFASILKHIKYQMARTYNTTPLIDKVHFYWICRDRNSFEWFSGLIGELEMENHNNFLEIHPYLTGALSAQEIRDVMYGDEEKDLITGFTTPTQFGRPKWDEIFADHALRYAEKDVGVFFCGPKLLSKSLYKASTHYTKTTTCRFHYNKENF

>D.discoideum_NOXB sp|Q86GL4|NOXB_DICDI Superoxide-generating NADPH oxidase heavy chain subunit B OS=Dictyostelium discoideum OX=44689 GN=noxB PE=2 SV=1

MNEKKELQQELELQEFQTPKNQQLEKLQEPNGEISSTGNETSESGISSPPISQNDNSNNENESLNITPNKPFVSMQEELQNLDIENIPIPPTIQTPKIYKNTNNLIHSKNNLSLPISLSQENIVKLDKVDIESNDQVNSNTDNNNNTNNNNNTNNNKNEKIGLRSKIFKSKIFIKIRGWWWHRGISTYIMLFYIALNIGVGVHMFYNMYHSDIFKFLGLSFCFSRTAARLINLNSAVILLPVLRNFLSWLRGTIVNNYIPIDKHLNFHKLCAFMLFCCTIIHCVGHYISFKKINDDVLKIDDGKSVAGDYLNININNFPDEKYLFFKSVPGITGHIMLLILILIVSSSMWRIRRPMFEIFWYVHHLFIPFYILLCFHGYSKILKKDPQSWMWIIAPFILYSIERLIRIARSKKRVILEKAIMHPSKVLELRMKRDNDNFNFKPGQYLYLNCPSIAYHEWHPFTITSAPDDPFISVHINIVGNWTRKLFKLLNPDNKLGLIQEDLKSTQNRGKRRILKIDGPFGAPAENFFKYRNLVLIGAGIGVTPFSSILRHLKNQNDKQTNADENHLKINKIYFIWISRQKNSFQWFTDILAELENDERIDSILEIHIFLTGALELDDYAKIKNAQKCHITNLHSKTLFGRPNFRSIFNQLTQLHQREKIGVFYCGNKALGKNIIKNCNKFNGKNNCHLIFHKENF

>D.discoideum_NOXC sp|Q54F44|NOXC_DICDI Superoxide-generating NADPH oxidase heavy chain subunit C OS=Dictyostelium discoideum OX=44689 GN=noxC PE=2 SV=1

MEKQKNISIKKFANPLSFPHEFRTNLHKSTFLDASLLELPNISVDKTKNENNFSITDKILMESLKENEFKIREFIKNQESGNRVLMIDKNSLKELFNINDNQALDLIFNQYVQSTRPNLIRKSSNSSLNIDLKKEIKKKTKKSRKSFSRNSKLNNNDSKIDDKNDNYIDKINDINNFNSDIEEIFKKKKEIENTLKIPIGGNSFPIPVEFNNNNNNNNNNNNNNNNNYNNNIDNNNNNNNNNNNNNINNCNNNNINNDNNNNNNNDNDNNNINTVDNHDDDIINNSNNFNKNEYPSSNISPISPKSSISSFPTNLNNSINNTGSMVSDSLSSCRNSISSSSIDSSVASIPITIQSIDFEDKNIKSDQFKIISKSNIENTIETNPIPPFNQTNNQCEVQLQSHSLPTILKQPHIYKSKSFSSSINSNSKIKKIKKSRSFEIESKINLFDVINHIYLNLSKVGSEEQKITSVFKLYDIYDKGFISRDDLKEVLNYRTKQNGLKFQDFTMESLIDHIFQQFDKNMDGYIDFEEFKSELTINNENKVKEKEENTNYNFKEENIGIYTEKESFHSLKRYLKIEGSKLFFISLFFIINSILVITSFLNVHANNKRAIELFGPGVYITRIAAQLIEFNAAIILMTMCKQLFTMIRNTKFKFLFPVDKYMTFHKLIGYTLIIASFLHTIGWIVGMAVATGKPDNIFYDCLAPHFKFRPTVWEMIFNSLPGVTGFIMISFLIIMAILSLKIIRKSNFELFYYSHHLFIGFYVLLILHGTMGWIRPPTFWKWFIVPGFFYTVDRSFRLFKRTHRVEVLDYCLKNERVINLTFSKPPSFDYKPGQYLLINVPHISKLQWHPFTMTSSPLEDKIYVHIRVTGNWTKKLFRWLSIKKQLQQQQQLYNNIKQQNVLPDGSNFIINNNNNIDQIDLEIGLKPFRINIDGPFGSSSQYALKQKQVILVGAGIGVSPMASLLKDISLKKQRLQLLNQGDQIALEQSKNEITTKFGLGNLEKVHFFWLNRDQHSFQWFEDLLIDISTNGNSNLPKISINTFNTRVFPKNDVRVFMLWNGLDKLFKAQGLDPTTNLPFKTHWGRPNWDTIFQYYSKKYSGESISVFCCGPSQLSKELYEKCRYYTCLKTGGTKFYFHKENF

>A.thaliana_RBOHD sp|Q9FIJ0|RBOHD_ARATH Respiratory burst oxidase homolog protein D OS=Arabidopsis thaliana OX=3702 GN=RBOHD PE=1 SV=1

MKMRRGNSSNDHELGILRGANSDTNSDTESIASDRGAFSGPLGRPKRASKKNARFADDLPKRSNSVAGGRGDDDEYVEITLDIRDDSVAVHSVQQAAGGGGHLEDPELALLTKKTLESSLNNTTSLSFFRSTSSRIKNASRELRRVFSRRPSPAVRRFDRTSSAAIHALKGLKFIATKTAAWPAVDQRFDKLSADSNGLLLSAKFWECLGMNKESKDFADQLFRALARRNNVSGDAITKEQLRIFWEQISDESFDAKLQVFFDMVDKDEDGRVTEEEVAEIISLSASANKLSNIQKQAKEYAALIMEELDPDNAGFIMIENLEMLLLQAPNQSVRMGDSRILSQMLSQKLRPAKESNPLVRWSEKIKYFILDNWQRLWIMMLWLGICGGLFTYKFIQYKNKAAYGVMGYCVCVAKGGAETLKFNMALILLPVCRNTITWLRNKTKLGTVVPFDDSLNFHKVIASGIVVGVLLHAGAHLTCDFPRLIAADEDTYEPMEKYFGDQPTSYWWFVKGVEGWTGIVMVVLMAIAFTLATPWFRRNKLNLPNFLKKLTGFNAFWYTHHLFIIVYALLIVHGIKLYLTKIWYQKTTWMYLAVPILLYASERLLRAFRSSIKPVKMIKVAVYPGNVLSLHMTKPQGFKYKSGQFMLVNCRAVSPFEWHPFSITSAPGDDYLSVHIRTLGDWTRKLRTVFSEVCKPPTAGKSGLLRADGGDGNLPFPKVLIDGPYGAPAQDYKKYDVVLLVGLGIGATPMISILKDIINNMKGPDRDSDIENNNSNNNSKGFKTRKAYFYWVTREQGSFEWFKGIMDEISELDEEGIIELHNYCTSVYEEGDARVALIAMLQSLQHAKNGVDVVSGTRVKSHFAKPNWRQVYKKIAVQHPGKRIGVFYCGMPGMIKELKNLALDFSRKTTTKFDFHKENF

>A.thaliana_RBOHF sp|O48538|RBOHF_ARATH Respiratory burst oxidase homolog protein F OS=Arabidopsis thaliana OX=3702 GN=RBOHF PE=1 SV=1

MKPFSKNDRRRWSFDSVSAGKTAVGSASTSPGTEYSINGDQEFVEVTIDLQDDDTIVLRSVEPATAINVIGDISDDNTGIMTPVSISRSPTMKRTSSNRFRQFSQELKAEAVAKAKQLSQELKRFSWSRSFSGNLTTTSTAANQSGGAGGGLVNSALEARALRKQRAQLDRTRSSAQRALRGLRFISNKQKNVDGWNDVQSNFEKFEKNGYIYRSDFAQCIGMKDSKEFALELFDALSRRRRLKVEKINHDELYEYWSQINDESFDSRLQIFFDIVDKNEDGRITEEEVKEIIMLSASANKLSRLKEQAEEYAALIMEELDPERLGYIELWQLETLLLQKDTYLNYSQALSYTSQALSQNLQGLRGKSRIHRMSSDFVYIMQENWKRIWVLSLWIMIMIGLFLWKFFQYKQKDAFHVMGYCLLTAKGAAETLKFNMALILFPVCRNTITWLRSTRLSYFVPFDDNINFHKTIAGAIVVAVILHIGDHLACDFPRIVRATEYDYNRYLFHYFQTKQPTYFDLVKGPEGITGILMVILMIISFTLATRWFRRNLVKLPKPFDRLTGFNAFWYSHHLFVIVYILLILHGIFLYFAKPWYVRTTWMYLAVPVLLYGGERTLRYFRSGSYSVRLLKVAIYPGNVLTLQMSKPTQFRYKSGQYMFVQCPAVSPFEWHPFSITSAPEDDYISIHIRQLGDWTQELKRVFSEVCEPPVGGKSGLLRADETTKKSLPKLLIDGPYGAPAQDYRKYDVLLLVGLGIGATPFISILKDLLNNIVKMEEHADSISDFSRSSEYSTGSNGDTPRRKRILKTTNAYFYWVTREQGSFDWFKGVMNEVAELDQRGVIEMHNYLTSVYEEGDARSALITMVQALNHAKNGVDIVSGTRVRTHFARPNWKKVLTKLSSKHCNARIGVFYCGVPVLGKELSKLCNTFNQKGSTKFEFHKEHF

>A.thaliana_RBOHC sp|O81210|RBOHC_ARATH Respiratory burst oxidase homolog protein C OS=Arabidopsis thaliana OX=3702 GN=RBOHC PE=2 SV=2

MSRVSFEVSGGYHSDAEAGNSGPMSGGQLPPIYKKPGNSRFTAENSQRTRTAPYVDLTVDVQDDTVSVHSLKMEGGSSVEESPELTLLKRNRLEKKTTVVKRLASVSHELKRLTSVSGGIGGRKPPRPAKLDRTKSAASQALKGLKFISKTDGGAGWSAVEKRFNQITATTGGLLLRTKFGECIGMTSKDFALELFDALARRRNITGEVIDGDQLKEFWEQINDQSFDSRLKTFFDMVDKDADGRLTEDEVREIISLSASANNLSTIQKRADEYAALIMEELDPDNIGYIMLESLETLLLQAATQSVITSTGERKNLSHMMSQRLKPTFNRNPLKRWYRGLRFFLLDNWQRCWVIVLWFIVMAILFTYKYIQYRRSPVYPVMGDCVCMAKGAAETVKLNMALILLPVCRNTITWLRNKTRLGRVVPFDDNLNFHKVIAVGIIVGVTMHAGAHLACDFPRLLHATPEAYRPLRQFFGDEQPKSYWHFVNSVEGITGLVMVLLMAIAFTLATPWFRRGKLNYLPGPLKKLASFNAFWYTHHLFVIVYILLVAHGYYLYLTRDWHNKTTWMYLVVPVVLYACERLIRAFRSSIKAVTIRKVAVYPGNVLAIHLSRPQNFKYKSGQYMFVNCAAVSPFEWHPFSITSAPQDDYLSVHIRVLGDWTRALKGVFSEVCKPPPAGVSGLLRADMLHGANNPDFPKVLIDGPYGAPAQDYKKYEVVLLVGLGIGATPMISIVKDIVNNIKAKEQAQLNRMENGTSEPQRSKKESFRTRRAYFYWVTREQGSFDWFKNIMNEVAERDANRVIEMHNYCTSVYEEGDARSALIHMLQSLNHAKNGVDIVSGTRVMSHFAKPNWRNVYKRIAMDHPNTKVGVFYCGAPALTKELRHLALDFTHKTSTRFSFHKENF

>A.thaliana_RBOHB sp|Q9SBI0|RBOHB_ARATH Respiratory burst oxidase homolog protein B OS=Arabidopsis thaliana OX=3702 GN=RBOHB PE=2 SV=1

MREEEMESSSEGETNKISRCKATGSDNPDEDYVEITLEVRDETINTMKAKATLRSVLSGRLKTMVKSLSFASRRLDRSKSFGAMFALRGLRFIAKNDAVGRGWDEVAMRFDKLAVEGKLPKSKFGHCIGMVESSEFVNELFEALVRRRGTTSSSITKTELFEFWEQITGNSFDDRLQIFFDMVDKNLDGRITGDEVKEIIALSASANKLSKIKENVDEYAALIMEELDRDNLGYIELHNLETLLLQVPSQSNNSPSSANKRALNKMLSQKLIPTKDRNPVKRFAMNISYFFLENWKRIWVLTLWISICITLFTWKFLQYKRKTVFEVMGYCVTVAKGSAETLKFNMALILLPVCRNTITWLRTKSKLIGSVVPFDDNINFHKVVAFGIAVGIGLHAISHLACDFPRLLHAKNVEFEPMKKFFGDERPENYGWFMKGTDGWTGVTMVVLMLVAYVLAQSWFRRNRANLPKSLKRLTGFNAFWYSHHLFVIVYVLLIVHGYFVYLSKEWYHKTTWMYLAVPVLLYAFERLIRAFRPGAKAVKVLKVAVYPGNVLSLYMSKPKGFKYTSGQYIYINCSDVSPLQWHPFSITSASGDDYLSVHIRTLGDWTSQLKSLYSKVCQLPSTSQSGLFIADIGQANNITRFPRLLIDGPYGAPAQDYRNYDVLLLVGLGIGATPLISIIRDVLNNIKNQNSIERGTNQHIKNYVATKRAYFYWVTREQGSLEWFSEVMNEVAEYDSEGMIELHNYCTSVYEEGDARSALITMLQSLHHAKSGIDIVSGTRVRTHFARPNWRSVFKHVAVNHVNQRVGVFYCGNTCIIGELKRLAQDFSRKTTTKFEFHKENF

>A.thaliana_RBOHA sp|O81209|RBOHA_ARATH Respiratory burst oxidase homolog protein A OS=Arabidopsis thaliana OX=3702 GN=RBOHA PE=2 SV=2

MMNRSEMQKLGFEHVRYYTESPYNRGESSANVATTSNYYGEDEPYVEITLDIHDDSVSVYGLKSPNHRGAGSNYEDQSLLRQGRSGRSNSVLKRLASSVSTGITRVASSVSSSSARKPPRPQLAKLRRSKSRAELALKGLKFITKTDGVTGWPEVEKRFYVMTMTTNGLLHRSRFGECIGMKSTEFALALFDALARRENVSGDSININELKEFWKQITDQDFDSRLRTFFAMVDKDSDGRLNEAEVREIITLSASANELDNIRRQADEYAALIMEELDPYHYGYIMIENLEILLLQAPMQDVRDGEGKKLSKMLSQNLMVPQSRNLGARFCRGMKYFLFDNWKRVWVMALWIGAMAGLFTWKFMEYRKRSAYEVMGVCVCIAKGAAETLKLNMAMILLPVCRNTITWLRTKTKLSAIVPFDDSLNFHKVIAIGISVGVGIHATSHLACDFPRLIAADEDQYEPMEKYFGPQTKRYLDFVQSVEGVTGIGMVVLMTIAFTLATTWFRRNKLNLPGPLKKITGFNAFWYSHHLFVIVYSLLVVHGFYVYLIIEPWYKKTTWMYLMVPVVLYLCERLIRAFRSSVEAVSVLKVAVLPGNVLSLHLSRPSNFRYKSGQYMYLNCSAVSTLEWHPFSITSAPGDDYLSVHIRVLGDWTKQLRSLFSEVCKPRPPDEHRLNRADSKHWDYIPDFPRILIDGPYGAPAQDYKKFEVVLLVGLGIGATPMISIVSDIINNLKGVEEGSNRRQSPIHNMVTPPVSPSRKSETFRTKRAYFYWVTREQGSFDWFKNVMDEVTETDRKNVIELHNYCTSVYEEGDARSALITMLQSLNHAKHGVDVVSGTRVMSHFARPNWRSVFKRIAVNHPKTRVGVFYCGAAGLVKELRHLSLDFSHKTSTKFIFHKENF

>A.thaliana_RBOHE sp|O81211|RBOHE_ARATH Respiratory burst oxidase homolog protein E OS=Arabidopsis thaliana OX=3702 GN=RBOHE PE=2 SV=2

MKLSPLSFSTSSSFSHADGIDDGVELISSPFAGGAMLPVFLNDLSRNSGESGSGSSWERELVEVTLELDVGDDSILVCGMSEAASVDSRARSVDLVTARLSRNLSNASTRIRQKLGKLLRSESWKTTTSSTAGERDRDLERQTAVTLGILTARDKRKEDAKLQRSTSSAQRALKGLQFINKTTRGNSCVCDWDCDCDQMWKKVEKRFESLSKNGLLARDDFGECVGMVDSKDFAVSVFDALARRRRQKLEKITKDELHDFWLQISDQSFDARLQIFFDMADSNEDGKITREEIKELLMLSASANKLAKLKEQAEEYASLIMEELDPENFGYIELWQLETLLLQRDAYMNYSRPLSTTSGGVSTPRRNLIRPRHVVQKCRKKLQCLILDNWQRSWVLLVWVMLMAILFVWKFLEYREKAAFKVMGYCLTTAKGAAETLKLNMALVLLPVCRNTLTWLRSTRARACVPFDDNINFHKIIACAIAIGILVHAGTHLACDFPRIINSSPEQFVLIASAFNGTKPTFKDLMTGAEGITGISMVILTTIAFTLASTHFRRNRVRLPAPLDRLTGFNAFWYTHHLLVVVYIMLIVHGTFLFFADKWYQKTTWMYISVPLVLYVAERSLRACRSKHYSVKILKVSMLPGEVLSLIMSKPPGFKYKSGQYIFLQCPTISRFEWHPFSITSAPGDDQLSVHIRTLGDWTEELRRVLTVGKDLSTCVIGRSKFSAYCNIDMINRPKLLVDGPYGAPAQDYRSYDVLLLIGLGIGATPFISILKDLLNNSRDEQTDNEFSRSDFSWNSCTSSYTTATPTSTHGGKKKAVKAHFYWVTREPGSVEWFRGVMEEISDMDCRGQIELHNYLTSVYDEGDARSTLIKMVQALNHAKHGVDILSGTRVRTHFARPNWKEVFSSIARKHPNSTVGVFYCGIQTVAKELKKQAQDMSQKTTTRFEFHKEHF

>P.anserina_NOX3 tr|B2AAS7|B2AAS7_PODAN PaNox3 NADPH oxidase encoded by the PaNox3 protein OS=Podospora anserina (strain S / ATCC MYA-4624 / DSM 980 / FGSC 10383) OX=515849 GN=PODANS_1_5020 PE=4 SV=1

MASNVAPWDDVKYLTDDEINFFLDDLDHNNDGLIDYSEVEQKLDKVHEEIAPKALPHHLHHDGREDLDRHAFLRAIIKSDKNRIPRAEFAETVKSWKIPSMKQDQANDSEQKDYMRKMGIWRRMKSYWAVHGPEIAFIGLVVAMQLAFGVWQFVKYLTGEYYTRGFGWGVVLAKTCAGALYPTFFFLILSMSRYFSTFLRRSYYISRFINWDLSQEFHIRISCVALVLASLHAIGHLGGSFVWGSRKENEDAVAILLGPDAVPRPYINYVKSLPGFTGLTALGLFYLLSLLSTPPVRKWNYEVFQMGHLLMYPIIGLLMAHGTAGLLQWPMFGYWLAFPTLLVLTERIVRLLVGFHKISAALQVLDSETVAIRAKIPSERIWKYNAGQYVFLQVPALSFFQWHPFTVSTCIGNEMQLHIKTDGNWTRRLRDLAGKDGIAQIQIGINGPFGAPAQRFYDFSHTIVVGAGIGVTPFSGILTDLQEKDDKAHNGPGLDAPITAGGRSTSHGSHLIGPGEDGLQDSSVRDREKVSTGAPIEDANHRETLTKGGNETDNHPSDYRRVDFHWSVRDKNNLLWLSDLLNRVSRSQQWHAQHHKEEHGPHLDIRIHTHVTQKRNNIATHVYRWLLEMHRTPEHPASPLTGLLNATHFGRPDFIRILDRHYEEMKGYKAELVRKDKEKWEDEEFKVGVFFCGTPIVGEILADRCRLLSARGRTDGSKIEYHFMMEVFN

>P.anserina_NOX2 tr|B2AL10|B2AL10_PODAN PaNox2 NADPH oxidase encoded by the PaNox2 protein OS=Podospora anserina (strain S / ATCC MYA-4624 / DSM 980 / FGSC 10383) OX=515849 GN=PODANS_5_9580 PE=4 SV=1

MSGYGGYDGGLRSSGSMKQSERSRWTPLTRMLLSGEMTQERQKELTPREKFDKWMVNEGYRRIFVFVFMFLHAILFAFSFVNFAVKENLQIARDTFGPTFMIARSAALVLHVDVALILFPVCRTLISMARQTPLNGIIQFDKNITFHITTAWSIVFWSWVHTIAHWNNFAQVAAKNNLGIYGWLLANFVSGPGWTGYVMLIALMGMVITSVEKTRRANYERFWYTHHMFIVFFFFWSIHGAFCMIQPDFAPFCISIGTQAIGVFWQYWMYGGFAYLAERVAREIRGRHKTYISKVIQHPSNVCEIQIKKEHTKTRAGQYIFFCCPAVSLWQYHPFTLTSAPEEDYISIHMRVVGDFTRAVAETLGCEFDKKKGDASKVVGVDQSNDEVDPALRRVLPRVYIDGPFGSASEDVFKYEISVLCGAGIGVTPFASILKSIWYRMNYPQKRTRLSKVYFFWICRDFGSFEWFRSLLLAIEAQDVDNRIEIHTYLTAKIKVDDATNIMINDANADKDTITGLRSPTNFGRPNWDMIFRGIRKLHTPAEAGVFFCGPKGLGSQLHVFCNKYSEPGFNFVWGKENF

>P.anserina_NOX1 tr|B2AA06|B2AA06_PODAN PaNox1 NADPH oxidase catalytic subunit encoded by the PaNox1 protein OS=Podospora anserina (strain S / ATCC MYA-4624 / DSM 980 / FGSC 10383) OX=515849 GN=PODANS_1_2410 PE=4 SV=1

MGGLVPLLKKQLTGSKILFHILFWTFHWGIFAYGWWKQAADARLAGLNTLQYSVWLSRGAGLVLSVDGMLILLPVCRTIMRFIRPKIKFIPLDENIWMHRQLAYSMLLFTIIHTAAHYVNFYNVEKTQIRPVTAVQIHYVQPGGATGHVMLLCMLLMYTTAHHRIRQQSFETFWYTHHLFIPFFLGLYTHTVGCFVRDTADAISPFAGDEYWEHCIGYLGWRWELWTGGFYLIERLYREIRAIRETKITRVVKHPYDVVEIQFNKPSFKYKAGQWLFLQVPSVSKYQWHPFTITSCPYDPYVSVHIRQVGDFTRELGNAVGAGGIHAKLYEGVDPLGMYDVALANGQKMPALRIDGPYGAPAEDVFENEIAVLIGTGIGVTPWASILKNIWHLRNGPNPPTRLRRVEFIWVCKDTSSFEWFQTLLLSLEEQSAEAARVPGSSGVEFLKIHTYLTQKLDMDTTQNIVLNSVGSSVDPLTELKARTNFGRPNFGRIFQSMSEGIQNRTYLNGLEGNMRTTVGVYFCGPSAAARDIKKAAKAASSSEVRFRFWKEHF

>P.yezoensis_rboh tr|Q2F9N3|Q2F9N3_PYRYE Respiratory burst oxidase-like protein OS=Pyropia yezoensis OX=2788 GN=rboh PE=2 SV=1

MGDDKPPPPKSRVSRVESYLSTNGFVLTFLGLYILANVILFFFAATPERRLWPVGHYRRNLTPVARGAGNLINFNSAVILLVSARKFMSWLRNTPLNMVVPFDKAMPAFHMLVGRVFLAASVVHVGFHLPVYVVSKPWGPGYNGFTQLFITGSMLVALFAILFVTSVRVNRSKRYELFWYSHAICASLGFVLLMIHGLHYGVYWTYRWAAGPMAVYIIDRLMRRVEQKEVRMEVSRDVGAIKGNSMLCLRLPRSFTYEPGQYAEVKVPAISSVQWHPFTIASAPHEPELVFYIKKSGDWTTNLHAMFASTDPTQVEIKVRGPYGSPAQHVGQFENVVLIGGGVGSTPFASVVKSAHNWMAASSTRGPEMSPSSSFNAAAGQVSVPAATARDATTVPASASLSARLTMQHSTSAATVTAQARMPTVDSLADMDEDLSSNGSRIPAARLSDDAALRPPQAPSSFTSDVLERRVAELDRLYSIADKEDNDRVFPPPSSVQSVVEQDMTGHAVDMDGEDGALSDDDDSSRLDEEEEDQLQSQQSIGRVLRHSAFINSTAGQQLIGLALDADADVVKQRAAAKEGANRRTSVLGAFFGGLKGGDDRRGTLPSEVVRARTKRVAVLQILHSVSVSLALLWAMVARFAIVALASIMRGFSPSTAGLAMFNTRGLVVADLVLASACAVPLAVSLGCEASILGVSVYFRQRGSLVDTLFLLPLLLAGVITDALALAGHGRSAAWFASVNLLVLWPLLLFALLFRLTRVVGSRLVLAQNLQSSHSLRSLDFVWTSPSPEHDAWLVEELLPISRSGTVRLHRHITRSAAEVEPWMLDYDEVPLKTTYKRPDWAAIFAGITERSRSGSVVGVFFCGPHPMSKSIQDGIARATALSLARGYRRGAIGLDGSREMRT

>C.crispus_rboh tr|Q2FA46|Q2FA46_CHOCR Respiratory burst oxidase-like protein OS=Chondrus crispus OX=2769 GN=rboh PE=2 SV=1

MIPRSKPDVARPSARIEAYLSTHAFKVLFFAFYGAAVTLMFAWGFKAEFTFEDNFDMPHFNTVRWFIGIARGMGYTLNLNTAFVILLASRLLFTKLRDSPLQLVLPFDAAFPALHIVVGYTIFFAVLVHGSFHFVWLITWDAWTWGLWSFNMSVITGFLLAIVFGTMLVLARPSVRKNNFRLFYAVHIIGATLFFGLLIIHGMFRQVPYTYKWVIPPLILYAIDRFLRRRKVSAVELFLSAENAVLKDGDILELRVPKAFSYQAGQYAEVQVPFINREWHPFTIASAPQDKTMCFYIKALGDWTKELRGAFQARVDGAVTDSLQVNIRGPYGAPAQHVGLYERVVLISGGIGSTPFTSICKDLHHRKVKENATSATGFEPSTSTLLKRIESRVSTAISTLYGVDISNAKDINQEEEEKRVYLANMLNLTAPGSGSSSGETTELEVEMVDASKQADESSSDSDRSTSMESYNVKNMLRKEQDEEYILDDIKNSANARRGNRERLSHLYEGRSKVLEFLHTSRVNLLLLFVLIARIFFICISSIIKADYIMINAEPHAIESGLWIVIVDTVLSIIFAIVLPLTIFLELSYMGSRFFRTVGRTLDFFVFLPLTITSASLGIKALVTERTDEQIVLFLHYIVFLPTLFVLLAVRMYRALGKRTLLTDAPCHCSHRDIVPNVDFVWTVPHENDDEWLRSELEPLADGTELKLHRYVTRAKEVDMEAGSEFITSSNTGRPEWDEIFGKIAAEAPSNSVVGVFFCGPHKMGDSVQSAMRRAEINSNLRGAYLRSTKEKTLMKDLGLPQRGLIKMLMGTGCSVRFVFREENFG

>M.brevicollis_1 XP_001746171.1

MLGINMLVFLAPLGVIALGSLIIEVSQKANRWQRTSIAAIMALLSVCCAIITILSWIYLLKPATVYNYDDDQQNVLENDALLVLIGGFIAMAFTPLTLGEPASRLVLLATRPDCPTHDVTFFSFLSPQIRARQPVTAYLATYHLTYRPATFRPKTSRQPLTRSSFKENWKPIAMLFLLFLVWGFSDGTFSAVWNYSAHSYFMATSSFAVIETEVDNQFYFKVYEDTLVYFAFLTFVVVLGVITHFSPRIQRTMHTRYKVYAPLSSYLPYGVTVGETLLLLAVAGLAGYWLYFWRWGYDRITVRAIKYHRALGVIVWTLVTSHMILWMIKWLRDSTLLHNIFTIDDLKITHENIHYDNFTIVMVETAWLLMTIMVGLALWTRRSSYELFYFTHYFGIFFYIAALVHAWSFWYYTIGGLVLWLYDRCMRIVSASGPVDLKNAYYDIDAGVVRVSMPASDQIQTRDGQLLTHPLDTGVLGSYLSQTVLHWNKPGFQHRAGQYAWICVPYVSHLEWHPFTISSHPSASEHTFHIKAMGKNTWTERLAHAVERDPHMITLRIDGPYGRPEYFDQCSNLLLVAGGIGITPIYAILSELSELYNSGEGLGKIRKVTLVWCMRQPALRQTFAQMIMMAARQSEVFDIKLYFTRGDVGSFGRATDEVEGSEKKQFLDSCVRKGRPDLDSIASKLPSGRDTMLMVCGPQPMIKECSDIAFAYDFSMHHEVFTF

>M.brevicollis_2 XP_001748123.1

MQFWWQQGLTFRQWFVNDGPMYTFVLLWMCANVGLFFYTFSFYTQDKYNYLRRIVHFGLPVARGAASVLNLNCALVLLPVCRNLVNFCRGIFESKRSIRRLFDKNILFHKWCAYVICVFASIHICAHFFNVNNLVEDGTYGRILRDGTPMSQEEVLFTTVAGGTGVGITVPLILMVTTASQQIRRSYFELFWYTHHLFVVFYVCLCLHGYSGFVERQDNPDTYPISLDRGVCTSRDDVLVSNGCASWNAVVSALANQNSSLTEPNLCIPGGVDVGDEEDAFCCPCRLVTQAVLQRGQAATWVWVIGPLILYILERLYRWYKSQTRRLRILKVVKHKDSVPVMEIQFQKVPTKAGQYVFINCPKINSLEWHPITLTSCPELDYVSVHIRLVGDWTTKLADACGFYEDNPKVGSELPYICIDGPFGTASEDMYHYPVAMLIGAGIGVTPFASLLKELYFRKSNPSAYPSFKTQKVYFYWMCPGFDAWGWFASLLIDLEDKLEQLGVPDFLEIRVFTTRGWSQDDAAKIMLQEDESGDSIVRDAETGRALRHKMNFGRPNWDSEFTSVANTHAGNNIGLFFCGPKVLSSQLHVTCNKFTSERAAEGTKFYYNKENF

>T.adhaerens_1 XP_002115436.1

SLDRDAKWLSWFEQQFAAVAGEDRQINIVEFKNALNVKESFFAERFFQLFDEDGSGTISLNELMGGLNKLTSGDNSEKLRFLFKVYDVDGNGFIDPFELKTVLQSCISESSLEFSESNLNALTQALFESADADGSGTITYEELEGELQKHPGVIENLTISAANWLKPPPKKKKNSLKQIYPYYLTLRYIRNNLSITLFMIVYWLTNFGLFGFAVYQQREKNIWIKIARGCGLCLNFNCTFIMVLMLRKTLTIIRSTTLGSYLPIDQHIDFHKMTGIVIGFFALVHTVAHVFNVVLPDPPNNRSAVDVLFTTKYKIGWVGGTAYITGYPLVIILLVMIICSMPFVRRKGYFQVFYWTHLLFVPWFALLIIHCPNFWHWFIVPGSIYVLERIYRSKFVKLARYGRTYIIAANMLPSKVTHLVINRPSNFHFQPGDYAFLQIPAIAKYAEWHPFTISSAPEQKHTLWFHVRSVGTWTTRLYEYFERKHVEAGSDALETTPLSPTHRQDRTRSSSVDISDQKANKKHLKVFIDGPYGTPSTHIFQADHAVLIGAGIGVTPFASILQSIIFRYRSAARQVCPQCNYTWTDKLPSSVMNLKKVDFFWINRDQKAFEWFISLLSQLEIEQTEDGNLNHLLDMHMYMTSALRKTDMKAIGLQMALDLIHKKSEKDLITGLRTKTEAGRPDWNQVFDSLSQSNHGKVSVFFCGSHQLGSLLKSYCQKYGFEFRKENF

>T.adhaerens_2 XP_002112911.1

LLWMIINVILFATTYVNYNTDKKYFYLRKYLKTAVAWARGSAAVLNLNCALILLPVCRNLLSYARGSCEYAICSRRVRRLMDKNITFHRYIGYMICLATAIHVYAHCFNFLYFLVSYKAGDPLLNTLNQLNTSLNPIRFSNVTPGITGIIITLALILMYASSTEVIRRSYFEIFWYAHHLFVIFFIGLCIHAVIGVIKVQVNLDTHDPDYCYNKRPWGPPNNLNCSIPIFKRTSTPQVWKWVIGPIVLYIFERIVRFVRSKQKVVISKVINHPSKVLEIQMQKRGFQSVAGQYVFINCPSIARFEWHPFTLTSAPGDDYFSVHVRIVGDWTGALFEACKANENKVQEAWKMPDVYIDGPFGTCSEDVFLYEVAICIGAGIGVTPFASILKSLWYKQRQSDAGPSKTKKVYFFWICPDSNAFEWFAELLKSLEREMLSHDLGDFLEYNIYLTRGLSDKQTKHIMLRETDSEAVDAITGLQQKTHYGRPRWDQIFSDLASTHSGSDLGVFFCGPKALSSVLHKECNKNTSANTRFFYNKENF

**Alignment used to construct the phylogenetic tree of CuZnSOD and MnSOD sequences**

>Bombyx.mori_SOD6

VI-SFLACAKLRLIPPKSSKVLINDG---IRGTVDFTQRSPFDP-TWVNFQLSNLRFVGSMLQYNIRELPPKLDA-----------------------------------NQVYMCNT--TGDIYN---------PMELD-LKSLPPPGLSTQDHYPVGDLLGKYKDLPGLAELSGAYWDVYLPLQGIY--------------------------------SVVHRSLVLERRP-----------------------GKRNVCGTILLYEQIQMSGAQVRRLFTDTVISLTGIMRKSLLIYD

>Drosophila.melanogaster_105_CuZnSOD

VL-SYLACTKIRHVQPLTHKTFINGG---VKGEVTFMQRSKFDP-TFLNFTLRKFA--EDVAAFRIHSLPPVPSM-----------------------------------GHEDYCLT--TGDMHN---------PREIN--ENIPPPGYGTQEQYPVGDLSGKLQGLPGTSELNGLYWDVFLPLQGRY--------------------------------SIAQRSLVIYTFN-----------------------SNIWGCSSLSQYQQQNMVTAQVRKMFVDGNLPLSGILGKSLVIYE

>Acanthaster.planci_61_CuZnSOD

FA-TFIACSPIQVLPSKTVSAEISSG---VNGSLEFRQRSPYDA-THISVNLANLQ--GLAGGYHVHEQPVKGRN-----------------------------------KDDLPASNDNVGGHYN---------PFGVDAGGS-PSPGTGTHDQYEVGDISGKFGRLTSMDAFSDQFVDWNMQLFGKY--------------------------------SVIGRSIVIHKEE-----------------------ANRWVYGNIGYPTEVT--TVKGKYFFTDPLLPLTGISGKSVVIHT

>Acanthaster.planci_62_CuZnSOD

FS-DPVACSPILEMKPKSSEAMISNN---VSGMVRFRQSSPYDV-TEIKVHLENLR--GLAGGFHVHVLPPVPRY-----------------------------------ASDQPAGPQNVAGHFN---------PYGIGAA---PPPGTGTNDQYEVGDLSGKFGLLTGYDNFNATFLDWNLPLFGRH--------------------------------SIIGRSLVIHKKE-----------------------GDRWVYAAIPYPTKKK--TVKAKFFFTETSLPLSGVSGRSIVIHA

>Strongylocentrotus.purpuratus_66_CuZnSOD

FSLTRQACSPIRLVKSKTVRAEFSNN---VTGIVTFTQASLWES-TNITVDLENLR--QMAGGYHVHKFPVPMRL-----------------------------------TSDFPGSADNVAGHFN---------PYNIDTTAD-PDYPNGTNDQYELGDISGKFGSLAGQDDFSGTFEDWNMPLFGAN--------------------------------SIVGRSVIIHLAS-----------------------SERWAYGLIEYPAPVR--TVTSKYFFTDVQLPLSGVADRAVVVHV

>Branchiostoma.floridae_63_CuZnSOD

-------CAEIYHLEAVSVSARFDSG---VKGTMVFSQNSPYEY-TTVTININNIG--ANAINYHVHEYPIPAKA-----------------------------------ADESMCSGTYTAGHLN---------PFGKVSSDSYPDPAAGTGDQYELGDLSGKFGNFNGMDTVSTVNTDWYLPLYGRY--------------------------------SIVGRSVIIHDTT-----------------------GSRWVCANIGYPGEVI--TAMARNFYTDVELPLSGVIGRSMVIHQ

>Danio.rerio_65_CuZnSOD

LSLSGYICAEIRSISEKTVNAVLNQG---IKGYFSFQQKSPFDL-TTITVNLTNLN--RRVGPYHVHQFPLPQMS-----------------------------------PSDSSCSNNNAGGHWN---------PFNVNQAPAYPPPKGSTHDRFEVGDLSSRHGSLENTSNFQATLIDWNLPLFGWN--------------------------------SIVGRSVVIHMPN-----------------------GTRFACSSIDYPGEVT--VAKAKNFFTDTTSWVLGMIGRSLVIHG

>Xenopus.tropicalis_67_CuZnSOD

LEFGKIICAKLKALRSKTASAHVSNG---VSGHFNFRQNSPLHP-TEIDLNLRNLK--GQFGEYSIHSLPVLARD-----------------------------------PGENLCNKNSTGDIWN---------PLGVNSASSYPTQPGSAHHLWEMGDLSGCHGSLQGYEEMRTKLIDWNLPLYGNN--------------------------------SVVGRSVVLSKAN-----------------------GTEWVCSTIRQEGDMV--VATARYLFTDSYSSLTGILGKALVIHG

>Sycon.ciliatum_68_CuZnSOD

IS-QRIACSDLLTLESREMSATFDQG---VKGVMVFSQQSPWDP-TWVTLDLSGLA--GQAAGYHVHEFPRSVLV-----------------------------------QYGSACSAQEVGGHWN---------PFRAPFPVTVPPGFASTPDLYEVGDLSGKHGSLANLQSVQQEMYDGQLPLFGPH--------------------------------SIAGRSIVIHRPN-----------------------APRWVCANIEMPQTAK-RIAVARFVYTDVFLPLQGVLGRSVVVHT

>Sycon.ciliatum_69_CuZnSOD

IS-QRIACSDLLTLESREMSATFDQG---VKGVMVFSQQSPWDP-TWVTLDLSGLA--GQAAGYHVHEFPRSVLV-----------------------------------QYGSACSAQEVGGHWN---------PFRAPFPVTVPPGFASTPDLYEVGDLSGKHGSLANLQSVQQEMYDGQLPLFGPH--------------------------------SIAGRSIVIHRPN-----------------------APRWVCANIEMPQTAK-RIAVARFVYTDVFLPLQGVLGRSVVVHT

>Sycon.ciliatum_70_CuZnSOD

FH-DRVVCSNIQELRSRRATAVFDGG---VRGRVELNQQSPWDN-TIVSVQLNGLM--MMAGGYHIHEWPVPLLL-----------------------------------STLTRCGSQAVGGHYN---------PFSAT-----PLPPGLTQDFYEVGDLSAKFGGLAGLNSLNATYSDTYITLFGPY--------------------------------SVVGRSIVLHNDS-----------------------SPRWQCATILLESTTL-SVARAKHFYTDVDLPVAAILGKSVVIHG

>Ciona.intestinalis_75_CuZnSOD

FW-TIIACTHINLQNPRSAVTRFASN---LRGTVTFTQDSPFDV-TMTSVDLLGVQ--FLNATYHIHKFPVPQRG-----------------------------------ASENKCSFNNVVGHYK---------PFGASSVG--PANAAGTHDQYEVGDLSGKYGKLTS-ATFAMNYTDWNLPLFGVH--------------------------------SILGRSVVVHKSS-----------------------SERYACANIVYNGAVV--TAVA-----------------------

>Amphimedon.queenslandica_71_CuZnSOD

LI--------ECCSANLTAIATVSRG---VTGTIIFTQNTPTSE-TNIRLTLTGLP---PLYQWHVHQYPF---------------------------------------ASAPSCSPPNTGGHFD---------PLGAFNQANYSTNCNKNNAACEIGDLSGKFGLLNV-SDLPFSEGDDTISLYGRH--------------------------------SIIGRSIVFHFSN-----------------------GTRFVCANILINSSQDVSRAYSRLFYSDIDLPLLIIENRSIVIHQ

>Xestospongia.bergquistia_74_CuZnSOD

IV--------SPTLGSLTAIATFSNG---ISGTITFAQNSSSSP-TSIHVNLTGLS---TLYQWHVHQYPF---------------------------------------TAAPSCSPANVGGHYD---------PLGASNNNNYKADCAMNKTICEIGDLSGKFGLLNT-SLLPLNINDSSLSLYGYQ--------------------------------SIIGRSVVLHLQN-----------------------GTRYVCANIDVSQPSNIEIEYVRLLYTDTDLPITAIIDRSVVIHQ

>Tethya.wilhelma_72_CuZnSOD

LP--------ASAHSQLTVSADFNRG---VKGSITFSQNDPQSS-TTISVNLTGLDAVSGPFQWHVHEYPYPNNF-----------------------------------TSGDICSAAVVGGHYD---------PLGANNVVNYSTVCNPSNQACEVGDLSGKFGPLPVDPTVTLSLDDQFLSLYGVY--------------------------------SIIGRSIVIHLND-----------------------NSRFICANIGYPDQDQIDILLVKQYYTDTDLPLVGIADRSVVIHE

>Tethya.wilhelma_73_CuZnSOD

LP--------ASAHSQLTVSADFNRG---VKGSITFSQNDPQSS-TTISVNLTGLDAVSGPFQWHVHEYPYPNNF-----------------------------------TSGDICSAAVVGGHYD---------PLGANNVVNYSTVCNPSNQACEVGDLSGKFGPLPVDPTVTLSLDDQFLSLYGVY--------------------------------SIIGRSIVIHLND-----------------------NSRFICANIGYPDQDQIDILLVKQYYTDTDLPLVGIADRSVVIHE

>Ephydatia.muelleri_111_CuZnSOD

-----------------------------------------------------------------------------------------------------------------------------------------------------------CALGDLSGRYGYLNSSSSFSA--SDADLSLYGAY--------------------------------TIFGRSVVLNW-------------------------STLFACANIGYPA-----VLYARLLYVDLLLPLLPIVGRSVVVHG

>Ephydatia.muelleri_107_CuZnSOD

-----------------------------------------------------GLE---LSYTWRVHQYPS---------------------------------------SGQHDCSYSAAGGCYD---------PQYVGQQPNYTALCAANQSLCEIGDLTGRHGPLTV--TRASVIEDSCLNLYGVS--------------------------------NIVGRSLVLNW-------------------------NEQLACTNIGYPTSTPATILYAKTFYMDLSLPFLD-INRSIVVVG

>Ciona.intestinalis_94_CuZnSOD

---------------------------------------QPFSA-TSVTANLAGLA--NTAGGYHIHILPLEAGS-----------------------------------SGVGACSTSVISGHYS---------PFDLPAIPN---ATVGTTDQYEVGDLSNKYGLLTSQASLSLPVLDDNLPLSTDV--------------------------------SVAGRSVLIHKAS-----------------------GTPWKCNSLEQPTGHV-IVAEVRKLFVDVDLPLNTVLGRSIVFHA

>Amphimedon.queenslandica_108_CuZnSOD

LV-SFAALHPPQTGRAILVAAYFDNG---IKGSVAFYQEKEHEP-VTITLSLSGLSQFGGRWGWHVHEYPINWAH-----------------------------------LERNPCNTEHIGGHYD---------PENRASDPNYAQLCMNNASLCEVGDLGGRHGHLR--GNQSYTFTDNTLNLYGPY--------------------------------SIVGRSLIIHRTN-----------------------GFRYACTNIEYNGISTIHTYRAQAFCTDTQLGIYPVNNTILVIED

>Xestospongia.bergquistia_109_CuZnSOD

VA-ALLLLAVPDNRIPINVGAYFDNH---ITGTIQFYQEREGKP-VVIHIVLNGLDQFSEMWGWHIHEYPINWAL-----------------------------------LESVSCGTPSVGGHYD---------PEKVGLASDYDQRCASDPKNCEVGDLSGRHGTLK--PNQTYTFTDSTLNLYGSY--------------------------------SLVGRSIVIHRTH-----------------------GYRWACANIEYDGHNSLHTFRAQGFCTDTQLGLYPYKDLVVGIQD

>Amphimedon.queenslandica_76_CuZnSOD

FS-LLLLLVAKGTEGQTYGRATFNRG---ITGYITFMPQPNNR--MMIMSNISGTN---ANHHWHVHLLPVDGSL-----------------------------------PPAMRCSNDLIGGHFD---------PLGAVSGSNYSMYCNPNNSACEVGDLSGKVGPLTS-GVRNYNETTGELVLFGVN--------------------------------GIIGRSIKIH--------------------------GTEDVCATIVSSTSPTITMLKASFFFQDPTLNLTG---GSLVINF

>Xestospongia.bergquistia_77_CuZnSOD

LS-CALLVMISTSQGQTYGIANFNRG---IQGTIKFMPNDNDH--VNITVDIQGTT---MDHVWHVHNFPVDTSL-----------------------------------PPQTRCHNDNIGGHFD---------PFGANAGSLYSTYCGPNNTVCEVGDLSGKVGNLTS-GTFNYIDKSGDLTLFGRY--------------------------------GIIGRSIKIH--------------------------GNDDVCATIYSSTNIKVTTLQAGFLYHDSFLNLTGVMGRSIALHS

>Tethya.wilhelma_78_CuZnSOD

AV-VYTAVASAQVANSHVAVVRFNRG---IQGNITFTELGNGN--VRIVANLQGLR---GFHNWHIHHYPVDQSL-----------------------------------DPAIQCRGVQIGGHYD---------PFGRSTDPNYDAQCAANQTLCEVGDLEGKFGPLPASGQVDVVDTTGQLALSGRY--------------------------------SVVGRPINIHDSV-----------------------DLHLECGTIRLLEGAEVTLLQA-----------------------

>Tethya.wilhelma_79_CuZnSOD

AV-VYTAVASAQVANSHIAVVRFNRG---IQGNITFTELGNGN--VRIVANLQGLR---GFHNWHIHHYPVDQSL-----------------------------------DPAIQCRGVQIGGHYD---------PFGRSSDPNYDAQCAANQTLCEVGDLEGKFGPLPASGQVDVVDTTGQLALSGRY--------------------------------SIVGRPINIHDSV-----------------------DSHLECGTIRLLEGAEVTLLQAAFFFTDSFLNLTV-TGRSLVVHV

>Tethya.wilhelma_80_CuZnSOD

AV-VYTAVASAQVANSHIAVVRFNRG---IQGNITFTELGNGN--VRIVANLQGLR---GFHNWHIHHYPVDQSL-----------------------------------DPAIQCRGVQIGGHYD---------PFGRSSDPNYDAQCAANQTLCEVGDLEGKFGPLPASGQVDVVDTTGQLALSGRY--------------------------------SIVGRPINIHDSV-----------------------DSHLECGTIRLLEGAEVTLLQAAFFFTDSFLNLTV-TGRSLVVHV

>Tethya.wilhelma_81_CuZnSOD

TL-SLLSLLCTAHAGYKIATATFNQG---IEGYITFTDLGNGS--IRINTNLDGLR---GMHNWHLHHFPVDQSL-----------------------------------SPDVQCGGQLIGGHYD---------PFGRNNDSNYDARCAADINMCEVGDLEGKFGPLPPDGQVDVIDTTGELTLRGRY--------------------------------SIVGRPINIHDHI-----------------------DSHLVCSTVRLYN-ATVTLLEAAFFFTDSALDLNV-VGRSLVIHI

>Tethya.wilhelma_82_CuZnSOD

TL-SLLSLLCTAHAGYKIATATFNQG---IEGYITFTDLGNGS--IRINTNLDGLR---GMHNWHLHHFPVDQSL-----------------------------------SPDVQCGGQLIGGHYD---------PFGRNNDSNYDARCAADINMCEVGDLEGKFGPLPPDGQVDVIDTTGELTLRGRY--------------------------------SIVGRPINIHDHI-----------------------DSHLVCSTVRLYN-ATVTLLEAAFFFTDSALDLNV-VGRSLVIHI

>Tethya.wilhelma_83_CuZnSOD

------------------------------------------------------------MHNWHIHHFPVDQSL-----------------------------------SPDVQCGGQLIGGHYD---------PFGRNNDSNYDARCAADTNMCEVGDLEGKFGPLPHDRPVQFVDTTGELTLNGRY--------------------------------SIVGRPINIHDHI-----------------------DSHLVCSTVRLYN---VTLLEAAFFFTDSALDLNV-VGRSLVIHV

>Tethya.wilhelma_84_CuZnSOD

------------------------------------------------------------MHNWHIHHFPVDQSL-----------------------------------SPDVQCGGQLIGGHYD---------PFGRNNDSNYDARCAADTNMCEVGDLEGKFGPLPHDRPVQFVDTTGELTLNGRY--------------------------------SIVGRPINIHDHI-----------------------DSHLVCSTVRLYN---VTLLEAAFFFTDSALDLNV-VGRSLVIHV

>Tethya.wilhelma_85_CuZnSOD

VA-SRTSMTAPWSIAQRSATARFNRG---IRGYITFTEEGPEN--VRIVANLDGLR---GLHDWHIHHYPVDQSL-----------------------------------EPTVQCSGRQVGGRYN---------PFNVNSDPNYITNCQNDQSLCEVGDLENKFGPLPQSGQFNGVDTSTQLDLTGRY--------------------------------SIVGRPVVIHQSD-----------------------TDHLECATIRFDDGAEVTLLQAAFFFTDSFLNLAV-TGRSIVVHV

>Tethya.wilhelma_86_CuZnSOD

VA-SRTSMTAPWSIAQRSATARFNRG---IRGYITFTEEGPEN--VRIVANLDGLRGVGGLHDWHIHHYPVDQSL-----------------------------------EPTVQCSGRQVGGRYN---------PFNVNSDPNYITNCQNDQSLCEVGDLENKFGPLPQSGQFNGVDTSTQLDLTGRY--------------------------------SIVGRPVVIHQSD-----------------------TDHLECATIRFDDGAEVTLLQAAFFFTDSFLNLAV-TGRSIVVHV

>Tethya.wilhelma_87_CuZnSOD

FC--------CAALASHSARVVFNRG---IRGYITFTEQEDGN--IRIVANLTGLRGGAALHLWHIHNFPVDQSL-----------------------------------DPADQCAINQLGGFYD---------PFGALANQNYSADCFNQNSLCAAGDLASKFMPLPMDGQLDIVDTTGQLSLSGRY--------------------------------SIVGRSLVIHVPD-----------------------ATPLECGTIRLVEGAQVRLMQA-----------------------

>Tethya.wilhelma_88_CuZnSOD

ME-------------------------------------------------------------------------------------------------------------------------------------PYKK---------------------------------------------------------------------------------------------------------------------------------------TLFEWAFFFTDSYLNLTV-VNRTVVVHL

>Tethya.wilhelma_89_CuZnSOD

ME-------------------------------------------------------------------------------------------------------------------------------------PYKK---------------------------------------------------------------------------------------------------------------------------------------TLFEWAFFFTDSYLNLTV-VNRTVVVHL

>Xestospongia.bergquistia_90_CuZnSOD

LL--TYVSAIDFSRTPLEATARINKG---VVGFVKFKTLNESA--IQITTNFSGLPINGGGLNWHVHRFPVDLTI-----------------------------------NPDHRCLNIYVGGHYD---------PLMARANENYTMNCSPGNFACEIGDLTGKFGQLQN-GYFVHVDNTGLLELGGLR--------------------------------GIVGRSIVVHASN-----------------------GINFVCGTIRSNINVEVITLSAAFFYTDIFLNLTGVNERSIAIHA

>Tethya.wilhelma_91_CuZnSOD

LS-TSLASQVASQEPENVATAVFGNG---ITGWIRFFEYEDDR--VVIIVNMVGFA---SNASWSMHDLPVDLTL-----------------------------------SPSEKCSEDYLGPVYD---------PMGMY-----------NATLCEAGDLGASYGIDD---------TPQLFTLSGPT--------------------------------SIVGRSLVVKNSE-----------------------G--VLCATIRSNSDDDIVTLKAAFFFTDSSLTLTI-EDLTLGIYS

>Tethya.wilhelma_92_CuZnSOD

LS-TSLASQVASQEPENVATAVFGNG---ITGWIRFFEYEDDR--VVIIVNMVGFA---SNASWSMHDLPVDLTL-----------------------------------SPSEKCSEDYLGPVYD---------PMGMY-----------NATLCEAGDLGASYGIDD---------TPQLFTLSGPT--------------------------------SIVGRSLVVKNSE-----------------------G--VLCATIRSNSDDDIVTLKAAFFFTDSSLTLTI-EDLTLGIYS

>Sycon.ciliatum_96_CuZnSOD

LA--------VIGELALRAVAEFSNG---VRGSLVFEDSSSSSG-NWVQIDSHGIDH-LGPLTWHVHLYPFKYTI-----------------------------------SPMHRCTS--TGGHYD---------PHGKATAVRYAQLCKGNPRDCEVGDLSGKFGPLRR----TLRDRTPNMSVA-----------------------------------ELIGRSVVLHVFS-----------------------GERFACANIVEVNVDRMRTLEALLYTVDLLSPLDL-RQRDLVLSD

>Sycon.ciliatum_98_CuZnSOD

LA--------VIGELALRAVAEFSNG---VRGSLVFEDSSSSSG-NWVQIDSHGIDH-LGPLTWHVHLYPFKYTI-----------------------------------SPMHRCTS--TGGHYD---------PHGKATAVRYAQLCKGNPRDCEVGDLSGKFGPLRR----TLRDRTPNMSVA-----------------------------------ELIGRSVVLHVFS-----------------------GERFACANIVEVNVDRMRTLEALLYTVDLLSPLDL-RQRDLVLSD

>Sycon.ciliatum_97_CuZnSOD

LA--------VIGELALRAVAEFSNG---VRGSLVFEDSSSSSG-NWVQIDSHGIDH-LGPLTWHVHLYPFKYTI-----------------------------------SPMHRCTS--TGGHYD---------PHGKATAVRYAQLCKGNPRDCEVGDLSGKFGPLRR----TLRDRTPNMSVA-----------------------------------ELIGRSVVLHVFS-----------------------GERFACANIVEVNVDRMRTLEALLYTVDLLSPLDL-RQRDLVLSD

>Sycon.ciliatum_99_CuZnSOD

LA--------VIGELALRAVAEFSNG---VRGSLVFEDSSSSSG-NWVQIDSHGIDH-LGPLTWHVHLYPFKYTI-----------------------------------SPMHRCTS--TGGHYD---------PHGKATAVRYAQLCKGNPRDCEVGDLSGKFGPLRR----TLRDRTPNMSVA-----------------------------------ELIGRSVVLHVFS-----------------------GERFACANIVEVNVDRMRTLEALLYTVDLLSPLDL-RQRDLVLSD

>Sycon.ciliatum_100_CuZnSOD

FI-VVMSLSKTSCRAQLNARADFHNG---VGGNILFSWNPQS---HQMTINTKGLS---PGLTWHVHEFPF---------------------------------------KLNPDCSASSTGGHFD---------PLGRASAPNYAQICQSMFRDCELGDLSGKFGTLQL-----REYETPNLSLSGNF--------------------------------GFVGRSVVIHR-------------------------GARWVCANIEPVLSNKRRLLVAKSLYTTMFYPIDF-IRRSLVLT-

>Sycon.ciliatum_101_CuZnSOD

LP-----------GLSVAQSAYFSGG---VAGHVDISGSRASTP-EPITITLTGFPS-GGALRWGVNERAF---------------------------------------RPGNACGTESVGGLWD---------PEMKATAQDYATNCLSSRTDCAAGDLSGKFGPPSS----SGTFADSSLRLI-----------------------------------DFLGRSLVLRYSN-----------------------GTALACANIVYVYMPAPSAFQALSFSGNSHAPVNG---KSIILG-

>Sycon.ciliatum_102_CuZnSOD

IT------------TMPCAQAYFTGG---VSGRIIFSGSSSS---GGVTINTNGYQQ-SGGILWHVHQFAF---------------------------------------PTGPDCGAAATGGPWD---------PGSRMANPNYASQCVKSWSVCSLGDLSGKIGPLSS-GQFA----------SGLL--------------------------------DLLGRAVVLHRSD-----------------------GTRFACANIQYTEASEPQVYWALSYSDATAVQFSS---RSIVLG-

>Ephydatia.muelleri_106_CuZnSOD

VV----GVLFVVAYSQSAAVATFDNG--VVKGTVRFTAADG----LQVQVSLSGLS---GTHPWHVHVNQLNSTY-----------------------------------GYPLVCDY--VDAHFD---------PFNANTSTNYTQRCSANQSLCEAGDLAGKFGSIPVNGTYGGTYSDTFLDTTGVS--------------------------------DCVGRSVVVHNFT-----------------------GSPMRCANVYAVS--------------------------------

>Tethya.wilhelma_110_CuZnSOD

VV-ALLATLYGRFGVEKNVQAEFDGG---VRGYVQFFQERPGDP-VEINVNLQGLD---QPYPWRIHTYPSRFAL-----------------------------------LADYPCGEAEVGPVLNH--------PEG------------------GVGDLFAQHG-----GDMPQTFVNPHITLCGPE--------------------------------SIVGRSLRIDR-------------------------GDEWICANIQYHGARV-ERLRARAFCVDGTVPCNF-YVTSLVIRD

>B.floridae_93_CuZnSOD*

LG-SVTADGKVRRLADKLALLKQAPGDSCLSNRTNYTQD------VQISRIMNRLD--VDGSTHDVHH------------------------------------------------------------------GPKGFNEQGNYATRCKPGNLRCELGDLSGKQGKYA--VGGGRVYTDVDIPLYGEL--------------------------------TVVGRSIVIHEAN--------------------------------------------------------------------

>Sycon.ciliatum_104_CuZnSOD

LAVLCIVVADHEAKGLDKAIAFFANG---FHGPVSYLNR------TTLHVDMRKLN---YPLAIDVHRLSF---------------------------------------TVGSPCES--IGDHYN---------PTNNP----------------MLGDLSGSPGGLNATTRFA---IETNLTFTGHH--------------------------------SIIGRSVAFHEGN-----------------------ETAVACANIVHANDNDQDTLEA--------LQAAPLIGKSLAIHR

>Oscarella.carmela_95_CuZnSOD

VV--------TASVVKGAHLVNIDNG---IKGVIRINATEGS---GTVAFDFHDLHTLANNMTAAIHQLPA---------------------------------------PYRSKCQPTATGLIYD---------PTDAMSGSNYTKACQSDATKCAVGDLGLRLGKINHESVLKTVFPGNNWYLSGSD--------------------------------GIVGRTLVLYNYT-----------------------GYAFACGIIERHVSKKEVIRKSHYFFIDTNLEVDF-VQKAVAVTD

>Sycon.ciliatum_103_CuZnSOD

LTYCSLPSTLAQTCQDERAVAYFNNG---FRGRVIISKDA-----GELSFNASGLQLATGQFSLAIHELSA---------------------------------------FVGSSCQG--IGAVYN---------PTSAQ-------------AGMELGGLSTLPALAN-----QVLSVTTGLNMINPY--------------------------------GVIGRTLVIQATS-----------------------GTATSCATIQHVYHNYEVTHVATSLCLDSQLPTGT--ERSLAVD-

>P.nodorum_CuZnSOD_3

LAAAQVAQHITAQTSMPASTATMPVG-TTVSGYIEGVGSPSG---VRFTVKLQNLPEKYGPFNWHIHALPV---------------------------------------PADGNCTA--TLGHLD---------PTNRG------EMCDAAAETCQAGDLAGKHGGIMAAGSFETAFVEKYLTTTGSF--------------------------------SFAGLGFVLHSMN-----------------------TTRLTCANFVQVNG-------------------------------

>P.nodorum_CuZnSOD_4

LAAASAVTPQVAGNPAGASVATLPKG-SPLRGAITAVSAPDGVG-VLFSVSFSGLPATGGPFMYHLHEKPV---------------------------------------PENGNCTA--TGAHLD---------PYKRG------EICDASKETCQTGDLSGKHGNTS--GEFSAEYVDPYSALPGNN--------------------------------AYFGLSFVLHLAN-----------------------KTRIGCANFSQGAP-------------------------------

>C.crispus_CuZnSOD_1

LLVAALLAAVSGCSNLPTLVCTVQPTGYSVEGVVYFRRRQPTCY-VRIMAAVAGLT--NPQHGFHVHTYG----------------------------------------DSVSDGSS--TGGHFN---------VAGDE-------HGLPDDEIRHWGDLGNLINDGK--GNAEYDRVDKVVRLG-----------------------------------ALVGRGITIHEDQDS-------------SEQPTGASGTRIGC-VIGYANPEVASR--------------------------

>C.crispus_CuZnSOD_2

LAVSCMPSTAARCLNSLPLVCNVVATGYNCTGYVKFNRRMPSCH-ARVRANLKGLS--PGRHGFHIHTYG----------------------------------------DRGLDGSS--TGGHFN---------VKGDD-------HGYASSPARHMGDLNNVMARAN--GEAKYSREDNVIRLG-----------------------------------AIRGRGITIHQDRDP-------------GSQPSGAAGDRVGC-VIGVVDAKI-----------------------------

>P.nodorum_CuZnSOD_1

---------------MVKAVAVLR-GDSNVKGTVTFEQENESSP-TKISWDITGND--NAERGMHVHAFG----------------------------------------DNTNGCTS--AGPHFN---------PHNKT-------HGAPEDEERHVGDLGNFKTDGQ--GNAQGSVSDKLIKLIGSE--------------------------------SVIGRTIVVHGGTDDLGRGGHE------ESKKTGNAGPRPACGVIGISN--------------------------------

>Podospora.anserina_1_SODCuZn

---------------MVKAVAVVR-GDSKVSGSVVFEQETENGP-TTITWDITGHD--NAKRGMHIHTFG----------------------------------------DNTNGCTS--AGPHFN---------PHGKT-------HGNRTDENRHVGDLGNIETDAQ--GNSKGTVTDNLIKLIGPE--------------------------------SVIGRTVVVHAGTDDLGKGDTE------ESLKTGNAGARPACGVIGISA--------------------------------

>Podospora.anserina_2_SODCuZn

---------------MVKAVAVVR-GDSKVSGSVVFEQETENGP-TTITWDITGHD--NAKRGMHIHTFG----------------------------------------DNTNGCTS--AGPHFN---------PHGKT-------HGNRTDENRHVGDLGNIETDAQ--GNSKGTVTDNLIKLIGPE--------------------------------SVIGRTVVVHAGTDDLGKGDTE------ESLKTGNAGARPACGVIGISA--------------------------------

>Acanthaster.planci_5_CuZnSOD

LA-------ALLVIGSVRLVCVLQPGGQQIRGQIQLTQEQQGGP-VKVTGDVTGLA--PGKHGFHVHEFG----------------------------------------DYTGGCTS--TGGHFN---------PFKKN-------HGAPTDEERHIGDLGNVEAGSD--GTVSVNITDKMISLIGPN--------------------------------SIIGRAVVVHADVDDLGKGGHE------LSLTTGNAGGRLSCGVIGISK--------------------------------

>Branchiostoma.floridae_6_CuZnSOD

--------------------------------------QSPGGP-VRVTGEVQGLT--EGPHGFHVHEFG----------------------------------------DYTNGCTS--MGAHYN---------PIGTN-------HGGPNDAVRHVGDLGNIVANVA--GVAQVDITDNQLSLYGAD--------------------------------SIIGRGVVVHADEDDLGKGGHE------LSDTTGNSGGRLACGIIGITK--------------------------------

>Branchiostoma.floridae_17_CuZnSOD

---------------SLKAVCVLV--GETVKGTVTFTQASSDSP-VEVTGTISNLT--PGKHGFHIHEFG----------------------------------------DTTNGCTS--AGSHFN---------PAKKN-------HGGPQDAERHVGDLGNVEVGDD--GVATINITDSQLQLTGPN--------------------------------SIVGRAVVVHAGEDDLGKGGFE------DSLTTGHAGGRLACGVIGITKQA------------------------------

>Capitella.teleta_7_CuZnSOD

---------------VLKAICILK-GSTPVEGTINFTQE-GDGP-VTLEGQIAGLA--PGKHGFHVHEFG----------------------------------------DNTNGCVS--AGSHFN---------PFGKT-------HGGPDSEVRHVGDLGNAVAGDD--GIAKINITDDQVTLTGPH--------------------------------SVIGRTMVVHADPDDLGLGGHE------LSPTTGNAGGRLACGVIGITK--------------------------------

>Sycon.ciliatum_8_CuZnSOD

---------------SVNAACVLA-GDSAVKGVITFTQA--EGA-TTVTGEVTGLA--PGNHGFHVHVYG----------------------------------------DNTNGCTS--AGPHFN---------PSNKT-------HGAPCDENRHAGDLGNVVAGDD--GVAKVNITDSQIPLSGPN--------------------------------SIIGRTVVVHADPDDLGKGGHE------LSLSTGNAGARVACGVIGIAK--------------------------------

>Arabidopsis.thaliana_1_SODCuZn

---------------MAKGVAVLN-SSEGVTGTIFFTQE-GDGV-TTVSGTVSGLK--PGLHGFHVHALG----------------------------------------DTTNGCMS--TGPHFN---------PDGKT-------HGAPEDANRHAGDLGNITVGDD--GTATFTITDCQIPLTGPN--------------------------------SIVGRAVVVHADPDDLGKGGHE------LSLATGNAGGRVACGIIGLQG--------------------------------

>Ephydatia.muelleri_18_CuZnSOD

---------------MSQAVCVLE--GQTVGGTIFFTEN-SDGS-THVTGMVTGLT--PGDHGFHVHEYG----------------------------------------DYSDGCVS--AGEHFN---------PYKKQ-------HGGPNDKERHAGDLGNITANES--GEALVDIVDKQIPLTGPN--------------------------------SIVGRSIVVHADKDDYGRGGFD------DSKTTGHSGARLACGVIGIAANQVSVVV-------------------------

>Ephydatia.muelleri_19_CuZnSOD

---------------MAQAVCVLE--GQTVRGTISFTES-GDGA-THVTGTVTGLT--PGDHGFHVHEYG----------------------------------------DYSGGCVS--AGPHFN---------PYKKQ-------HGGPNDEERHAGDLGNITANES--GEALVDIVDKQIPLTGPN--------------------------------SIVGRSIVVHADKDDYGRGGFD------DSKTTGHAGARLACGVIGIAKPVVA----------------------------

>Danio.rerio_9_CuZnSOD

---------------VNKAVCVLK-GTGEVTGTVYFNQEGEKKP-VKVTGEITGLT--PGKHGFHVHAFG----------------------------------------DNTNGCIS--AGPHFN---------PHDKT-------HGGPTDSVRHVGDLGNVTADAS--GVAKIEIEDAMLTLSGQH--------------------------------SIIGRTMVIHEKEDDLGKGGNE------ESLKTGNAGGRLACGVIGITQ--------------------------------

>Homo.sapiens_10_CuZnSODSOD1

---------------AT--------------------KAESNGP-VKVWGSIKGLT--EGLHGFHVHEFG----------------------------------------DNTAGCTS--AGPHFN---------PLSRK-------HGGPKDEERHVGDLGNVTADKD--GVADVSIEDSVISLSGDH--------------------------------CIIGRTLVVHEKADDLGKGGNE------ESTKTGNAGSRLACGVIGIAQ--------------------------------

>Homo.sapiens_11_CuZnSODSOD1

---------------ATKAVCVLK-GDGPVQGIINFEQKESNGP-VKVWGSIKGLT--EGLHGFHVHEFG----------------------------------------DNTAGCTS--AGPHFN---------PLSRK-------HGGPKDEERHVGDLGNVTADKD--GVADVSIEDSVISLSGDH--------------------------------CIIGRTLVVHEKADDLGKGGNE------ESTKTGNAGSRLACGVIGIAQ--------------------------------

>Lingula.anatina_12_CuZnSOD

---------------ALKAVCVLK-GASDVIGTVYFEQTSADGP-CKVTGEVTGLK--EGLHGFHVHQFG----------------------------------------DNTNGCTS--AGPHFN---------PSGKT-------HGGPDDENRHFGDLGNIKAGSD--GKATVAITDKLVTLTGPN--------------------------------SVIGRTIVVHEGQDDLGKGGNE------ESLKTGNAGGRLACGVIGITH--------------------------------

>Lingula.anatina_13_CuZnSOD

---------------QWVGVCV--------------------------------------------------------------------------------------------------------------------------------------HFGDLGNIKAGSD--GKATVAITDKLVTLTGPN--------------------------------SVIGRTIVVHEGQDDLGKGGNE------ESLKTGNAGGRLACGVIGITH--------------------------------

>Nematostella.vectensis_15_CuZnSOD

---------------PIQAVCCMS-GTEGVKGTIKFVQEAEGKP-CKITGTIEGLK--AGNHGFHIHVYG----------------------------------------DNTNGCVS--AGPHFN---------PFKKE-------HGGPSDENRHVGDLGNVVAGDD--GKACIDMTDALVTLVGEH--------------------------------SVVGRSVVVHADEDDLGRGGHE------DSKTTGHAGGRLACGVIGITQAS------------------------------

>Nematostella.vectensis_14_CuZnSOD

---------------VIRGVCCLV-GDNEVKGVIHFTQQAPDGP-CTLRGRITGLT--EGKHGFHIHEFG----------------------------------------DNTNGCTS--AGAHYN---------PHGKM-------HGAPEDKDRHLGDLGNIEADAN--GIADVSITDCLVSLTGQC--------------------------------SIIGRSLVVHEGMDDLGAGGHE------LSLTTGNAGGRVACGVIGIAL--------------------------------

>Trichoplax.adhaerens_SODCuZn

---------------ALKAVCCLQ--GPVVSGTIFFQQESGTGP-IRISGEVKGLA--PGKHGFHVHEFG----------------------------------------DNTQGCTS--AGGHYN---------PHKKV-------HGAPGDEIRHVGDLGNIEANEQ--GVASINMTDRMVTLTGPY--------------------------------SCIGRTIVVHEGVDDLGKGGHE------LSLTTGNAGARVACGVIGITKC-------------------------------

>Arabidopsis.thaliana_2_SODCuZn

--------------MAKKAVAVLK-GTSDVEGVVTLTQD-DSGP-TTVNVRITGLT--PGPHGFHLHEFG----------------------------------------DTTNGCIS--TGPHFN---------PNNMT-------HGAPEDECRHAGDLGNINANAD--GVAETTIVDNQIPLTGPN--------------------------------SVVGRAFVVHELKDDLGKGGHE------LSLTTGNAGGRLACGVIGLTPL-------------------------------

>Drosophila.melanogaster_16a_CuZnSODSOD1

---------------VVKAVCVIN---GDAKGTVFFEQESSGTP-VKVSGEVCGLA--KGLHGFHVHEFG----------------------------------------DNTNGCMS--SGPHFN---------PYGKE-------HGAPVDENRHLGDLGNIEATGD--CPTKVNITDSKITLFGAD--------------------------------SIIGRTVVVHADADDLGQGGHE------LSKSTGNAGARIGCGVIGIAKV-------------------------------

>Drosophila.melanogaster_16b_CuZnSODSOD1

---------------VVKAVCVIN---GDAKGTVFFEQESSGTP-VKVSGEVCGLA--KGLHGFHVHEFG----------------------------------------DNTNGCMS--SGPHFN---------PYGKE-------HGAPVDENRHLGDLGNIEATGD--CPTKVNITDSKITLFGAD--------------------------------SIIGRTVVVHADADDLGQGGHE------LSKSTGNAGARIGCGVIGIAKV-------------------------------

>Mnemiopsis.leidyi_27_CuZnSOD

----------------MNAICVLR-NDKESFGCIKFSQASEGAP-TTLKVEISSLT--EGEHGFHMHQFG----------------------------------------DNTNGCIS--AGPHFN---------PFGKT-------HGGPTDEVRHVGDLGNVVVDGS--GVCSTEITDPQVSLFGAN--------------------------------SIIGRTMVLHAGVDDLGKGGNE------ESLKTGNAGGRVACGIVGLCS--------------------------------

>Dictyostelium.discoideum_1_SODCuZn

---------------SKTAVCVIK--GEKVNGVVKFTQENKDSP-VTVNYDITGLE--KGEHGFHVHAFG----------------------------------------DTTNGCVS--AGPHFN---------PFGKN-------HGAPSDEDRHVGDLGNIVADGE--SNTKGTISDKIISLFGEH--------------------------------TIVGRTMVVHADQDDLGKGGKP------DSLTTGAAGARLGCGVIGVSQ--------------------------------

>Dictyostelium.discoideum_3_SODCuZn

----------------MSAICVIK--GDGVDGIINFKQNDNKSP-VIISGVISGLK--EGKHGFHVHEFG----------------------------------------DTTNGCLS--AGAHFN---------PFKKE-------HGSPNDENRHVGDLGNIESNKD--KKSIINITDNIITLFGQN--------------------------------SIIGRSIVVHDKEDDLGRGNSQ------DSKITGNAGSRLGCGIIALSKI-------------------------------

>Caenorhabditis.elegans_29_CuZnSODSOD1

LL-------TAIFPQSNRAVAVLR--GETVTGTIWITQKSENDQ-AVIEGEIKGLT--PGLHGFHVHQYG----------------------------------------DSTNGCIS--AGPHFN---------PFGKT-------HGGPKSEIRHVGDLGNVEAGAD--GVAKIKLTDTLVTLYGPN--------------------------------TVVGRSMVVHAGQDDLGEGGKE------ESKKTGNAGARAACGVIALAAPQ------------------------------

>Caenorhabditis.elegans_30_CuZnSODSOD1

---------------SNRAVAVLR--GETVTGTIWITQKSENDQ-AVIEGEIKGLT--PGLHGFHVHQYG----------------------------------------DSTNGCIS--AGPHFN---------PFGKT-------HGGPKSEIRHVGDLGNVEAGAD--GVAKIKLTDTLVTLYGPN--------------------------------TVVGRSMVVHAGQDDLGEGGKE------ESKKTGNAGARAACGVIALAAPQ------------------------------

>Caenorhabditis.elegans_31_CuZnSODSOD1

---------------SNRAVAVLR--GETVTGTIWITQKSENDQ-AVIEGEIKGLT--PGLHGFHVHQYG----------------------------------------DSTNGCIS--AGPHFN---------PFGKT-------HGGPKSEIRHVGDLGNVEAGAD--GVAKIKLTDTLVTLYGPN--------------------------------TVVGRSMVVHAGQDDLGEGGKE------ESKKTGNAGARAACGVIALAAPQ------------------------------

>Caenorhabditis.elegans_32_CuZnSODSOD5

IL-------SAVLPQSKRAVAVLR--GTAVFGTVWLTQKAEGEE-TEFEGEIKGLS--PGLHGFHIHQYG----------------------------------------DSTDGCTS--AGPHFN---------PCKMN-------HGGRDSVVRHVGDLGNVEAGAD--GVAKIKFSDKVVSLFGAN--------------------------------TVIGRSMVVHVDRDDLGQGDKE------ESLKTGNAGARAACGVIALAAPA------------------------------

>Ciona.intestinalis_24_CuZnSOD

---------------VLEAVCVMK-GSESVSGTIKFSQVGDGEP-CKISGSLTGLA--AGKHGFHIHEFG----------------------------------------DHTNGCTS--TGGHFN---------PQKCD-------HGAPEAEVRHFGDLGNVTADSS--GVAEVNISDKYVTLTGIN--------------------------------SVIGRAVVVHADVDDLGLTSHP------QSKTTGNAGGRLACGVIGITNHR------------------------------

>Dictyostelium.discoideum_2_SODCuZn

---------------MVKAICVVK--GAVVNGTIIFSQENEGSP-VYVNGTISGLS--GGLHGFHIHEFG----------------------------------------DTSNGCLS--AGAHFN---------PFHVE-------HGGPNSAIRHVGDLGNITSPSS--KVANVLIQDNVISLFGDL--------------------------------SIIGRTLVVHENQDDLGLGGN-------LSKTTGNAGARVACGILAKI---------------------------------

>Dictyostelium.discoideum_4_SODCuZn

---------------MVNAIVIIK--GLGVEGKVTLSQECEGSP-IYINGTVSGLT--PGQHGMHVHEFG----------------------------------------DTSNGCIS--AGDHYN---------PLHRE-------HGSPLDVERHIGDLGNIKALSN--GVATISIRDTIMSLFGDI--------------------------------SVMGRTMVIHSDRDDYGRGNFP------DSKTAGHSGKRVGCGIIAKI---------------------------------

>Tethya.wilhelma_20_CuZnSOD

LI-------AVCVLVNTAAVCVLE--GAEVKGTISFTPVEGSSA-VRVTGQVTGLS--PGNHGFHIHQFG----------------------------------------DYSAGCVS--AGPHFN---------PAGKE-------HGGPTDEERHVGDLGNIVADAS--GTATVAITDSQLTLSGPN--------------------------------SIIGRSVVVSDSTTEYTRLQF------------------------------------------------------------

>Tethya.wilhelma_21_CuZnSOD

--------------------------------MLSFLSTQGSSA-VRVTGQVTGLS--PGNHGFHIHQFG----------------------------------------DYSAGCVS--AGPHFN---------PAGKE-------HGGPTDEERHVGDLGNIVADAS--GTATVAITDSQLTLSGPN--------------------------------SIIGRSVVVSDSTTEYTRLQF------------------------------------------------------------

>Tethya.wilhelma_22_CuZnSOD

---------------STVGVCVLE---GEVKGTIRFQPE--GSA-VKVTGEVTGLK--PGKHGFHIHQFG----------------------------------------DYSAGCVS--AGSHFN---------PAGKE-------HGGPTDEERHVGDLGNIVADDS--GKAVVAITDSQLTLSGPN--------------------------------TIIGRSVVSLDDSDSMYFGDED------PQLLTGTAGTRQGCGLVELTHAALLGLS-------------------------

>Tethya.wilhelma_23_CuZnSOD

---------------STVGVCVLE---GEVKGTIRFQPE--GSA-VKVTGEVTGLK--PGKHGFHIHQFG----------------------------------------DYSAGCVS--AGSHFN---------PAGKE-------HGGPTDEERHVGDLGNIVADDS--GKAVVAITDSQLTLSGPN--------------------------------TIIGRSVVSLDDSDSMYFGDED------PQLLTGTASTSK-------------------------------------VL--

>Xestospongia.bergquistia_25_CuZnSOD

---------------KAKGGGTID----ENMGLVNSKHQTDSGL-CYITGEVTGLT--PGKHGFHIHEFG----------------------------------------DYSAGCVS--AGGHFN---------PHRKN-------HGGPDDTDRHAGDLGNIVADDS--GKATINITDKQIPLSGEN--------------------------------NIIGRSVVVHADPDDLGKGGFP------DSLTTGHAGGRLSCGVIGYAKS-------------------------------

>Amphimedon.queenslandica_26_CuZnSOD

--------------MVARAVCILA-SSDDVKGTIEFIQN-EQGI-TKVTGKVTSLA--PGDHGFHIHQFG----------------------------------------DYTSGCVS--AGSHFN---------PAGKN-------HGGPKDGERHAGDLGNITSTG---GDTEIELYDDQIPLTGPN--------------------------------SIIGRSVVVHADPDDLGKDGHP------DSLTTGHAGARLACGVIGSTKLQ------------------------------

>Strongylocentrotus.purpuratus_28_CuZnSOD

---------------SVKAVCMLV--GEAVKGRIEFEQGEGSNS-VSV-----------------------------------------------------------------KGCVS--AGGHFN---------PFGKE-------HGAPEDEMRHVGDLGNIIADAS--GKVDVNLSDKLLSLSGPQ--------------------------------SIIGRAVVVHADVDDLGKGGHA------TSKTTGNAGGRLACGVIGIQA--------------------------------

>Fonticula.alba_CuZnSOD_1

---------------MTKAVAVLN-GLNGLKLVATFSQQSPDSP-TVIDIEGSGIK--PGLHGFHVHEFG----------------------------------------DTTNGCVS--AGPHFN---------PHGHT-------HGAPSAAIRHVGDLGNLSVDET--GSVKVTITDHLVSLSGVN--------------------------------NVVGRALVLHADVDDLGLGGHE------LSATTGNAGDRIACGVIGITK--------------------------------

>Acanthaster.planci_1_CuZnSOD

---------------------------------------------MNVSVQLTGLD--TSLHGFHVHQVG----------------------------------------DLSNGCAS--TGGHFN---------PFDEQ-------HGGPSDTQRHVGDFGNVERDSV--GSVNEEFSDEVASLVGAD--------------------------------TIIGRAIVLHKDTDDLGRGDYD------DSLTTGHAGARLACCII------------------------------------

>Lingula.anatina_4_CuZnSOD

ILLIWTSCQQQLARMYTVGHCDMRPGVHGISGDIYLVQR--GNL-LEVRVNISGLP--EGKHGLHVHTYG----------------------------------------DLSDGCAS--TGGHFN---------PANVR-------HGSPTDNKRHVGDWGNVERDSD--GNVVTAFLDSVASLWGPN--------------------------------TIIGRAIVIHASEDDLGRGGDK------GSSLSGNAGPRLACCVIGISNGNNLLQQ-------------------------

>Branchiostoma.floridae_2_CuZnSOD

-------------------------------------------------------------YGVTVHELG----------------------------------------DQSKGCTS--TRDHFN---------PFNLG-------HGGPKDEDRHLGDWGNVEADST--GEVNAIIKDEVASLVGPY--------------------------------SIIGRTIVIHANEDDLGRGETA------ESKKTGNVGKRLACCVLG-----------------------------------

>Acanthaster.planci_3_CuZnSOD

MQ-------NPEGDGNVYAHCRMVPGIEPIVGDINMRQRAVGGQ-MDVKVFLYGMT--PKVHGIHVHTYG----------------------------------------DLGNGCGS--AGGHYN---------PGGNN-------HSAPYDKDRHMGDWGNIQVDDF--GMVEHTFSDKVATLVGPN--------------------------------SILGRTIVIHIGEDDLGKGGTA------DSKTTGNAGGRLGCCVIGHSDGSAWANF-------------------------

>Lingula.anatina_35_CuZnSOD

VLVFGCGWSESLAMQGVFAHCALKPGTVNVHGNIDMIQR--QGV-LEVRVNISGLP--DGEHGLHVHAFG----------------------------------------DLSGGCGS--TKGHYN---------PTGVT-------HGAPTDVVRHIGDWGNVPQDKN--GQIVTSFFDGIASLVGKN--------------------------------NIVGRAIVLHTGKDDLGRGGNA------ASLANGNAGPRLGCCVIGITNGQRVAVP-------------------------

>Nematostella.vectensis_41_CuZnSOD

-----------------------------------------------INVRLRGVP--PLVHGFHIHKSG----------------------------------------DITKGCQS--AKGHFN---------PYGKT-------HAGPRKRDRHVGDLGNVWSDYH--GNVRTSFFDHMVSLYGPD--------------------------------SVIGRSIVLHAERDDLGRGGYT------GSLATGNAGARLACCVIVH----------------------------------

>Capitella.teleta_37_CuZnSOD

II-LAARVAVQDKESYIYAQCQVAVAINHVIGTIDLRQTVGRAV-SEIRLNLTGFAADDGYHGFHVHELG----------------------------------------DLSNGCDS--TGSHYN---------PLDVD-------HGAPHDSNRHIGDLGNIEEDLG--GNCIRTITDTLVTLQGRF--------------------------------SVIGRALVIHETYDDLGRSGVD------DSLTTGNAGARLSCCVIGLTDDQHWD---------------------------

>Capitella.teleta_38_CuZnSOD

-------------------------------------------------------------HGIAIHQYG----------------------------------------DLSEGCTN--LGDHFN---------PLNVN-------HGGRLQSQRHVGDLGNIHVNLD--GSASSELKDYLIQLQGRY--------------------------------TVVGRSVAIHELTDDLGKGGDS------DSSTNGHSGSAIAC---------------------------------------

>Drosophila.melanogaster_36a_CuZnSODSOD3

VVSLALCATAQTRNMPIQAIAYLI-GPTQVKGNVTFTQNDCGQN-VHVRVQLEGLK--EGKHGFHIHEKG----------------------------------------DLTNGCIS--MGAHYN---------PDKVD-------HGGPDHEVRHVGDLGNLEANST--GIIDVTYTDQVITLTGKL--------------------------------GIIGRGVVVHELEDDLGLGNHT------DSKKTGNAGGRIACGVIGINGPSVPAPA-------------------------

>Drosophila.melanogaster_36b_CuZnSODSOD3

VVSLALCATAQTRNMPIQAIAYLI-GPTQVKGNVTFTQNDCGQN-VHVRVQLEGLK--EGKHGFHIHEKG----------------------------------------DLTNGCIS--MGAHYN---------PDKVD-------HGGPDHEVRHVGDLGNLEANST--GIIDVTYTDQVITLTGKL--------------------------------GIIGRGVVVHELEDDLGLGNHT------DSKKTGNAGGRIACGVIGIK---------------------------------

>Drosophila.melanogaster_36c_CuZnSODSOD3

VVSLALCATAQTRNMPIQAIAYLI-GPTQVKGNVTFTQNDCGQN-VHVRVQLEGLK--EGKHGFHIHEKG----------------------------------------DLTNGCIS--MGAHYN---------PDKVD-------HGGPDHEVRHVGDLGNLEANST--GIIDVTYTDQVITLTGKL--------------------------------GIIGRGVVVHELEDDLGLGNHT------DSKKTGNAGGRIACGVIGIK---------------------------------

>Drosophila.melanogaster_36e_CuZnSODSOD3

VVSLALCATAQTRNMPIQAIAYLI-GPTQVKGNVTFTQNDCGQN-VHVRVQLEGLK--EGKHGFHIHEKG----------------------------------------DLTNGCIS--MGAHYN---------PDKVD-------HGGPDHEVRHVGDLGNLEANST--GIIDVTYTDQVITLTGKL--------------------------------GIIGRGVVVHELEDDLGLGNHT------DSKKTGNAGGRIACGVIGIK---------------------------------

>Drosophila.melanogaster_36d_CuZnSODSOD3

VVSLALCATAQTRNMPIQAIAYLI-GPTQVKGNVTFTQNDCGQN-VHVRVQLEGLK--EGKHGFHIHEKG----------------------------------------DLTNGCIS--MGAHYN---------PDKVD-------HGGPDHEVRHVGDLGNLEANST--GIIDVTYTDQVITLTGKL--------------------------------GIIGRGVVVHELEDDLGLGNHT------DSKKTGNAGGRIACGVIGINSDVDEWPC-------------------------

>Caenorhabditis.elegans_33_CuZnSODSOD4

VLILALSVCIEAASEVIRARAIFKAGKTELIGTIDFDQS--GSF-LKLNGSVSGLA--AGKHGFHIHEKG----------------------------------------DTGNGCLS--AGGHYN---------PHKLS-------HGAPDDSNRHIGDLGNIESPAS--GDTLISVSDSLASLSGQY--------------------------------SIIGRSVVIHEKTDDLGRGTSD------QSKTTGNAGSRLACGTIGTV---------------------------------

>Caenorhabditis.elegans_34_CuZnSODSOD4

VLILALSVCIEAASEVIRARAIFKAGKTELIGTIDFDQS--GSF-LKLNGSVSGLA--AGKHGFHIHEKG----------------------------------------DTGNGCLS--AGGHYN---------PHKLS-------HGAPDDSNRHIGDLGNIESPAS--GDTLISVSDSLASLSGQY--------------------------------SIIGRSVVIHEKTDDLGRGTSD------QSKTTGNAGSRLACGTIGIVEERILETT-------------------------

>Ephydatia.muelleri_40_CuZnSOD

VL-SILLAVSQSVPETTRAVSVMS-GPTDVKGVIVFESL-GHDW-IRVTGSITGLQ--PGPHGFHVHKLG----------------------------------------DLTNGCVS--AGEHFN---------PFRQT-------HGGLKSKVRHAGDLGNIVANAE--GVAMVDLVARQMALSGPL--------------------------------SIIGRSIVIHADKDDLGLGHEP------DSPTTGHSGTRVGCGVIGRS---------------------------------

>Capitella.teleta_39_CuZnSOD

LI-------PEAAGEKITAECVIEAGTGPIQGTIRFEQDITGGA-TSITGSVSGFT--TGKHGFHVHAVG----------------------------------------NLGNRCSD--ATGHYN---------PFDKN-------HGAPDASERHVGDLGNIVENAN--GVADISMDDSLVSLVGEY--------------------------------TVIGRSIVVHAGEDDLGLGGDS------GSLTTGNAGARLGCCIIQEVGGQILHQG-------------------------

>Oscarella.carmela_43_CuZnSOD

LVFLFIGTSQYACCQDLVAVANLVNSDRTVTGTVLFHQLAEGGE-TLVRGVVKGLQ--AGRHGFHIHQQS----------------------------------------DNTLGCSS--YGGHYN---------PFHAH-------HSGRFDSERHVGDLGNIEFEET--GVASFTFRDSLAQLLGPQ--------------------------------SVLNRGVVIHARGDDLGKGGDE------GSRKTGNAGHRVACGILGVSGNESGLPE-------------------------

>Branchiostoma.floridae_42_CuZnSOD

-------------------------------------------------------------HGFHIHADG----------------------------------------DLSMGCDT--AGPIYD---------PFNKS-------HGGLNTTERKVGDLGNLDCDEL--GRVMMLLEVEDASLLGPY--------------------------------SILGRSMVIHANEDDFGHGGHE------MSPVDGNSGVRLACCVIGRTSDAELRWA-------------------------

>Mnemiopsis.leidyi_44_CuZnSOD

-----------------MAVLYKNSGQLDLVGIVQFTQAGPAAK-LMVNINVEYFA--DGLHGFHIHQMG----------------------------------------NVYGGCTQ--TLGHYN---------PLDVL-------HGGPDSAVRHTGDFGNIEIKNH---AFTGEHTDSQASLYGQY--------------------------------SIIGRGVVLHETQDDLGKGGDL------GSQKTGNAGSRLACGVIGISSEGYDNAS-------------------------

>Mnemiopsis.leidyi_50_CuZnSOD

VLLLTL---IQLSVSTKKASCIMKNGENTEVGKVTFSVHDNT---QTVEAHLAAIA--PGLHGFHVHTLG----------------------------------------VDGYDCGS--TGGHFN---------PYKKY-------HGVLDKDSRHLGDFGNVLAGDR--GEINYTVTSPQFELEGEN--------------------------------SIVGRAVVLHAGQDDLGQGGDD------GSLATGNAGSRVACCTLHLAHPY------------------------------

>Mnemiopsis.leidyi_51_CuZnSOD

ILFLTSLQLSAANHHIRRASCSMKNGKDKVIGKVTFSEHNNI---QTIHAHLDEIV--PGLHGLHIHTDG----------------------------------------VTDNDCAS--SGGHFN---------PDGNK-------HGELDADLRHVGDFGNVEAGER--GEINYTIHSPRFNLKGKN--------------------------------SVVGRAVVLHAGEDDLGLGGDE------GSRKTGNAGERVACCTLEL----------------------------------

>Mnemiopsis.leidyi_52_CuZnSOD

----------------------MKHNNGTDIGEVSFSSGSGNT--QEVSVFLSKIP--QGYYGLHVHTFP----------------------------------------VIEFDCSSSSTGDHFN---------PKGNR-------HGSPSANDRHVGDFGNIRAGER--GEINASIKSPLFRLEGED--------------------------------SIVGRAAVIHAHEDDQGKSGTA------KSTKTGNAGKPIACCTLQL----------------------------------

>Danio.rerio_45_CuZnSOD

LSCHVLCGSGSSNSDSLQAVCRMQPGMPRVYGHILFRQSGPKEK-LSVTFRLYGLP--ADPRAMHIHEYG----------------------------------------DLSRGCDS--TGGHYN---------PLNVN-------H------PQHPGDFGNFVPVN---KKIRQSL-ESPATLFGKL--------------------------------SIVGRSVVIHEGKDDLGRGGNV------GSLLNGNAGGRLACCVIGLRNPQN-----------------------------

>Homo.sapiens_46_CuZnSODSOD3

CSCLLLAAGRRDDDGALHAACQVQPAQPRVTGVVLFRQLAPRAK-LDAFFALEGFP--TESRAIHVHQFG----------------------------------------DLSQGCES--TGPHYN---------PLAVP-------H------PQHPGDFGNFAVRD---GSLWRYRAGLAASLAGPH--------------------------------SIVGRAVVVHAGEDDLGRGGNQ------ASVENGNAGRRLACCVVGVCGPGLWERQ-------------------------

>Xenopus.tropicalis_47_CuZnSOD

TVCELLSAGAEDNDGIAYATCSLSPSEVKVTGLVLFKQVFPSGT-LEAIFDLEGFP--TDARAIHIHTYG----------------------------------------DLTNGCDS--AGGHYN---------PMSVD-------H------PQHPGDFGNFRVRD---GKIQKFFANLDATLFGPF--------------------------------SVIGRSVVVHKQADDLGKGNNQ------ASLENGNAGKRLACCIIGSSSKNNWEKY-------------------------

>Xenopus.tropicalis_48_CuZnSOD

TVCELLSAGAEDNDGIAYATCSLSPSEVKVTGLVLFKQVFPSGT-LEAIFDLEGFP--TDARAIHIHTYG----------------------------------------DLTNGCDS--AGGHYN---------PMSVD-------H------PQHPGDFGNFRVRD---GKIQKFFANLDATLFGPF--------------------------------SVIGRSVVVHKQADDLGKGNNQ------ASLENGNAGKRLACCIIGSSSKNNWEKY-------------------------

>Danio.rerio_49_CuZnSOD

ISVILLALIVFEFNNTIYATCEVSPGQPKIFGQVLFRQVFPNGT-AEVKINLRGFP--ETVRAIHIHQYG----------------------------------------DLSQGCVT--AGPHYN---------PQDVP-------H------PNHPGDMGNFMPKQ---GLIRRFLKLPEVKLFGGQ--------------------------------SVLGRAVVVHEKEDDLGMGADE------ESKRSGNAGRRIAGCVIGITKPHLWQKT-------------------------

>Mnemiopsis.leidyi_54_CuZnSOD

ITMVLGAAALQVSWAEYEAECTMKHNTEIEIGKVVFKSGEGF---QSIQANLEVIT--EGLHGFHVHEKP----------------------------------------VMGNDCGAASTGGHYN---------PDGSN-------HSNLPSGERHAGDFGNVMAGQR--GEINLDMKITVGTMLGEP--------------------------------SMRCAPIKVKVYPDGRD-----------GMKRSGKRKSEIRCQPLSLER--------------------------------

>Mnemiopsis.leidyi_59_CuZnSOD

TV-------LVAETSATRASCYLRHNDGRIIGTAKFYSTGRY---QNVSVDLSGLT--DGLHGFHIHEQG----------------------------------------VTGCDCLT--TGGHYN---------PDGVN-------HGGAAASSRHVGDFGNVESRMSKASEVNTEISKTHITLKNAK--------------------------------SIIGATVILQPIEEENCQGTIR------DPSKSPNAEQRKKTDVIKIDESKNISET-------------------------

>Pseudanabaena.ABRG5-3_CuZnSOD

VLLSCATVA------AQRASSRIF-VKGDLVGTATFIQTPLG---VKVDLKVQNLA--QGEHMVHLHEQG----------------------------------------KDAPDFKT--SGNHFD---------PEEKD-------HMQMHHKHKPAGDLPNIIVKQD--GNGNLTALLPKLTLSGKN--------------------------------SLLGTSILIHAGANGK------------STIPNVDFKTRIACGVIKSE---------------------------------

>Pseudanabaena.SR411_CuZnSOD

VLLPLITMA------TKSASSQIF-AKGELVGTATFTQSTSG---VKVTLQVQKLA--QGEHMVHLHENG----------------------------------------KEAPDFKS--AGNHFD---------PKRSP-------HDHMHHKHQPAGDLPNIMVQQD--GTGTLTATLSELTLTGHN--------------------------------SLLGTAIVIHAGANGK------------STIPNVDYKTRIACGVVKSQ---------------------------------

>Pseudanabaena.PCC7429_CuZnSOD

FGLACMTITAQIIRNPQSAIASIF-IKGEQVGTATFTQTPLG---LQVIVNVENLT--QGEHMIHIHENG----------------------------------------KDAPDFKT--SGNHFN---------PKNKD-------HGAHENGHNPAGDLPNIVVKKD--GKGKLNALLPRLSLNKPN--------------------------------SLLGTSILIHAGANGN------------STIPNVDYKTRIACGVIKKF---------------------------------

>Pseudanabaena.PCC7367_CuZnSOD

IAIGSSTIATESAHSDKGAMAMIK-IEGQLVGTASFRDTEAG---LEVIVEAQNLA--PGEHAIHIHEVG----------------------------------------KEAPDFKS--AGGHFN---------PSNPS-------HGAMHHNHGAAGDLPNLNISRD--GTGVLTAVLPDLALDSSN--------------------------------SILGTAVLIHAGAAGV------------TTIPGVDADTRVACGVVSAAE--------------------------------

>Gloeobacter.kilaueensis.JS1.SODC_CuZnSOD

LL-LIPVFAASAQTGTRQATAALV-SNGQTVGTAALSQSSAGV--LSVKLDVKGLP--PGEHGIHFHAVG----------------------------------------LEGPGFTS--AGGHFN---------PATRQ-------HGLENPKGAHAGDLPNLSVAAD--GTASYSATDVRASLPNAN--------------------------------NLLGTALIIHARPDDL------------KSDPVGNSGARIACGVLKPAK--------------------------------

>Gloeobacter.kilaueensis.JS1.GKIL_3379_CuZnSOD

PLIALLLAAPVLAGNPPQATAQIK-AKGEPVGTASFVQQPKG---VLVTVEVKGLE--PGKHPMHIHAVG----------------------------------------KDAPDFRS--AGPHLG---------DHKMA--------GMKGSMATMAGDLPELLVGAD--GTGKAEVLNPEISL-GSK--------------------------------SLLGTALLIHDTK----------------------PSKRIACGVISTNPNP------------------------------

>Gloeobacter.violaceus.PCC7421.glr1981_CuZnSOD

LAVGWLLAAPALAGEQPAAVSAIK-LNGQVVGTATFRQQPEG---VLVNIQVQGLQ--PGKHAMHIHAVG----------------------------------------KEPPAFKS--AGPHLG---------DAKMA------PHGAKGATAAMAGDLPDLVVGSD--GSGKAEILNAEVRLPGRN--------------------------------FLLGTALLIHDAD----------------------PNQRIACGVISKAPNSSPEAK-------------------------

>Gloeobacter.violaceus.PCC7421.glr2170_CuZnSOD

LLCLLLMAVPADADRWLQAKIELK-ADGLPVGNASLTQLSDG---VRVSVQVQGLL--PGKYPIHFHSKG----------------------------------------KVAPDFRS--SRGVFD---------THSLG-------HKPDGQPVPPAGLLPALIVGAA--GTGELNALNTDVTLAHKH--------------------------------SLLGSALVIHAAH----------------------SRQIIACGAVTRTPVSD-----------------------------

>Capitella.teleta_56_CuZnSOD

-----------------EVVMQVSPGNGPTVGKVVIHYADDAQG-VRFQPALHDLP--PGAHGFHVHENP----------------------------------------SPGEDALA--AGSHYD---------PEKTG-------HHEGPKGHGHLGDLPALTVDDY--GNAVTPVEAPRLSLP-----------------------------------ELKGHSLVIHAGSDNYS-----------DHPPLGGGGARIACGII------------------------------------

>Drosophila.melanogaster_55a_CuZnSOD

KLILALVIGYGGLVPQWQAGAKLMDGEAGVAGMISFVQLPNSD--IRVTINVTGLP--PGKHALHIHTFG----------------------------------------DLSDGCKS--TGGQF----------PNNF---------------------LGNVDTKDD--GSISAVFQSIYLQLFGIN--------------------------------GIVGRSIVIHSKAIDLNTALNA------EVFSSNSLGPAIACGVISIMSTAASSSG-------------------------

>Drosophila.melanogaster_55b_CuZnSOD

KLILALVIGGGQLVPQWQAGAKLMDGEAGVAGMISFVQLPNSD--IRVTINVTGLP--PGKHALHIHTFG----------------------------------------DLSDGCKS--TGGQF----------PNNF---------------------LGNVDTKDD--GSISAVFQSIYLQLFGIN--------------------------------GIVGRSIVIHSKAIDLNTALNA------EVFSSNSLGPAIACGVISIMSTAASSSG-------------------------

>Mnemiopsis.leidyi_53_CuZnSOD

---------------SIELNCLLRFSEPNLEYNANLAQWSEQNP-LRVKNALRGNP---------------------------------------------------------GNCDR--TGAIFK--------------------------KRGRVLGDLGNVRAGER--GEVNHTLNKPLFHLQGKF--------------------------------SIVGRAVLLQVGS----------------LTSVGSPGDAIACCTLQLEQ--------------------------------

>Strongylocentrotus.purpuratus_57_CuZnSOD

MT-----IDEEGEDQSTRSPAVITPNDHRFRGTVNLRQNRSNGS-SDVTIRLFGLS--PSGHGVYIREFG----------------------------------------DLGDGCQR--LGPIFA--------------------TNRNPDQQVGSTGLLAVVSPDES--GFVQYRTTEVGFDLAGRN--------------------------------SIFGRSIVIEQNQDTY--------------------NAPLGCCVIGVTRDAE-----------------------------

>Strongylocentrotus.purpuratus_58_CuZnSOD

MT-----IDEEGEDQSTKSPAVITPNEHRFKGTVNLRQNRSSGS-SDVTIRLFGLS--PSGHGVYIREFG----------------------------------------DLGDGCQR--LGPIFD--------------------TNSNPDQQIGSTGLLAVVSPDES--GFVQSRTTEVGFDLVGRN--------------------------------SIYGRSIVIEQNQDTY--------------------NAPLGCCVIGVTRDAE-----------------------------

>Acanthaster.planci_60_CuZnSOD

ST-ETGKALASGGSEHPQDVCKLKPGDNHVVGLLVFKQKKSGGP-MEIKASLYGLD--DGDHTVDVHEFG----------------------------------------DLSNGCKS--TGTRYS---------------------------ATYSLGVMGSALRDET--SEVRAAWSTTSEGLVGEH--------------------------------SILGRAVVVHAVPHDSGEREE---------------SPKLACCNVARSSEV------------------------------

>Mnemiopsis.leidyi_115_CuZnSOD

LL---GKGNSVQTASTVESILRFSVGQNKAKGIVRLLEDENG---LNIEGTVSGLE--KGSYSVSINEYG----------------------------------------DISNACLS--TGN------------PVSKA-------------EETPCGILGQVESDGS---------IDTKFSLSGRL--------------------------------SIMGRSLVLQNET------------------------KKIACGIVGRSPGVSENNKK------------------------

>Caenorhabditis.elegans_113_CuZnSOD

IEETTTTTTEASVPPATTVIATFDPGTGAHIGRFTFSQLTSTA--LRIHGEVYTLP--VGRHAVVLHQFG----------------------------------------DSSEGCSR--VGAPFS--------------------------KSLSPLGDITET-------GKFDRIVEWPVI--------------------------------------DVVGRAVVIYSFSTAEWSLRAG--------------EKPLACGTIGIAKVR------------------------------

>Caenorhabditis.elegans_114_CuZnSOD

IEETTTTTTEASVPPATTVIATFDPGTGAHIGRFTFSQLTSTA--LRIHGEVYTLP--VGRHAVVLHQFG----------------------------------------DSSEGCSR--VGAPFS--------------------------KSLSPLGDITET-------GKFDRIVEWPVI--------------------------------------DVVGRAVVIYSFSTAEWSLRAG--------------EKPLACGTIGIAKVR------------------------------

>Caenorhabditis.elegans_115_CuZnSOD

IEETTTTTTEASVPPATTVIATFDPGTGAHIGRFTFSQLTSTA--LRIHGEVYTLP--VGRHAVVLHQFG----------------------------------------DSSEGCSR--VGAPFS--------------------------KSLSPLGDITET-------GKFDRIVEWPVI--------------------------------------DVVGRAVVIYSFSTAEWSLRAG--------------EKPLACGTIGIAKVR------------------------------

>Ciona.intestinalis_112_CuZnSOD

VA-------------------------SPVSGHICFTEKGGQ---VEAHVQVSGLD---RNYRVHVHEHA----------------------------------------PASDGCTT--VGGHYN----WAD--PQNID-----------------EGDMLTLKSNTE--SVLHHIHPNSALSLNGER--------------------------------SIIGRSVAIHV-------------------------GHKVCCPIVACSKYRHMRNLES-----------------------

>Acanthaster.planci_1_MnSOD

--------------------SRAVRRLLASPGTLSTVNSRLEHTLPPLPYDYGALSPVINADIMELHHKKHHATYVNNLNLAEKQLQEAQEAGDIGKMI------------ALQPAVKFNGGGHINHSIFWTVMSP-----------NGGGE----PSGELLSAINRFGSFDNMKQKLSAASIGVQGSGWGWLGYNKTTRTLAIATCANQD-PLEPTTGLVPLFGIDVWEHAYYLQYKNVRPDYVKAIFNVANWANIEERFQKAMA------------------------------------

>Branchiostoma.floridae_2_MnSOD

--------------------ACVTQRVASHPGLLAAAGTRLKHTLPDLAYDYGALEPTISAEIMQLHHSKHHATYVNNLNVAEEKLAEAQAKGDVTTEI------------ALGPALKFNGGGHLNHSIFWTNLSP-----------NGGGE----PQGEVLEAINRFGSFENLKKKMSAASVAVQGSGWGWLGYDKENNRLSIAACANQD-PLQATTGLIPLLGIDVWEHAYYLQYKNVRPDYVNAIWNVVSWENINERFLAAKK------------------------------------

>Danio.rerio_3_MnSOD

--------------------CRVVRCAATFNPLLGAVTSRQKHALPDLTYDYGALEPHICAEIMQLHHSKHHATYVNNLNVTEEKYQEALAKGDVTTQV------------SLQPALKFNGGGHINHTIFWTNLSP-----------NGGGE----PQGELLEAIKRFGSFQKMKEKISAATVAVQGSGWGWLGFEKESGRLRIAACANQD-PLQGTTGLIPLLGIDVWEHAYYLQYKNVRPDYVKAIWNVVNWENVSERFQAAKK------------------------------------

>Homo.sapiens_4_MnSOD

---------------------LSRATSRQLAPVLGYLGSRQKHSLPDLPYDYGALEPHINAQIMQLHHSKHHAAYVNNLNVTEEKYQEALAK------------------------------------------------------------------GELLEAIKRFGSFDKFKEKLTAASVGVQGSGWGWLGFNKERGHLQIAACPNQD-PLQGTTGLIPLLGIDVWEHAYYLQYKNVRPDYLKAIWNVINWENVTERYMACKK------------------------------------

>Homo.sapiens_5_MnSOD

---------------------LSRATSRQLAPVLGYLGSRQKHSLPDLPYDYGALEPHINAQIMQLHHSKHHAAYVNNLNVTEEKYQEALAK------------------------------------------------------------------GELLEAIKRFGSFDKFKEKLTAASVGVQGSGWGWLGFNKERGHLQIAACPNQD-PLQGTTGLIPLLGIDVWEHAYYLQYKNVRPDYLKAIWNVINWENVTERYMACKK------------------------------------

>Homo.sapiens_6_MnSOD

---------------------------------------------------------------MQLHHSKHHAAYVNNLNVTEEKYQEALAK------------------------------------------------------------------GELLEAIKRFGSFDKFKEKLTAASVGVQGSG-----------------------------------------------------------------------------------------------------------------

>Homo.sapiens_7_MnSOD

---------------------LSRATSRQLAPVLGYLGSRQKHSLPDLPYDYGALEPHINAQIMQLHHSKHHAAYVNNLNVTEEKYQEALAKGDVTAQI------------ALQPALKFNGGGHINHSIFWTNLSP-----------NGGGE----PKGELLEAIKRFGSFDKFKEKLTAASVGVQGSGWGWLGFNKERGHLQIAACPNQD-PLQGTTGLIPLLGIDVWEHAYYLQYKNVRPDYLKAIWNVINWENVTERYMACKK------------------------------------

>Homo.sapiens_8_MnSOD

---------------------LSRATSRQLAPVLGYLGSRQKHSLPDLPYDYGALEPHINAQIMQLHHSKHHAAYVNNLNVTEEKYQEALAKGDVTAQI------------ALQPALKFNGGGHINHSIFWTNLSP-----------NGGGE----PKGELLEAIKRFGSFDKFKEKLTAASVGVQGSGWGWLGFNKERGHLQIAACPNQD-PLQGTTGLIPLLGIDVWEHAYYLQYKNVRPDYLKAIWNVINWENVTERYMACKK------------------------------------

>Homo.sapiens_9_MnSOD

---------------------------------------------------------------MQLHHSKHHAAYVNNLNVTEEKYQEALAKGDVTAQI------------ALQPALKFNGGGHINHSIFWTNLSP-----------NGGGE----PKGELLEAIKRFGSFDKFKEKLTAASVGVQGSGWGWLGFNKERG------------------------------------------------------------------------------------------------------

>Homo.sapiens_10_MnSOD

---------------------------------------------------------------MQLHHSKHHAAYVNNLNVTEEKYQEALAKGDVTAQI------------ALQPALKFNGGGHINHSIFWTNLSP-----------NGGGE----PKGELLEAIKRFGSFDKFKEKLTAASVGVQGSGWGWLGFNKERGHLQIAACPNQD-PLQGTTGLIPLLGIDVWEHAYYLQYKNVRPDYLKAIWNVINWENVTERYMACKK------------------------------------

>Homo.sapiens_11_MnSOD

--------------------ARRESTSRQLAPVLGYLGSRQKHSLPDLPYDYGALEPHINAQIMQLHHSKHHAAYVNNLNVTEEKYQEALAKGDVTAQI------------ALQPALKFNGGGHINHSIFWTNLSP-----------NGGGE----PKGELLEAIKRFGSFDKFKEKLTAASVGVQGSGWGWLGFNKERGHLQIAACPNQ--------------------------------------------------------------------------------------------

>Homo.sapiens_12_MnSOD

--------------------PRVALTSRQLAPVLGYLGSRQKHSLPDLPYDYGALEPHINAQIMQLHHSKHHAAYVNNLNVTEEKYQEALAKGDVTAQI------------ALQPALKFNGGGHINHSIFWTNLSP-----------NGGGE----PKGELLEAIKRFGSFDKFKEKLTAASVGVQGSGWGWLGFNKERGHLQ---------------------------------------------------------------------------------------------------

>Homo.sapiens_14_MnSOD

---------------------LSRATSRQLAPVLGYLGSRQKHSLPDLPYDYGALEPHINAQIMQLHHSKHHAAYVNNLNVTEEKYQEALAKGDVTAQI------------ALQPALKFNGGGHINHSIFWTNLSP-----------NGGGE----PK------------------------------------------------------------GLIPLLGIDVWEHAYYLQYKNVRPDYLKAIWNVINWENVTERYMACKK------------------------------------

>Xenopus.tropicalis_13_MnSOD

--------------------LCRLRGRLRCAPALTYFTSREKHTLPDLPYDYGALQPHISAEIMQLHHSKHHATYVNNLNITEEKYAEALAKGDVTTQV------------SLQAALKFNGGGHINHTIFWTNLSP-----------NGGGE----PQGELLDAIKRFGSFEKFKEKLSTVSVGVQGSGWGWLGYNKESNRLQLAACANQD-PLQGTTGLIPLLGIDVWEHAYYLQYKNVRPDYMKAIWNVINWENVAERYRASKK------------------------------------

>Nematostella.vectensis_15_MnSOD

--------------------LARVARLSSRKAVLPVALVRAKHTLPDLPYDYDALEPTINTEIMRLHHSKHHATYVNNLNIAEEKCLEAQAKGDVATAI------------ALQPAVKFNGGGHLNHSIFWTNLSP-----------NGGGE----PTGELMEAIKRFGSFENFKERFNAATIAVQGSGWGWLGYDKVNKRLAIATCFNQD-PLQPTTGLVPLLGIDVWEHAYYLQYKNVRPDYVKAIYDVINWTNVAERLQAASS------------------------------------

>Capitella.teleta_16_MnSOD

--------------------LSAAVNSLKHAVPRCAVSSRLKHTLPDLPYDYNALEPTISADIMQLHHSKHHATYVNNLNVAEEKLAEARATGDVSTEI------------ALGSALIFNGGGHINHSIFWQNLSP-----------NGGGE----PTGELMSAIQRFGSFENMKKTLSASTVAVQGSGWGWLGYNKAAGKLQIATCANQD-PLEATKGLVPMFGIDVWEHAYYLQYKNVRPDYVNAIWNVANWADITERFNKARQ------------------------------------

>Oscarella.carmela_17_MnSOD

--------------------SQALLSRLGVRSLGGFLCCRSKHTLPDLPYDYGALEPAISAEIMQLHHSKHHATYVNNLNGAEEKLAEALATGDTASQI------------ALQPALKFNGGGHINHSIFWTNMCP-----------GGSGE----PQGDLMNAIKRFGSSEKLKTSLSAASVAVQGSGWGWLGYNKIRKRLEVATCANQD-PLEATTGLVPLLGIDVWEHAYYLQYKNVRPDYVKAIWEVVNWPNVAERFAAAQG------------------------------------

>Sycon.ciliatum_18_MnSOD

--------------------AACSAARQCAARSLASACAAQKHTLPDLPYDYGALAPHISPEIMQLHHSKHHQTYVNNLNVAEEKLQECAAKGDASGVI------------ALGGALKFNGGGHINHSIFWQNLSP-----------NGGGE----PTGELNNAIVTFGSFDAFKQKMTTAAVGVQGSGWAWLGLNKETMQLSVSACPNQD-PLQSTTGLVPLLGIDVWEHAYYLQYKNVRPDYLKAIWNVINWEDVASRYTGAQ-------------------------------------

>Strongylocentrotus.purpuratus_23_MnSOD

-----------------------QPLQLLRSQARVVSGVSSLHTLPELPYDYNALSPVISTEIMELHHKKHHNTYVTNLNVAEEALEHAVEAGDISTII------------SLQAALRFNGGGHINHTIFWQNLSP-----------DGGGE----PTGELLNVIKKFGSYDDMRSKLSAATVAIQGSGWGWLGYNKGTRSLQIATCPNQD-PLKATTGLIPLFGIDVWEHAYYLQYKNVRPDYVKAIFNIANWSDVEKRLTDAMT------------------------------------

>Amphimedon.queenslandica_20_MnSOD

L-------------------SATFFGGKNSLSSLSSISRRHKHVLPELPYGYKALEPVISGDIMELHHTKHHATYVNNLNATEGKMKECLEAGDVSGAV------------ALEGAYRFNGGGHINHSIFWNNLSP-----------NGGGT----PQGKLMEAIERFGSFDEFKSQLTARTVAIQGSGWGWLGFNQVTGRLQIATCPNQD-PLQATTGLVPLLGIDVWEHAYYLQYRNVRPDYVKAIWDVINWDDVSKRLP----------------------------------------

>Xestospongia.bergquistia_19_MnSOD

--------------------SILSHPFKGTGVTALTSLSRQNHSLPELPYRYNALEPVISGEIMELHHKKHHATYVNNLNTAEEQLKQCVDEGNVGGVI------------ALQPAIKFNGGGHINHSIFWNNLSP-----------NGGGE----PQGDFRAAIERFSSFDNFKSQLSAKTIAIQGSGWGWLGYNKESGRLQIATCANQD-PLQGTTGLIPLLGIDVWEHAYYLQYKNVRPDYVKAIWDVINWQDVTERYSNAMT------------------------------------

>Tethya.wilhelma_24_MnSOD

--------------------SAALLRHSRQATCVAATQTRNKFTLPELPYKYNALEPVISGEIMELHHSKHHATYVNNLNAAEEKLKQAIEDGNIKGIL------------DTQGAIKFNGGGHLNHSIFWTHLSP-----------NGGGK----PTGDLAAAIDRFGSFDNLMDQMTTKTVAIQGSGWGWLGYSKSNGRLVISTTANQD-PLQATTGLIPLLGIDVWEHAYYLQYKNVRPEYVKAIWKVMDWKDIESKFSAAKS------------------------------------

>Ciona.intestinalis_21_MnSOD

-----------------------LLSSRCTSKVVPVWASRGKHTLPDLPYDYSALEPHISAEIMETHYAKHHATYVNNLNIAEEKLHEAEAKNDISSII------------SLGPALKFNGGGHINHSIFWETLSP-----------NGGSE----PCGELKTAIDRFGSFENLKAKLTAASVGVQGSGWSWLGLDKEKGQLQVVACPNQD-PLHATTGLVPLFGIDVWEHAYYLQYKNVRPDYIKAIFNVVNWENVGKRFTDA--------------------------------------

>Mnemiopsis.leidyi_22_MnSOD

L-------------------------QSRLLKLSRLAQARCKYTLPELPYNYEALEPVINREIMEIHHSKHHATYVNNLNATAEKVSAAVAEGNAGAVI------------GLHGALKFNGGGHINHSIFWNNLSP-----------NGGGV----PSGELLSAIEAFGSFDQFKAKMSAATIAVQGSGWGWLGLNPDSKRLQIATCANQD-PLQATTGLAPLLGIDVWEHAYYLQYKNVRPEYVNSIWDIVNWEDVAARYAAASS------------------------------------

>Caenorhabditis.elegans_25_MnSOD

--------------------LQNTVSK-LVQPITGVAAVRSKHSLPDLPYDYADLEPVISHEIMQLHHQKHHATYVNNLNQIEEKLHEAVSKGNVKEAI------------ALQPALKFNGGGHINHSIFWTNLAK-----------D-GGE----PSAELLTAIKSFGSLDNLQKQLSASTVAVQGSGWGWLGYCPKGKILKVATCANQD-PLEATTGLVPLFGIDVWEHAYYLQYKNVRPDYVNAIWKIANWKNVSERFAKAQQ------------------------------------

>Caenorhabditis.elegans_26_MnSOD

--------------------LQNTVSK-LVQPITGVAAVRSKHSLPDLPYDYADLEPVISHEIMQLHHQKHHATYVNNLNQIEEKLHEAVSKGNVKEAI------------ALQPALKFNGGGHINHSIFWTNLAK-----------D-GGE----PSAELLTAIKSFGSLDNLQKQLSASTVAVQGSGWGWLGYCPKGKILKVATCANQD-PLEATTGLVPLFGIDVWEHAYYLQYKNVRPDYVNAIWKIANWKNVSERFAKAQQ------------------------------------

>Caenorhabditis.elegans_27_MnSOD

--------------------LQNTVSK-LVQPITGVAAVRSKHSLPDLPYDYADLEPVISHEIMQLHHQKHHATYVNNLNQIEEKLHEAVSKGNVKEAI------------ALQPALKFNGGGHINHSIFWTNLAK-----------D-GGE----PSAELLTAIKSFGSLDNLQKQLSASTVAVQGSGWGWLGYCPKGKILKVATCANQD-PLEATTGLVPLFGIDVWEHAYYLQYKNVRPDYVNAIWKIANWKNVSERFAKAQQ------------------------------------

>Caenorhabditis.elegans_28_MnSOD

--------------------LQSTASK-LVQPVAGVLAVRSKHTLPDLPFDYADLEPVISHEIMQLHHQKHHATYVNNLNQIEEKLHEAVSKGNLKEAI------------ALQPALKFNGGGHINHSIFWTNLAK-----------D-GGE----PSKELMDTIKRFGSLDNLQKRLSDITIAVQGSGWGWLGYCKKDKILKIATCANQD-PLE---GMVPLFGIDVWEHAYYLQYKNVRPDYVHAIWKIANWKNISERFANARQ------------------------------------

>Trichoplax.adhaerens_1_SODMn

--------------------LANVLGRNSCHSALLITQARRKHDLPPLPYAYNALEPTISAEIMELHHSKHHQTYVTNLNAAEEKLAEATSKNDISGVI------------TLQGALRFNGGGHINHSIFWKNLSN-----------DGGGL----PTGELGDAINAFGSFDNFKSKLSAATIAIQGSGWGWLGYCKESNSLKIATCANQD-PLQATTGYVPLLGIDVWEHAYYLQYKNVRPNYVNAIFDVINWNDVANNFRNAKA------------------------------------

>Monosiga.brevicollis_SODMn

--------------------------LARAMRSATTGARRMKHTLPDLAYDYAALEPVISAKIMELHHSKHHNTYVNNLNIAEEQYAEAVHTGDLTKAI------------GLQSAIKFNGGGHINHSIFWTNLAPKE---------QGGGE----QDGELKTAIEEFGSVETMQQKLNAMTAAVQGSGWGWLGYNKASKQLQLATCANQD-PLETTHGLVPLFGIDVWEHAYYLDYKNVRPDYLKAVWEIANWQNVEERYQAAKL------------------------------------

>Lingula.anatina_29_MnSOD

--------------------CAMLRFVPQQKAALGFTASRLAHSLPDLPYDFNALEPVIGAEIMEIHYKKHHATYVNNLNIAEEKMEKALAEGDTNAAI------------SLLPALNFNGGGHINHSIFWTNLSP-----------NGGGE----PTGDLKESINRFGGFESMKKELISKTVAIQGSGWGWLGYNPHTQRLRLAVRPNQD-SLFPTLGLIPLLGIDVWEHAYYLQYKNDRGAYVNAIWNIINWDNVADRYANARM------------------------------------

>Drosophila.melanogaster_30_MnSODa

F-------------------------VARKISQTASLAVRGKHTLPKLPYDYAALEPIICREIMELHHQKHHQTYVNNLNAAEEQLEEAKSKSDTTKLI------------QLAPALRFNGGGHINHTIFWQNLSP-----------N-KTQ----PSDDLKKAIESWKSLEEFKKELTTLTVAVQGSGWGWLGFNKKSGKLQLAALPNQD-PLEASTGLIPLFGIDVWEHAYYLQYKNVRPSYVEAIWDIANWDDISCRFQEAKK------------------------------------

>Drosophila.melanogaster_30_MnSODb

F-------------------------VARKISQTASLAVRGKHTLPKLPYDYAALEPIICREIMELHHQKHHQTYVNNLNAAEEQLEEAKSKSDTTKLI------------QLAPALRFNGGGHINHTIFWQNLSP-----------N-KTQ----PSDDLKKAIESWKSLEEFKKELTTLTVAVQGSGWGWLGFNKKSGKLQLAALPNQD-PLEASTGLIPLFGIDVWEHAYYLQYKNVRPSYVEAIWDIANWDDISCRFQEAKK------------------------------------

>Dictyostelium.discoideum_SODMn

--------------------PRSIKKVGESNGLRNFGSQSNSYTLPDLPYDYGALSPVISPEIMTLHHKKHHQTYVNNLNIALDKLSSASSAKDVAQMI------------ALQSAIKFNGGGHVNHSIFWTNLAPKNQ--------DGGVA----PSGPLADAINKYGSIEKLIEKMSAETTAIQGSGWGWLGYDKANDRLVIQTQQNQD-PL-SVSGYVPLLGIDVWEHAYYLDYKNVRADYVKNIWQIVNWKNVAERYNTAKK------------------------------------

>Arabidopsis.thaliana_SODMn

-------------------AASRLAGLKETSSRLLRIRGIQTFTLPDLPYDYGALEPAISGEIMQIHHQKHHQAYVTNYNNALEQLDQAVNKGDASTVV------------KLQSAIKFNGGGHVNHSIFWKNLAPSS---------EGGGE---PPKGSLGSAIDAFGSLEGLVKKMSAEGAAVQGSGWVWLGLDKELKKLVVDTTANQD-PLVTKGGLVPLVGIDVWEHAYYLQYKNVRPEYLKNVWKVINWKYASEVYEKENN------------------------------------

>P.nodorum_CuZnSOD_2

--------------------SAATAPAVSRAGLAGTTFVRGKATLPDLSYDYGALEPAISGKIMELHHKNHHNTYVTSFNNFSEQIAEAKQKQDIAAQI------------ALQPLINFHGGGHLNHTLFWENLAPTS---------QGGGE---PPTGALSKAINDYGSLDAFKEKFNTALAGIQGSGWAWLVQDTQTGSVQIRTYANQD-PV--VGQFRPILGVDAWEHAYYLQYQNRKAEYFKAIWDVINWKAAEKRFK----------------------------------------

>Podospora anserina_1_SODMn

--------------------------------------MAQEYTLPALPYAYNALEPHISAQIMELHHSKHHQAYVTNLNNALRLHVAAVGAGDIASQI------------EMQQVIKFNGGGHINHSLFWKNLAP-----------AESEETKPEAAKELVAAVEKWGSLDDFKNAFSSTLLGIQGSGWGWLVKDSANG-LRIVTTKDQD-PV--VGRDVPVFGVDMWEHAYYLQYLNGKAAYVENIWNVINWKTAEERFLGTSE------------------------------------

>Podospora.anserina_2_SODMn

-----------------------------------------------------------------------------NLNNALRLHVAAVGAGDIASQI------------EMQQVIKFNGGGHINHSLFWKNLAP-----------AESEETKPEAAKELVAAVEKWGSLDDFKNAFSSTLLGIQGSGWGWLVKDSANG-LRIVTTKDQD-PV--VGRDVPVFGVD---------------------------------------------------------------------------

>Capitella.teleta_31_MnSOD

----------------------------------------------------------ISKETLDYHYGKHHNTYVVKLNGLVE------GTELANKSLEEII-------KTSEGGIFNNAAQVWNHTFYWNCLSP-----------NGGGK----PSGELAAAIDKFGSFDEFVAKFSDMAVNNFGSSWTWLVKNTDGS-LEIVNTSNAATPL--TGDQKPLLTCDLWEHAYYIDYRNVRPDYLKGFWALANWEFVADNFAG---------------------------------------

>Gloeobacter.violaceus.PCC7421.sodB_MnSOD.1

----------------------------------------MAHTLPPLPYDENALAPYVSAQTLSFHYGKHHTGYLNNMNKAIA------GTELESLSLVDLIRTAAK--NAEQKTLFNNAAQVWNHTFYWNSMRP-----------GGGGE----PGGTLGEMIKDFGSYDEFKKQFVTAGTTQFGSGYAWLVKDGEK--LVVTKTPNAETPI--TDESKPLLNMDVWEHAYYLDYQNLRPDYENAFANLINWEFAEKNLERVFA------------------------------------

>Gloeobacter.kilaueensis.JS1.sodA_MnSOD

----------------------------------------MAFTLPPLPYDESALAPYISAQTLSFHYGKHHKGYVDTLNKLVA------GSEAENTPLEELIKSVHG--QPDKAAIFNNAAQIWNHTFYWNSLKP-----------GGGGE----PTGTIAELIKDFGSYDEFKKQFITAGTTQFGSGYAWLVKDKNSGKLSVIKTPNAETPL--TDPTKPVLTFDVWEHAYYLDYQNLRPKYEEAVVHLLNWEFAEKNLASA--------------------------------------

>Pseudanabaena.ABRG5-3.sodB_MnSOD

----------------------------------------MAFELPPLPYAQDALAASMSAETLSFHYGKHHKAYVDNLNNLIK------DTDLADKSLEEIIKISY---KEGKAGIFNNAAQVWNHTFYWNGIKP-----------AGGGA----PTGALLDAINAFGSLDNFKTEFKNAGATQFGSGWAWLVAEGGT--LKITKTPNAENPL-IHEGQVPLLTMDVWEHAYYLDFQNSRPNFMANFVKLINWDFVAANFAAV--------------------------------------

>Pseudanabaena.PCC7429_MnSOD

----------------------------------------MAFELPSLPYAQDALAASMSAETLSFHYGKHHKAYVDNLNNLIK------DTDLADKPLEEIIKISY---KEGKAGIFNNAAQVWNHTFYWNGIKP-----------AGGGA----PTGALLDAINAFGSLDNFKTEFKNAGATQFGSGWAWLVAEGGT--LKITKTPNAENPL-IHEGQVPLLTMDVWEHAYYLDFQNSRPNFMANFVKLINWDFVAANFAAA--------------------------------------

>Pseudanabaena.SR411_MnSOD

----------------------------------------MAFELPSLPYAQDALAASMSAETLSFHYGKHHKAYVDNLNNLIK------DTDLADKSLEEIIKISY---KEGKAGIFNNAAQVWNHTFYWNGIKP-----------AGGGA----PTGALLDAINAFGSLDNFKTEFKNAGATQFGSGWAWLVAEGDK--LKITKTPNAENPL-VHEGQVPLLTMDVWEHAYYLDFQNSRPNFMANFVKLINWDFVAANFSAV--------------------------------------

>Nematostella.vectensis_32_MnSOD

----------------------------------------MAFELPKLPYAYDALEPHIDARTMEIHHSKHHNGYTTNLNNAIS------GTDLEGKSIENILINL----DLNNGAVRNNGGGYYNHNLFWTVMSP-----------NGGGN----PTGELAAAIDAFGSFEAFKTEFSKAAATRFGSGWAWLCVHKGGK-LEVCSSANQDNPL--MPGICPILALDVWEHAYYLNYQNRRPDYVEAFFNVIDWTEVTRRFATEK-------------------------------------

>Algoriphagus.machipongonensis_SODMn

----------------------------------------MAFELPSLPYAYDALEPNIDAKTMEIHHSKHHNGYVTNLNKAVE------GTDLEGKSLEELLKI-----AGSNTAVRNNGGGHFNHSLFWSILSP-----------DGGGE----PTGELADSISAFGSFAAFKETFNKAAATRFGSGWAWLCIDTKKE-LCVCSSPNQDNPL--MDVACPILGLDVWEHAYYLNYQNRRPDYINAFWNVIDWDAVSKRYAAAK-------------------------------------

>Chondrus.crispus_1_SODMn

----------------------------------------MSFALPNLPYQYDALEPYVDSTTMNIHHTKHHQTYVNNINKVIDG---PSGSALKGLSLPAIQANITSLPAEIQTPVINSGGGHFNHAMFWTLMGR-----------PGNTA----PVGSIKDKINAFGSFDEMKAKFNSAAAARFGSGWAWLSVGADGK-LFISSTKNQENPL--MAGVVPVLGLDVWEHAYYLKYQNRRPEYISAFWNVVNWDQVTKNYDSVCS------------------------------------

>Pyropia.yezoensis_SODMn

----------------------------------------MAFALPPLPYAYDALEPYIDSTTMNIHHTKHHNTYVTNVNNVLAG---ENGGALKGLSLSAIQKEVVTLPDSIKTAVRNSGGGHWNHSFFWSVMGK-----------TGAEA----PTGDLKSSIESFGSLDEMQKKFNTAAASRFGSGWAWLSVNADGQ-LFISSTPNQDNPL--MEGIVPILGLDVWEHAYYLKYQNRRPEYIASWWKTVDFDVIAKNYSAAKS------------------------------------

>Chondrus.crispus_2_SODMn

-------------------------SRASQDVASPAARSVSTFSLPDLKYGYASLEPHFDEKTMTVHHTRHHQTYVSNLNGVLTG---EHGSEIQDLSLSQIQRKVSSLPEAIRTTVMNHGGGHYNHTLFFSILAP-----------ETTSA----PVDELKTMVETFGSFDAMRKEFNAAAMKVFGSGWAWLGVGNDGK-LCISNTRNQENPL--MEGIVPIMGLDIWEHAMYLKYLNRRPEYIDAFWHIIDWEQVAANYSAARD------------------------------------

>Pseudanabaena.PCC7367_MnSOD

----------------------LSAAMGLTGSLAQADTPDDPFTLPPLPYDYNALEPHIDVRTMQIHHDRHHAGYVRNLNKAIAT-----YPDLAGMSAEDMLRDLTQVPEPIRTTVRNNAGGHVNHSMFWEIMSP-----------NGGGT----PTGAIAAMIAAFGNFENLQTAFNQAGASRFGSGWAWLVLDKQGN-LKVTSTANQDSPL--LEGLFPIMGNDVWEHAYYLNYQNRRGDYLKAWWNLVNWEEVNNRFLNAMV------------------------------------

>Gloeobacter.violaceus.PCC7421.sodB_MnSOD.2

--------------------LSTATVTVPSQPQASTTASGYPYKLAPLPYDYSALEPYIDAETMKLHHDKHHQAYVDNLNKALEK-----YPDLQKKSPEQLLRDLKQVPEDTRAAVRGNGGGHVNHTMFWEIMKP-----------KGGGE----PAGPIAAAIRTFGSFDAFKTQFNEAGTKRFGSGWVWLVSNPGGK-LEIVSTANQDSPI--ADGKYPIMGNDVWEHAYYLKYQNRRADYLSAWWNTLNWDEINRRFQKAST------------------------------------

>Amphimedon.queenslandica_33_MnSOD

---------------------SFTLTLSQSSIYDEMFYPVDEYSFPQLPYDYHELEPYIDQRTLTVHHKKHHQGYTVKMNQALKDWRKQEQSDLAKSSIIDILQNLEMVPDKWRTTLQNNAGGYVNHIYYWVTMCP-----------KPGEI-----SKALLKKIKAFAGMSEFKESFTTASLSLFGSGYVWLVTDDEGS-ISIISTKNQDCPI--SSNLYPLLVLDVWEHSYYLKHQNLRADYISDWWNVVCWQNVETFWINRQI------------------------------------

>Amphimedon.queenslandica_34_MnSOD

-----------------------------------MFHPADEYSFPQLPYDYHELGPYIDQRTLTVHHKKHHQGYTVKMNQALKDWRKQEQNDLARSSIINILQNLERVPDKWRTTLQNNAGGYVNHIYYWVTMCP-----------KPGDI-----PKALLKKIEAFAGMSEFKNSFTTASLSLFGSGYVWLVTDDEGS-ISIISTKNQ--VL--YCHAFILIMLYMYHYKRFL-----RELYFTRCTKFLIWR-----------------------------------------------

>Ephydatia.muelleri_35_MnSOD

-------------------------ACNSEQIYEDLVVYHDELKLPDLPYDYNALEPYIDEATMKVHHLGHHKTYGDNTNKALKEWRKGDTSPLARSSIVQILQKITEVPETWQVAIRNNGGGFVNHVIYWATMCP-----------GGGGS----PSGELLMQISRFGSYVAFQEHFTSAASKLFGSGYVWLCENAKGD-LVITSSKNQDSPL--SDGLYPLLVMDIWEHAYYLKHQNKRAGYIQSWWNIVCWDEVPKFWHQNRN------------------------------------

>Tethya.wilhelma_36_MnSOD

--------------------LSLHCNSGQRDAYEQLTHPATDYPLPDLPYSYDELEPLIDTHTLKVHHLGHHKAYTDKMNAALNQWKHEETSKLAQSSIITILQSILSVPEKWQTAIRNNGGGYVNHIFYWVTMCP-----------HGHDQ--DVPGGKLLEAIINFGGFEEFKSEFSQTAAKLFGSGYVWLCESQAGD-LSIVTTYNQDSPL--SDGLYPLLVLDVWEHAYYLKHQNKRTEYIRDWWSVVCWDHVADFWMTGAN------------------------------------

>Xestospongia.bergquistia_47_MnSOD

--------------------DSAAIFVDEATIYDRIFQKADKYELPNLPYQYNDLEPYLDERTLIVHHQGHHKAYTDKMNAALQEWREKYH-------WHDL--ELDQIPEKWQTALKNNGGGYVNHIFYWATMCP-----------------PKAPPPTLTAAIEEFGGISYFFDNFTSSAKSLFGSGYVWLVQDKEDN-LYIIQTKDQVNPV--SKGMNQITAIELGEHQNYHDVSNRDFQFLQE---LVPFQNVSKQWLPVAN------------------------------------

>Xestospongia.bergquistia_48_MnSOD

--------------------LLSRVQVDEATIYDRIFQKADKYELPNLPYQYNDLEPYLDERTLIVHHQGHHKAYTDKMNAALQEWREKMHDTLATLSIFDILKRLDQIPEKWQTALKNNGGGYVNHIFYWATMCP-----------------PKAPPPTLTAAIEEFGGISYFFDNFTSSAKSLFGSGYVWLVQDKEDN-LYIIQTKDQ--------------------------------------------------------------------------------------------

>Branchiostoma.floridae_37_MnSOD

--------------------NIIPKTVSVDTPYKGIQLFADEYPVPPLPYEYDALEPYIDERTARVHHLGHHAGYTKKMNAALKAWRDEGGVGLQSNSILHILENLNQVPDKHRTAIRNNGGGFVNHAIYWATMSP-----------RREGEPPRLPEATLATEIIAFQNFSNFQEQFTNTAKTLFGSGYVWLSRNPREQTLIIHTTANQDSPI--SDKLRPILVIDIWEHAYYLKHQNKRPDHIQDWWALVDWGQVKAWWAAYDA------------------------------------

>Lingula.anatina_38_MnSOD

--------------------VVSSHRFVASWPYDDSALSKETYLLPALPYGYGALEPHMDEAT--VHHQGHHAAYTAKMNAALKQWRR-----------------------------------------------------------------------------------------------------YVWLSRDQDGK-LIISTTANQDSPV--TERLHPILVIDIWEHAYYLKHQNKRANFVEAWWNLVDWRAVETWWKGRQT------------------------------------

>Lingula.anatina_39_MnSOD

--------------------IVTPHQTADARMYEDCVQQREDYPLPPLPYSYNSLEPHMDATTVQIHHLKHHAAYTKKMNAALKQWRDEDENTLATGSIVEILQHLYNVPEKYRTVLRNNGGGYVNHAIYFACMSS-----------RGAA-----ISQDLENDIIKFGSYNNFTKQFTTKAMGLFGSGYVWLSRGGNDK-LVISTTANQDSPV--TESFRPILVIDIWEHAYYLKYQYRRADFVAAWWKLVDWKAVVDWWRVNYR------------------------------------

>Lingula.anatina_40_MnSOD

--------------------VVTPHQTAAIGMYEESAVPKEAYPLPPLPYPYGGLEPHMDERTVQIHHQGHHAAYTTKMNAALKQWREEDGNTFAKSSILNILQNVSSVPEKYRTALRNNGGGYFNHAIYWACMSP-----------GGAA-----ITPDLKSDIIQFGTYDNFKDQFTTKAMALFGSGYVWLSRGENEQ-LVISTTANQDSPV--TEDLHPILVIDIWEHAYYLKHQNKRADFVADWWKLVNWNAVRDWWKRI--------------------------------------

>Lingula.anatina_41_MnSOD

--------------------IVTPHQTAAAAMYEDSVVLREDYLLPPLPYAYNSLEPHMDEKTVEIHHLEHHAAFTTKMNAVLKQWRDEDENTFAKSSILNILENLYDVPEKYRTALRNNGGGYFNHAIYWACMSP-----------RGAVT---CINPDLENDIKKFGSYNNFTNQFTTKAMALFGSGYVWLSRGKNEQ-LVISTTANEDSPV--TEDLRPILVIDIWEHAYYLKHQNKRADFVEAWWNLVNWDAVWDWWKEKHM------------------------------------

>Lingula.anatina_42_MnSOD

--------------------AVSSLRFVASWPYDDSGFSKDTYLLPALPYGYGALEPHMDEATVKVHHQGHHAAYTAKMNAALKQWRSEAGDKFANSSILNILQKLSHVPEKYRMTLRNNGGGYFNHAIYWACMSP-----------KGAR-----ISPGLEKDIKDFGTYENFKEQFTAKSLSLFGSGYVWLSRDQDGK-LIISTTANQDSPV--TERLHPILVIDIW-------------------------------------------------------------------------

>Nematostella.vectensis_43_MnSOD

---------------------------------------MEKYTLPELPYDYNELEPHIDEATLRVHHLGHHAAYTKKLNAALK-------------DIVEILRNNEQIPDKWRTDVINNGGGFVNHALYWATMSP-----------NPKSE-PRTPTGKIGDLIDKHGNFSMFKQWFDEQVNSMFGSGYTWLCQDVTSGFLTILNMGNQESPV--AYRLNPVLVIDLWEHAFYLKHQNKRPGYVHSWWHLVDWERVNEWWQNQNI------------------------------------

>Strongylocentrotus.purpuratus_45_MnSOD

--------------------WMCTTSYCVSYPFECIAEIRSEYILPDLPFGYDDLEPYIDSATLNVHHRGHHAGYTRKLNAALHQWGSE-EDSYAATSILDLLQHLDDIPPKYKQSIRNNGGGFVNHNIYWSTMAS-----------NKENV-TTMPSGDLLGEINEFGSFEEFKNQFTGRALTLFGSGYVWLSRRKPSSSLIISTTANQDTPI--SNGLQPILVLDVWEHAYYLKHQNKRANHIEDWWRVVDWSAVANWWSGDYE------------------------------------

>Capitella.teleta_46_MnSOD

--------------------VGCHSGLNGESPFTTIASKAELYSLPALTFGYADLEPFFDEATLHAHYDGHHETYRKKMNSALSEWRESDLDSFSTEPILEILKNLNKVPENFRNAIKNSGGGFVNHALYWACMSP-----------NPTRE-IRQPTGALLQDIENFGSFVKFSLKFTDRAVSLFGSGYVWLSRNPSSGALIITTTVNQDSPI--SDGLHPILVIDVWEHSYYLKHQFRRHVYVADWWKVVDWENVEEWWREVGS------------------------------------

>Oscarella.carmela_44_MnSOD

--------------------CLASYALDIPDGLLKIEKPEDAYKLPTLDFDYNALEPYIDEKTVRVHHLGHHAGYTHKMNAILRKWAEKKQQDLLSLPIVNILEQVNDIPLDIREDLQNHGGGFINHAWYWATIAP-----------VKAESRESEPVGSVAGAIEKYGDFKKFQQEFNHTAFTFFGSGYVWLCEVPPTGILQITTFPNQMSPL--GFGLKPILVLDVWEHAYYLKHQNKRAGYVHDWWEIIDWNKVELAWKNEQA------------------------------------

>Oscarella.carmela_49_MnSOD

--------------------PITFSAMAGSFPYLKIKDSAEGYLLPEVGYDYADLEPHIDEATMKLHHSRHHAGYTRKMNAALDAWRKSGVPELAAKPIENIIQHVDDVPKKWQRAIRNNGGGFINHAFYWQNMSP-----------PNDPNAQREPSGPLLEKIKSFGSFEALKQKITETAAGLFGSGWVWLVEVPGSGELQLLSSENQDNPM--SQGLKPILALDVWEHAYYLKHKNDRPSYIQSWWNIADWRKVGDAWNSQQ-------------------------------------

>Trichoplax.adhaerens_2_SODMn

---------------------------------------------LPLPYDKNELEPTLSKNTFDYHYDKHYLGYANKLSALVD------GTKYYNMLLADIIIESY---KNNDTPIYNNAAQVWNHIFYWKSIGKT----------------QKCPT-KLIELLNRFGSYDTFIEKFIQAGVTLFGSGWIWLVQDKESKKLSILQTKDADNPL--ILDKTPILTIDVWEHAYYIDYKNDRLKYLDQIINNIKWLFAVSNLN----------------------------------------

>Trichoplax.adhaerens_3_SODMn

---------------------------------------------------------------------------------------------------EDIVKKSY---ISKDTKIFNNAAQVWNHEFYWDSIRK-----------KTDNQNEAKPTYDLFNKINEFGSFEEFCKKFVEAGVALFGSGWIWLVQDPDTKKLEIMQTYNADSPM--LHNKIAILTIDVWEHAYYIDYKNDRLSYLENVIFHLNWDFAKNNMK----------------------------------------

>Sycon.ciliatum_50_MnSOD

--------------------LAAEALPAVSVRLLDIRLENGSYELPRLMYSYEALEPYMSERTVRVHHTGHHAAYTKKMNSILSRMPS--ADAVVASNPLQQLMNLDALPEDVRDAFRNQAGGFVNHAFTFFVMGP-----------LDQSVVDQGPSGKILELITEFGSTDAMKEQFSGAAAKLFGSGYVWLCLENVAGRLTITENANQDSPL--SGGFRPVLGIDLWEHAYYLQYQNKRVDYIATWWQLVSWERVGLAWIETEP------------------------------------

**Alignment used to construct the phylogenetic tree of CuZnSOD sequences only**

>C.crispus_CuZnSOD_1

NLPTLVCTVQDGYSVEGVVYFRRGVGTCYVRIMAAVAGLTNPQHGFHVHTYGDVSSDGSSTGGHFNVAGIEHGLPDDEIRHWGDLGNLINDGKGNAEYDRVDKVVRL---GALVGRGITIHEDQDAG-----SSEQPTGASGTRIGFCVIGYANP

>C.crispus_CuZnSOD_2

NSLPLVCNVVKGYNCTGYVKFRRGMPSCHARVRANLKGLSPGRHGFHIHTYGDIRLDGSSTGGHFNVKGLPHGYASSPARHMGDLNNVMARANGEAKYSREDNVIRL---GAIRGRGITIHQDRDMG-----PGSQPSGAAGDRVGTCVIGVVDA

>P.nodorum_CuZnSOD_1

-MVKAVAVLRGDSNVKGTVTFEQENESSPTKISWDITGNDNAERGMHVHAFGDNTNGCTSAGPHFNPHNKTHGAPEDEERHVGDLGNFKTDGQGNAQGSVSDKLIKLIGSESVIGRTIVVHGGTDDLGRGGHEESKKTGNAGPRPACGVIGISN-

>Podospora.anserina_1_SODCuZn

-MVKAVAVVRGDSKVSGSVVFEQETENGPTTITWDITGHDNAKRGMHIHTFGDNTNGCTSAGPHFNPHGKTHGNRTDENRHVGDLGNIETDAQGNSKGTVTDNLIKLIGPESVIGRTVVVHAGTDDLGKGDTEESLKTGNAGARPACGVIGISA-

>Podospora.anserina_2_SODCuZn

-MVKAVAVVRGDSKVSGSVVFEQETENGPTTITWDITGHDNAKRGMHIHTFGDNTNGCTSAGPHFNPHGKTHGNRTDENRHVGDLGNIETDAQGNSKGTVTDNLIKLIGPESVIGRTVVVHAGTDDLGKGDTEESLKTGNAGARPACGVIGISA-

>Acanthaster.planci_5_CuZnSOD

LSVRLVCNVLAVQQIRGQIQLTQYQPGGPVKVTGDVTGLAPGKHGFHVHEFGDYTGGCTSTGGHFNPFKKNHGAPTDEERHIGDLGNVEAGSDGTVSVNITDKMISLIGPNSIIGRAVVVHADVDDLGKGGHELSLTTGNAGGRLSCGVIGISK-

>Branchiostoma.floridae_6_CuZnSOD

-----------------------QSPGGPVRVTGEVQGLTEGPHGFHVHEFGDYTNGCTSMGAHYNPIGTNHGGPNDAVRHVGDLGNIVANVAGVAQVDITDNQLSLYGADSIIGRGVVVHADEDDLGKGGHELSDTTGNSGGRLACGIIGITK-

>Capitella.teleta_7_CuZnSOD

MVLKAICILKGSTPVEGTINFTQE-GDGPVTLEGQIAGLAPGKHGFHVHEFGDNTNGCVSAGSHFNPFGKTHGGPDSEVRHVGDLGNAVAGDDGIAKINITDDQVTLTGPHSVIGRTMVVHADPDDLGLGGHELSPTTGNAGGRLACGVIGITK-

>Sycon.ciliatum_8_CuZnSOD

MSVNAACVLAGDSAVKGVITFTQA--EGATTVTGEVTGLAPGNHGFHVHVYGDNTNGCTSAGPHFNPSNKTHGAPCDENRHAGDLGNVVAGDDGVAKVNITDSQIPLSGPNSIIGRTVVVHADPDDLGKGGHELSLSTGNAGARVACGVIGIAK-

>Arabidopsis.thaliana_1_SODCuZn

-MAKGVAVLNSSEGVTGTIFFTQE-GDGVTTVSGTVSGLKPGLHGFHVHALGDTTNGCMSTGPHFNPDGKTHGAPEDANRHAGDLGNITVGDDGTATFTITDCQIPLTGPNSIVGRAVVVHADPDDLGKGGHELSLATGNAGGRVACGIIGLQG-

>Danio.rerio_9_CuZnSOD

MVNKAVCVLKGTGEVTGTVYFNQEGEKKPVKVTGEITGLTPGKHGFHVHAFGDNTNGCISAGPHFNPHDKTHGGPTDSVRHVGDLGNVTADASGVAKIEIEDAMLTLSGQHSIIGRTMVIHEKEDDLGKGGNEESLKTGNAGGRLACGVIGITQ-

>Homo.sapiens_10_CuZnSODSOD1

MA-------------------TKAESNGPVKVWGSIKGLTEGLHGFHVHEFGDNTAGCTSAGPHFNPLSRKHGGPKDEERHVGDLGNVTADKDGVADVSIEDSVISLSGDHCIIGRTLVVHEKADDLGKGGNEESTKTGNAGSRLACGVIGIAQ-

>Homo.sapiens_11_CuZnSODSOD1

MATKAVCVLKGDGPVQGIINFEQKESNGPVKVWGSIKGLTEGLHGFHVHEFGDNTAGCTSAGPHFNPLSRKHGGPKDEERHVGDLGNVTADKDGVADVSIEDSVISLSGDHCIIGRTLVVHEKADDLGKGGNEESTKTGNAGSRLACGVIGIAQ-

>Lingula.anatina_12_CuZnSOD

MALKAVCVLKGASDVIGTVYFEQTSADGPCKVTGEVTGLKEGLHGFHVHQFGDNTNGCTSAGPHFNPSGKTHGGPDDENRHFGDLGNIKAGSDGKATVAITDKLVTLTGPNSVIGRTIVVHEGQDDLGKGGNEESLKTGNAGGRLACGVIGITH-

>Lingula.anatina_13_CuZnSOD

MRIAGVCV------------------------------------------------------------------------HFGDLGNIKAGSDGKATVAITDKLVTLTGPNSVIGRTIVVHEGQDDLGKGGNEESLKTGNAGGRLACGVIGITH-

>Branchiostoma.floridae_17_CuZnSOD

MSLKAVCVLVGE-TVKGTVTFTQASSDSPVEVTGTISNLTPGKHGFHIHEFGDTTNGCTSAGSHFNPAKKNHGGPQDAERHVGDLGNVEVGDDGVATINITDSQLQLTGPNSIVGRAVVVHAGEDDLGKGGFEDSLTTGHAGGRLACGVIGITKQ

>Ephydatia.muelleri_18_CuZnSOD

-MSQAVCVLEGQ-TVGGTIFFTEN-SDGSTHVTGMVTGLTPGDHGFHVHEYGDYSDGCVSAGEHFNPYKKQHGGPNDKERHAGDLGNITANESGEALVDIVDKQIPLTGPNSIVGRSIVVHADKDDYGRGGFDDSKTTGHSGARLACGVIGIAAN

>Ephydatia.muelleri_19_CuZnSOD

-MAQAVCVLEGQ-TVRGTISFTES-GDGATHVTGTVTGLTPGDHGFHVHEYGDYSGGCVSAGPHFNPYKKQHGGPNDEERHAGDLGNITANESGEALVDIVDKQIPLTGPNSIVGRSIVVHADKDDYGRGGFDDSKTTGHAGARLACGVIGIAKP

>Nematostella.vectensis_15_CuZnSOD

MPIQAVCCMSGTEGVKGTIKFVQEAEGKPCKITGTIEGLKAGNHGFHIHVYGDNTNGCVSAGPHFNPFKKEHGGPSDENRHVGDLGNVVAGDDGKACIDMTDALVTLVGEHSVVGRSVVVHADEDDLGRGGHEDSKTTGHAGGRLACGVIGITQA

>Trichoplax.adhaerens_SODCuZn

MALKAVCCLQGP-VVSGTIFFQQESGTGPIRISGEVKGLAPGKHGFHVHEFGDNTQGCTSAGGHYNPHKKVHGAPGDEIRHVGDLGNIEANEQGVASINMTDRMVTLTGPYSCIGRTIVVHEGVDDLGKGGHELSLTTGNAGARVACGVIGITKC

>Nematostella.vectensis_14_CuZnSOD

MVIRGVCCLVGDNEVKGVIHFTQQAPDGPCTLRGRITGLTEGKHGFHIHEFGDNTNGCTSAGAHYNPHGKMHGAPEDKDRHLGDLGNIEADANGIADVSITDCLVSLTGQCSIIGRSLVVHEGMDDLGAGGHELSLTTGNAGGRVACGVIGIAL-

>Tethya.wilhelma_20_CuZnSOD

TNTAAVCVLEGA-EVKGTISFTPREGSSAVRVTGQVTGLSPGNHGFHIHQFGDYSAGCVSAGPHFNPAGKEHGGPTDEERHVGDLGNIVADASGTATVAITDSQLTLSGPNSIIGRSVVVSDSTTEYTR--------------------------

>Tethya.wilhelma_21_CuZnSOD

-----------------MLSFSSTQGSSAVRVTGQVTGLSPGNHGFHIHQFGDYSAGCVSAGPHFNPAGKEHGGPTDEERHVGDLGNIVADASGTATVAITDSQLTLSGPNSIIGRSVVVSDSTTEYTR--------------------------

>Tethya.wilhelma_22_CuZnSOD

MSTVGVCVLEGE--VKGTIRFQPE--GSAVKVTGEVTGLKPGKHGFHIHQFGDYSAGCVSAGSHFNPAGKEHGGPTDEERHVGDLGNIVADDSGKAVVAITDSQLTLSGPNTIIGRSVVSLDDSDSMYFGDEDFSFSDGSGGNRLICSTLKLPKR

>Tethya.wilhelma_23_CuZnSOD

MSTVGVCVLEGE--VKGTIRFQPE--GSAVKVTGEVTGLKPGKHGFHIHQFGDYSAGCVSAGSHFNPAGKEHGGPTDEERHVGDLGNIVADDSGKAVVAITDSQLTLSGPNTIIGRSVVSLDDSDSMYFGDEDFSFSDGSGGNRLICSTLKLPKR

>Drosophila.melanogaster_16a_CuZnSODSOD1

MVVKAVCVINGD--AKGTVFFEQESSGTPVKVSGEVCGLAKGLHGFHVHEFGDNTNGCMSSGPHFNPYGKEHGAPVDENRHLGDLGNIEATGDCPTKVNITDSKITLFGADSIIGRTVVVHADADDLGQGGHELSKSTGNAGARIGCGVIGIAKV

>Drosophila.melanogaster_16b_CuZnSODSOD1

MVVKAVCVINGD--AKGTVFFEQESSGTPVKVSGEVCGLAKGLHGFHVHEFGDNTNGCMSSGPHFNPYGKEHGAPVDENRHLGDLGNIEATGDCPTKVNITDSKITLFGADSIIGRTVVVHADADDLGQGGHELSKSTGNAGARIGCGVIGIAKV

>Arabidopsis.thaliana_2_SODCuZn

AAKKAVAVLKGTSDVEGVVTLTQD-DSGPTTVNVRITGLTPGPHGFHLHEFGDTTNGCISTGPHFNPNNMTHGAPEDECRHAGDLGNINANADGVAETTIVDNQIPLTGPNSVVGRAFVVHELKDDLGKGGHELSLTTGNAGGRLACGVIGLTPL

>Xestospongia.bergquistia_25_CuZnSOD

-------MMKAK-ENMGLVNSKQDKLSGLCYITGEVTGLTPGKHGFHIHEFGDYSAGCVSAGGHFNPHRKNHGGPDDTDRHAGDLGNIVADDSGKATINITDKQIPLSGENNIIGRSVVVHADPDDLGKGGFPDSLTTGHAGGRLSCGVIGYAKS

>Ciona.intestinalis_24_CuZnSOD

MVLEAVCVMKGSESVSGTIKFSQVGDGEPCKISGSLTGLAAGKHGFHIHEFGDHTNGCTSTGGHFNPQKCDHGAPEAEVRHFGDLGNVTADSSGVAEVNISDKYVTLTGINSVIGRAVVVHADVDDLGLTSHPQSKTTGNAGGRLACGVIGITNH

>Dictyostelium.discoideum_1_SODCuZn

MSKTAVCVIKGE-KVNGVVKFTQENKDSPVTVNYDITGLEKGEHGFHVHAFGDTTNGCVSAGPHFNPFGKNHGAPSDEDRHVGDLGNIVADGESNTKGTISDKIISLFGEHTIVGRTMVVHADQDDLGKGGKPDSLTTGAAGARLGCGVIGVSQ-

>Dictyostelium.discoideum_3_SODCuZn

--MSAICVIKGD-GVDGIINFKQNDNKSPVIISGVISGLKEGKHGFHVHEFGDTTNGCLSAGAHFNPFKKEHGSPNDENRHVGDLGNIESNKDKKSIINITDNIITLFGQNSIIGRSIVVHDKEDDLGRGNSQDSKITGNAGSRLGCGIIALSKI

>Mnemiopsis.leidyi_27_CuZnSOD

--MNAICVLRNDKESFGCIKFSQASEGAPTTLKVEISSLTEGEHGFHMHQFGDNTNGCISAGPHFNPFGKTHGGPTDEVRHVGDLGNVVVDGSGVCSTEITDPQVSLFGANSIIGRTMVLHAGVDDLGKGGNEESLKTGNAGGRVACGIVGLCS-

>Dictyostelium.discoideum_2_SODCuZn

-MVKAICVVKGA-VVNGTIIFSQENEGSPVYVNGTISGLSGGLHGFHIHEFGDTSNGCLSAGAHFNPFHVEHGGPNSAIRHVGDLGNITSCSSKVANVLIQDNVISLFGDLSIIGRTLVVHENQDDLGLGGN-LSKTTGNAGARVACGILAKI--

>Dictyostelium.discoideum_4_SODCuZn

-MVNAIVIIKGL-GVEGKVTLSQECEGSPIYINGTVSGLTPGQHGMHVHEFGDTSNGCISAGDHYNPLHREHGSPLDVERHIGDLGNIKALSNGVATISIRDTIMSLFGDISVMGRTMVIHSDRDDYGRGNFPDSKTAGHSGKRVGCGIIAKI--

>Caenorhabditis.elegans_29_CuZnSODSOD1

MSNRAVAVLRGE-TVTGTIWITQKSENDQAVIEGEIKGLTPGLHGFHVHQYGDSTNGCISAGPHFNPFGKTHGGPKSEIRHVGDLGNVEAGADGVAKIKLTDTLVTLYGPNTVVGRSMVVHAGQDDLGEGKAEESKKTGNAGARAACGVIALAAP

>Caenorhabditis.elegans_30_CuZnSODSOD1

MSNRAVAVLRGE-TVTGTIWITQKSENDQAVIEGEIKGLTPGLHGFHVHQYGDSTNGCISAGPHFNPFGKTHGGPKSEIRHVGDLGNVEAGADGVAKIKLTDTLVTLYGPNTVVGRSMVVHAGQDDLGEGKAEESKKTGNAGARAACGVIALAAP

>Caenorhabditis.elegans_32_CuZnSODSOD5

ESKRAVAVLRGT-AVFGTVWLTQKAEGEETEFEGEIKGLSPGLHGFHIHQYGDSTDGCTSAGPHFNPCKMNHGGRDSVVRHVGDLGNVEAGADGVAKIKFSDKVVSLFGANTVIGRSMVVHVDRDDLGQGKAEESLKTGNAGARAACGVIALAAP

>Amphimedon.queenslandica_26_CuZnSOD

PVARAVCILASSDDVKGTIEFIQN-EQGITKVTGKVTSLAPGDHGFHIHQFGDYTSGCVSAGSHFNPAGKNHGGPKDGERHAGDLGNITS-TGGDTEIELYDDQIPLTGPNSIIGRSVVVHADPDDLGKDGHPDSLTTGHAGARLACGVIGSTKL

>Fonticula.alba_CuZnSOD_1

-MTKAVAVLNGLNGLKLVATFSQQSPDSPTVIDIEGSGIKPGLHGFHVHEFGDTTNGCVSAGPHFNPHGHTHGAPSAAIRHVGDLGNLSVDETGSVKVTITDHLVSLSGVNNVVGRALVLHADVDDLGLGGHELSATTGNAGDRIACGVIGITK-

>Strongylocentrotus.purpuratus_28_CuZnSOD

MSVKAVCMLVGE-AVKGRIEFEQGEGSNSVSV-----------------------KGCVSAGGHFNPFGKEHGAPEDEMRHVGDLGNIIADASGKVDVNLSDKLLSLSGPQSIIGRAVVVHADVDDLGKGGHATSKTTGNAGGRLACGVIGIQA-

>Caenorhabditis.elegans_33_CuZnSODSOD4

EVIRARAIFKGKTELIGTIDFDQS--GSFLKLNGSVSGLAAGKHGFHIHEKGDTGNGCLSAGGHYNPHKLSHGAPDDSNRHIGDLGNIESPASGDTLISVSDSLASLSGQYSIIGRSVVIHEKTDDLGRGTSDQSKTTGNAGSRLACGTIGTV--

>Caenorhabditis.elegans_34_CuZnSODSOD4

EVIRARAIFKGKTELIGTIDFDQS--GSFLKLNGSVSGLAAGKHGFHIHEKGDTGNGCLSAGGHYNPHKLSHGAPDDSNRHIGDLGNIESPASGDTLISVSDSLASLSGQYSIIGRSVVIHEKTDDLGRGTSDQSKTTGNAGSRLACGTIGIVEE

>Acanthaster.planci_1_CuZnSOD

-----------------------------MNVSVQLTGLDATLHGFHVHQVGDLSNGCASTGGHFNPFDEQHGGPSDTQRHVGDFGNVERDSVGSVNEEFSDEVASLVGADTIIGRAIVLHKDTDDLGRGDYDDSLTTGHAGARLACCIIEQV--

>Lingula.anatina_4_CuZnSOD

MYTVGHCDMRGVHGISGDIYLVQR--GNLLEVRVNISGLPGNKHGLHVHTYGDLSDGCASTGGHFNPANVRHGSPTDNKRHVGDWGNVERDSDGNVVTAFLDSVASLWGPNTIIGRAIVIHASEDDLGRGGDKGSSLSGNAGPRLACCVIGISNG

>Lingula.anatina_35_CuZnSOD

QGVFAHCALKGTPEVHGNIDMIQR--QGVLEVRVNISGLPSQEHGLHVHAFGDLSGGCGSTKGHYNPTGVTHGAPTDVVRHIGDWGNVPQDKNGQIVTSFFDGIASLVGKNNIVGRAIVLHTGKDDLGRGGNAASLANGNAGPRLGCCVIGITNG

>Branchiostoma.floridae_2_CuZnSOD

-------------------------------------------YGVTVHELGDQSKGCTSTRDHFNPFNLGHGGPKDEDRHLGDWGNVEADSTGEVNAIIKDEVASLVGPYSIIGRTIVIHANEDDLGRGETAESKKTGNVGKRLACCVLG----

>Acanthaster.planci_3_CuZnSOD

ENVYAHCRMVDPEPIVGDINMRQRAVGGQMDVKVFLYGFMKSVHGIHVHTYGDLGNGCGSAGGHYNPGGNNHSAPYDKDRHMGDWGNIQVDDFGMVEHTFSDKVATLVGPNSILGRTIVIHIGEDDLGKGGTADSKTTGNAGGRLGCCVIGHSDG

>Capitella.teleta_37_CuZnSOD

SYIYAQCQVADTNHVIGTIDLRQTVGRAVSEIRLNLTGFADGYHGFHVHELGDLSNGCDSTGSHYNPLDVDHGAPHDFNRHIGDLGNIEEDLGGNCIRTITDTLVTLQGRFSVIGRALVIHETYDDLGRSGVDDSLTTGNAGARLSCCVIGLTDD

>Capitella.teleta_38_CuZnSOD

-------------------------------------------HGIAIHQYGDLSEGCTNLGDHFNPLNVNHGGRLQFQRHVGDLGNIHVNLDGSASSELKDYLIQLQGRYTVVGRSVAIHELTDDLGKGGDSDSSTNGHSGSAIAC--------

>Nematostella.vectensis_41_CuZnSOD

-------------------------------INVRLRGVPPLVHGFHIHKSGDITKGCQSAKGHFNPYGKTHAGPRKRDRHVGDLGNVWSDYHGNVRTSFFDHMVSLYGPDSVIGRSIVLHAERDDLGRGIGEGSLATGNAGARLACCVIVH---

>Drosophila.melanogaster_36a_CuZnSODSOD3

MPIQAIAYLIQSTQVKGNVTFTQNDCGQNVHVRVQLEGLKEGKHGFHIHEKGDLTNGCISMGAHYNPDKVDHGGPDHEVRHVGDLGNLEANSTGIIDVTYTDQVITLTGKLGIIGRGVVVHELEDDLGLGNHTDSKKTGNAGGRIACGVIGINGP

>Drosophila.melanogaster_36e_CuZnSODSOD3

MPIQAIAYLIQSTQVKGNVTFTQNDCGQNVHVRVQLEGLKEGKHGFHIHEKGDLTNGCISMGAHYNPDKVDHGGPDHEVRHVGDLGNLEANSTGIIDVTYTDQVITLTGKLGIIGRGVVVHELEDDLGLGNHTDSKKTGNAGGRIACGVIGIK--

>Drosophila.melanogaster_36d_CuZnSODSOD3

MPIQAIAYLIQSTQVKGNVTFTQNDCGQNVHVRVQLEGLKEGKHGFHIHEKGDLTNGCISMGAHYNPDKVDHGGPDHEVRHVGDLGNLEANSTGIIDVTYTDQVITLTGKLGIIGRGVVVHELEDDLGLGNHTDSKKTGNAGGRIACGVIGINSD

>Ephydatia.muelleri_40_CuZnSOD

ETTRAVSVMSGPTDVKGVIVFESL-GHDWIRVTGSITGLQPGPHGFHVHKLGDLTNGCVSAGEHFNPFRQTHGGLKSKVRHAGDLGNIVANAEGVAMVDLVARQMALSGPLSIIGRSIVIHADKDDLGLGHEPDSPTTGHSGTRVGCGVIGR---

>Capitella.teleta_39_CuZnSOD

EKITAECVIEGTGPIQGTIRFEQDITGGATSITGSVSGFTTGKHGFHVHAVGNLGNRCSDATGHYNPFDKNHGAPDASERHVGDLGNIVENANGVADISMDDSLVSLVGEYTVIGRSIVVHAGEDDLGLGGDSGSLTTGNAGARLGCCIIQEVGG

>Branchiostoma.floridae_42_CuZnSOD

-------------------------------------------HGFHIHADGDLSMGCDTAGPIYDPFNKSHGGLNTTERKVGDLGNLDCDELGRVMMLLEVEDASLLGPYSILGRSMVIHANEDDFGHGGHEMSPVDGNSGVRLACCVIGRTSD

>Oscarella.carmela_43_CuZnSOD

QDLVAVANLVSDRTVTGTVLFHQLAEGGETLVRGVVKGLQAGRHGFHIHQQSDNTLGCSSYGGHYNPFHAHHSGRFDSERHVGDLGNIEFEETGVASFTFRDSLAQLLGPQSVLNRGVVIHARGDDLGKGGDEGSRKTGNAGHRVACGPISCMDN

>Mnemiopsis.leidyi_44_CuZnSOD

-----MAVLYGQLDLVGIVQFTQAGPAAKLMVNINVEYFADGLHGFHIHQMGNVYGGCTQTLGHYNPLDVLHGGPDSAVRHTGDFGNIEIKNHA-FTGEHTDSQASLYGQYSIIGRGVVLHETQDDLGKGGDLGSQKTGNAGSRLACGVIGISSE

>Mnemiopsis.leidyi_50_CuZnSOD

STKKASCIMKGENTEVGKVTFSVHDNTQTVEAHLHNAAIAPGLHGFHVHTLGVDGYDCGSTGGHFNPYKKYHGGLDKDSRHLGDFGNVLAGDRGEINYTVTSPQFELEGENSIVGRAVVLHAGQDDLGQGGDDGSLATGNAGSRVACCTLHLEYA

>Mnemiopsis.leidyi_51_CuZnSOD

AIRRASCSMKGKDKVIGKVTFSEHNNIQTIHAHLHEDEIVPGLHGLHIHTDGVTDNDCASSGGHFNPDGNKHGGLDADLRHVGDFGNVEAGERGEINYTIHSPRFNLKGKNSVVGRAVVLHAGEDDLGLGGDEGSRKTGNAGERVACCTLEL---

>Mnemiopsis.leidyi_52_CuZnSOD

--------MKNNGTDIGEVSFSSSGNTQEVSVFLQTSKIPQGYYGLHVHTFPVIEFDCSSTGDHFNPKGNRHGSSSANDRHVGDFGNIRAGERGEINASIKSPLFRLEGEDSIVGRAAVIHAHEDDQGKSGTAKSTKTGNAGKPIACCTLQLV--

>Danio.rerio_45_CuZnSOD

DSLQAVCRMQGMPRVYGHILFRQSGPKEKLSVTFRLYGLPQQPRAMHIHEYGDLSRGCDSTGGHYNPLNVNH------PQHPGDFGNFVP-VNKKIRQSL-ESPATLFGKLSIVGRSVVIHEGKDDLGRGGNVGSLLNGNAGGRLACCVIGLRNP

>Homo.sapiens_46_CuZnSODSOD3

GALHAACQVQAQPRVTGVVLFRQLAPRAKLDAFFALEGFPSSSRAIHVHQFGDLSQGCESTGPHYNPLAVPH------PQHPGDFGNFAV-RDGSLWRYRAGLAASLAGPHSIVGRAVVVHAGEDDLGRGGNQASVENGNAGRRLACCVVGVCGP

>Xenopus.tropicalis_48_CuZnSOD

GIAYATCSLSSEVKVTGLVLFKQVFPSGTLEAIFDLEGFPQSARAIHIHTYGDLTNGCDSAGGHYNPMSVDH------PQHPGDFGNFRV-RDGKIQKFFANLDATLFGPFSVIGRSVVVHKQADDLGKGNNQASLENGNAGKRLACCIIGSSSK

>Danio.rerio_49_CuZnSOD

NTIYATCEVSGQPKIFGQVLFRQVFPNGTAEVKINLRGFPNQVRAIHIHQYGDLSQGCVTAGPHYNPQDVPH------PNHPGDMGNFMP-KQGLIRRFLKLPEVKLFGGQSVLGRAVVVHEKEDDLGMGADEESKRSGNAGRRIAGCVIGITKP

>Mnemiopsis.leidyi_54_CuZnSOD

AEYEAECTMKNTEIEIGKVVFKSGEGFQSIQANLEGEVITEGLHGFHVHEKPVMGNDCASTGGHYNPDGSNHSNSPSGERHAGDFGNVMAGQRGEINLDMKITVSTMLGEPSMRCAPIKVKVYPDGMKRSGKRNARRKGNRAPRNACQPLSLNFK

>Mnemiopsis.leidyi_59_CuZnSOD

---RASCYLRNDGRIIGTAKFYSITGYQNVSVDLYDSGLTDGLHGFHIHEQGVTGCDCLTTGGHYNPDGVNHGGGAASSRHVGDFGNVESRMSKASEVNLESPKLFFTGPFTEISKTITLKNAT---------KSKTTENQGSAIATVILQPIEE

>Mnemiopsis.leidyi_53_CuZnSOD

------------------------------------ANLLKDQAQWKDHSDPECRPGCDRTGAIFKKRG----------RVLGDLGNVRAGERGEVNHTLNKPLFHLQGKFSIVGRAVLLQVGS---------LTSVDGSPGDAIACCTL-----

>Pseudanabaena.ABRG5-3_CuZnSOD

TQSRASSRIFVKGDLVGTATFIQTPLG--VKVDLKVQNLAQGEHMVHLHEQGK----CKTSGNHFDPEEKD-DDDHGKHKPAGDLPNIIVKQDGNGNLTALLPKLTLSGKNSLLGTSILIHAGANGK------STIPNVDFKTRIACGVIKS---

>Pseudanabaena.PCC7429_CuZnSOD

NPQSAIASIFIKGEQVGTATFTQTPLG--LQVIVNVENLTQGEHMIHIHENGK----CKTSGNHFNPKNPKDDDNHEKHNPAGDLPNIVVKKDGKGKLNALLPRLSLNKPNSLLGTSILIHAGANGN------STIPNVDYKTRIACGVIKK---

>Pseudanabaena.SR411_CuZnSOD

LKSSASSQIFAKGELVGTATFTQSTSG--VKVTLQVQKLAQGEHMVHLHENGK----CKSAGNHFDPKRSPMDATHDQHQPAGDLPNIMVQQDGTGTLTATLSELTLTGHNSLLGTAIVIHAGANGK------STIPNVDYKTRIACGVVKS---

>Pseudanabaena.PCC7367_CuZnSOD

SDKGAMAMIKIEGQLVGTASFRDTEAG--LEVIVEAQNLAPGEHAIHIHEVGK----CKSAGGHFNPSNPSHSSKHHDHGAAGDLPNLNISRDGTGVLTAVLPDLALDSSNSILGTAVLIHAGAAGV------TTIPGVDADTRVACGVVSAA--

>Gloeobacter.kilaueensis.JS1.GKIL_3379_CuZnSOD

NPPQATAQIKAKGEPVGTASFVQQPKG--VLVTVEVKGLEPGKHPMHIHAVGK----CRSAGPHLGGMK--------GMTMAGDLPELLVGADGTGKAEVLNPEISL-GSKSLLGTALLIHDAT---------------KPSKRIACGVISTNP-

>Gloeobacter.violaceus.PCC7421.glr1981_CuZnSOD

EQPAAVSAIKLNGQVVGTATFRQQPEG--VLVNIQVQGLQPGKHAMHIHAVGK----CKSAGPHLGPTSGHHGAATMHAAMAGDLPDLVVGSDGSGKAEILNAEVRLPGRNFLLGTALLIHDVA---------------DPNQRIACGVISKAPN

>Gloeobacter.kilaueensis.JS1.SODC_CuZnSOD

GTRQATAALVSNGQTVGTAALSQSSAG-VLSVKLDVKGLPPGEHGIHFHAVGL----CTSAGGHFNPATRQHGLENPKGAHAGDLPNLSVAADGTASYSATDVRASLPNANNLLGTALIIHARPDDL------KSDPVGNSGARIACGVLKPAK-

>Gloeobacter.violaceus.PCC7421.glr2170_CuZnSOD

RWLQAKIELKADGLPVGNASLTQLSDG--VRVSVQVQGLLPGKYPIHFHSKGK----CRSSRGVFDTHSLGHKRPDQPVPPAGLLPALIVGAAGTGELNALNTDVTLAHKHSLLGSALVIHAA----------------HSRQIIACGAVTRTPV

>Drosophila.melanogaster_55a_CuZnSOD

PQWQAGAKLMGEAGVAGMISFVQLPYNSDIRVTINVTGLPPGKHALHIHTFGDLSDGCKSTGGQFNNF----------------LGNVDTKDDGSISAVFQSIYLQLFGINGIVGRSIVIHSKAIDLNTALNAEVFSSLQAGPAIACGVISISTA

>Drosophila.melanogaster_55b_CuZnSOD

PQWQAGAKLMGEAGVAGMISFVQLPYNSDIRVTINVTGLPPGKHALHIHTFGDLSDGCKSTGGQFNNF----------------LGNVDTKDDGSISAVFQSIYLQLFGINGIVGRSIVIHSKAIDLNTALNAEVFSSLQAGPAIACGVISISTA

>Capitella.teleta_56_CuZnSOD

---EVVMQVSGNGPTVGKVVIHYADDAQGVRFQPALHDLPPGAHGFHVHENPSCEPGELAAGSHYDPEKTGHHEGPKGHGHLGDLPALTVDDYGNAVTPVEAPRLSLP---ELKGHSLVIHAGSDNY-----SDHPPLGGGGARIACGIIQ----

>Acanthaster.planci_61_CuZnSOD

PSKTVSAEISVSG-VNGSLEFRQRSPYDATHISVNLANLQ--AGGYHVHEQPVDDLPADNVGGHYNPFGVDAGGGTHDQYEVGDISGKFGRLTDAFSDQFVDWNMQLFGKYSVIGRSIVIHKEENAAGEIHADGSDTTNHNAPRYACANLYCLPT

>Acanthaster.planci_62_CuZnSOD

KPKSSEAMISVNN-VSGMVRFRQSSPYDVTEIKVHLENLR--AGGFHVHVLPPSDQPAQNVAGHFNPYGIGA-AGTNDQYEVGDLSGKFGLLTDNFNATFLDWNLPLFGRHSIIGRSLVIHKKEGDVGKIYADGTDTTGHNSPRISCADLLEVPP

>Strongylocentrotus.purpuratus_66_CuZnSOD

KSKTVRAEFSQNN-VTGIVTFTQASLWESTNITVDLENLR--AGGYHVHKFPVSDFPGDNVAGHFNPYNIDTTAGTNDQYELGDISGKFGSLADDFSGTFEDWNMPLFGANSIVGRSVIIHLASSEVGQIHSDGEDTTEHDPERLGCGDTLDVFQ

>Danio.rerio_65_CuZnSOD

SEKTVNAVLNMQG-IKGYFSFQQKSPFDLTTITVNLTNLN--VGPYHVHQFPLSDSSCNNAGGHWNPFNVNQAPSTHDRFEVGDLSSRHGSLESNFQATLIDWNLPLFGWNSIVGRSVVIHMPNGTVGTVYGQSAQSTNHSATRIACANLTLYRF

>Xenopus.tropicalis_67_CuZnSOD

RSKTASAHVSMNG-VSGHFNFRQNSPLHPTEIDLNLRNLK--GGEYSIHSLPVGENLCNSTGDIWNPLGVNSASSAHHLWEMGDLSGCHGSLQEEMRTKLIDWNLPLYGNNSVVGRSVVLSKANGTVGRVSLSGQ-TSGHNAPRLACANIIPQHP

>Branchiostoma.floridae_63_CuZnSOD

EAVSVSARFDMSG-VKGTMVFSQNSPYEYTTVTININNIG--AINYHVHEYPIDESMCTYTAGHLNPFGKVSSDGTGDQYELGDLSGKFGNFNDTVSTVNTDWYLPLYGRYSIVGRSVIIHDTTGSAGHMYIDGTAGTDHSGGRLACANIFQLPA

>Sycon.ciliatum_68_CuZnSOD

ESREMSATFDMQG-VKGVMVFSQQSPWDPTWVTLDLSGLA--AAGYHVHEFPRYGSACQEVGGHWNPFRAPFPVSTPDLYEVGDLSGKHGSLAQSVQQEMYDGQLPLFGPHSIAGRSIVIHRPNAPAGTIRTDGGPSSGHHAPRLACATIEPYAD

>Sycon.ciliatum_69_CuZnSOD

ESREMSATFDMQG-VKGVMVFSQQSPWDPTWVTLDLSGLA--AAGYHVHEFPRYGSACQEVGGHWNPFRAPFPVSTPDLYEVGDLSGKHGSLAQSVQQEMYDGQLPLFGPHSIAGRSIVIHRPNAPAGTIRTDGGPSSGHHAPRLACATIEPYAD

>Sycon.ciliatum_70_CuZnSOD

RSRRATAVFDYGG-VRGRVELNQQSPWDNTIVSVQLNGLM--AGGYHIHEWPVSTRFCQAVGGHYNPFSAST--LTQDFYEVGDLSAKFGGLANSLNATYSDTYITLFGPYSVVGRSIVLHNASSPVGSILISGNSTASHNGGRLACADISRFAV

>Ciona.intestinalis_75_CuZnSOD

NPRSAVTRFAWSN-LRGTVTFTQDSPFDVTMTSVDLLGVQ--NATYHIHKFPVSENKCNNVVGHYKPFGASSVGGTHDQYEVGDLSGKYGKLTATFAMNYTDWNLPLFGVHSILGRSVVVHKSSSEYGYMNTDHG-VSWHN--------------

>Amphimedon.queenslandica_71_CuZnSOD

ANLTAIATVSQRG-VTGTIIFTQNTPTSETNIRLTLTGLP--LYQWHVHQYPFLPSPCPNTGGHFDPLGAFNQANNPAACEIGDLSGKFGLLNSDLPFSEGDDTISLYGRHSIIGRSIVFHFSNGTVGNISA-----TDHNAPRVACGNLTALKP

>Xestospongia.bergquistia_74_CuZnSOD

GSLTAIATFSQNG-ISGTITFAQNSSSSPTSIHVNLTGLS--LYQWHVHQYPFLPSPCANVGGHYDPLGASNNNMNKTICEIGDLSGKFGLLNSLLPLNINDSSLSLYGYQSIIGRSVVLHLQNGTFGNISAN---PTRHDAPRIACGNLTRLQS

>Tethya.wilhelma_72_CuZnSOD

SQLTVSADFNQRG-VKGSITFSQNDPQSSTTISVNLTGLDGGPFQWHVHEYPYSGDICAVVGGHYDPLGANNVVSNRQACEVGDLSGKFGPLPMTVTLSLDDQFLSLYGVYSIIGRSIVIHLNDNSTGSIRVMNEENSGHNGPRIACANITLYRP

>Tethya.wilhelma_73_CuZnSOD

SQLTVSADFNQRG-VKGSITFSQNDPQSSTTISVNLTGLDGGPFQWHVHEYPYSGDICAVVGGHYDPLGANNVVSNRQACEVGDLSGKFGPLPMTVTLSLDDQFLSLYGVYSIIGRSIVIHLNDNSTGSIRVMNEENSGHNGPRIACANITLYRP

>Ephydatia.muelleri_111_CuZnSOD

---------------------------------------------------------C-----------------------LGDLSGRYGYLNSSSTFSASDADLSLYGAYTIFGRSVVLNWS--TAGNVAVNGLVNSGHNLARLACANISLLFP

>Ephydatia.muelleri_107_CuZnSOD

------------------------------------MGLE--SYTWRVHQYPSQHDPCSAAGGCYDPQYVGQQPANQSLCEIGDLTGRHGPLTTRAVSVIEDSCLNLYGVSNIVGRSLVLNWE--TVGAVIANDLMESEHSTNVIACASMRVLSQ

>Amphimedon.queenslandica_108_CuZnSOD

RAILVAAYFDMNG-IKGSVAFYQEKEHEPVTITLSLSGLSGERWGWHVHEYPIERNPCEHIGGHYDPENRASDPNNASLCEVGDLGGRHGHLRNQSVYTFTDNTLNLYGPYSIVGRSLIIHRTNGFQGEVRVDGGSINTHNGNNLACAKFEPVVP

>Xestospongia.bergquistia_109_CuZnSOD

IPINVGAYFDMNH-ITGTIQFYQEREGKPVVIHIVLNGLDEEMWGWHIHEYPIESVSCPSVGGHYDPEKVGLASSDPKNCEVGDLSGRHGTLKNQTTYTFTDSTLNLYGSYSLVGRSIVIHRTHGYQGEIRVDNEPNEEYSGNIVACTKFETVTP

>Ciona.intestinalis_94_CuZnSOD

--------------------MIQPNEFSATSVTANLAGLA--AGGYHIHILPLGVGACSVISGHYSPFDPAI--GTTDQYEVGDLSNKYGLLTASLSLPVTDDNLPLSTDVSVAGRSVLIHKASGTTGYINNNGS-LTNHNSARIGCSNIQPINS

>Amphimedon.queenslandica_76_CuZnSOD

GQTYGRATFNIRG-ITGYITFMPQPN-NRMMIMSNISGTN--NHHWHVHLLPVPAMRCDLIGGHFDPLGAVSGSNNQSACEVGDLSGKVGPLTGVRNYNETTGELVLFGVNGIIGRSIKIHGTE-DGGVVWVNNSSSTNHPNNVLACAPLVQVET

>Xestospongia.bergquistia_77_CuZnSOD

GQTYGIANFNMRG-IQGTIKFMPNDN-DHVNITVDIQGTT--DHVWHVHNFPVPQTRCDNIGGHFDPFGANAGSNNQTVCEVGDLSGKVGNLTGTFNYIDKSGDLTLFGRYGIIGRSIKIHGND-DGGTIWVTNSQTTNHKTPVMACAPLIEVET

>Tethya.wilhelma_78_CuZnSOD

ASHVAVVRFNSRG-IQGNITFTELGN-GNVRIVANLQGLR--FHNWHIHHYPVPAIQCVQIGGHYDPFGRSTDPANQTLCEVGDLEGKFGPLPGQVDVVDTTGQLALSGRYSVVGRPINIHDSVDLAGTIWVNDSESTQHN--------------

>Tethya.wilhelma_79_CuZnSOD

ASHIAVVRFNSRG-IQGNITFTELGN-GNVRIVANLQGLR--FHNWHIHHYPVPAIQCVQIGGHYDPFGRSSDPANQTLCEVGDLEGKFGPLPGQVDVVDTTGQLALSGRYSIVGRPINIHDSVDSAGTIWVNDSESTQHNGPALACAPLVVSEE

>Tethya.wilhelma_80_CuZnSOD

ASHIAVVRFNSRG-IQGNITFTELGN-GNVRIVANLQGLR--FHNWHIHHYPVPAIQCVQIGGHYDPFGRSSDPANQTLCEVGDLEGKFGPLPGQVDVVDTTGQLALSGRYSIVGRPINIHDSVDSAGTIWVNDSESTQHNGPALACAPLVVSEE

>Tethya.wilhelma_81_CuZnSOD

GYKIATATFNAQG-IEGYITFTDLGN-GSIRINTNLDGLR--MHNWHLHHFPVPDVQCQLIGGHYDPFGRNNDSADINMCEVGDLEGKFGPLPGQVDVIDTTGELTLRGRYSIVGRPINIHDHIDSAGTVWVNDSESTDHNGPALACAPLMESHN

>Tethya.wilhelma_82_CuZnSOD

GYKIATATFNAQG-IEGYITFTDLGN-GSIRINTNLDGLR--MHNWHLHHFPVPDVQCQLIGGHYDPFGRNNDSADINMCEVGDLEGKFGPLPGQVDVIDTTGELTLRGRYSIVGRPINIHDHIDSAGTVWVNDSESTDHNGPALACAPLMESHN

>Tethya.wilhelma_83_CuZnSOD

------------------------------------------MHNWHIHHFPVPDVQCQLIGGHYDPFGRNNDSADTNMCEVGDLEGKFGPLPRPVQFVDTTGELTLNGRYSIVGRPINIHDHIDSAGTVWVNDSESTDHNGPALACAPLMESHN

>Tethya.wilhelma_84_CuZnSOD

------------------------------------------MHNWHIHHFPVPDVQCQLIGGHYDPFGRNNDSADTNMCEVGDLEGKFGPLPRPVQFVDTTGELTLNGRYSIVGRPINIHDHIDSAGTVWVNDSESTDHNGPALACAPLMESHN

>Tethya.wilhelma_85_CuZnSOD

AQRSATARFNSRG-IRGYITFTEEGP-ENVRIVANLDGLR--LHDWHIHHYPVPTVQCRQVGGRYNPFNNRSDPNDQSLCEVGDLENKFGPLPGQFNGVDTSTQLDLTGRYSIVGRPVVIHQSDTDAGTIWVNDSMSTEHNGPALACAPLVRSEN

>Tethya.wilhelma_86_CuZnSOD

AQRSATARFNSRG-IRGYITFTEEGP-ENVRIVANLDGLRRSLHDWHIHHYPVPTVQCRQVGGRYNPFNNRSDPNDQSLCEVGDLENKFGPLPGQFNGVDTSTQLDLTGRYSIVGRPVVIHQSDTDAGTIWVNDSMSTEHNGPALACAPLVRSEN

>Tethya.wilhelma_87_CuZnSOD

ASHSARVVFNSRG-IRGYITFTEQED-GNIRIVANLTGLR-GLHLWHIHNFPVPADQCNQLGGFYDPFGALANQQNQSLCAAGDLASKFMPLPGQLDIVDTTGQLSLSGRYSIVGRSLVIHVPDATAGTIWVDDTTSTGHN--------------

>Tethya.wilhelma_88_CuZnSOD

----------------------------------------------------------------MEPYG-------------------------------------------------------------------------PGIACAPLVMSEN

>Tethya.wilhelma_89_CuZnSOD

----------------------------------------------------------------MEPYG-------------------------------------------------------------------------PGIACAPLVMSEN

>Xestospongia.bergquistia_90_CuZnSOD

TPLEATARINLKG-VVGFVKFKTLNE-SAIQITTNFSGLPR-GLNWHVHRFPVPDHRCIYVGGHYDPLMARANEGNPFACEIGDLTGKFGQLQGYFVHVDNTGLLELGGLRGIVGRSIVVHASNGIAGTIWVNGSPTTRHNAPIIACAPLVRTEE

>Tethya.wilhelma_91_CuZnSOD

PENVATAVFGANG-ITGWIRFFEYED-DRVVIIVNMVGFA--NASWSMHDLPVPSEKCDYLGPVYDPMGMY----NATLCEAGDLGASYGLIDTPQLFNDSMTFITLSGPTSIVGRSLVVKNSEGVAGSIWVNDNEISDHNEVI-----------

>Tethya.wilhelma_92_CuZnSOD

PENVATAVFGANG-ITGWIRFFEYED-DRVVIIVNMVGFA--NASWSMHDLPVPSEKCDYLGPVYDPMGMY----NATLCEAGDLGASYGLIDTPQLFNDSMTFITLSGPTSIVGRSLVVKNSEGVAGSIWVNDNEISDHNSDIVACAPIVLSEV

>Sycon.ciliatum_96_CuZnSOD

QSRRAVAEFSLNG-VRGSLVFEDSSSSSGNWVQIDSHGID--PLTWHVHLYPFPMHRCTSTGGHYDPHGKATAVSSGRDCEVGDLSGKFGPLRRTLTPNMS--------VAELIGRSVVLHVFSGEAGSVTVSGLPSTGLNSQRVACAWIEEVQP

>Sycon.ciliatum_98_CuZnSOD

QSRRAVAEFSLNG-VRGSLVFEDSSSSSGNWVQIDSHGID--PLTWHVHLYPFPMHRCTSTGGHYDPHGKATAVSSGRDCEVGDLSGKFGPLRRTLTPNMS--------VAELIGRSVVLHVFSGEAGSVTVSGLPSTGLNSQRVACAWIEEVQP

>Sycon.ciliatum_97_CuZnSOD

QSRRAVAEFSLNG-VRGSLVFEDSSSSSGNWVQIDSHGID--PLTWHVHLYPFPMHRCTSTGGHYDPHGKATAVSSGRDCEVGDLSGKFGPLRRTLTPNMS--------VAELIGRSVVLHVFSGEAGSVTVSGLPSTGLNSQRVACAWIEEVQP

>Sycon.ciliatum_99_CuZnSOD

QSRRAVAEFSLNG-VRGSLVFEDSSSSSGNWVQIDSHGID--PLTWHVHLYPFPMHRCTSTGGHYDPHGKATAVSSGRDCEVGDLSGKFGPLRRTLTPNMS--------VAELIGRSVVLHVFSGEAGSVTVSGLPSTGLNSQRVACAWIEEVQP

>Sycon.ciliatum_100_CuZnSOD

AQLNARADFHMNG-VGGNILFSWNP--QSHQMTINTMGLKS-GLTWHVHEFPFSPDPCSSTGGHFDPLGRASAPQMSRDCELGDLSGKFGTLQLR-----ETPNLSLSGNFGFVGRSVVIHR--GAAGEVWLDGNPASGYMTRRIACATIHEVQP

>Ephydatia.muelleri_106_CuZnSOD

SQSAAVATFDANGVVKGTVRFTALA--DGLQVQVSLSGLS--THPWHVHVNQLSTGYCDYVDAHFDPFNANTSTANQSLCEAGDLAGKFGSIPGTYGGTYSDTFLDTTGVSDCVGRSVVVHNFTGS-----------------------------

>Sycon.ciliatum_101_CuZnSOD

SSFTLSAYFSNGG-VAGHVDISGSRASTPEPITITLNSLTSGALRWGVNERAFQSNACESVGGLWDPEMKATAQSSFTDCAAGDLSGKFGPSSS---GTFADSSLRLI---DFLGRSLVLRYSNGTAGDMDVTGSGNLGLSSEALACANVYSSKL

>Sycon.ciliatum_102_CuZnSOD

MPCSAAAYFTNGG-VSGRIIFSGSSSSGG--VTINTTQLNGSGILWHVHQFAFGAPDCAATGGPWDPGSRMANPSAWSVCSLGDLSGKIGPLSS---GQFADGSLGL----DLLGRAVVLHRSDGTAGTMDVTGMPSQNLSPLPVACAAVKPVSR

>Tethya.wilhelma_110_CuZnSOD

RTDKAVAEFDMGG-VRGYVQFFQERPGDPVEINVNLQGLDSDPYPWRIHTYPSADYPCAEVGPVLDPGV---------EGGVGDLFAQHGGLRDMPIQTFVNPHITLCGPESIVGRSLRIDR--GDQGDVRNQGVPETGHEMTILGCSELGPPPP

>Sycon.ciliatum_104_CuZnSOD

KGLDAIAFFAMNG-FHGPVSLYNR-----TTLHVDMRKLN--PLAIDVHRLSFSGSPCESIGDHYNPTN--------PMLALGDLSGSPGGRLNATTRRFAETNLTFTGHHSIIGRSVAFHEGNETAGRIYTNGNNASNHLSKIIACANIAKVHP

>Sycon.ciliatum_103_CuZnSOD

QDERAVAYFNDNG-FRGRVIISK----DAGELSFNASGLQTGQFSLAIHELSASESSCQGIGAVYNPTSAG--------MELGGLSTL-----ALANQVLSTTGLNMINPYGVIGRTLVIQATSGTSGRVSTQGRAASGHQDSLLACARVHRVPH

>P.nodorum_CuZnSOD_3

QSMPASTMFNGTT-VSGYIEGVGSPSG--VRFTVKLQNLPYGPFNWHIHALPVADGNCTATLGHLDPTNRGELIAAPETCQAGDLAGKHGGIAGSFETAFVEKYLTTTGPFSFAGLGFVLHSMNTTGGNMPS-------------ATG-------

>P.nodorum_CuZnSOD_4

AGNPAGATLPGSP-LRGAITAVSAPDGVGVLFSVSFSGLPGGPFMYHLHEKPVENGNCTATGAHLDPYKRGEVPSKPETCQTGDLSGKHGNTSGEFSAEYVDPYSALPGNNAYFGNLFVLHLANKTSGAAPS-------------ATGSVMPTGG

>Drosophila.melanogaster_105_CuZnSOD

QPLTHKTFINSGG-VKGEVTFMQRSKFDPTFLNFTLAPLSEDVAAFRIHSLPPHEDYCLTTGDMHNPRE--NENGTQEQYPVGDLSGKLQGLPSELNGLYWDVFLPLQGRYSIAQRSLVIYTFNRSVGRVQADGSQNTDHRGERLACSAVIGHFR

>Oscarella.carmela_95_CuZnSOD

AHLPALVNIDYNG-IKGVIRINATEGSG--TVAFDFHDLHAFVMTAAIHQLPAYRSKCTDVGLIYDPTDAMSGSSDATKCAVGDLGLRLGKINSVLKTVFPGNNWYLSGSDGIVGRTLVLYNYTGYGGEVYVDGSNTSGHGSTSLSCGNVYNEDL

>Strongylocentrotus.purpuratus_57_CuZnSOD

DTHLAACELRAKHRFRGTVNLRQNRSNGSSDVTIRLFGLSPSGHGVYIREFGDLGDGCQRLGPIFATNR----NPDQQVGSTGLLAVVSPDESGFVQYRTTVGHFDLAGRNSIFGRSIVIEQNQDT---------------NAPLGCCVIGVTRD

>Strongylocentrotus.purpuratus_58_CuZnSOD

ETHLAACELRAKHRFKGTVNLRQNRSSGSSDVTIRLFGLSPSGHGVYIREFGDLGDGCQRLGPIFDTNS----NPDQQIGSTGLLAVVSPDESGFVQSRTTVGHFDLVGRNSIYGRSIVIEQNQDT---------------NAPLGCCVIGVTRD

>Acanthaster.planci_60_CuZnSOD

QAVPACITQNGDMLVVGLLVFKQKKSGGPMEIKASLYGLDDGDHTVDVHEFGDLSNGCKSTGTRYSATY----------SLSGVMGSALRDETSEVRAAWSTTSEGLVGEHSILGRAVVVHAVPHDSGE------------SPKLACCNVSSE--

>Mnemiopsis.leidyi_115_CuZnSOD

SLIQAAVAIIGGNKAKGIVRLLEDENG--LNIEGTVSGLEKGSYSVSINEYGDISNACLSTG---NPVSKAS-----EETPCGILGQVESDGS-------IDTKFSLSGRLSIMGRSLVLQNET------------------KKIACGIVGRSPS

>Caenorhabditis.elegans_113_CuZnSOD

SECPATTFDSGSSTHIGRFTFSQLT-STALRIHGEVYTLPVGRHAVVLHQFGDSSEGCSRVGAPFSSLS----------PSLGDIT------EGKFDRIVEWPVI------DVVGRAVVIYSFSTAFGE-------------KPLACGTIGIAKV

>Caenorhabditis.elegans_115_CuZnSOD

SECPATTFDSGSSTHIGRFTFSQLT-STALRIHGEVYTLPVGRHAVVLHQFGDSSEGCSRVGAPFSSLS----------PSLGDIT------EGKFDRIVEWPVI------DVVGRAVVIYSFSTAFGE-------------KPLACGTIGIAKV

>B.floridae_93_CuZnSOD*

LSVTADSKLAASG-LYGVEVMKESSCTRQIEQDVQINRLDDGTSTHDVH--------------HGVPKGFREQGGNPLRCELGDLSGKQGKAVGGGQRVYTDVDIPLYGELTVVGRSIVIHEANGGGG---------------RLGCGTIRPMTQ

>Ciona.intestinalis_112_CuZnSOD

GSKYAHCMFK----VSGHICFTEK--GGQVEAHVQVSGLDRENYRVHVHEHAPASGSCTTVGGHYNPQNIDE----------GDMLTLKSNTESVLHHPL-NSALSLNGERSIIGRSVAIHVGHKVCPMSGIRGVNEAEHNNRRIALANTKELQD

**Alignment used to construct the phylogenetic tree of MnSOD sequences only**

>Gloeobacter.violaceus.PCC7421.sodB_MnSOD.1

M---------------------AHTLPPLPYDENALAPYVSAQTLSHYGKHHTGYLNNMNKAIA-------GTELESLSLVDLIRTAAKNAE--QKTLFNNAAQVWNHTFYWNSMRPGGGGEPGGTLGEMIKDAFGSYDEFKKQFVTAGTTQFGSGYAWLVKDGE--KLVVTKTPNAETPIT-DESKLPLLNMDVWEHAYYLDYQNLRPDYENAFANLINWEFAEKNLERVFA

>Gloeobacter.kilaueensis.JS1.sodA_MnSOD

M---------------------AFTLPPLPYDESALAPYISAQTLSHYGKHHKGYVDTLNKLVA-------GSEAENTPLEELIKSVHGQPD--KAAIFNNAAQIWNHTFYWNSLKPGGGGEPTGTIAELIKDAFGSYDEFKKQFITAGTTQFGSGYAWLVKDKNSGKLSVIKTPNAETPLT-DPTKVPVLTFDVWEHAYYLDYQNLRPKYEEAVVHLLNWEFAEKNLASA--

>Pseudanabaena.ABRG5-3.sodB_MnSOD

M---------------------AFELPPLPYAQDALAASMSAETLSHYGKHHKAYVDNLNNLIK-------DTDLADKSLEEIIKISY---KEGKAGIFNNAAQVWNHTFYWNGIKPAGGGAPTGALLDAINASFGSLDNFKTEFKNAGATQFGSGWAWLVAEG--GTLKITKTPNAENPLI-HEGQVPLLTMDVWEHAYYLDFQNSRPNFMANFVKLINWDFVAANFAAV--

>Pseudanabaena.PCC7429_MnSOD

M---------------------AFELPSLPYAQDALAASMSAETLSHYGKHHKAYVDNLNNLIK-------DTDLADKPLEEIIKISY---KEGKAGIFNNAAQVWNHTFYWNGIKPAGGGAPTGALLDAINASFGSLDNFKTEFKNAGATQFGSGWAWLVAEG--GTLKITKTPNAENPLI-HEGQVPLLTMDVWEHAYYLDFQNSRPNFMANFVKLINWDFVAANFAAA--

>Pseudanabaena.SR411_MnSOD

M---------------------AFELPSLPYAQDALAASMSAETLSHYGKHHKAYVDNLNNLIK-------DTDLADKSLEEIIKISY---KEGKAGIFNNAAQVWNHTFYWNGIKPAGGGAPTGALLDAINASFGSLDNFKTEFKNAGATQFGSGWAWLVAEG--DKLKITKTPNAENPLV-HEGQVPLLTMDVWEHAYYLDFQNSRPNFMANFVKLINWDFVAANFSAV--

>Capitella.teleta_31_MnSOD

--------------------------------------HISKETLDHYGKHHNTYVVKLNGLVE-------GTELANKSLEEI--------------IKTSEGGIFNHTFYWNCLSPNGGGKPSGELAAAIDKAFGSFDEFVAKFSDMAVNNFGSSWTWLVKNTD-GSLEIVNTSNAATPL--TGDQKPLLTCDLWEHAYYIDYRNVRPDYLKGFWALANWEFVADNFAG---

>Gloeobacter.violaceus.PCC7421.sodB_MnSOD.2

MVLPPSVTMPSATQASTTASGYPYKLAPLPYDYSALEPYIDAETMKHHDKHHQAYVDNLNKALEKY------PDLQKKSPEQLLRDLKQVPEDTRAAVRGNGGGHVNHTMFWEIMKPKGGGEPAGPIAAAIRTNFGSFDAFKTQFNEAGTKRFGSGWVWLVSNPG-GKLEIVSTANQDSPI--ADGKYPIMGNDVWEHAYYLKYQNRRADYLSAWWNTLNWDEINRRFQKAST

>Pseudanabaena.PCC7367_MnSOD

MKRMGLTGSLDHLLAQADTPDDPFTLPPLPYDYNALEPHIDVRTMQHHDRHHAGYVRNLNKAIATY------PDLAGMSAEDMLRDLTQVPEPIRTTVRNNAGGHVNHSMFWEIMSPNGGGTPTGAIAAMIAATFGNFENLQTAFNQAGASRFGSGWAWLVLDKQ-GNLKVTSTANQDSPL--LEGLFPIMGNDVWEHAYYLNYQNRRGDYLKAWWNLVNWEEVNNRFLNAMV

>Nematostella.vectensis_32_MnSOD

M---------------------AFELPKLPYAYDALEPHIDARTMEHHSKHHNGYTTNLNNAIS-------GTDLEGKSIENILINL----DLNNGAVRNNGGGYYNHNLFWTVMSPNGGGNPTGELAAAIDAAFGSFEAFKTEFSKAAATRFGSGWAWLCVHKG-GKLEVCSSANQDNPLMPGCGGTPILALDVWEHAYYLNYQNRRPDYVEAFFNVIDWTEVTRRFATEK-

>Algoriphagus.machipongonensis_SODMn

M---------------------AFELPSLPYAYDALEPNIDAKTMEHHSKHHNGYVTNLNKAVE-------GTDLEGKSLEELLKI-----AGSNTAVRNNGGGHFNHSLFWSILSPDGGGEPTGELADSISAKFGSFAAFKETFNKAAATRFGSGWAWLCIDTK-KELCVCSSPNQDNPLMDVCPGTPILGLDVWEHAYYLNYQNRRPDYINAFWNVIDWDAVSKRYAAAK-

>Chondrus.crispus_1_SODMn

M---------------------SFALPNLPYQYDALEPYVDSTTMNHHTKHHQTYVNNINKVIDG----PSGSALKGLSLPAIQANITSLPAEIQTPVINSGGGHFNHAMFWTLMGRPGNTAPVGSIKDKINADFGSFDEMKAKFNSAAAARFGSGWAWLSVGAD-GKLFISSTKNQENPLMAGQPGSPVLGLDVWEHAYYLKYQNRRPEYISAFWNVVNWDQVTKNYDSVCS

>Pyropia.yezoensis_SODMn

M---------------------AFALPPLPYAYDALEPYIDSTTMNHHTKHHNTYVTNVNNVLAG----ENGGALKGLSLSAIQKEVVTLPDSIKTAVRNSGGGHWNHSFFWSVMGKTGAEAPTGDLKSSIESTFGSLDEMQKKFNTAAASRFGSGWAWLSVNAD-GQLFISSTPNQDNPLMEGQPGTPILGLDVWEHAYYLKYQNRRPEYIASWWKTVDFDVIAKNYSAAKS

>Chondrus.crispus_2_SODMn

MVWANYRSLSQDVASPAARSVSTFSLPDLKYGYASLEPHFDEKTMTHHTRHHQTYVSNLNGVLTG----EHGSEIQDLSLSQIQRKVSSLPEAIRTTVMNHGGGHYNHTLFFSILAPETTSAPVDELKTMVETDFGSFDAMRKEFNAAAMKVFGSGWAWLGVGND-GKLCISNTRNQENPLMEGAPSTPIMGLDIWEHAMYLKYLNRRPEYIDAFWHIIDWEQVAANYSAARD

>Acanthaster.planci_1_MnSOD

MLSVLRRLLASPGTLSTVNSRLEHTLPPLPYDYGALSPVINADIMEHHKKHHATYVNNLNLAEKQLQEAQEAGDIGKM-------------IALQPAVKFNGGGHINHSIFWTVMSPNGGGEPSGELLSAINRDFGSFDNMKQKLSAASIGVQGSGWGWLGYNKTTRTLAIATCANQD-PLEPTTGLVPLFGIDVWEHAYYLQYKNVRPDYVKAIFNVANWANIEERFQKAMA

>Branchiostoma.floridae_2_MnSOD

MLALTQRVASHPGLLAAAGTRLKHTLPDLAYDYGALEPTISAEIMQHHSKHHATYVNNLNVAEEKLAEAQAKGDVTTE-------------IALGPALKFNGGGHLNHSIFWTNLSPNGGGEPQGEVLEAINRDFGSFENLKKKMSAASVAVQGSGWGWLGYDKENNRLSIAACANQD-PLQATTGLIPLLGIDVWEHAYYLQYKNVRPDYVNAIWNVVSWENINERFLAAKK

>Danio.rerio_3_MnSOD

MLCYVRCAATFNPLLGAVTSRQKHALPDLTYDYGALEPHICAEIMQHHSKHHATYVNNLNVTEEKYQEALAKGDVTTQ-------------VSLQPALKFNGGGHINHTIFWTNLSPNGGGEPQGELLEAIKRDFGSFQKMKEKISAATVAVQGSGWGWLGFEKESGRLRIAACANQD-PLQGTTGLIPLLGIDVWEHAYYLQYKNVRPDYVKAIWNVVNWENVSERFQAAKK

>Homo.sapiens_4_MnSOD

MLSAVCGTSRLAPVLGYLGSRQKHSLPDLPYDYGALEPHINAQIMQHHSKHHAAYVNNLNVTEEKYQEALA----------------------------------------------------KGELLEAIKRDFGSFDKFKEKLTAASVGVQGSGWGWLGFNKERGHLQIAACPNQD-PLQGTTGLIPLLGIDVWEHAYYLQYKNVRPDYLKAIWNVINWENVTERYMACKK

>Homo.sapiens_5_MnSOD

MLSAVCGTSRLAPVLGYLGSRQKHSLPDLPYDYGALEPHINAQIMQHHSKHHAAYVNNLNVTEEKYQEALA----------------------------------------------------KGELLEAIKRDFGSFDKFKEKLTAASVGVQGSGWGWLGFNKERGHLQIAACPNQD-PLQGTTGLIPLLGIDVWEHAYYLQYKNVRPDYLKAIWNVINWENVTERYMACKK

>Homo.sapiens_6_MnSOD

--------------------------------------------MQHHSKHHAAYVNNLNVTEEKYQEALA----------------------------------------------------KGELLEAIKRDFGSFDKFKEKLTAASVGVQGSG-----------------------------------------------------------------------------

>Homo.sapiens_7_MnSOD

MLSAVCGTSRLAPVLGYLGSRQKHSLPDLPYDYGALEPHINAQIMQHHSKHHAAYVNNLNVTEEKYQEALAKGDVTAQ-------------IALQPALKFNGGGHINHSIFWTNLSPNGGGEPKGELLEAIKRDFGSFDKFKEKLTAASVGVQGSGWGWLGFNKERGHLQIAACPNQD-PLQGTTGLIPLLGIDVWEHAYYLQYKNVRPDYLKAIWNVINWENVTERYMACKK

>Homo.sapiens_8_MnSOD

MLSAVCGTSRLAPVLGYLGSRQKHSLPDLPYDYGALEPHINAQIMQHHSKHHAAYVNNLNVTEEKYQEALAKGDVTAQ-------------IALQPALKFNGGGHINHSIFWTNLSPNGGGEPKGELLEAIKRDFGSFDKFKEKLTAASVGVQGSGWGWLGFNKERGHLQIAACPNQD-PLQGTTGLIPLLGIDVWEHAYYLQYKNVRPDYLKAIWNVINWENVTERYMACKK

>Homo.sapiens_9_MnSOD

--------------------------------------------MQHHSKHHAAYVNNLNVTEEKYQEALAKGDVTAQ-------------IALQPALKFNGGGHINHSIFWTNLSPNGGGEPKGELLEAIKRDFGSFDKFKEKLTAASVGVQGSGWGWLGFNKERG------------------------------------------------------------------

>Homo.sapiens_10_MnSOD

--------------------------------------------MQHHSKHHAAYVNNLNVTEEKYQEALAKGDVTAQ-------------IALQPALKFNGGGHINHSIFWTNLSPNGGGEPKGELLEAIKRDFGSFDKFKEKLTAASVGVQGSGWGWLGFNKERGHLQIAACPNQD-PLQGTTGLIPLLGIDVWEHAYYLQYKNVRPDYLKAIWNVINWENVTERYMACKK

>Homo.sapiens_11_MnSOD

AFHRVAFTSRLAPVLGYLGSRQKHSLPDLPYDYGALEPHINAQIMQHHSKHHAAYVNNLNVTEEKYQEALAKGDVTAQ-------------IALQPALKFNGGGHINHSIFWTNLSPNGGGEPKGELLEAIKRDFGSFDKFKEKLTAASVGVQGSGWGWLGFNKERGHLQIAACPNQ--------------------------------------------------------

>Homo.sapiens_12_MnSOD

MLPGATSTSRLAPVLGYLGSRQKHSLPDLPYDYGALEPHINAQIMQHHSKHHAAYVNNLNVTEEKYQEALAKGDVTAQ-------------IALQPALKFNGGGHINHSIFWTNLSPNGGGEPKGELLEAIKRDFGSFDKFKEKLTAASVGVQGSGWGWLGFNKERGHLQ---------------------------------------------------------------

>Homo.sapiens_14_MnSOD

MLSAVCGTSRLAPVLGYLGSRQKHSLPDLPYDYGALEPHINAQIMQHHSKHHAAYVNNLNVTEEKYQEALAKGDVTAQ-------------IALQPALKFNGGGHINHSIFWTNLSPNGGGEPK-------------------------------------------------------------GLIPLLGIDVWEHAYYLQYKNVRPDYLKAIWNVINWENVTERYMACKK

>Xenopus.tropicalis_13_MnSOD

MLCLVCGRGRCAPALTYFTSREKHTLPDLPYDYGALQPHISAEIMQHHSKHHATYVNNLNITEEKYAEALAKGDVTTQ-------------VSLQAALKFNGGGHINHTIFWTNLSPNGGGEPQGELLDAIKRDFGSFEKFKEKLSTVSVGVQGSGWGWLGYNKESNRLQLAACANQD-PLQGTTGLIPLLGIDVWEHAYYLQYKNVRPDYMKAIWNVINWENVAERYRASKK

>Nematostella.vectensis_15_MnSOD

MLLRVARLSSRKAVLPVALVRAKHTLPDLPYDYDALEPTINTEIMRHHSKHHATYVNNLNIAEEKCLEAQAKGDVATA-------------IALQPAVKFNGGGHLNHSIFWTNLSPNGGGEPTGELMEAIKRDFGSFENFKERFNAATIAVQGSGWGWLGYDKVNKRLAIATCFNQD-PLQPTTGLVPLLGIDVWEHAYYLQYKNVRPDYVKAIYDVINWTNVAERLQAASS

>Capitella.teleta_16_MnSOD

MLSA-ANSLKHAVPRCAVSSRLKHTLPDLPYDYNALEPTISADIMQHHSKHHATYVNNLNVAEEKLAEARATGDVSTE-------------IALGSALIFNGGGHINHSIFWQNLSPNGGGEPTGELMSAIQRDFGSFENMKKTLSASTVAVQGSGWGWLGYNKAAGKLQIATCANQD-PLEATKGLVPMFGIDVWEHAYYLQYKNVRPDYVNAIWNVANWADITERFNKARQ

>Oscarella.carmela_17_MnSOD

MSQALLSRLGVRSLGGFLCCRSKHTLPDLPYDYGALEPAISAEIMQHHSKHHATYVNNLNGAEEKLAEALATGDTASQ-------------IALQPALKFNGGGHINHSIFWTNMCPGGSGEPQGDLMNAIKRDFGSSEKLKTSLSAASVAVQGSGWGWLGYNKIRKRLEVATCANQD-PLEATTGLVPLLGIDVWEHAYYLQYKNVRPDYVKAIWEVVNWPNVAERFAAAQG

>Sycon.ciliatum_18_MnSOD

MALALCSAARCAASLASACAAQKHTLPDLPYDYGALAPHISPEIMQHHSKHHQTYVNNLNVAEEKLQECAAKGDASGV-------------IALGGALKFNGGGHINHSIFWQNLSPNGGGEPTGELNNAIVTQFGSFDAFKQKMTTAAVGVQGSGWAWLGLNKETMQLSVSACPNQD-PLQSTTGLVPLLGIDVWEHAYYLQYKNVRPDYLKAIWNVINWEDVASRYTGAQ-

>Amphimedon.queenslandica_20_MnSOD

MLSLLFGGKNSLSSLSSISRRHKHVLPELPYGYKALEPVISGDIMEHHTKHHATYVNNLNATEGKMKECLEAGDVSGA-------------VALEGAYRFNGGGHINHSIFWNNLSPNGGGTPQGKLMEAIERDFGSFDEFKSQLTARTVAIQGSGWGWLGFNQVTGRLQIATCPNQD-PLQATTGLVPLLGIDVWEHAYYLQYRNVRPDYVKAIWDVINWDDVSKRLP----

>Xestospongia.bergquistia_19_MnSOD

MLSILRAPFKGTGTALTSLSRQNHSLPELPYRYNALEPVISGEIMEHHKKHHATYVNNLNTAEEQLKQCVDEGNVGGV-------------IALQPAIKFNGGGHINHSIFWNNLSPNGGGEPQGDFRAAIERDFSSFDNFKSQLSAKTIAIQGSGWGWLGYNKESGRLQIATCANQD-PLQGTTGLIPLLGIDVWEHAYYLQYKNVRPDYVKAIWDVINWQDVTERYSNAMT

>Tethya.wilhelma_24_MnSOD

MLSQLLRHSRQATCVAATQTRNKFTLPELPYKYNALEPVISGEIMEHHSKHHATYVNNLNAAEEKLKQAIEDGNIKGI-------------LDTQGAIKFNGGGHLNHSIFWTHLSPNGGGKPTGDLAAAIDRDFGSFDNLMDQMTTKTVAIQGSGWGWLGYSKSNGRLVISTTANQD-PLQATTGLIPLLGIDVWEHAYYLQYKNVRPEYVKAIWKVMDWKDIESKFSAAKS

>Ciona.intestinalis_21_MnSOD

ML-RLLSSRCTSKVVPVWASRGKHTLPDLPYDYSALEPHISAEIMEHYAKHHATYVNNLNIAEEKLHEAEAKNDISSI-------------ISLGPALKFNGGGHINHSIFWETLSPNGGSEPCGELKTAIDRDFGSFENLKAKLTAASVGVQGSGWSWLGLDKEKGQLQVVACPNQD-PLHATTGLVPLFGIDVWEHAYYLQYKNVRPDYIKAIFNVVNWENVGKRFTDA--

>Mnemiopsis.leidyi_22_MnSOD

ML------QRLLKLSRLAQARCKYTLPELPYNYEALEPVINREIMEHHSKHHATYVNNLNATAEKVSAAVAEGNAGAV-------------IGLHGALKFNGGGHINHSIFWNNLSPNGGGVPSGELLSAIEAEFGSFDQFKAKMSAATIAVQGSGWGWLGLNPDSKRLQIATCANQD-PLQATTGLAPLLGIDVWEHAYYLQYKNVRPEYVNSIWDIVNWEDVAARYAAASS

>Strongylocentrotus.purpuratus_23_MnSOD

IFVAPGRALLRSQARVVSGVSSLHTLPELPYDYNALSPVISTEIMEHHKKHHNTYVTNLNVAEEALEHAVEAGDISTI-------------ISLQAALRFNGGGHINHTIFWQNLSPDGGGEPTGELLNVIKKDFGSYDDMRSKLSAATVAIQGSGWGWLGYNKGTRSLQIATCPNQD-PLKATTGLIPLFGIDVWEHAYYLQYKNVRPDYVKAIFNIANWSDVEKRLTDAMT

>Monosiga.brevicollis_SODMn

ML------ARAMRSATTGARRMKHTLPDLAYDYAALEPVISAKIMEHHSKHHNTYVNNLNIAEEQYAEAVHTGDLTKA-------------IGLQSAIKFNGGGHINHSIFWTNLAPQGGGEQDGELKTAIEEAFGSVETMQQKLNAMTAAVQGSGWGWLGYNKASKQLQLATCANQD-PLETTHGLVPLFGIDVWEHAYYLDYKNVRPDYLKAVWEIANWQNVEERYQAAKL

>Caenorhabditis.elegans_25_MnSOD

MLQVRCVSKLVQPITGVAAVRSKHSLPDLPYDYADLEPVISHEIMQHHQKHHATYVNNLNQIEEKLHEAVSKGNVKEA-------------IALQPALKFNGGGHINHSIFWTNLAKD-GGEPSAELLTAIKSDFGSLDNLQKQLSASTVAVQGSGWGWLGYCPKGKILKVATCANQD-PLEATTGLVPLFGIDVWEHAYYLQYKNVRPDYVNAIWKIANWKNVSERFAKAQQ

>Caenorhabditis.elegans_26_MnSOD

MLQVRCVSKLVQPITGVAAVRSKHSLPDLPYDYADLEPVISHEIMQHHQKHHATYVNNLNQIEEKLHEAVSKGNVKEA-------------IALQPALKFNGGGHINHSIFWTNLAKD-GGEPSAELLTAIKSDFGSLDNLQKQLSASTVAVQGSGWGWLGYCPKGKILKVATCANQD-PLEATTGLVPLFGIDVWEHAYYLQYKNVRPDYVNAIWKIANWKNVSERFAKAQQ

>Caenorhabditis.elegans_27_MnSOD

MLQVRCVSKLVQPITGVAAVRSKHSLPDLPYDYADLEPVISHEIMQHHQKHHATYVNNLNQIEEKLHEAVSKGNVKEA-------------IALQPALKFNGGGHINHSIFWTNLAKD-GGEPSAELLTAIKSDFGSLDNLQKQLSASTVAVQGSGWGWLGYCPKGKILKVATCANQD-PLEATTGLVPLFGIDVWEHAYYLQYKNVRPDYVNAIWKIANWKNVSERFAKAQQ

>Caenorhabditis.elegans_28_MnSOD

MLQARTASKLVQPVAGVLAVRSKHTLPDLPFDYADLEPVISHEIMQHHQKHHATYVNNLNQIEEKLHEAVSKGNLKEA-------------IALQPALKFNGGGHINHSIFWTNLAKD-GGEPSKELMDTIKRDFGSLDNLQKRLSDITIAVQGSGWGWLGYCKKDKILKIATCANQD-PLE---GMVPLFGIDVWEHAYYLQYKNVRPDYVHAIWKIANWKNISERFANARQ

>Trichoplax.adhaerens_1_SODMn

MLARQLGRNSCHSALLITQARRKHDLPPLPYAYNALEPTISAEIMEHHSKHHQTYVTNLNAAEEKLAEATSKNDISGV-------------ITLQGALRFNGGGHINHSIFWKNLSNDGGGLPTGELGDAINACFGSFDNFKSKLSAATIAIQGSGWGWLGYCKESNSLKIATCANQD-PLQATTGYVPLLGIDVWEHAYYLQYKNVRPNYVNAIFDVINWNDVANNFRNAKA

>Lingula.anatina_29_MnSOD

MLCLKRFVPQQKAALGFTASRLAHSLPDLPYDFNALEPVIGAEIMEHYKKHHATYVNNLNIAEEKMEKALAEGDTNAA-------------ISLLPALNFNGGGHINHSIFWTNLSPNGGGEPTGDLKESINRDFGGFESMKKELISKTVAIQGSGWGWLGYNPHTQRLRLAVRPNQD-SLFPTLGLIPLLGIDVWEHAYYLQYKNDRGAYVNAIWNIINWDNVADRYANARM

>Drosophila.melanogaster_30a_MnSOD

MF-----VARKISQTASLAVRGKHTLPKLPYDYAALEPIICREIMEHHQKHHQTYVNNLNAAEEQLEEAKSKSDTTKL-------------IQLAPALRFNGGGHINHTIFWQNLSPN-KTQPSDDLKKAIESQWKSLEEFKKELTTLTVAVQGSGWGWLGFNKKSGKLQLAALPNQD-PLEASTGLIPLFGIDVWEHAYYLQYKNVRPSYVEAIWDIANWDDISCRFQEAKK

>Drosophila.melanogaster_30b_MnSOD

MF-----VARKISQTASLAVRGKHTLPKLPYDYAALEPIICREIMEHHQKHHQTYVNNLNAAEEQLEEAKSKSDTTKL-------------IQLAPALRFNGGGHINHTIFWQNLSPN-KTQPSDDLKKAIESQWKSLEEFKKELTTLTVAVQGSGWGWLGFNKKSGKLQLAALPNQD-PLEASTGLIPLFGIDVWEHAYYLQYKNVRPSYVEAIWDIANWDDISCRFQEAKK

>Dictyostelium.discoideum_SODMn

MLPLIKKVGESNGLRNFGSQSNSYTLPDLPYDYGALSPVISPEIMTHHKKHHQTYVNNLNIALDKLSSASSAKDVAQM-------------IALQSAIKFNGGGHVNHSIFWTNLAPDGGVAPSGPLADAINKQYGSIEKLIEKMSAETTAIQGSGWGWLGYDKANDRLVIQTQQNQD-PL-SVSGYVPLLGIDVWEHAYYLDYKNVRADYVKNIWQIVNWKNVAERYNTAKK

>Arabidopsis.thaliana_SODMn

MAIASRTLAGLKETSSRIRGIQTFTLPDLPYDYGALEPAISGEIMQHHQKHHQAYVTNYNNALEQLDQAVNKGDASTV-------------VKLQSAIKFNGGGHVNHSIFWKNLAPEGGGEPKGSLGSAIDAHFGSLEGLVKKMSAEGAAVQGSGWVWLGLDKELKKLVVDTTANQD-PLVTKGGLVPLVGIDVWEHAYYLQYKNVRPEYLKNVWKVINWKYASEVYEKENN

>Podospora_anserina_1_SODMn

M------------------AVQEYTLPALPYAYNALEPHISAQIMEHHSKHHQAYVTNLNNALRLHVAAVGAGDIASQ-------------IEMQQVIKFNGGGHINHSLFWKNLAPETKPEAAKELVAAVEKTWGSLDDFKNAFSSTLLGIQGSGWGWLVKDSA-NGLRIVTTKDQD-PV--VGRDVPVFGVDMWEHAYYLQYLNGKAAYVENIWNVINWKTAEERFLGTSE

>Podospora.anserina_2_SODMn

---------------------------------------------------------NLNNALRLHVAAVGAGDIASQ-------------IEMQQVIKFNGGGHINHSLFWKNLAPETKPEAAKELVAAVEKTWGSLDDFKNAFSSTLLGIQGSGWGWLVKDSA-NGLRIVTTKDQD-PV--VGRDVPVFGVD---------------------------------------

>Amphimedon.queenslandica_33_MnSOD

MEFFFTTLSQSSIYDEMFYPVDEYSFPQLPYDYHELEPYIDQRTLTHHKKHHQGYTVKMNQALKDWRKQEPQSDLAKSSIIDILQNLEMVPDKWRTTLQNNAGGYVNHIYYWVTMCPKPGEIS-KALLKKIKASFAGMSEFKESFTTASLSLFGSGYVWLVTDDE-GSISIISTKNQDCPI--SSNLYPLLVLDVWEHSYYLKHQNLRADYISDWWNVVCWQNVETLRQFWIN

>Amphimedon.queenslandica_34_MnSOD

----------------MFHPADEYSFPQLPYDYHELGPYIDQRTLTHHKKHHQGYTVKMNQALKDWRKQEPQNDLARSSIINILQNLERVPDKWRTTLQNNAGGYVNHIYYWVTMCPKPGDIP-KALLKKIEASFAGMSEFKNSFTTASLSLFGSGYVWLVTDDE-GSISIISTKNQ--VL--YCHAFILIMLYMYHYKRF-----LRELYFTRCTKFLIWRF----------

>Ephydatia.muelleri_35_MnSOD

M-LCFVACNSEQIYEDLVVYHDELKLPDLPYDYNALEPYIDEATMKHHLGHHKTYGDNTNKALKEWRKGDPTSPLARSSIVQILQKITEVPETWQVAIRNNGGGFVNHVIYWATMCPGGGGSPSGELLMQISRSFGSYVAFQEHFTSAASKLFGSGYVWLCENAK-GDLVITSSKNQDSPL--SDGLYPLLVMDIWEHAYYLKHQNKRAGYIQSWWNIVCWDEVPKLQHFWHN

>Tethya.wilhelma_36_MnSOD

MKILSLHCNSGQAYEQLTHPATDYPLPDLPYSYDELEPLIDTHTLKHHLGHHKAYTDKMNAALNQWKHEEPTSKLAQSSIITILQSILSVPEKWQTAIRNNGGGYVNHIFYWVTMCPHGHDQPGGKLLEAIINTFGGFEEFKSEFSQTAAKLFGSGYVWLCESQA-GDLSIVTTYNQDSPL--SDGLYPLLVLDVWEHAYYLKHQNKRTEYIRDWWSVVCWDHVADLEQFWMN

>Xestospongia.bergquistia_47_MnSOD

MTYTSKFVDEATIYDRIFQKADKYELPNLPYQYNDLEPYLDERTLIHHQGHHKAYTDKMNAALQEWREKYSHDL-----------ELDQIPEKWQTALKNNGGGYVNHIFYWATMCPIPKAPP-PTLTAAIEENFGGISYFFDNFTSSAKSLFGSGYVWLVQDKE-DNLYIIQTKDQVNPV--SKGMNKSQITAIEEHQNYVPFQNDSLQVSKQWLNQIDFDSVNEYSSFWSN

>Xestospongia.bergquistia_48_MnSOD

MVYLSRQVDEATIYDRIFQKADKYELPNLPYQYNDLEPYLDERTLIHHQGHHKAYTDKMNAALQEWREKMPHDTLATLSIFDILKRLDQIPEKWQTALKNNGGGYVNHIFYWATMCPIPKAPP-PTLTAAIEENFGGISYFFDNFTSSAKSLFGSGYVWLVQDKE-DNLYIIQTKDQ--------------------------------------------------------

>Branchiostoma.floridae_37_MnSOD

MACLSNTVSVDTPYKGIQLFADEYPVPPLPYEYDALEPYIDERTARHHLGHHAGYTKKMNAALKAWRDEVPGVGLQSNSILHILENLNQVPDKHRTAIRNNGGGFVNHAIYWATMSPRREGEPEATLATEIIANFQNFSNFQEQFTNTAKTLFGSGYVWLSRNPREQTLIIHTTANQDSPI--SDKLRPILVIDIWEHAYYLKHQNKRPDHIQDWWALVDWGQVKALSDWWAA

>Lingula.anatina_38_MnSOD

--VVSSRFVASWPYDDSALSKETYLLPALPYGYGALEPHMDEAT--HHQGHHAAYTAKMNAALKQWRR----------------------------------------------------------------------------------------YVWLSRDQD-GKLIISTTANQDSPV--TERLHPILVIDIWEHAYYLKHQNKRANFVEAWWNLVDWRAVETLDTWWKG

>Lingula.anatina_39_MnSOD

MAVVTPQTADARMYEDCVQQREDYPLPPLPYSYNSLEPHMDATTVQHHLKHHAAYTKKMNAALKQWRDEDPENTLATGSIVEILQHLYNVPEKYRTVLRNNGGGYVNHAIYFACMSSRGA-AISQDLENDIIKDFGSYNNFTKQFTTKAMGLFGSGYVWLSRGGN-DKLVISTTANQDSPV--TESFRPILVIDIWEHAYYLKYQYRRADFVAAWWKLVDWKAVVDLDKWWRV

>Lingula.anatina_40_MnSOD

MAVVTPQTAAIGMYEESAVPKEAYPLPPLPYPYGGLEPHMDERTVQHHQGHHAAYTTKMNAALKQWREEDPGNTFAKSSILNILQNVSSVPEKYRTALRNNGGGYFNHAIYWACMSPGGA-AITPDLKSDIIQNFGTYDNFKDQFTTKAMALFGSGYVWLSRGEN-EQLVISTTANQDSPV--TEDLHPILVIDIWEHAYYLKHQNKRADFVADWWKLVNWNAVRDLDQWWKR

>Lingula.anatina_41_MnSOD

MAVVTPQTAAAAMYEDSVVLREDYLLPPLPYAYNSLEPHMDEKTVEHHLEHHAAFTTKMNAVLKQWRDEDPENTFAKSSILNILENLYDVPEKYRTALRNNGGGYFNHAIYWACMSPRGAVTINPDLENDIKKDFGSYNNFTNQFTTKAMALFGSGYVWLSRGKN-EQLVISTTANEDSPV--TEDLRPILVIDIWEHAYYLKHQNKRADFVEAWWNLVNWDAVWDLDQWWKP

>Lingula.anatina_42_MnSOD

--AVSSRFVASWPYDDSGFSKDTYLLPALPYGYGALEPHMDEATVKHHQGHHAAYTAKMNAALKQWRSEAAGDKFANSSILNILQKLSHVPEKYRMTLRNNGGGYFNHAIYWACMSPKGA-RISPGLEKDIKDNFGTYENFKEQFTAKSLSLFGSGYVWLSRDQD-GKLIISTTANQDSPV--TERLHPILVIDIW-------------------------------------

>Strongylocentrotus.purpuratus_45_MnSOD

MAMMCTSYCVSYPFECIAEIRSEYILPDLPFGYDDLEPYIDSATLNHHRGHHAGYTRKLNAALHQWGSEEP-DSYAATSILDLLQHLDDIPPKYKQSIRNNGGGFVNHNIYWSTMASNKENVPSGDLLGEINESFGSFEEFKNQFTGRALTLFGSGYVWLSREEEPASLIISTTANQDTPI--SNGLQPILVLDVWEHAYYLKHQNKRANHIEDWWRVVDWSAVANLRDWWSD

>Nematostella.vectensis_43_MnSOD

M--------------------EKYTLPELPYDYNELEPHIDEATLRHHLGHHAAYTKKLNAALK--------------DIVEILRNNEQIPDKWRTDVINNGGGFVNHALYWATMSPNPKSEPTGKIGDLIDKSHGNFSMFKQWFDEQVNSMFGSGYTWLCQDVTSGFLTILNMGNQESPV--AYRLNPVLVIDLWEHAFYLKHQNKRPGYVHSWWHLVDWERVNELLEWWQN

>Oscarella.carmela_44_MnSOD

MMFAWSALDIPDLKIEKPEDGADYKLPTLDFDYNALEPYIDEKTVRHHLGHHAGYTHKMNAILRKWAEKKEQQDLLSLPIVNILEQVNDIPLDIREDLQNHGGGFINHAWYWATIAPSRESEPVGSVAGAIEKRYGDFKKFQQEFNHTAFTFFGSGYVWLCEVPPTGILQITTFPNQMSPL--GFGLKPILVLDVWEHAYYLKHQNKRAGYVHDWWEIIDWNKVELISQAWKN

>Capitella.teleta_46_MnSOD

MSLCHLGLNGESPFTTIASKAELYSLPALTFGYADLEPFFDEATLHHYDGHHETYRKKMNSALSEWRESDPLDSFSTEPILEILKNLNKVPENFRNAIKNSGGGFVNHALYWACMSPNPTREPTGALLQDIENEFGSFVKFSLKFTDRAVSLFGSGYVWLSRNPSSGALIITTTVNQDSPI--SDGLHPILVIDVWEHSYYLKHQFRRHVYVADWWKVVDWENVEELDKWWRG

>Oscarella.carmela_49_MnSOD

MAFV--------SYLKIKDSAEGYLLPEVGYDYADLEPHIDEATMKHHSRHHAGYTRKMNAALDAWRKSGEVPELAAKPIENIIQHVDDVPKKWQRAIRNNGGGFINHAFYWQNMSPNAQREPSGPLLEKIKSNFGSFEALKQKITETAAGLFGSGWVWLVEVPGSGELQLLSSENQDNPM--SQGLKPILALDVWEHAYYLKHKNDRPSYIQSWWNIADWRKVGDILAAWNS

>Sycon.ciliatum_50_MnSOD

MNHFARHADASSELLDIRLENGSYELPRLMYSYEALEPYMSERTVRHHTGHHAAYTKKMNSILSRMPSAD---AVVASNPLQQLMNLDALPEDVRDAFRNQAGGFVNHAFTFFVMGPVADQGPSGKILELITESFGSTDAMKEQFSGAAAKLFGSGYVWLCLVAGVRRLTITENANQDSPL--SGGFRPVLGIDLWEHAYYLQYQNKRVDYIATWWQLVSWERVGLLLDAWIP

>Trichoplax.adhaerens_2_SODMn

MI---------------------KALP-LPYDKNELEPTLSKNTFDHYDKHYLGYANKLSALVDKYYNML---------LADIIIES----YKNNDTPIYNNAAQVNHIFYWKSIGKT-QKCPT-KLIELLNRDFGSYDTFIEKFIQAGVTLFGSGWIWLVQDKESKKLSILQTKDADNPL--ILDKTPILTIDVWEHAYYIDYKNDRLKYLDQIKNNIKWLAVSNLN-----

>Trichoplax.adhaerens_3_SODMn

--------------------------------------------------------------------------------EDIVKKS----YISKDTKIFNNAAQVNHEFYWDSIRKKTQNEPTYDLFNKINECFGSFEEFCKKFVEAGVALFGSGWIWLVQDPDTKKLEIMQTYNADSPM--LHNKIAILTIDVWEHAYYIDYKNDRLSYLENVKFHLNWDAKNNMKL----

**Alignment used to construct the phylogenetic tree of NOX sequences**

>D.discoideum_NOXB

-TYIMLFYIALNIGVGVF-FLGLSFCFSRTAARLINLNSAVILLPVLRNFLSWLRGTIVIP---IDLFHKLCAFMLFCCTIIHCVGHYISFI-LFFSVPGITGHIMLLILILIVSSSMWRIRRFEIFWYVHHLIPFILLCFHGYSKILS--WWIIAPFILYSIERLIRIARVILAIMHPSKVLMRNDNFPGQLLCPSIEWHPFTITSAPDDPFISVHINIVGWTLLS-TILKIDGPFGAPAENFFRNLVLIGAGIGVTPFSSILLKNQLKINKIFIWISRQKNSFQWFTDILAELEI--LEIHIFLTALELDDYAKI----------KKCHITNLHSKTFGRPNFIFLIGVFCGNKALGKIIKNCNCLIFENF

>D.discoideum_NOXA

-LILVILYTLGNIAAFVF-VVGYGVCFARGCAQLLKLNCALILVPVLRNLLSFLRGTFLVP---FDIFHKLIAWVICFATFGHVMAHFNNFY--AFTLAGWTGHVVCIVMVLMYTSAVESIRRFEGFWYTHHLVVFGLLVVHGLHSILSF-WWVIGPCALYIVERLIRLLRTMLARIHPSRVVM-TERFPGQLLCPTIEWHPFTITSAPEEDFVSCHINVVGWTLLS-PILRIDGPFGAASEEVFKQVILVGAGIGVTPFASILIKYQL-IDKVFYWICRDRNSFEWFSGLIGELE--FLEIHPYLTALSAQEIRDVM--------YGKDLITGFTTPTFGRPKWIFHVGVFCGPKLLSKLYKASTCFHYENF

>M.brevicollis_2

-YTFVLLWMCANVGLFFFLIVHFGLPVARGAASVLNLNCALVLLPVCRNLVNFCRGIFESKRRLFDIFHKWCAYVICVFASIHICAHFFNVLPVLFTVAGGTGVGITVPLILMVTTASQQIRRFELFWYTHHLVVFVCLCLHGYSGFVALTWWVIGPLILYILERLYRWYKLRIVVKHKDSVPI-FQKVAGQVICPKIEWHPITLTSCPELDYVSVHIRLVGWTLAL-PYICIDGPFGTASEDMYPVAMLIGAGIGVTPFASLLLYFRFKTQKVFYWMCPGFDAWGWFASLLIDLELEFLEIRVFTTGWSQDDAAKIML-------QEGDAETGLRHKMFGRPNWEFVIGLFCGPKVLSSLHVTCGTFYYENF

>A.queenslandica_6_NOX2

---WLGTWFLLNIVLFIFMYLKEALAFARGPALPINFNVILILLPVCRNIISVIRGCTVVPRRVLDLFHKAIAWMLVVSSTMHVVAHYYNYL-ACFTYAGVTGHIITLVLFLMITSSVTYIRRFEVFWYTHQLIVFVGLGIHQYGRLLLFSWFLLCGLLIYGLERILRVVRVVIVVQHPSNTIM-RKGFAGQVVCPSVEWHPFTLTSAPEEDYFSVHIRILGWTIKL-PSIYIDGPFGTASEDAFPVALLVGAGIGVTPFASVLLYYRLVLKKLFVWICPETYAFEWFANLFQELELIFLTSWIYLTGWKDNQAFYLML-------NEGDAITGLRAETFGRPEWIFVVGVFCGPSVLSHLHKQCGAFYYENF

>X.bergquistia_1_NOX2

----LGTWFLLNIALFIFIYIREALAFARGPALPINFNVILILLPVCRNIISVIRGCTVVPRRLLDLFHKAIAWMLVVSSAMHIMAHYYNYL-ACFTYAGVTGHIITLALFLMVTSSLEFIRRFEVFWYTHHLLIFIGLGIHQYGRLLLFSWYLLCGLLIYGLERILRVVRVVIVVQHPSNTIM-RKGFAGQVLCPSVEWHPFTLTSAPEEDYFSVHIRIVGWTLIL-PSMYVDGPFGTASEDVFSVGLLVGAGIGVTPFASVLLYYRLVLKKLFVWICPETHSFEWFANLFQELELHFLTSWIYLTGWRDNQAFYLML-------NEGDAITGLRANTFGRPEWIFLVGVFCGPSVLSHLHGYCGAFYYENF

>E.muelleri_1_NOX2

LAIVFGSWICMNIALFIFSVLREALSFARGPAIIINFNIILILLPVCRNLISLLRGTGCVPRRVLDLFHKAVAWMIIAASAMHIMAHYYNYI-VCLTVAGLTGHVITVALFLMVTSSLEFMRRFEIFWYTHHLVIFLGLAFHQFQQLLAFSWFMLGGLTIYIIERVIRFIRVVLVVQHPSKTFM-KKGFAGQVIVPSVEWHPFTLTSAPEEDYFSAHIRVVGWTVQL-PIVAIDGPFGTASEDVFEVGMLVGAGIGVTPFASILVFYKTALKKVFYWICPEPQSFEWFASMLASVELSFLDIHIYLSGWKDKDAFAVYL-------REKDAVTGLQARTYGRPDWIFIIGVFCGPPVLSHLHEKCRAFFYENF

>O.carmela_2_NOX2

-WLFMFLWLGANIGLYFHTILGSALAVARGSAACLNFNCMLILIPVCRNLISMMRGSCCTPRRILDLFHKLVAWAIVFFTALHTGSHMFNYYGVMFTVAGITGHVIIIVLFLMVTSSIEVMRRFEVFWYTHHLVIFGCLIAHGVQGLVAVTWWVVGPLFLYFLERIVRFYRVVVVVEHPSKVIL-KRGFAGQVVAPEVEWHPFTLTSSPEEDYFSVHIRLMGWTMRM-PKILIDGPFGTPSEDIFEVGIGIGAGIGVTPFASILLWYQMKLKKVFFWICPDTGAFEWFQSLLQHLEMLFLEYHIYLTGWDPKMAREIYM-------KEGDIITGLQQKTYGRPHWIFLMGS-CPACTRNAVSDCIQMLTIKTF

>S.ciliatum_1_NOX2

-YLVLILWIGMQAYFFVCLVIGKGLCIARGSAANLNFNSMLILLPVCRNLISFLRGSNGVRRRLLDIFHKCIAWSVVLWTAIHTGSHFWNYIPVMWTYAGISGHLIVVALFLMVTSSMEIIRRFEVFWYTHHLIIFVGAVVHGLQGLIAFTWWVVGPLVLYLVERIIRFIRVVIVVQHPSKTIM-MKGFAGQIMVPEIEWHPFTLTSSPEENYFSLHIRIAGWTLRM-PTIRIDGPFGTPSEDIFRTGVCIGAGIGVTPFASILIWYQLKLKKVLFWICPDTNSFEWFADLLKSLEMIFLDYNIYLTGWGTDEAKNIML-------HEQDPITGLQQKTYGRPQWIFMLGVFCGPKILSTLHMMCETFFYENF

>S.ciliatum_3_NOX2

-YFVLFIWIAVNCFMFGCLVLKRGLCLARGSAACLNLNCMLILIPVCRNLISLVRGSGSVRRRLLDIFHKIVAWSIVFFSAIHTGSHFWNYIPVMWTWAGFTGHIILVALFLMVTSSIEIIRRFEVFWYTHHLVVFACLIIHGFQGLLAFTWWVVGPLALYLLERLIRFYRVVIVVQHPSKTLM-KKGFAGQIMVPEVEWHPFTLTSSPEERYFSVHIRVVGWTLRM-PTVNIDGPFGTASEDIFRVGVCIGAGIGVTPFASILIWYQLKLQKVVYWICPDTQAFEWFADLLKTLELIFLDYNIYLTGWDQNMAKNIML-------HERDVITGLQQKTFGRPQWIFMIGVFCGPKILSSLHVECGTFYYENF

>S.ciliatum_7_NOX2

-WAFTAIWIGLNIFLFACLYIGVGLCLARGAAVPINLNCTLILLPVCRNLISLVRGSGGVWRRLLDLFHKTLAWAILFWSAIHTGAHFWNYVPIMWTVAGFTGFIILAVFFLMITSSIEVIRRFEVFWYTHHLLIFAAFLAHGFGELIAFSWWVVGPLALYFLERVIRIWRATIVVQHPSKVIM-KEGFVGQVIAPEIEWHPFTLSSSPEEDCFSVHIRIVGWTLRP-PKLLIDGPFGTASEDVFRVAVCIGAGIGVTPFASVLIWYQISVQKVFFWLCSDLSAFEWFADLMRSLEMNFVQFRIYLTPGNTAEQIRALV-------ASEDPIVQMQDANFARPNWIFIIGVFCGPKALSSLHTICGTFFYENF

>N.vectensis_3NOX2

-YIVLIIWMGINIYLFIYLLVKFGLALARAPAMVLNFNCMLILLTMCRNLLSFIRGSCCCS-RLLDVFHKYIAYMICLQTAIHCIAHIFNVLPVLWIVSGVTGAVITLALILMLSSSTELIRRFEVFWFNHHCVIFIGLVLHGVQGIIPFTWWVVGPMFLYCIERLIRFVRIQIVVKHPSNVIM-KAGFVGQILCPKIEWHPFTLTSAPEEDTFSVHIRIVGWTLLM-PRLALDGPFGTASIDVFGVGMYIGAGIGVTPFASVLIWYRLNIKKVFFWICNDTNAFEWFTDLLKILEMIFLEYNIYLTGWGANMARDIYL-------REEDPITRLRQKTFGRPEWIFIIGVFCGPKALSHLHKMSGAFYYENF

>L.anatina_3_NOX2

-WVVLIIWLLTNVGLFIFIIVGLGLPFARGAAACLNFNCMLILLPVCRNLISFLRKSCCCKRRQLDIFHKYIAYMICLHTAIHAGAHCFNVFPEALLIAGVTGIVITLALIVMVSSSTDFIRLFEAFWFTHHLIVFIALVVHGIQGVVPFTWWVLAPMIFYFIERCIRFFRVTIVIKHPSRVLM-KAGFAGQVLCPSIEWHPFTLTSAPEDDYFSLHIRLVGWTLAL-PSIAVDGPFGTATEDVFQVDVFVGTGIGVTPFASVLIWHKTKLTKVFYWICPDTNAFEWFTDLLSSLEMAFLDYNIYLTGWDSDQAKNIML-------QDTDAVTGLQKKTYGRPNWIFIIGVFCGPKALSALHKMSGTFVYENF

>B.floridae_7

-WLILVLWFGINIGLFVFLLLGHGLAWARAPAACLNFNCMLILLPVCRNLISYLRGSCCCRRRQLDIFHRAVAYMICLHTAIHFGAHCYNLLAEGLTVAGVTGVVITLALIVMVSSSTEVIRRFEVFWYTHHLIVFIGLVLHGIGGIVPFTWWVLFPMILYIAERCIRFYRVVIVVKHPSKVLM-KRGFAGQIICPSIEWHPFTLTSAPQEDHFSVHIRIVGWTLAM-PQIAVDGPFGTASEDIFQVGVLVGAGIGVTPFASVLIWYQLKLKKVFYWICPDTLSFEWFADLLQSLEMLFLNYNIYLTGWDANQAKNIIL-------HESDVITGLQQKTYGRPHWIFIVGVFCGPKGLSSLHHMCGVFHYENF

>D.rerio_4_NOX2

-VFVILVWLGINVFLFVYTILGHALSWARAPAACLNFNCMLILLPVCRNLLSFLRGSICCSRRQLDIFHKLVAYMIAFHTAVHIIAHLFNFFPVMFTVAGLTGVVITLALILIITSSMEVIRRFEVFWFTHHLIVFIGLVLHGIGRIVPFTWWVVGPMFLYVCERLVRFYRVVIVVTHPSKTLM-KKGFVGQIMCPSIEWHPFTLTSAPEEDHFSVHIRIVGWTLAL-PKMAVDGPFGTASEDVFEAVMLVGAGIGVTPFASVLVWYKVFTKKIFYWLCPETQAFEWFADLLQSLEMSFLSYNIYLTRWKDAEAAHLRV-------QYDDPITGLKQKTYGKPNWEFIVGVFCGPTALGKLSKQCGTFIFENF

>X.tropicalis_1_NOX2

-IAVIIVWLGLNGYLFWYSLYGSALAWARAPAACLNFNCLLILLPVCRNLLSFLRGSSCCGRRQLDLFHKMVAWMIALHTAIHTGAHLFNVLPVAFFLAGLTGVVITLALILIITSSTKTIRRFEVFWYTHHLVIFIGLVIHGAGKIVPFTWWVVAPMVLYVFERLVRFWRVVIVVTHPFKTLM-MKGFVGQIVCPAVEWHPFTLTSAPEEDFFSIHIRIVGWTLAM-PKIAVDGPFGTASEDVFEVAMLVGAGIGVTPFASVLVWYRLRLKKIFYWLCRDTQAFEWFADLLQSLEMQFLVYNIYLTGWDESQATAFSL-------HHKDVITGLKQKTYGRPNWEFIVGVFCGPESLAELNKQSGVFIFENF

>X.tropicalis_9

-IAVIIVWLGLNGYLFWYSLYGSALAWARAPAACLNFNCLLILLPVCRNLLSFLRGSSCCGRRQLDLFHKMVAWMIALHTAIHTGAHLFNVLPVAFFLAGLTGVVITLALILIITSSTKTIRRFEVFWYTHHLVIFIGLVIHGAGKIVPFTWWVVAPMVLYVFERLVRFWRVVIVVTHPFKTLM-MKGFVGQIVCPAVEWHPFTLTSAPEEDFFSIHIRIVGWTLAM-PKIAVDGPFGTASEDVFEVAMLVGAGIGVTPFASVLVWYRLRLKKIFYWLCRDTQAFEWFADLLQSLEMQFLVYNIYLTGWDESQATAFSL-------HHKDVITGLKQKTYGRPNWEFIVGVFCGPESLAELNKQSGVFIFENF

>H.sapiens_CYBB

-IFVILVWLGLNVFLFVYTLLGSALALARAPAACLNFNCMLILLPVCRNLLSFLRGSSCCSTRQLDLFHKMVAWMIALHSAIHTIAHLFNVCPLAVLLAGITGVVITLCLILIITSSTKTIRRFEVFWYTHHLVIFIGLAIHGAERIVPFTWWIVGPMFLYLCERLVRFWRVVIVVTHPFKTLM-KKGFVGQIVCPKVEWHPFTLTSAPEEDFFSIHIRIVGWTLAL-PKIAVDGPFGTASEDVFEVVMLVGAGIGVTPFASILVWYKLKLKKIFYWLCRDTHAFEWFADLLQLLEMQFLSYNIYLTGWDESQANHFAV-------HHKDVITGLKQKTYGRPNWEFIIGVFCGPEALAELSKQSGVFIFENF

>D.rerio_5_NOX1

-AFIVVVWMAINIALFVYTLLGSALAWARAPAAVLNFNCMLILLPVCRNLLSLLRGSFCCGRKQLDLFHKLVAYMIALMTAVHTIAHLFNAYPFVFSIAGLTGVVITLALILMITSSMEVIRRFEVFWYTHHLIVFAGLVFHGAGRVVPFTWYVIGPMIIYICERLLRFIRVTYIVIRPSKVLL-KPGFVGQVLCPAIEWHPFTLTSAPEEDFFSVHIRSVGWTLML-PRMGVDGPFGTASEDVFEVSMLVGAGIGVTPFASILIWYKLRTKRIFYWLCRETHAFEWFADLLQVLEMEFLTYKLYLTGWDQSHADHAMV-------HFTDIITGLKQKTYGRPNWEFVVGTFCGPQALAKLEKKCRTFYFENF

>X.tropicalis_6

-VVVLATWLGLNIFIFIFTLLGSALAWARGSAACLNFNCLLILLPVCRNLLSFLRGTCCVQRKQLDLFHKLVGYTIALMTAIHTIAHLFNVYPFAFTIAGLTGVVITLALILMITSSTEFIRRFEVFWYTHHLVIFIGLVFHGAGRIVPFTWWVLAPMILYIFERTLRFYRVVIAVSHPSKVIM-KRGFVGQIICPSVEWHPFTLTSAPEEDCFSVHIRSAGWTLVP-PRLEVDGPFGTASEDVFEVSMLVGAGIGVTPFASILIWYKLKTKKIFYWICRETGSFAWFADLLRSLEMIFLNYRLFLTSWDSKIAGHVVI-------DFTDTVTGLRQKTYGRPIWEFVVGVFCGPQALGKLKQCCKVFYFENF

>H.sapiens_NOX1

-VLFLVVWLGLNVFLFVFTILG------------------------------------FCSRKQLDLFHKLVAYMICLHTAIHIIAHLFNFYVVTFSIAGLTGVIMTIALILMVTSATEFIRRFEVFWYTHHLIFYLGLGIHGIGGIVPFSWWILAPVILYICERILRFYRVVIVVMHPSKVLM-KRGFVGQIVCPSIEWHPFTLTSAPEEDFFSIHIRAAGWTLAI-PRIEVDGPFGTASEDVFEVAVLVGAGIGVTPFASILIWYKLKTKKIFYWICRETGAFSWFNNLLTSLEMEFLNYRLFLTGWDSNIVGHAAL-------NFTDIVTGLKQKTFGRPMWEFIVGVFCGPRTLAKLRKCCKVFYFENF

>H.sapiens_NOX1.2

-VLFLVVWLGLNVFLFVFTILGSTLACARASALCLNFNSTLILLPVCRNLLSFLRGTCFCSRKQLDLFHKLVAYMICLHTAIHIIAHLFNFYVVTFSIAGLTGVIMTIALILMVTSATEFIRRFEVFWYTHHLIFYLGLGIHGIGGIVPFSWWILAPVILYICERILRFYRVVIVVMHPSKVLM-KRGFVGQIVCPSIEWHPFTLTSAPEEDFFSIHIRAAGWTLAI-PRIEVDGPFGTASEDVFEVAVLVGAGIGVTPFASILIWYKLKTKKIFYWICRETGAFSWFNNLLTSLEMEFLNYRLFLTGWDSNIVGHAAL-------NFTDIVTGLKQKTFGRPMWEFIVGVFCGPRTLAKLRKCCKVFYFENF

>H.sapiens_NOX1.4

-VLFLVVWLGLNVFLFVFTILGSTLACARASALCLNFNSTLILLPVCRNLLSFLRGTCFCSRKQLDLFHKLVAYMICLHTAIHIIAHLFNFYVVTFSIAGLTGVIMTIALILMVTSATEFIRRFEVFWYTHHLIFYLGLGIHGIGGIVPFSWWILAPVILYICERILRFYRVVIVVMHPSKVLM-KRGFVGQIVCPSIEWHPFTLTSAPEEDFFSIHIRAAGWTLAI-PRIEVDGPFGTASEDVFEVAVLVGAGIGVTPFASILIWYKLKTKKV----------------------------------------GHAAL-------NFTDIVTGLKQKTFGRPMWEFIVGVFCGPRTLAKLRKCCKVFYFENF

>H.sapiens_NOX3

-TILVLSWLGINFYLFIFTILGSTLAWARASALCLNFNCMLILIPVSRNLISFIRGTSCCRGRQLDLFHKLVAYGIAVNATIHIVAHFFNLYTELLTIAGVTGLVISLALVLIMTSSTEFIRQYELFWYTHHVIVFLSLAIHGTGRIVPFAWWILGPVVLYACERIIRFWRVVIVVSHPSGVLM-KRGFPGQIVCPAIEWHPFTLTSAPQEDFFSVHIRAAGWTLAL-PRLAVDGPFGTALTDVFPVCVCVAAGIGVTPFAALLIWYKLKLSKVFYWICRDARAFEWFADLLLSLEMSFLSYHIFLTGWDENQALHIAL-------HWTDVITGLKQKTYGRPNWEFIIGVFCGPKALSRLQKMCGVFYYESF

>M.domestica_NOX3

-VVLLLSWLGGNFYLFIFTILGSALAWARASAVCLNYNCMLILLPVSRNFISFLRGTVCCKGRQLDLFHKLVAYAIAINATIHIVAHVINMYIEFLTIAGVTGLVISVVLILIITSSAEIIREYELFWYIHHMVIFIFLVIHGTGQIVPFAWWVLGPMVLYMCERIIRFWRVVIVVAHPSEVLM-KRGFPGQILCPSIEWHPFTLTSAPEEDFFSIHIRVAGWTLVL-PRLSVDGPFGAAVTNVFQVSVCIAAGIGVTPFASVLIYYQLKLNKVFYWICRVPCAFEWFADLLFLLEMAFLSYHIFLTGWDENQAAHIAL-------HWVDAITGLKQKTYGRPNWEFLIGVFCGPKALSKLQKMCGVFNYENS

>T.adhaerens_2

-----LLWMIINVILFAYLYLKTAVAWARGSAAVLNLNCALILLPVCRNLLSYARGSCICSRRLMDIFHRYIGYMICLATAIHVYAHCFNFFP------GITGIIITLALILMYASSTEVIRRFEIFWYAHHLVIFIGLCIHAVIGVIPFVWWVIGPIVLYIFERIVRFVRVVIVINHPSKVIM-KRGFAGQVICPSIEWHPFTLTSAPGDDYFSVHVRIVGWTLAM-PDVYIDGPFGTCSEDVFEVAICIGAGIGVTPFASILLWYKSKTKKVFFWICPDSNAFEWFAELLKSLEMLFLEYNIYLTGLSDKQTKHIML-------RETDAITGLQQKTYGRPRWIFLLGVFCGPKALSSLHKEC--FFYENF

>C.teleta_2_NOX2

-ISLQTLFLIINIVLFVFLMLGLSLPFARASAGALNFNCMLILLPVCRNLITLMRGSCCCYRRQLDILHKLCAYTICFWTAVHYFAHCFNFFPELFNIPGYTGVLITIPLIFIVTSSTELIRRFEVFWFTHHLVVFIALVTHGLGGVVPFTWWVIGPMVLYVIERIVRFVRVTVVIEHPSRVLM-KSGFPGQVLCPSIEWHPFTLTSSPQEDFFSVHVRIAGWTLLL-PKISVDGPFGTCSEDVFQVDVLVAAGIGVTPFASVLIWFKMKLRKVFYWICPDTNAFEWFVDLLKCVEMIFLEINIFWTGWSARQANIIGK-------TVTDVITGLMHKTFGRPNWEFIVGVFCGPKVLSSLHKMSGTFVYENF

>A.planci_7_NOX2

-TIFLLLWLGANVGYWIFIIIGPGVSVAKACGACLNLNSMLILLPVCRNLISFLRGSCLCRRRQLDLFHKTLAYMITLLNIVHCIAHFFNFLPQGVQIAGWSGVVLTLVFIIMFSSATEFIRRFETFWVTHHLVIYAMLLVHGVGGVIPFSWWCVGPIAIYILERFVRFIRVQVVVKHPSKVLM-KSGFSGQVLCPALQWHPFTLTSAPEDDFFSIHIRTVGWTLVL-ARVAVDGPFGTASLDVFQAAVCVGAGIGVTPFASILIWHQLKLKKVFFWICPDTNAFEWFSEMLDSLEMIFLKYNIYLTGWNTKQAKNIYL-------QEVDAITGLKQKTYGRPKWIFIVGVFCGPKVLSELHKCSGAFFYENF

>S.purpuratus_8_NOX2

-YFFLLLWLAANVAYWVFIITGVGLSIAKASGATLNLNSMIILLPICRNLISFFRGSCLCRRRQLDLFHKTVAYMIVIWTIVHVVAHAFNFLPQALPIAGWSGAVLTLVLILMFSSATEFIRRFETFWITHHLIVYAMLLAHGVGGIIPFSYWVSGPLFIYLLERMIRFWRV-------------------------------TLTKAPEDDHFSVHIRVVGWTLAL-ARVAVDGPFGTASIDIFEVAICVGAGIGVTPFASILIWLKLKLKKVFFWICPDTNAFEWFSTLLDSIDFTFLKYYIYLSGWNNTQAKNIYL-------QEIDAITGLRQKTYGRPKWNFIIGVFCGPKALSSLHENAGAFFYENF

>C.intestinalis_1_NOX2

-YIVFLLWLGLNGFLFGYTLLGPALALAR-PAACLNLNCLLVLLPVCRNLLSLFRKACCCPRRVLDIFHRMCAYMIVLMTLIHYFAHCFNVFVAVFLTGGWTGVIITLSLFFMVTSSLEFIRRFEVFWFTHHLIVFGFLVVHGISMQVPFTWWVIAPMVLYVIERIIRLVRVEVVIKHPSRVIM-KNGFVGQVICPQLEWHPFTLTSAPEEDYFSIHVRIVGWTLVM-PRLAIDGPFGTASEDVFPVAICVGSGIGVTPFASLLVWYKMVLKKVFFWICPETHAFEWFGDLLKYLELTLIEYHIYLTGWDHKQAKAIYA-------HEHDVITGLEQKTYGRPNWIFTIGVFCGVAALSALHKMSGVFHYENF

>S.purpuratus_5_NOX1

-WAVLAAWAIVNLIIWLFTLMKNGLPVARASAACLNFNSMLILFPVCRNMISYLRGSCFSRRRQLDIFHKLIAYAIGFFVILHVGAHCFNLLVPGLLLAGWTGAVLALTYILMFTSATEFI-RFETFWLTHHLVIYAMLMTHGMGGVVPFSWWCVTPLCVYFLERILRMIRVTIVVQHQSKVLM-KQGFPGQILCSPIQWHPFTLTSAPEEDYFSLHIRRVGWTLKL-PRVQVDGPFGTSCTDIFDVVMCVSAGIGVTPYASTLICHRLHLKRMFYWICRDTHAFEWFVELLSSLELRLLSYSIYLTGWDYTQAKNIFM-------QEIDAVTGLRQKTYGRPKWNFIIGVFCGPKSLSTLHQSCGTFVYENF

>N.vectensis_2_NOX2

-YFSQIIWAVINIILFAYLVIKNGLPIARGAAMVLNFNCMLILLSMCRNLNSLIRRHCTCMARVLDIFHKYIAYTICFFTIVHVGAHCYNFLK-RGKFAGVTGAVITLCLVVMVSSSTELIRRFEVFWYSHHLIIFAGLVAHGCGEILPFTWIVLLPMVIYFLERCLRFWRVKVVVKHPSRVIM-KPGFAGQVLVPKIEWHPFTLTSAPEEDYFSLHIRVVGWTLQM-PRIAVDGPYGTASTDVFEVVMCIGAGIGVTPFASILIWYRLRVKKVFYWICRDTFAFEWFSDLLKHIEMDFIQINIFLTGWDKKLANQVVM-------ERRDPITGLFARTYGRPEWIFVVGVFCGPSGLSHLHKMCGVFFYENF

>L.anatina_5_NOX4

-YSVVVLWIVLNAVVFTYVILGPTLCISRGTAAVLNLNCGLVLLPMCRVLVSLMRFSRISRRMLLDKFHMLCGLTIVLASVIHFIGHLFNAFPIVASVPGITGLVLMLVLCTMATTSLYVVRTYDLFWYTHRFLVFFFLMVHAVRGVLPFSWWVGLPLLVYLVDLTYRRCKTRIVTEHPGDVIV-KKGFPGQIVCPAIEWHPFTVSMCPTSESFTIHIHAVGWSLYL-PRLVIEGPYGSPSGDILRVNVLIAGGIGVTPFTAFLLYSQ-NIERIFIWMCKDLRSFKWFTQLLCRLYLWLLCIRLHVTA----------------------PVPGLEKRLIGRPDWTLIIGVFCGPKGLATVEEECNALFCESF

>D.rerio_1_NOX4

-HFMMMVWVAVNVFLFWFLMLGLGLCISRASASVLNLNCSLVLLPMCRSLLTFLRGSQVTGRRLLDKFHVACGVTICIFSVVHVSAHLVNLFPLIVTVPGVTGVLLVLVLFLMCTASSYCIRLYQIFWYTHNLILFIILMVHVAGGALAFTWWVSAPLCLYCAERLYRFIRVAIVISHQCDVLM-KSGFPGQILCPTVENHPFTLTTCPTGETFGIHLRVLGWTFLL-PTVHVDGPFGSPSEEVFEVSLCVAGGIGVTPFACVLLYYK--LKRLFVWICRDIQCFYWFADLLCGLYLWYLNVQLYLSS-----------------------SQGLSSRLIGRPNWLFIVGVFCGPKGISKLHTLCHTFEYESF

>X.tropicalis_3_NOX4

---------------------------------------------MCRTVIGLLRGPKVLSRRMLDKFHAACGLAICLFSAVHVGAHVLNAFPIIFSVPGVTGVLMVLILFLMCTASTSSIRTYGIFLHTHNLFIFLLLLLHACAGVLPFTWWISGPLCLYCAERLYRYIRVTIVITHPCDVIM-KEKFPGQILCPSVETHPFTLTMCPTEATFAIHIKVVGWTFLL-PKIYVDGPFGSPSEEVFQISLCIAGGIGVTPFASVLLLYK--LQRLFVWVCRDIHSFLWFADLLCLLHLWYLNIQLYLSQ-----------------------TNGLNSRLIGRPQWLFVVGVFCGPKGISKLHKLCGTFEYESF

>X.tropicalis_5_NOX4

-HLFLLSWLALNIGLFYFLMLGLGLCVSRASASVLNLNCSLVLLPMCRTVIGLLRGPKVLSRRMLDKFHAACGLAICLFSAVHVGAHVLNAFPIIFSVPGVTGVLMVLILFLMCTASTSSIRTYGIFLHTHNLFIFLLLLLHACAGVLPFTWWISGPLCLYCAERLYRYIRVTIVITHPCDVIM-KEKFPGQILCPSVETHPFTLTMCPTEATFAIHIKVVGWTFLL-PKIYVDGPFGSPSEEVFQISLCIAGGIGVTPFASVLLLYK--LQRLFVWVCRDIHSFLWFADLLCLLHLWYLNIQLYLSQ-----------------------TNGLNSRLIGRPQWLFVVGVFCGPKGISKLHKLCGTFEYESF

>X.tropicalis_8_NOX4

-------------------LFLLGLCVSRASASVLNLNCSLVLLPMCRTVIGLLRGPKVLSRRMLDKFHAACGLAICLFSAVHVGAHVLNAFPIIFSVPGVTGVLMVLILFLMCTASTSSIRTYGIFLHTHNLFIFLLLLLHACAGVLPFTWWISGPLCLYCAERLYRYIRVTIVITHPCDVIM-KEKFPGQILCPSVETHPFTLTMCPTEATFAIHIKVVGWTFLL-PKIYVDGPFGSPSEEVFQISLCIAGGIGVTPFASVLLLYK--LQRLFVWVCRDIHSFLWFADLLCLLHLWYLNIQLYLSQ-----------------------TNGLNSRLIGRPQWLFVVGVFCGPKGISKLHKLCGTFEYESF

>H.sapiens_NOX4.2

------------------NLVILGLCLSRASASVLNLNCSLILLPMCRTLLAYLRGSQVPSRRLLDRFHITCGVTICIFSGVHVAAHLVNAFPLLFTVPGLTGVCMVVVLFLMITASTYAIRVYDIFWYTHNLFVFMLLTLHVSGGLLPFTWWISGPLCLYCAERLYRYIRVTIVMSHPSDVIM-KENFPGQILCPSVENHPFTLTMCPTEATFGVHLKIVGWTFLL-PKLYIDGPFGSPFEESLEVSLCVAGGIGVTPFASILLLYK--LRRLFIWVCRDIQSFRWFADLLCMLHFWYVNIQLYLSQ-----------------------TDGLNSRLIGRPRWLFIVGVFCGPNSLSKLHKLSGTFEYESF

>H.sapiens_NOX4.4

----------MNVLLFWFLMLGLGLCLSRASASVLNLNCSLILLPMCRTLLAYLRGSQVPSRRLLDRFHITCGVTICIFSGVHVAAHLVNAFPLLFTVPGLTGVCMVVVLFLMITASTYAIRVYDIFWYTHNLFVFMLLTLHVSGGLLPFTWWISGPLCLYCAERLYRYIRVTIVMSHPSDVIM-KENFPGQILCPSVENHPFTLTMCPTEATFGVHLKIVGWTFLL-PKLYIDGPFGSPFEESLEVSLCVAGGIGVTPFASILLLYK--LRRLFIWVCRDIQSFRWFADLLCMLHFWYVNIQLYLSQ-----------------------TDGLNSRLIGRPRWLFIVGVFCGPNSLSKLHKLSGTFEYESF

>H.sapiens_NOX4.9

----------MNVLLFWFLMLGLGLCLSRASASVLNLNCSLILLPMCRTLLAYLRGSQVPSRRLLDRFHITCGVTICIFSGVHVAAHLVNAFPLLFTVPGLTGVCMVVVLFLMITASTYAIRVYDIFWYTHNLFVFMLLTLHVSGGLLPFTWWISGPLCLYCAERLYRYIRVTIVMSHPSDVIM-KENFPGQILCPSVENHPFTLTMCPTEATFGVHLKIVGWTFLL-PKLYIDGPFGSPFEESLEVSLCVAGGIGVTPFASILLLYK--LRRLFIWVCRDIQSFRWFADLLCMLHFWYVNIQLYLSQ-----------------------TDGLNSRLIGRPRWLFIVGVFCGPNSLSKLHKLSGTFEYESF

>H.sapiens_NOX4.10

----------MNVLLFWFLMLGLGLCLSRASASVLNLNCSLILLPMCRTLLAYLRGSQVPSRRLLDRFHITCGVTICIFSGVHVAAHLVNAFPLLFTVPGLTGVCMVVVLFLMITASTYAIRVYDIFWYTHNLFVFMLLTLHVSGGLLPFTWWISGPLCLYCAERLYRYIRVTIVMSHPSDVIM-KENFPGQILCPSVENHPFTLTMCPTEATFGVHLKIVGWTFLL-PKLYIDGPFGSPFEESLEVSLCVAGGIGVTPFASILLLYK--LRRLFIWVCRDIQSFRWFADLLCMLHFWYVNIQLYLSQ-----------------------TDGLNSRLIGRPRWLFIVGVFCGPNSLSKLHKLSGTFEYESF

>H.sapiens_NOX4.12

-HLCLFIWLSMNVLLFWFLMLGLGLCLSRASASVLNLNCSLILLPMCRTLLAYLRGSQVPSRRLLDRFHITCGVTICIFSGVHVAAHLVNAFPLLFTVPGLTGVCMVVVLFLMITASTYAIRVYDIFWYTHNLFVFMLLTLHVSGGLLPFTWWISGPLCLYCAERLYRYIRVTIVMSHPSDVIM-KENFPGQILCPSVENHPFTLTMCPTEATFGVHLKIVGWTFLL-PKLYIDGPFGSPFEESLEVSLCVAGGIGVTPFASILLLYK--LRRLFIWVCRDIQSFRWFADLLCMLHFWYVNIQLYLSQ-----------------------TDGLNSRLIGRPRWLFIVGVFCGPNSLSKLHKLSGTFEYESF

>H.sapiens_NOX4.7

-HLCLFIWLSMNVLLFWFLMLGLGLCLSRASASVLNLNCSLILLPMCRTLLAYLRGSQVPSRRLLDRFHITCGVTICIFSGVHVAAHLVNAFPLLFTVPGLTGVCMVVVLFLMITASTYAIRVYDIFWYTHNLFVFMLLTLHVSGGLLPFTWWISGPLCLYCAERLYRYIRVTIVMSHPSDVIM-KENFPGQILCPSVENHPFTLTMCPTEATFGVHLKIVGWTFLL-PK------------------------------------YK--LRRLFIWVCRDIQSFRWFADLLCMLHFWYVNIQLYLSQ-----------------------TDGLNSRLIGRPRWLFIVGVFCGPNSLSKLHKLSGTFEYESF

>H.sapiens_NOX4.11

----------MNVLLFWFLMLGLGLCLSRASASVLNLNCSLILLPMCRTLLAYLRGSQVPSRRLLDRFHITCGVTICIFSGVHVAAHLVNAFPLLFTVPGLTGVCMVVVLFLMITASTYAIRVYDIFWYTHNLFVFMLLTLHVSGGLLPFTWWISGPLCLYCAERLYRYIRVTIVMSHPSDVIM-KENFPGQILCPSVENHPFTLTMCPTEATFGVHLKIVGWTFLL-PK------------------------------------YK--LRRLFIWVCRDIQSFRWFADLLCMLHFWYVNIQLYLSQ-----------------------TDGLNSRLIGRPRWLFIVGVFCGPNSLSKLHKLSGTFEYESF

>H.sapiens_NOX4.5

-HLCLFIWLSMNVLLFWFLMLGLGLCLSRASASVLNLNCSLILLPMCRTLLAYLRGSQVPSRRLLDRFHITCGVTICIFSGVHVAAHLVNAFPLLFTVPGLTGVCMVVVLFLMITASTYAIRVYDIFWYTHNLFVFMLLTLHVSG-------------------------------------------------------------------------------------------------------------------------YK--LRRLFIWVCRDIQSFRWFADLLCMLHFWYVNIQLYLSQ-----------------------TDGLNSRLIGRPRWLFIVGVFCGPNSLSKLHKLSGTFEYESF

>H.sapiens_NOX4.8

-HLCLFIWLSMNVLLFWFLMLGLGLCLSRASASVLNLNCSLILLPMCRTLLAYLRGSQVPSRRLLDRFHITCGVTICIFSGVHVAAHLVNAFPLLFTVPGLTGVCMVVVLFLMITASTYAIRVYDIFWYTHNLFVFMLLTLHVSG-------------------------------------------------------------------------------------------------------------------------VQ--LK----------------------------------------------------------------------PKQGFI-------------------------

>B.floridae_5

---------------------------------------------MCRTVLAVIRGSTCVGRRVLDRFHMLCGAAICVAAATHCLGHFYNAFMLMPTVPGLTGVAMVLLLMLIVSTSSMAIRTYELFWYTHHLLILCLMLLHPMRGILPFVWWLVGPFCLYCAERVYRLVRAAIIIEHPDEVVL-KEGFPGQVICPDVEYHPFTLT-MPSSMSIRLYTRIYVPFLHA-P-LYVDGPFGSPSENVFDVSLCIAGGIGITPFASVLLLSR--LRRLLVWVCKNITSFLWIAQVLCSLQAWFLNIHLHLTR-----------------------SRDLNSRVVGRPQWIFVIGVFCGPKGMSKVKKICHTFEFENF

>C.intestinalis_2_NOX4

-LFIWVAWISINASLFYFLMLGYGLCISRASAACINLNSSFILFPMCRGLVTFMRGLPGVGRRLLDRFHILCGYILCLLAGVHCAAHAYNAFPMLVSLSGITGILLVISLVVISAFASRPIRRHNKFWKTHHIIVFALIFIHAMDGVIPFAWWLCAPLIIYVIERIGRHFRTTIFIEHPCDVLL-RNGFPGQIVCPQLESHPFSLTSVPSKPTFGIHVKLRGWTLLMMPILCVEGPTGGAMEDIFKISMCVAGGIGVTPYASVLLLKDMKLKRLLIWSCKDPRSFSWFASLIRDVQLWLLSVRLHITG--------------------TLQESGQGCHVYGRPDVVFIVGVFCGHRLLVSVKHHCKVFLFEAF

>N.vectensis_1_NOX4

-FFFQVLWTGPFIVIFWYLMLGNSLSWSRGAAAVLWFSCSLMLLPMCRNLLAFVRNTLCSSRRLLDIFHKACAITTIIAAVVHTVAHLINGFPFVMSVAGFTGMGMMLVLLIMIAASTPIVRNYEVFWFTHHAIAFLMLAVHGLGGVICYSWLLILPLSIYLIDRLIRVVRVTVVVNHPCGVLM-KSGFPGQVVCHSVEWHPFTLTKCPSSDSFSIHIKRTGWTLQLAPVLSVDGPYGSPCMDVERVSMCIATGIGVTPFAALIIRSQPRPHRLFVWICREVGALQWFADLIHETSLWFLTCLFYITS--------------------KECINHFDARLHGRPDWIFVVGVFCGARGPSSLRRCCGAFVFEVS

>P.anserina_NOX2

-RIFVFVFMFLHAILFAFATFGPTFMIARSAALVLHVDVALILFPVCRTLISMARQTPLIQ---FDIFHITTAWSIVFWSWVHTIAHWNNFV-ANFSGPGWTGYVMLIALMGMVITSVEKTRRYERFWYTHHMIVFFFWSIHGPDFV--F-WYWMYGGFAYLAERVAREIRTYIVIQHPSNVII-KEHTAGQIFCPAVQYHPFTLTSAPEEDYISIHMRVVGFTVTL-PRVYIDGPFGSASEDVFEISVLCGAGIGVTPFASILIWYRTRLSKVFFWICRDFGSFEWFRSLLLAIER--IEIHTYLTKIKVDDATNIMIN------DAKDTITGLRSPTFGRPNWIFIAGVFCGPKGLGSLHVFCGFFVWENF

>P.anserina_NOX1

-ILFHILFWTFHWGIFAWLTLQYSVWLSRGAGLVLSVDGMLILLPVCRTIMRFIRPK-IIP---LDIMHRQLAYSMLLFTIIHTAAHYVNFV-IHYQPGGATGHVMLLCMLLMYTTAHHRIRQFETFWYTHHLIPFLGLYTHTVGC---FVWWELWTGGFYLIERLYREIRTKIVVKHPYDVIF-KPSFAGQLLVPSVQWHPFTITSCPYDPYVSVHIRQVGFTLAM-PALRIDGPYGAPAEDVFEIAVLIGTGIGVTPWASILIWHLTRLRRVFIWVCKDTSSFEWFQTLLLSLESAFLKIHTYLTKLDMDTTQNIVLN------SVVDPLTELKARTFGRPNFIFMVGVYCGPSAAARIKKAAEVFRFEHF

>A.thaliana_RBOHD

ILWIMMLWLGICGGLFTF-VMGYCVCVAKGGAETLKFNMALILLPVCRNTITWLRNTKLVP---FDLFHKVIASGIVVGVLLHAGAHL----PWFVGVEGWTGIVMVVLMAIAFTLATPWFRRFNAFWYTHHLIIVALLIVHGIKLYLTT-WYLAVPILLYASERLLRAFRVKMVAVYPGNVLMKPQGFSGQMVCRAVEWHPFSITSAPGDDYLSVHIRTLGWTLVFFPKVLIDGPYGAPAQDYKDVVLLVGLGIGATPMISILIINNFKTRKAFYWVTREQGSFEWFKGIMISI----IELHNYCTVYEEGDARVALIAMLQSLQHAVDVVSGTRVKSFAKPNWVYIIGVFCGMPGMIKLKNLATTFDFENF

>A.thaliana_RBOHC

ICWVIVLWFIVMAILFTY-VMGDCVCMAKGAAETVKLNMALILLPVCRNTITWLRNTRLVP---FDLFHKVIAVGIIVGVTMHAGAHL----PHFVSVEGITGLVMVLLMAIAFTLATPWFRRFNAFWYTHHLVIVILLVAHGYYLYLTT-WYLVVPVVLYACERLIRAFRVTIVAVYPGNVILRPQNFSGQMVCAAVEWHPFSITSAPQDDYLSVHIRVLGWTLVFFPKVLIDGPYGAPAQDYKEVVLLVGLGIGATPMISIVIVNNFRTRRAFYWVTREQGSFDWFKNIMVAV----IEMHNYCTVYEEGDARSALIHMLQSLNHAVDIVSGTRVMSFAKPNWVYIVGVFCGAPALTKLRHLASTFSFENF

>A.thaliana_RBOHA

IVWVMALWIGAMAGLFTF-VMGVCVCIAKGAAETLKLNMAMILLPVCRNTITWLRTTKLVP---FDLFHKVIAIGISVGVGIHATSHL----TDFVSVEGVTGIGMVVLMTIAFTLATTWFRRFNAFWYSHHLVIVSLLVVHGFYVYLTT-WYLMVPVVLYLCERLIRAFRVSVVAVLPGNVLLRPSNFSGQMLCSAVEWHPFSITSAPGDDYLSVHIRVLGWTLLFFPRILIDGPYGAPAQDYKEVVLLVGLGIGATPMISIVIINNFRTKRAFYWVTREQGSFDWFKNVMVTV----IELHNYCTVYEEGDARSALITMLQSLNHAVDVVSGTRVMSFARPNWVFIVGVFCGAAGLVKLRHLSSTFIFENF

>A.thaliana_RBOHB

IIWVLTLWISICITLFTF-VMGYCVTVAKGSAETLKFNMALILLPVCRNTITWLRTSKIVP---FDIFHKVVAFGIAVGIGLHAISHL----PWFMGTDGWTGVTMVVLMLVAYVLAQSWFRRFNAFWYSHHLVIVVLLIVHGYFVYLTT-WYLAVPVLLYAFERLIRAFRVKVVAVYPGNVLMKPKGFSGQIICSDVQWHPFSITSASGDDYLSVHIRTLGWTLLYFPRLLIDGPYGAPAQDYRDVLLLVGLGIGATPLISIIVLNNVATKRAFYWVTREQGSLEWFSEVMVAM----IELHNYCTVYEEGDARSALITMLQSLHHAIDIVSGTRVRTFARPNWVFVVGVFCGNTCIIGLKRLATTFEFENF

>A.thaliana_RBOHF

IIWVLSLWIMIMIGLFLF-VMGYCLLTAKGAAETLKFNMALILFPVCRNTITWLRSTRLVP---FDIFHKTIAGAIVVAVILHIGDHL----PDLVGPEGITGILMVILMIISFTLATRWFRRFNAFWYSHHLVIVILLILHGIFLYFTT-WYLAVPVLLYGGERTLRYFRVRLVAIYPGNVLMKPTQFSGQMVCPAVEWHPFSITSAPEDDYISIHIRQLGWTLVFLPKLLIDGPYGAPAQDYRDVLLLVGLGIGATPFISILLLNNLKTTNAFYWVTREQGSFDWFKGVMVAV----IEMHNYLTVYEEGDARSALITMVQALNHAVDIVSGTRVRTFARPNWVLLIGVFCGVPVLGKLSKLCSTFEFEHF

>A.thaliana_RBOHE

ISWVLLVWVMLMAILFVF-VMGYCLTTAKGAAETLKLNMALVLLPVCRNTLTWLRSTRAVP---FDIFHKIIACAIAIGILVHAGTHL----PDLMGAEGITGISMVILTTIAFTLASTHFRRFNAFWYTHHLVVVIMLIVHGTFLFFTT-WYISVPLVLYVAERSLRACRVKIVSMLPGEVLMKPPGFSGQILCPTIEWHPFSITSAPGDDQLSVHIRTLGWTLVLRPKLLVDGPYGAPAQDYRDVLLLIGLGIGATPFISILLLNN-KAVKAFYWVTREPGSVEWFRGVMISQ----IELHNYLTVYDEGDARSTLIKMVQALNHAVDILSGTRVRTFARPNWVFIVGVFCGIQTVAKLKKQATTFEFEHF

>A.queenslandica_5_Duox

FIFYLSMFFLITAAIFVALIAGYGVTVTRGAASCMMWTYSVLLITMARNLFTYLRETIFIP---FDIFHKVVAMTALFTTVMHCIGHGINFIPWLFTMTGFSAFVLTLITVIIFVFAVQYARRFQSFWLTHHLIVFILMFLHGSGRLVPF-GFFLGPGIVYVIDRLVSLGRVSIADILPSQVIFRPPSFAGQVISLAQEYHPFTLSSAPNEENLSLHIRAVGWTFICLPKLFVDGPFGEGHQDWYEAAVLVGGGIGVTPFASILLVHRIQCKKVFIWVTRTQHQFEWMADIIKEVEL--VEVHVFVTFFDKFDLRTSMLYVAERHFQRRSLFTGMRAITFGRPDFFFLIGVFCGPPGMTNVEEACGPFIHENF

>X.bergquistia_3_Duox

FIFYLTMFFLIVFCIFIALIAGYGVTVTRGAASSMMFTYSVLLITMARNFFTYLRETIFIP---FDIFHKIVALTALAFTVMHGIGHGINFIPWLFTMTGFSAFILSLVTVVIFVFAVQYARRFQAFWFTHHLIVFILMFLHGSGRLVPF-GFFLGPGIVFVLDRLISLGRVSVADILPSQVIFRPPSFAGQVISLGQEYHPFTLSSAPNEENLSLHIRAVGWTFIFYPKLYVDGPFGEGHQDWYEAAVLVGGGIGVTPFASILLVHRVQCKKVFIWVTRTQSQFEWMADIIKEVEL--VEVHIFVTFYDKFDLRTAMLYIAERHFQRRSLFTGMRAVTFGRPDFFFLIGVFCGPPGMTNVEEACGPFIHENF

>T.wilhelma_4_Duox

---------------------------------------------------------------------------------------------------GFAAFMVTLVTVVIFVFATQYARRYRAFWVTHHWVIFILSFLHGSGRLVPF-GFFLGPGIVYALDLIISVGRLAVADILPSNVIFRPVTFAGQVISAAQEFHSFTISSAPHEDLLSLHIRAVGWTFNYYPKVFIDGPFGEGHQDWFEVAVLVGGGIGVTPFASILLVHRIECKKVFIWVARSQKQYEWFSDIIRDVEN--VEIHIFVTFFDKFDLRTTMLYVCERHFQKKSLFTGLRAVTFGRPDFFFLIGVFCGPPGMTNVEDACGPFIHENF

>E.muelleri_7_Duox

AIFYLSLFFLISAGIFVALIAGYGVTITRGAASGMMWTYSILLITMSRNFITYLRETPFVP---FDIFHKIVAFTGLLFTITHCIGHGLNFIPWIFTMTGFSAILLTLITIIIFVFATQYARRFQAFWFTHHWVLFVFMFLHGSGRLVPF-GFFLGPVCVYTLDKIIGVSRVSVAEMLPSGVLMRPTGFAGQVVCSKLEYHPFTISSAPHEEEMTMHVRAIGWTIVYYPKLSIDGPFGEGHQDWYMLPSWLLASLHFHPSSRTLLGHEFRCT--------------------------------------------------------------------------------CGSPVPRSMSGSP--------

>E.muelleri_2_Duox

AIFYLSIYAFITMGIFVALIAGFGVTITRGAASGMMWTYSVLLVTMSRNFITYLRETPLVP---FDVFHKIVALTGLIFTLTHIVGHGINFIPWLFTTTGFSAFVLTLITIAIFVFATQYARRFQAFWLTHHWILFMFMLLHGTGRLVPF-GFLLGPAILYTIDKIISVSRVGVAELLPSGVVMRPASFAGQMISAQLEYHPFTISSAPHEEHLSVHVRAVGWTLLYLPKLRVDGPFGEGHQDWYDVSILVGGGIGITPFASILIVHRFQCKKLFIWVTRTQNQYEWFTDLIREVEL--VEVHIFITFFSKFDLRTTMLYVCERHFQKRSLFTGLRSVTFGRPDFFFLIGVFCGPPGMTTVNEACGPFIHENF

>E.muelleri_6_Duox

AIFYLSLFFLITAGIFVGLIAGYGVTITRGAASGMMWTYSVLLITMSRNFITYLRETPFVP---FDIFHKIVAFTALLFTITHCIGHGLNFIPWLFTMTGFSAFLLTLIVIVIFVFATQYARRFQAFWFTHHWVLFIFMFLHGSGRLVPF-GFFLGPVCVYTLDKIISVSRVSVADMLPSGVLMRPTGFAGQVVCSKLEYHPFTISSAPHEEEMTMHIRAIGWTIVYYPKLSIDGPFGEGHQDWYDVAILVGAGIGVTPFSSILIVHKIQCKKVFLWVTRTQKQYEWFTDVIREVEL--VEMHIFITLLNKFDLRTTMLYACERHFQKRSLFTGLRSVTFGRPDFFFLVGVFCGPPSLTNVEDACGPFVHENF

>O.carmela_4_Duox_m.32550

IIFWVLLFHLSVMLIFVALIAGYGVTITRGAASAMAFTYSVILIPMCRNTITLLRETPFVP---FDHFHKHIAITALVYTLTHIIGHSINFIPWCWTLTGVSAVLVTLVMATMYVFAVPYARRFKFFWSTHKLIVFALLVLHGCGRLVPF-YYFAFPAVLFLVDKLISISRIRVAEILPSAVLFRPDDFSGQVISHGLEYHPFTLTSAPHEEHLSLHIRAVGWTLIYYPLLYLDGPFGEGHQDWHDVSVMVGGGIGVTPFASILVVHKMKCKKLFVWVTRTQKQFEWLTDIIREVEL--VDIHIFITFYKKFDLRTTMLYICERNMERRSLFTGLRSVTFGRPKFFFMIGVFCGPPPMTDVNAACGPFHHENF

>O.carmela_1_Duox

VIFLTIIYQLVTVCIFVALIAGYGVTVTRGAASAMMFTYSTLLLTMCRNAITHLRSTQFIP---FDHFHRQFAMTALFYTAIHIVGHAINFIPWLFTLTGVTAVLLTAVTAVIFVFATPYARRFNAFWITHRLIVFILMILHGAGRLVPF-YYFVGPVCAFIIDRLISSSRLAVAELLPSGVLIKPRNFSGQVICLAQEFHPFTLTSAPHEESLSLHIRAVGWTLTYFPKLYLDGPFGEGHQDWYEVAVMVGGGIGVTPFASILIVRKLNCKKCFVWVTRTQRQFEWMTDIIREVEL--IDVHIFITFYNKFDLRTTMLYVCERHFQRRSLFTGLRAITFGRPEFFLIIGVFCGPLPMTGVKSACGPFVHENF

>S.ciliatum_2_Duox

LVFWLCLYHLVVFCIFAALIAGYGITVTRGAASAMMFTYSTLLVTMCRNFITRLRETPVIP---FDHFHKVVAMTALLFTCIHIIGHSMNFIPWMFTLTGMTGFLLTLVVATMYIFALPVVRQYFVFWKIHQLVVLILLILHGTGKLVPF-QYFLAPVVCYTLDRLISLSRITVAELLPSDVLFKPDNFSGQVICLALEYHPFTLTSAPHEDTLAVHVRAVGWTLVFLPKLHLDGPFGEGHQDWYSVSVLVGGGIGVTPFAAILIVHRLPCKKVFLWVTRTQRQFEWFTDIIRLVEF--IDIHIFITFYQKFDL-----------------------------------------------------------

>S.ciliatum_4_Duox1

LIFWFTLYQLVVGAIFVALIAGYGVTVTRGAASAMMFTYSSLLVTMSRNIITRLRETFLIP---FDHMHKLIAYTALIYTIVHCIGHAINFIPWLFTLTGVTGYLLVLVCVTLYIFAMPYARRFRTFWITHHLIVMILLILHGSGRLVPF-QFFIVPASVFAIDKLISYSRIVVAEKLPSGVLFKPSNFSGQVICLSLEYHPFTLTSAPGEPTLDLHIRAVGWTLVYFPRLYLDGPFGEGHQDWFEIAVLVGGGIGVTPFASILVVHKFAAKKVFLWVTRTQKQFEWMTDIIRQVEL--TDIHIFITFYQKFDLRTIMLYICERHFQKRSLFSGLRSVTFGRPQFFFLIGVFCGPPPMTRVEEGCGPFIHENF

>S.ciliatum_5_Duox1

LIFWFALYQLVTLAIFGALIAGYGVTVTRGAASAMMFTYSSLLVTMSRNVITHLRETFLIP---FDHAHKAFAMTALFYTVIHILGHCINFIPWMFTLTGVTGYVLTLVIITMYVFALPTARRFNTFWFTHHLFVLALLILHGSGRLVPF-QYFIGPALLFAADKLISISRIIVAEKLPSGVLFKPTNFSGQCICLALEYHPFTLTSAPHEVTLDLYIRAVGWTVVYFPKLYLDGPFGEGHQDWYQVAVLVGGGIGVTPFASILLVFKIACKKVFLWVTRTQKQFEWLTDIIRQVEL--TDIHIFITFYQKFDL-----------------------------------------------------------

>S.ciliatum_6_Duox2

IIFWLTLYLLVVLCIFAALIAGYGVTVTRGAASAMMFTYSSILVTMCRNLLTKLRETPLIP---FDHAHKVIACVALFFTVMHILGHCIKFIPWMFTLTGITGLLLTFVVVTIYIFALPQARRFNTFWIVHRLIVLILLILHGSGRLVPF-QFFIVPAALFVVDKLISLKHIPVAEKLPSGVLFKPKNFSGQCISRALEYHPFTLTSAPHEETLDLYIRAVGWTIIYFPRIYLDGPFGEGHQDWYDIAVLVGGGIGVTPFASILLVCKIPCKKVFFWVTKTQKQFEWLTEIIKQVEF--TGIHIFITFYQKFDIRTIMLYICERHFQKQSLFTGLKSTVFGRPQFMFLIGTFCGPPPMTRVEAACGPFFHENF

>A.planci_6_Duox

VLFYLVLYNLVLAGIFIALIAGYGVTVTRGAASAMMFTYSTLLATMCRNTITFLRGTFLVP---FDLFHKFVAMLALFWSVVHTIGHSLNFIPWAYTITGFTGILLVMVCAIMYTFAFSYARRFNLFWLTHNLAFYFLMVMHGSGNLVPF-YFFLPPVIVFTLDKLVSVSRITVAELLPSNVLFRPTTFSGQVICKTLEYHPFTLSSAPHEENLSLHIRAVGWTLTFYPKLFLDGPYGEGHQDWYEVSVLVGGGIGVTPFASILLVYRFTCKKVFIWVTRTQKQFEWLTDIIREVEL--VSVHIFITFFQKFDLRTTMLYICERHFQKRSLFTGLRSITFGRPQFFLLIGVFCGPPGMTNVEKACGAFIHENF

>S.purpuratus_9_Duoxb

VIFYVVLYLLVLAGVFIALIAGFGVSVTRGAASAMMFTYSSLLVTMCRNTITKLRETFLVP---FDLMHKLIAMLALFFSIMHTIGHSINFIPWAWTITGFTGILLVMVCTVIYTFAFQYARRFNLFWFTHNMIIYILMFLHGSGRLVPT-HFALGPIVLFTLDKLVSISRIAVAELLPSDVLFRPQGFSGQVICKTLEYHPFTLTSAPHEENLSLHIRAIGWTLTYLPKLFLDGPYGEGHQDWYEVAVLVGGGIGVTPFASILIVNKVTCKKVFIWVTRTQKHYEWLTDIIRDVEL--VSVHIFVTFFQKFDLRTTMLYICERHFQKRSLFTGLKSITFGRPQFFLLIGVFCGPPGMTGVEQACGAFIHENF

>L.anatina_2_Duoxd

-IFWSVLYSLVCIGIFVALIAGFGVTVTRGAASGMMFTFACLLVTMCRNLITFLRETVAIP---FDIFHKFIAAWALFFTAMHIIGHGINFIPWCWTITGFTGVLLTLLVILMYVFATQYSRRFQAFWITHNMYLLILMVMHGAGRLVGT-HFILGPLILFALDKLVSVSRIAVAEALPSDILFKPTSFSGQVICITMEYHPFTLTSAPHEDTLSLHIRAVGWTLTYFPKLYLDGPFGEGHQDWYDVSVLVGGGIGVTPFASILLVHRFTCKKIFLWVTRTQKHFEWLTDIIREVEL--VSVHIFITFFHKFDLRTTMLYICERHFQKRSLFTGLRSITFGRPQFFLLIGVFCGPPAMTRVETACGAFIHENF

>C.teleta_3_Duoxa

AIFWCTLYTLITLGIFIALIAGYGVTVTRGAASGMMFTYASLLVTMCRNTVTFLRETFLIP---FDIFHKYIAYLALFFTLLHIIGHCINFIPWCWTLTGVTGVLLTINMFVIYVFAVQYARRFKAFWFTHNTPIFILMILHGAGRLVPT-HFILGPVVLFTLDRLVSINRIAVAELLPSDVLFRPLNFSGQCICLVQEYHPFTLTSAPHEENLSLHIRAVGWTLTYWPKVFLDGPYGEGHQEWYEVAVLVGGGIGVTPFASILIKYKFVCKKVFLWVTRTQRQFEWLTDIIREVEL--VSVHVFITFKEKYDIRTTMLYICERYFQKRSLFTGLRSITFGRPEFFFLFGVFCGPPPMTSVEKACNVFQHENF

>B.floridae_2

IIFWLTLYTLVTIGIFAALIAGYGVTVTRGAASGMMWAFSIILVTMCRNTITHMRETFLIP---FDILHKIIAMTGLFFTVMHCIGHAINFIPWCFTTTGMTGVLLVLVIAIMYVFATQYARRFKAFWLTHNLPILILTIVHGSGNLVPF-YFFLGPVILFTLDKLVSISRIPVAEHLPSNVLFRPTNFSGQVISAALEYHPFTLTSAPHEDTLSLHIRSVGWTLVYYPKVFVDGPYGEGHQDWYPVAILIGGGIGVTPFAAILIVQKFNCKKVFLWVTRTQKQFEWLTDIIREVEL--VSVHIFITFYQKFDLRTTMLYICERHFQRRSLFTGLNSITFGRPNFFFLFGVFCGPPPMTYADKACGAFIHENF

>B.floridae_4

VIFWMTLYLLVTAGIFIALITGFGVSVTRGAASGQMWTYSVILLTMCRNTITHMRETFLIP---FDIMHKIVAMSALFFTIMHCFGHGINFIPWLYTTTGMTGVLLVLLLAVMYVFSTQYARRFKAFWFTHNLPILILTIVHGSGHLVPF-YFLLGPLVLFTLDKLVSISRIPVAELLPSAVLLRPANFSGQVISAALEYHPFTLTSAPHEDTLSLHIRSVGWTLVYLPMVFVDGPYGEGHQDWYPVAVLIGGGIGVTPFAAILIVHKFVCKKIFLWVTRTQKQFEWLTDIIREVEL--VSVHIFITFYQKFDLRTTMLYICERHFQRRSLFTGLRSITFGRPDFFLLVGCFCAGPGVTYVEEQQNFFCDENV

>B.floridae_8

-LLYLIFYFAITFALFVAILTQYGIIASRGAAASMSFSFSLLLLTMCRNSLTKLRETFLIP---FDVFHKIVAITALVFTIIHTLGHLVNVFPWAFTMAGVTGVLLLLVVSIIYVFATGYARRFSAFWATHQLIVMILTVLHGSGRLIPF-HYLIGPAIIFTLDKLVSISHVDITELLPSDVIFRPDDFSGQVICPEQEYHPMTLTSAPHENTLSVHVRAVGWTLTYTQKLFLDGPFGEGHQDWYKIAVLVGGGIGITPFASILVAYLMNCKKVFLWVTRTQRHFEWFIDIIREVEL--VAVHIFITFFEKFDVRTTMLYIYERHFQRRSLFTGLRATTFGRPEFLLIIGVFCGPPGMTRVEDACLAYVSENF

>L.anatina_10_Duoxc

FIFYLIIFYGICIGLFSFLIASYGVPVSRGSAAALTFCYSLILLTVCRNLITRLRETFLIP---FDVFHKVIAWTALFFTLAHVVGHALNFIPWLLTLTGMTGVLVTIVIFVIYIFATQCARQFNAFWATHKLIPMILTILHGSGRLLPF-WYFVGPAILFTIDKLVSISRISVAELLPSDVLFRPPNFSGQVICLSQEYHPFTLTSAPQEDTLSLHIRAIGWTLLYYPKLFLDGPYGSGHQDWYDVSILVGGGIGVTPFASILFVHMIKCQKLFIWVTASQRHYEWLIDILRQVEL--VNIHIFITFFQKFDLRTAMLYICEEHFTKKSLFTGLRAKTFGRPQFIFVVGVFCGPPGLTKVEKACKAFEHENF

>D.rerio_2_Duox1

VIICTVVIYAISAGLALCITSMVGVLVSRGSAAAISFLFPYMLLTVCRNLITMCRETFLIP---FDILHRQMAATALILSVVHSLGHLVNVFPWFFTVPGITGVILLLIFAFMYVFASHYFRRFRGFWITHHLVLIVLTVVHGSYGLLPF-HYLIPPGLLFLLDKLISLSRIPVAELLPSDVLFRPQGFSGQVICLTLEYHPFTLTSAPHEETLSLHIRAAGWTLAYLPKLYLDGPFGEGHQEWTEVSVLVGAGIGVTPFASILLVFKFHCKKVFLWVTRTQRQFEWLSDIIREVEL--VSVHIYITLPEKFDLRTTMLYVCERHFQKRSLFTGLRSVTFGRPPFFLLVGVFCGPPGLTKVEKACQTFVHENF

>D.rerio_3_Duox1

VIICTVVIYAISAGLALCITSMVGVLVSRGSAAAISFLFPYMLLTVCRNLITMCRETFLIP---FDILHRQMAATALILSVVHSLGHLVNVFPWFFTVPGITGVILLLIFAFMYVFASHYFRRFRGFWITHHLVLIVLTVVHGSYGLLPF-HYLIPPGLLFLLDKLISLSRIPVAELLPSDVLFRPQGFSGQVICLTLEYHPFTLTSAPHEETLSLHIRAAGWTLAYLPKLYLDGPFGEGHQEWTEVSVLVGAGIGVTPFASILLVFKFHCKKVFLWVTRTQRQFEWLSDIIREVEL--VSVHIYITLPEKFDLRTTMLYVCERHFQKRSLFTGLRSVTFGRPPFFLLVGVFCGPPGLTKVEKACQTFVHENF

>D.rerio_6_Duox1

VIICTVVIYAISAGLALCITSMVGVLVSRGSAAAISFLFPYMLLTVCRNLITMCRETFLIP---FDILHRQMAATALILSVVHSLGHLVNVFPWFFTVPGITGVILLLIFAFMYVFASHYFRRFRGFWITHHLVLIVLTVVHGSYGLLPF-HYLIPPGLLFLLDKLISLSRIPVAELLPSDVLFRPQGFSGQVICLTLEYHPFTLTSAPHEETLSLHIRAAGWTLAYLPKLYLDGPFGEGHQEWTEVSVLVGAGIGVTPFASILLVFKFHCKKVFLWVTRTQRQFEWLSDIIREVEL--VSVHIYITLPEKFDLRTTMLYVCERHFQKRSLFTGLRSVTFGRPPFFLLVGVFCGPPGLTKVEKACQTFVHENF

>X.tropicalis_2_Duox2

-IVCLIIFYGISAGLFAAIATFIGLIISRGSAASISFMFSYMLLTMCRNLITLLRETFLIP---FDVFHRLIAVTALVLSILHSLGHLVNVFPWFFTVPGMTGVLLLAVMALMYVFSCYHFRRFRCFWLTHHLVVFILTIIHGSFALIPF-HFFIVPALIYSADKLISLSRINVIQRLPSDVLFRPNDFSGQVICLDLEYHPFTLTSAPHEDTLSLHIRAVGWTLLYYPKLYLDGPFGEGHQEWNEVSVLVGGGIGVTPFASILLVFKIHCKKVFIWVTRTQHQFEWLTDIIREVEL--LSVHIYITLAEKFDFRTTMLYICEQHFQKQSLMTGLRSVTFGRPPFFFLIGVFCGPPGMTKVENACESFVHENF

>X.tropicalis_7_Duox1

LIVCVVIFYGISVGLFLAIVTMPGIIISRGTAASISFMFSYILLTMCRNLITFLRETFLIP---FDVFHRLVAKTAIILTVLHSVGHAVNVFPWFFTIPGLTGVLLLAVLALMYVFSSHHFRRFRGFWVTHHFILLILTILHGSFGLLPF-HFLMAPALIFIRDSLISLRRINVADLLPSDVLFRPADFSGQVICLALEYHPFTLTTAPHEDILSLHIRAAGWTLLYYPKIYLDGPFGEGHQEWNEVSVLVGGGIGVTPFASILLVFKIACKKIFIWVTRTQRHFEWFADIIREVEL--VSVHIYITLAEKFDLRTTMLYICERHFQKRSLFTGLRSITFGRPQFFFLIGVFCGPPGMTKVEKACQAFSHENF

>H.sapiens_DUOX2

LIVCVAIFSAICVGVFAAITTLVGIILSRGTAASVSFMFSYILLTMCRNLITFLRETFLVP---FDVFHRWIAMAAVVLAILHSAGHAVNVFPWFFTVPGMTGVLLLLVLAIMYVFASHHFRRFRGFWLTHHLILLALLIIHGSYALIPF-HYFLVPAIIYGGDKLVSLSRISVAELLPSGVLFRPQGFSGQVICLALEYHPFTLTSAPHEDTLSLHIRAVGWTLIYYPKLYLDGPFGEGHQEWHEVSVLVGGGIGVTPFASILLVFKMLCKKIFIWVTRTQRQFEWLADIIQEVEL--VSVHIYVTLAEKFDLRTTMLYICERHFQKRSLFTGLRSITFGRPPFFFLIGVFCGPPGMTKVEKACRAFMHENF

>H.sapiens_DUOX2.2

LIVCVAIFSAICVGVFAAITTLVGIILSRGTAASVSFMFSYILLTMCRNLITFLRETFLVP---FDVFHRWIAMAAVVLAILHSAGHAVNVFPWFFTVPGMTGVLLLLVLAIMYVFASHHFRRFRGFWLTHHLILLALLIIHGSYALIPF-HYFLVPAIIYGGDKLVSLSRISVAELLPSGVLFRPQGFSGQVICLALEYHPFTLTSAPHEDTLSLHIRAVGWTLIYYPKLYLDGPFGEGHQEWHEVSVLVGGGIGVTPFASILLVFKMLCKKIFIWVTRTQRQFEWLADIIQEVEL--VSVHIYVTLAEKFDLRTTMLYICERHFQKRSLFTGLRSITFGRPPFFFLIGVFCGPPGMTKVEKACRAFMHENF

>H.sapiens_DUOX1

LIGCVAVFYAIAGGLFLAITTRVGIILSRGTAASISFMFSYILLTMCRNLITFLRETFLVP---FDVFHRLIASTAIVLTVLHSVGHVVNVFPWFFTVPGLTGVVLLLILAIMYVFASHHFRRFRGFWLTHHLILLVLLIIHGSFALIPF-HFFLVPAIIYGGDKLVSLSRISVAELLPSGVLFRPQGFSGQVICLALEYHPFTLTSAPHEDTLSLHIRAAGWTLIYYPKLYLDGPFGEGHQEWHEVSVLVGGGIGVTPFASILLVFKVFCKKIFIWVTRTQRQFEWLADIIREVEL--VSVHIYITLAEKFDLRTTMLYICERHFQKRSLFTGLRSITFGRPPFFFLIGVFCGPPGMTKVEKACRTFSHENF

>DUOX1.2

LIGCVAVFYAIAGGLFLAITTRVGIILSRGTAASISFMFSYILLTMCRNLITFLRETFLVP---FDVFHRLIASTAIVLTVLHSVGHVVNVFPWFFTVPGLTGVVLLLILAIMYVFASHHFRRFRGFWLTHHLILLVLLIIHGSFALIPF-HFFLVPAIIYGGDKLVSLSRISVAELLPSGVLFRPQGFSGQVICLALEYHPFTLTSAPHEDTLSLHIRAAGWTLIYYPKLYLDGPFGEGHQEWHEVSVLVGGGIGVTPFASILLVFKVFCKKIFIWVTRTQRQFEWLADIIREVEL--VSVHIYITLAEKFDLRTTMLYICERHFQKRSLFTGLRSITFGRPPFFFLIGVFCGPPGMTKVEKACRTFSHENF

>H.sapiens_DUOX1.3

LIGCVAVFYAIAGGLFLAITTRVGIILSRGTAASISFMFSYILLTMCRNLITFLRETFLVP---FDVFHRLIASTAIVLTVLHSVGHVVNVFPWFFTVPGLTGVVLLLILAIMYVFASHHFRRFRGFWLTHHLILLVLLIIHGSFALIPF-HFFLVPAIIYGGDKLVSLSRISVAELLPSGVLFRPQGFSGQVICLALEYHPFTLTSAPHEDTLSLHIRAAGWTLIYYPKLYLDGPFGEGHQEWHEVSVLVGGGIGVTPFASILLVFKVFCKKIFIWVTRTQRQFEWLADIIREVEL--VSVHIYITLAEKFDLRTTMLYICERHFQKRSLFTGLRSITFGRPPFFFLIGVFCGPPGMTKVEKACRTFSHENF

>C.teleta_5_CapteP191097

FLVYLFIFFSICLLVFAFLIAGYGVALTRGAAAGMSFTFSLLLLTMCRNTITRLRETVIIP---FDIFHKIVACAALFFTVVHIVGYCFNFVPWLFNLTGITGILLVLVICIIYIFATATSRRFNAFWITHQFYLLLLIFLHGASRIIPF-LYFIGPGIIYMFDKVISLSRLSVAELLPSGVVIRPVDFSGQVLCDPLEYHALTLTSAPHEETLSVHVRAVGWTLLMYPKLYIDGPFGAGQQDWFEVSILVAGGIGVTPYASILFAFMMKCKKVFIWVTGNQKHFEWFVDIIRQVEI--VDTHIFITMFNSFDLRTMMLYICEENFQRRSTFTGLKAKTFGRPNFIFVVGVFCGPPGLTKVCEASKTFIHENF

>C.intestinalis_7_Duoxb

LIFCLSLYSLITAGVFLALIGGPLMALARASAAALMFNFSTLLLTMCRNIITFLRETFLIP---FDVMHRIVAWMALAFTALHILAHGINFIPWLFTITGITGVILTLALIVMYVFASNYARRFNWFRWTHKLLSLFFSFVHGSGMLIPF-YYFLVPGILFTLDKVYTYSRISVAELFPSDVLFRPKNFAGQVICLAQEYHPFTLSSAPHEDTLKLHIRAVGWTLIYYPKLFLDGPFGEGHQDWYEVSVLVGGGIGVTPFASILLVNRITCKAVFIWVTRDQNQYEWLTDIIQEVEI--LNTHIFITFPQKFDLRTKMLYICEENFQKKSLFTGLRAITFGRPDFFFLFGVFCGPPPMTEVEKACGPFSHENF

>C.intestinalis_6_Duoxb

LIFCLSLYSLITAGVFLALIGGPLMALARASAAALMFNFSTLLLTMCRNIITFLRETFLIP---FDVMHRIVAWMALAFTALHILAHGINFIPWLFTITGITGVILTLALIVMYVFASNYARRFNWFRWTHKLLSLFFSFVHGSGMLIPF-YYFLVPGILFTLDKVYTYSRISVAELFPSDVLFRPKNFAGQVICLAQEYHPFTLSSAPHEDTLKLHIRAVGWTLIYYPKLFLDGPFGEGHQDWYEVSVLVGGGIGVTPFASILLVNRITCKAVFIWVTRDQNQYEWLTDIIQEVEI--LNTHIFITFPQKFDLRTKMLYICEENFQKKSLFTGLRAITFGRPDFFFLFGVFCGPPPMTEVEKACGPFSHENF

>C.intestinalis_3_Duoxb

LIFCLSLYSLITAGVFLALIGGPLMALARASAAALMFNFSTLLLTMCRNIITFLRETFLIP---FDVMHRIVAWMALAFTALHILAHGINFIPWLFTITGITGVILTLALIVMYVFASNYARRFNWFRWTHKLLSLFFSFVHGSGMLIPF-YYFLVPGILFTLDKVYTYSRISVAELFPSDVLFRPKNFAGQVICLAQEYHPFTLSSAPHEDTLKLHIRAVGWTLIYYPKLFLDGPFGEGHQDWYEVSVLVGGGIGVTPFASILLVNRITCKAVFIWVTRDQNQYEWLTDIIQEVEI--LNTHIFITFPQKFDLRTKMLYICEENFQKKSLFTGLRAITFGRPDFFFLFGVFCGPPPMTEVEKACGPFSHENF

>C.intestinalis_4_Duoxa

-IFWTSLYIWITIGVFLALIAGNALPLARASAAALMFNISTLLLTMCKNILTFLRETQLIP---FDVFHKLVAWMALFFTALHIIAHGINFIPWCLTLTGNTGVLLTIIFIVMYVFSLDYPRQFNWFQWIHFFISVFFTVLHGSGMLIPF-YYFLVPAILYTFDKLYSVYRLPVAEILPSDVLFRPSDFAGQVICVGLEYHPFTLSSSPDEETLQLHIRAVGWTIIYYPKLYVDGPFGEGHQDWYEVAVLVGGGIGVTPFASILLVNKIPCKSVFLWVARDQRQFEWLLDIIEETEI--LSTHIFITIPNKFDLRTTMLYVCEQHFKKKSMFTGLNAVTFGRPNFFLLIGVFCGPPSMTEVESACGPYAHENF

>C.intestinalis_5_Duoxa

LIFWTSLYIWITIGVFLALIAGNALPLARASAAALMFNISTLLLTMCKNILTFLRETQLIP---FDVFHKLVAWMALFFTALHIIAHGINFIPWCLTLTGNTGVLLTIIFIVMYVFSLDYPRQFNWFQWIHFFISVFFTVLHGSGMLIPF-YYFLVPAILYTFDKLYSVYRLPVAEILPSDVLFRPSDFAGQVICVGLEYHPFTLSSSPDEETLQLHIRAVGWTIIYYPKLYVDGPFGEGHQDWYEVAVLVGGGIGVTPFASILLVNKIPCKSVFLWVARDQRQFEWLLDIIEETEI--LSTHIFITIPNKFDLRTTMLYVCEQHFKKKSMFTGLNAVTFGRPNFFLLIGVFCGPPSMTEVESACGPYAHENF

>L.anatina_13_Duoxa

FIFYLALFFLITIAIFVSLMAGYGISVTRGAASSMSFTFSVVLLTMCKNTMNLLRTTWVVP---FDFFHKVSAYTSTFIALGHTIGHCINFLPYLFTVTGLTGVVLVVLLTLMIVFATPWARKYTAFWTVHNLVLLALIILHGSAVLVPF-YFYLGPMLLFALDKLVSISNVTVAEHLPSNVILKPRTFSGQAIIVGHEYHPFTITAAEHEDVIAFHIRAVGWTLLMYPKMYIDGPFGGVSEDWSEVAILVGGGIGVTPFAAILVSYLINCKKIFFWVTQNQRQFEWMIDIIREVEL--VTVNVFVTFFGKFDLRTVMMFLCEKHFVSRSLFTGLKAPTFGRPDFVIIFGVFCGPPALTNVSAACSAFSFENF

>D.melanogaster_2_Duox

IIFYLFLFYVVTIVLFVFLIMGVGIAITRGSAASLSFCYSLLLLTMSRNLITKLKEFPIIP---LDIFHKIAACTALFFSVLHTVGHIVNFVPWLFTVTGTTGVMLFIIMCIIFVFAHPTIRKYNFFWNMHTLIGLLLSLIHGLARLTPF-WFFLGPGIVYTLDKIVSLRTLDVTDLLPSDVIFRPPNLSGQVLCTAFEMHSFTLTSAPHENFLSCHIKAQGWTLYFQPKIRIEGPFGGGNQDWYEVAVMVGGGIGVTPYASILLVFGVACKKVFLWICPSHKHFEWFIDVLRDVEV--LEIHIFITFFHKFDLRTTMLYICENHFQRTSIFTGLKAVNFGRPDMFLVIGVFCGPRPLTKVMSACLPFIHENF

>L.anatina_6_Duoxb

IIIYLSFFYLIAIALFISLMVGHGITLTRGAASAMCWTYSVQLLTMCRNTINALRGTVVIP---SDFFHMIVGWTSFGISVFHFVGHMFNFLHYLLTVTGMTGVLLTGVMGIMAVFALPWARRYNIFWRVHKLIILALIILHGSAVLVPF-YFFLGPALLFVLDKTVSLSRVDVSDILPSDVLIKPTGFAGEILIKGYEYHPITIGSAPHEETL---------------KVYIDGPFGSASQDWSDVTVMVGGGIGVTPFASILIIFLISCTKIFVWVSTDQHQFEWMIDIIREVEL--VHISLFITFYKKYDLRTLLMYLCERHFIRRSLLMGLQSPLFGHPNFLLVIGVFYGPPQMVKVAKACKMYEHEVY

>C.elegans_2_Duox

LVFIVFCFVAINLVLFFFLVMGAGIAITRGAAGALSFCMALILLTVCRNIITLLRETVIIP---FDIFHKIVALFAAFWATLHTVGHCVNFVPWFFTITGLTGIALVAVMCIIYVFALPCFIKYHAFRLTHLLIAFALTLLHGLPKLLPF-GYVVGPIVLFVIDRIIGLMQLEIAEILPSDIIYRPREFSGQVVSPSIESHAFSIASSPQDENMKLYIKAVGWTL--FPLIHMKGPYGDGNQEWMEVAIMVGAGIGVTPYASTLLVQRVRCRKVFLWVCSTHKNYEWFVDVLKNVEI--LETHIFVTTFHKFDLRTTMLYICEKHFRAISMFTGLHAKNFGRPNFFFIIGVFCGPVNLNEIAEGCAPFAHETF

>C.elegans_1_Duox

LVFIIFCFVAINIVLFFFLVMGAGIAITLSSAGALSFCMALILLTVCRNIITLLRETVIIP---FDIFHKIVALFTLFWSTLHTIGHCVNFVPWFYTITGLTGIGLVIVMSIIYVFALPKFTRYHAFRLTHLLIGFALTILHGLPSLFPF-GYVVGPIVLFVIDRIIGLMQLDIAEILPSDIIYRPREFSGQIVSPSIESHAFSIASSPQDENMKLYIKAVGWTL--FPLIHMKGPYGDGNQEWMEVAIMVGAGIGVTPYASTLLVQKVRCRKVFLWVCSSHKNFEWFVDMLKNVEI--LETHIFVTMFHKFDLRTTMLYICEKHFRAISMFTGLHAKNFGRPNFFFIIGVFCGPVNLNEIAEGCAPFAHETF

>A.queenslandica_2_NOX5a

LIFCIALWISVNVILFVA-----FVLIARGCGQCLNFNPVIVLSLMMRKCMTWLRSSRVLP---LDILHKLTGYAILFYSTLHFLAHLANFRFYLFGMAGLTGFLLLIIIAIMFICALPFIRRFQVFYWTHNLIAWIILILHGPN----F-WWFAVPAIIYIGEFVLRLKITYIGILLPSKVLIRPGNFPGDVIIPKVEWHPFTISSAPEKGVFWLHIRAVGWTLLFHPEVFFDGPYGSPSVHIFEHAVLIGAGIGVTPFASILIMIRMTLKKVFFWINRHQKYFEWFVSLLSQLEIEFLDMHMYMTALDKTDMKAIGLHMALDLLHKRDLITGLKTRTAGRPNWVFIVTVFCGLPSLGKLQKYC-YFGFENF

>T.wilhelma_3_NOX5a

LVIWLILYIGVNTILFVA-----AVAIARGCGQCLNFNPTFVIIVMMRRGLTWLRSTRVFP---LDILHKMCGWVIFIFAIIHTMAHIVNFL-YFFGTAGITGDILILIITIMVLCSLPCVRRFEVFYWTHFLIVFILLILHATH----F-WWFLVPGIIYILERILRSKWTYIGILLPSKVLMRPTNFPGDVLVPSIEWHPFTISSAPEQGFIWLHIRSVGWTLFFHPEVYIDGPYGTPSAHIFEHAVLIGAGIGVTPFASILIMMRMRLKKVFYWINRDQRSFEWFVSLLSQLEIEFLDMHMYMTALRKTDVKAIGLQMALDLIHKRDLITGLRTRTAGRPDWVFLVTIFCGPPAISNLKTKC-YFEFENF

>T.wilhelma_2_NOX5a

LVIWLILYIGVNTILFVA-----AVAIARGCGQCLNFNPTFVIIVMMRRGLTWLRSTRVFP---LDILHKMCGWVIFIFAIIHTMAHIVNF--YFFGTAGITGDILILIITIMVLCSLPCVRRFEVFYWTHFLIVFILLILHATH----F-WWFLVPGIIYILERILRSKWTYIGILLPSKVLMRPTNFPGDVLVPSIEWHPFTISSAPEQGFIWLHIRSVGWTLFFHPEVYIDGPYGTPSAHIFEHAVLIGAGIGVTPFASILIMMRMRLKKVFYWINRDQRSFEWFVSLLSQLEIEFLDMHMYMTALRKTDVKAIGLQMALDLIHKRDLITGLRTRTAGRPDWVFLVTIFCGPPAISNLKTKC-YFEFENF

>O.carmela_3_NOX5a

-VVAAIIFIIVNAGLFAA-----PISIARGCGMCIDFNSVLMIVLMLRKFLTWLRGTRLLP---LDIFHKIVGMLLAVYSAIHSAAHFVNLVHYLFGTSGISGLVLCVILAVMIICSQPCIRRFEVFYWSHMLVLWLLLILHAQN----F-WWFVVPGLLYSFERIMRSKWTYIGILLPSRVLIRPPGFPGDIIIPEIEWHPFTISSCPEQDVIWLHIRSVGWTLFFHPEIYMDGPYGTPSGHIFEHAVLIGAGIGVTPFASILIIMRMRLKKVFYWINRDQHSFEWFVSLLSQLEIEFLEMHMHMTALKKTDMKAIGLQMALDLIHKRDLITGLKTRTAGRPDWVFILTVFCGSPALSSLKSKC-FFEFENF

>T.adhaerens_1

LTLFMIVYWLTNFGLFGV-----WIKIARGCGLCLNFNCTFIMVLMLRKTLTIIRSTTLLP---IDIFHKMTGIVIGFFALVHTVAHVFNVPRVLFGTAYITGYPLVIILLVMIICSMPFVRRFQVFYWTHLLVPWALLIIHCPN----F-WWFIVPGSIYVLERIYRSKFTYIANMLPSKVLIRPSNFPGDALIPAIEWHPFTISSAPEQHTLWFHVRSVGWTLYFTPKVFIDGPYGTPSTHIFDHAVLIGAGIGVTPFASILIIFRMNLKKVFFWINRDQKAFEWFISLLSQLEIELLDMHMYMTALRKTDMKAIGLQMALDLIHKKDLITGLRTKTAGRPDWVFLVSVFCGSHQLGSLKSYC-YFEFENF

>D.melanogaster_1_NOX5

LVTYLFFYITVNLCLFIA-----FVIIARACGQCLNFNCAWVLVLMLRHSLTYLRGRGLLP---LDVLHKLTGITISVLSLIHTIMHLFNFIYWLLGCANPTGVALLAILVVMFVCSQPFVRRFEVFYWTHLLVPFILCLFHGPN----F-WWFLLPGLVYIVERALRFIWTYIGLLLPSKVLIRPHHFPGDVVIPAIEWHPFTISSAPEQDYMWLHIRTVGWTLYFRPEIFIDGPYGAPSSHIFQHAVLIGTGIGVTPFASILIMHRMNLRKVFFWINRDQRSFEWFVNLLSQLEIEFLDMHMYITALQRTDMKAVGLQLALDLLHERDLITGLKTRTAGRPNWVFLVTVFCGPPQLAKLRYKC-YFAFECF

>A.planci_1_NOX5b

LVIFVIAYCLLNAALFTA-----WIVIARGCGQCLNLNGSLILVLMLRQTLTYVRSTRLLP---IDIFHKTVGLIIAALSLVHSLAHVGNAINVLFHSAFLTGWLLDIILVVMVICSLPFVRRFQVFYWTHLMIFFVLLLMHGPR----F-WWFIIPGIIFILERISQLRLMYVAELMPSGVLIRPPHFPGDLLIPEIEWHPFTISSAPEQDTLSLHIRSAGWTLFF-PEVFINGPYGTATRAIFEHAVLIGAGIGVTPFASILIMLRMRIKKVFIWINRNQKAFEWFVRLLTQLELQFLDMHMFMTALGKTDMKGVGLQMALDIMHSKDMITGLKTRTPGRPDWLFIVQVFCGSPILGKIKAMC-FFHFENF

>S.purpuratus_1_NOX5a

LILFLVVFILINVALFTA-----WLITARGCGQCLNFNSAFVLVLMLRKTITTLRTTKALP---TDIFHKLVGIFIALLSGIHTLGHIGNAVVLLFGSAFLTGWVLDIILAIMVICSMPFVRRFQVFYFTHMLVVFGLLLIHGPR----F-WWFVVPGIIFIVEKLSQTKCTYVVNLLPSGVLLRPNRFAGDIIIPQIEWHPFTISSAPEQGTISMHIRSAGWTLFFDVQVFIDGPYGTATRGIFEHAILVGAGIGVTPFASILIMHRMRLKKVFIWINRNQNAFEWFVSLLTQLEMEFLELHMYMTAMAKNDMKGIGLQMALDIMHKRDLITGLKTRTPGRPDWIFIVQVFCGSPTLAKIKKSC-FFSFENF

>A.queenslandica_3_NOX5b

LFIFLSVFFMINIVLFVA-----LISIARGAGACLNFTPVVVLILMYRHIATLIRSTRLFP---LDILHKVVGYTIIALSLVHFVAHLANFIYYLFGTAGLTGFLLLIILIIMCVCSLPVVRRFEIFYWSHCLIMWIVLILHGPH----F-WWFIGPAIVFIIEKIFRSKLTYIVNLLPSKVLIRPPNFPGDVIIPSIEWHPFTISSAPEMDVFWLHVRGVGWTLHYT----------APSSRDLEFS--------------------------------------------------------------------------------------------------------------------C--------

>A.queenslandica_1NOX5b2

LFIFLSVFFMINTVLFVA-----LVSIARGAGACLNFSPVVVLILMYRHIATLIRSTRLFP---LDILHKIVGYTIIVLSLVHFLAHLANFISYLFGSAGLTGLLLLIILIIMFICSLPVVRRFEVFYWSHCLIIWIVLILHGPH----F-WWFVGPAIVFIIEKIFRSKLTFIVNLLPSKVLIRPPNFPGDVIIPSIEWHPFTISSAPEMDVFWLHIRGVGWTLHFT-EVHIDGPYGTPSSAIFEHAVLISSGIGVTPFASILIMNRLNLKKVFIWINRDQRHFEWFMELLNELEMEFFDMHMYITALQRTDVKALGLQMALDLIHKIDLITGLKTRTTGRPNWIFLVTVFCGSPVLKQLDLQCLFFETNNF

>D.rerio_7_NOX5

LLFFLCLYGLLNTFLFIM-----WIMLARGCGQCLNLNCTFVMVLMLRRCLTWLRATWVLP---LDILHQIVGYAILIFSVGHTGAHIMNFLYYLFGTASITGVVIQILIGLMVVCSSTFVRRFEVFYWSHLSIWVALLVVHCAN----F-WWFVVPGVAFLIEKLVGIAVLYIVNLLPSKVLIRPPFFPGDVIIPTIEWHPFTISSAPEQETLWLHIRSMGWTLYFNPKCYVDGPFGTPTRQIFEHAILIGAGIGITPFASILIMCRMKLRKVFIWINRDQKSFEWFVSLLTKLEMDFLEMHMYMTALSKNDMKAIGLQMALDLLAKRDSITGLRTRTPGRPDWVFVVHVFCGSPALAKIKAQC-FFHFENF

>X.tropicalis_4_NOX5

LLLFMCCYWCLNVLLFGA-----WIMVAKGCGQCLNFNCTFIVVMMLRRCLTWLRTTCVLP---LDVLHELIGYVIFVLTVIHTAAHVTNFLYYLLGTASITGILLQLLICLMLLFSNTFVRKFEVFYWTHLSIWIILLFLHTPK----F-WWFLVPGLLFLLEKLFGAAVVYIVNLLASKVLIRPPSFPGDILIPVIEWHPFTISSAPEQDTIWLHIRSLGWTLYFNPKCYIDGPYGTPTRRIFDHAVLIGAGIGITPFASILIMYRMDLRKVFIWINRDQKFFEWFVSLLTKLELDFLEMHMYMTALSKNDMKAIGLQMALDLLAQKDSITGLRTRTPGRPDWVFIVQVFCGSPALAKIKAHC-FFKFEN-

>H.sapiens_NOX5

LLFCLATYAGLHVLLFGA-----SVMVAKGCGQCLNFDCSFIAVLMLRRCLTWLRATWLLP---LDIFHQLMGYVVVGLSLVHTVAHTVNFQFLLLGSASPTGVALLLLLLLMFICSSSCIRRFEVFYWTHLSLLVLLLIFHGPN----F-WWLLVPGILFFLEKAIGLAVVCIVNLLPSKVLIRPPFFPGDLLIPTIEWHPFTISSAPEQDTIWLHIRSQGWTLSF--KCYIDGPYGTPTRRIFEHAVLIGAGIGITPFASILIMYRMKLHKVFIWINRDQRSFEWFVSLLTKLEMDFLELHMYMTALGKNDMKAIGLQMALDLLANKDSITGLQTRTPGRPDWVFVVQVFCGSPALAKLKGHC-FFRFENF

>H.sapiens_NOX5.2

LLFCLATYAGLHVLLFGA-----SVMVAKGCGQCLNFDCSFIAVLMLRRCLTWLRATWLLP---LDIFHQLMGYVVVGLSLVHTVAHTVNFQFLLLGSASPTGVALLLLLLLMFICSSSCIRRFEVFYWTHLSLLVLLLIFHGPN----F-WWLLVPGILFFLEKAIGLAVVCIVNLLPSKVLIRPPFFPGDLLIPTIEWHPFTISSAPEQDTIWLHIRSQGWTLSF--KCYIDGPYGTPTRRIFEHAVLIGAGIGITPFASILIMYRMKLHKVFIWINRDQRSFEWFVSLLTKLEMDFLELHMYMTALGKNDMKAIGLQMALDLLANKDSITGLQTRTPGRPDWVFVVQVFCGSPALAKLKGHC-FFRFENF

>H.sapiens_NOX5.4

LLFCLATYAGLHVLLFGA-----SVMVAKGCGQCLNFDCSFIAVLMLRRCLTWLRATWLLP---LDIFHQLMGYVVVGLSLVHTVAHTVNFQFLLLGSASPTGVALLLLLLLMFICSSSCIRRFEVFYWTHLSLLVLLLIFHGPN----F-WWLLVPGILFFLEKAIGLAVVCIVNLLPSKVLIRPPFFPGDLLIPTIEWHPFTISSAPEQDTIWLHIRSQGWTLSF--KCYIDGPYGTPTRRIFEHAVLIGAGIGITPFASILIMYRMKLHKVFIWINRDQRSFEWFVSLLTKLEMDFLELHMYMTALGKNDMKAIGLQMALDLLANKDSITGLQTRTPGRPDWVFVVQVFCGSPALAKLKGHC-FFRFENF

>H.sapiens_NOX5.5

LLFCLATYAGLHVLLFGA-----SVMVAKGCGQCLNFDCSFIAVLMLRRCLTWLRATWLLP---LDIFHQLMGYVVVGLSLVHTVAHTVNFQFLLLGSASPTGVALLLLLLLMFICSSSCIRRFEVFYWTHLSLLVLLLIFHGPN----F-WWLLVPGILFFLEKAIGLAVVCIVNLLPSKVLIRPPFFPGDLLIPTIEWHPFTISSAPEQDTIWLHIRSQGWTLSF--KCYIDGPYGTPTRRIFEHAVLIGAGIGITPFASILIMYRMKLHKVFIWINRDQRSFEWFVSLLTKLEMDFLELHMYMTALGKNDMKAIGLQMALDLLANKDSITGLQTRTPGRPDWVFVVQVFCGSPALAKLKGHC-FFRFENF

>H.sapiens_NOX5.6

LLFCLATYAGLHVLLFGA-----SVMVAKGCGQCLNFDCSFIAVLMLRRCLTWLRATWLLP---LDIFHQLMGYVVVGLSLVHTVAHTVNFQFLLLGSASPTGVALLLLLLLMFICSSSCIRRFEVFYWTHLSLLVLLLIFHGPN----F-WWLLVPGILFFLEKAIGLAVVCIVNLLPSKVLIRPPFFPGDLLIPTIEWHPFTISSAPEQDTIWLHIRSQGWTLSF--KCYIDGPYGTPTRRIFEHAVLIGAGIGITPFASILIMYRMKLHKVFIWINRDQRSFEWFVSLLTKLEMDFLELHMYMTALGKNDMKAIGLQMALDLLANKDSITGLQTRTPGRPDWVFVVQVFCGSPALAKLKGHC-FFRFENF

>H.sapiens_NOX5.3

-LFCLATYAGLHVLLFGA-----SVMVAKGCGQCLNFDCSFIAVLMLRRCLTWLRATWLLP---LDIFHQLMGYVVVGLSLVHTVAHTVNFQFLLLGSASPTGVALLLLLLLMFICSSSCIRRFEVFYWTHLSLLVLLLIFHGPN----F-WWLLVPGILFFLEKAIGLAVVCIVNLLPSKTLSGCTFGP--CMSPSRHW-----------------------------------------------AVLRG----------------------------------------------------------------------------------------------------------------C-------V

>C.teleta_1_NOX5
[truncated: 9,852 more chars]
